# Mathematical modeling and application of IL-1β/TNF signaling pathway in regulating chondrocyte apoptosis

**DOI:** 10.3389/fcell.2023.1288431

**Published:** 2023-11-02

**Authors:** Yishu Wang, Jingxiang Liu, Boyan Huang, Xiaojun Long, Xiuyun Su, Deshun Sun

**Affiliations:** ^1^ Medical Research Center, Southern University of Science and Technology Hospital, Shenzhen, China; ^2^ School of Marine Electrical Engineering, Dalian Maritime University, Dalian, China; ^3^ Department of Medical Oncology, Southern University of Science and Technology Hospital, Shenzhen, China; ^4^ Department of Colorectal Surgery, Key Laboratory of Biological Treatment of Zhejiang Province, Sir Run Run Shaw Hospital, School of Medicine, Zhejiang University, Hangzhou, Zhejiang, China; ^5^ Intelligent Medical Innovation Institute, Southern University of Science and Technology Hospital, Shenzhen, China; ^6^ Shenzhen Key Laboratory of Tissue Engineering, Shenzhen Laboratory of Digital Orthopedic Engineering, Guangdong Provincial Research Center for Artificial Intelligence and Digital Orthopedic Technology, Shenzhen Second People’s Hospital (The First Hospital Affiliated to Shenzhen University, Health Science Center), Shenzhen, China

**Keywords:** chondrocyte apoptosis, TNF signal pathway, mathematical model, parameter estimate, sensitive analysis

## Abstract

**Introduction:** Mathematical model can be used to model complex biological processes, and have shown potential in describing apoptosis in chondrocytes.

**Method:** In order to investigate the regulatory mechanisms of TNF signaling pathway in regulating chondrocyte apoptosis, a fractional-order differential equation model is proposed to describe the dynamic behavior and mutual interaction of apoptosis-related genes under the activation of TNF signaling pathway. Compared with the traditional molecular biology techniques, the proposed mathematical modeling has advantages to providing a more comprehensive understanding of the regulatory mechanisms of TNF signaling pathway in chondrocyte apoptosis.

**Result:** In this paper, differentially expressed genes induced by IL-1β in human chondrocyte apoptosis are screened using high-throughput sequencing. It is found that they were significantly enriched in the TNF signaling pathway. Therefore, a mathematical model of the TNF signaling pathway is built. Using real-time PCR experiments, mRNA data is measured and used to identify the model parameters, as well as the correlation coefficient. Finally, the sensitivity of the model parameters is discussed by using numerical simulation methods, which can be used to predict the effects of different interventions and explore the optimal intervention strategies for regulating chondrocyte apoptosis.

**Discussion:** Therefore, fractional-order differential equation modeling plays an important role in understanding the regulatory mechanisms of TNF signaling pathway in chondrocyte apoptosis and its potential clinical applications.

## 1 Introduction

Osteoarthritis (OA) is a chronic degenerative joint disease characterized by articular cartilage destruction, subchondral bone remodeling, osteophyte formation, and inflammatory changes in periarticular tissues, which seriously affects the quality of life of patients. According to statistics, the incidence of osteoarthritis is 50% in people over 50 years old, 80% in people over 60 years old, and the disability rate is as high as 53% (Wang et al., 2019). Osteoarthritis is the first chronic disease that causes disability in adults, and it is also one of the most common diseases in the world (Frank et al., 2015; Yuan et al., 2016). With the aggravation of population aging in China, the prevalence of OA will show an increasing trend, so the research on OA is becoming more and more urgent. In the process of OA, the apoptosis of chondrocytes is the main pathological feature of OA. Studies have found that the content of IL-1β in OA patients is significantly increased. When the content of IL-1β is increased, it will activate the TNF signaling pathway, and TNF-α and IL-1β in the TNF signaling pathway can promote each other to absorb and degrade articular cartilage, aggravating the degree of OA. The higher the content of IL-1β, the more severe the OA, and there is a positive correlation between them.

However, for the treatment of OA, the current goal is mainly to relieve pain and improve joint function, and there is no specific drug. Therefore, exploring an intervention that can effectively delay the progress of OA and improve the OA condition in the early stage is considered to be a very potential treatment strategy that can effectively reduce the development process of OA patients (Yuan et al., 2016). Studies have shown that chondrocyte apoptosis plays a key role in the occurrence and development of osteoarthritis, and its number is positively correlated with the severity of osteoarthritis (Gu et al., 2016; Toshihisa, 2016). Therefore, preventing and reducing chondrocyte apoptosis is an effective method for the treatment of osteoarthritis (Zahoor et al., 2017).

The signal transduction pathway of chondrocyte apoptosis is very complex, and the signal pathways involved mainly include TNF signaling pathway (Wang et al., 2005; Qiu et al., 2007). MAPK signaling pathway (Yoon and Kim, 2004; Ma and Ren, 2012; Gao et al., 2014; Cai and Li, 2019; Liu et al., 2020), JAKS/STAT signaling pathway (Li et al., 2016; Liu et al., 2016), Wnt/β-catenin signaling pathway (Hu et al., 2019; Kalamakis et al., 2019; Liu et al., 2020) and NF-κB signaling pathway (Rigoglou and Papavassiliou, 2013; Ji et al., 2017; Zhao and Gong, 2019) are the hot topics at home and abroad. Tumor necrosis factor-alpha (TNF-α) in the TNF signaling pathway is a cytokine with pleiotropic biological effects. The biological effects of TNF are triggered by two TNF receptors (TNFR) on the cell surface, and its signal transduction pathways mainly include caspase family-mediated apoptosis, adaptor protein TRAF-mediated activation of transcription factor NF-κB and JNK protein kinase (Wang et al., 2005). When TNF-α level is increased, JNK signaling pathway is activated and starts to participate in chondrocyte apoptosis, which also leads to a significant decrease in the expression of apoptosis inhibitor protein Bcl-2 (Yoon and Kim, 2004). In addition, JNK is also involved in inhibiting the expression of Sox-9 and blocking the apoptosis of chondrocytes induced by NO, while SP600125, an inhibitor of JNK, can significantly inhibit the pathological damage of cartilage (Gao et al., 2014), providing another direction for the treatment of OA.

In the IL-1β-induced primary human chondrocytes, baicalin downregulated the mRNA and protein expression of MMP1 and MMP13, and promoted the expression of collagen type II and Aggrecan. Baicalin significantly inhibits cartilage degradation in DMM-induced OA mice, suggesting that breviscapine may be a potential drug for OA treatment (Liu et al., 2020). In addition, breviscapine inhibited the migration of β-catenin and the phosphorylation of p38 into the nucleus, which is associated with the regulation of MAPK signaling pathway.

Studies have found that (Hu et al., 2019), OA chondrocytes were transfected with miR-320c and its inhibitor β-catenin-siRNA, and the results suggested that MiR-320c was decreased and β-catenin was increased in the late stage of OA chondrocytes formation. Overexpression of miR-320c and knockdown of β-catenin had similar effects on cartilage specific gene expression and hypertrophy related gene expression in OA chondrocytes. Injection of mmu-miR-320-3p attenuated OA progression in a mouse model of OA, indicating that miR-320c inhibits osteoarthritis chondrocytes degeneration by inhibiting the canonical Wnt signaling pathway, and miR-320c has the potential as a new drug for osteoarthritis treatment. Hu et al. (2020) reported that pretreatment with Loureirin A significantly inhibited IL-1β-induced production of NO, PGE2, COX-2, TNF-α, iNOS, and IL-6 in mouse articular chondrocytes. In addition, Loureirin A significantly inhibited IL-1β-mediated AKT phosphorylation and NF-κB entry into the nucleus in chondrocytes in signaling pathway studies; therefore, Loureirin A may be a potential therapeutic candidate for OA. Wu et al. (2018) found that after the activation of Notch signaling pathway by the specific activator Jagged1 protein in rat knee joint, Bax protein was upregulated and Bcl-2 protein was downregulated through the apoptotic pathway, thereby promoting chondrocyte apoptosis and aggravating OA. After the Notch signaling pathway was inhibited by the injection of γ-secretase inhibitor DAPT (GSI-IX), the Bax protein was downregulated and the Bcl-2 protein was upregulated through the apoptotic pathway, thereby inhibiting the apoptosis of chondrocytes and alleviating the development of OA.

In summary, the signaling pathways involved in the regulation of articular chondrocyte apoptosis are diverse and extremely complex, but the most significant signaling pathway regulating chondrocyte apoptosis is still unclear, and the most critical signaling molecules in the signaling pathway are also unclear. Therefore, it is urgent to screen the signaling pathways that most significantly affect chondrocyte apoptosis and discover the most critical signaling molecules in this signaling pathway. The mathematical modeling of signaling pathways through high-throughput sequencing is the key to solve the above problems.

Dynamic modeling and analysis of biological systems can simplify the biological system into a mathematical model for analysis and numerical simulation, thus replacing the actual complex, long-term, expensive and even impossible experiments, greatly improving the research efficiency, and studying the influence of artificially imposed control conditions on the operation process of biological systems. For example, infectious disease modeling (Sun and Liu, 2017; Sun and Liu, 2018; Sun et al., 2020) and signaling pathway modeling (Liu et al., 2017), etc. Kinetic modeling based on signaling pathways by relevant scholars mainly includes:

In 2003, Matsuno et al. (2003), based on the Notch signaling pathway to regulate the formation mechanism of *Drosophila* large intestine boundary cells, used Hybrid functional Petri net (HFPN) for modeling. In 2009, Manu et al. (2009) used gene regulatory networks to elucidate interactions between four target genes in the early embryonic period. The equilibrium point, stability and branching of the system were analyzed by using the dynamics theory of ordinary differential equations. Based on the qualitative characteristics of the dynamic system, the complex mechanism of the channel formation and pattern formation of *Drosophila* germ layer was further understood. Immediately following this, Nakamasu et al. (2009) proposed a system of diffusion differential equations with three factors. This model takes into account cell spreading and computationally simulates models of the wild-type pigment production mode as well as other models of mutant production modes.

In the above study, although Manu et al. (2009) calculated the equilibrium point, stability and Hopf branching, they only studied the model theoretically and did not combine the experimental phenomenon with the theoretical results. Although some progress has been made in the system of diffusion differential equations with three factors proposed by Nakamasu et al. (2009), it does not consider that model organism development is susceptible to the influence of external environment such as temperature, especially the interference of random factors. In addition, fluctuations in the level of gene products can generate noise at the molecular level, which not only affects the accuracy of the signal gradient but also reduces the output of the target. Fully considering the above reasons and current situation, the applicant established a deterministic model of *Drosophila* large intestine border cell formation relying on Delta-Notch signaling pathway (Liu et al., 2017), and added white noise to study the effect of random factors on *Drosophila* large intestine border cell formation. The model is as follows:

$$\left\{\begin{array}{l} \frac{dD_{i}}{dt}=\frac{\lambda }{1+\Delta \cdot A_{i}}-d_{1}D_{i}-\sum_{NG\left(i\right)}f_{1}\cdot D_{i},1\leq i\leq NC,\\ \frac{dN_{i}}{dt}=\lambda_{N}-d_{2}N_{i}+\sum_{j\in NG\left(i\right)}f_{2}\cdot D_{j}-\frac{aN_{i}}{bD_{i}+N_{i}},\\ \frac{dA_{i}}{dt}=-d_{3}A_{i}+\frac{aN_{i}}{bD_{i}+N_{i}}. \end{array}\right.$$ (1)

Differential equation dynamics theory was used to study the equilibrium point and its stability when the Delta gene was not expressed and overexpressed. The different phenotypes of the deterministic system are calculated by means of numerical simulations, which are in good agreement with the experimental results.

In addition to the Notch signaling pathway mentioned above, in 2018, Frederik et al. established a corresponding mathematical model based on the mechanism of WNT signaling pathway regulating adult hippocampal nerves, and revealed age-related changes in the nervous system of adult hippocampus (Ziebell et al., 2018; Alahdal et al., 2021). In 2019, Georgios et al. used mathematical kinetic modeling to study that inflammatory signaling and WNT antagonist sFRP5 induce quiescent neural stem cells to regulate the maintenance and regeneration ability of stem cells in the aging brain (Kalamakis et al., 2019).

However, there are few studies on the mathematical modeling of chondrocyte apoptosis based on signal pathways. Accurate mathematical models can quantitatively analyze the mechanism of each signal molecule in the signaling pathway regulating chondrocyte apoptosis at the system level. Therefore, it is particularly urgent to screen the signal pathways that most significantly affect chondrocyte apoptosis, and to establish a kinetic model of chondrocyte apoptosis regulated by this signal pathway from the perspective of mathematical biology.

## 2 High-throughput sequencing and enriched signaling pathway

Knee Human chondrocyte was collected from the Shenzhen Second People’s Hospital (The First Affiliated Hospital of Shenzhen University). Written informed consent was obtained from all patients. The study has been approved by the Shenzhen Second People’s Hospital (The First Affiliated Hospital of Shenzhen University), China. The method to deal with the cartilage in order to obtain the proper chondrocytes is as follows:

After scraping the cartilage tissue with a blade, rinse it three times with physiological saline containing 3% PS. Use a two-step digestion method to separate the articular chondrocytes. Transfer the cartilage tissue to a sterile culture dish and wash it three times with PBS containing 3% PS. Carefully remove any attached soft tissue or synovial fluid, and use ophthalmic scissors to crush it into pieces smaller than 1mm3. Transfer the crushed tissue to a new 50 mL centrifuge tube and add 5 times the volume of 0.25% trypsin. Digest for 20 min and then wash the remaining tissue three times with low glucose DMEM medium. Add 5 times the volume of a solution containing 0.2% type II collagenase and digest gently on a shaking bed at 37°C for 4-5 times, 25 min each time. After each digestion, collect the cells in the supernatant and culture the cells using low glucose DMEM medium containing 20% fetal bovine serum.

**FIGURE 1:**
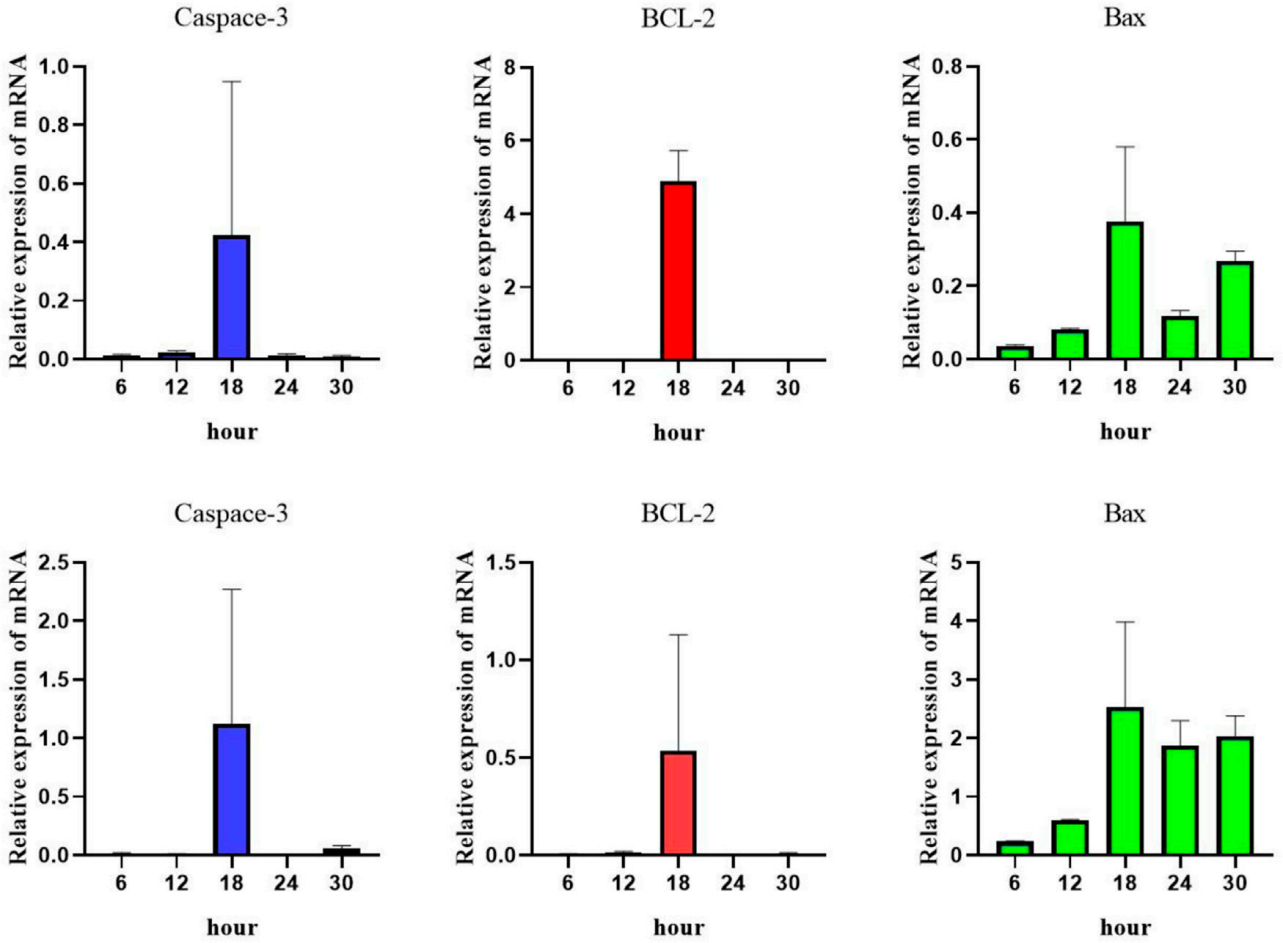
The mRNA expression levels of Caspase-3, BCL-2 and Bax were detected by real-time PCR.

In the early stage, normal chondrocytes were stimulated with IL-1β at a concentration of 10 ng/mL. The results showed that chondrocytes began to apoptosis at 6h, reached the peak at 18h, and then the degree of apoptosis decreased (Figure 1).

Therefore, in this study, the experiments are divided into three groups: 1) normal chondrocytes served as blank control, 2) normal chondrocytes were stimulated with IL-1β (10 ng/mL) for 6 h, and 3) normal chondrocytes were stimulated with IL-1β(10 ng/mL) for 18 h. The cells were collected and subjected to high-throughput sequencing.

The results of sequencing showed that the significant difference value was *p* = 3.21e-09 for TNF signaling pathway, *p* = 5.76e-05 for NF-κβ signaling pathway, *p* = 1.23e-04 for Cytokine-cytokine receptor interaction signaling pathway. The significant difference value for T-cell receptor signaling was *p* = 1.77e-04. The detailed results are shown in Table 1.

**TABLE 1:** High-throughput sequencing Significance table of each signaling pathway affecting chondrocyte apoptosis.

#	Pathway	DEGs in term (256)	All gene in term	Richfactor	*p*-value	QValue	Pathway ID
1	TNF signaling pathway	17(6.64%)	137(1.11%)	0.1241	3.207e-9	8.115e-7	Ko04668
2	NF-kappa B signaling pathway	10 (3.91%)	104 (0.84%)	0.0962	5.764e-5	7.291e-3	Ko04064
3	Cytokine-cytokine receptor interaction	16 (6.25%)	264 (2.14%)	0.0606	0.000123	9.534e-3	Ko04060
4	T cell receptor signaling pathway	11 (4.3%)	142 (1.15%)	0.0775	0.000177	9.534e-3	Ko04660
5	Legionellosis	8 (3.13%)	79 (0.64%)	0.1013	0.000222	9.534e-3	Ko05134
6	Apoptosis	11 (4.3%)	146 (1.18%)	0.0753	0.000226	9.534e-3	Ko04210
7	Epstein-Barr virus infection	14 (5.47%)	229 (1.85%)	0.0611	0.000226	1.079e-2	Ko05169
8	SNARE interaction in vesicular transport	5 (1.95%)	34 (0.28%)	0.1471	0.000299	1.974e-2	Ko04130
9	Pathway in cancer	22 (8.59%)	501 (4.05%)	0.0439	0.000624	2.074e-2	Ko05220
10	MAPK signaling pathway	7 (2.73%)	81 (0.66%)	0.0864	0.000738	3.598e-2	Ko04013
11	Mineral absorption	5 (1.95%)	45 (0.36%)	0.1111	0.001422	5.048e-2	Ko04978
12	Intestinal immune network for IgA production	6 (2.34%)	66 (0.53%)	0.0909	0.002278	5.048e-2	Ko04672
13	Protein processing in endoplasmic reticulum	10 (3.91%)	173 (1.4%)	0.0578	0.003236	6.024e-2	Ko04141
14	Amyotrophic lateral sclerosis	5 (1.95%)	50 (0.4%)	0.1	0.003633	6.024e-2	Ko05014
15	B cell receptor signaling pathway	6 (2.34%)	72 (0.58%)	0.8333	0.003716	6.024e-2	Ko04662
16	Small cell lung cancer	10 (3.91%)	177 (1.43%)	0.0565	0.003809	6.024e-2	Ko05222
17	Non-alcoholic fatty liver disease	9 (3.52%)	155 (1.25%)	0.0580	0.004972	7.400e-2	Ko04932
18	HTLV-1 infection	11 (5.47%)	313 (2.53%)	0.0447	0.005706	8.021e-2	Ko05166

The results of the above high-throughput sequencing suggest that the most significant signaling pathway regulating chondrocyte apoptosis under IL-1β-stimulated conditions is the TNF signaling pathway. Therefore, this project proposes a scientific hypothesis: Through the mathematical modeling of IL-1β/TNF signaling pathway screened by high-throughput sequencing, the regulatory effect of signal molecules in the IL-1β/TNF signaling pathway on chondrocyte apoptosis can be systematically analyzed, and new targets for the treatment of osteoarthritis can be further explored through the sensitivity analysis of parameters.

## 3 Mathematical model

**FIGURE 2:**
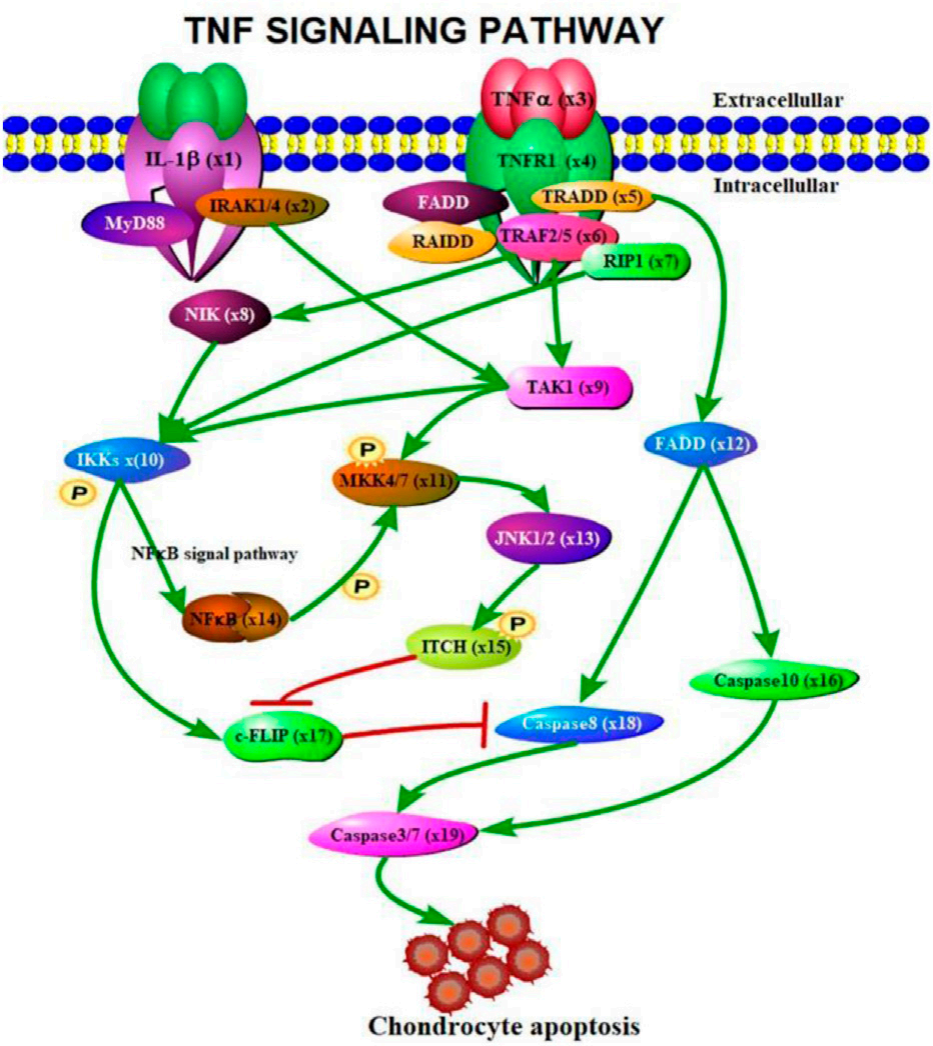
Signal molecule transduction diagram of TNF signaling pathway regulating chondrocyte apoptosis.

According to the signaling molecular transduction mechanism of TNF signaling pathway regulating chondrocyte apoptosis, we use differential equations to model the dynamics of chondrocyte apoptosis. Firstly, the signal molecule transduction map of TNF signaling pathway regulating chondrocyte apoptosis was drawn by Portable Pathway Builder Tool 2.0 (as shown in Figure 2).

Here, $x_{1}\left(t\right),x_{2}\left(t\right),...,x_{n}\left(t\right)$ is utilized to denote the relative concentrations of mRNA for each of the signaling molecule at time t . The differential equation governing changes in signal molecule concentration is expressed as follows.

$$\frac{dx_{i}\left(t\right)}{dt}=v_{i,production}-v_{i,\deg radation}$$ (2)

where $v_{i,production}$ and $v_{i,\deg radation}$ denote the production and degradation rates of the *i*th signaling molecule, respectively. The IL-1β/TNF signaling pathway can be modeled as a system of differential equations, with each signaling molecule’s dynamic changes represented by its own equation. In this paper, the variables $x_{1}\left(t\right)$ , $x_{2}\left(t\right)$ , $x_{3}\left(t\right)$ , $x_{4}\left(t\right)$ , $x_{5}\left(t\right)$ , $x_{6}\left(t\right)$ , $x_{7}\left(t\right)$ , $x_{8}\left(t\right)$ , $x_{9}\left(t\right)$ , $x_{10}\left(t\right)$ , $x_{11}\left(t\right)$ , $x_{12}\left(t\right)$ , $x_{13}\left(t\right)$ , $x_{14}\left(t\right)$ , $x_{15}\left(t\right)$ , $x_{16}\left(t\right)$ , $x_{17}\left(t\right)$ , $x_{18}\left(t\right)$ and $x_{19}\left(t\right)$ represents the relative concentration of mRNA expressed by the signaling molecule IL-1β, IRAK1/4, TNFα, TNFR1, TRADD, TRAF2/5, RIP1, NIK, TAK1, IKKs, MKK4/7, FADD, JNK1/2, NF-κB, ITCH, Caspase10, c-FLIP, Caspase8 and Caspase3/7 at time t , respectively. Parameter $a_{1}$ is used to denote the growth rate of IL-1β expressed mRNA and $d_{1}$ the degradation rate of IL-1β. Therefore, the differential equation about the rate of mRNA change of IL-1β at time t is given as $\frac{dx_{1}\left(t\right)}{dt}=a_{1}-d_{1}x_{1}\left(t\right)$ . Similarly, parameter $a_{2}$ denotes the growth rate of IRAK1/4 mRNA expression, while $b_{2}$ represents the activation rate of IL-1β on IRAK1/4. Furthermore, considering the time delay effect of IL-1β on IRAK1/4 activation, a delay term denoted by τ is incorporated in this study. Additionally, $d_{2}$ signifies the degradation rate of IRAK1/4. Therefore, the differential equation governing the change in mRNA level of IRAK1/4 at given time t is expressed as: $\frac{dx_{2}\left(t\right)}{dt}=a_{2}+b_{2}x_{1}\left(t-\tau \right)x_{2}\left(t-\tau \right)-d_{2}x_{2}\left(t\right)$ . $a_{3}$ and $d_{3}$ represent the growth rate and degradation rate of TNFα, respectively. $a_{4}$ and $d_{4}$ represent the growth rate and degradation rate of TNFR1, $b_{4}$ represents the activation rate of TNFα on TNFR1. $a_{5}$ and $d_{5}$ represent the growth rate and degradation rate of TRADD, $b_{5}$ represents the activation rate of TNFR1 on TRADD. $a_{6}$ and $d_{6}$ represent the growth rate and degradation rate of TRAF2/5, $b_{6}$ represents the activation rate of TRADD on TRAF2/5. $a_{7}$ and $d_{7}$ represent the growth rate and degradation rate of RIP1, $b_{7}$ represents the activation rate of TRAF2/5 on RIP1. $a_{8}$ and $d_{8}$ represent the growth rate and degradation rate of NIK, $b_{8}$ represents the activation rate of TRAF2/5 on NIK. $a_{9}$ and $d_{9}$ represent the growth rate and degradation rate of TAK1, $b_{9}$ and $c_{9}$ represents the activation rate of IRAK1/4 and TRAF2/5 on TAK1. $a_{10}$ and $d_{10}$ represent the growth rate and degradation rate of IKKs, $b_{10}$ , $c_{10}$ and $f_{10}$ represents the activation rate of RIP1, NIK and TAK1 on IKKs, respectively. $a_{11}$ and $d_{11}$ represent the growth rate and degradation rate of MKK4/7, $b_{11}$ and $c_{11}$ represents the activation rate of TAK1 and NF-κB on IKKs. $a_{12}$ and $d_{12}$ represent the growth rate and degradation rate of FADD, $b_{12}$ represents the activation rate of TRADD on FADD. $a_{13}$ and $d_{13}$ represent the growth rate and degradation rate of JNK1/2, $b_{13}$ represents the activation rate of MKK4/7 on JNK1/2. $a_{14}$ and $d_{14}$ represent the growth rate and degradation rate of NF-κB, $b_{14}$ represents the activation rate of IKKs on NF-κB. $a_{15}$ and $d_{15}$ represent the growth rate and degradation rate of ITCH, $b_{15}$ represents the activation rate of JNK1/2 on ITCH. $a_{16}$ and $d_{16}$ represent the growth rate and degradation rate of Caspase10, $b_{16}$ represents the activation rate of FADD on Caspase10. $a_{17}$ and $d_{17}$ represent the growth rate and degradation rate of c-FLIP, $b_{17}$ represents the activation rate of IKKs on c-FLIP and $c_{17}$ represents the inhibiting rate of ITCH on c-FLIP. $a_{18}$ and $d_{18}$ represent the growth rate and degradation rate of Caspase8, $b_{18}$ represents the activation rate of c-FLIP on Caspase8 and $c_{18}$ represents the inhibiting rate of FADD on Caspase8. $a_{19}$ and $d_{19}$ represent the growth rate and degradation rate of Caspase3/7, $b_{19}$ and $c_{19}$ represents the activation rate of Caspase10 and Caspase8 on Caspase3/7. Subsequently, a comprehensive mathematical model for the entire system is established. The time delay of activation or inhibition of downstream signaling molecules varies among different signaling molecules. However, for the sake of convenience in calculation and simulation, a uniform time delay τ is adopted in this study. In subsequent mathematical modeling, different time delays will be utilized. In this paper, the Eq. 2 was operated by Matlab 2020b.

$$\left\{\begin{array}{l} \frac{d^{\alpha }x_{1}\left(t\right)}{dt}=a_{1}-d_{1}x_{1}\left(t\right),\\ \frac{d^{\alpha }x_{2}\left(t\right)}{dt}=a_{2}+b_{2}x_{1}\left(t-\tau \right)x_{2}\left(t-\tau \right)-d_{2}x_{2}\left(t\right),\\ \frac{d^{\alpha }x_{3}\left(t\right)}{dt}=a_{3}-d_{3}x_{3}\left(t\right),\\ \frac{d^{\alpha }x_{4}\left(t\right)}{dt}=a_{4}+b_{4}x_{3}\left(t-\tau \right)x_{4}\left(t-\tau \right)-d_{4}x_{4}\left(t\right),\\ \frac{d^{\alpha }x_{5}\left(t\right)}{dt}=a_{5}+b_{5}x_{4}\left(t-\tau \right)x_{5}\left(t-\tau \right)-d_{5}x_{5}\left(t\right),\\ \frac{d^{\alpha }x_{6}\left(t\right)}{dt}=a_{6}+b_{6}x_{5}\left(t-\tau \right)x_{6}\left(t-\tau \right)-d_{6}x_{6}\left(t\right),\\ \frac{d^{\alpha }x_{7}\left(t\right)}{dt}=a_{7}+b_{7}x_{6}\left(t-\tau \right)x_{7}\left(t-\tau \right)-d_{7}x_{7}\left(t\right),\\ \frac{d^{\alpha }x_{8}\left(t\right)}{dt}=a_{8}+b_{8}x_{6}\left(t-\tau \right)x_{8}\left(t-\tau \right)-d_{8}x_{8}\left(t\right),\\ \frac{d^{\alpha }x_{9}\left(t\right)}{dt}=a_{9}+b_{9}x_{2}\left(t-\tau \right)x_{9}\left(t-\tau \right)+c_{9}x_{6}\left(t-\tau \right)x_{9}\left(t-\tau \right)-d_{9}x_{9}\left(t\right),\\ \frac{d^{\alpha }x_{10}\left(t\right)}{dt}=a_{10}+\left[b_{10}x_{7}\left(t-\tau \right)+c_{10}x_{8}\left(t-\tau \right)+f_{10}x_{9}\left(t-\tau \right)\right]x_{10}\left(t-\tau \right)-d_{10}x_{10}\left(t\right),\\ \frac{d^{\alpha }x_{11}\left(t\right)}{dt}=a_{11}+b_{11}x_{9}\left(t-\tau \right)x_{11}\left(t-\tau \right)+c_{11}x_{14}\left(t-\tau \right)x_{11}\left(t-\tau \right)-d_{11}x_{11}\left(t\right),\\ \frac{d^{\alpha }x_{12}\left(t\right)}{dt}=a_{12}+b_{12}x_{5}\left(t-\tau \right)x_{12}\left(t-\tau \right)-d_{12}x_{12}\left(t\right),\\ \frac{d^{\alpha }x_{13}\left(t\right)}{dt}=a_{13}+b_{13}x_{11}\left(t-\tau \right)x_{13}\left(t-\tau \right)-d_{13}x_{13}\left(t\right),\\ \frac{d^{\alpha }x_{14}\left(t\right)}{dt}=a_{14}+b_{14}x_{10}\left(t-\tau \right)x_{14}\left(t-\tau \right)-d_{14}x_{14}\left(t\right),\\ \frac{d^{\alpha }x_{15}\left(t\right)}{dt}=a_{15}+b_{15}x_{13}\left(t-\tau \right)x_{15}\left(t-\tau \right)-d_{15}x_{15}\left(t\right),\\ \frac{d^{\alpha }x_{16}\left(t\right)}{dt}=a_{16}+b_{16}x_{12}\left(t-\tau \right)x_{16}\left(t-\tau \right)-d_{16}x_{16}\left(t\right),\\ \frac{d^{\alpha }x_{17}\left(t\right)}{dt}=a_{17}+b_{17}x_{10}\left(t-\tau \right)x_{17}\left(t-\tau \right)-c_{17}x_{15}\left(t-\tau \right)x_{17}\left(t-\tau \right)-d_{17}x_{17}\left(t\right),\\ \frac{d^{\alpha }x_{18}\left(t\right)}{dt}=a_{18}+b_{18}x_{17}\left(t-\tau \right)x_{18}\left(t-\tau \right)-c_{18}x_{12}\left(t-\tau \right)x_{18}\left(t-\tau \right)-d_{18}x_{18}\left(t\right),\\ \frac{d^{\alpha }x_{19}\left(t\right)}{dt}=a_{19}+b_{19}x_{16}\left(t-\tau \right)x_{19}\left(t-\tau \right)+c_{19}x_{18}\left(t-\tau \right)x_{19}\left(t-\tau \right)-d_{19}x_{19}\left(t\right). \end{array}\right.$$ (3)

## 4 Real-time PCR experiment

### 4.1 Extract total RNA by Trizol method

(1) Pre-processing of samples

Cell: Add 1 mL Trizol reagent, mix thoroughly by pipetting, transfer to a RNase-free 1.5 mL EP tube, and incubate for 10 min to lyse the cells.

(2) Add 200 μL chloroform, vigorously shake the tube several times, and let it stand at room temperature for 5 min.
(3) Centrifuge at 4°C and 12,000 rpm for 15 min to separate the sample into three phases: the upper phase (RNA), the middle phase (protein), and the lower phase (DNA).
(4) Transfer the upper aqueous phase (approximately 400 μL) to a new 1.5 mL EP tube, add 400 μL isopropanol, mix thoroughly, and let it stand at room temperature for 10 min.
(5) Centrifuge at 4°C and 12,000 rpm for 10 min, and a white RNA pellet should be visible at the bottom of the tube.
(6) Discard the supernatant, add 1 mL 75% ethanol without RNase, vortex, and centrifuge at 4°C and 10,000 rpm for 5 min.
(7) Repeat step 6 once.
(8) Discard the supernatant, air-dry the RNA pellet for 5–10 min, and dissolve the pellet in 20 μL DEPC water.
(9) Measure the OD260, OD280, and OD260/OD280 values of the RNA using a micro spectrophotometer, and calculate the purity and concentration of the RNA. Estimate the RNA quality based on the OD260/OD280 ratio, which should be between 1.8 and 2.0 for experimental requirements. Calculate the total RNA concentration (μg/μL) using the following formula: Total RNA concentration = OD260 × 40×10^−3^.
(10) Store the total RNA at −80°C for future use.

### 4.2 Reverse transcribed into cDNA

#### 4.2.1 Genomic DNN removal reaction

Melt the RNA template, NASe-free Water, and 10× gDNA Remover Buffer on ice. In a nuclease-free micro centrifuge tube, prepare a reaction system (10 μL) on ice according to the table below. The reaction system is shown in Supplementary Table S1.

Subsequently, the mixed system was blown and briefly centrifuged using a pipettor, and the reaction was carried out on a PCR instrument. The procedure is given as follows:

The cells were incubated at 42°C for 2 min and at 60°C for 5 min.

#### 4.2.2 Reverse transcription reaction

Following the preceding reaction, the system was rapidly chilled on ice and subsequently subjected to a brief centrifugation. The addition of specific components was then carried out based on the desired detection index (Supplementary Tables S2, S3).

Subsequently, employing a liquid motion beat blending system and brief centrifugation, the sample was added to the PCR reaction with the following program: incubation at 25°C for 10 min, followed by incubation at 50°C for 15 min and then further incubated at 85°C for an additional 5 min. The reverse transcription product was subsequently placed on ice or refrigerated as required.

### 4.3 Real-time PCR detection

The Real-time PCR reaction system is shown in Table 2. Subsequently, the mixed system was blown with a pipette and briefly centrifuged, and then placed on a fluorescent quantitative PCR instrument for amplification detection, the procedure is given in Supplementary Table S5.

**TABLE 2:** Real-time PCR reaction system.

Reagent	10 (μL)system	20 (μL)system	Final concentration
cDNA template	1	2	
Forward Primer (10 μM)	0.4	0.8	0.4 μM
Reverse Primer (10 μM)	0.4	0.8	0.4 μM
2×TSINGKE^®^ Matser qPCR Mix (SYBR Green I)	5	10	1×
50×ROX Reference Dye II	0.2	0.4	1×
ddH_2_O	3	6	

The operation data was obtained, and the final data was analyzed by 2^-△△Ct^ method (as shown in Table 3). A more intuitive bar graph is shown in Supplementary Figure S1.

**TABLE 3:** Gene relative expression of IRAK1 (*x*_2_), TNF-α (*x*_3_), TRADD (*x*_5_), RIP1(*x*_7_), NIK (*x*_8_), TAK1 (*x*_9_), IKK-β (*x*_10_), MKK4(*x*_11_), FADD (*x*_12_), ITCH (*x*_15_), Caspase-8 (*x*_18_) and Caspase-3 (*x*_19_) with the interval was 6 h from 0 h to 72 h.

(h)	IRAK1	TNFα	TRADD	RIP1	NIK	TAK1	IKKβ	MKK4	FADD	ITCH	Caspase8	Caspase3
	(*x*_2_)	(*x*_3_)	(*x*_5_)	(*x*_7_)	(*x*_8_)	(*x*_9_)	(*x*_10_)	(*x*_11_)	(*x*_12_)	(*x*_15_)	(*x*_18_)	(*x*_19_)
0	1.00 ± 0.073	1.00 ± 0.104	1.00 ± 0.071	1.00 ± 0.098	1.00 ± 0.062	1.00 ± 0.06	1.00 ± 0.069	1.00 ± 0.066	1.00 ± 0.077	1.00 ± 0.098	1.00 ± 0.09	1.00 ± 0.051
6	1.01 ± 0.106	1.00 ± 0.04	1.01 ± 0.051	1.18 ± 0.116	1.63 ± 0.09	1.87 ± 0.081	0.97 ± 0.096	1.18 ± 0.051	1.20 ± 0.12	1.03 ± 0.097	0.80 ± 0.042	1.04 ± 0.13
12	14.49 ± 1.397	19.89 ± 1.524	12.40 ± 1.6	1.09 ± 0.097	2.28 ± 0.126	2.20 ± 0.108	0.99 ± 0.051	1.36 ± 0.08	11.67 ± 0.512	1.52 ± 0.017	1.04 ± 0.075	1.11 ± 0.119
18	21.28 ± 2.411	30.26 ± 2.36	12.60 ± 1.05	1.28 ± 0.107	2.39 ± 0.072	2.38 ± 0.207	1.15 ± 0.056	1.38 ± 0.02	16.06 ± 0.529	1.42 ± 0.142	1.23 ± 0.1	1.02 ± 0.085
24	22.71 ± 0.371	38.05 ± 2.385	21.88 ± 0.97	1.56 ± 0.105	2.36 ± 0.218	2.12 ± 0.105	1.90 ± 0.085	1.19 ± 0.061	20.00 ± 2.3	1.43 ± 0.11	1.30 ± 0.14	1.00 ± 0.08
30	58.85 ± 4.031	36.51 ± 2.032	21.06 ± 2.221	2.30 ± 0.075	2.42 ± 0.11	3.37 ± 0.195	2.01 ± 0.146	1.18 ± 0.11	19.77 ± 1.451	1.96 ± 0.128	1.41 ± 0.094	0.95 ± 0.093
36	18.72 ± 0.953	37.50 ± 1.059	26.89 ± 1.846	1.87 ± 0.052	1.54 ± 0.141	1.60 ± 0.159	2.43 ± 0.256	1.72 ± 0.094	15.76 ± 1.773	0.73 ± 0.077	1.22 ± 0.147	1.01 ± 0.046
42	15.79 ± 0.829	14.28 ± 0.979	15.03 ± 1.029	2.24 ± 0.116	2.47 ± 0.118	3.33 ± 0.257	1.90 ± 0.082	1.90 ± 0.071	14.26 ± 0.852	0.93 ± 0.084	0.87 ± 0.047	1.05 ± 0.127
48	14.43 ± 0.606	12.98 ± 1.364	9.67 ± 0.905	1.81 ± 0.142	3.21 ± 0.202	3.08 ± 0.213	2.10 ± 0.232	2.41 ± 0.11	12.49 ± 0.973	1.21 ± 0.132	0.98 ± 0.035	1.12 ± 0.033
54	13.77 ± 0.453	8.70 ± 0.259	6.30 ± 0.445	1.47 ± 0.133	3.81 ± 0.294	6.89 ± 0.39	1.53 ± 0.176	2.33 ± 0.103	6.41 ± 0.278	1.83 ± 0.198	1.34 ± 0.052	1.40 ± 0.115
60	6.59 ± 0.487	7.54 ± 0.752	7.24 ± 0.963	1.45 ± 0.165	3.43 ± 0.186	3.75 ± 0.226	1.19 ± 0.071	1.36 ± 0.108	6.50 ± 0.661	1.43 ± 0.108	0.97 ± 0.136	1.71 ± 0.176
66	5.08 ± 0.684	6.02 ± 0.703	4.70 ± 0.437	1.27 ± 0.071	1.74 ± 0.077	2.36 ± 0.212	1.18 ± 0.135	1.17 ± 0.091	3.46 ± 0.222	1.51 ± 0.116	1.02 ± 0.104	1.28 ± 0.138
72	5.44 ± 0.451	5.26 ± 0.522	4.85 ± 0.491	1.08 ± 0.095	1.73 ± 0.038	1.81 ± 0.147	1.06 ± 0.104	1.12 ± 0.104	3.24 ± 0.318	1.36 ± 0.153	1.17 ± 0.093	1.30 ± 0.05

## 5 Parameter estimate and numerical simulations

According to the expression of various signaling molecules in the TNF signaling pathway, we divided parameter estimation into three stages. The first stage was from 0^th^ h to 36th h, the second stage was from 36th h to 60th h, and the third stage was from 60th h to 72nd h. The nonlinear least square method was programmed to estimate the 62 parameters by Matlab 2020b and the parameters of stage 1 are as follows (see Table 4):

**TABLE 4:** The estimated parameters of stage 1 for numerical simulation.

Param	Value	Param	Value	Param	Value	Param	Value	Param	Value
$a_{1}$	0.0139	$a_{6}$	0.92	$a_{10}$	1.0549	$b_{13}$	0.684	$g_{17}$	0.1843
$d_{1}$	0.1028	$b_{6}$	0.04	$b_{10}$	0.15	$d_{13}$	2.4042	$d_{17}$	2.4042
$a_{2}$	0.08	$d_{6}$	1.2661	$g_{10}$	0.25	$a_{14}$	0.093	$a_{18}$	0.5
$b_{2}$	0.51	$a_{7}$	1.15	$f_{10}$	0.1395	$b_{14}$	0.2843	$b_{18}$	0.8
$d_{2}$	0.105	$b_{7}$	0.27	$d_{10}$	1.9	$d_{14}$	2.4042	$g_{18}$	0
$a_{3}$	8.254	$d_{7}$	1.1789	$a_{11}$	0.99	$a_{15}$	1.07	$d_{18}$	1.4042
$d_{3}$	0.660	$a_{8}$	1.2	$b_{11}$	0.2	$b_{15}$	1.2	$a_{19}$	2.4
$a_{4}$	2.117	$b_{8}$	0.2	$g_{11}$	0.2	$d_{15}$	2.4042	$b_{19}$	0.2843
$b_{4}$	0.017	$d_{8}$	0.9	$d_{11}$	1.2661	$a_{16}$	0.093	$g_{19}$	0.33
$d_{4}$	1.9	$a_{9}$	2.1655	$a_{12}$	5.6	$b_{16}$	0.1	$d_{19}$	2.0
$a_{5}$	1.2639	$b_{9}$	0.02663	$b_{12}$	0.073	$d_{16}$	2.4042		
$b_{5}$	0.06	$g_{9}$	0.2663	$d_{12}$	1.8	$a_{17}$	2.093		
$d_{5}$	0.13	$d_{9}$	1.8661	$a_{13}$	2.293	$b_{17}$	0.843		

The parameters of stage 2 are as follows (see Table 5):

**TABLE 5:** The estimated parameters of stage 2 for numerical simulation.

Param	Value	Param	Value	Param	Value	Param	Value	Param	Value
$a_{1}$	0.0139	$a_{6}$	0.92	$a_{10}$	1.95	$b_{13}$	0.6843	$g_{17}$	0.1843
$d_{1}$	0.1028	$b_{6}$	0.04	$b_{10}$	0.1	$d_{13}$	2.4042	$d_{17}$	2.4042
$a_{2}$	0.08	$d_{6}$	0.7661	$g_{10}$	0.1	$a_{14}$	0.093	$a_{18}$	0.9
$b_{2}$	0.46	$a_{7}$	1.45	$f_{10}$	0.12	$b_{14}$	0.2843	$b_{18}$	0.7
$d_{2}$	0.105	$b_{7}$	0.064	$d_{10}$	1.9	$d_{14}$	2.4042	$g_{18}$	0.01
$a_{3}$	6.9	$d_{7}$	1.12	$a_{11}$	0.99	$a_{15}$	1.5	$d_{18}$	1.95
$d_{3}$	0.960	$a_{8}$	0.072	$b_{11}$	0.36	$b_{15}$	0.46	$a_{19}$	2.6
$a_{4}$	2.117	$b_{8}$	0.04	$g_{11}$	0.22	$d_{15}$	2.4042	$b_{19}$	2.88
$b_{4}$	0.017	$d_{8}$	0.2	$d_{11}$	1.12	$a_{16}$	0.093	$g_{19}$	0.001
$d_{4}$	1.9	$a_{9}$	2.1655	$a_{12}$	5.6	$b_{16}$	0.143	$d_{19}$	2.5
$a_{5}$	1.2639	$b_{9}$	0.03663	$b_{12}$	0.079	$d_{16}$	2.4042		
$b_{5}$	0.03	$g_{9}$	0.1863	$d_{12}$	1.8	$a_{17}$	2.093		
$d_{5}$	0.23	$d_{9}$	1.9661	$a_{13}$	2.293	$b_{17}$	0.843		

The parameters of stage 3 are as follows (see Table 6):

**TABLE 6:** The estimated parameters of stage 3 for numerical simulation.

Param	Value	Param	Value	Param	Value	Param	Value	Param	Value
$a_{1}$	0.0139	$a_{6}$	0.92	$a_{10}$	1.3	$b_{13}$	0.6843	$g_{17}$	0.2843
$d_{1}$	0.1028	$b_{6}$	0.04	$b_{10}$	0.1	$d_{13}$	2.4042	$d_{17}$	1.4042
$a_{2}$	0.08	$d_{6}$	0.7661	$g_{10}$	0.1	$a_{14}$	0.093	$a_{18}$	0.4
$b_{2}$	0.4	$a_{7}$	1.45	$f_{10}$	0.12	$b_{14}$	0.2843	$b_{18}$	0.78
$d_{2}$	0.115	$b_{7}$	0.056	$d_{10}$	1.8	$d_{14}$	2.4042	$g_{18}$	0.01
$a_{3}$	6.554	$d_{7}$	1.252	$a_{11}$	0.5	$a_{15}$	2.8	$d_{18}$	1.45
$d_{3}$	1.160	$a_{8}$	0.072	$b_{11}$	0.23	$b_{15}$	0.5	$a_{19}$	2.6
$a_{4}$	2.117	$b_{8}$	0.07	$g_{11}$	1.1663	$d_{15}$	2.4042	$b_{19}$	2.88
$b_{4}$	0.017	$d_{8}$	0.2	$d_{11}$	1.0	$a_{16}$	0.093	$g_{19}$	0.001
$d_{4}$	1.9	$a_{9}$	0.00655	$a_{12}$	6.6	$b_{16}$	0.2843	$d_{19}$	2.2
$a_{5}$	1.2639	$b_{9}$	0.008663	$b_{12}$	0.071	$d_{16}$	2.4042		
$b_{5}$	0.03	$g_{9}$	0.08663	$d_{12}$	1.95	$a_{17}$	2.093		
$d_{5}$	0.33	$d_{9}$	0.3	$a_{13}$	0.293	$b_{17}$	0.2843		

**FIGURE 3:**
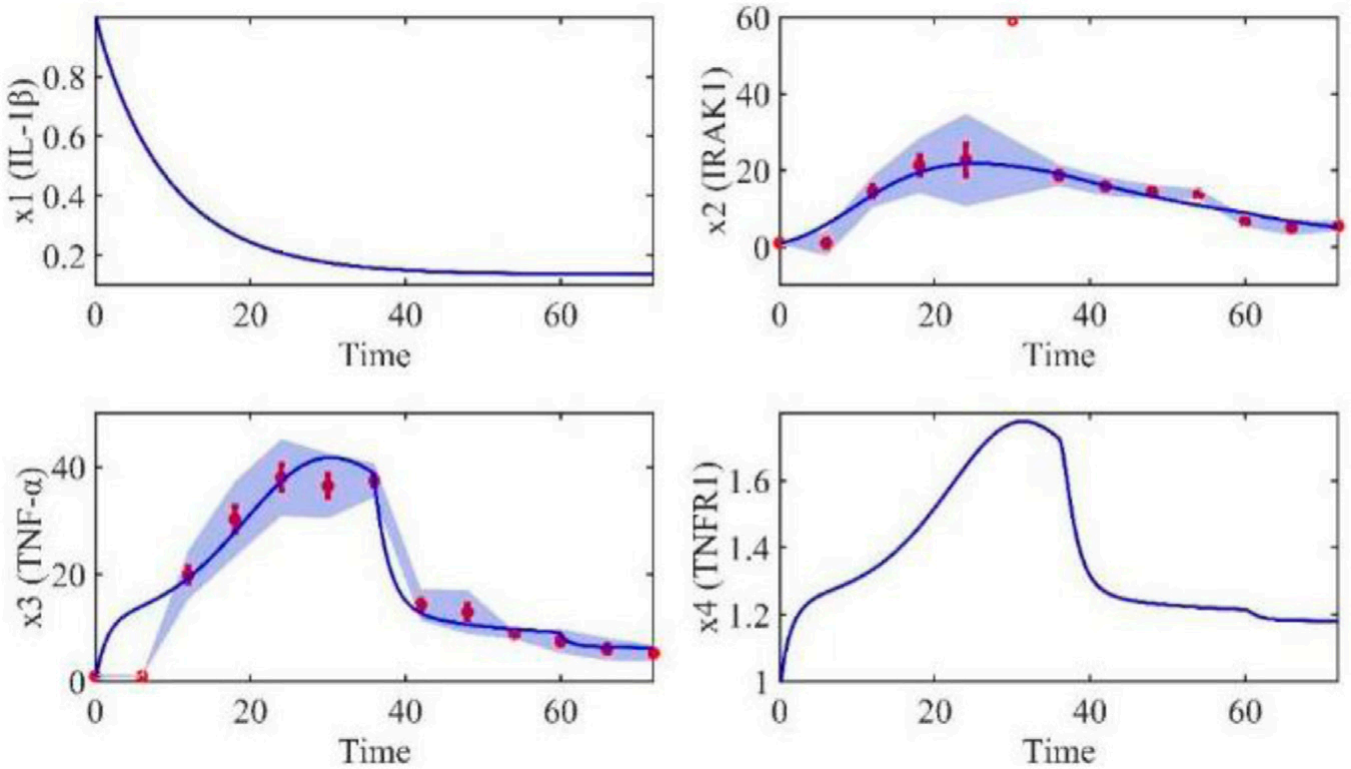
The gene relative expression of IL-1β, IRAK1, TNF-α and TNFR1 from 0 h to 72 h.

**FIGURE 4:**
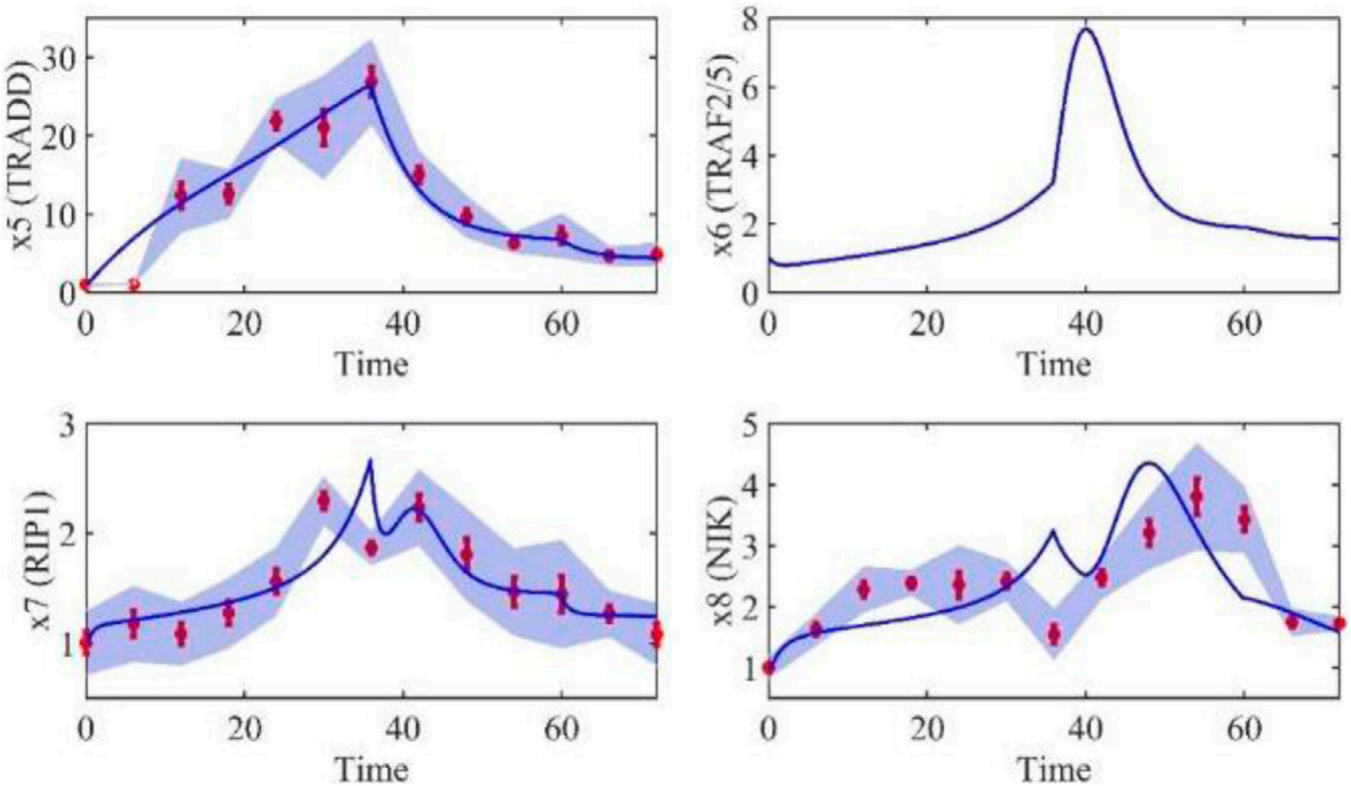
The gene relative expression of TRADD, TRAF2/5, RIP1 and NIK from 0 h to 72 h.

**FIGURE 5:**
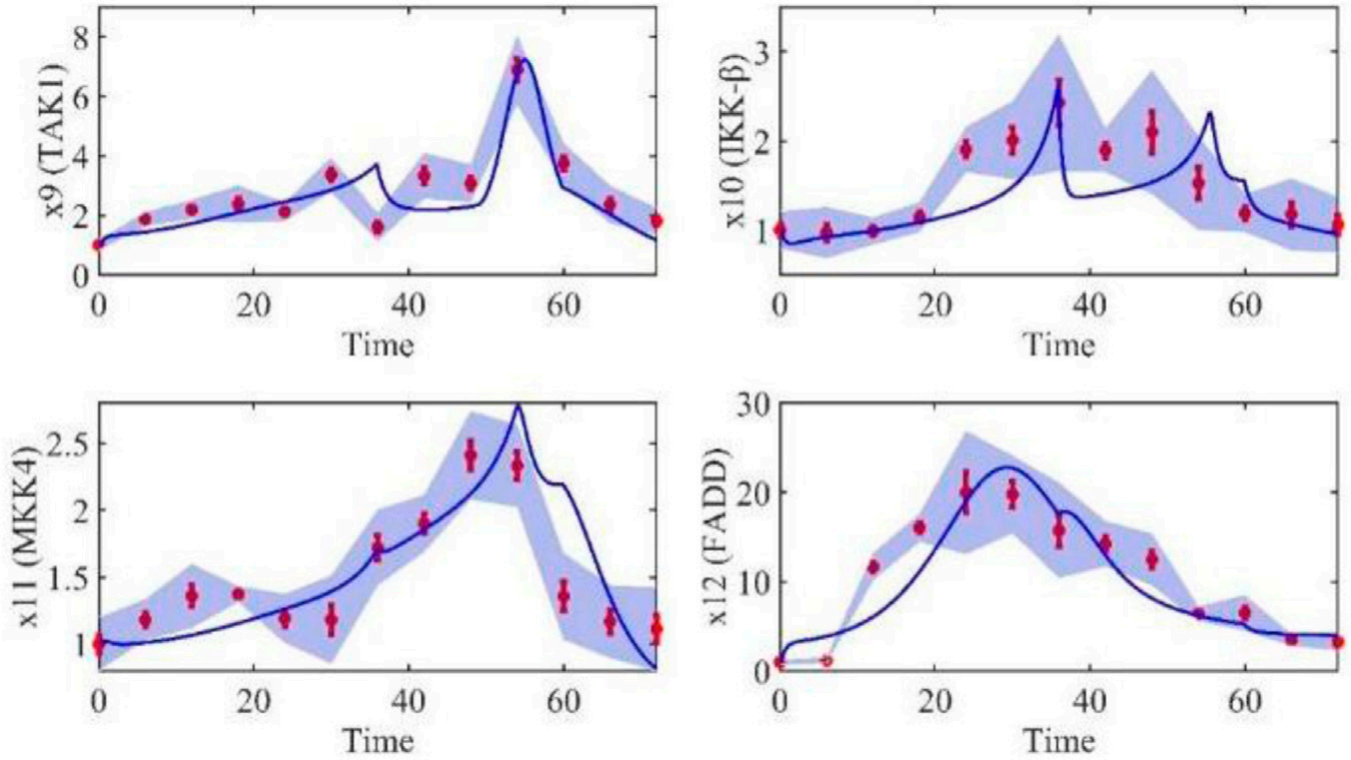
The gene relative expression of TAK1, IKK-β, MKK4 and FADD from 0 h to 72 h.

**FIGURE 6:**
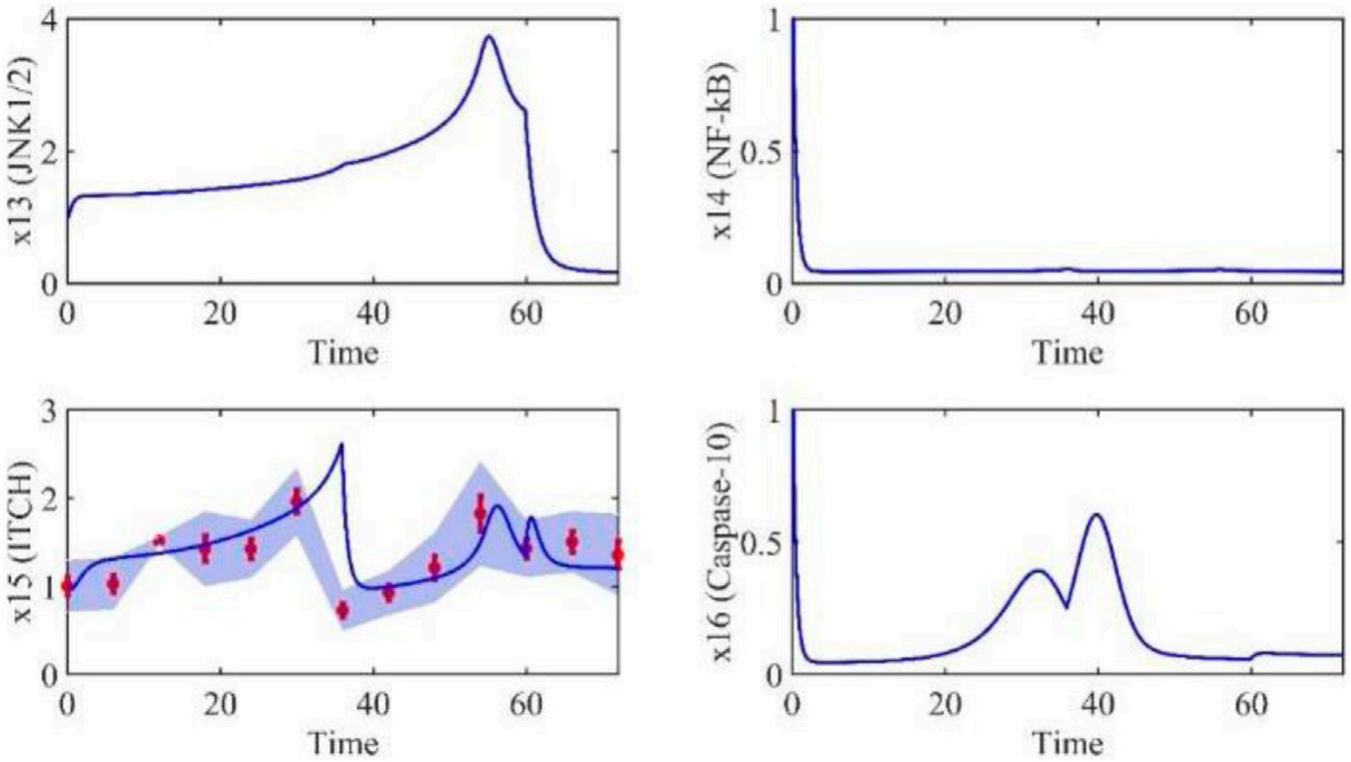
The gene relative expression of JNK1/2, NF-kB, ITCH and Caspase-10 from 0 h to 72 h.

**FIGURE 7:**
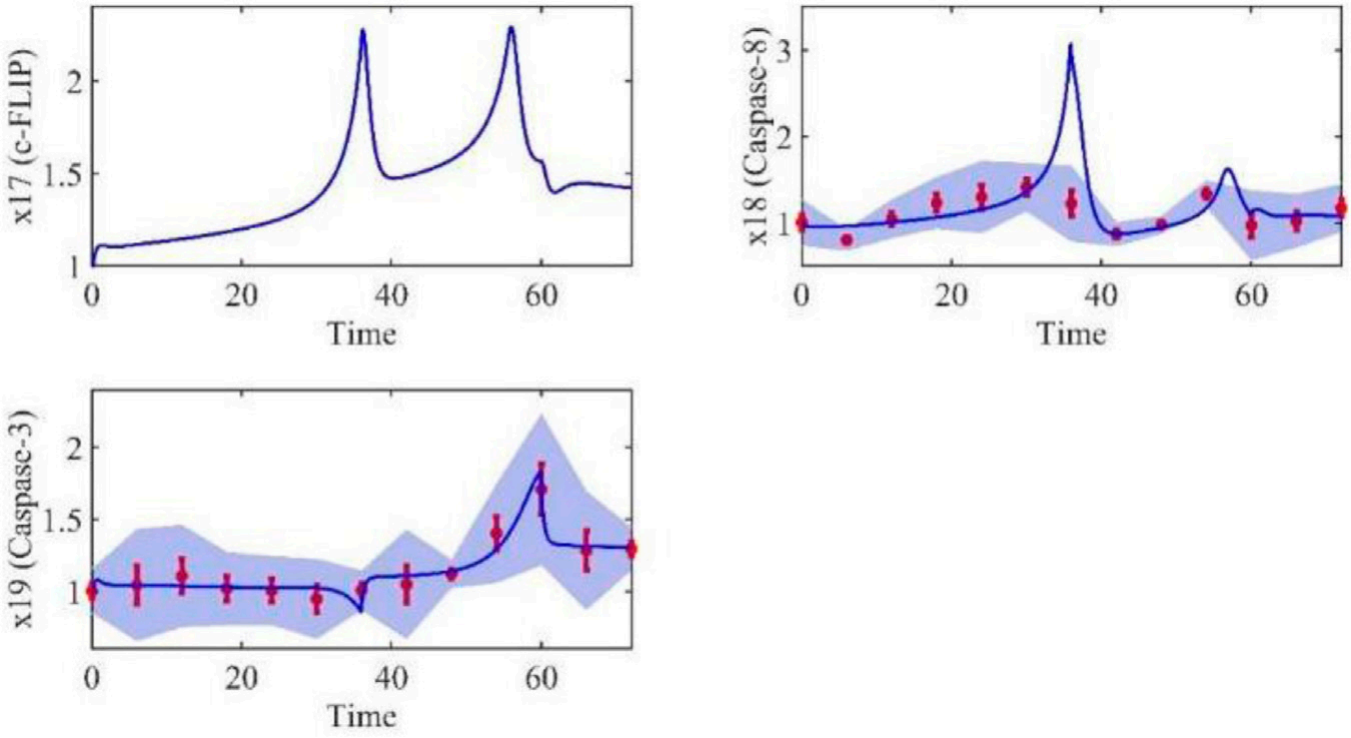
The gene relative expression of c-FLIP, Caspase-8 and Caspase-3 from 0 h to 72 h.

Based on the model parameters obtained from three stages and combined with mathematical model (2), we obtained simulation results of 19 genes, as shown in Figures 3–7. The red dots with error bars represent the real experimental data, while the deep blue curves are numerical simulations of the model, and the light blue areas are three times variance of fitted curves.

The correlation coefficient (R) was calculated to compare the experimental findings with model stimulation. The results, as presented in Table 7, demonstrate that all tested variables exhibited relatively strong correlations, indicating the efficiency of the model.

**TABLE 7:** The correlation coefficient between experimental data and numerical simulation.

*R*_*2*_	*R*_*3*_	*R*_*5*_	*R*_*7*_	*R*_*8*_	*R*_*9*_	*R*_*10*_	*R*_*11*_	*R*_*12*_	*R*_*15*_	*R*_*18*_	*R*_*19*_
**0.969**	0.957	0.963	0.934	0.761	0.858	**0.750**	0.821	0.917	0.813	0.837	0.962

The bold values 0.969 means the largest correlation coefficient, 0.750 means the smallest correlation coefficient.

## 6 The sensitivity analysis of molecules based on the mathematical model

Based on the accurate mathematical model obtained, the influence of model parameter changes on the system will be studied next, that is, the sensitivity of parameters in the model will be analyzed. Given the positive correlation between chondrocyte apoptosis and osteoarthritis severity, this project aims to employ gene interference experiments to silence related genes while making subtle adjustments to model parameters that significantly impact output. The ultimate goal is to reduce chondrocyte apoptosis and identify new targets and strategies for treating osteoarthritis. In this section, sensitivity of molecules based on the mathematical model was analyzed by numerical simulations. Because the severity of chondrocyte apoptosis is positively correlated with the severity of osteoarthritis, this project aims to greatly change the output of the model by making subtle adjustments to the sensitive parameters of the model, that is, to reduce chondrocyte apoptosis, and to provide new targets and strategies for the treatment of osteoarthritis. Therefore, in this study, the degree of change in the mRNA expression of the gene Caspase-3 was used as an indicator of parameter sensitivity. The simulation results show that $b_{4}$ , $a_{5}$ , $d_{5}$ , $a_{6}$ , $a_{10}$ , $b_{10}$ , $g_{10}$ , $f_{10}$ , $d_{10}$ , $b_{12}$ , $d_{12}$ , $a_{16}$ , $b_{16}$ , $d_{16}$ , $a_{17}$ , $a_{18}$ , $b_{18}$ , $a_{19}$ , $b_{19}$ , $g_{19}$ , $d_{19}$ are sensitive parameters and others are insensitive parameters. Below we randomly selected a sensitive parameter ( $a_{5}$ ) and demonstrated its simulation results. The results are as follows:

Similarly, an insensitive parameter ( $b_{7}$ ) was randomly selected and the simulation results were demonstrated. The results are shown in Supplementary Figures S2–S6.

**FIGURE 8:**
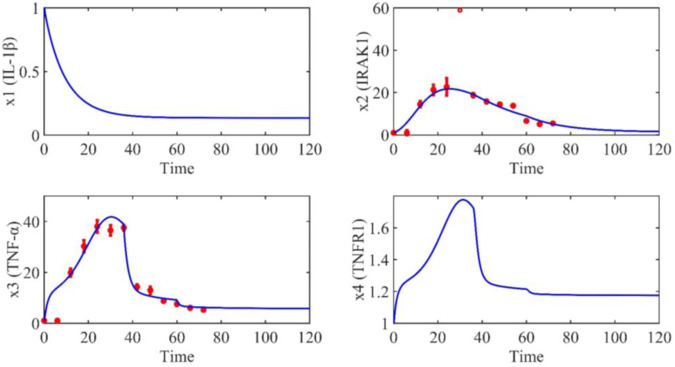
The dynamic trend of IL-1β, IRAK1, TNF-α and TNFR1 from 0 h to 72 h when the parameter $a_{5}$ increases from 0.6 to 3.8, and the interval is 0.8.

**FIGURE 9:**
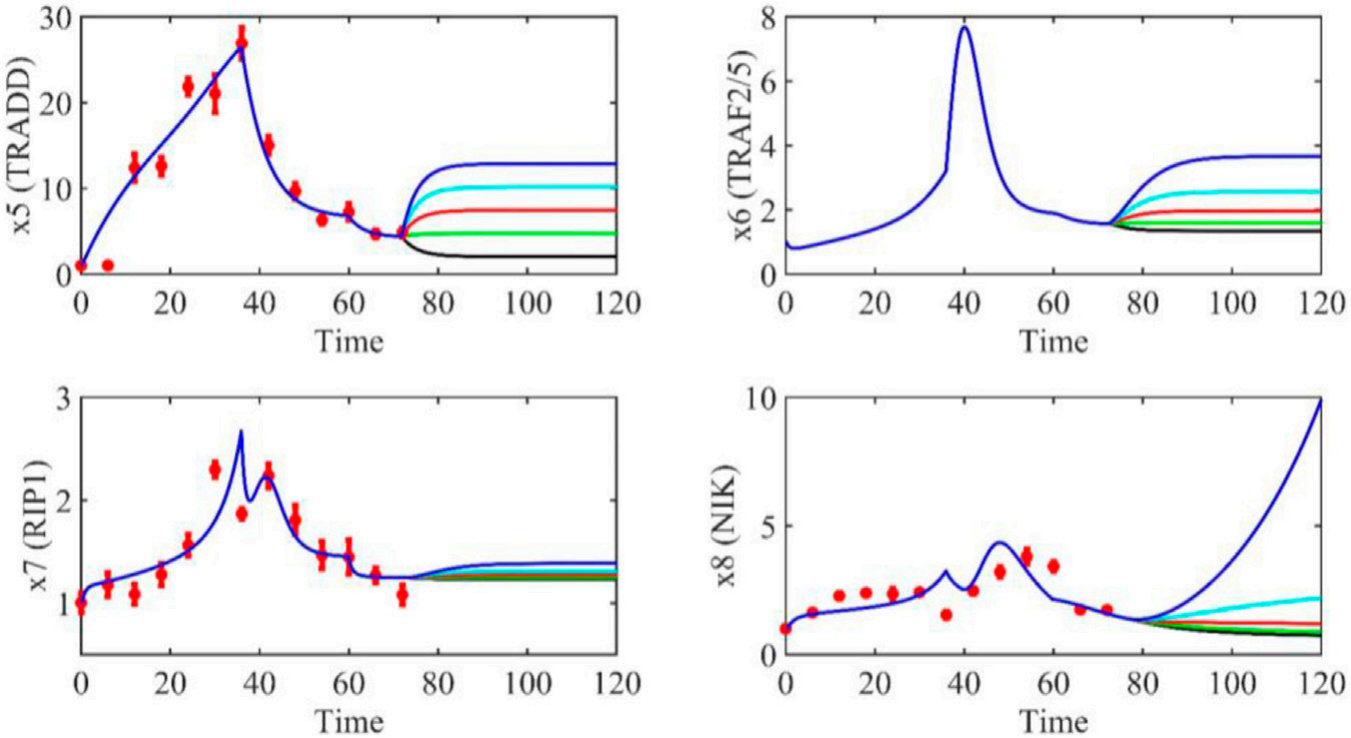
The dynamic trend of TRADD, TRAF2/5, RIP1 and NIK from 0 h to 72 h when the parameter $a_{5}$ increases from 0.6 to 3.8, and the interval is 0.8.

**FIGURE 10:**
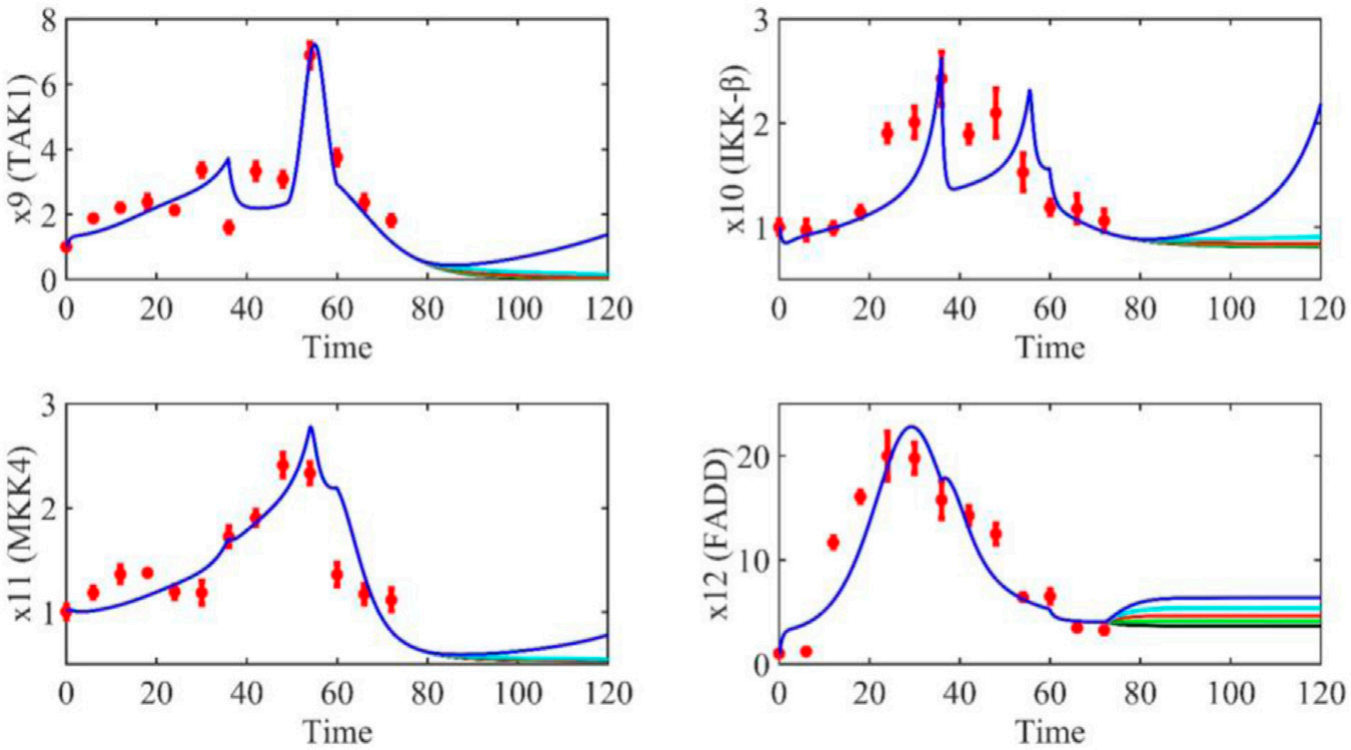
The dynamic trend of TAK1, IKK-β, MKK4 and FADD from 0 h to 72 h when the parameter $a_{5}$ increases from 0.6 to 3.8, and the interval is 0.8.

**FIGURE 11:**
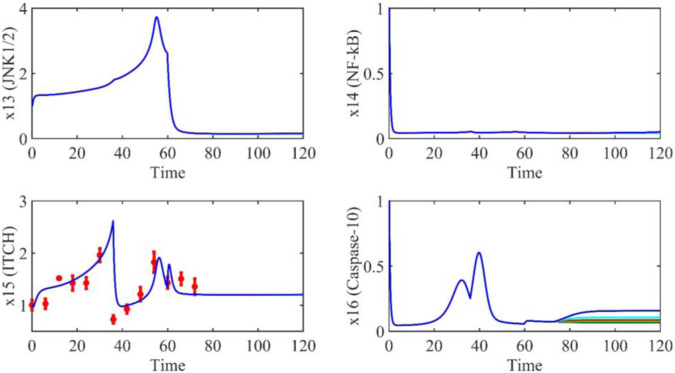
The dynamic trend of JNK1/2, NF-kB, ITCH and Caspase-10 from 0 h to 72 h when the parameter $a_{5}$ increases from 0.6 to 3.8, and the interval is 0.8.

**FIGURE 12:**
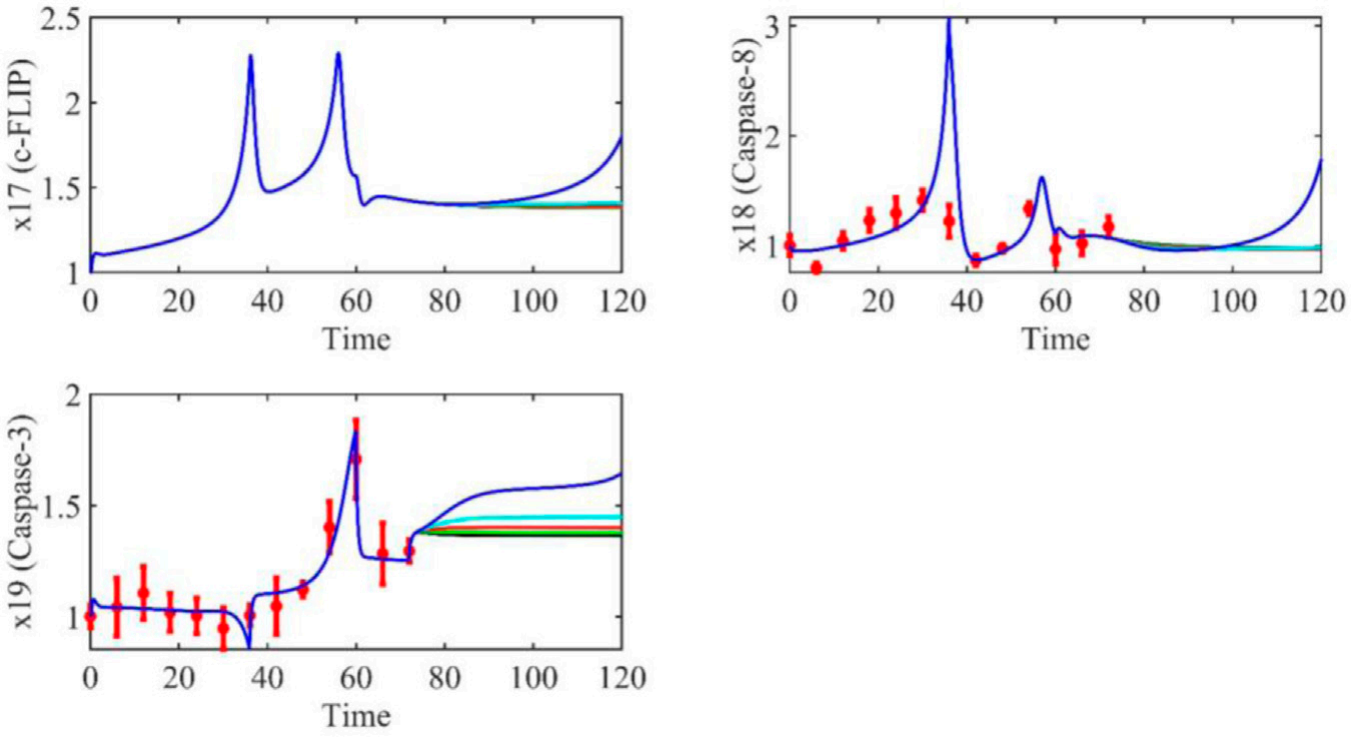
The dynamic trend of c-FLIP, Caspase-8 and Caspase-3 from 0 h to 72 h when the parameter $a_{5}$ increases from 0.6 to 3.8, and the interval is 0.8.

In this section, all parameters in the model were numerically simulated (see Supplementary Material). We randomly selected parameter $a_{5}$ to demonstrate the sensitivity of parameters on Caspase-3. The numerical simulation results show that when parameter $a_{5}$ increases from 0.6 to 3.8 with a step size of 0.8, the signaling molecules IL-1β, IRAK1, TNF-α, and TNFR1 show no significant changes (as shown in Figure 8). This is because parameter $a_{5}$ represents the production rate of the signaling molecule TRADD, which does not have a feedback regulatory effect on upstream signaling molecules. From Figure 9, it can be clearly seen that as parameter $a_{5}$ increases from 0.6 to 3.8, the mRNA expression level of TRADD gradually increases, and the growth amplitude is large. The black, green, red, light blue, and dark blue curves represent the dynamic trends of TRADD over time when $a_{5}$ is 0.8, 1.4, 2.0, 2.6, 3.2, and 3.8, respectively. Similarly, with the increase of $a_{5}$ , the mRNA expression levels of TRAF2/5 and NIK also gradually increase, and the increase is significant. Especially when $a_{5}$ is 3.8, the mRNA expression level of NIK is approximately 10 times that of $a_{5}=0.8$ . As for RIP1, although the expression level increases with the increase of $a_{5}$ , the increase is relatively small. From Figure 10, it can be seen that when $a_{5}$ increases from 0.8 to 3.2, the expression levels of TAK1, IKK-β, and MKK4 only increase slightly, but when $a_{5}$ increases to 3.8, the expression levels of TAK1, IKK-β, and MKK4 increase significantly. The expression level of FADD also increases gradually with the gradient increase of $a_{5}$ . Despite the large increase in $a_{5}$ from 0.8 to 3.8, JNK1/2, NF-kB, and ITCH show no significant changes. However, with the increase of $a_{5}$ , the mRNA expression level of Caspase-10 gradually increases, and when $a_{5}$ is 3.8, the mRNA expression level of Caspase-10 is significantly higher than the other cases (as shown in Figure 11). From Figure 12, it can be seen that when $a_{5}$ increases from 0.8 to 3.2, the mRNA expression levels of c-FLIP and Caspase-8 increase but not significantly, and the mRNA expression level of Caspase-3 also increases with the gradient increase of $a_{5}$ . However, when $a_{5}$ increases to 3.8, c-FLIP, Caspase-8, and Caspase-3 all undergo a stepwise increase. For example, when $a_{5}$ is 3.8, c-FLIP increases by 1.38 times, Caspase-8 increases by 1.8 times, and Caspase-3 increases by 1.27 times. Therefore, from the numerical simulation results, it can be seen that small changes in parameter $a_{5}$ can significantly affect the mRNA expression of downstream signaling molecules in the signaling pathway, especially the indicator signaling molecule Caspase-3, which is indicative of the severity of osteoarthritis. The mRNA expression level also undergoes significant changes. Therefore, parameter $a_{5}$ is a sensitive parameter that affects the progression of osteoarthritis and is a potential therapeutic target for treating osteoarthritis.

On the other hand, when parameter $b_{7}$ increases from 0.04 to 0.12 with a step size of 0.02, only the mRNA expression level of the signal molecule RIP1 in the entire signal pathway shows a significant change, while the other signal molecules show no significant changes. The numerical simulation results are shown in Supplementary Figures S2–S6. Therefore, parameter $b_{7}$ has no significant impact on osteoarthritis and is considered an insensitive parameter.

## 7 Conclusion

Since most of the current research on the regulation of signal pathways in chondrocyte apoptosis is based on molecular biology methods, it can only focus on studying the signaling molecules in a specific pathway, making it difficult to understand the mechanism of signal transduction at a systemic level. In this study, high-throughput sequencing is firstly used to screen for differentially expressed genes involved in IL-1β-induced apoptosis in human chondrocytes. The results show significant enrichment in the TNF signaling pathway. Therefore, mathematical modeling approach is adopted to quantitatively analyze the mechanism of TNF signaling pathway in regulating chondrocyte apoptosis. Next, qPCR experiments are performed to measure the mRNA expression levels of IRAK1 (*x*_2_), TNF-α (*x*_3_), TRADD (*x*_5_), RIP1(*x*_7_), NIK (*x*_8_), TAK1 (*x*_9_), IKK-β (*x*_10_), MKK4(*x*_11_), FADD (*x*_12_), ITCH (*x*_15_), Caspase-8 (*x*_18_) and Caspase-3 (*x*_19_) at 0 h, 6 h, 12 h, 18 h, 24 h, 30 h, 36 h, 42 h, 48 h, 54 h, 60 h, 66 h, and 72 h. The 62 parameters of the model are estimated by using the collected experimental data. Since the process of chondrocyte apoptosis is time-dependent, the parameter estimation was divided into three stages. The effectiveness of parameter estimation is evaluated by calculating the correlation coefficient between the experimental data and the mathematical model, with a maximum correlation coefficient of 0.969 (See Table 7). Finally, numerical simulation are used to calculate the sensitivity of the model parameters, and the results show that parameters $b_{4}$ , $a_{5}$ , $d_{5}$ , $a_{6}$ , $a_{10}$ , $b_{10}$ , $g_{10}$ , $f_{10}$ , $d_{10}$ , $b_{12}$ , $d_{12}$ , $a_{16}$ , $b_{16}$ , $d_{16}$ , $a_{17}$ , $a_{18}$ , $b_{18}$ , $a_{19}$ , $b_{19}$ , $g_{19}$ and $d_{19}$ are sensitive parameters. These sensitive parameters significantly affect chondrocyte apoptosis and further influence the severity of osteoarthritis. Therefore, in future studies, we will design siRNA interference experiments to validate the sensitive and insensitive parameters at the cellular level, further explore the sensitive factors that affect chondrocyte apoptosis, and potentially identify new therapeutic targets for the treatment of osteoarthritis at a systemic level.

## Data Availability

The platform for the high-throughput sequencing is Health Time Gene and the sequencing data were uploaded to NCBI, the number is PRJNA1019114.
