# Supplementary material for: Mathematical modeling and application of IL-1β/TNF signaling pathway in regulating chondrocyte apoptosis
Source: Front Cell Dev Biol. 2023 Nov 2;11:1288431. doi: 10.3389/fcell.2023.1288431 (PMC10652750; doi:10.3389/fcell.2023.1288431)
Supplement: Supplementary file 1 [file Presentation1.pdf]

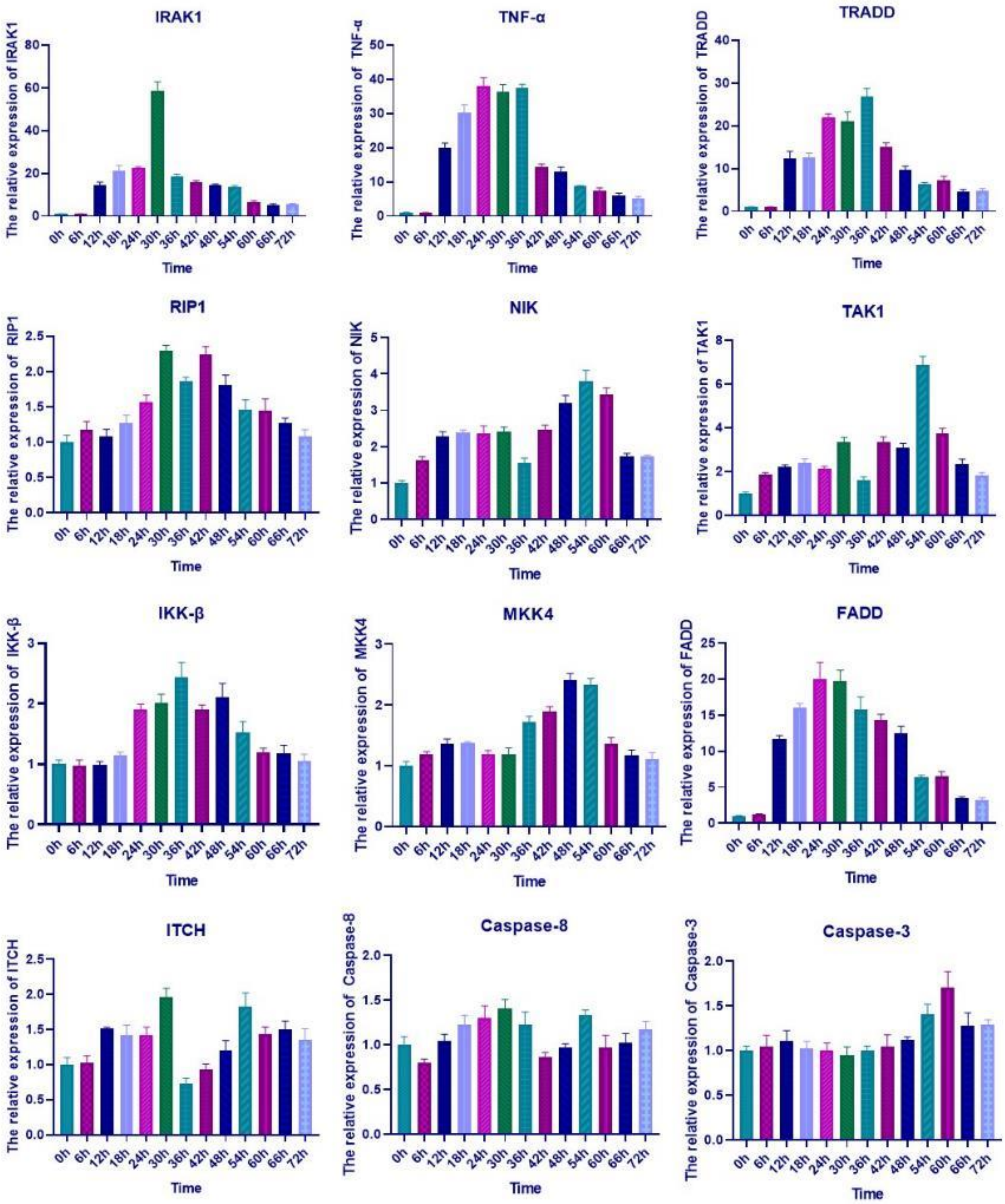

Figure S1 Gene relative expression of IRAK1(x_2), TNF-α (x_3), TRADD(x_5), RIP1(x_7), NIK (x_8), TAK1(x_9), IKK-β (x_{10}), MKK4(x_{11}), FADD (x_{12}), ITCH (x_{15}), Caspase-8 (x_{18}) and Caspase-3 (x_{19}) with the interval was 6h from 0h to 72h.

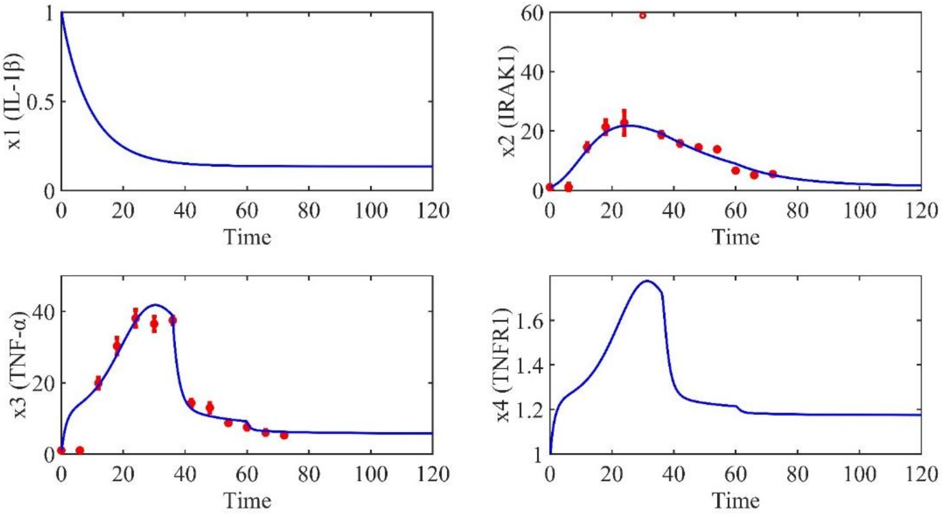

Figure S2 The dynamic trend of IL-1β, IRAK1, TNF-α and TNFR1 from 0 h to 72h when the parameter b_7 increases from 0.04 to 0.12, and the interval is 0.02.

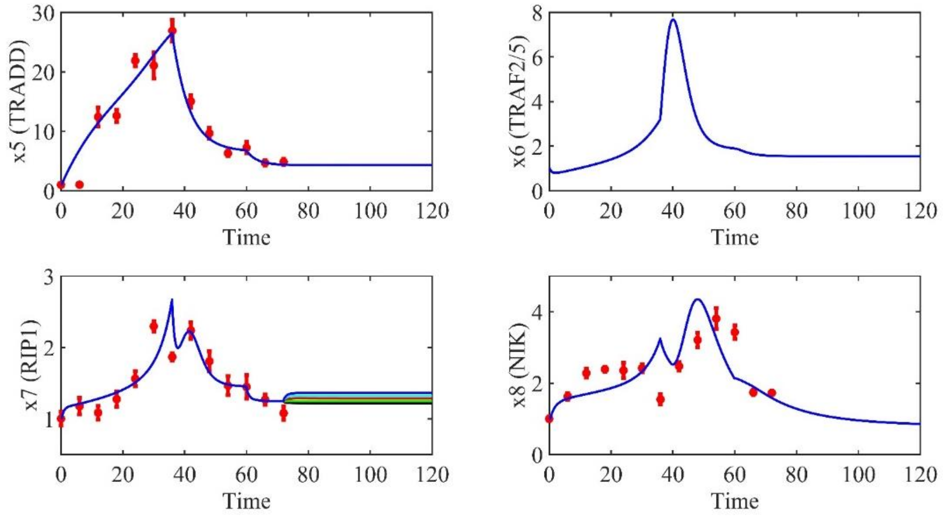

Figure S3 The dynamic trend of TRADD, TRAF2/5, RIP1 and NIK from 0 h to 72h when the parameter b_7 increases from 0.04 to 0.12, and the interval is 0.02.

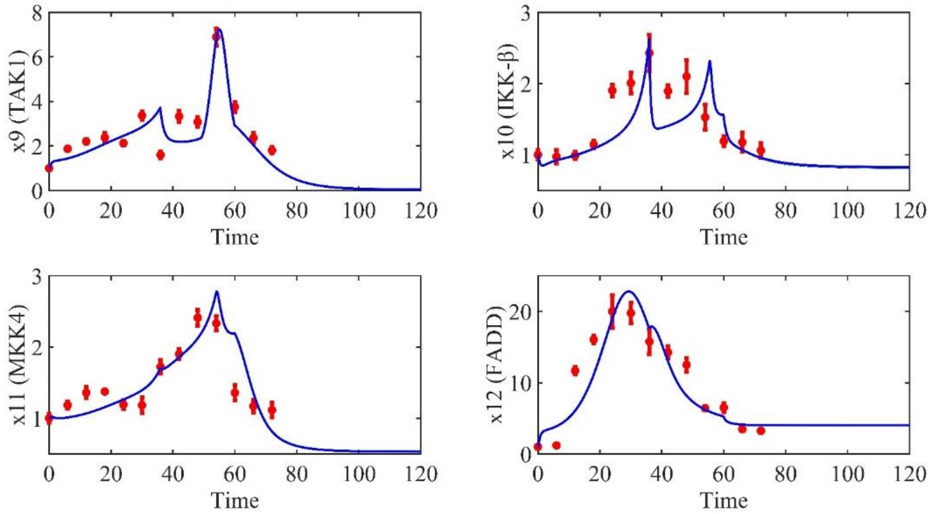

Figure S4 The dynamic trend of TAK1, IKK-β, MKK4 and FADD from 0 h to 72h when the parameter b_7 increases from 0.04 to 0.12, and the interval is 0.02.

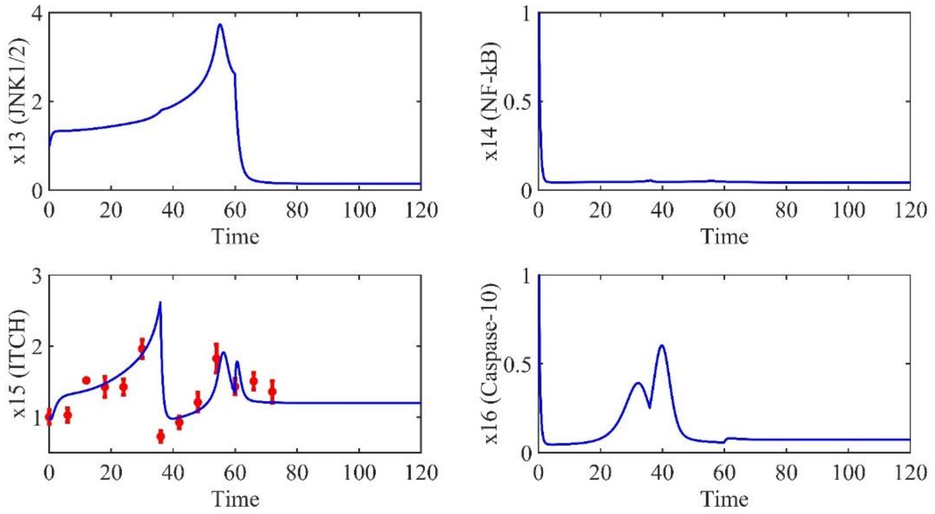

Figure S5 The dynamic trend of JNK1/2, NF-kB, ITCH and Caspase-10 from 0 h to 72h when the parameter b_7 increases from 0.04 to 0.12, and the interval is 0.02.

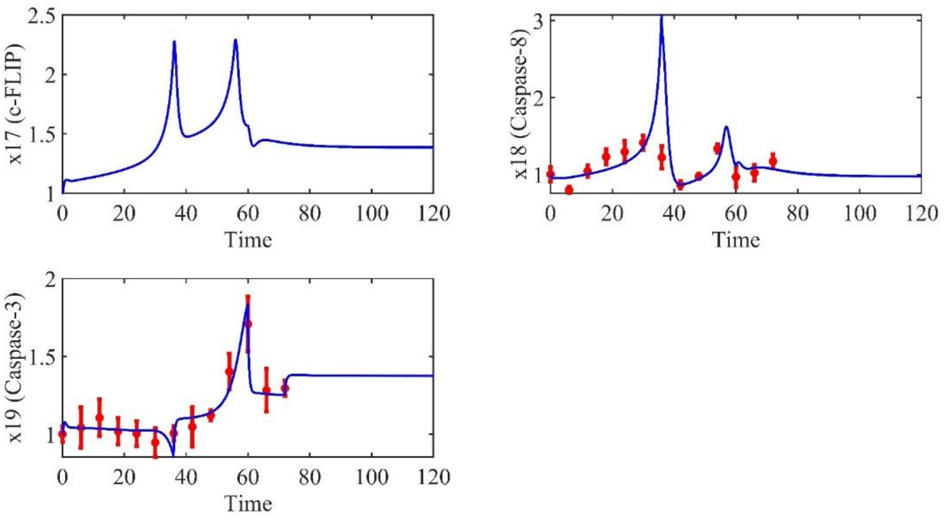

Figure S6 The dynamic trend of c-FLIP, Caspase-8 and Caspase-3 from 0 h to 72h when the parameter b_7 increases from 0.04 to 0.12, and the interval is 0.02.
