# Supplementary figures and images for: Mathematical modeling and application of IL-1β/TNF signaling pathway in regulating chondrocyte apoptosis (part 2 of 4)
Source: Front Cell Dev Biol. 2023 Nov 2;11:1288431. doi: 10.3389/fcell.2023.1288431 (PMC10652750; doi:10.3389/fcell.2023.1288431)

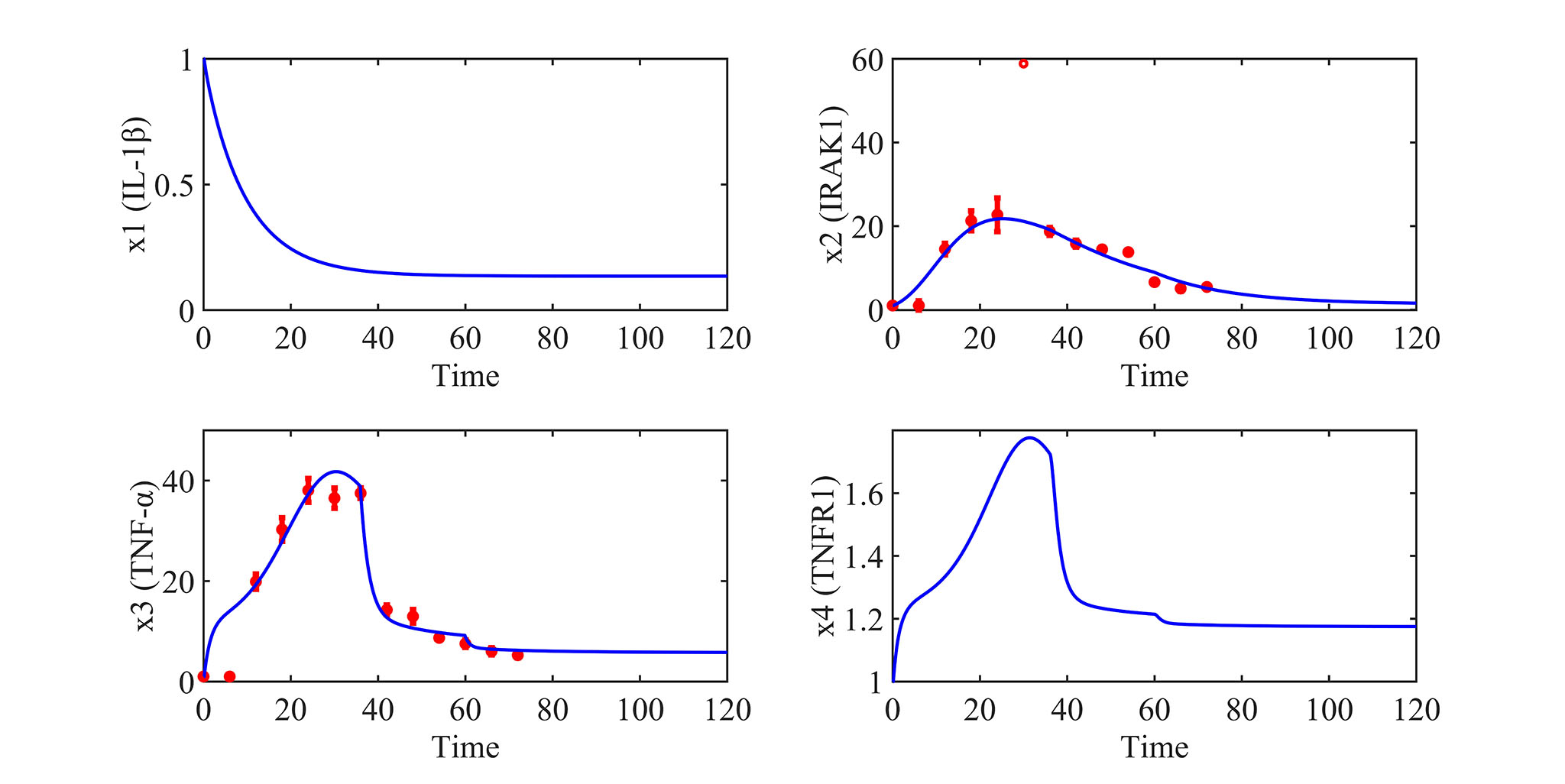

Supplement: Supplementary file 2 [file DataSheet1.zip › Supplementary material_image1/Parameter_b11(小)/1.jpg]

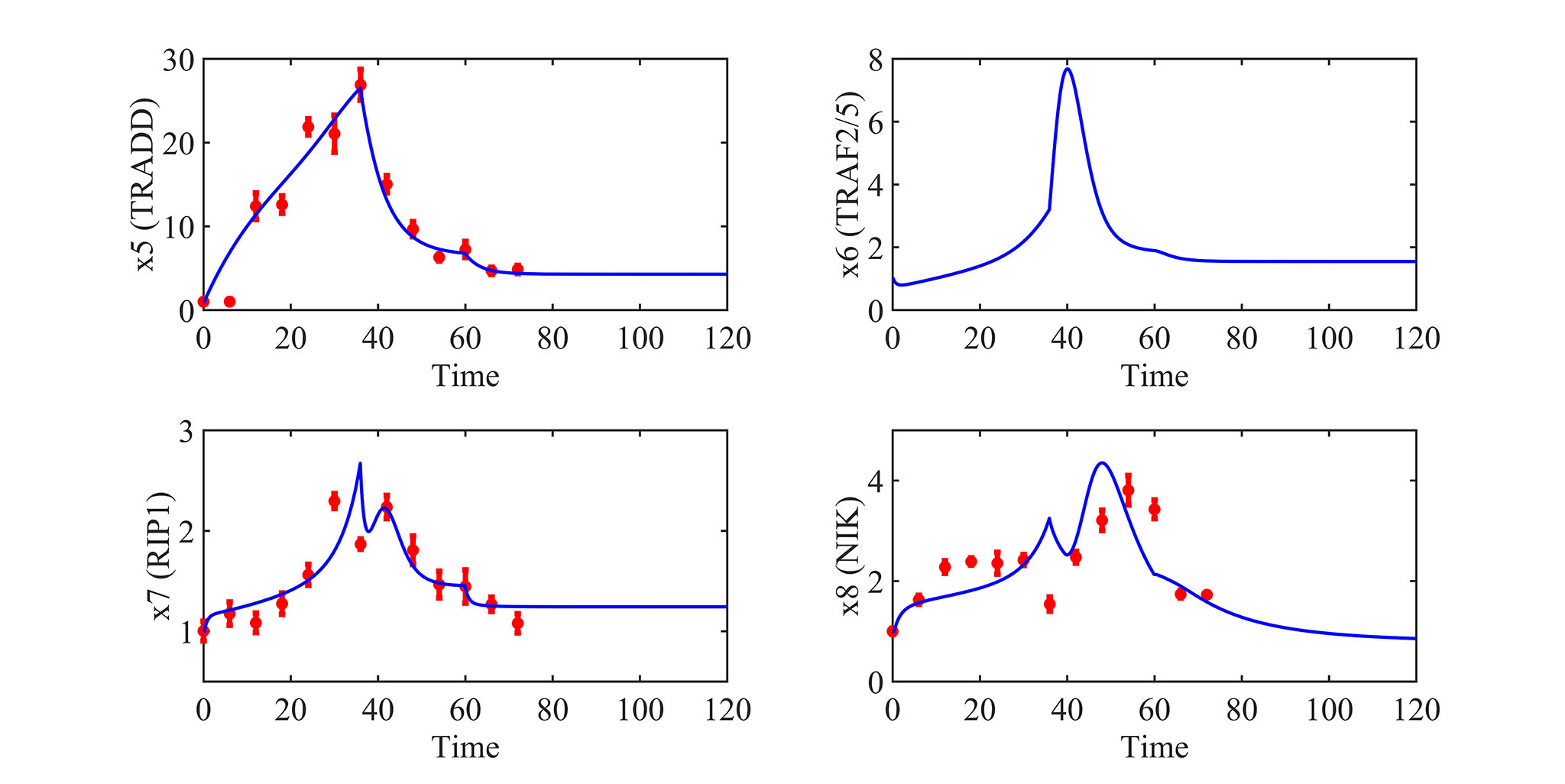

Supplement: Supplementary file 2 [file DataSheet1.zip › Supplementary material_image1/Parameter_b11(小)/2.jpg]

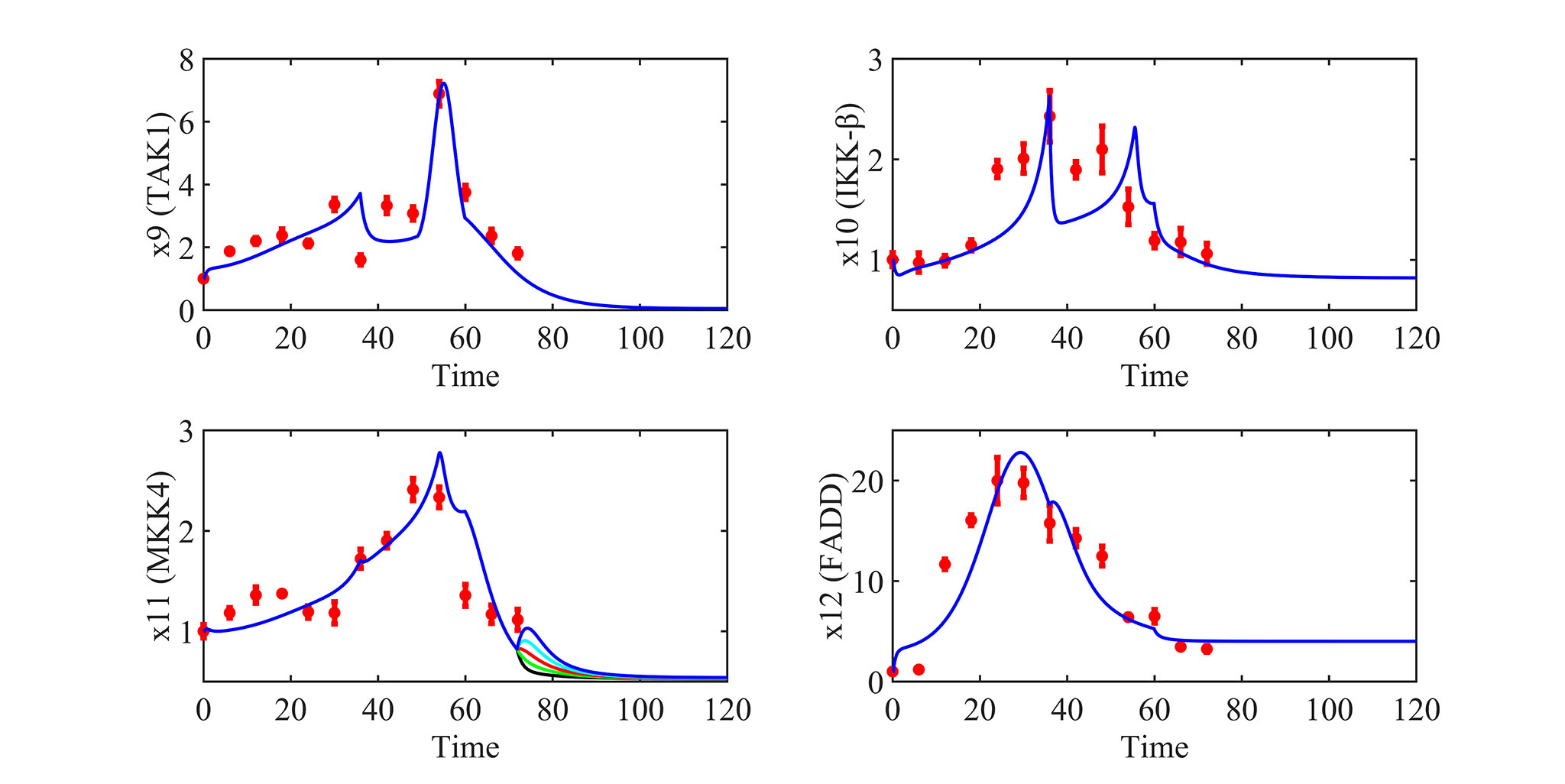

Supplement: Supplementary file 2 [file DataSheet1.zip › Supplementary material_image1/Parameter_b11(小)/3.jpg]

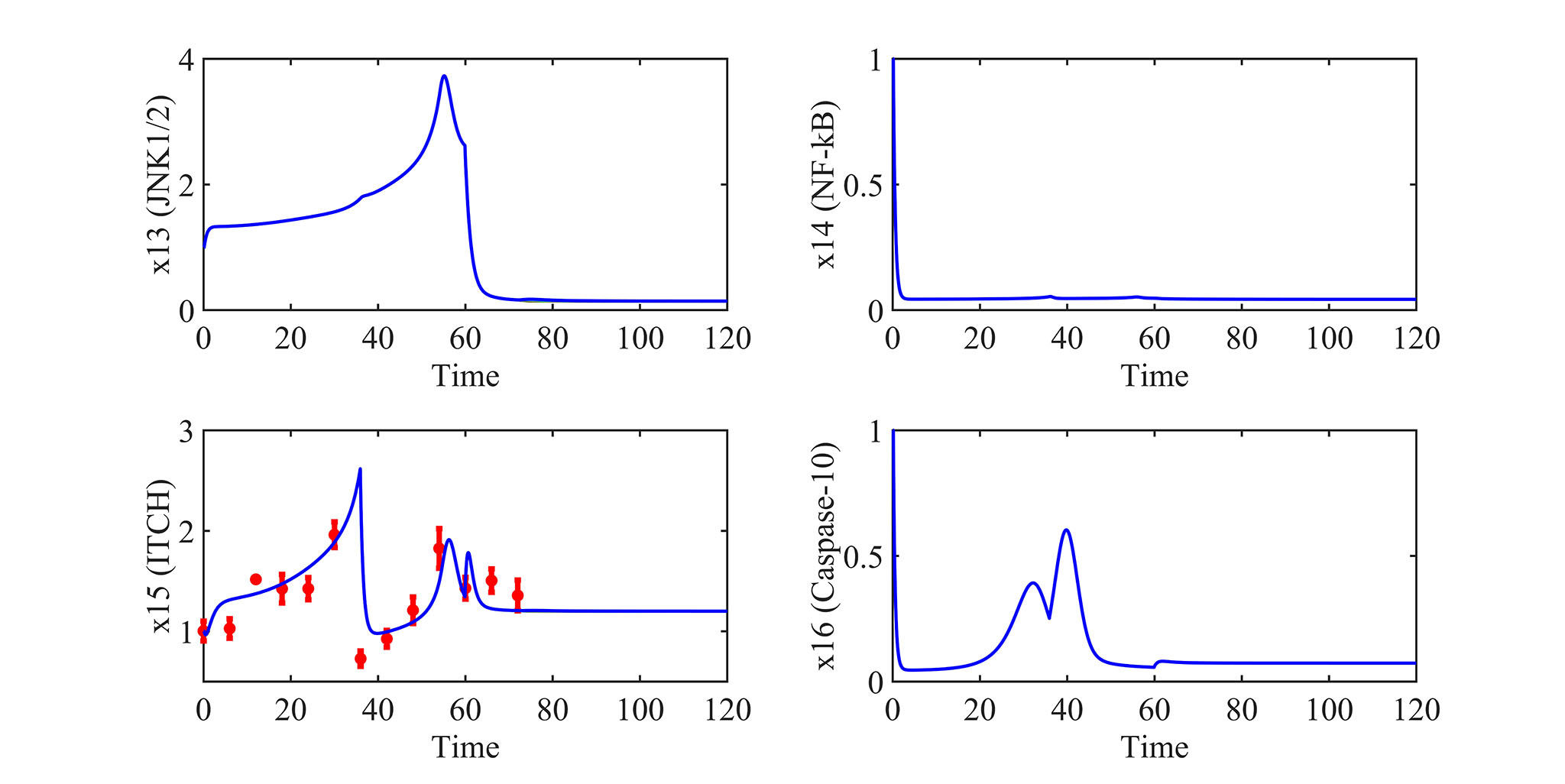

Supplement: Supplementary file 2 [file DataSheet1.zip › Supplementary material_image1/Parameter_b11(小)/4.jpg]

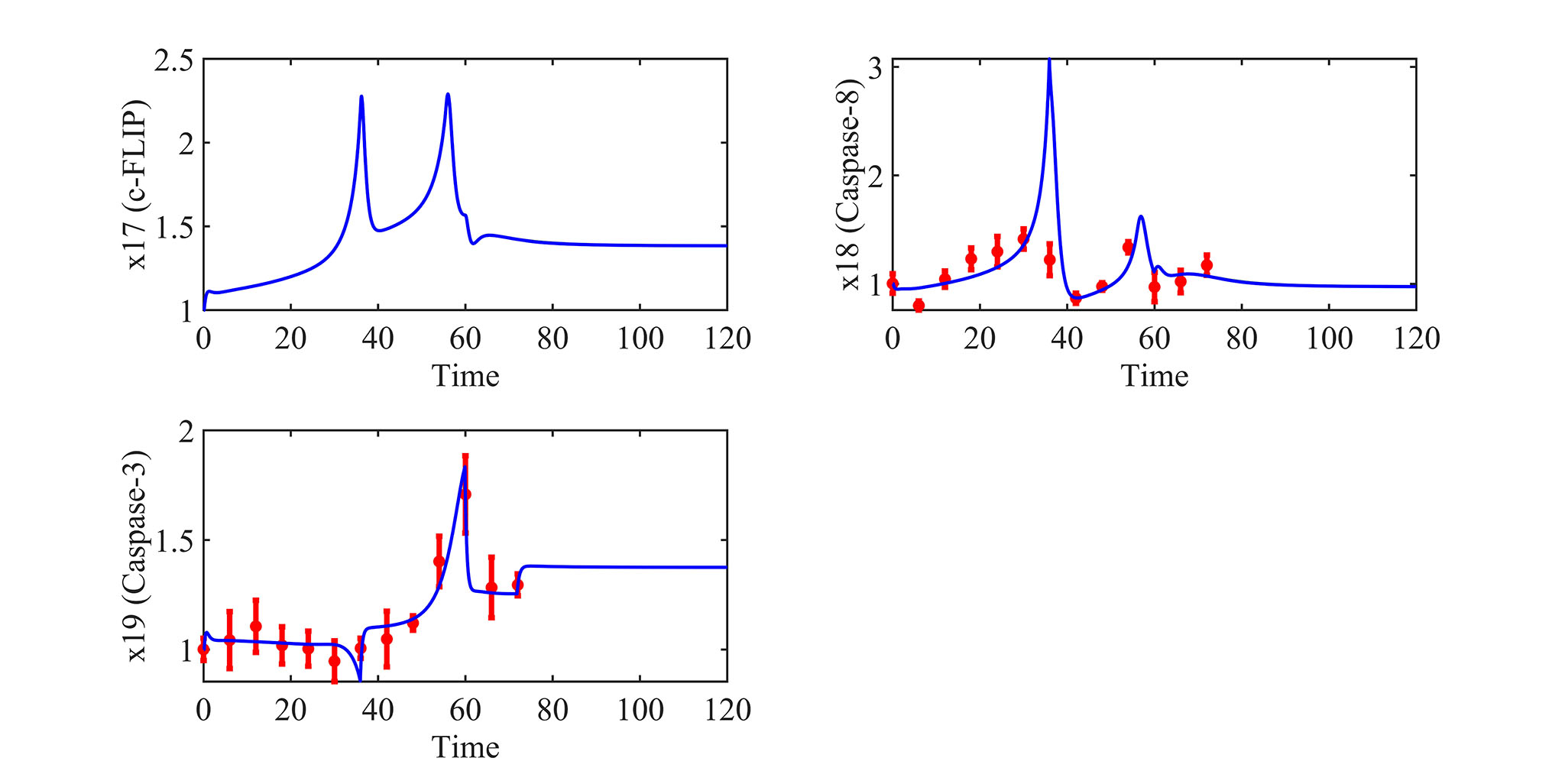

Supplement: Supplementary file 2 [file DataSheet1.zip › Supplementary material_image1/Parameter_b11(小)/5.jpg]

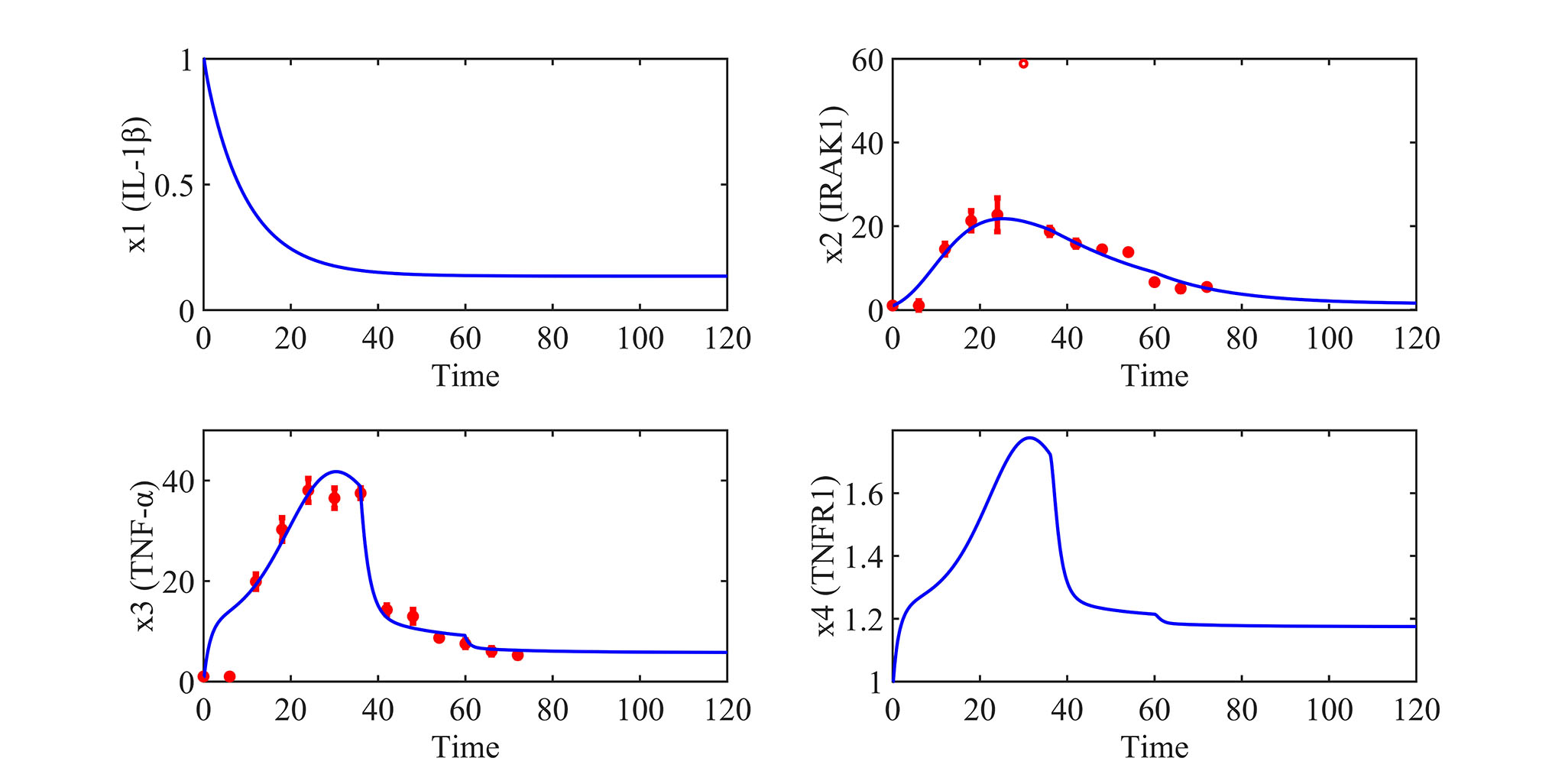

Supplement: Supplementary file 2 [file DataSheet1.zip › Supplementary material_image1/Parameter_b12(大)/1.jpg]

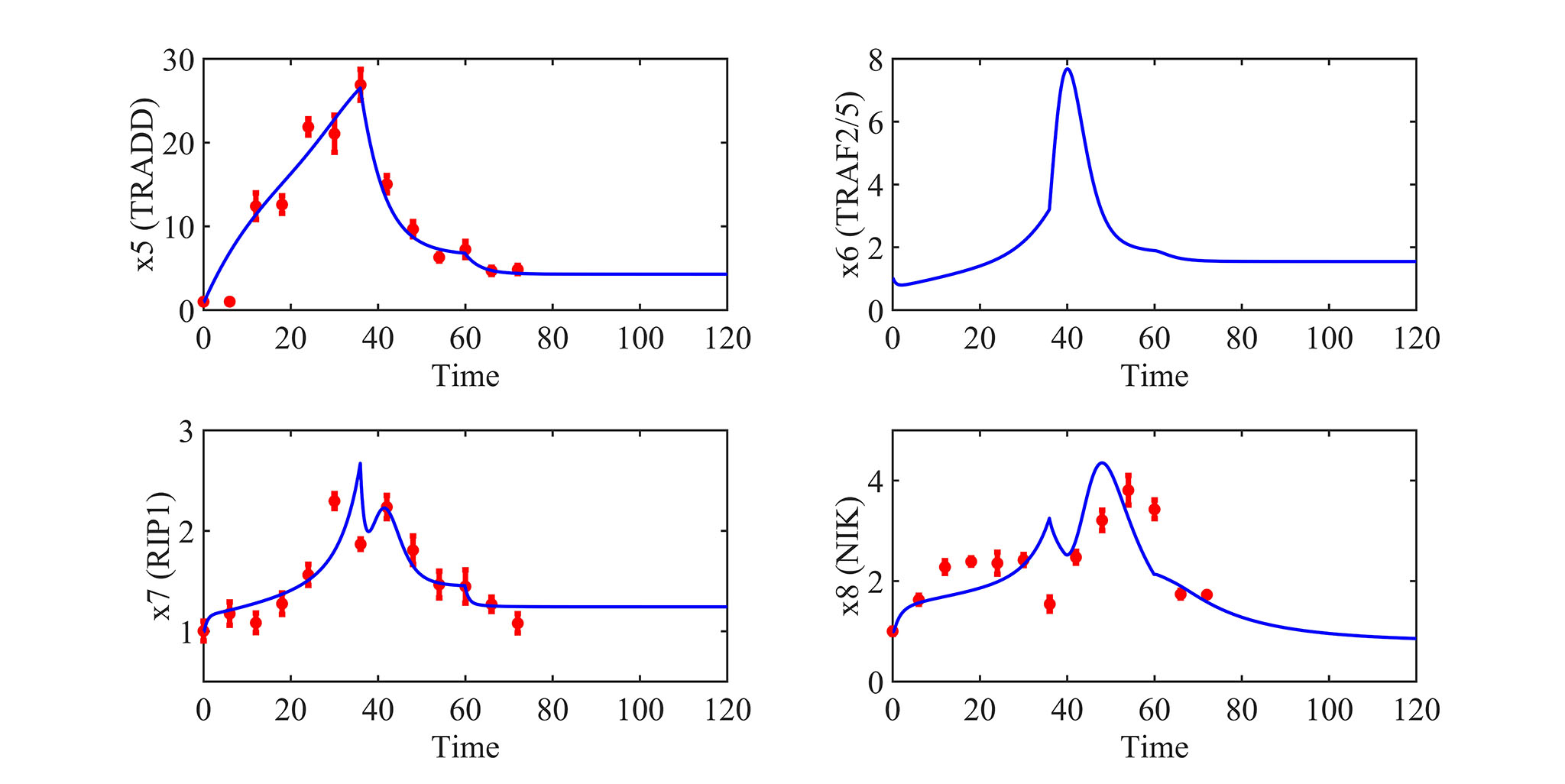

Supplement: Supplementary file 2 [file DataSheet1.zip › Supplementary material_image1/Parameter_b12(大)/2.jpg]

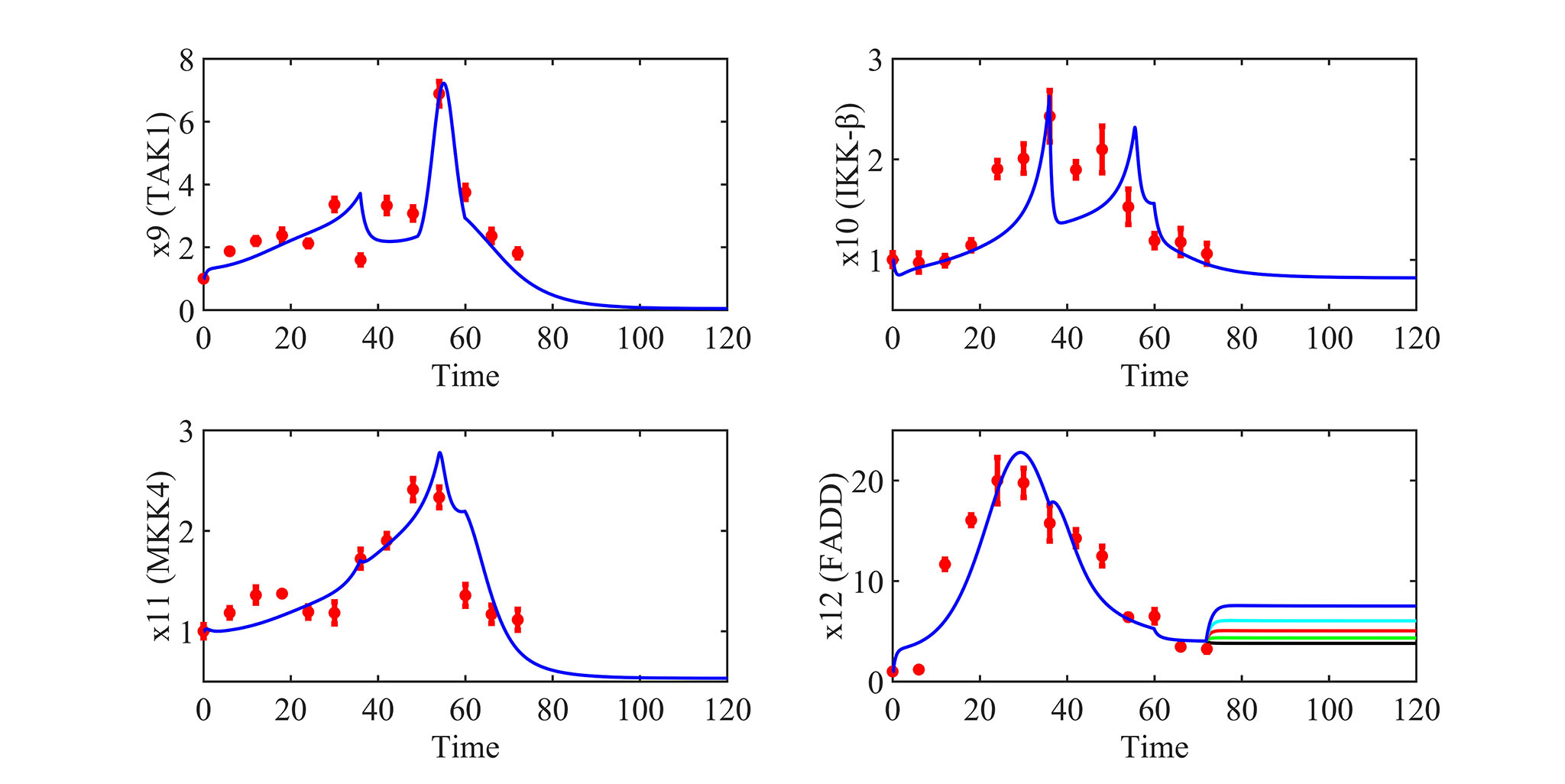

Supplement: Supplementary file 2 [file DataSheet1.zip › Supplementary material_image1/Parameter_b12(大)/3.jpg]

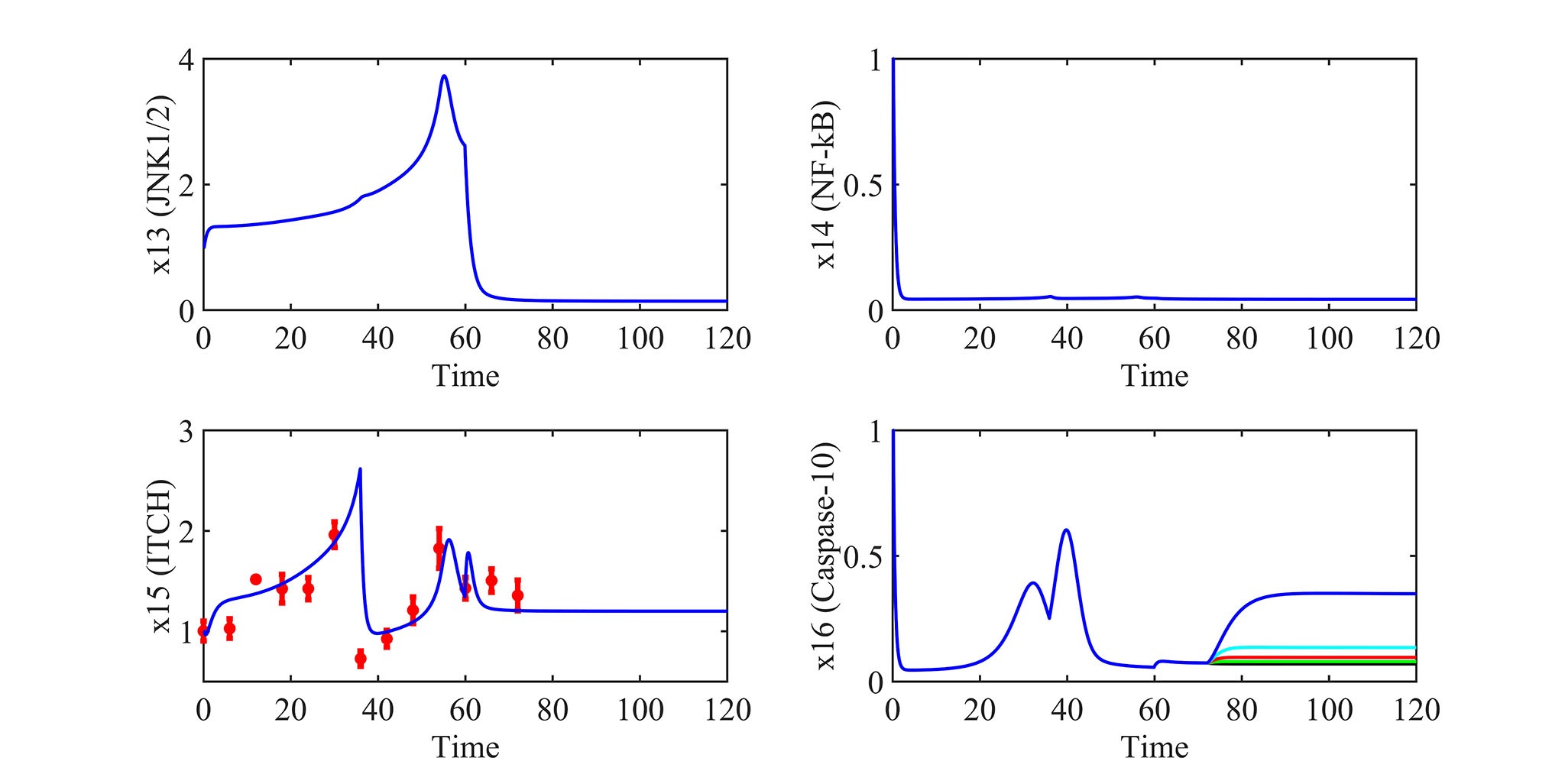

Supplement: Supplementary file 2 [file DataSheet1.zip › Supplementary material_image1/Parameter_b12(大)/4.jpg]

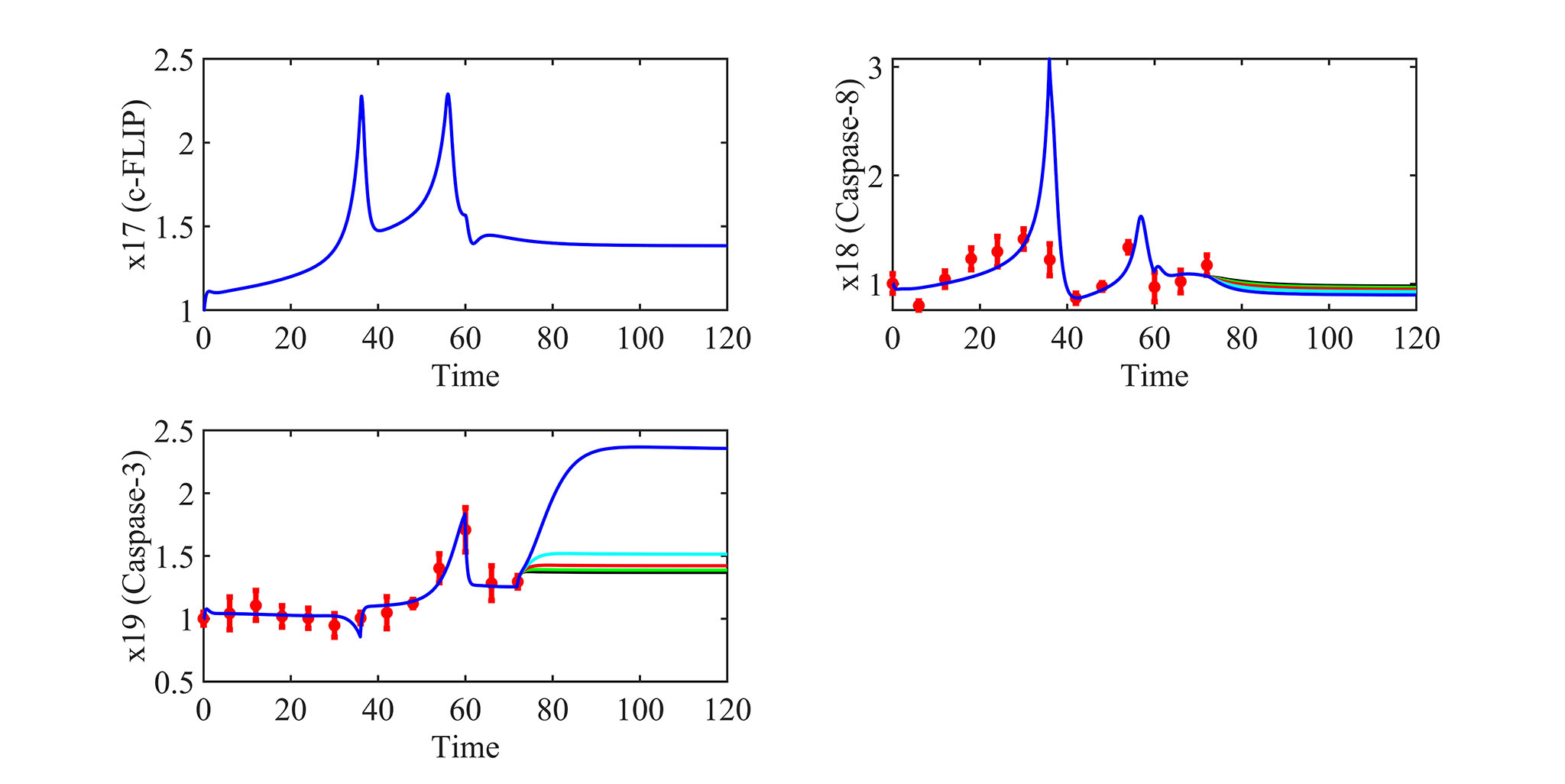

Supplement: Supplementary file 2 [file DataSheet1.zip › Supplementary material_image1/Parameter_b12(大)/5.jpg]

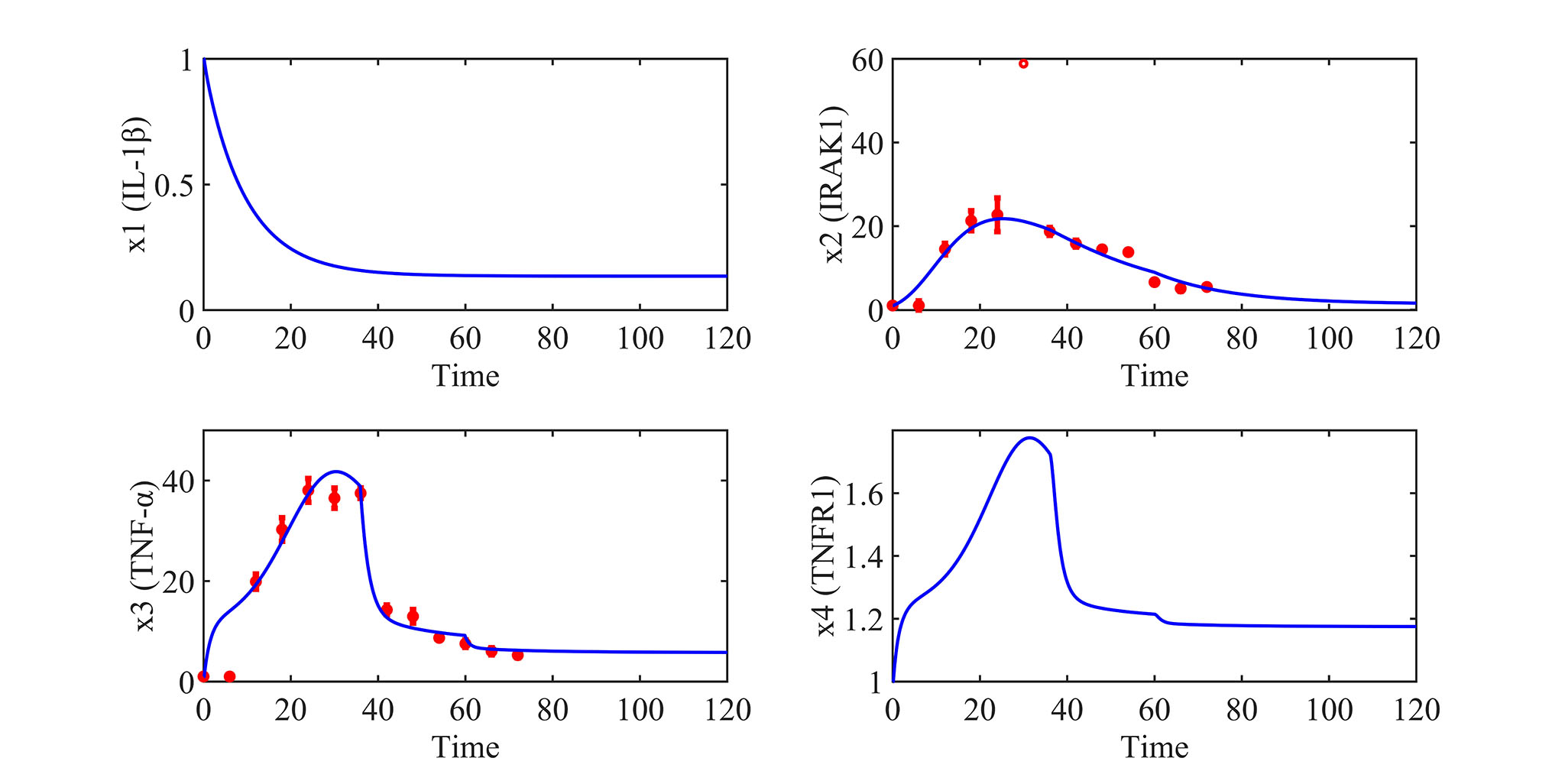

Supplement: Supplementary file 2 [file DataSheet1.zip › Supplementary material_image1/Parameter_b13(小)/1.jpg]

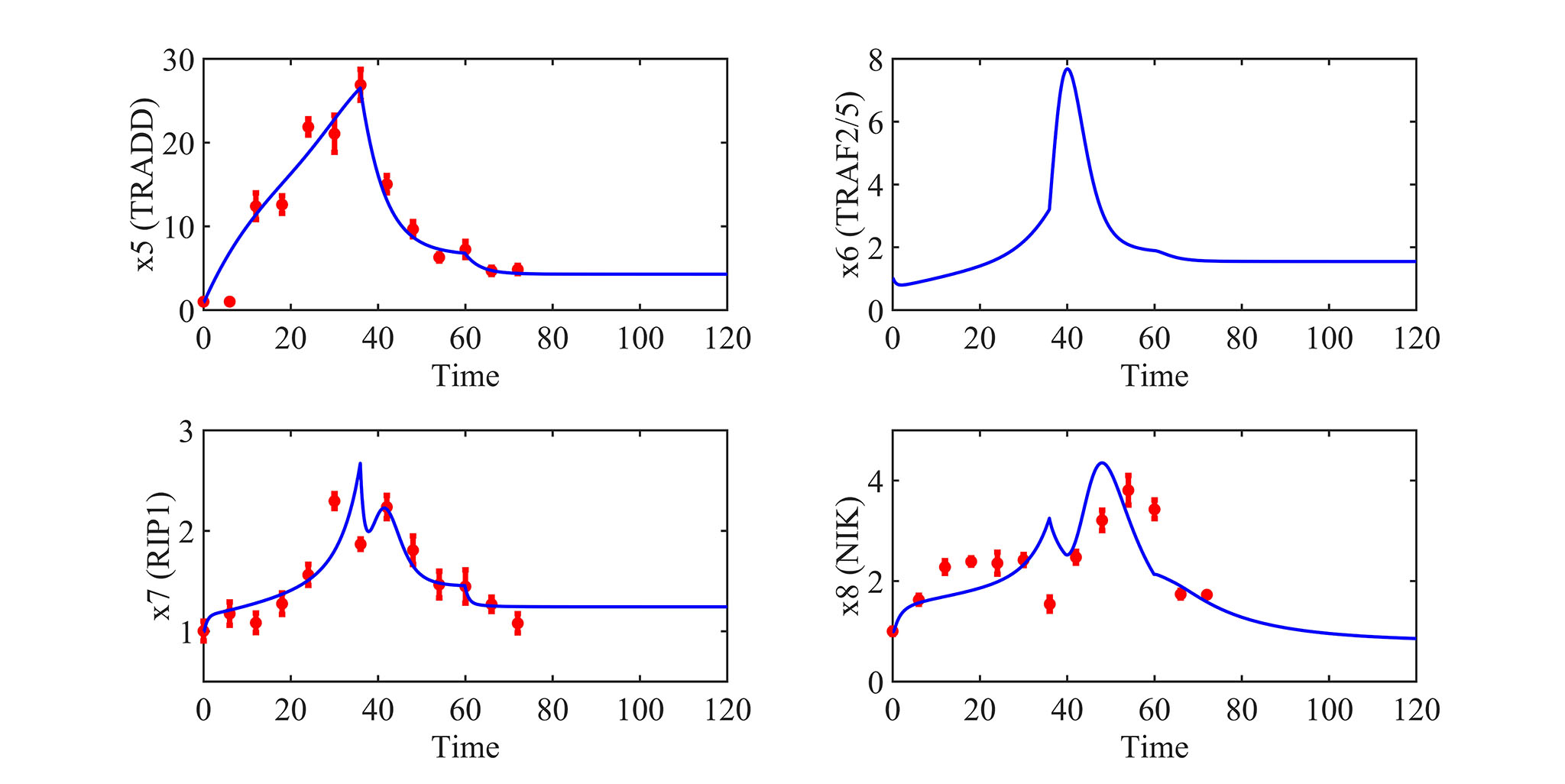

Supplement: Supplementary file 2 [file DataSheet1.zip › Supplementary material_image1/Parameter_b13(小)/2.jpg]

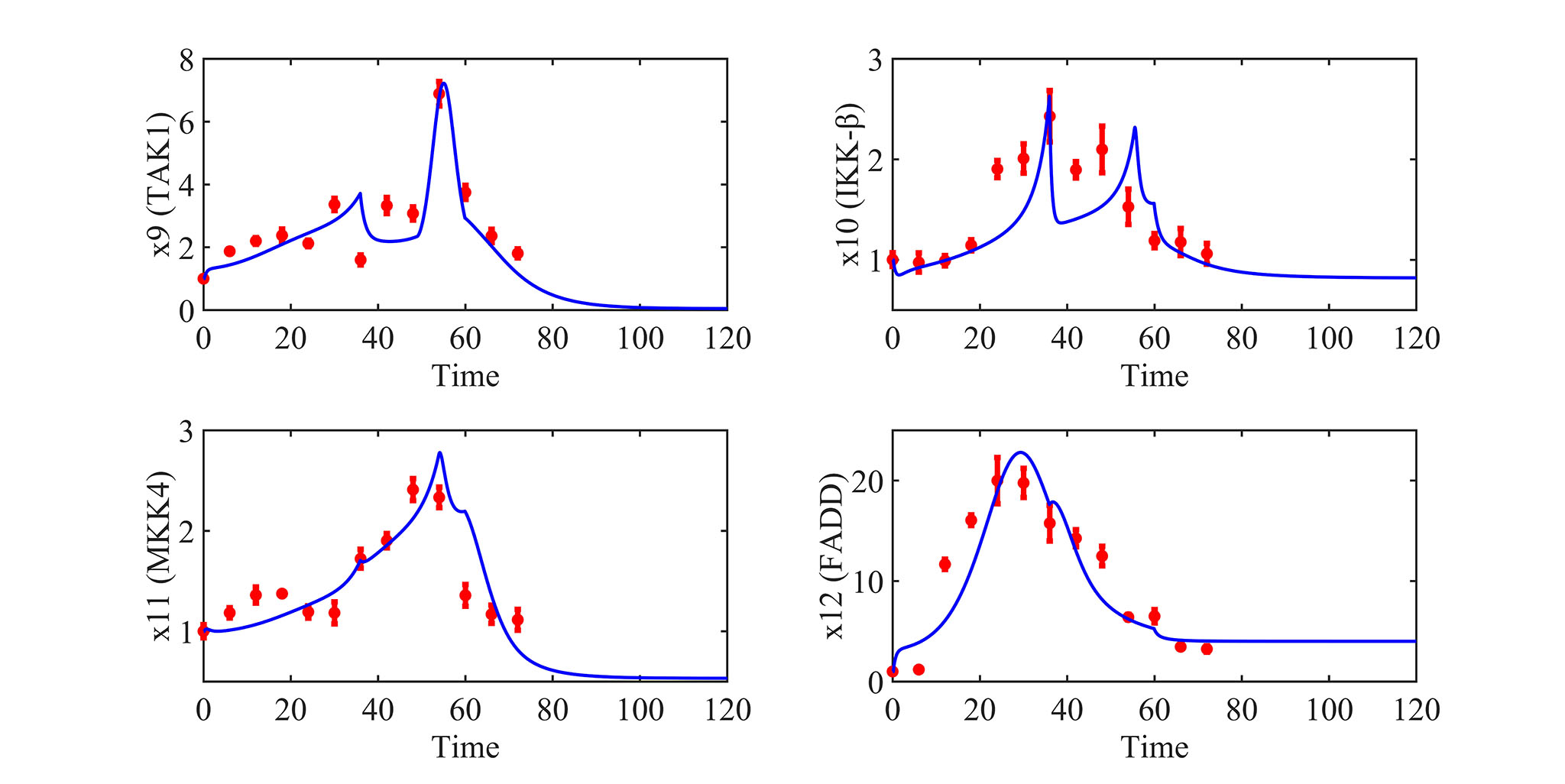

Supplement: Supplementary file 2 [file DataSheet1.zip › Supplementary material_image1/Parameter_b13(小)/3.jpg]

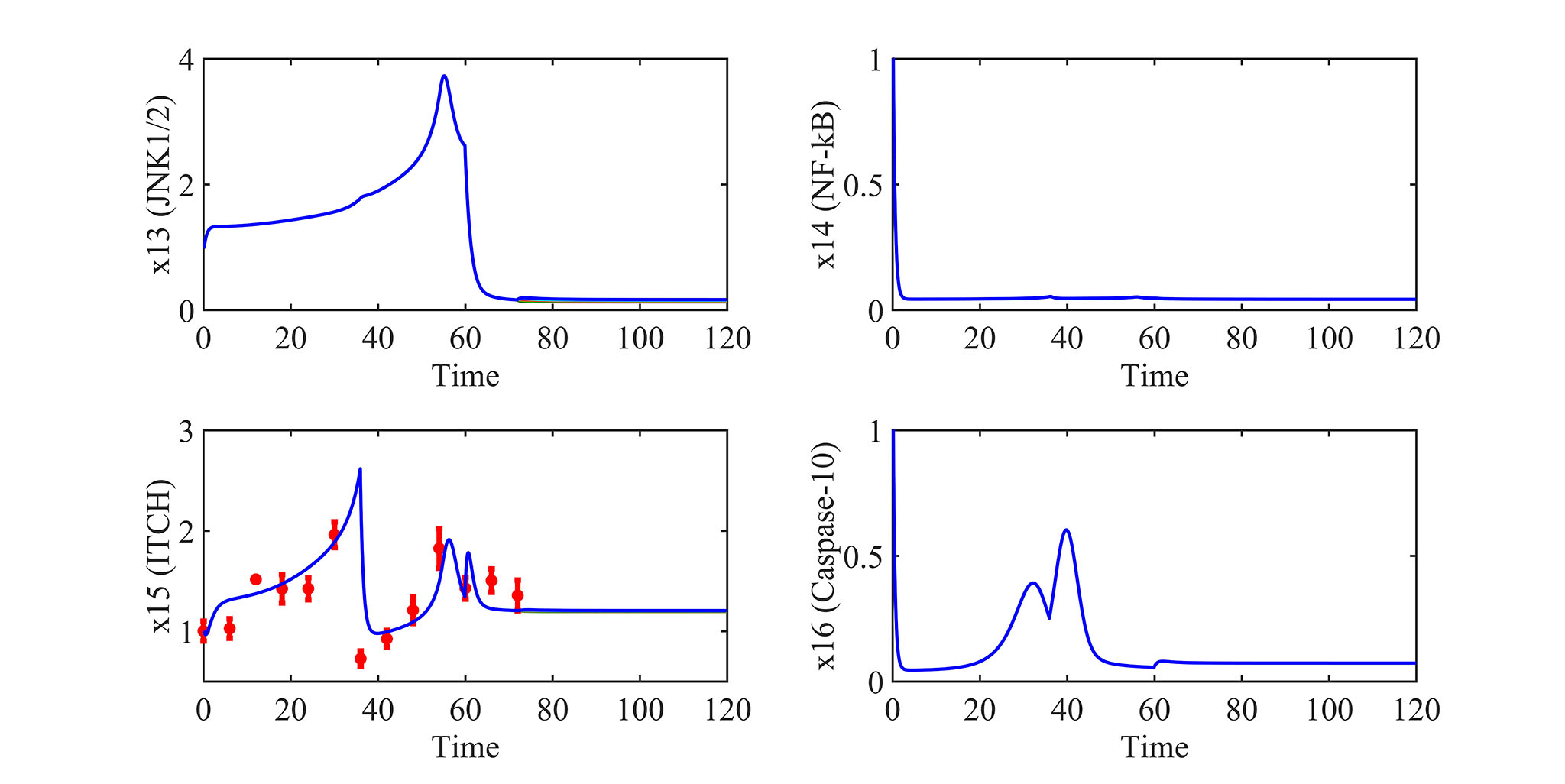

Supplement: Supplementary file 2 [file DataSheet1.zip › Supplementary material_image1/Parameter_b13(小)/4.jpg]

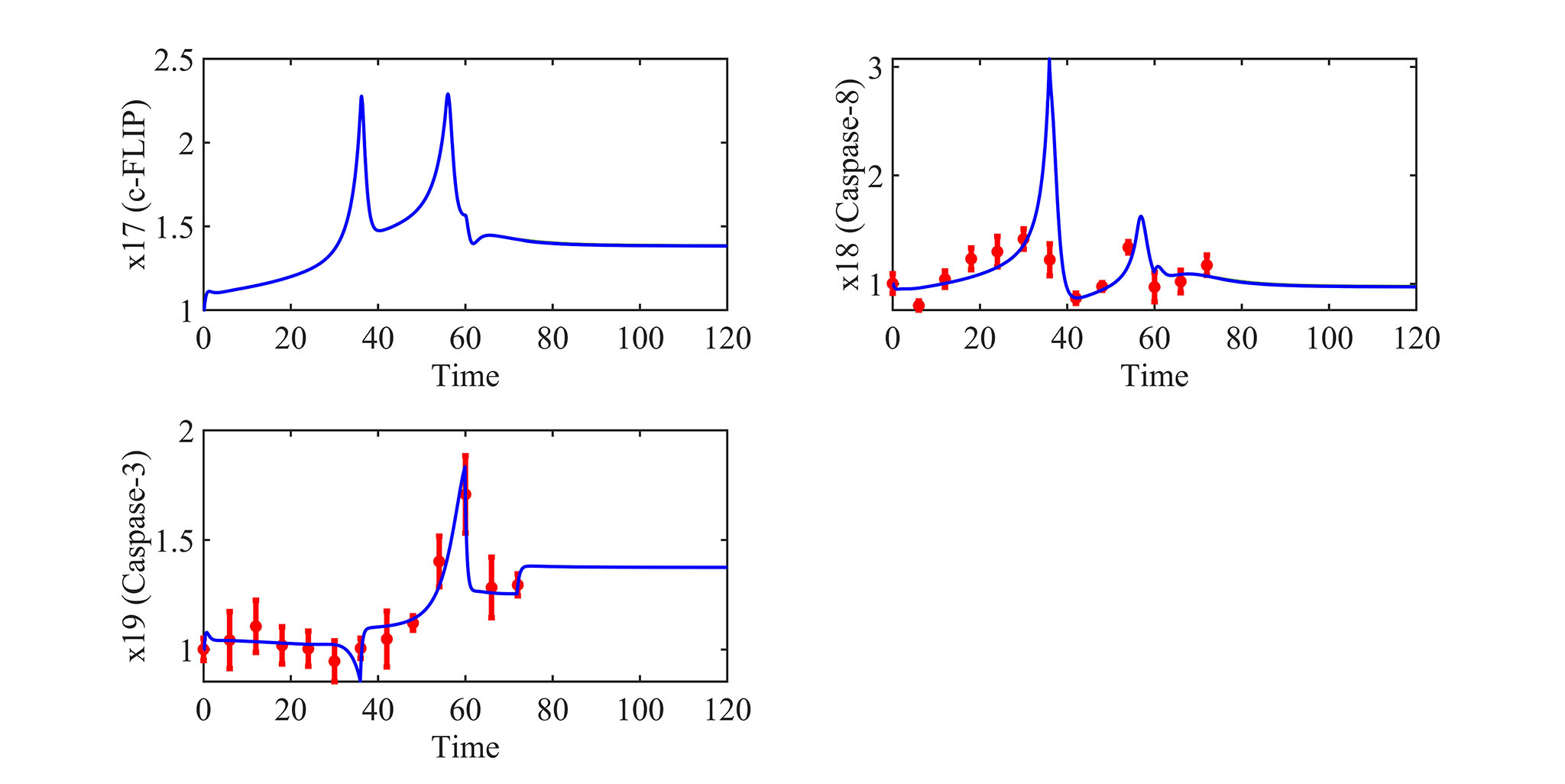

Supplement: Supplementary file 2 [file DataSheet1.zip › Supplementary material_image1/Parameter_b13(小)/5.jpg]

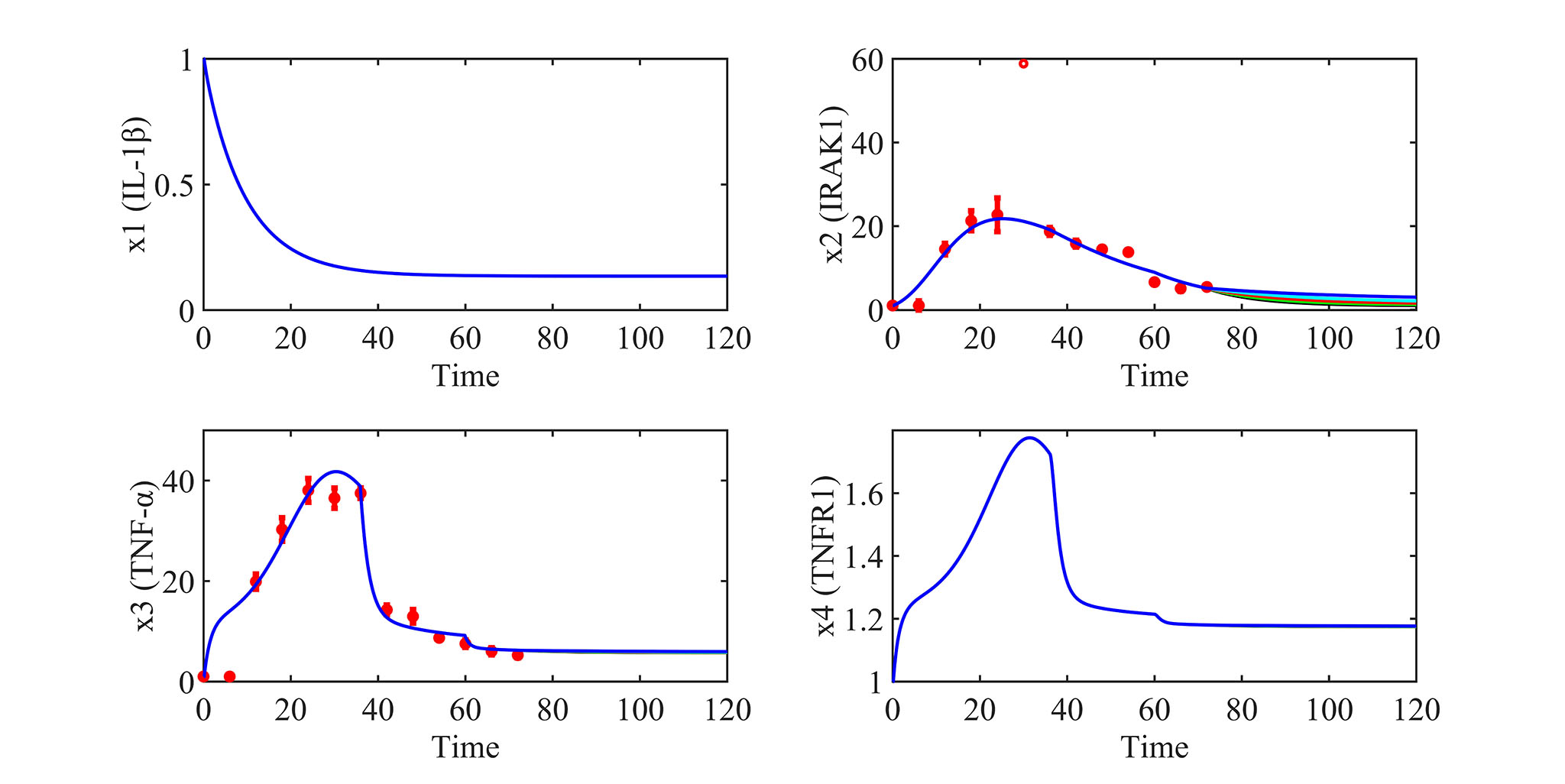

Supplement: Supplementary file 2 [file DataSheet1.zip › Supplementary material_image1/Parameter_b2(小)/1.jpg]

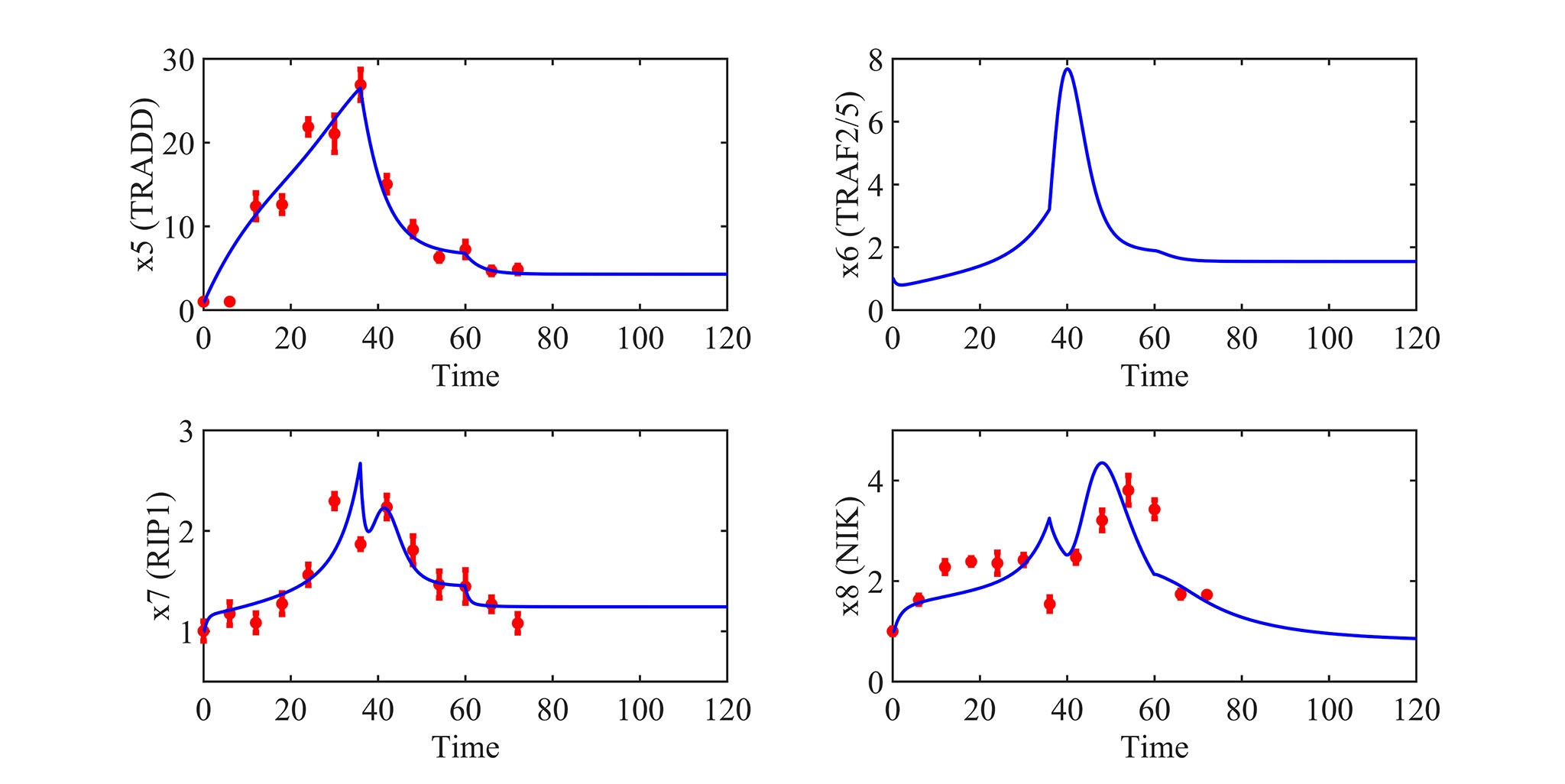

Supplement: Supplementary file 2 [file DataSheet1.zip › Supplementary material_image1/Parameter_b2(小)/2.jpg]

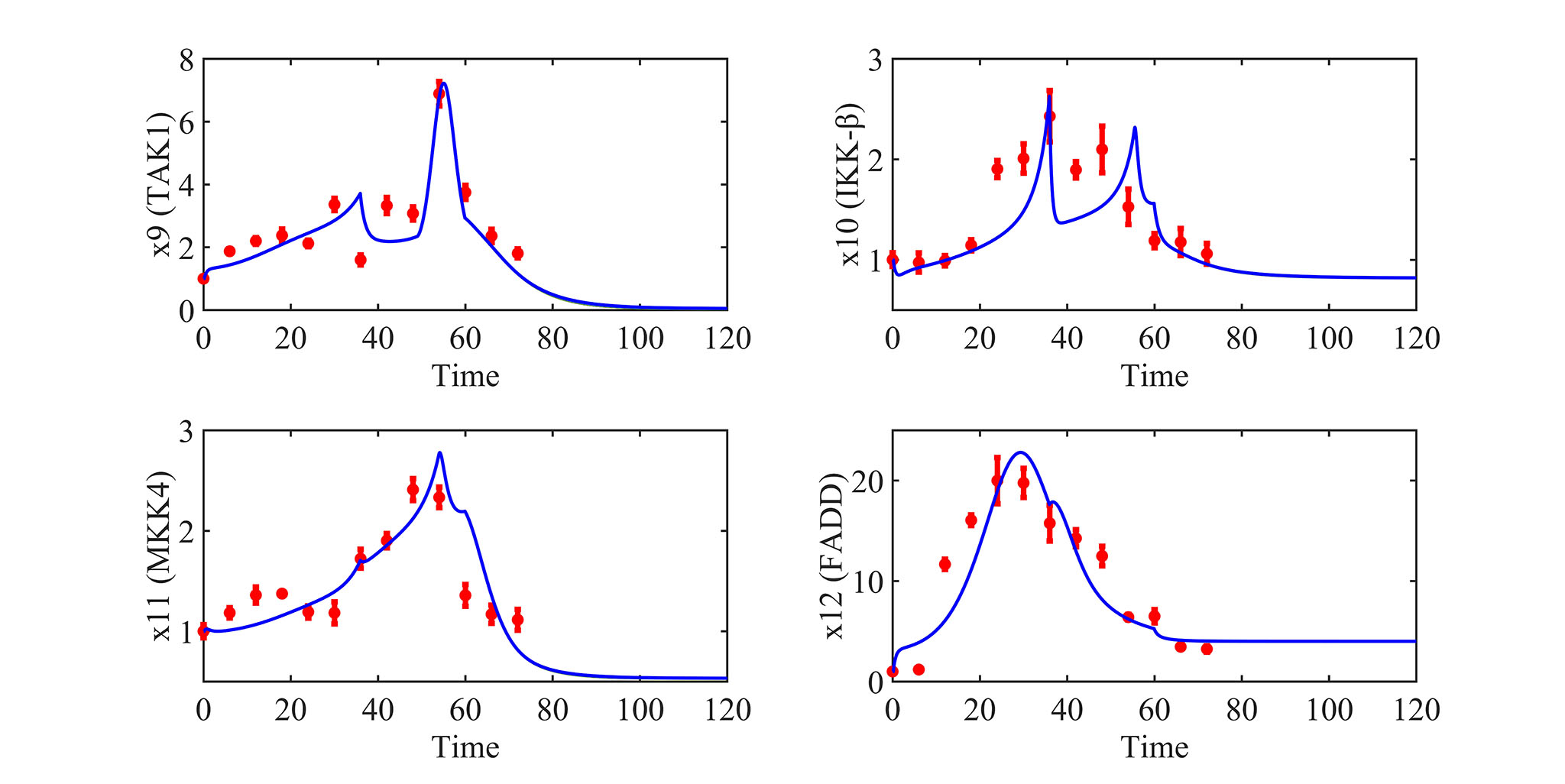

Supplement: Supplementary file 2 [file DataSheet1.zip › Supplementary material_image1/Parameter_b2(小)/3.jpg]

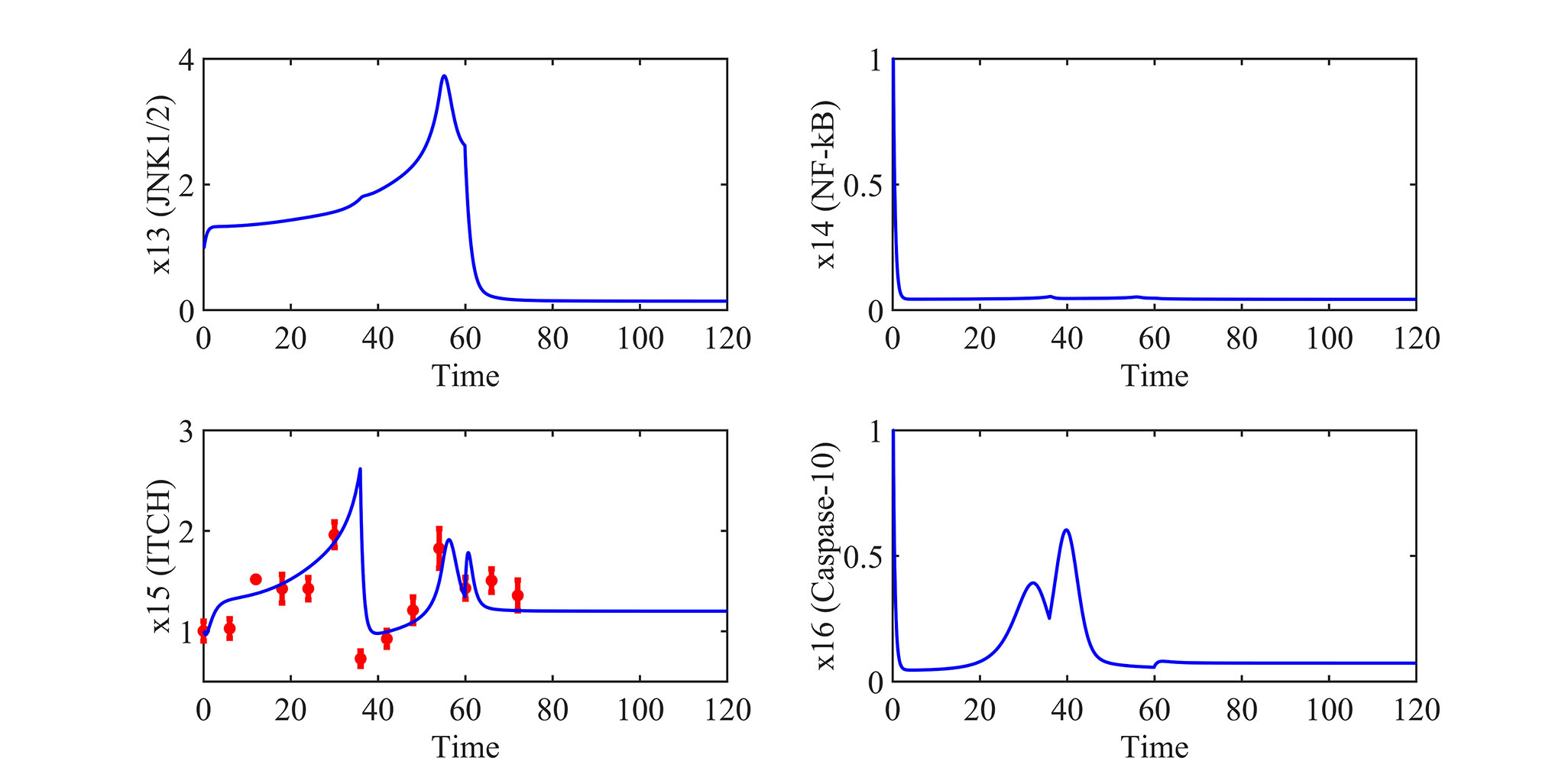

Supplement: Supplementary file 2 [file DataSheet1.zip › Supplementary material_image1/Parameter_b2(小)/4.jpg]

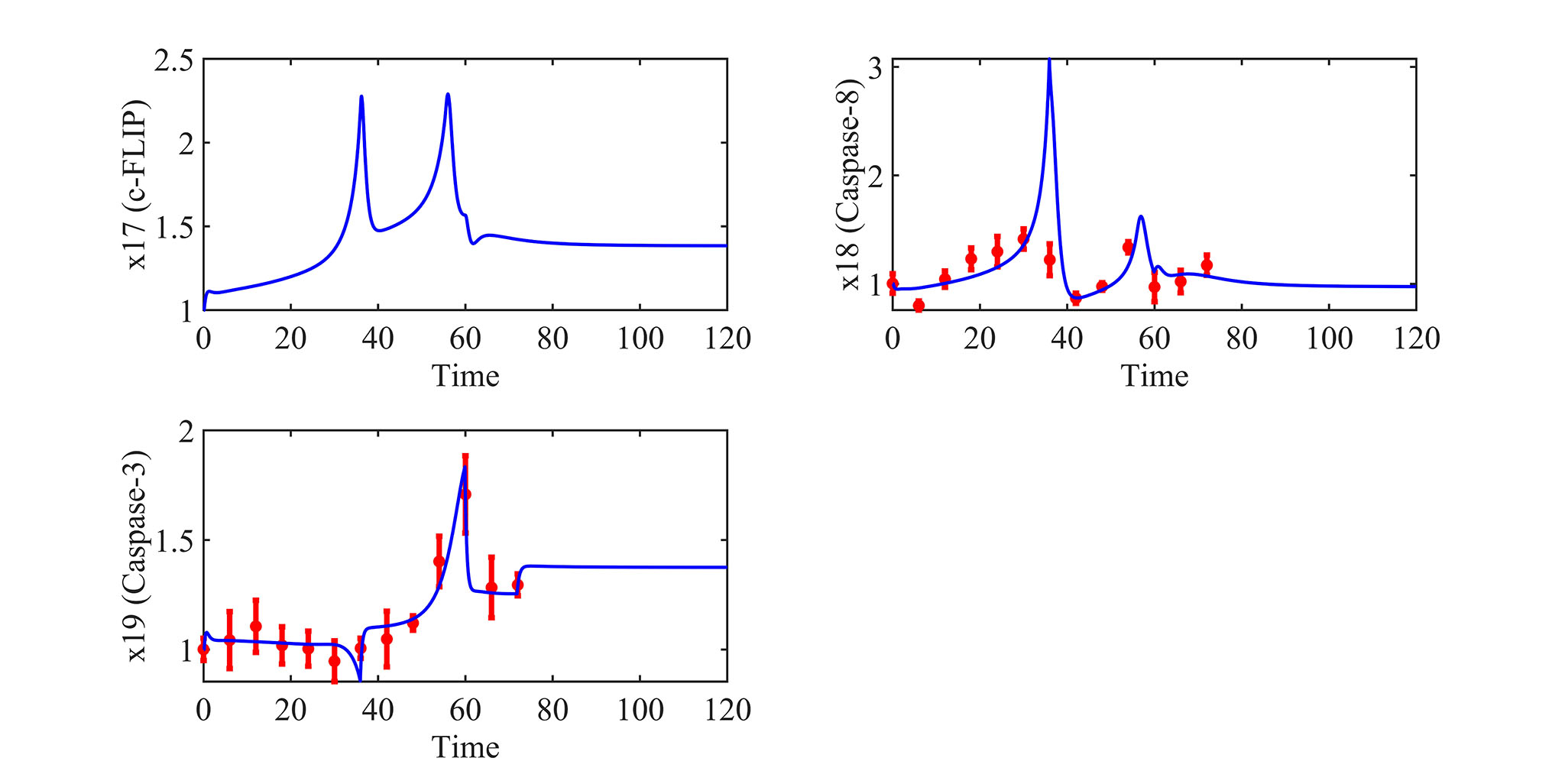

Supplement: Supplementary file 2 [file DataSheet1.zip › Supplementary material_image1/Parameter_b2(小)/5.jpg]

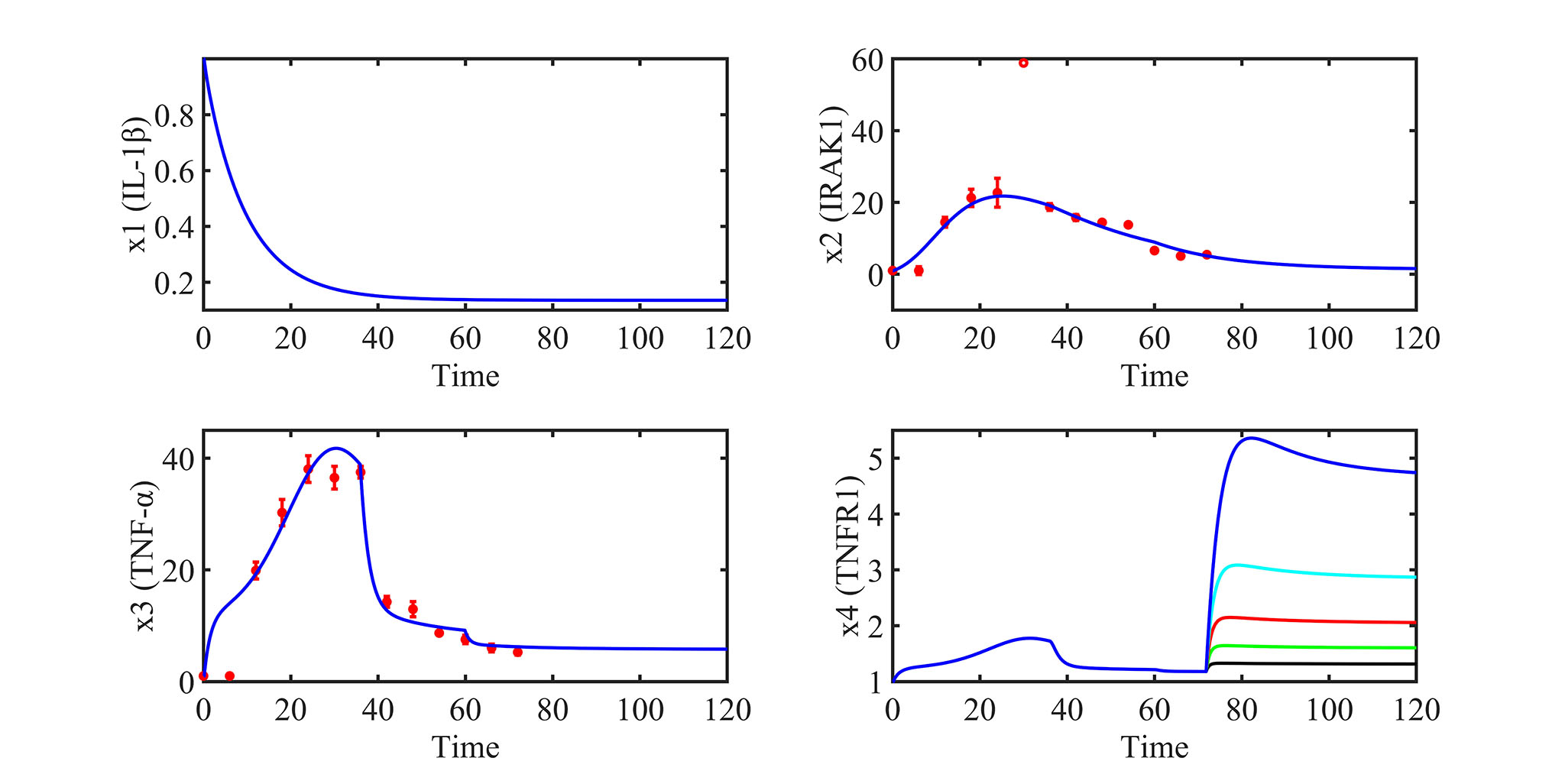

Supplement: Supplementary file 2 [file DataSheet1.zip › Supplementary material_image1/Parameter_b4(小)/11.jpg]

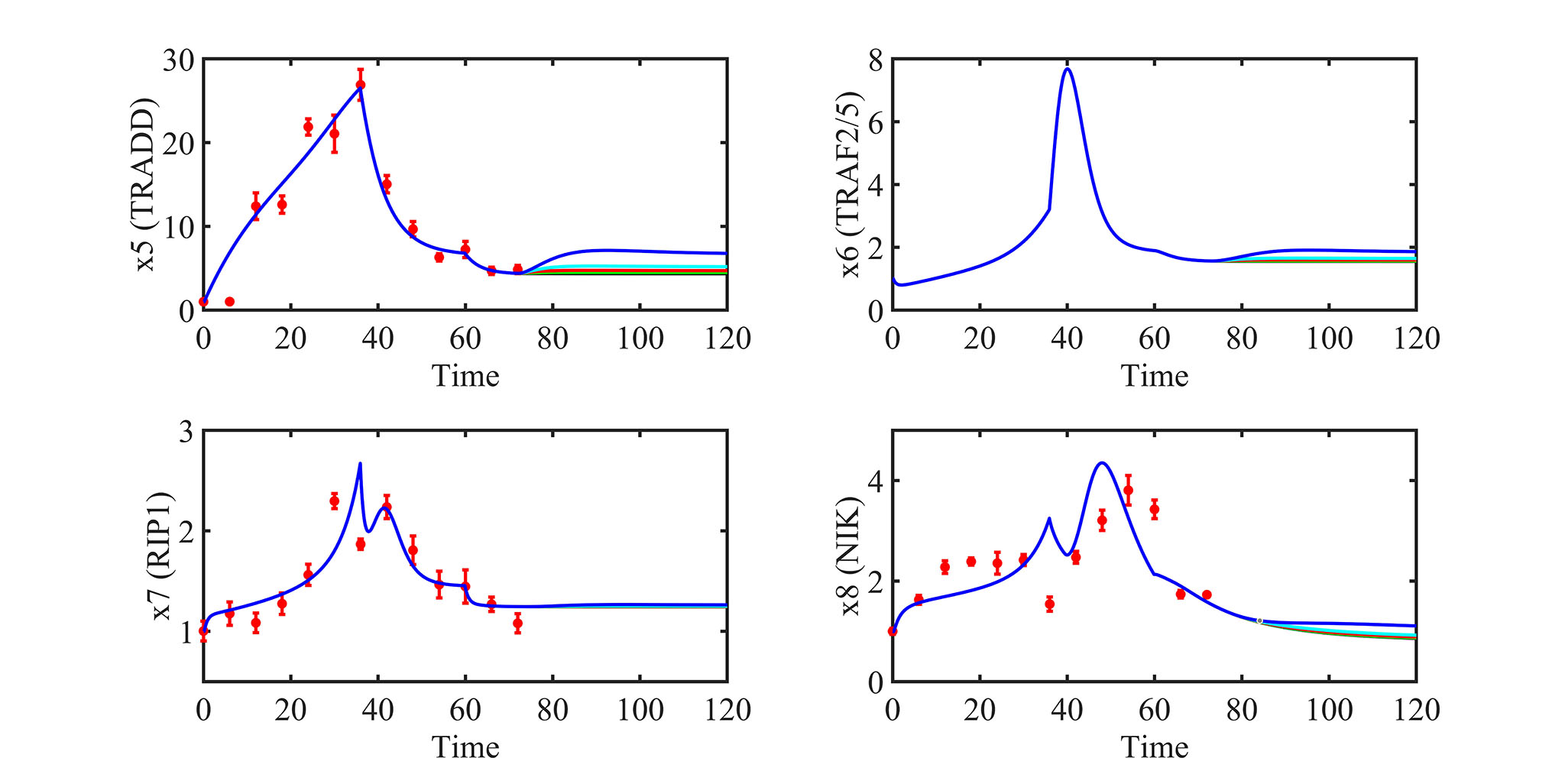

Supplement: Supplementary file 2 [file DataSheet1.zip › Supplementary material_image1/Parameter_b4(小)/22.jpg]

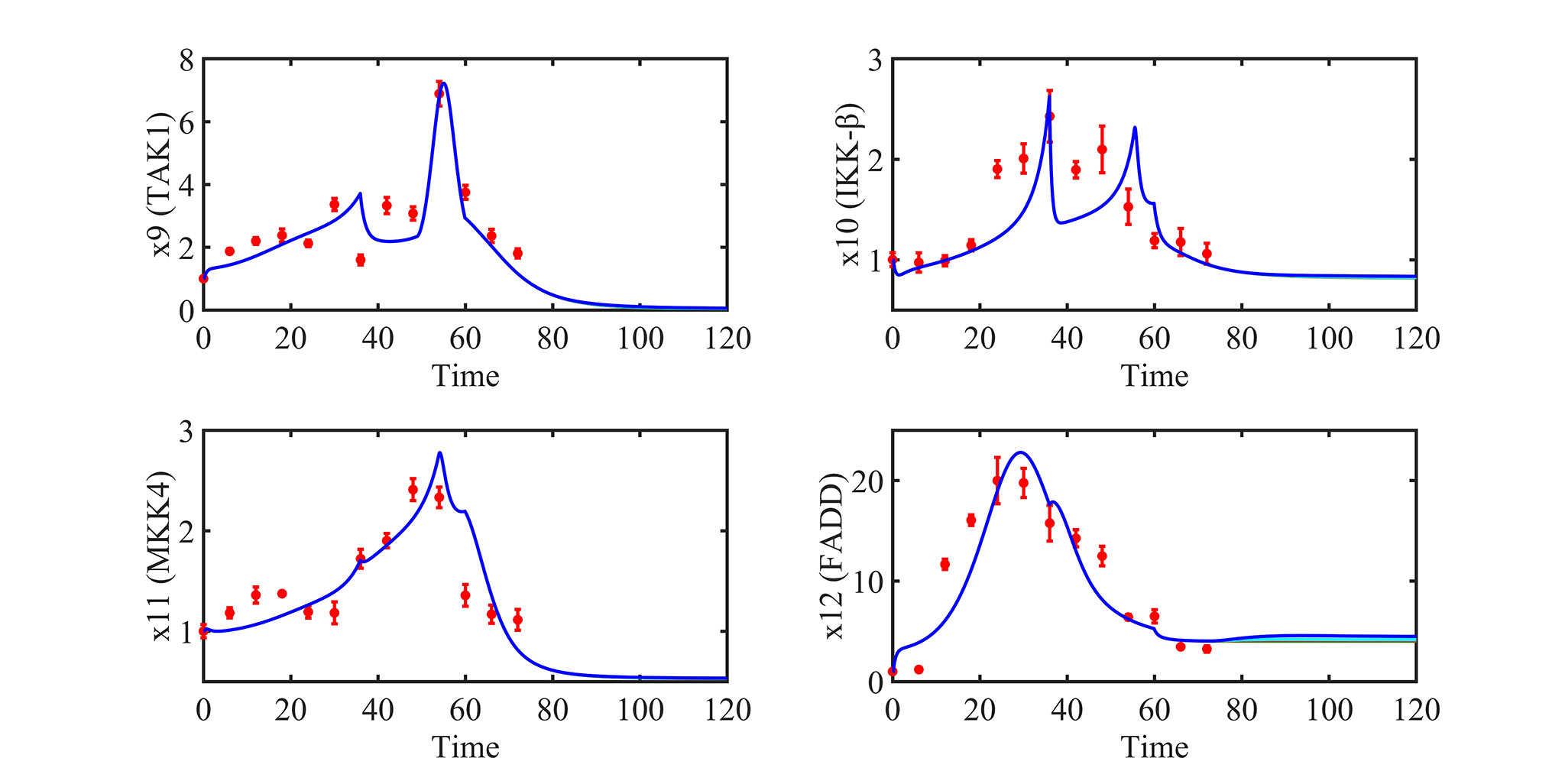

Supplement: Supplementary file 2 [file DataSheet1.zip › Supplementary material_image1/Parameter_b4(小)/33.jpg]

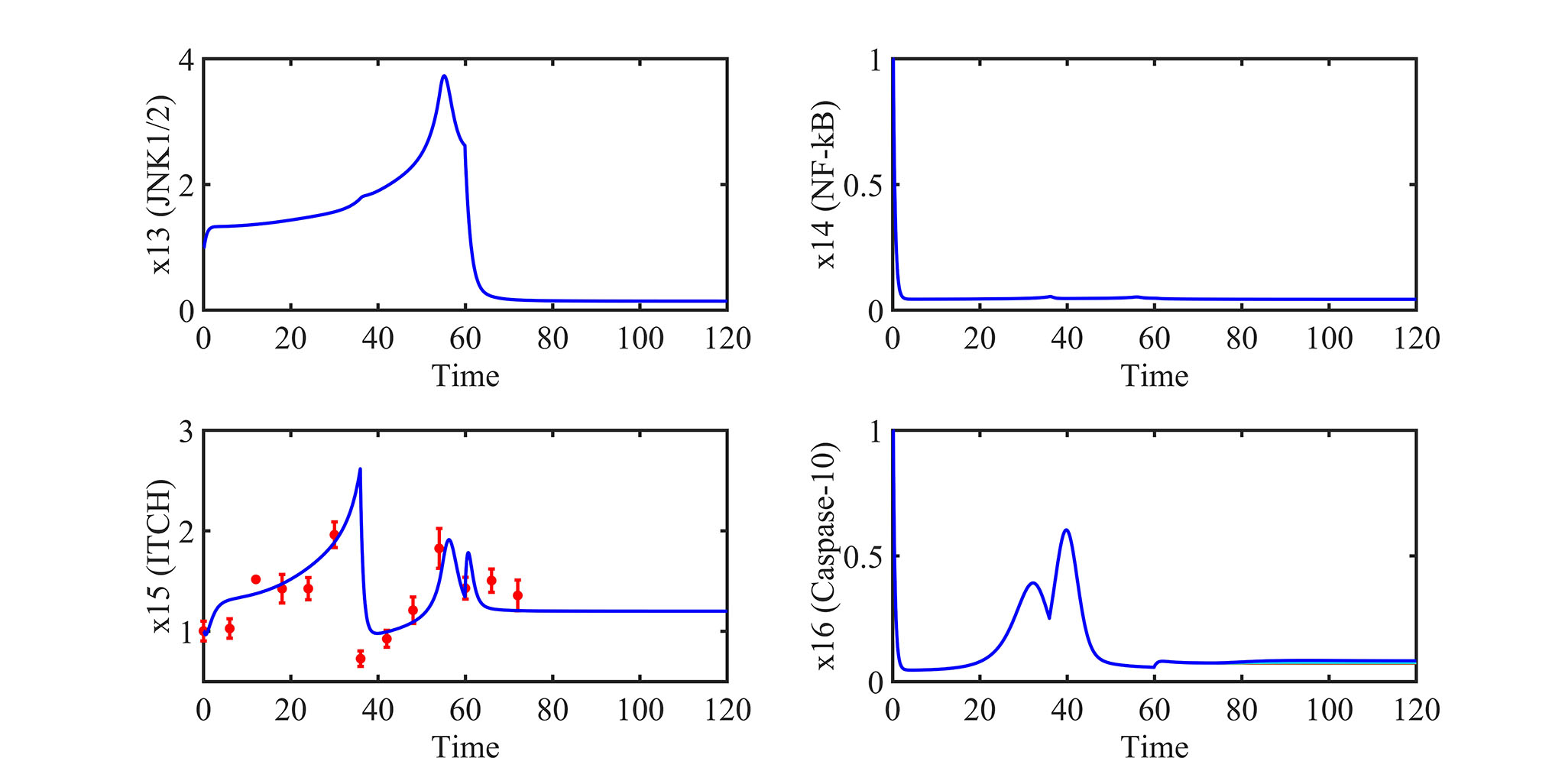

Supplement: Supplementary file 2 [file DataSheet1.zip › Supplementary material_image1/Parameter_b4(小)/44.jpg]

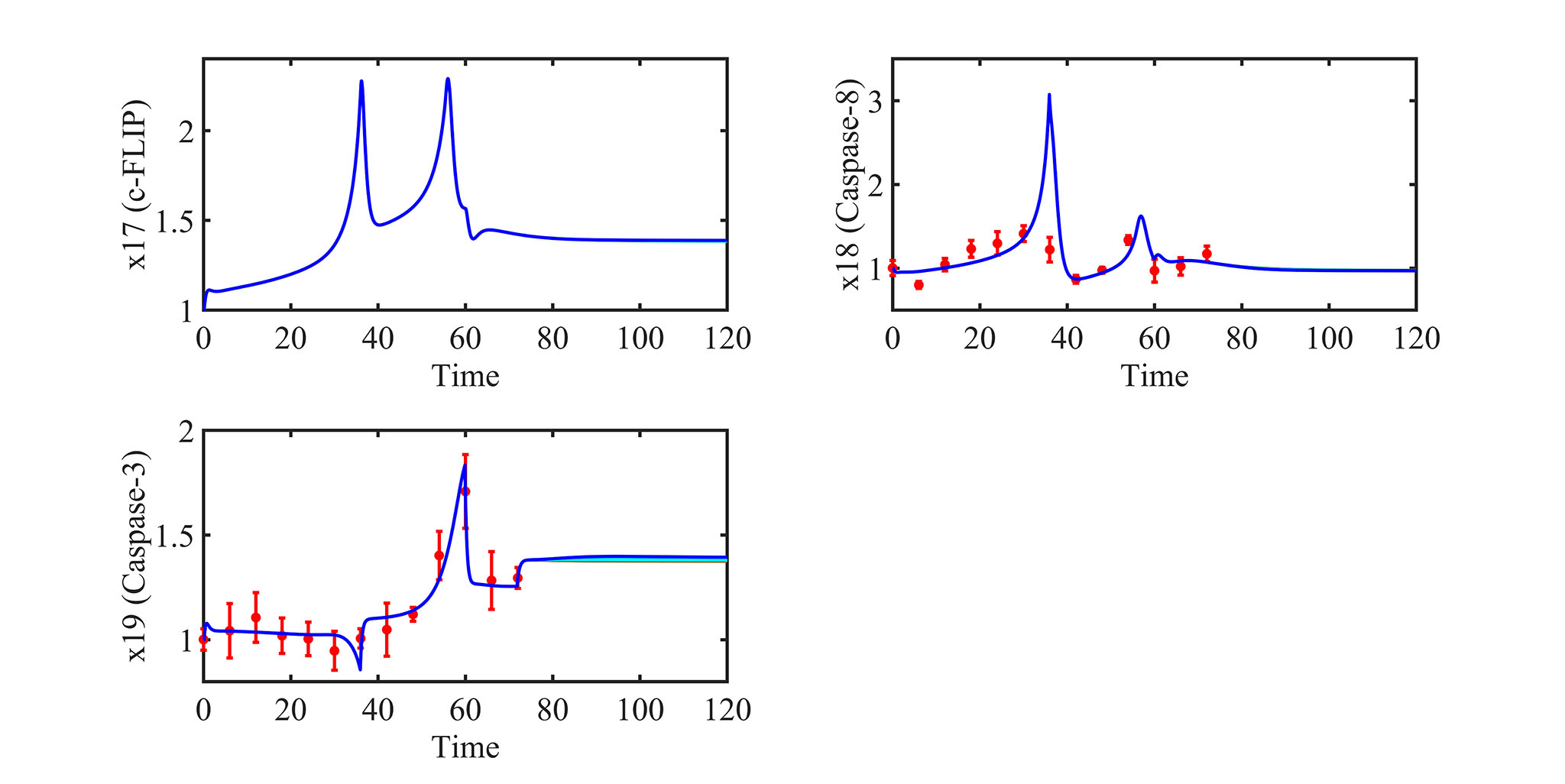

Supplement: Supplementary file 2 [file DataSheet1.zip › Supplementary material_image1/Parameter_b4(小)/55.jpg]

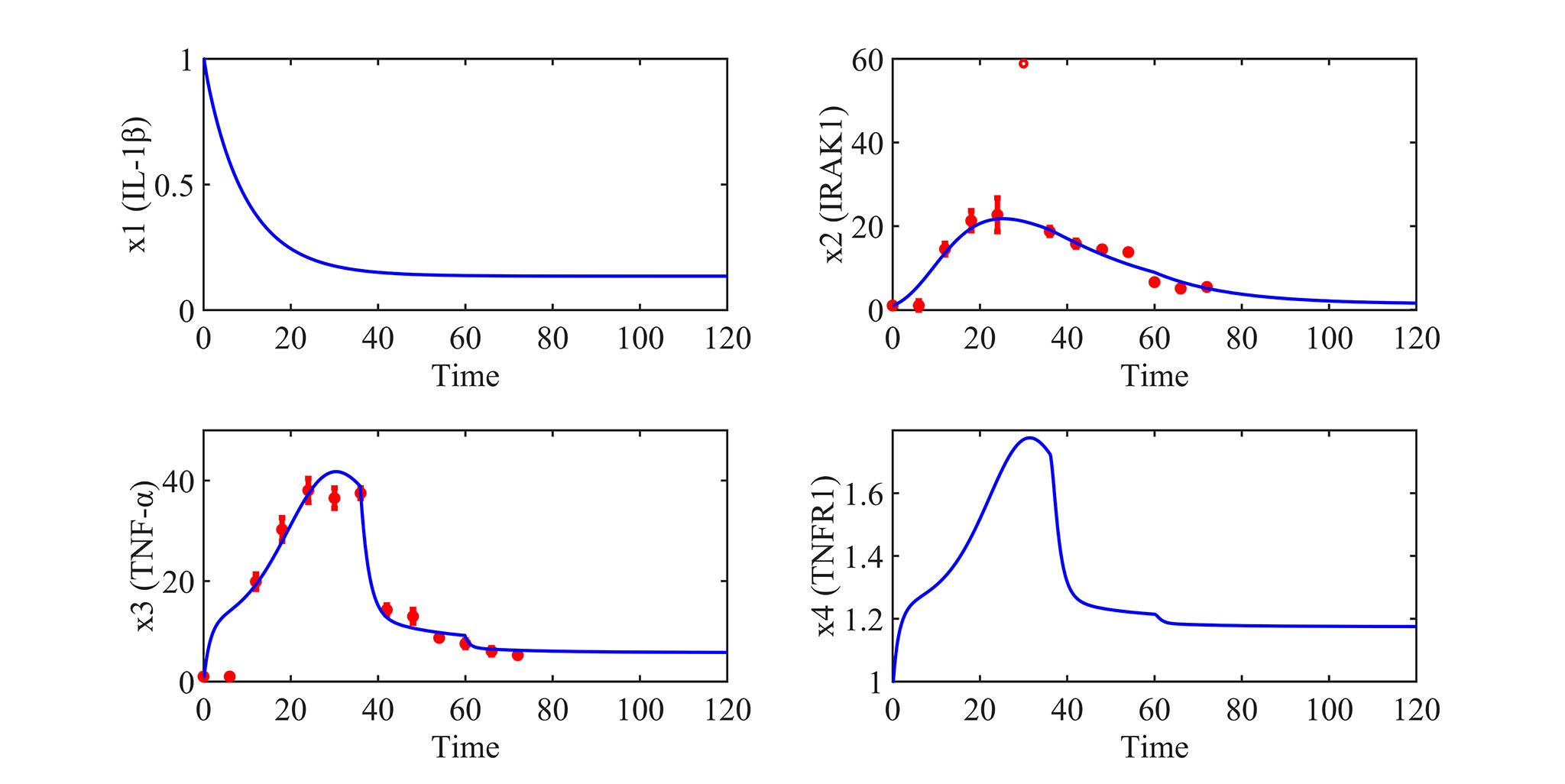

Supplement: Supplementary file 2 [file DataSheet1.zip › Supplementary material_image1/Parameter_b5(小)/1.jpg]

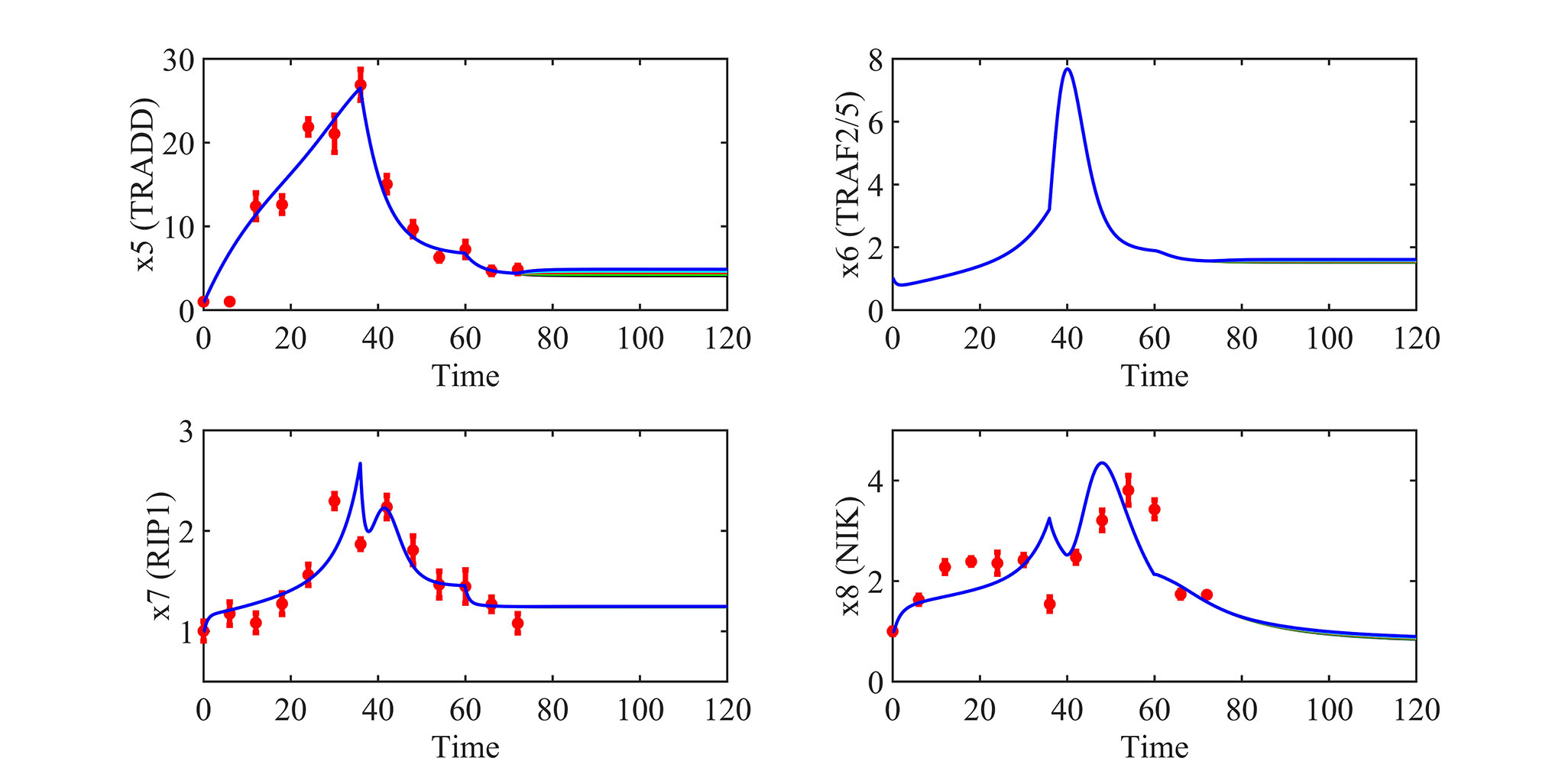

Supplement: Supplementary file 2 [file DataSheet1.zip › Supplementary material_image1/Parameter_b5(小)/2.jpg]

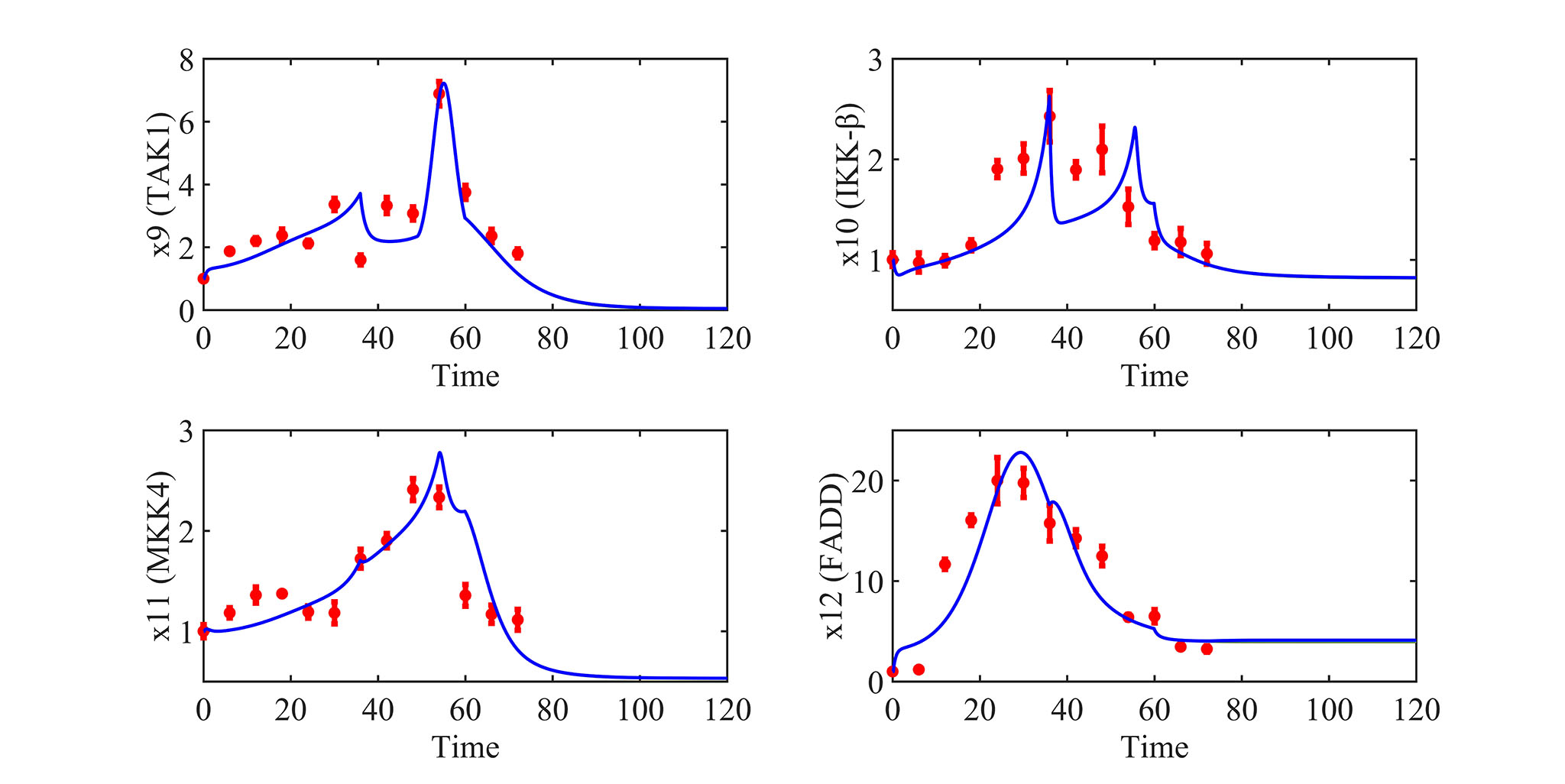

Supplement: Supplementary file 2 [file DataSheet1.zip › Supplementary material_image1/Parameter_b5(小)/3.jpg]

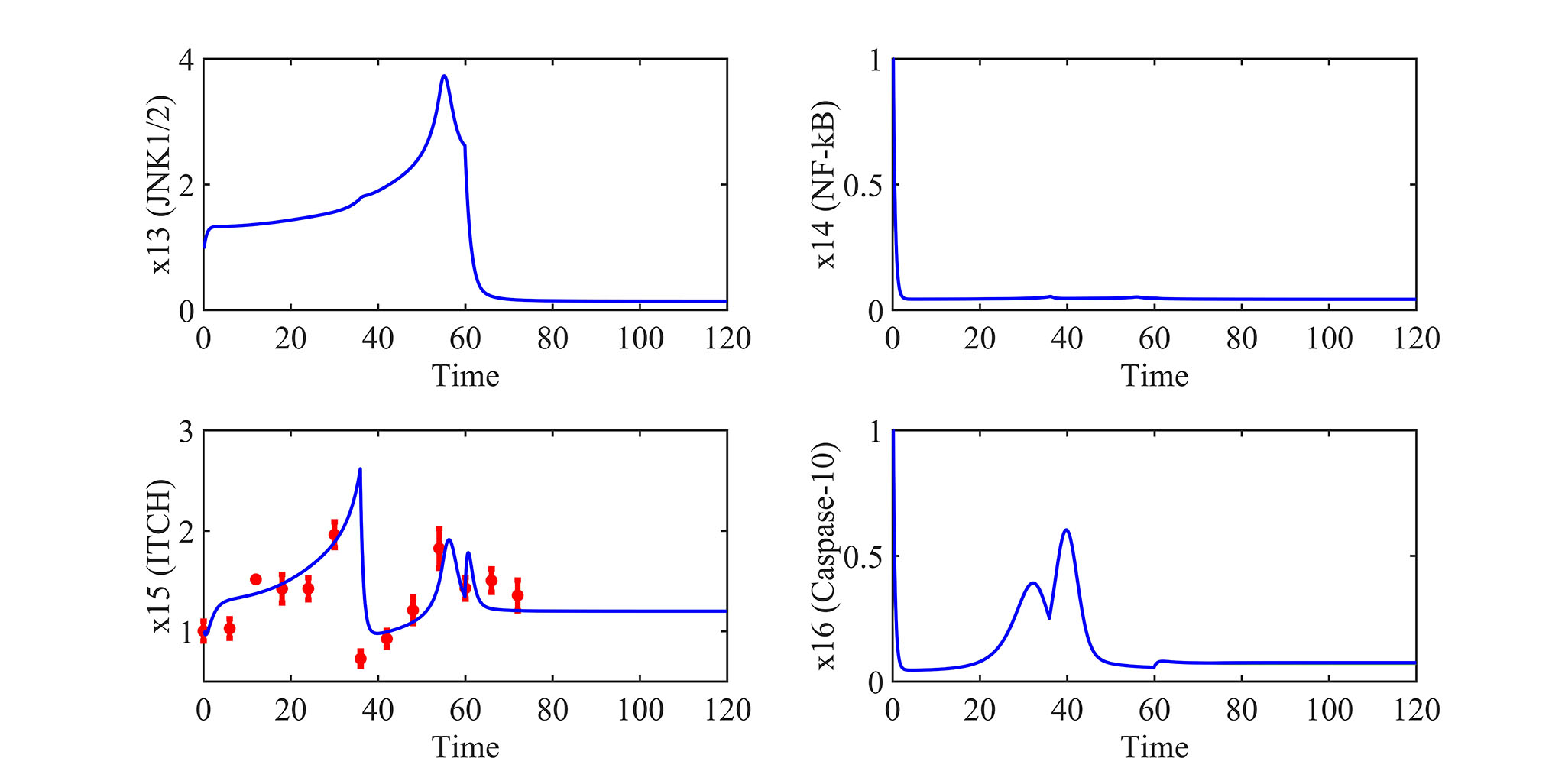

Supplement: Supplementary file 2 [file DataSheet1.zip › Supplementary material_image1/Parameter_b5(小)/4.jpg]

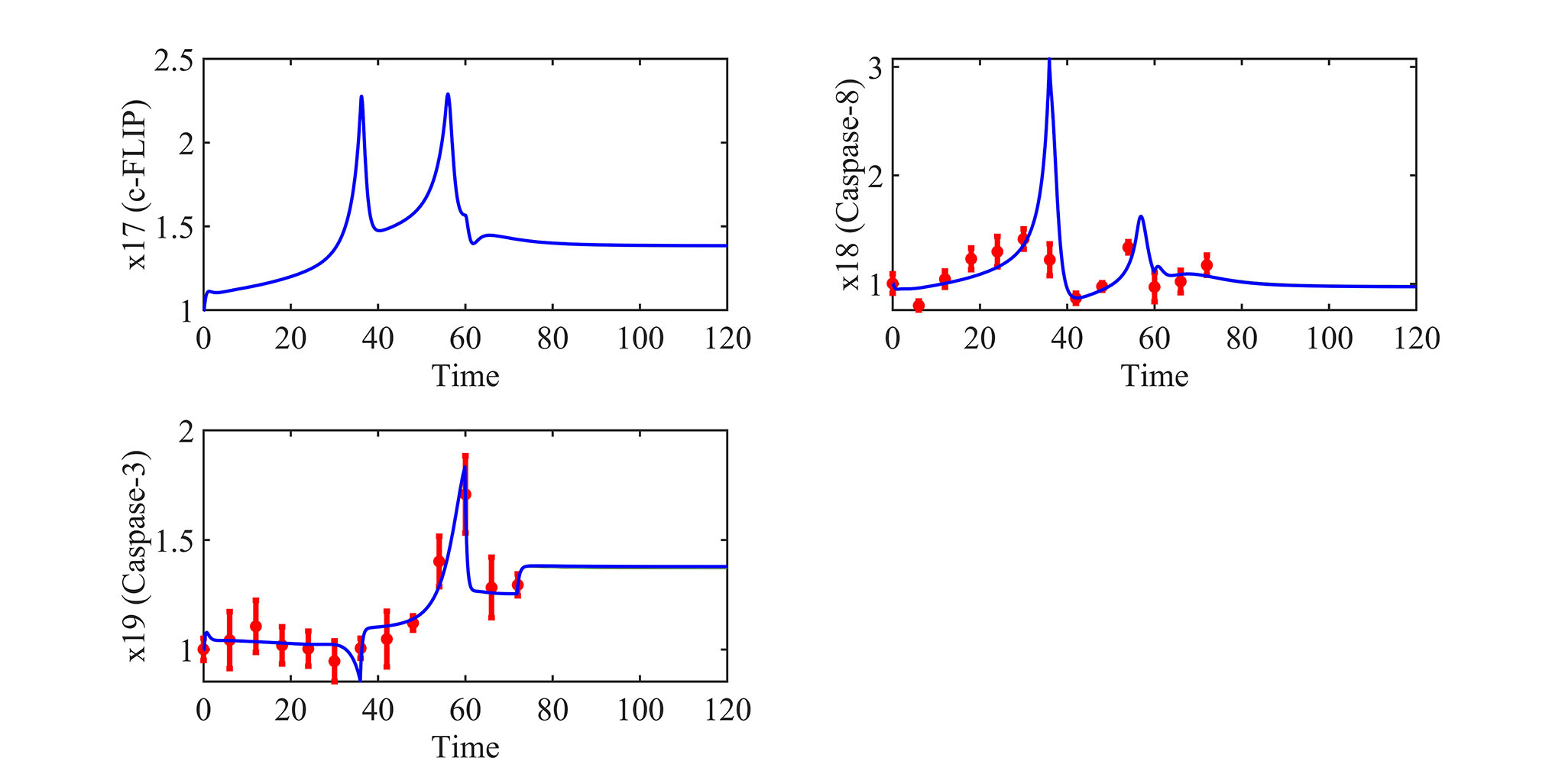

Supplement: Supplementary file 2 [file DataSheet1.zip › Supplementary material_image1/Parameter_b5(小)/5.jpg]

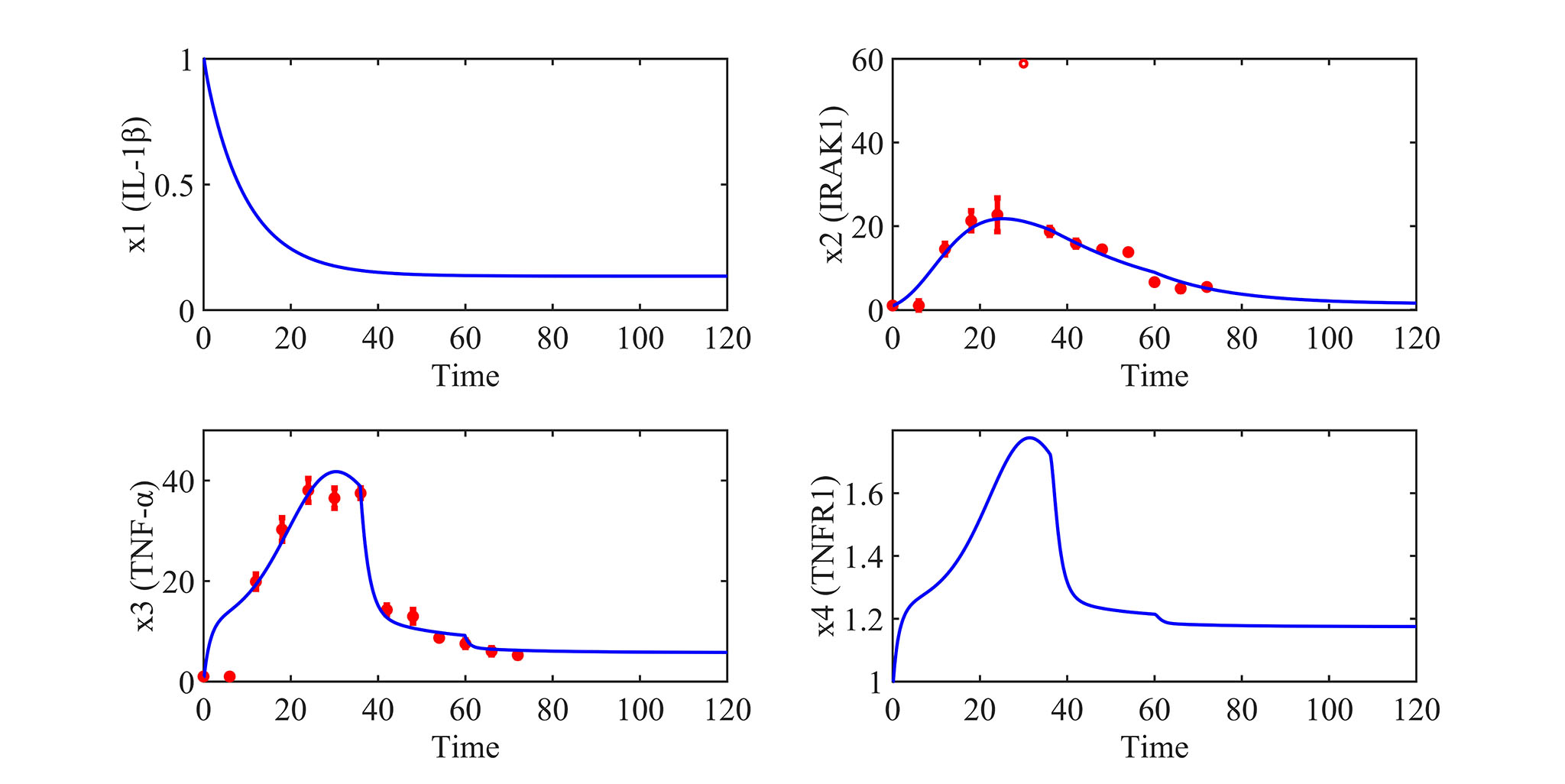

Supplement: Supplementary file 2 [file DataSheet1.zip › Supplementary material_image1/Parameter_b6(小)/1.jpg]

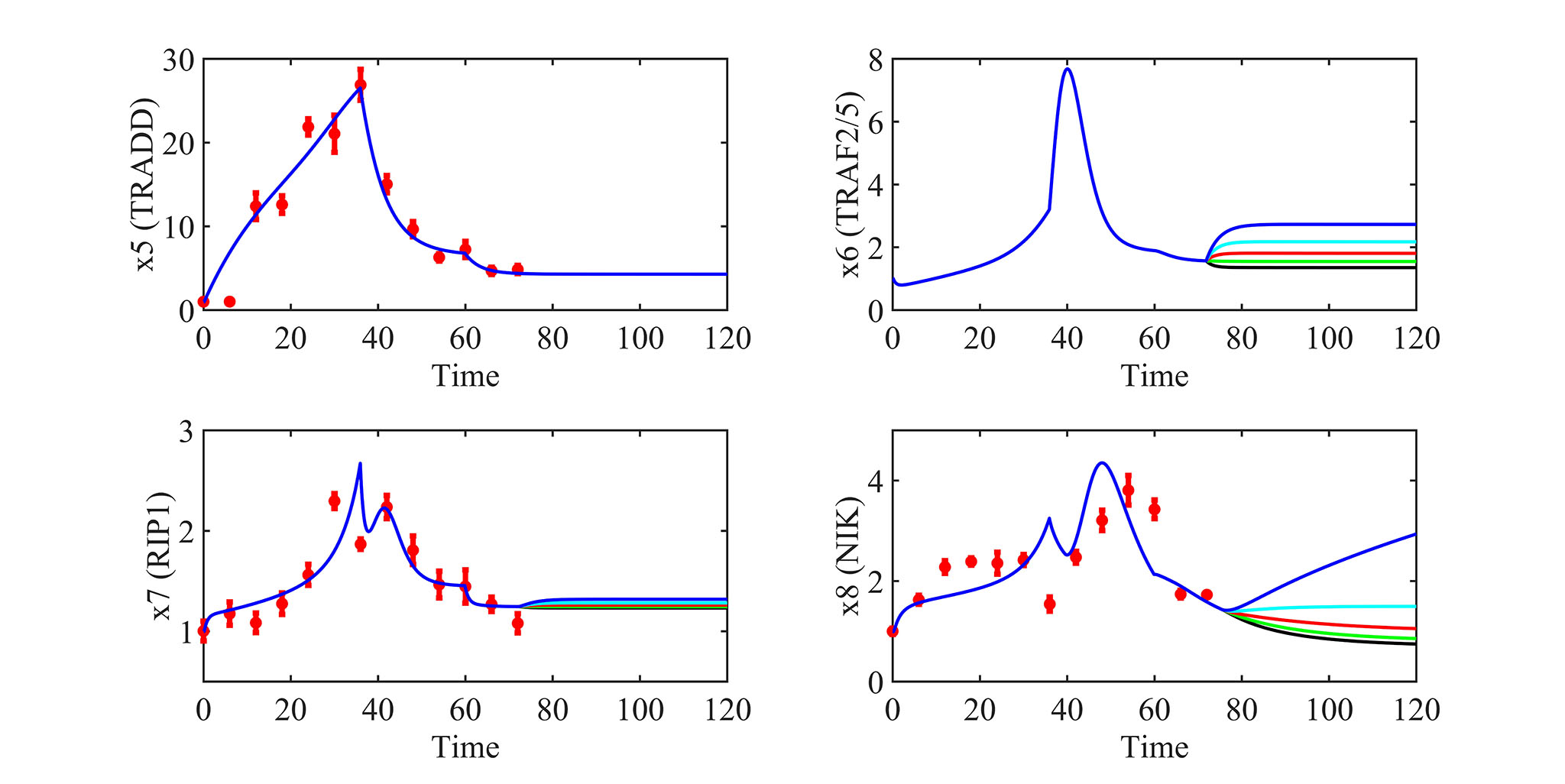

Supplement: Supplementary file 2 [file DataSheet1.zip › Supplementary material_image1/Parameter_b6(小)/2.jpg]

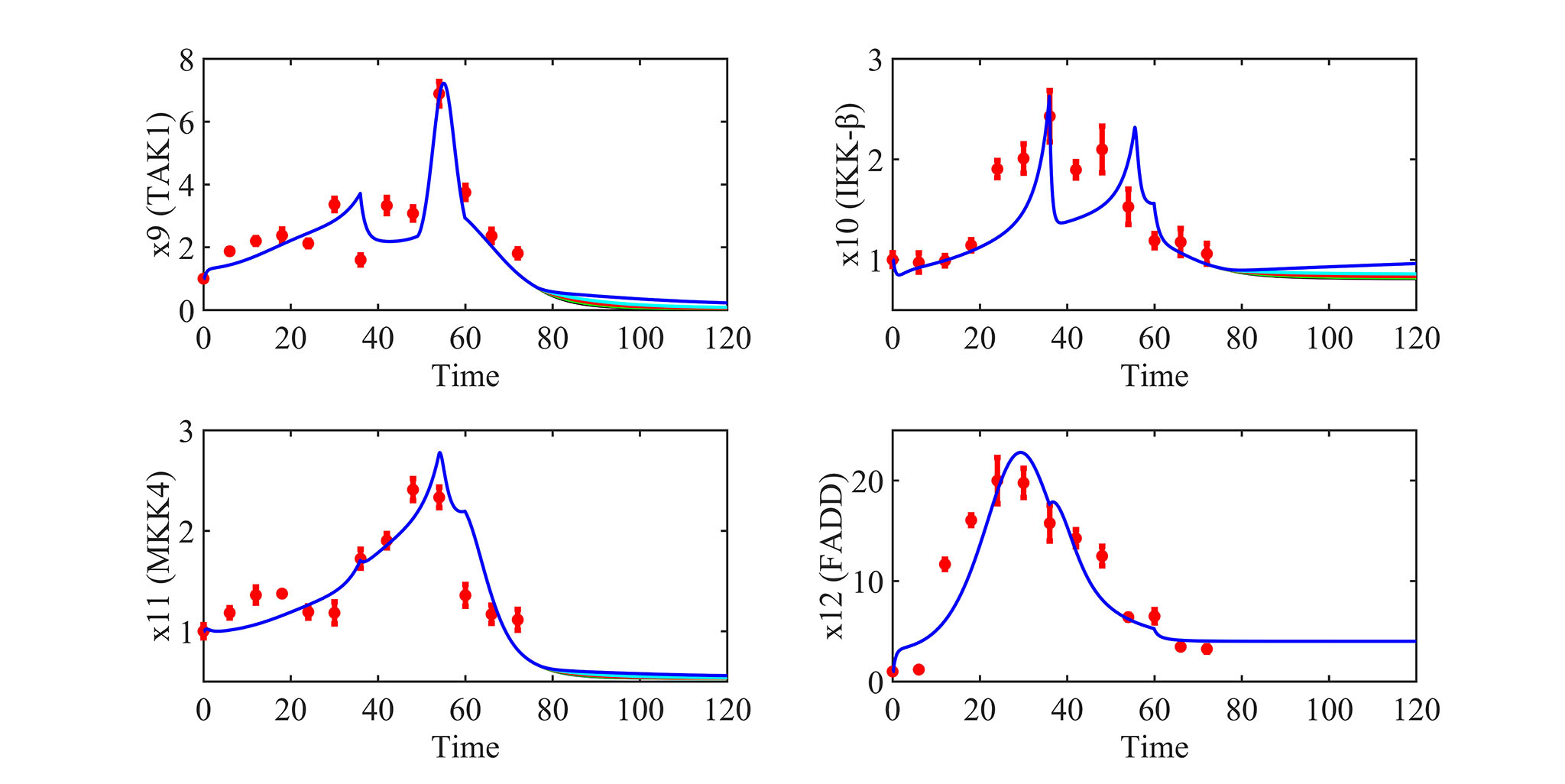

Supplement: Supplementary file 2 [file DataSheet1.zip › Supplementary material_image1/Parameter_b6(小)/3.jpg]

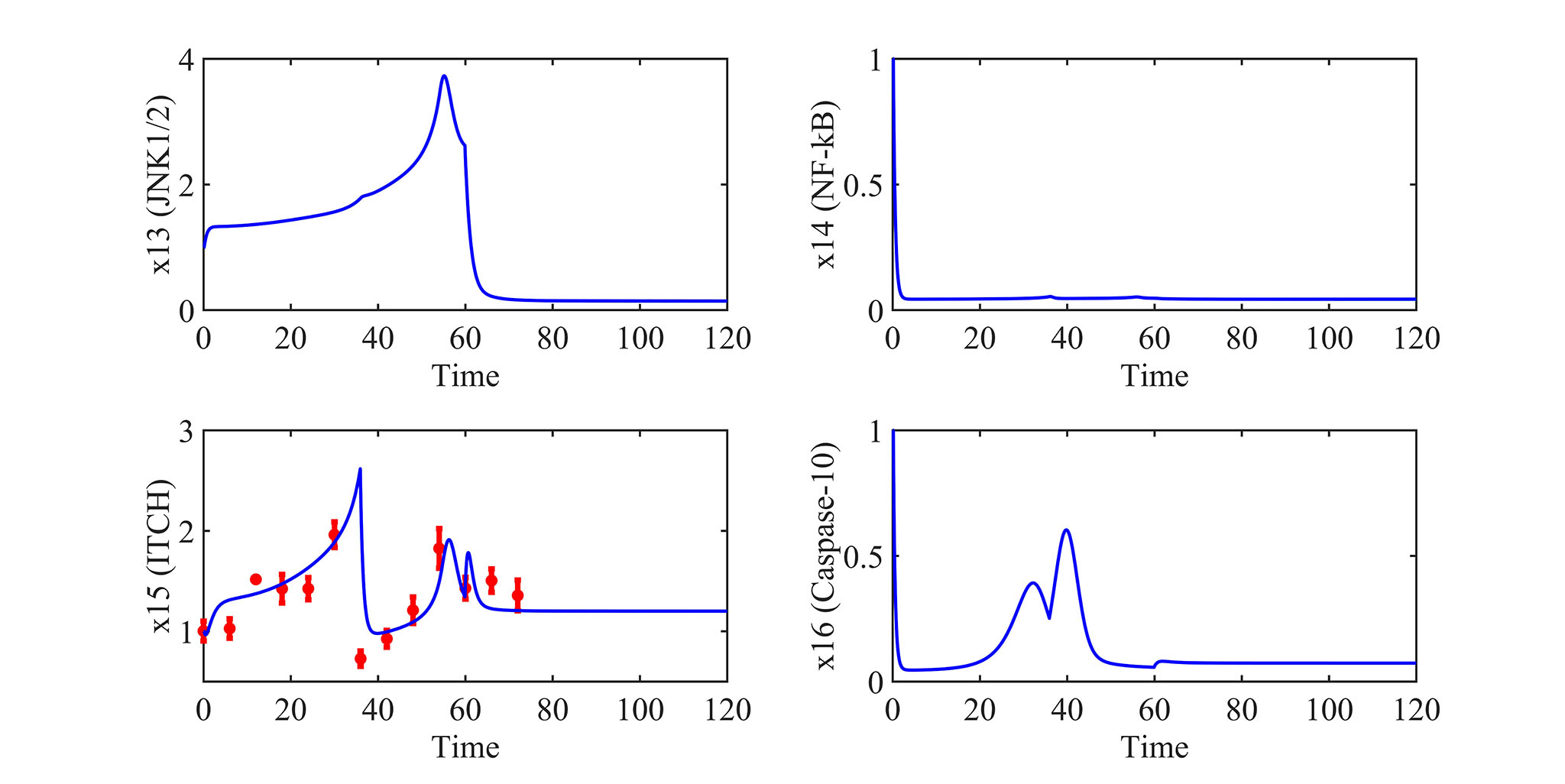

Supplement: Supplementary file 2 [file DataSheet1.zip › Supplementary material_image1/Parameter_b6(小)/4.jpg]

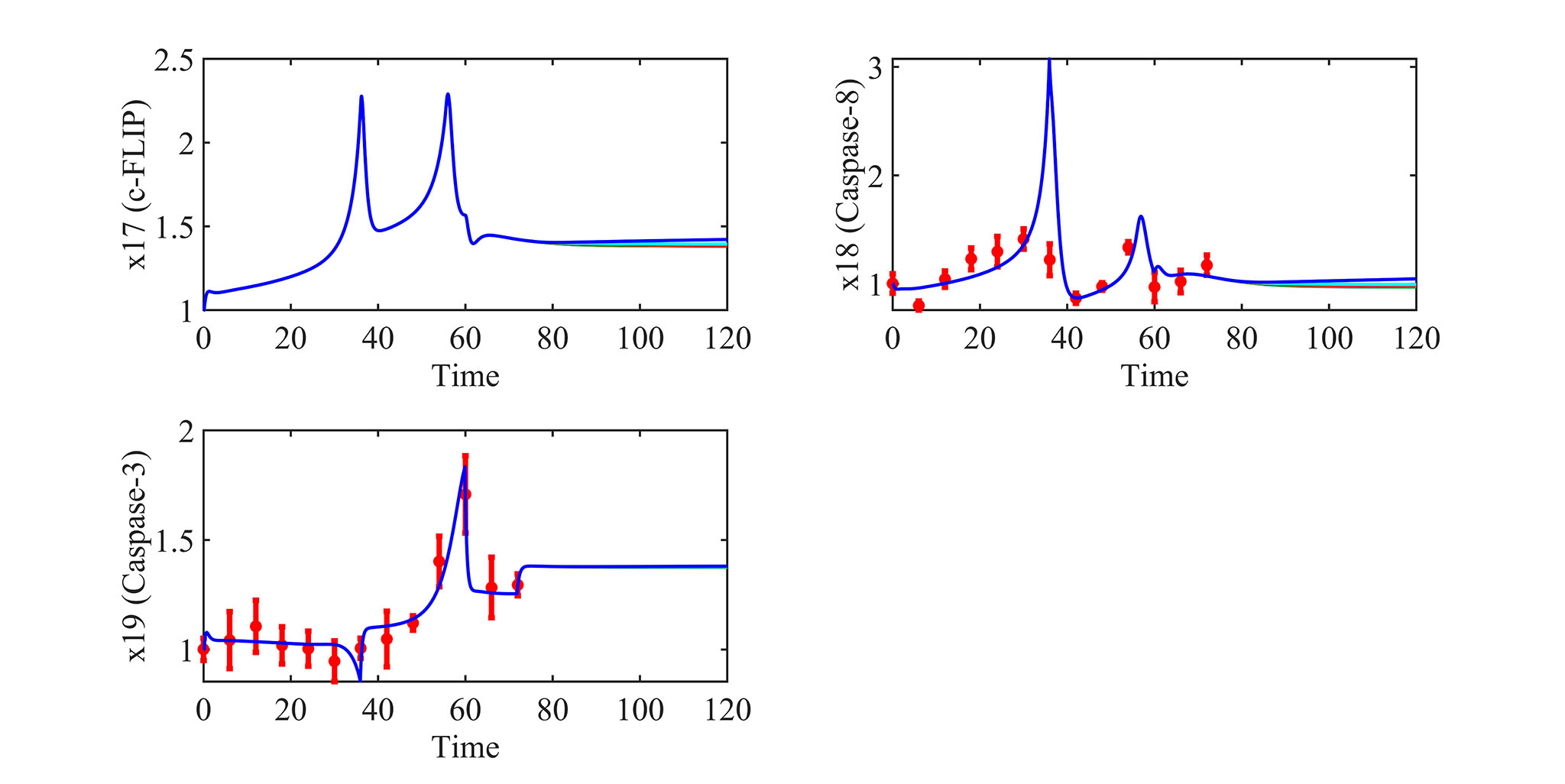

Supplement: Supplementary file 2 [file DataSheet1.zip › Supplementary material_image1/Parameter_b6(小)/5.jpg]

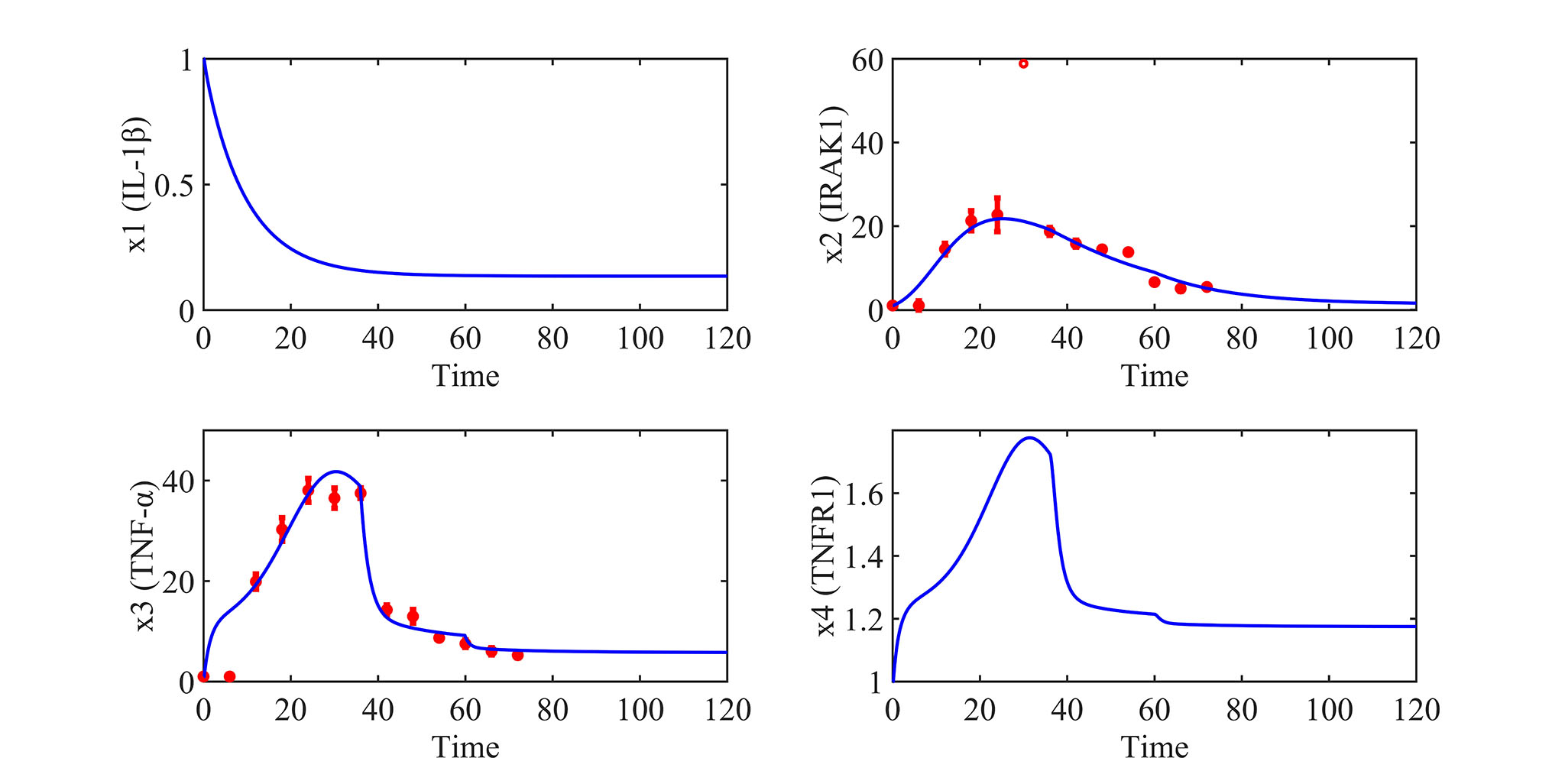

Supplement: Supplementary file 2 [file DataSheet1.zip › Supplementary material_image1/Parameter_b7(小)/1.jpg]

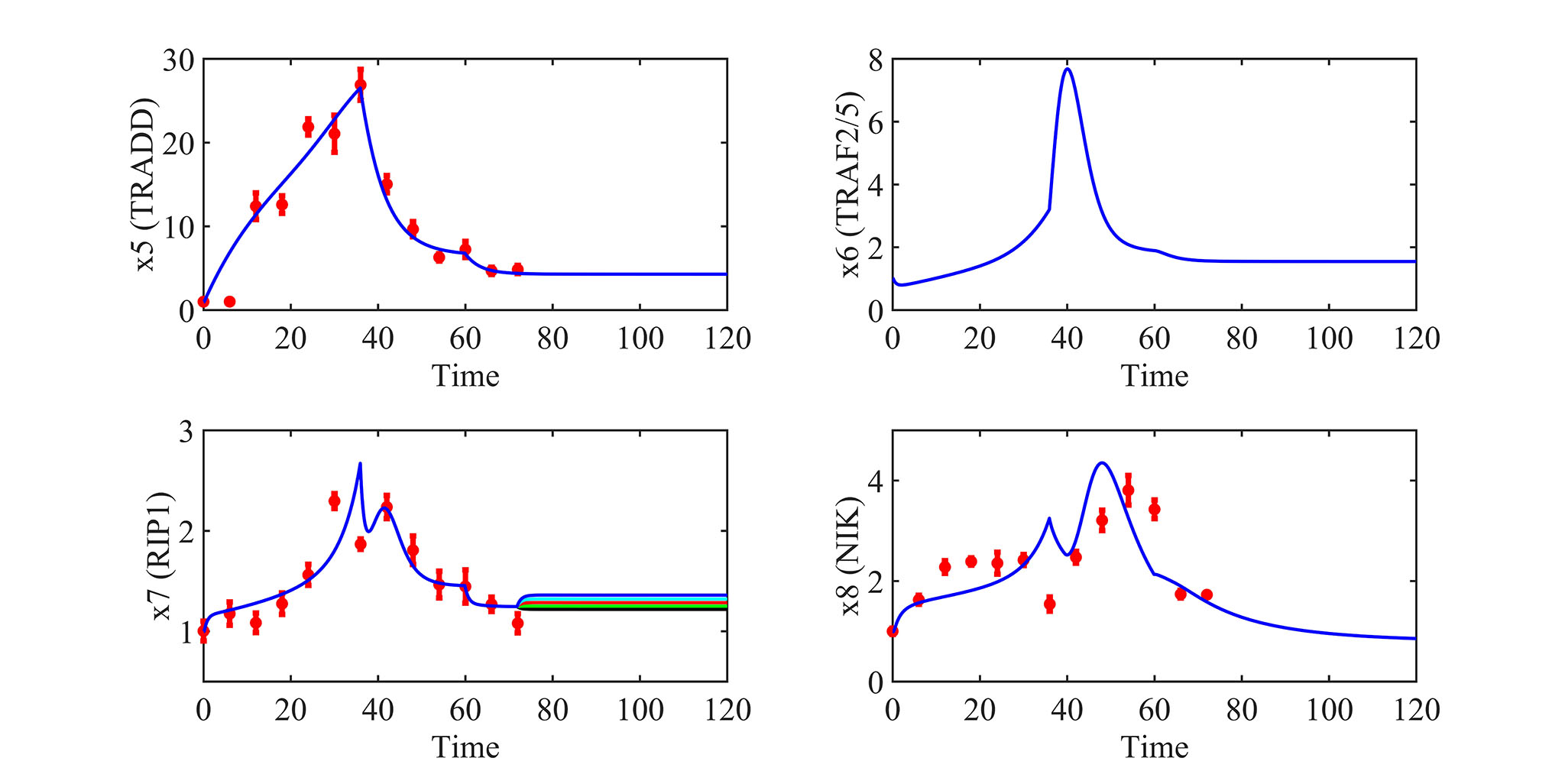

Supplement: Supplementary file 2 [file DataSheet1.zip › Supplementary material_image1/Parameter_b7(小)/2.jpg]

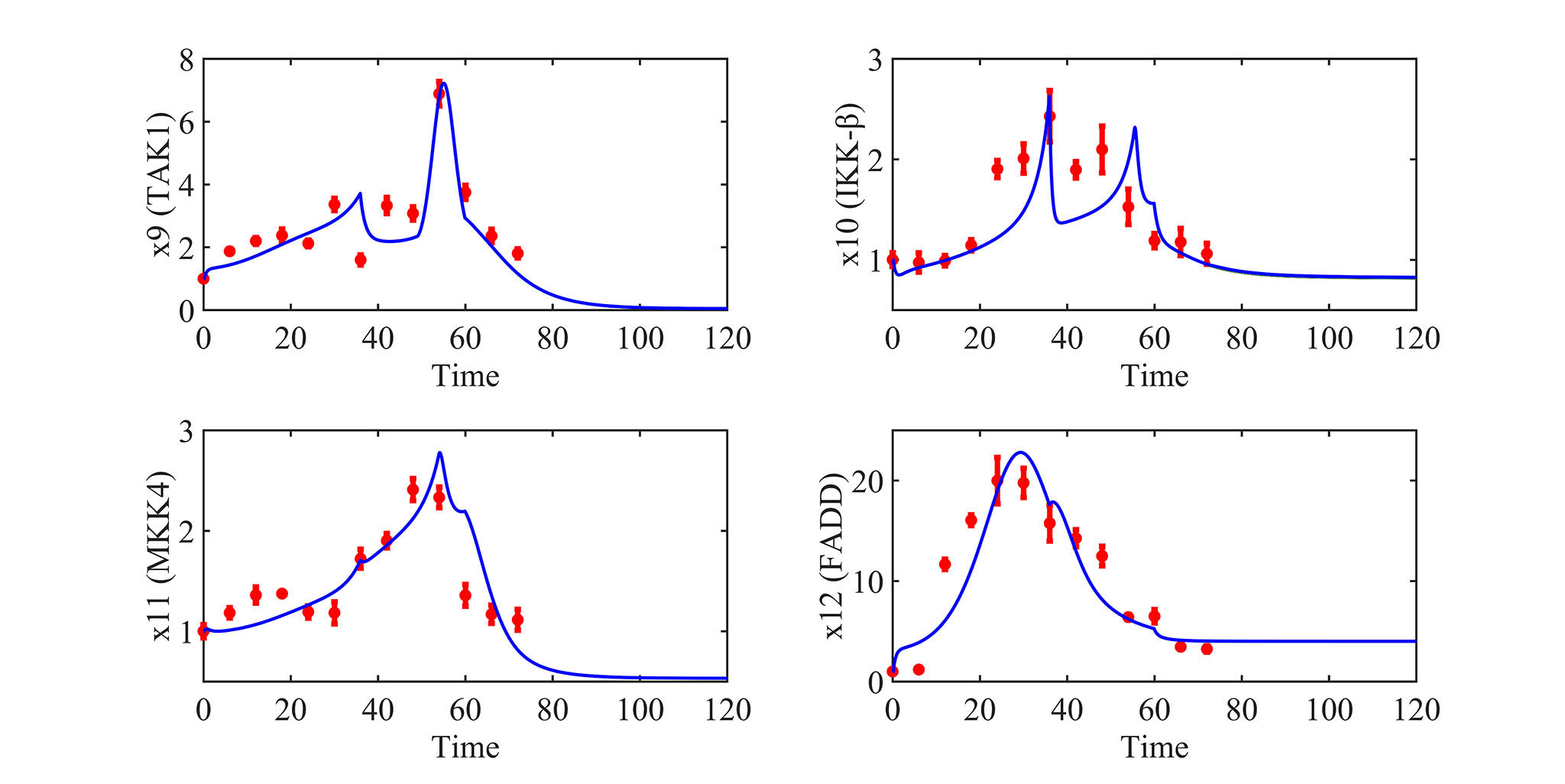

Supplement: Supplementary file 2 [file DataSheet1.zip › Supplementary material_image1/Parameter_b7(小)/3.jpg]

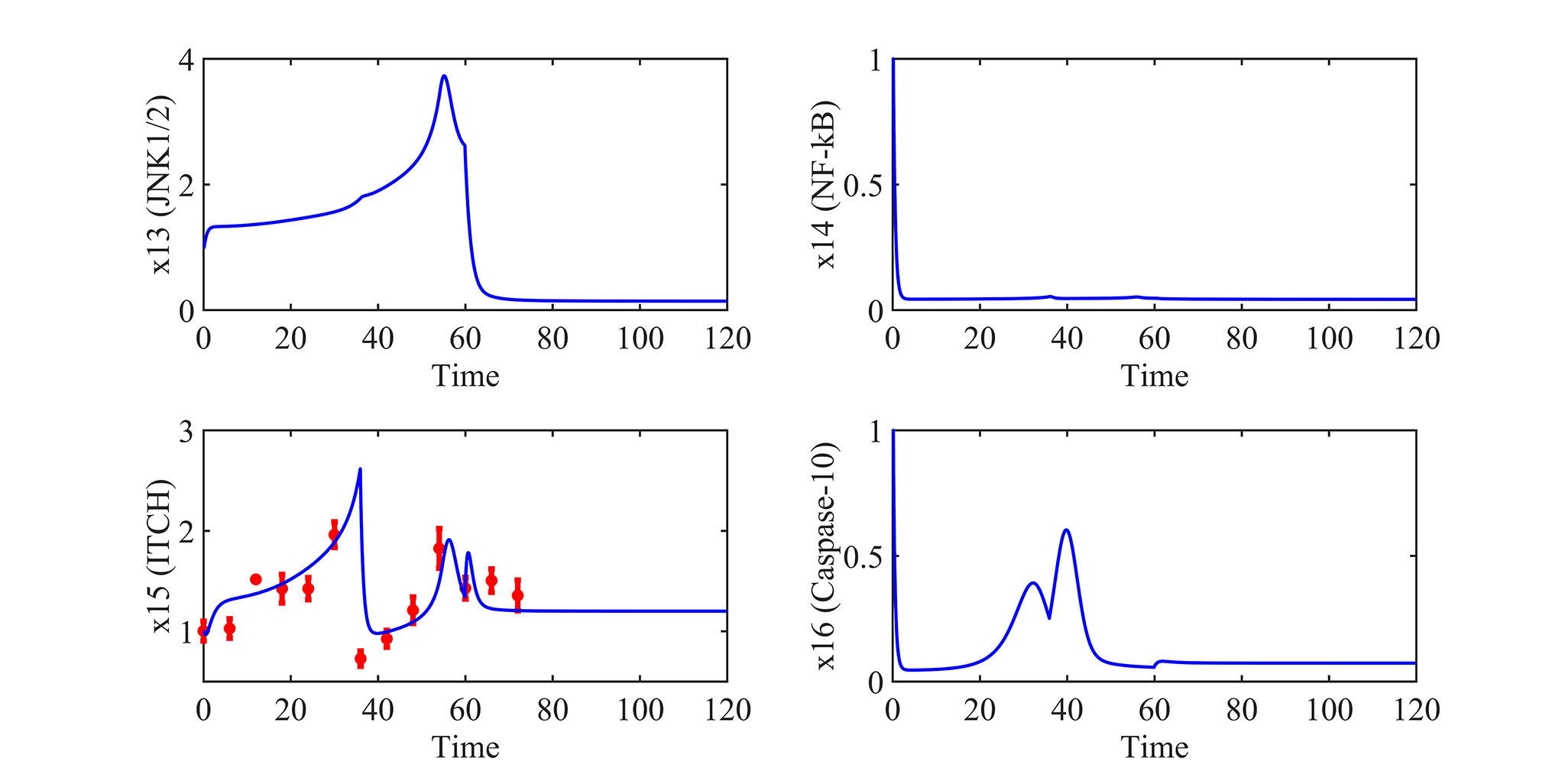

Supplement: Supplementary file 2 [file DataSheet1.zip › Supplementary material_image1/Parameter_b7(小)/4.jpg]

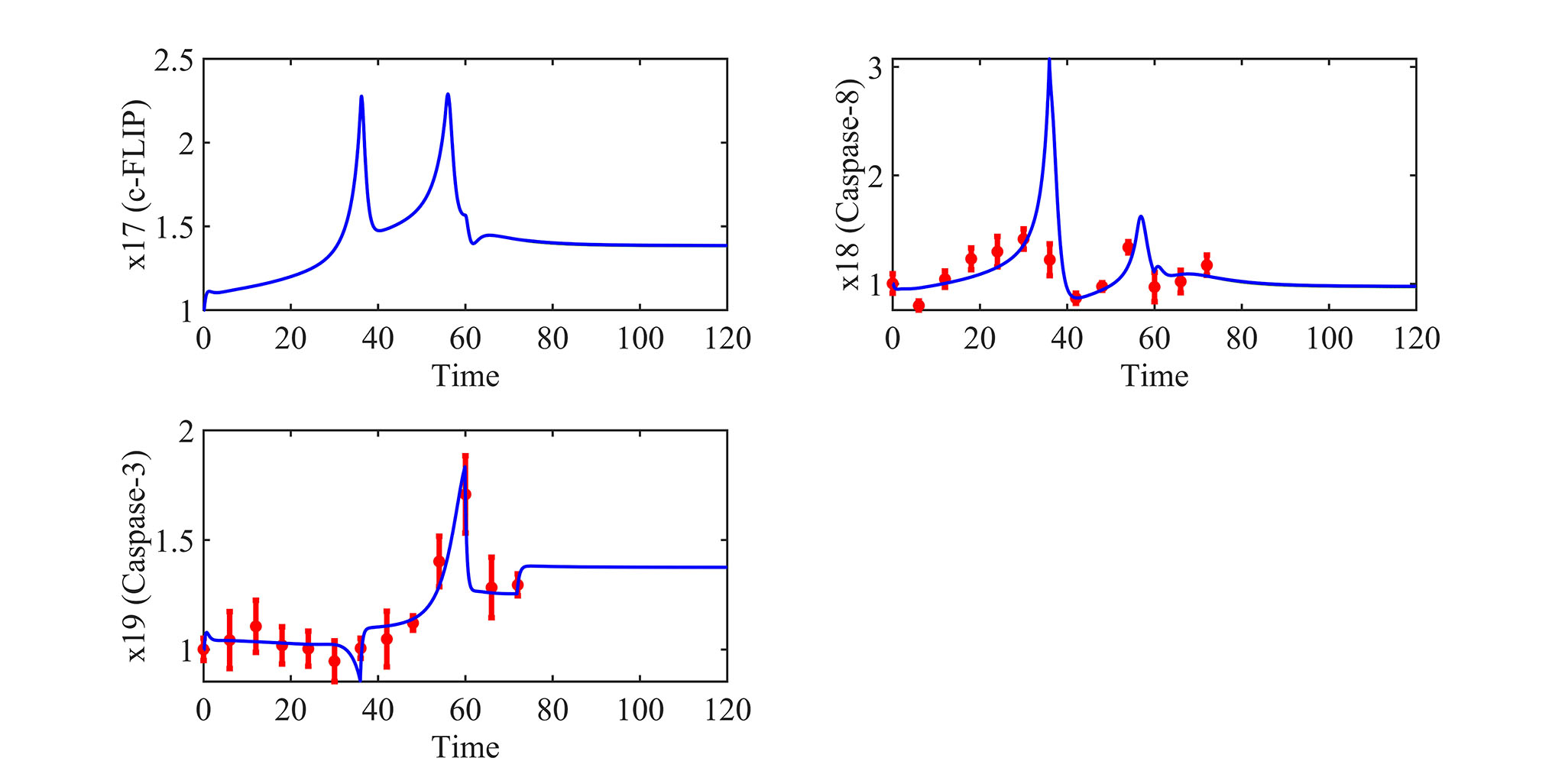

Supplement: Supplementary file 2 [file DataSheet1.zip › Supplementary material_image1/Parameter_b7(小)/5.jpg]

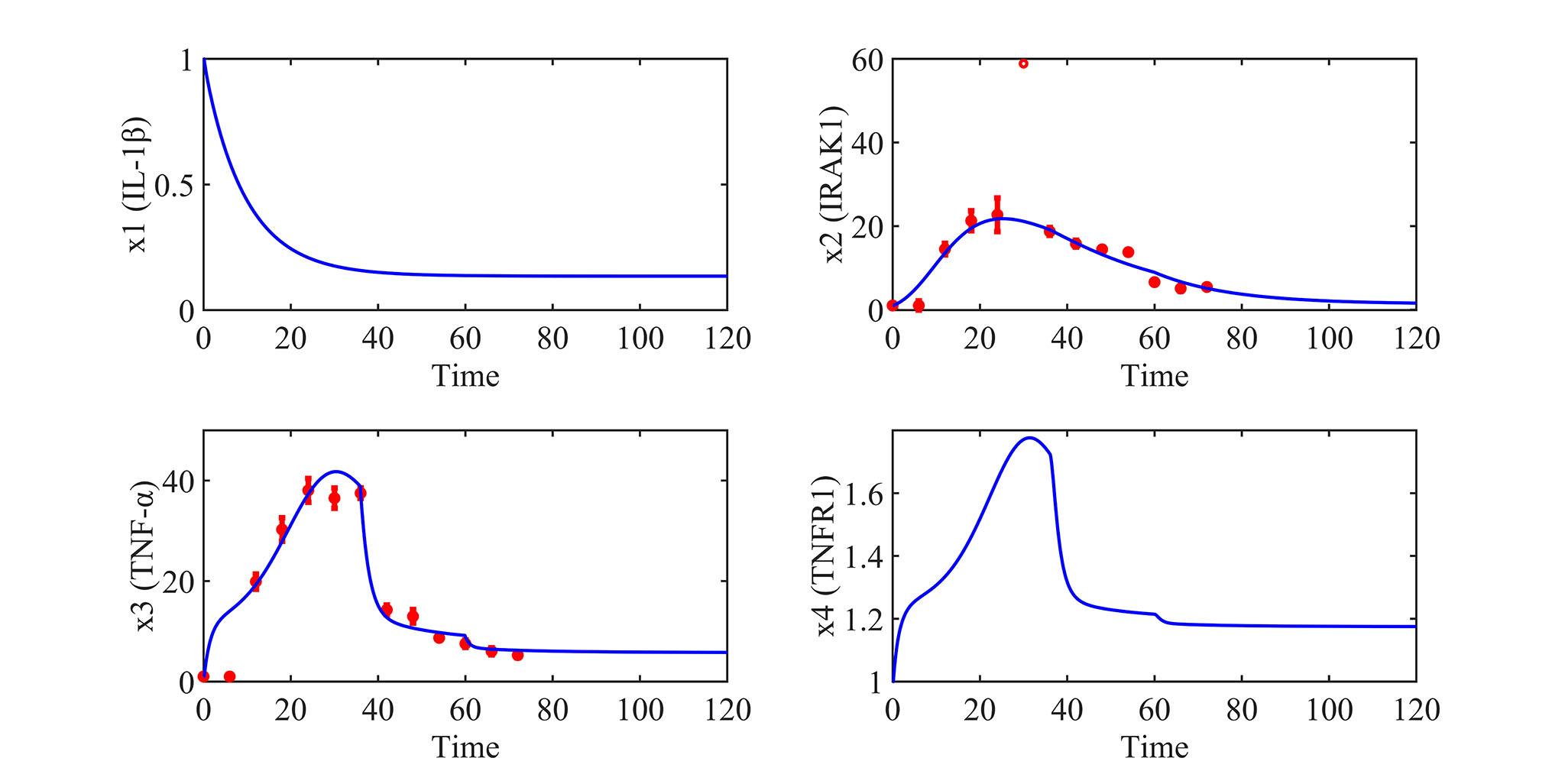

Supplement: Supplementary file 2 [file DataSheet1.zip › Supplementary material_image1/Parameter_b8(小)/1.jpg]

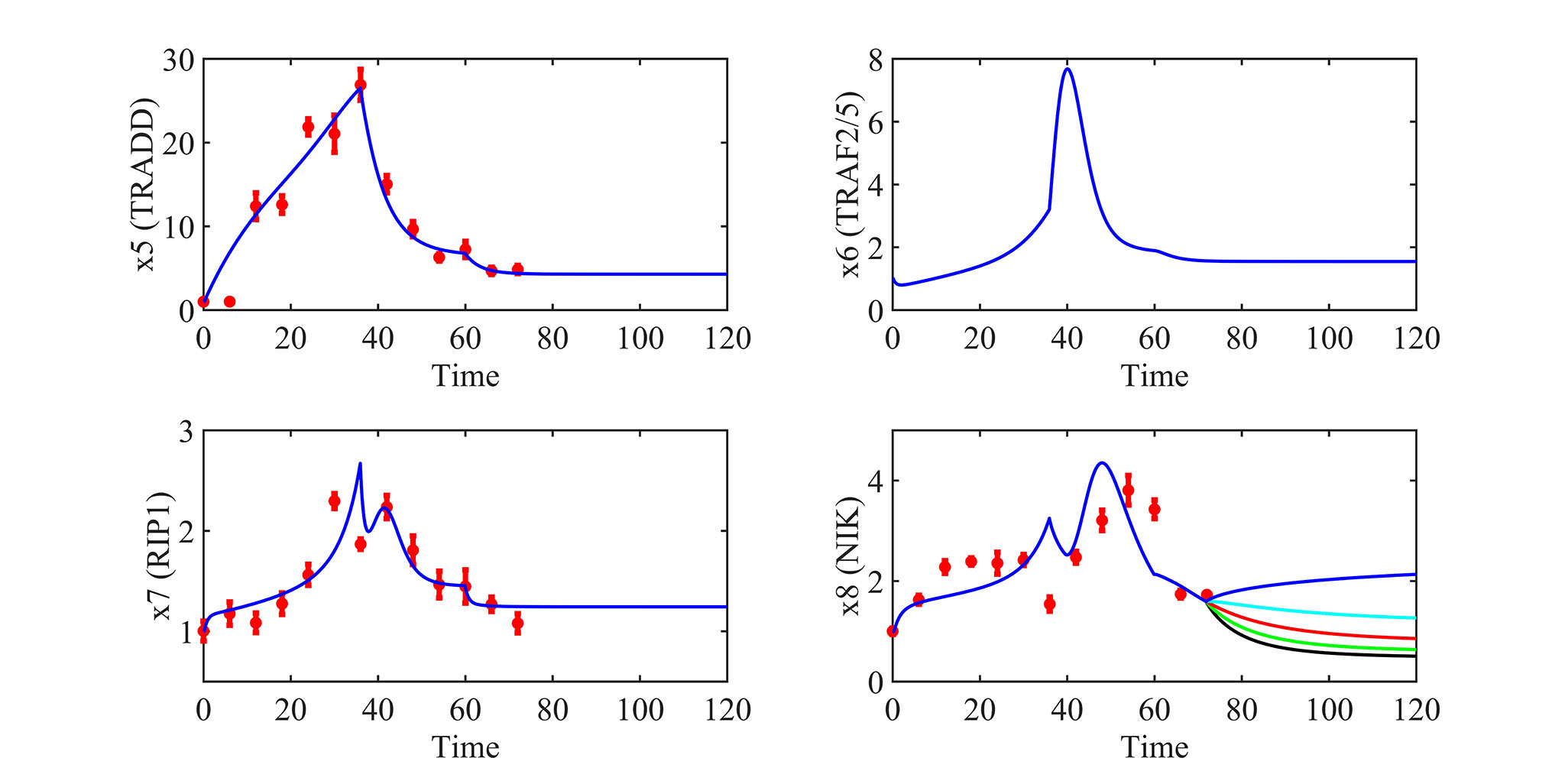

Supplement: Supplementary file 2 [file DataSheet1.zip › Supplementary material_image1/Parameter_b8(小)/2.jpg]

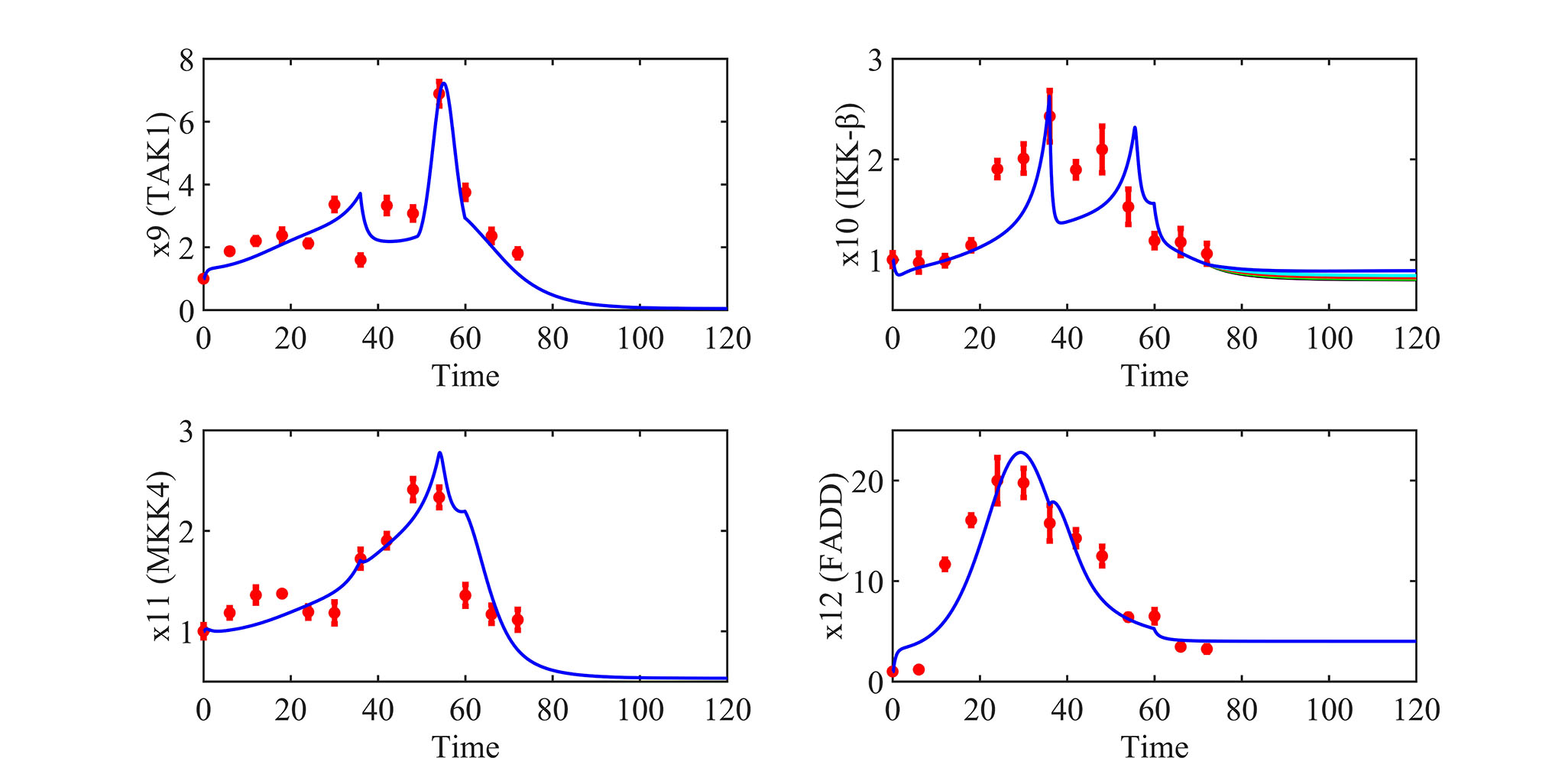

Supplement: Supplementary file 2 [file DataSheet1.zip › Supplementary material_image1/Parameter_b8(小)/3.jpg]

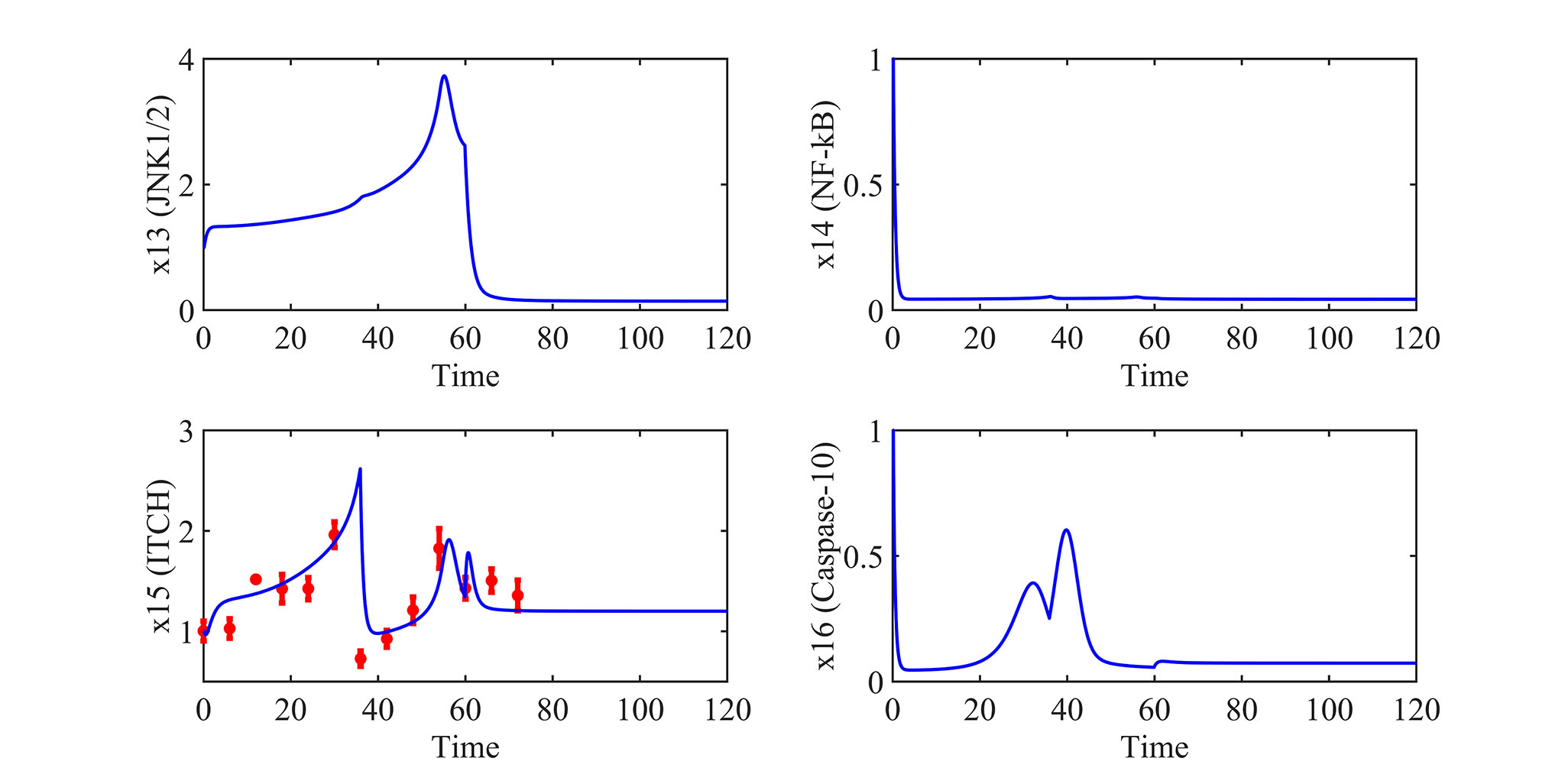

Supplement: Supplementary file 2 [file DataSheet1.zip › Supplementary material_image1/Parameter_b8(小)/4.jpg]

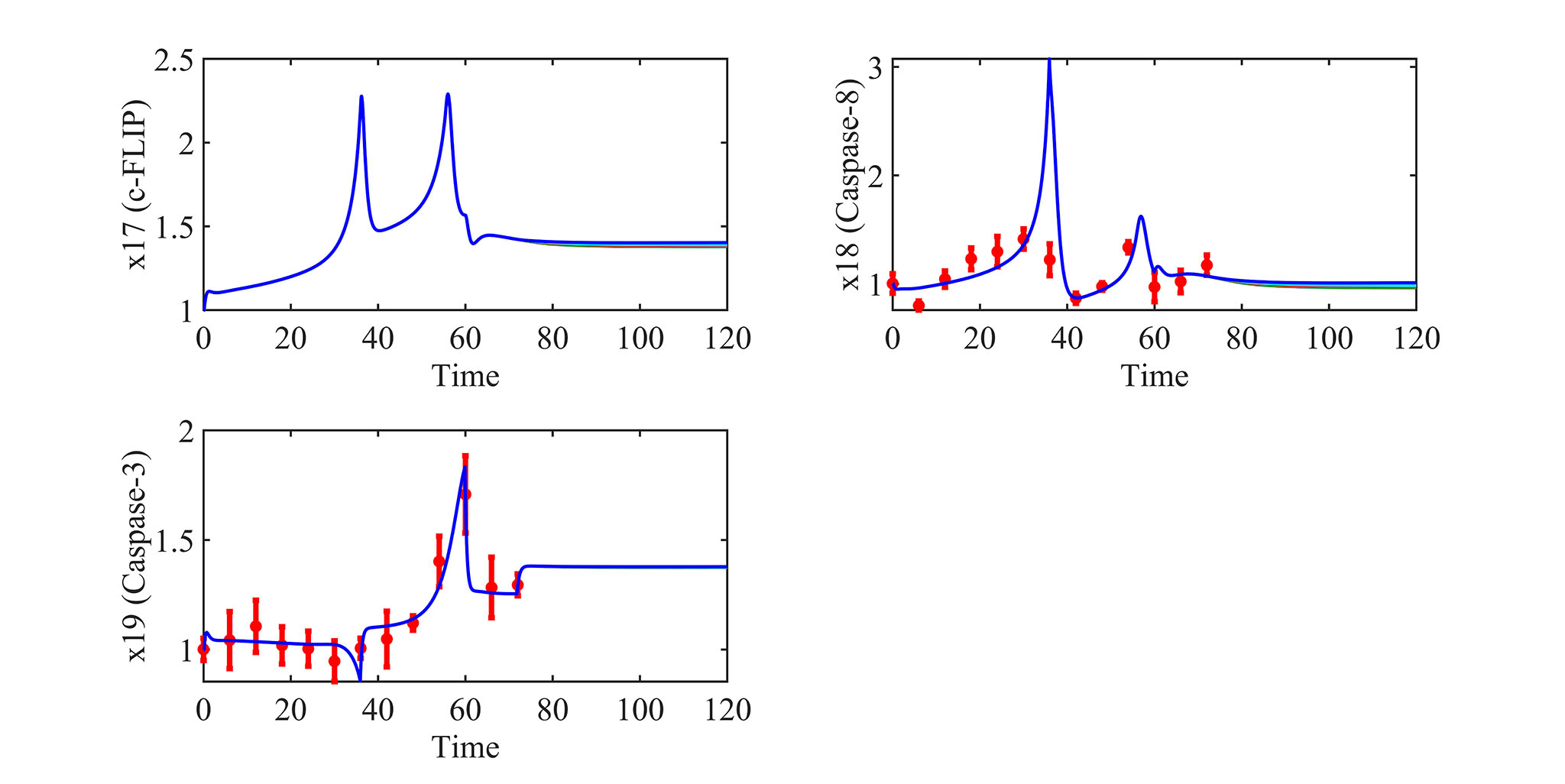

Supplement: Supplementary file 2 [file DataSheet1.zip › Supplementary material_image1/Parameter_b8(小)/5.jpg]

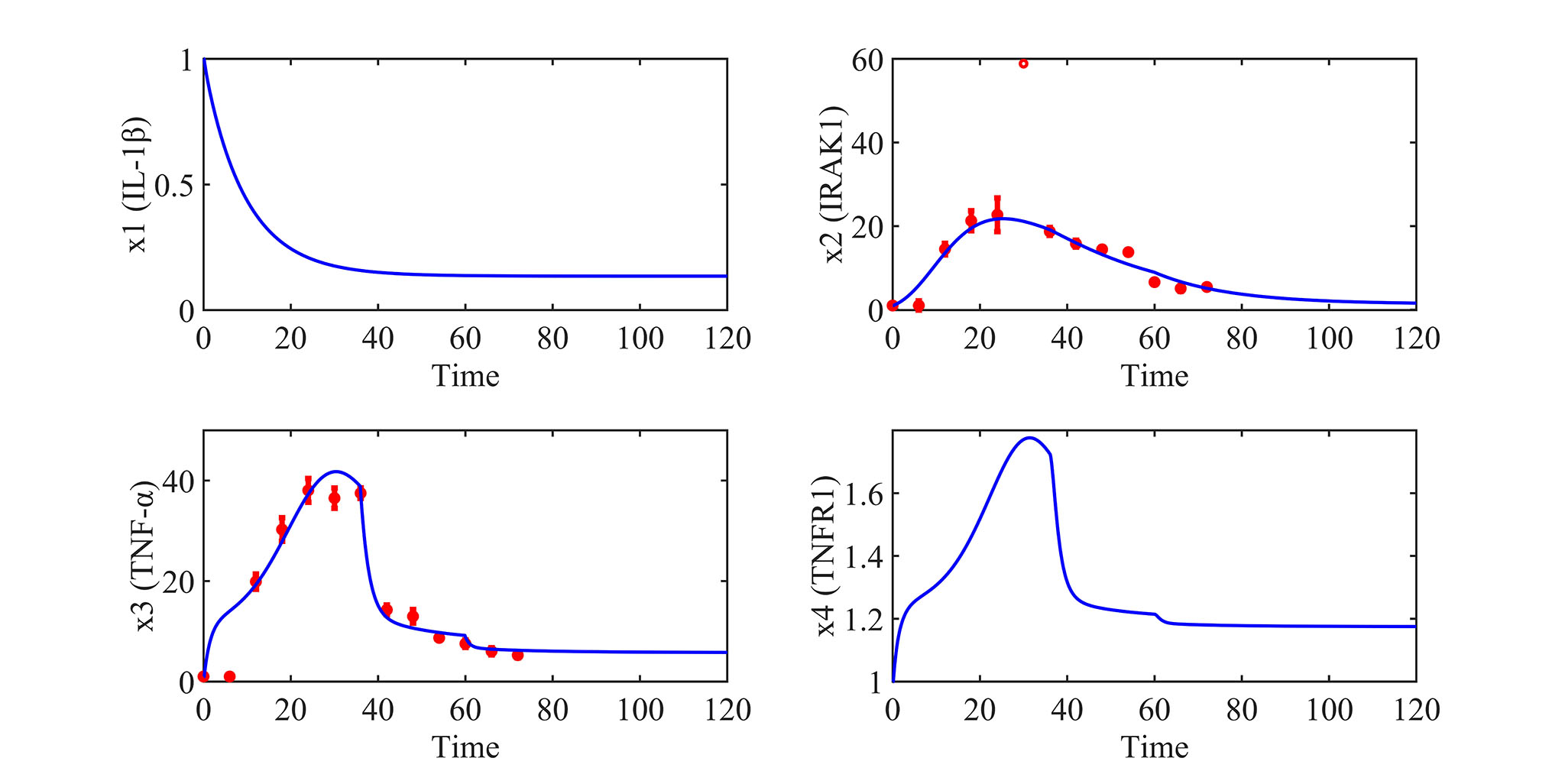

Supplement: Supplementary file 2 [file DataSheet1.zip › Supplementary material_image1/Parameter_b9(小)/1.jpg]

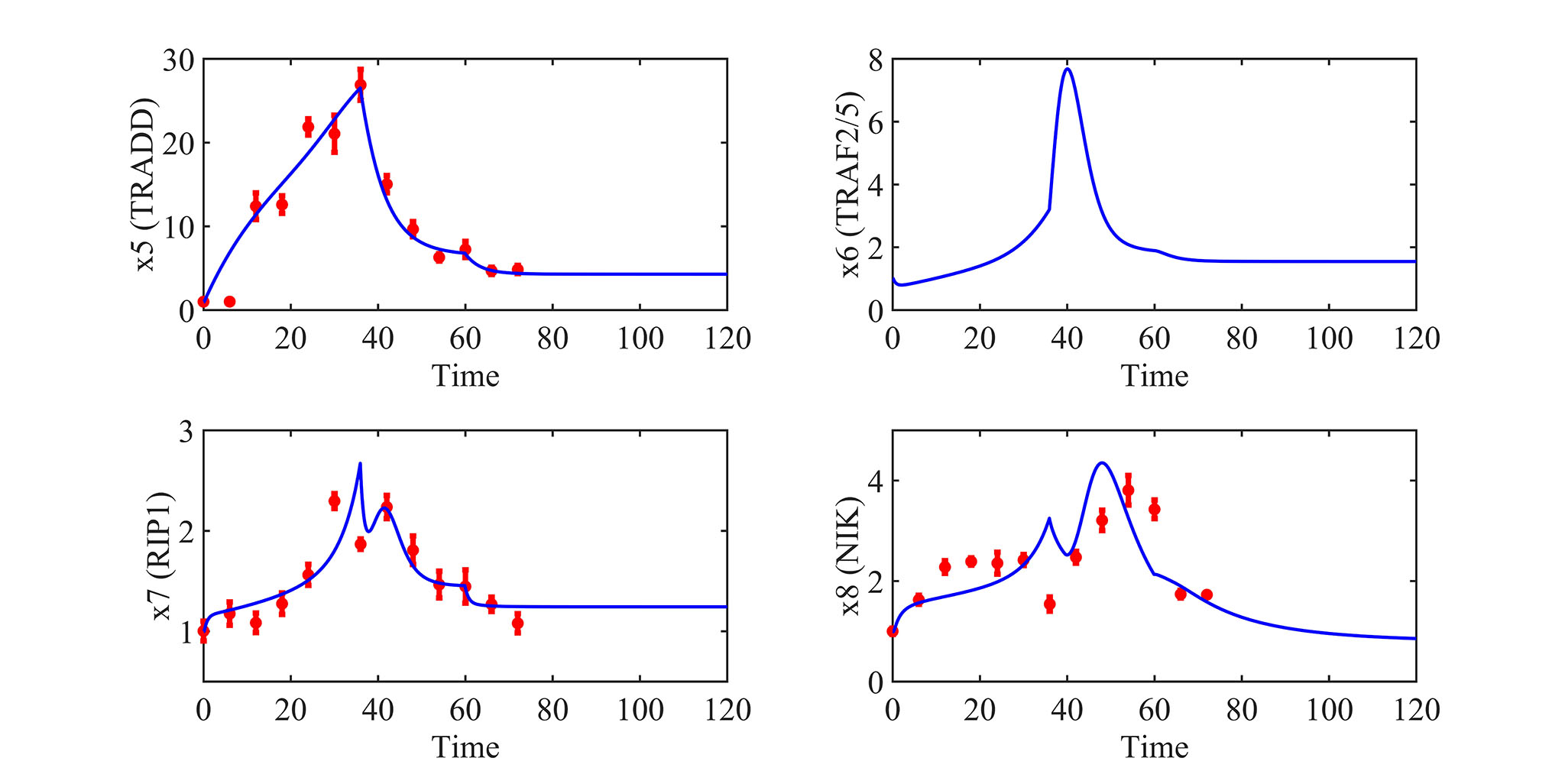

Supplement: Supplementary file 2 [file DataSheet1.zip › Supplementary material_image1/Parameter_b9(小)/2.jpg]

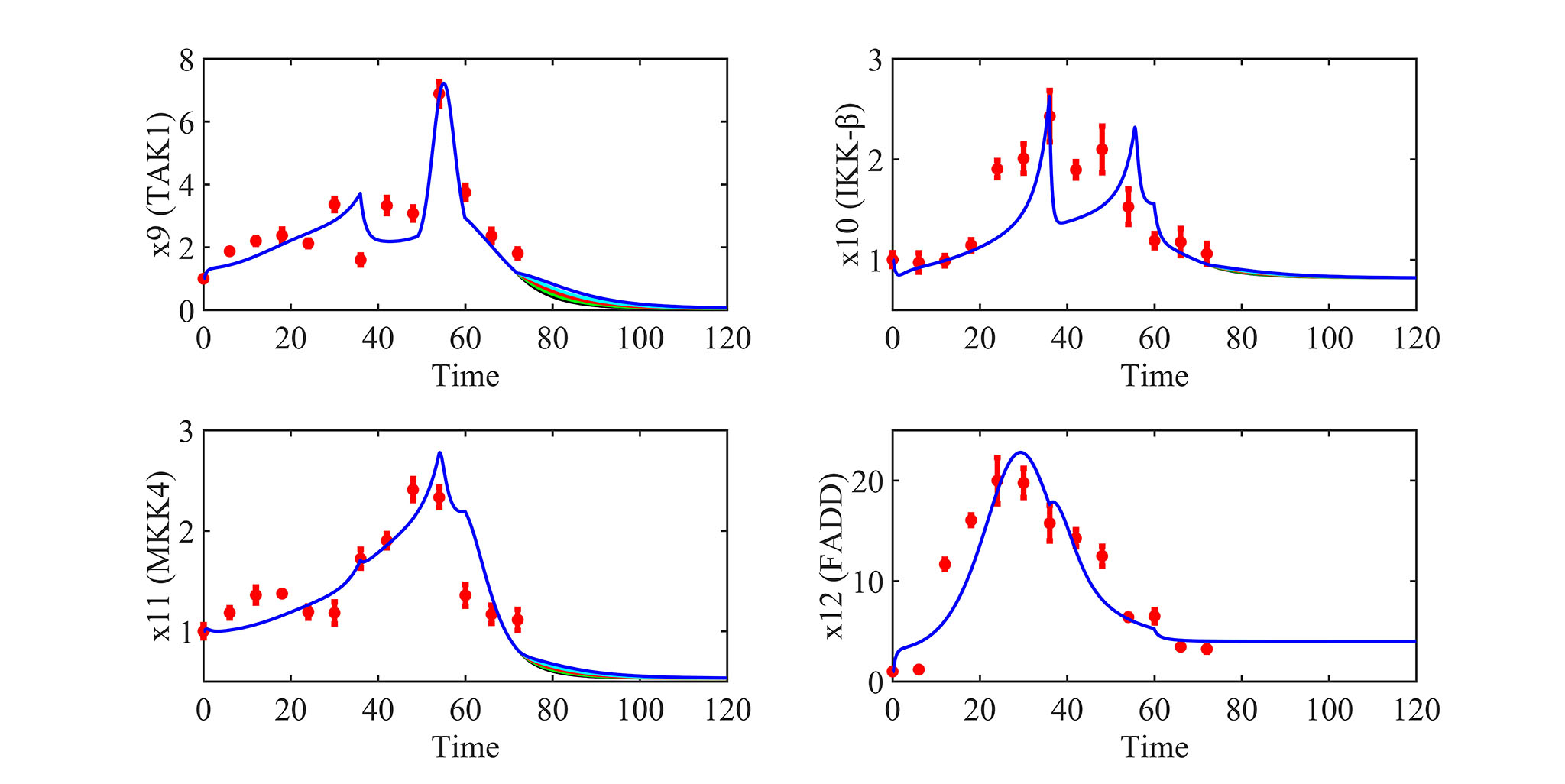

Supplement: Supplementary file 2 [file DataSheet1.zip › Supplementary material_image1/Parameter_b9(小)/3.jpg]

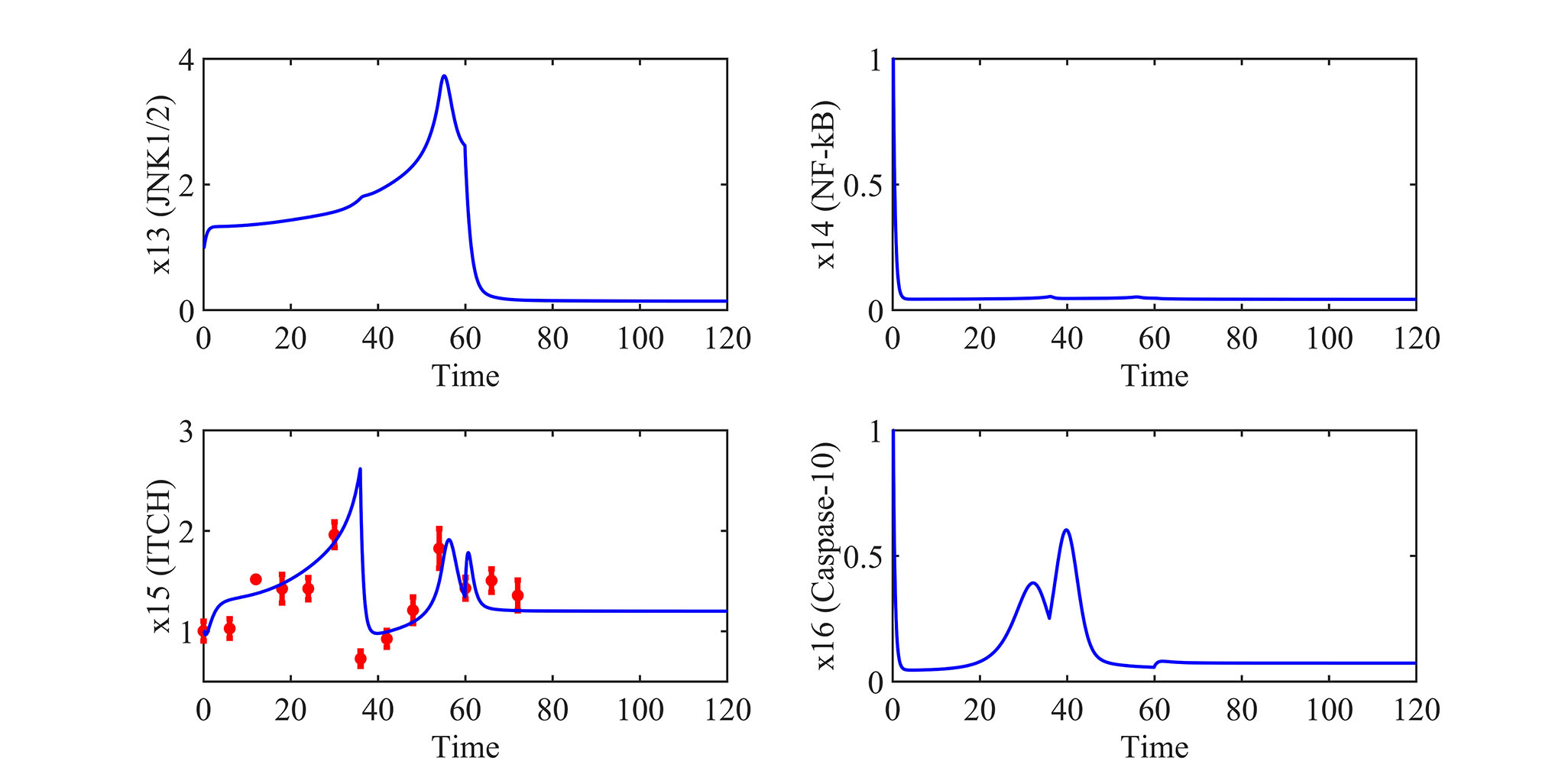

Supplement: Supplementary file 2 [file DataSheet1.zip › Supplementary material_image1/Parameter_b9(小)/4.jpg]

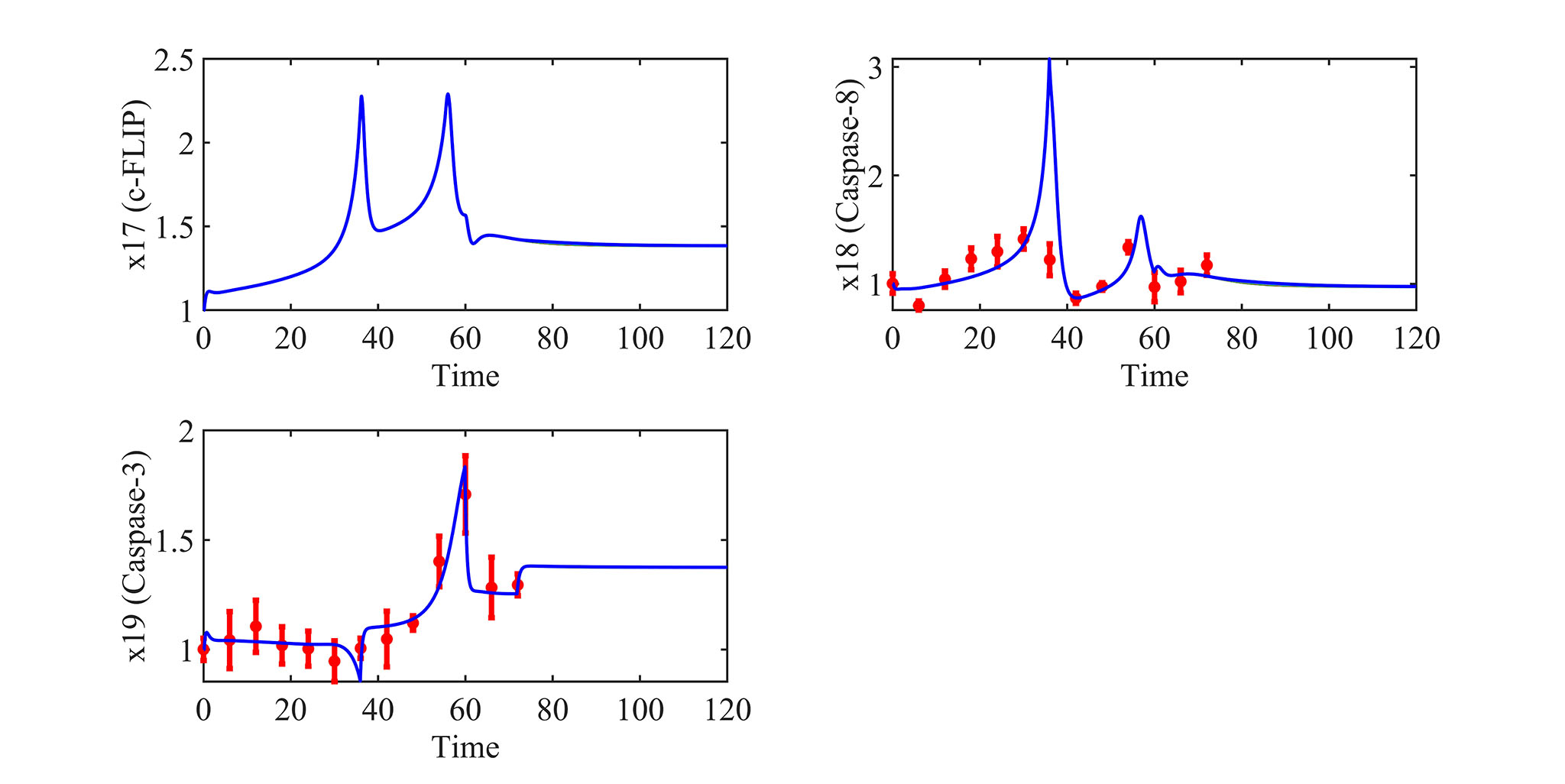

Supplement: Supplementary file 2 [file DataSheet1.zip › Supplementary material_image1/Parameter_b9(小)/5.jpg]

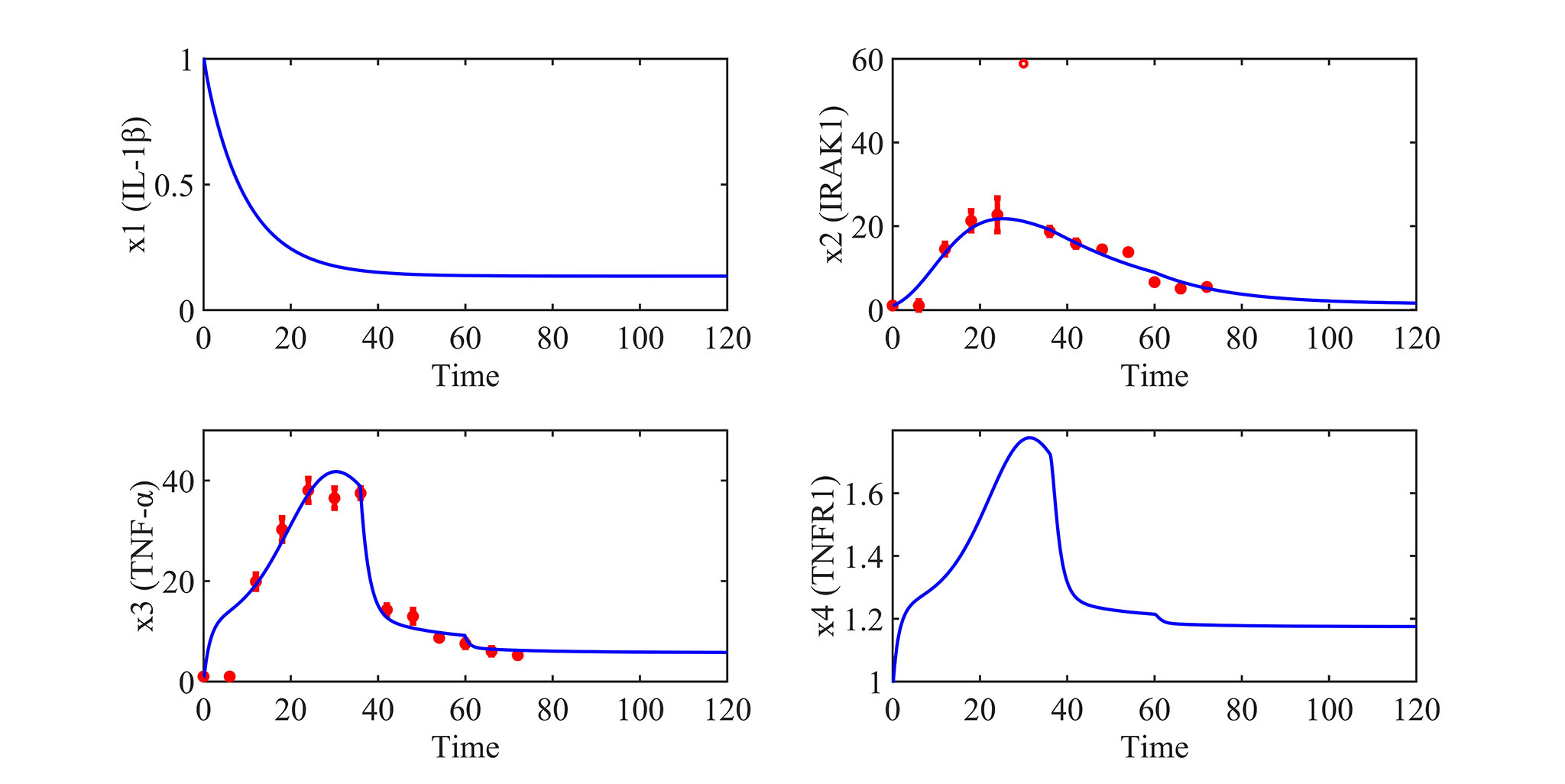

Supplement: Supplementary file 4 [file DataSheet2.zip › Supplementary material_image2/Parameter_b14(小)/1.jpg]

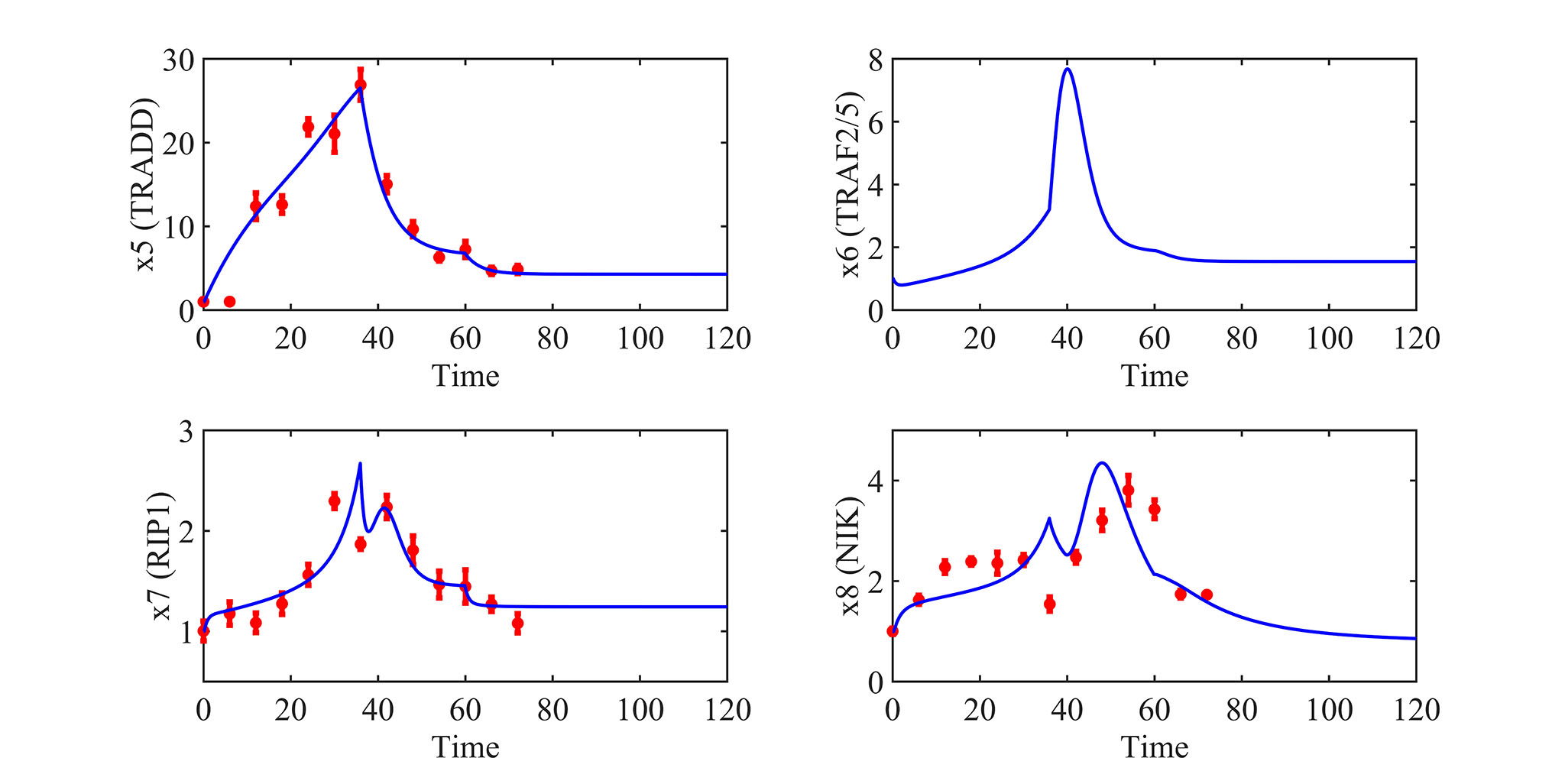

Supplement: Supplementary file 4 [file DataSheet2.zip › Supplementary material_image2/Parameter_b14(小)/2.jpg]

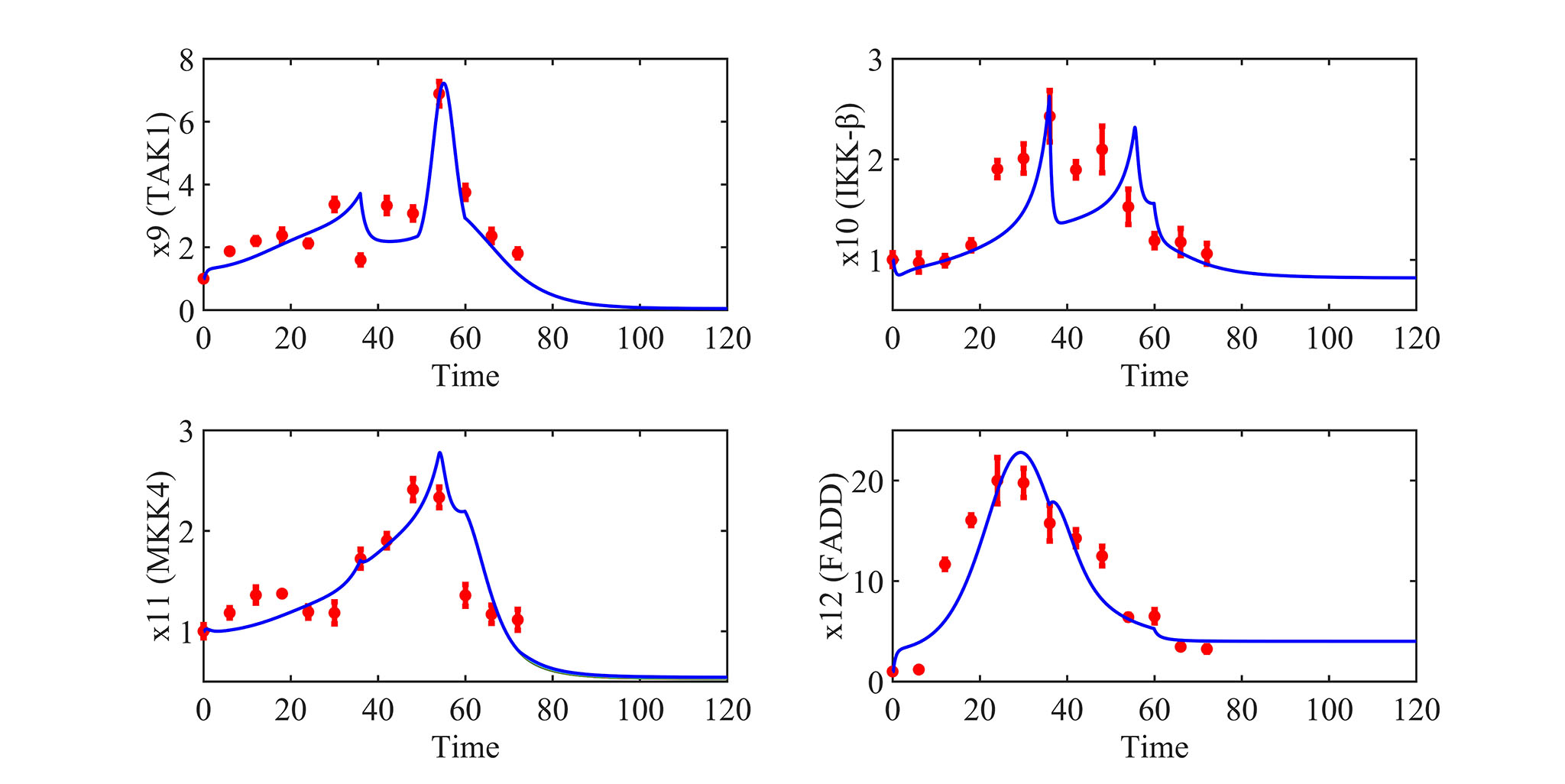

Supplement: Supplementary file 4 [file DataSheet2.zip › Supplementary material_image2/Parameter_b14(小)/3.jpg]

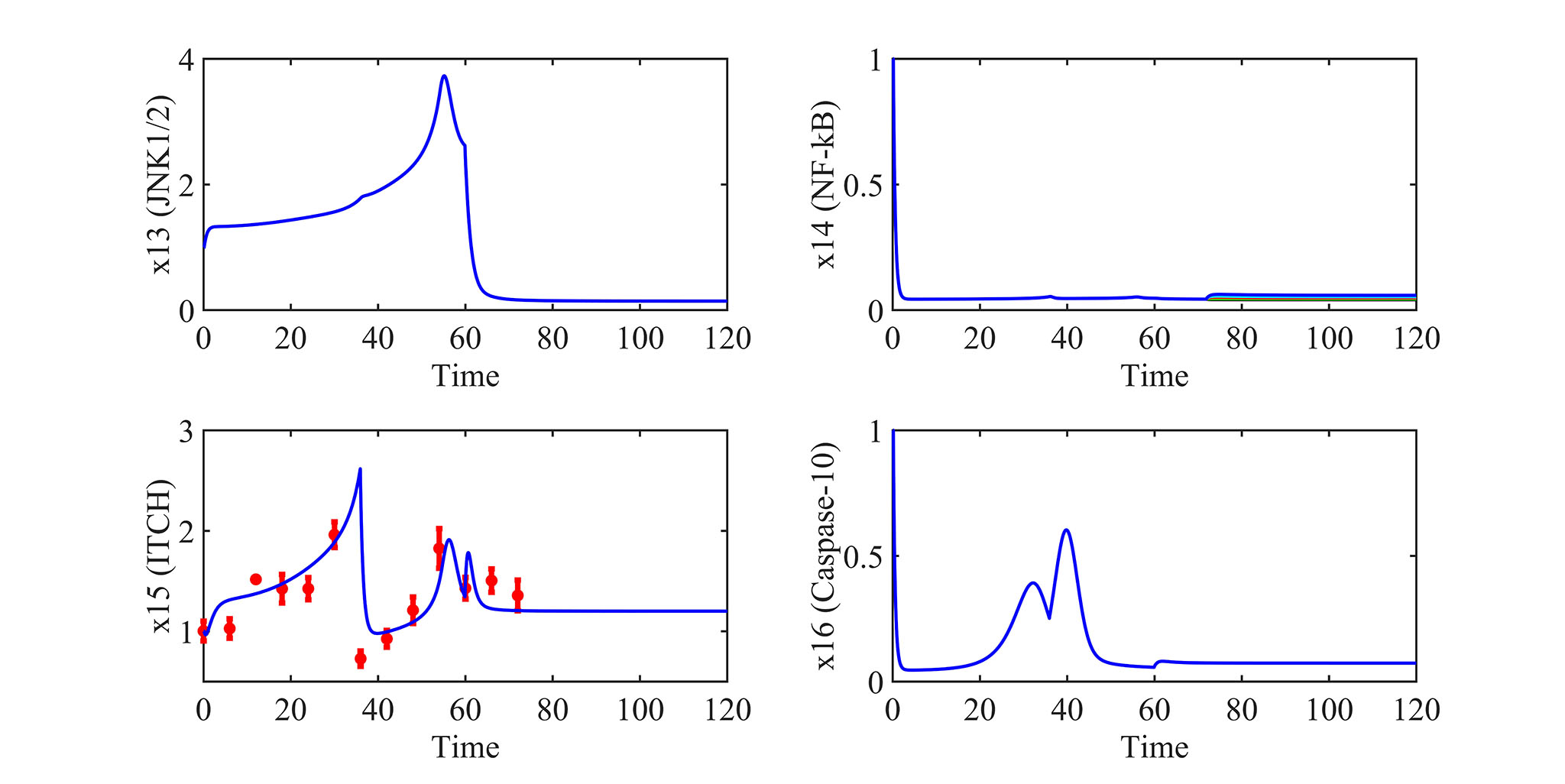

Supplement: Supplementary file 4 [file DataSheet2.zip › Supplementary material_image2/Parameter_b14(小)/4.jpg]

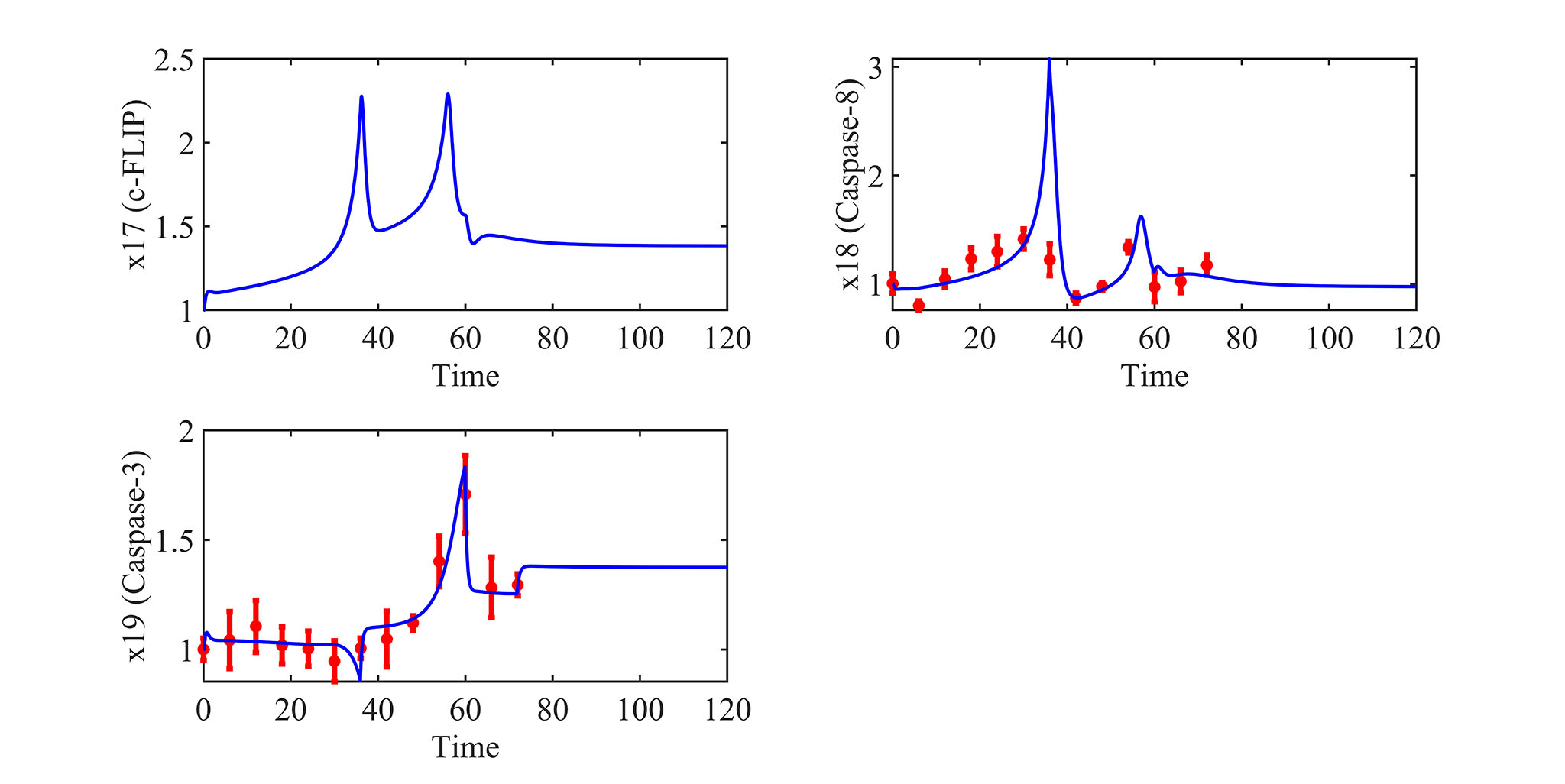

Supplement: Supplementary file 4 [file DataSheet2.zip › Supplementary material_image2/Parameter_b14(小)/5.jpg]

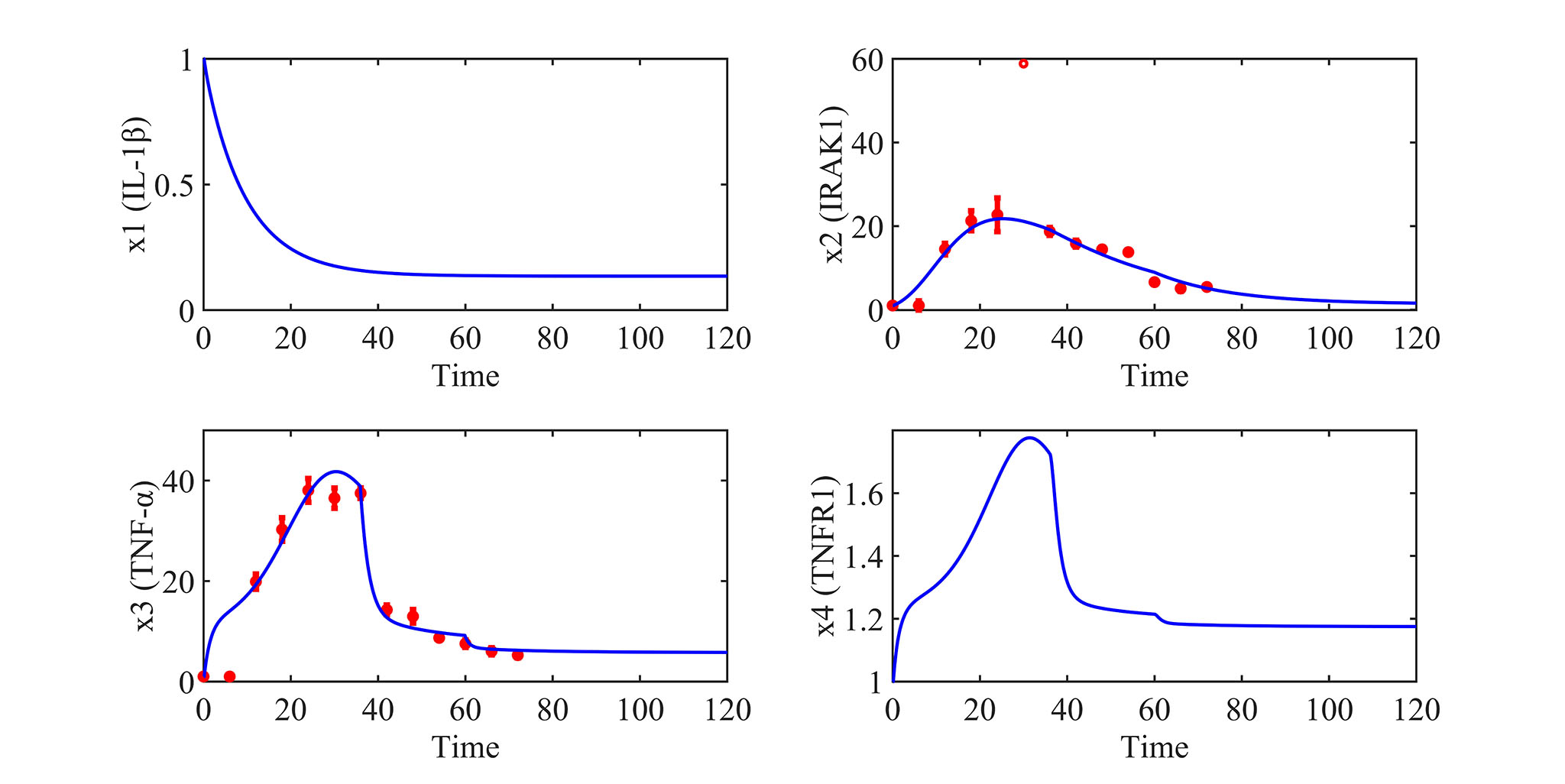

Supplement: Supplementary file 4 [file DataSheet2.zip › Supplementary material_image2/Parameter_b15(小)/1.jpg]

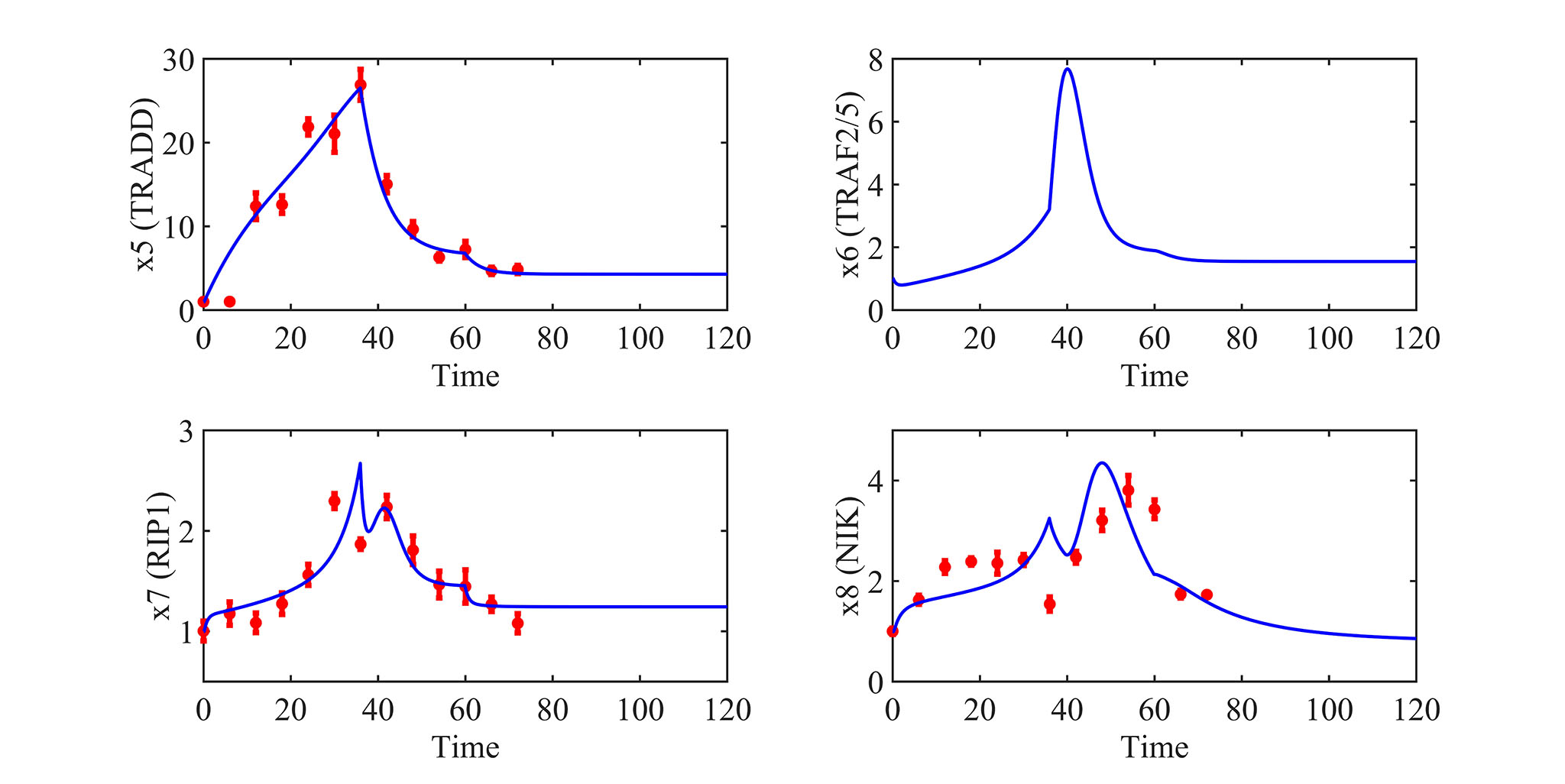

Supplement: Supplementary file 4 [file DataSheet2.zip › Supplementary material_image2/Parameter_b15(小)/2.jpg]

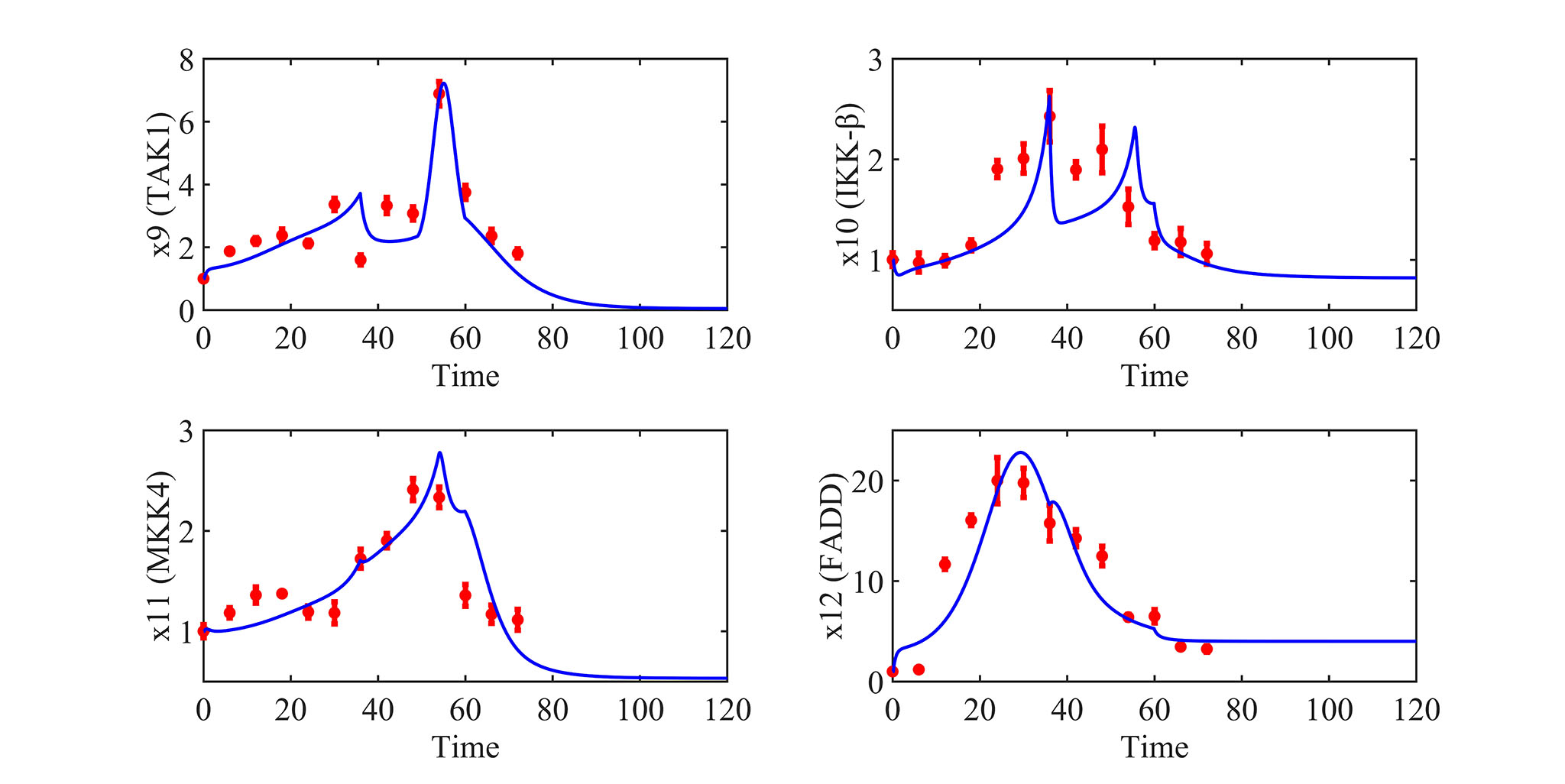

Supplement: Supplementary file 4 [file DataSheet2.zip › Supplementary material_image2/Parameter_b15(小)/3.jpg]

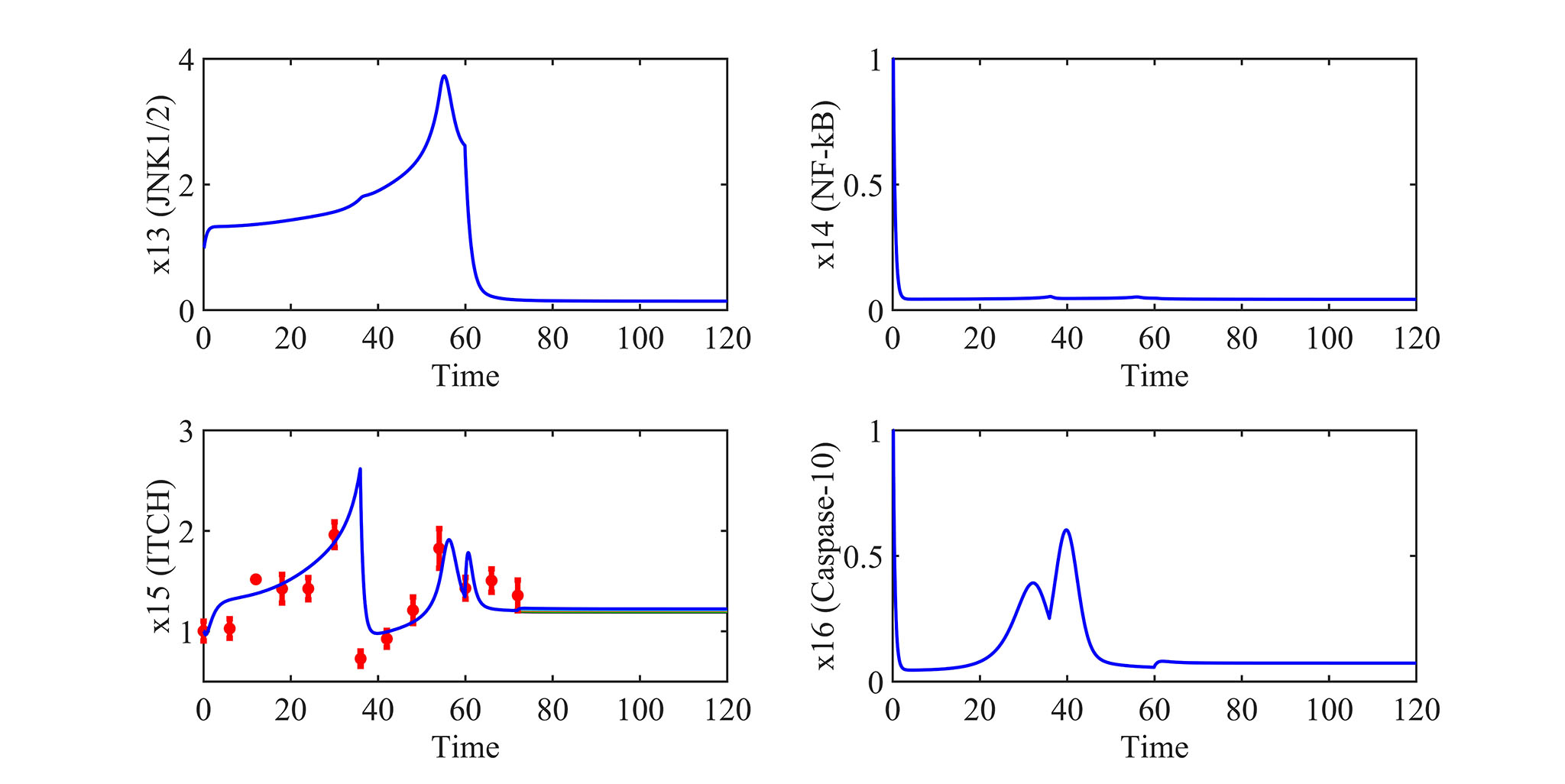

Supplement: Supplementary file 4 [file DataSheet2.zip › Supplementary material_image2/Parameter_b15(小)/4.jpg]

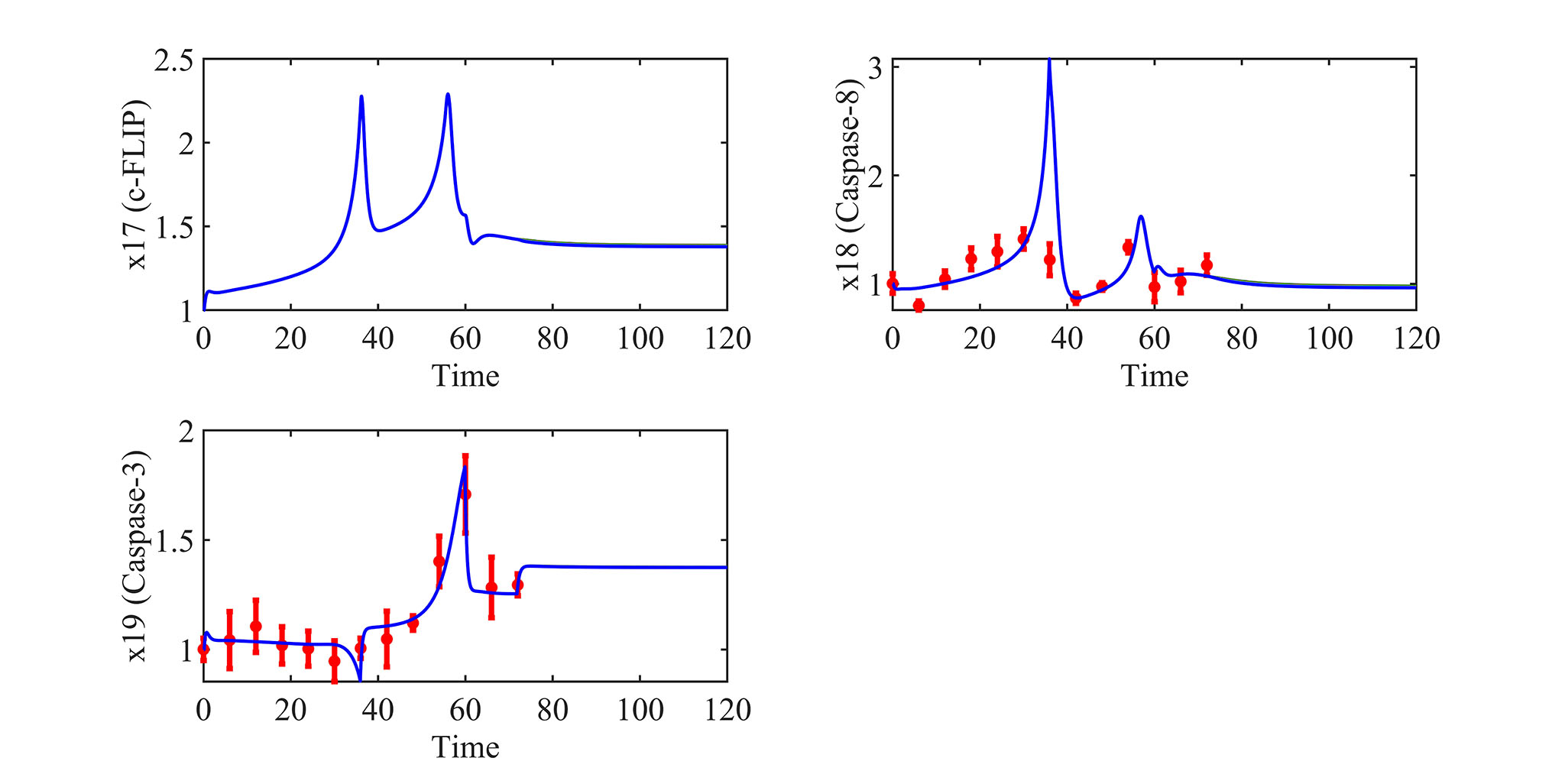

Supplement: Supplementary file 4 [file DataSheet2.zip › Supplementary material_image2/Parameter_b15(小)/5.jpg]

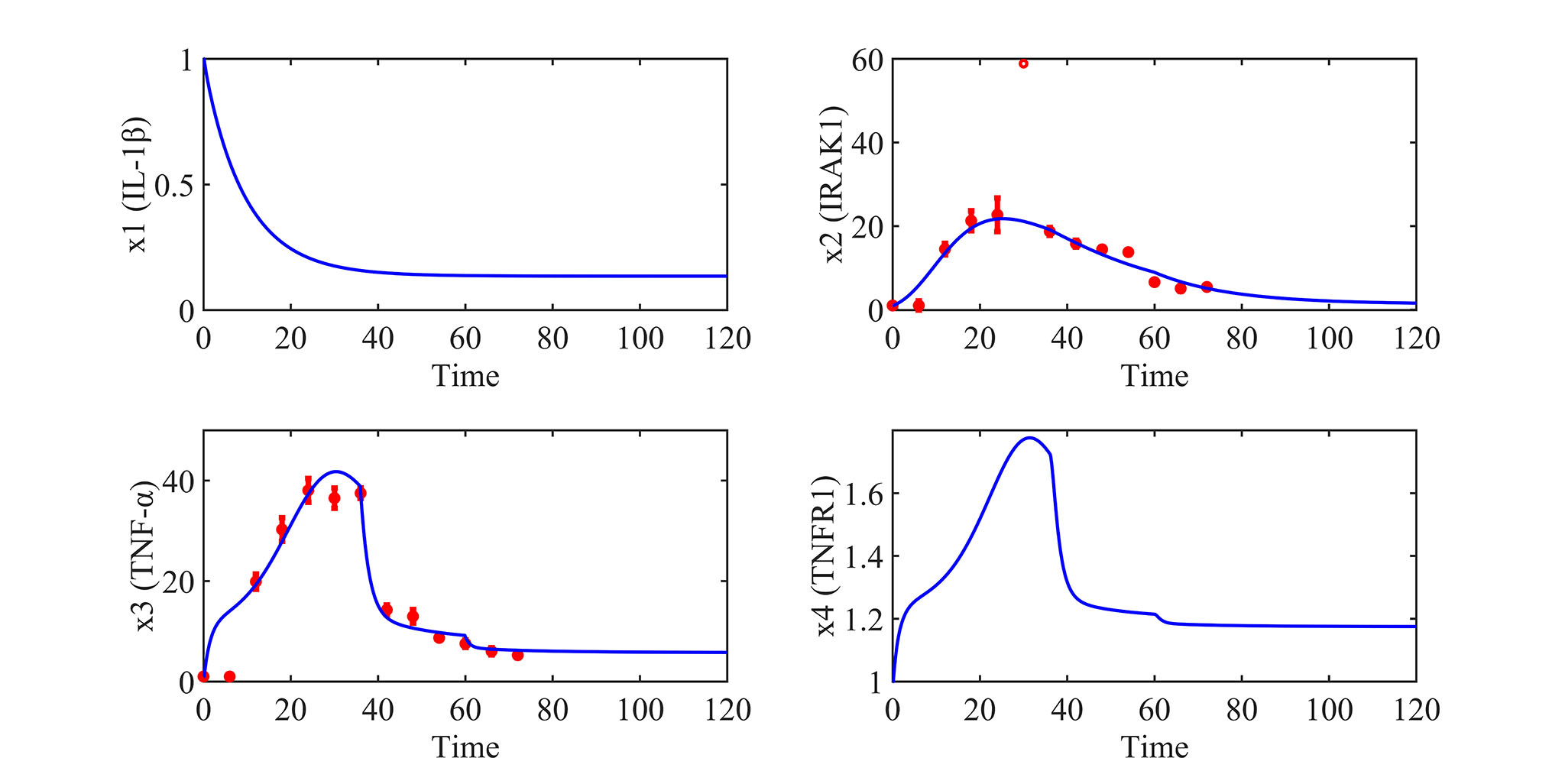

Supplement: Supplementary file 4 [file DataSheet2.zip › Supplementary material_image2/Parameter_b16(大)/1.jpg]

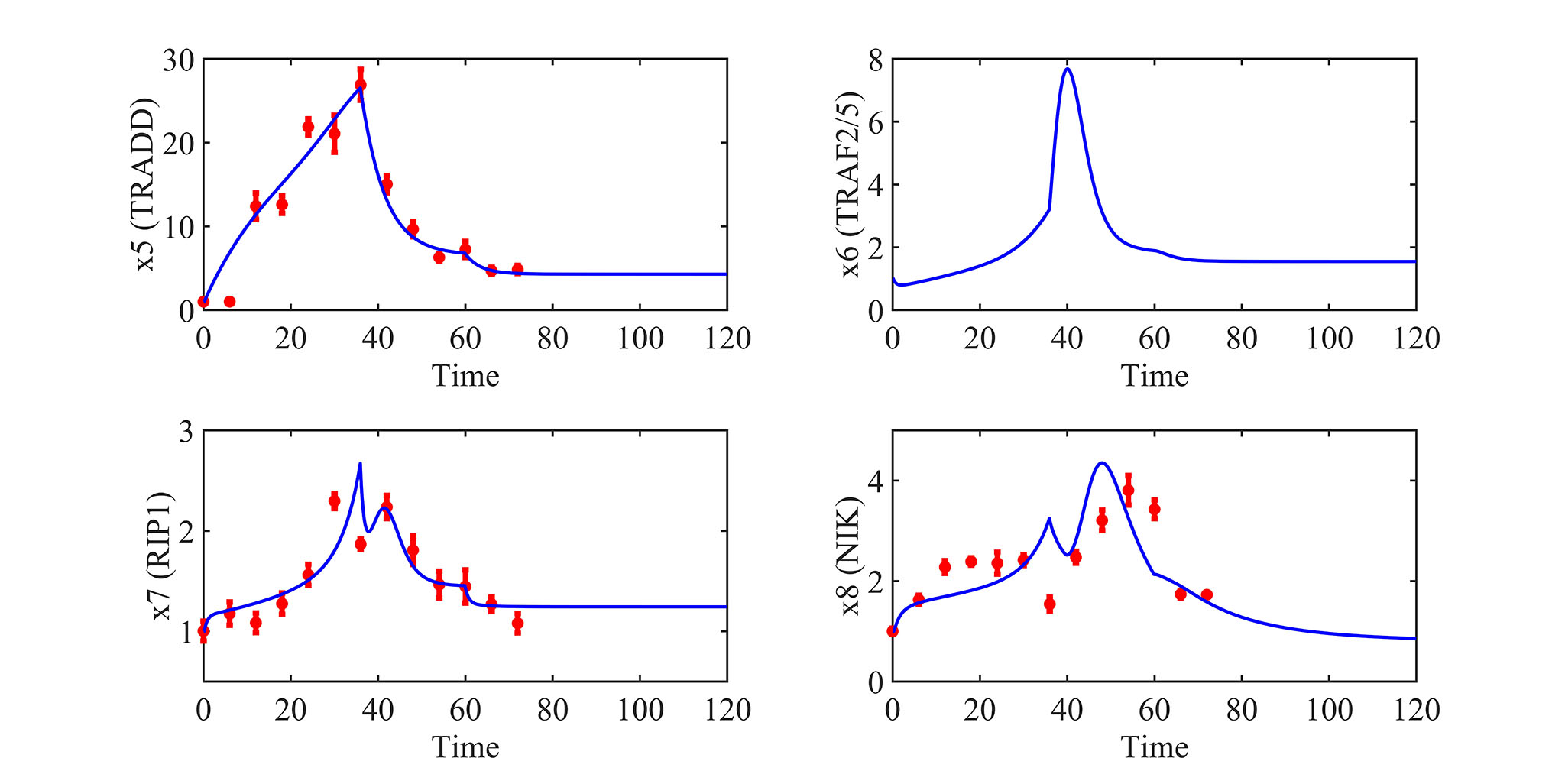

Supplement: Supplementary file 4 [file DataSheet2.zip › Supplementary material_image2/Parameter_b16(大)/2.jpg]

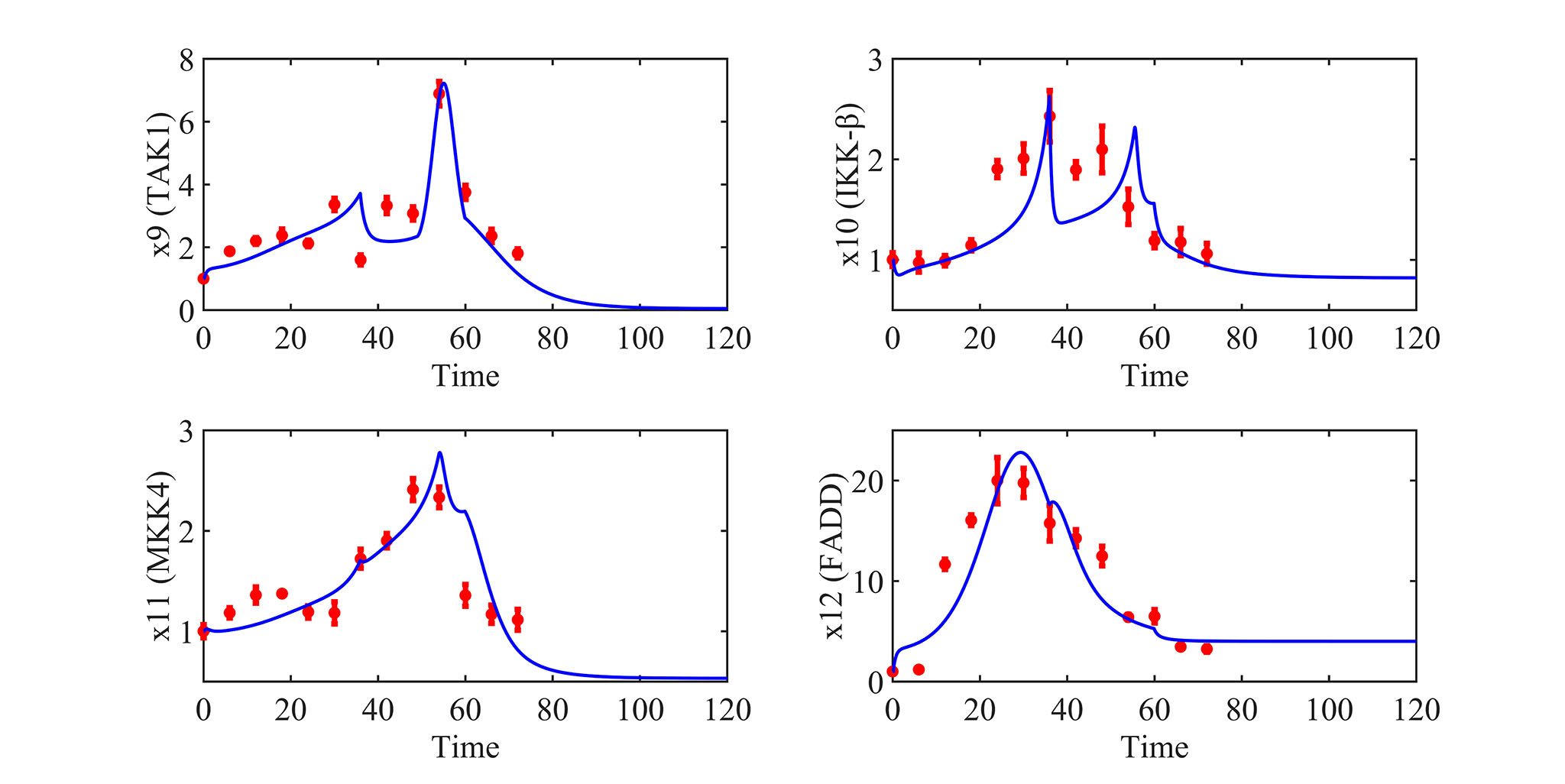

Supplement: Supplementary file 4 [file DataSheet2.zip › Supplementary material_image2/Parameter_b16(大)/3.jpg]

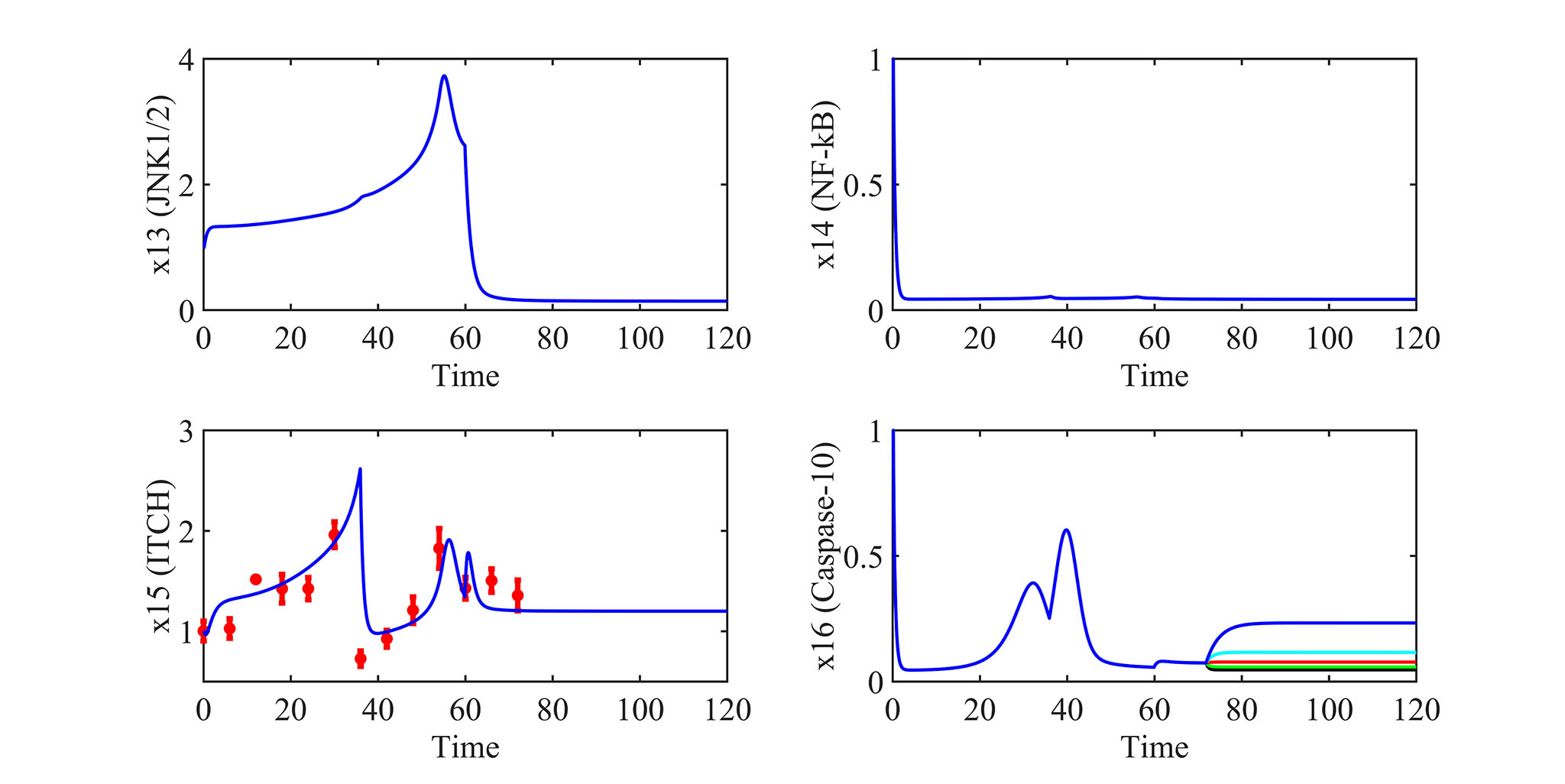

Supplement: Supplementary file 4 [file DataSheet2.zip › Supplementary material_image2/Parameter_b16(大)/4.jpg]

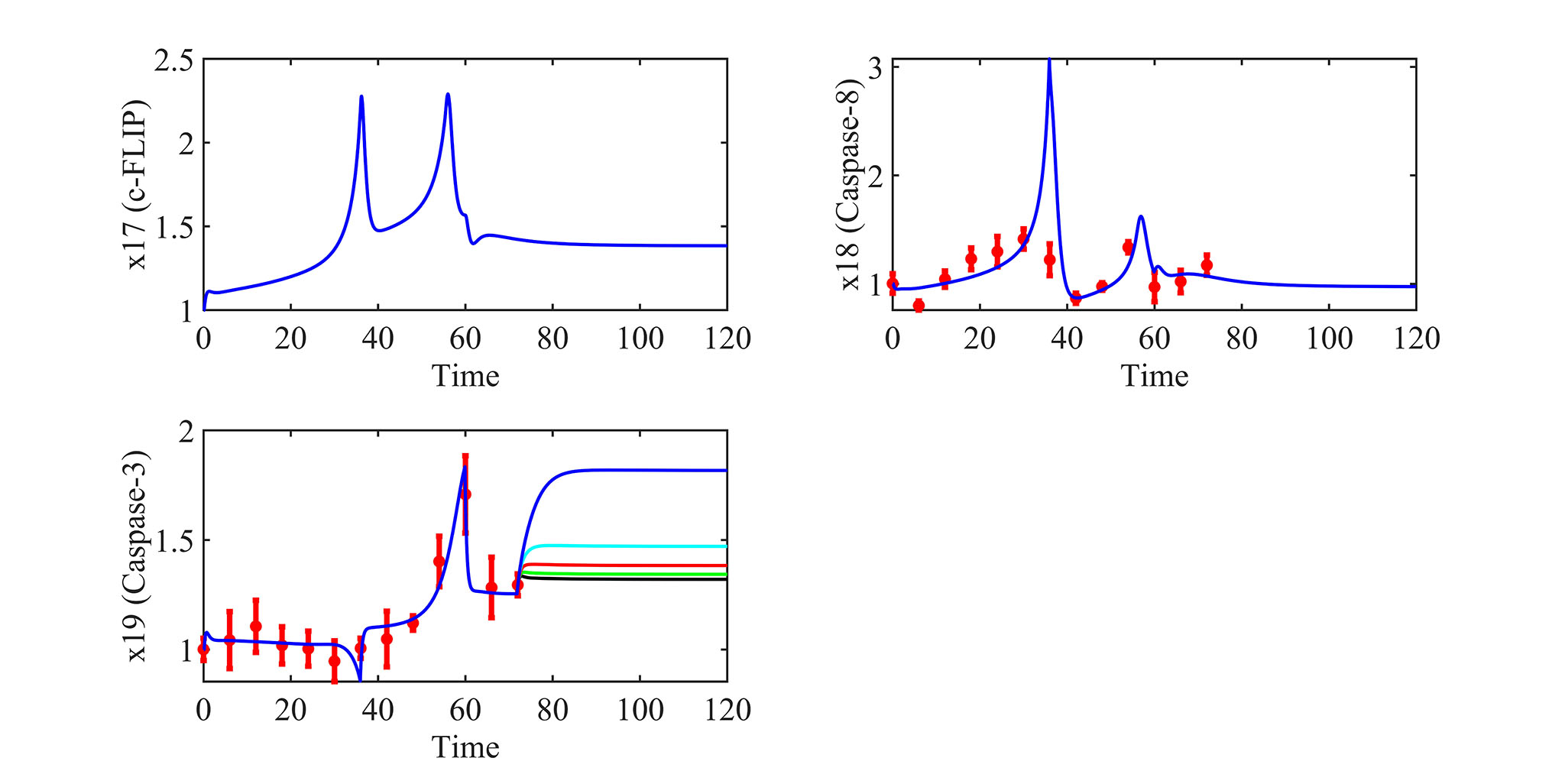

Supplement: Supplementary file 4 [file DataSheet2.zip › Supplementary material_image2/Parameter_b16(大)/5.jpg]

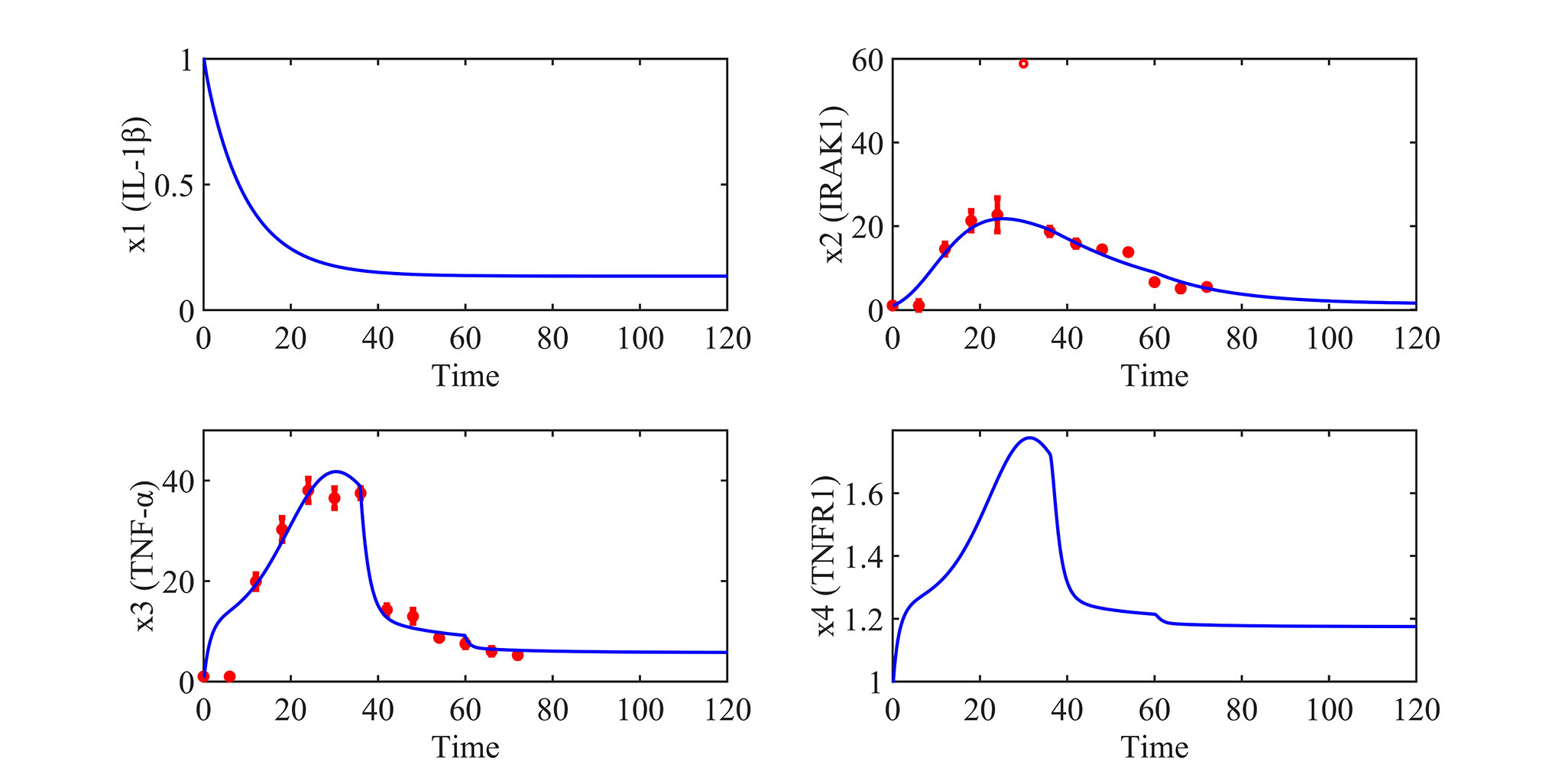

Supplement: Supplementary file 4 [file DataSheet2.zip › Supplementary material_image2/Parameter_b18(大)/1.jpg]

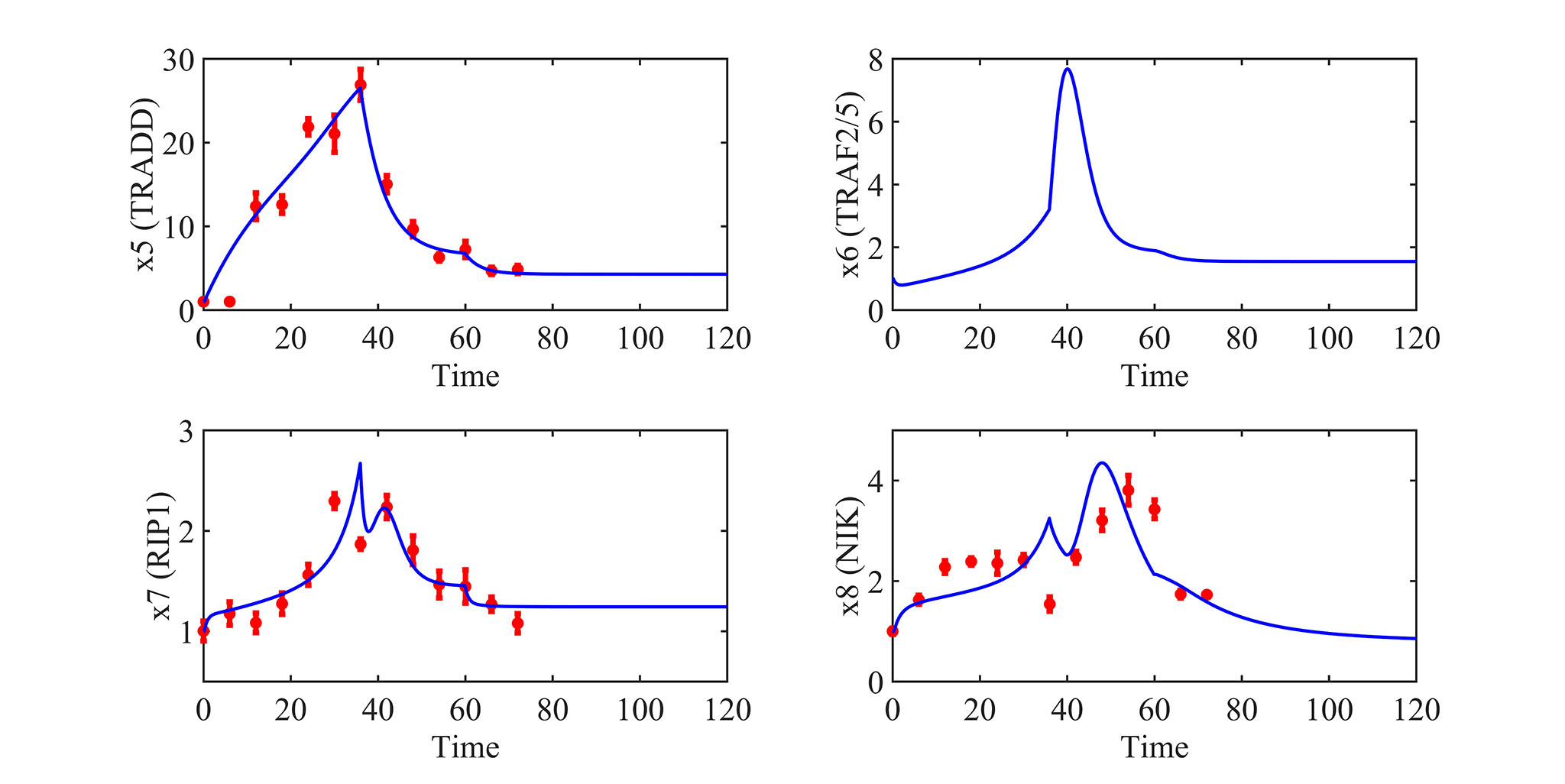

Supplement: Supplementary file 4 [file DataSheet2.zip › Supplementary material_image2/Parameter_b18(大)/2.jpg]

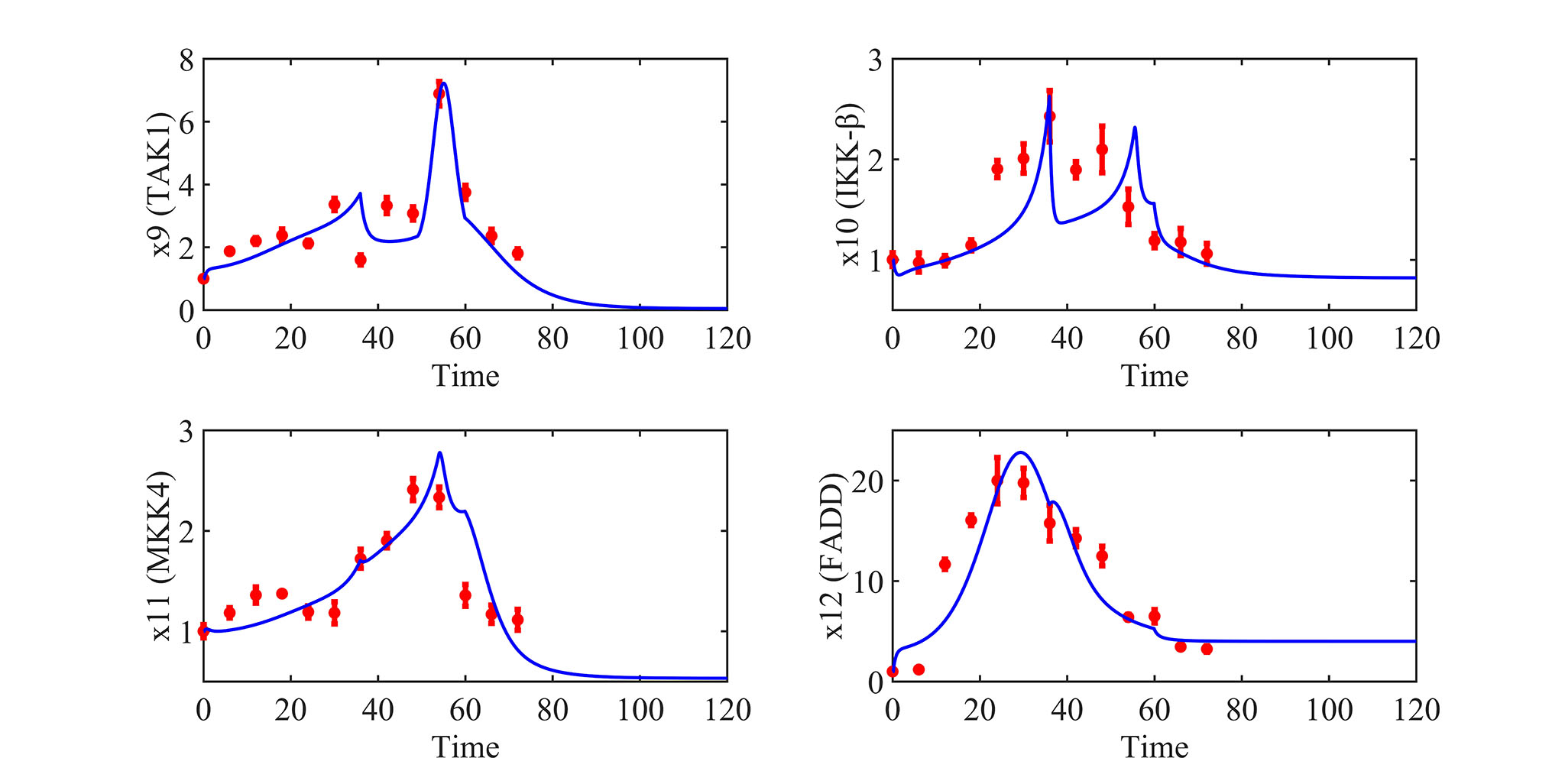

Supplement: Supplementary file 4 [file DataSheet2.zip › Supplementary material_image2/Parameter_b18(大)/3.jpg]

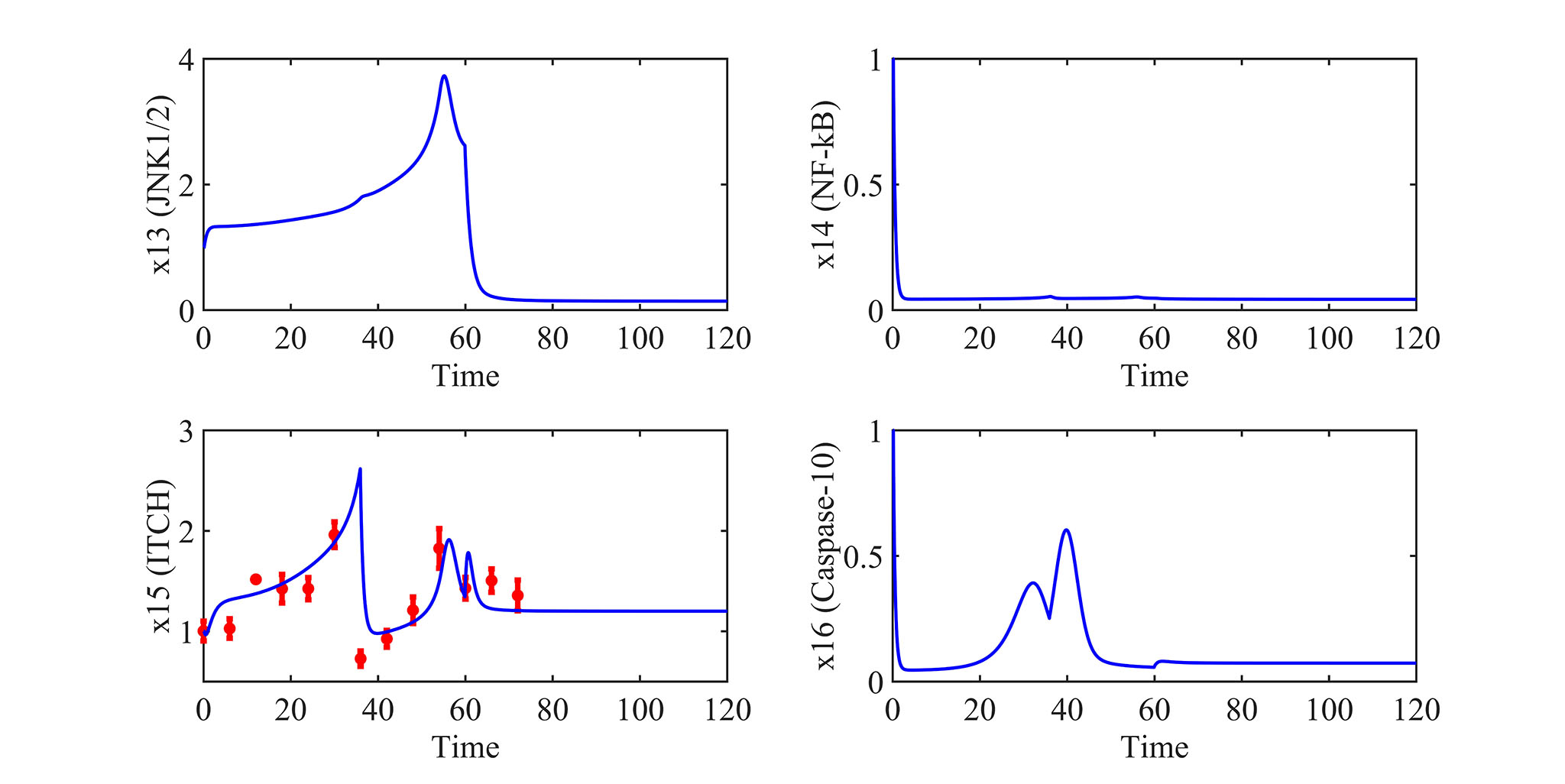

Supplement: Supplementary file 4 [file DataSheet2.zip › Supplementary material_image2/Parameter_b18(大)/4.jpg]

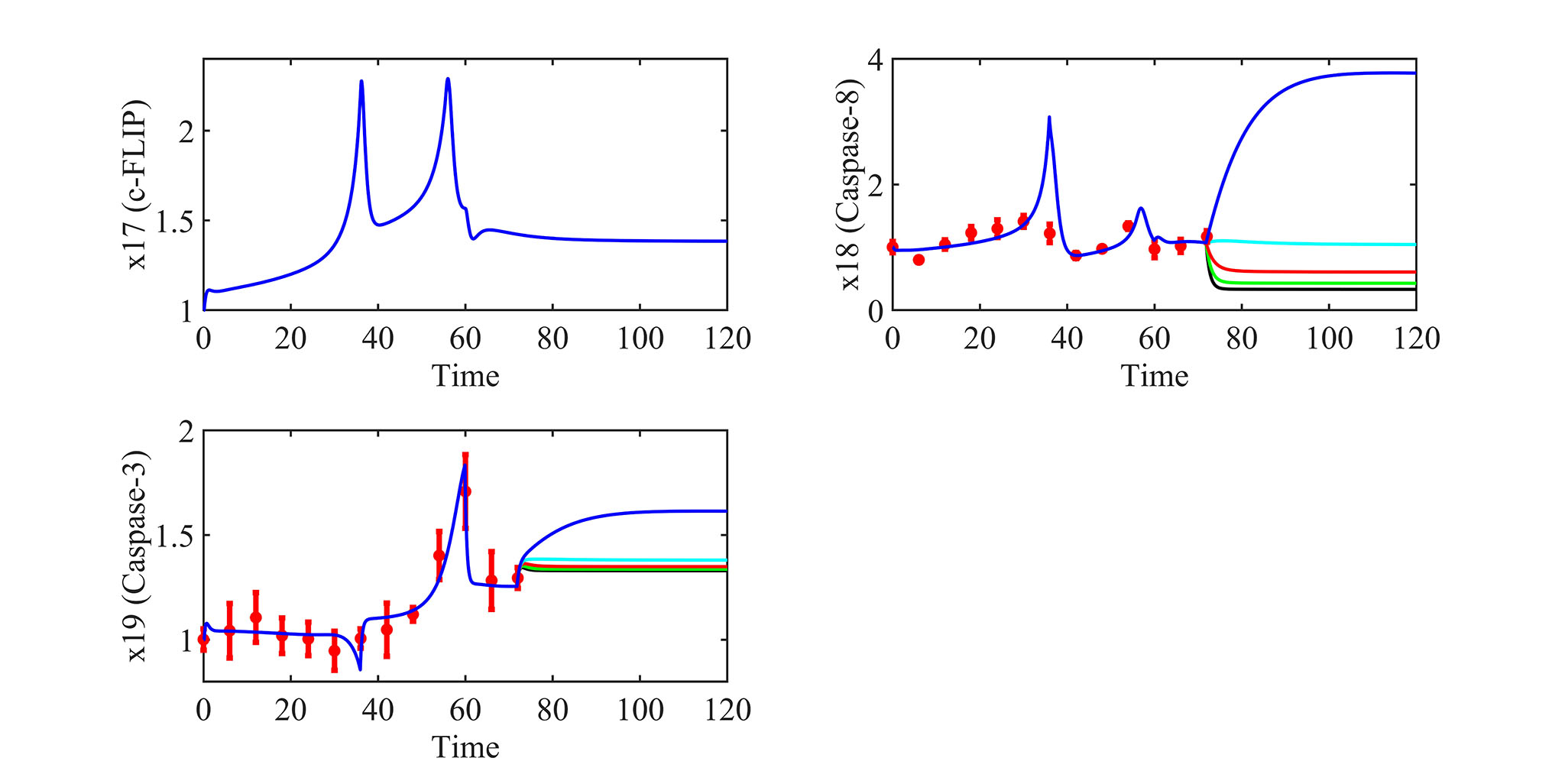

Supplement: Supplementary file 4 [file DataSheet2.zip › Supplementary material_image2/Parameter_b18(大)/5.jpg]

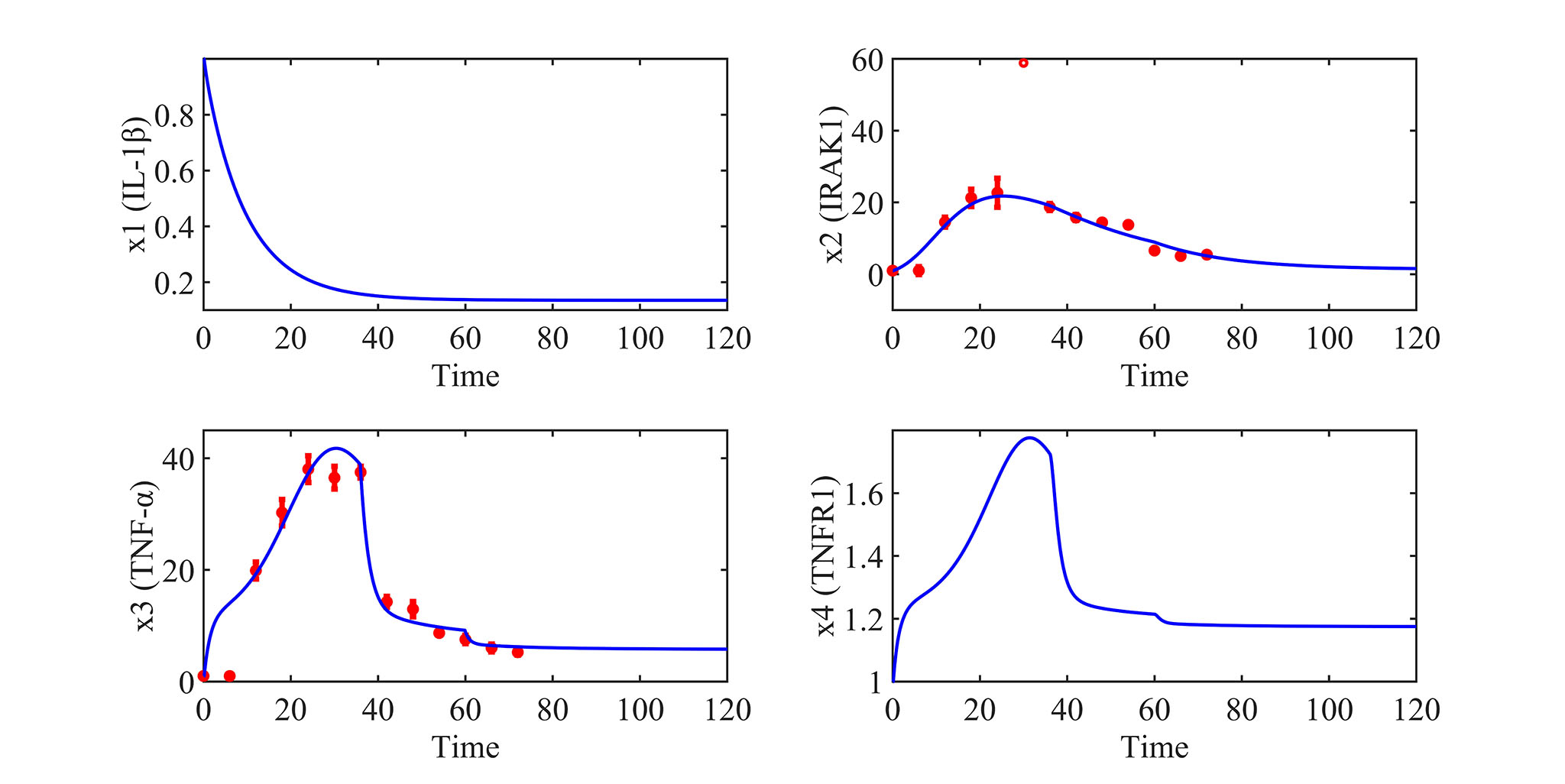

Supplement: Supplementary file 4 [file DataSheet2.zip › Supplementary material_image2/Parameter_b19(大)/1.jpg]

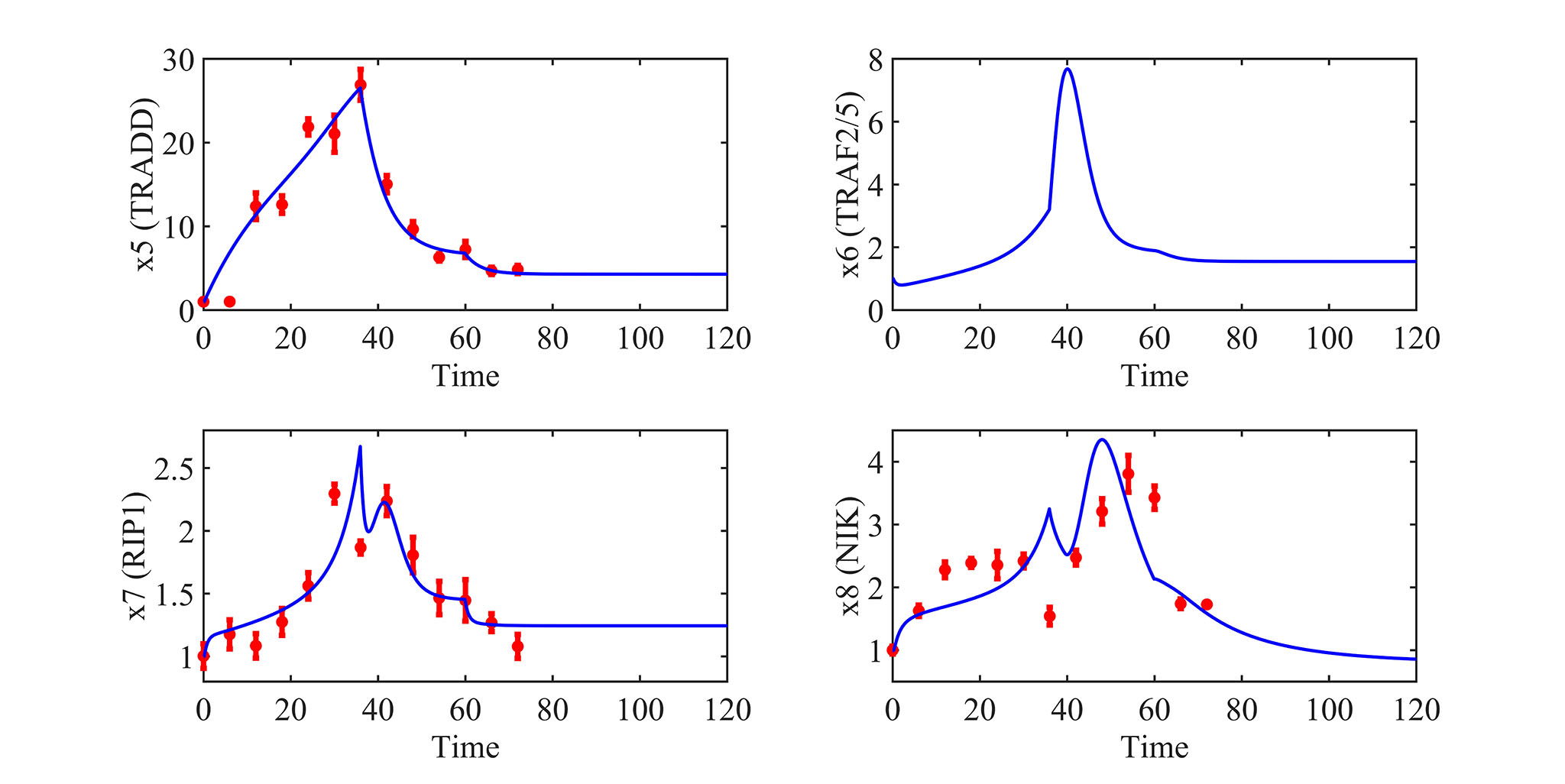

Supplement: Supplementary file 4 [file DataSheet2.zip › Supplementary material_image2/Parameter_b19(大)/2.jpg]

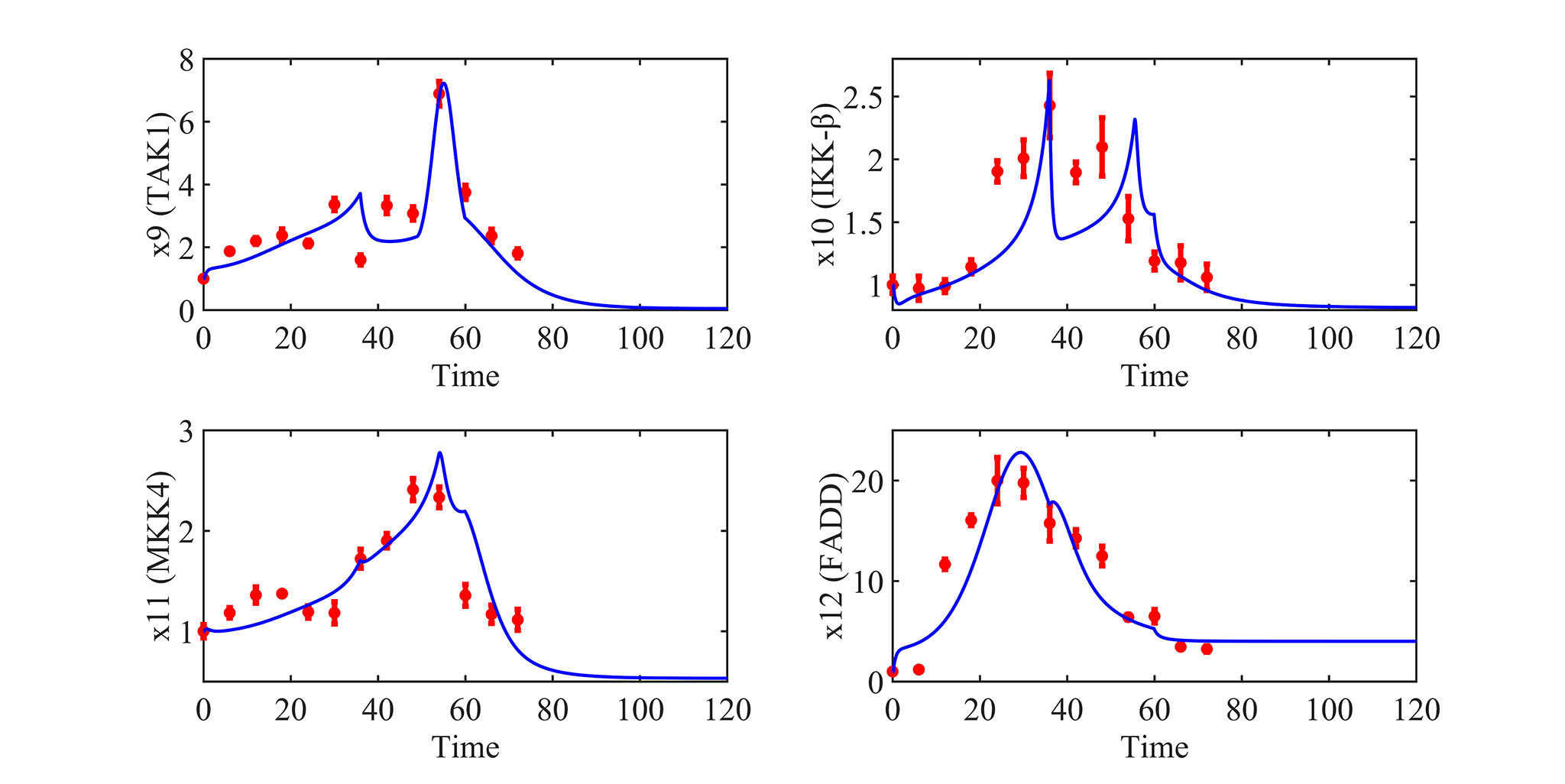

Supplement: Supplementary file 4 [file DataSheet2.zip › Supplementary material_image2/Parameter_b19(大)/3.jpg]

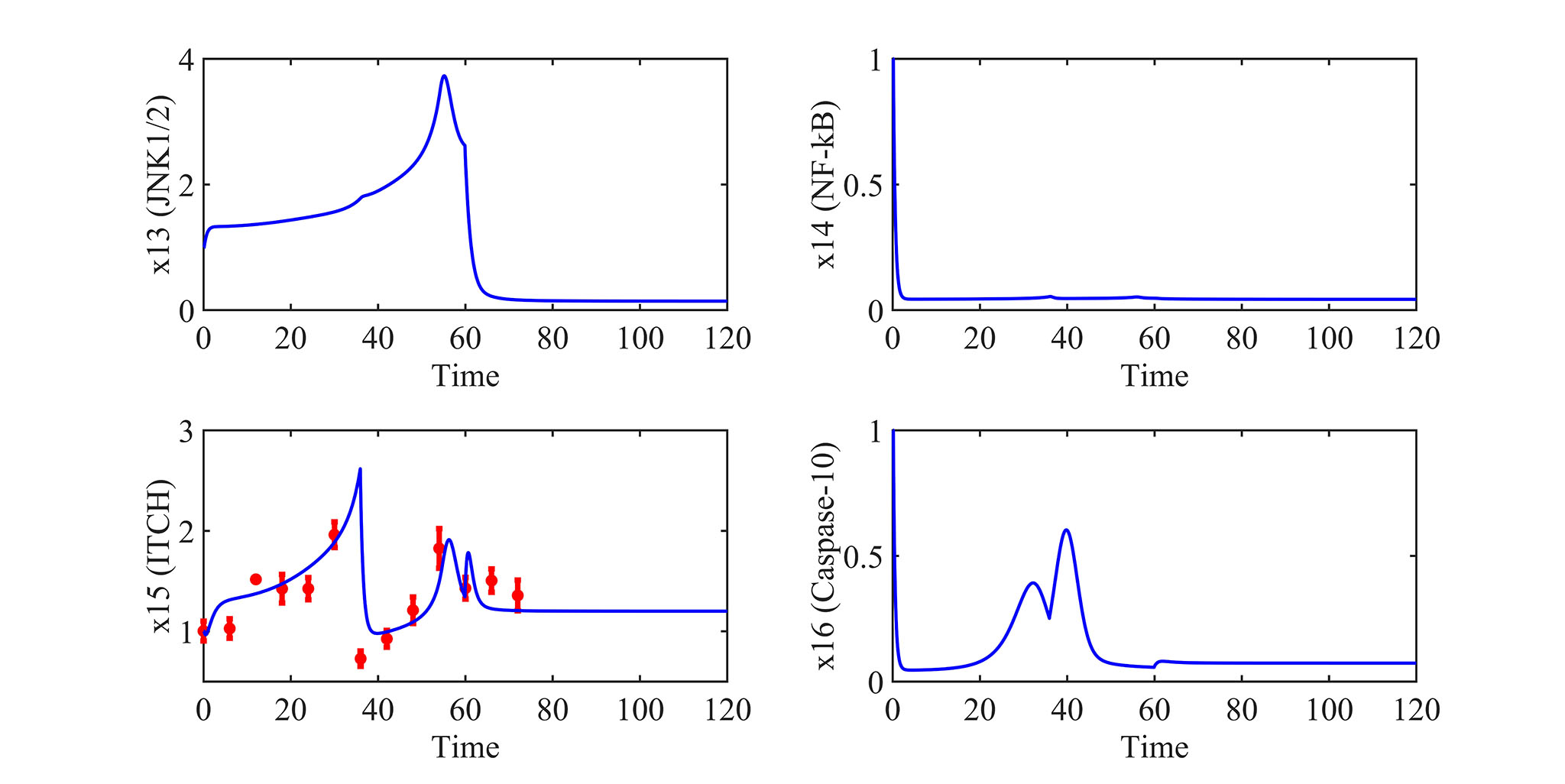

Supplement: Supplementary file 4 [file DataSheet2.zip › Supplementary material_image2/Parameter_b19(大)/4.jpg]

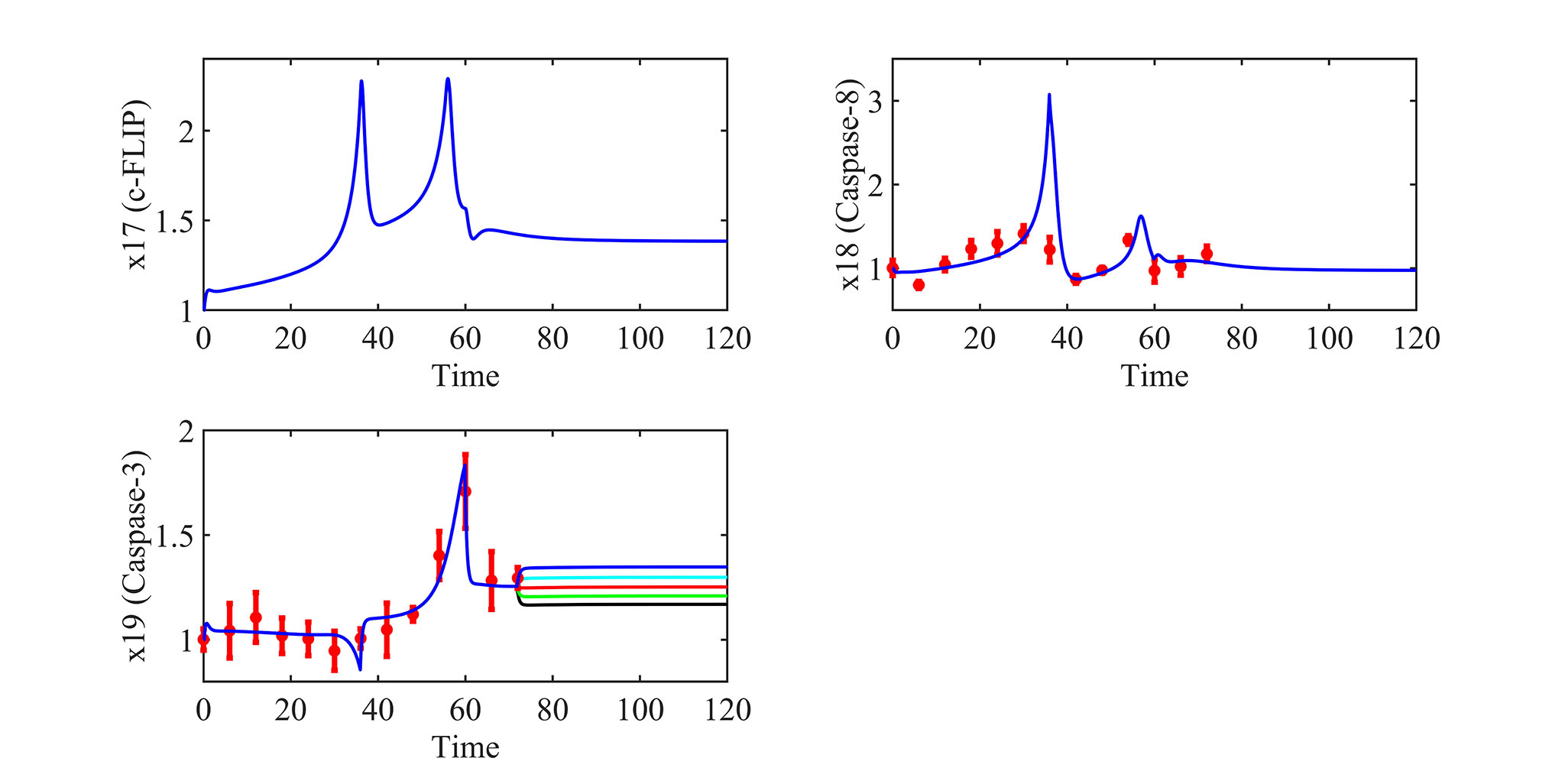

Supplement: Supplementary file 4 [file DataSheet2.zip › Supplementary material_image2/Parameter_b19(大)/5.jpg]

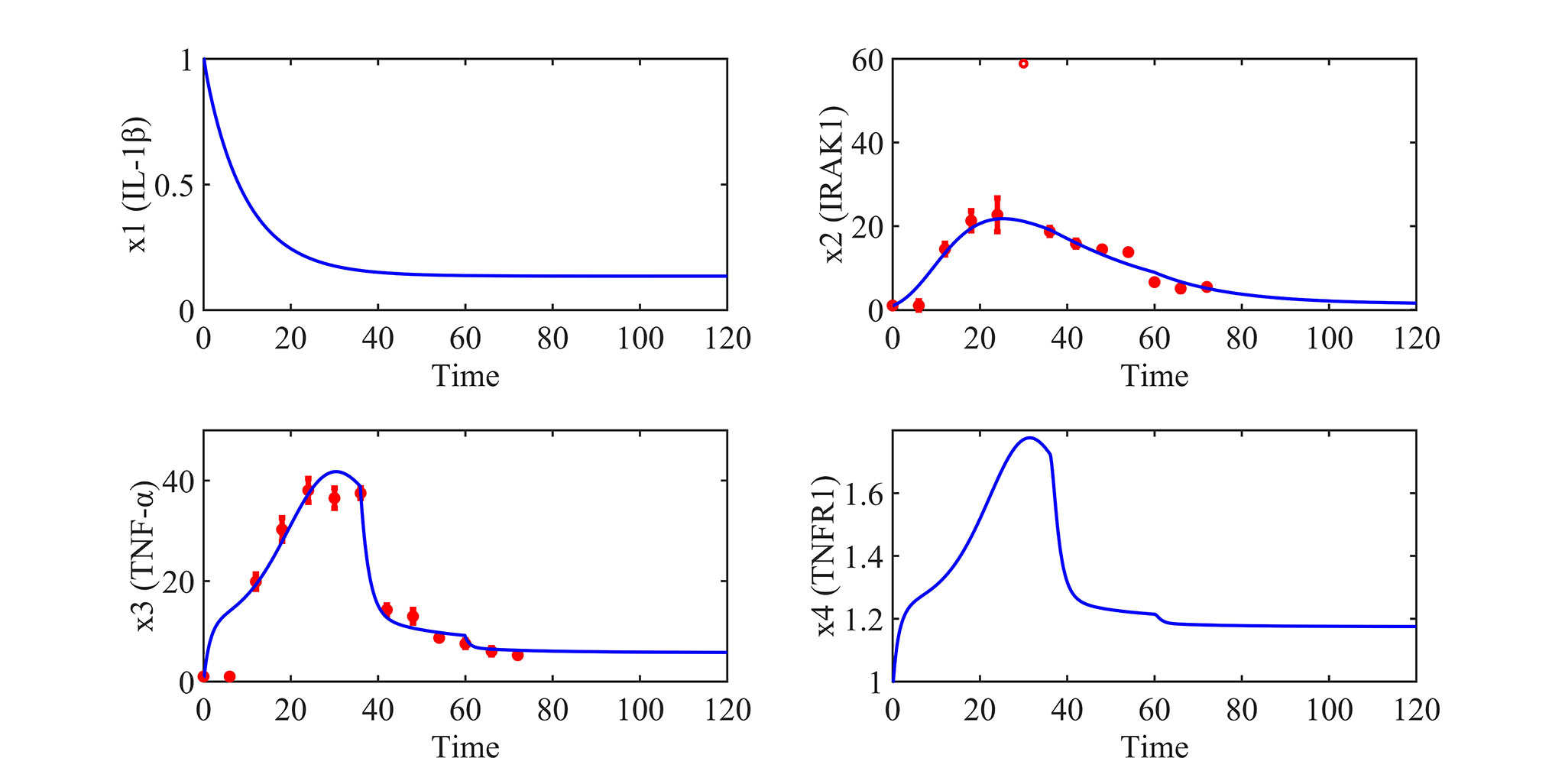

Supplement: Supplementary file 4 [file DataSheet2.zip › Supplementary material_image2/Parameter_d10(大)/1.jpg]

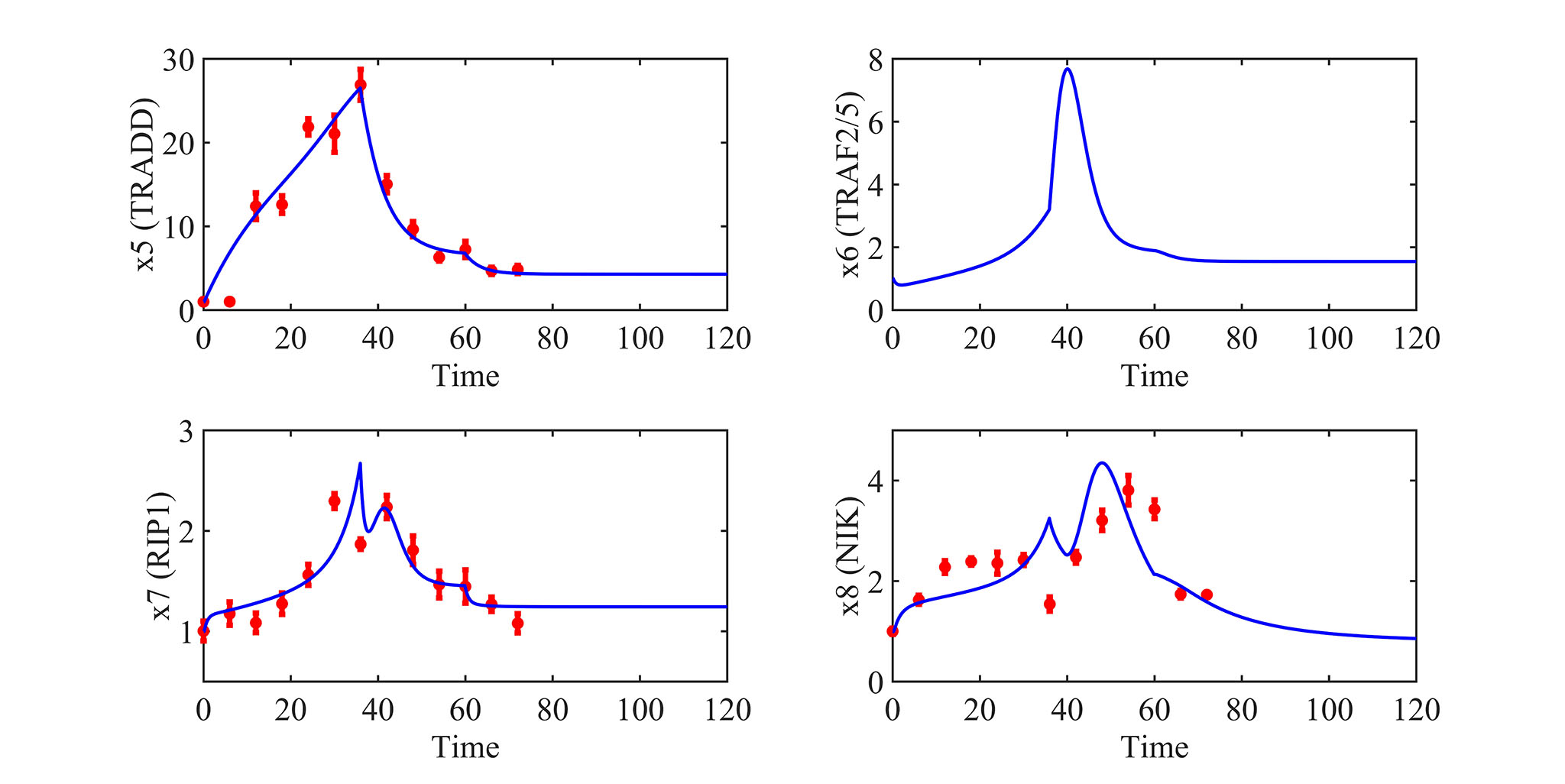

Supplement: Supplementary file 4 [file DataSheet2.zip › Supplementary material_image2/Parameter_d10(大)/2.jpg]

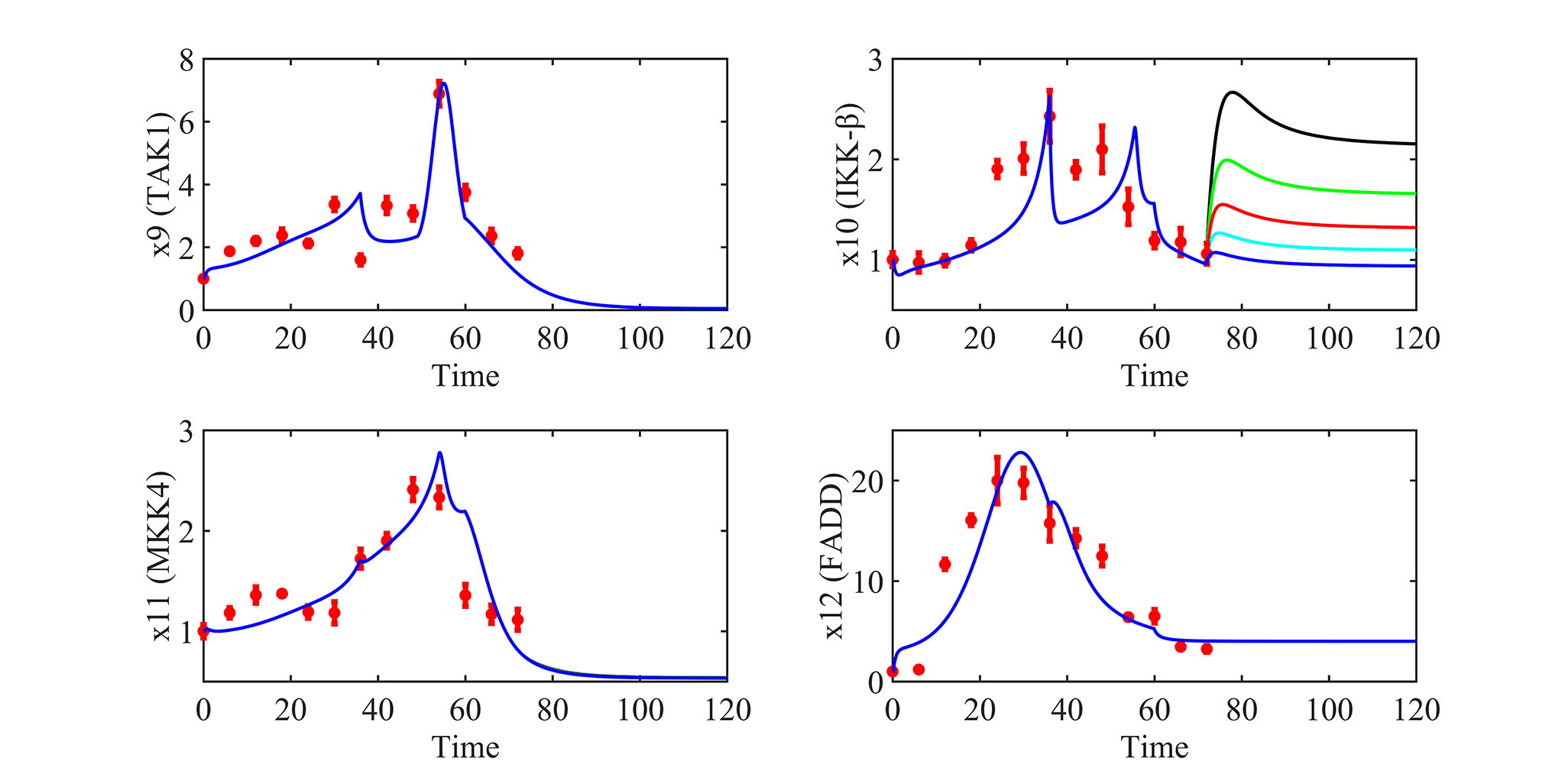

Supplement: Supplementary file 4 [file DataSheet2.zip › Supplementary material_image2/Parameter_d10(大)/3.jpg]

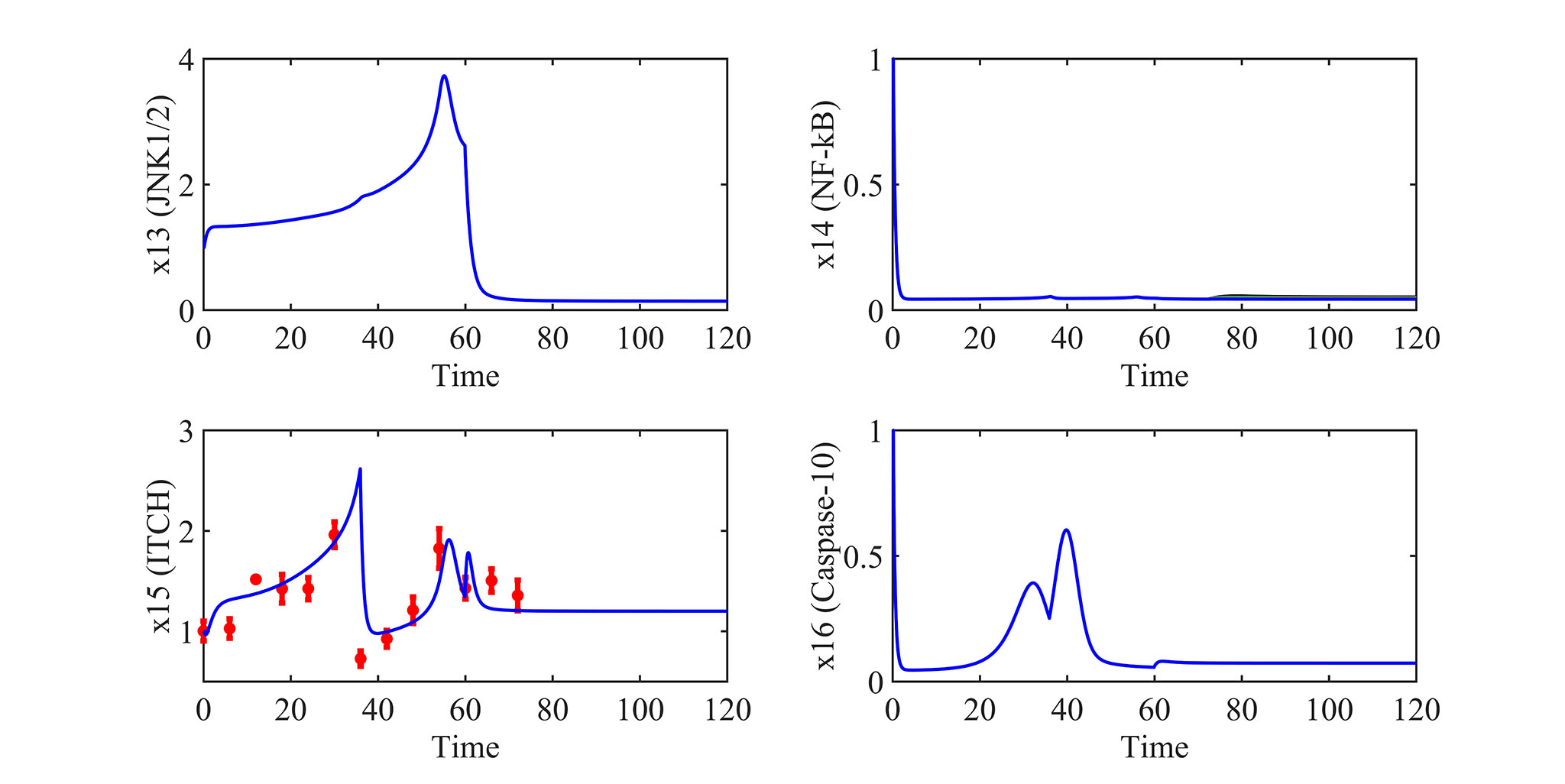

Supplement: Supplementary file 4 [file DataSheet2.zip › Supplementary material_image2/Parameter_d10(大)/4.jpg]

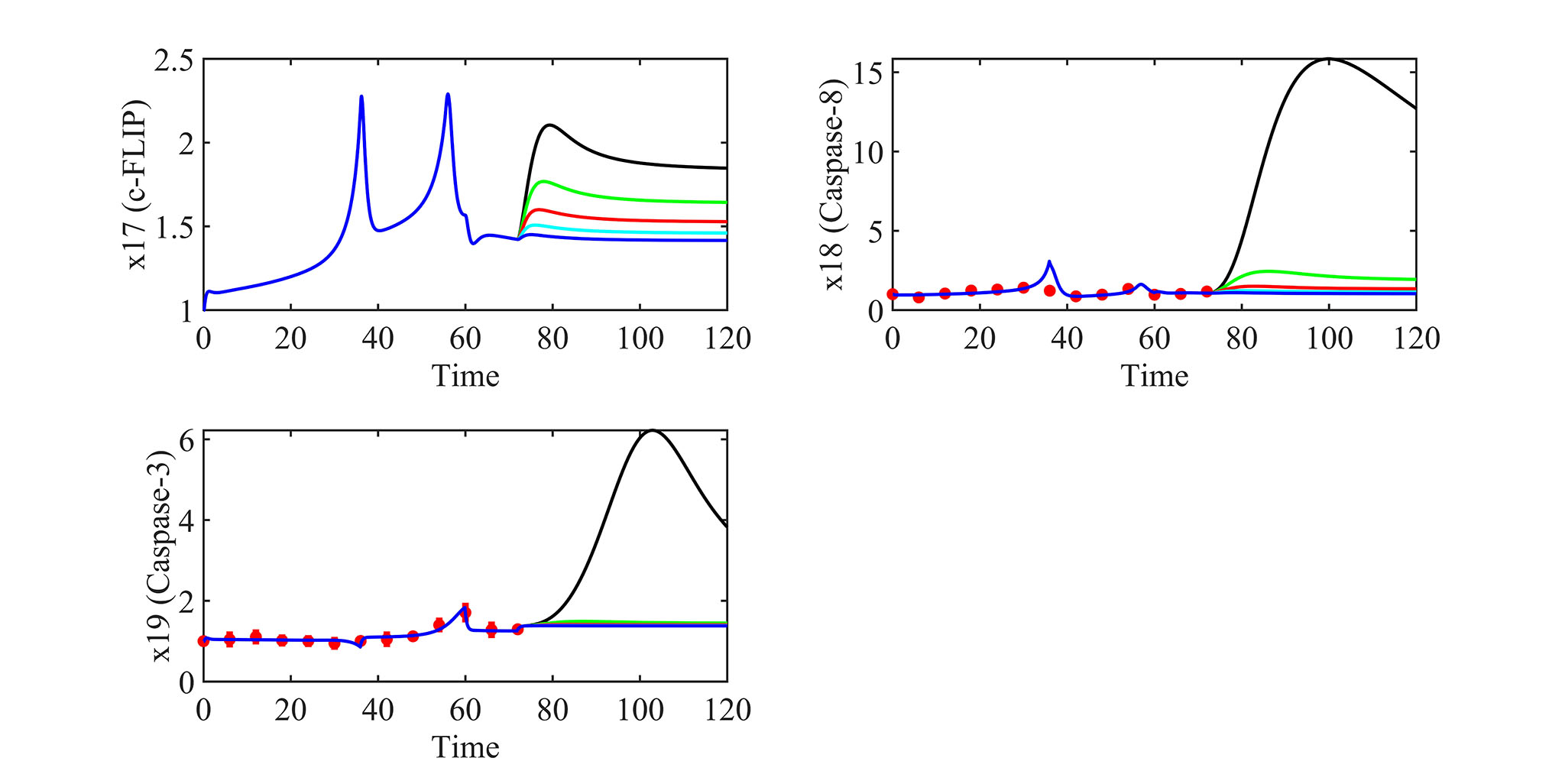

Supplement: Supplementary file 4 [file DataSheet2.zip › Supplementary material_image2/Parameter_d10(大)/5.jpg]

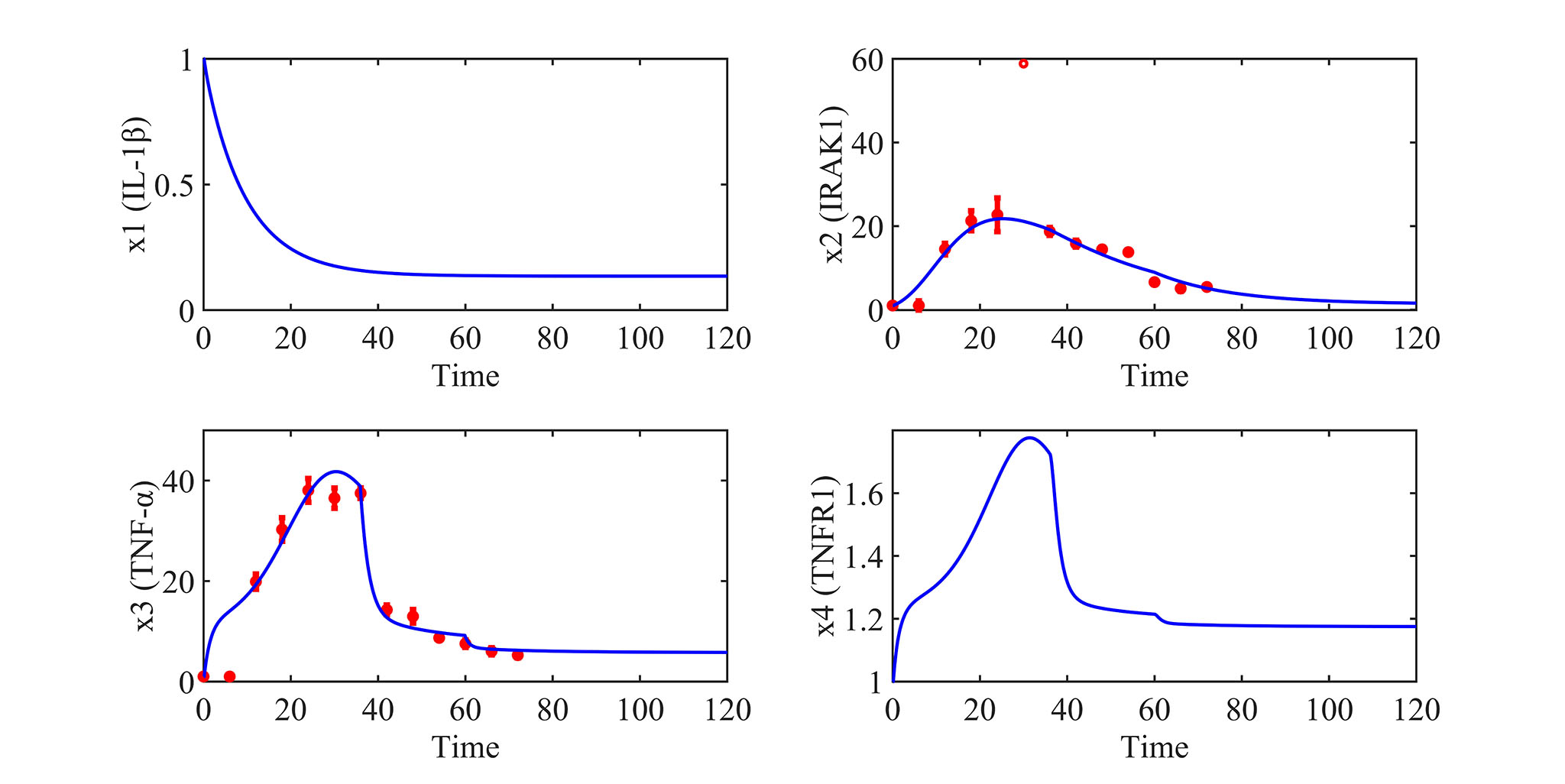

Supplement: Supplementary file 4 [file DataSheet2.zip › Supplementary material_image2/Parameter_d11(小)/1.jpg]

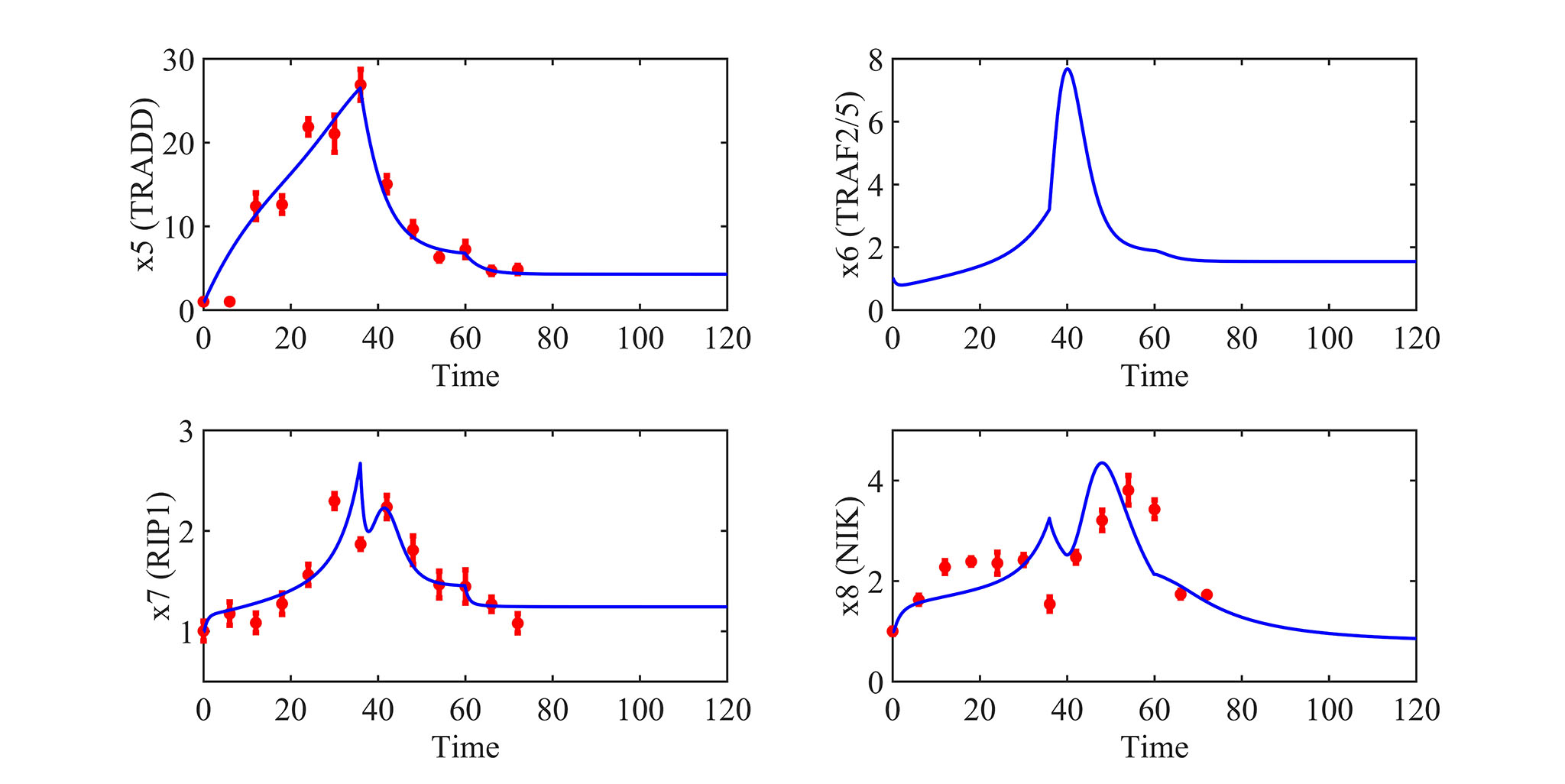

Supplement: Supplementary file 4 [file DataSheet2.zip › Supplementary material_image2/Parameter_d11(小)/2.jpg]

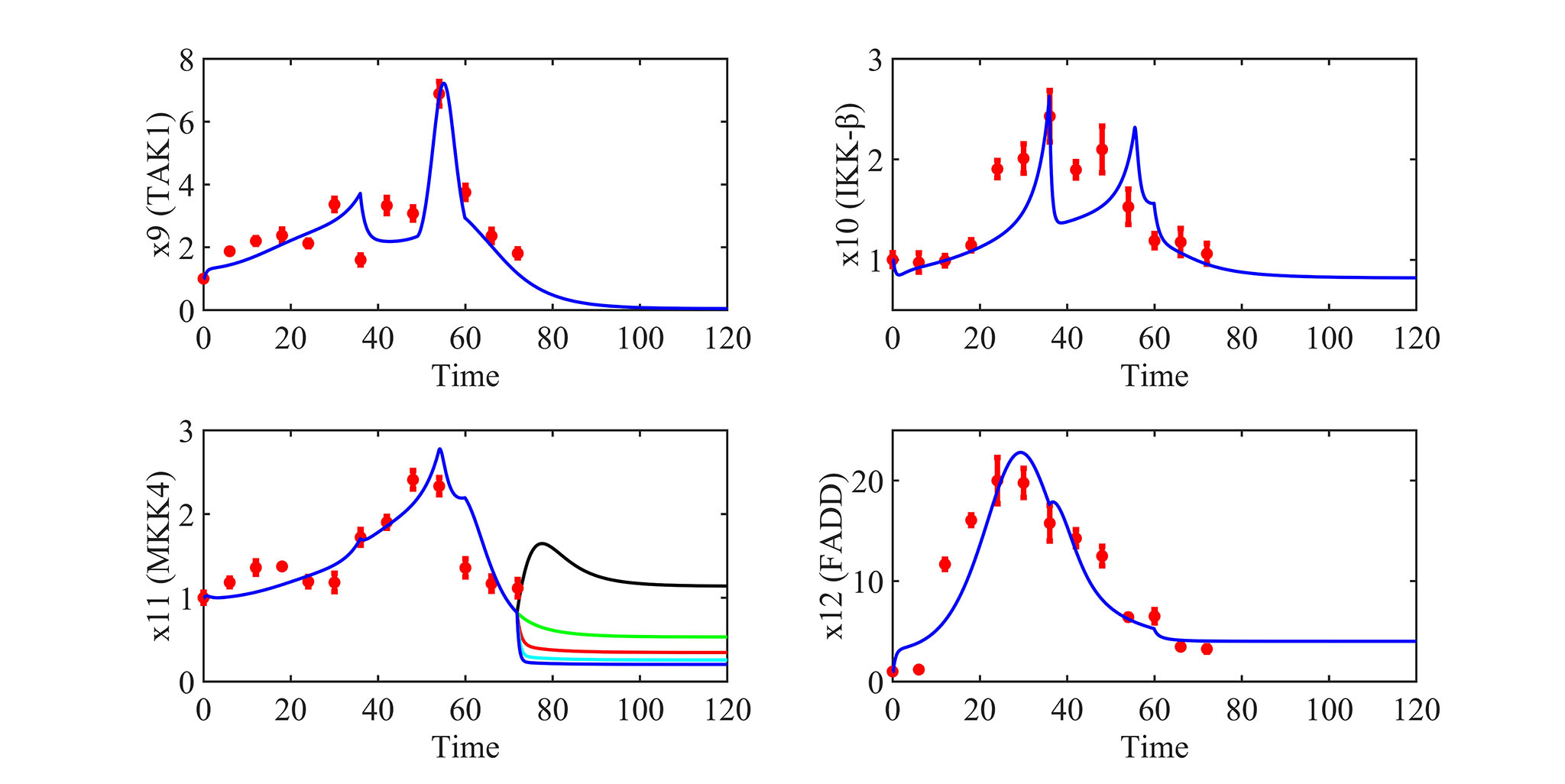

Supplement: Supplementary file 4 [file DataSheet2.zip › Supplementary material_image2/Parameter_d11(小)/3.jpg]

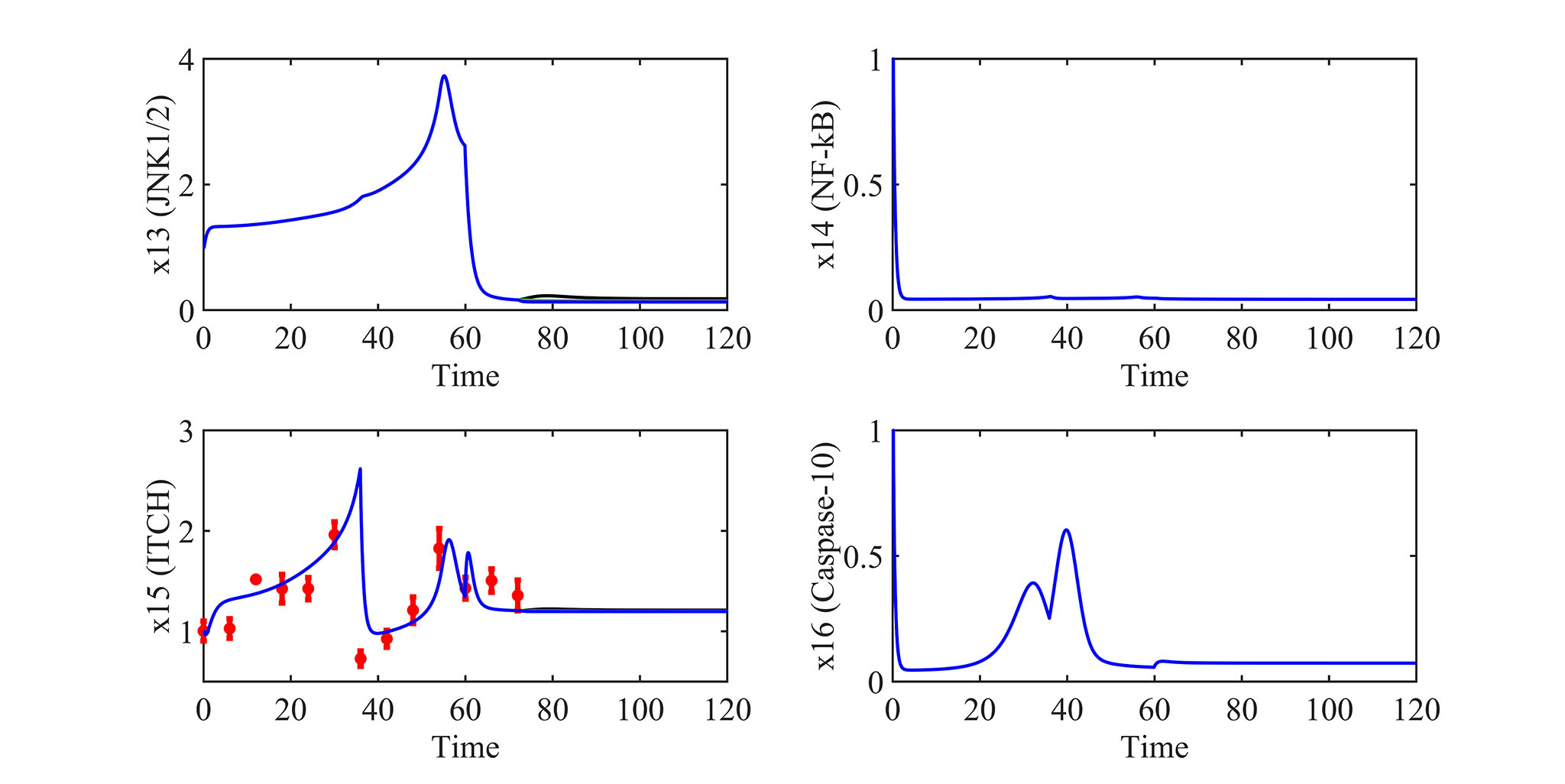

Supplement: Supplementary file 4 [file DataSheet2.zip › Supplementary material_image2/Parameter_d11(小)/4.jpg]

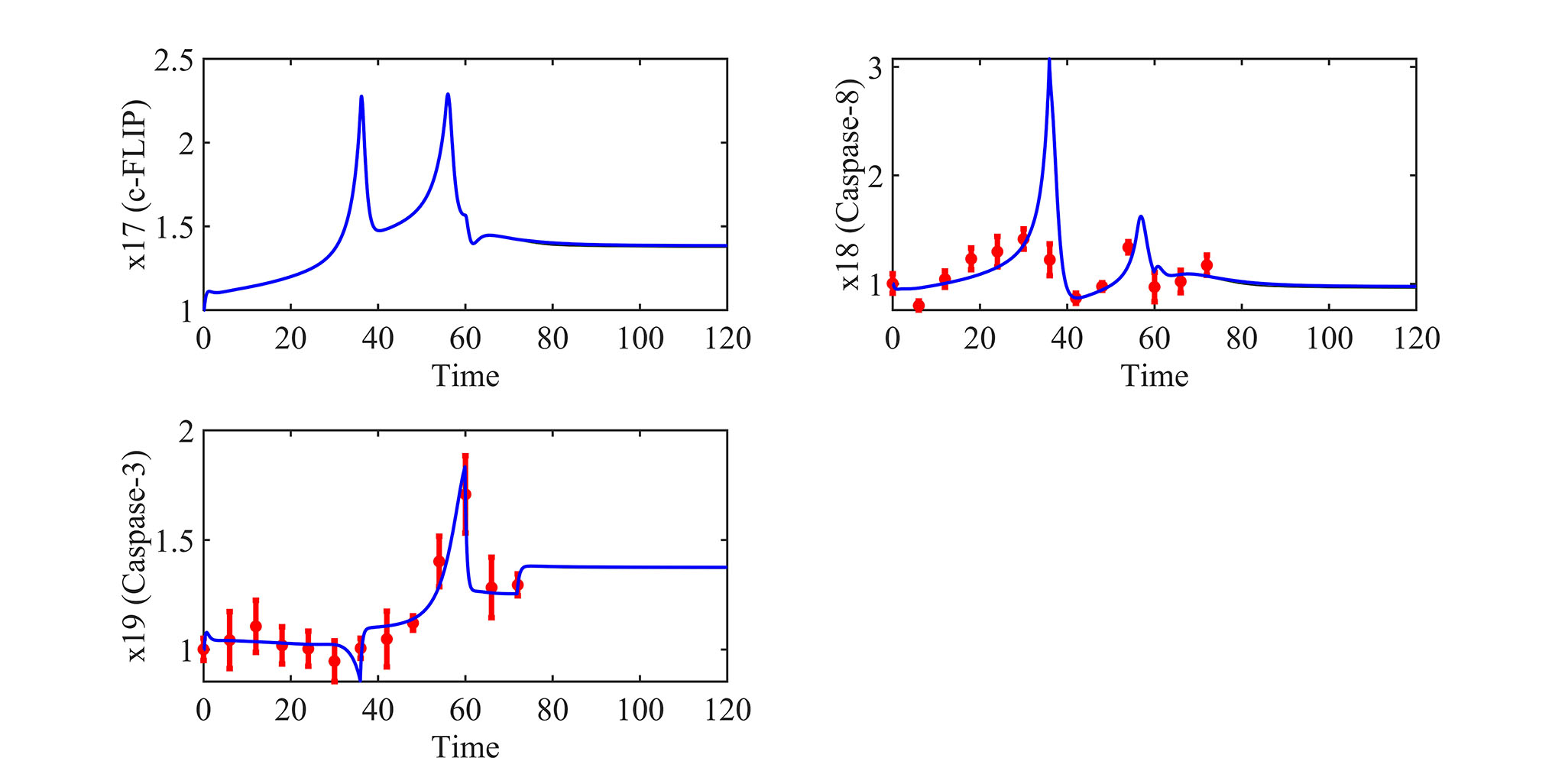

Supplement: Supplementary file 4 [file DataSheet2.zip › Supplementary material_image2/Parameter_d11(小)/5.jpg]

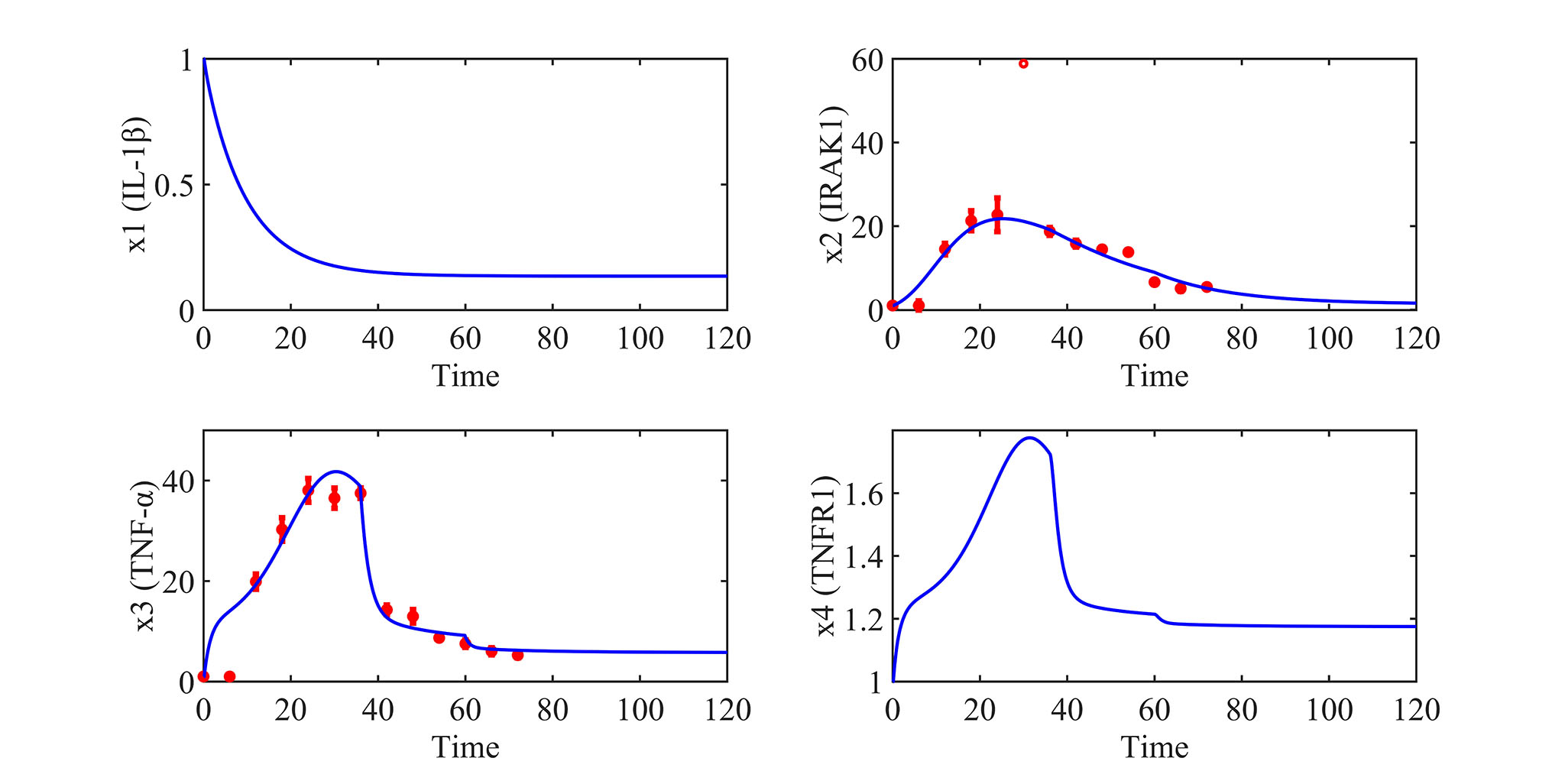

Supplement: Supplementary file 4 [file DataSheet2.zip › Supplementary material_image2/Parameter_d12(大)/1.jpg]

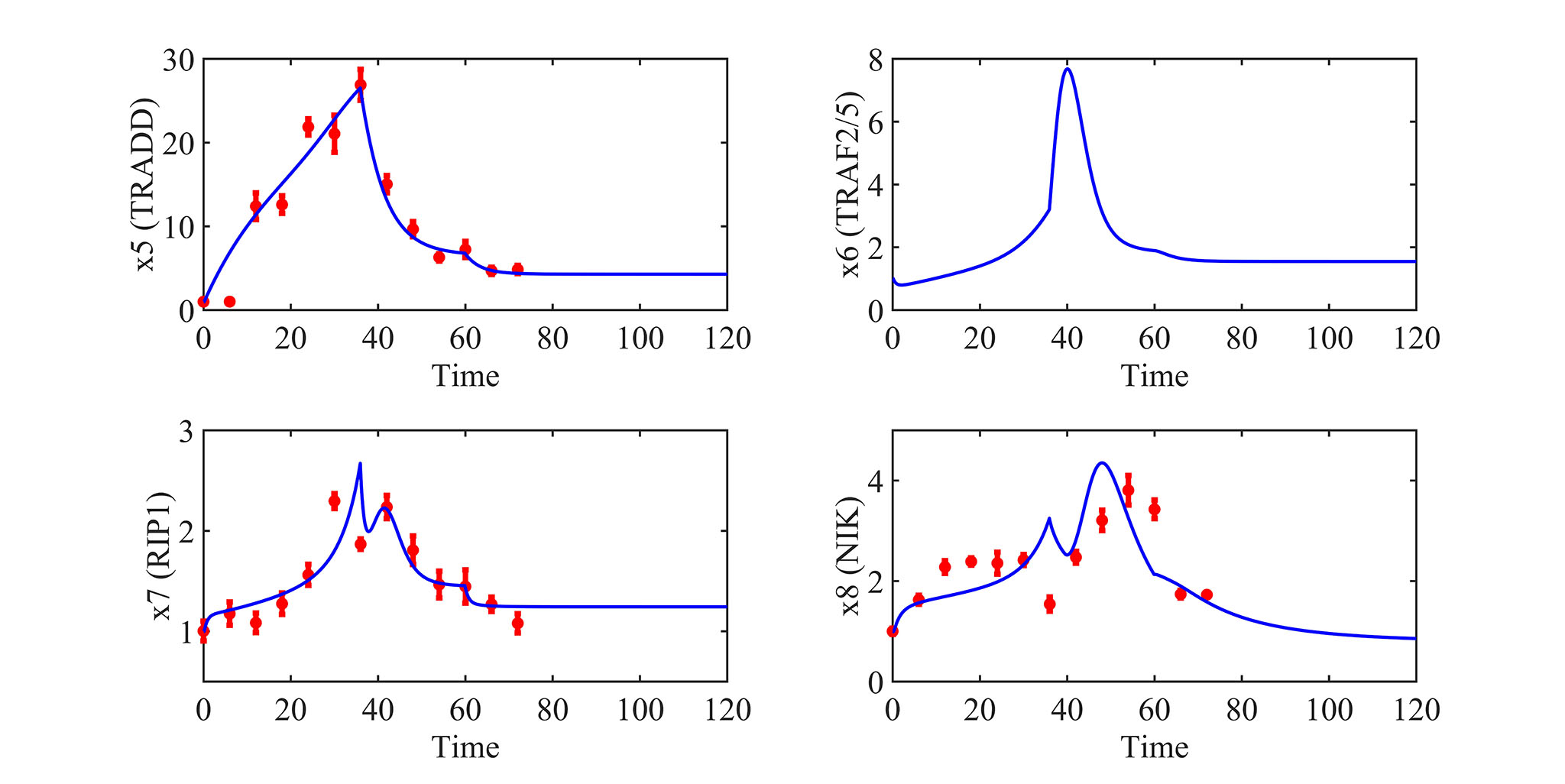

Supplement: Supplementary file 4 [file DataSheet2.zip › Supplementary material_image2/Parameter_d12(大)/2.jpg]

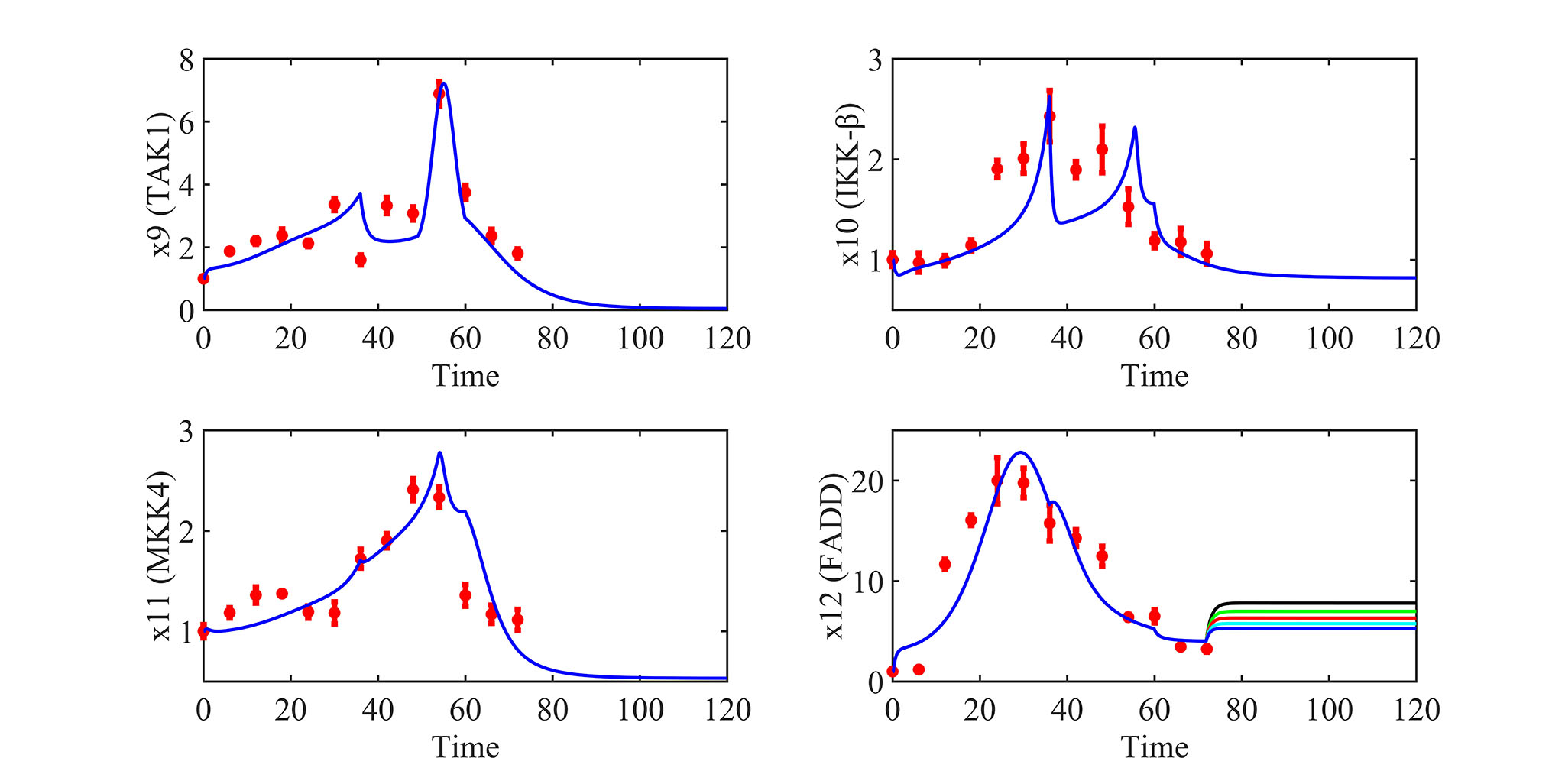

Supplement: Supplementary file 4 [file DataSheet2.zip › Supplementary material_image2/Parameter_d12(大)/3.jpg]

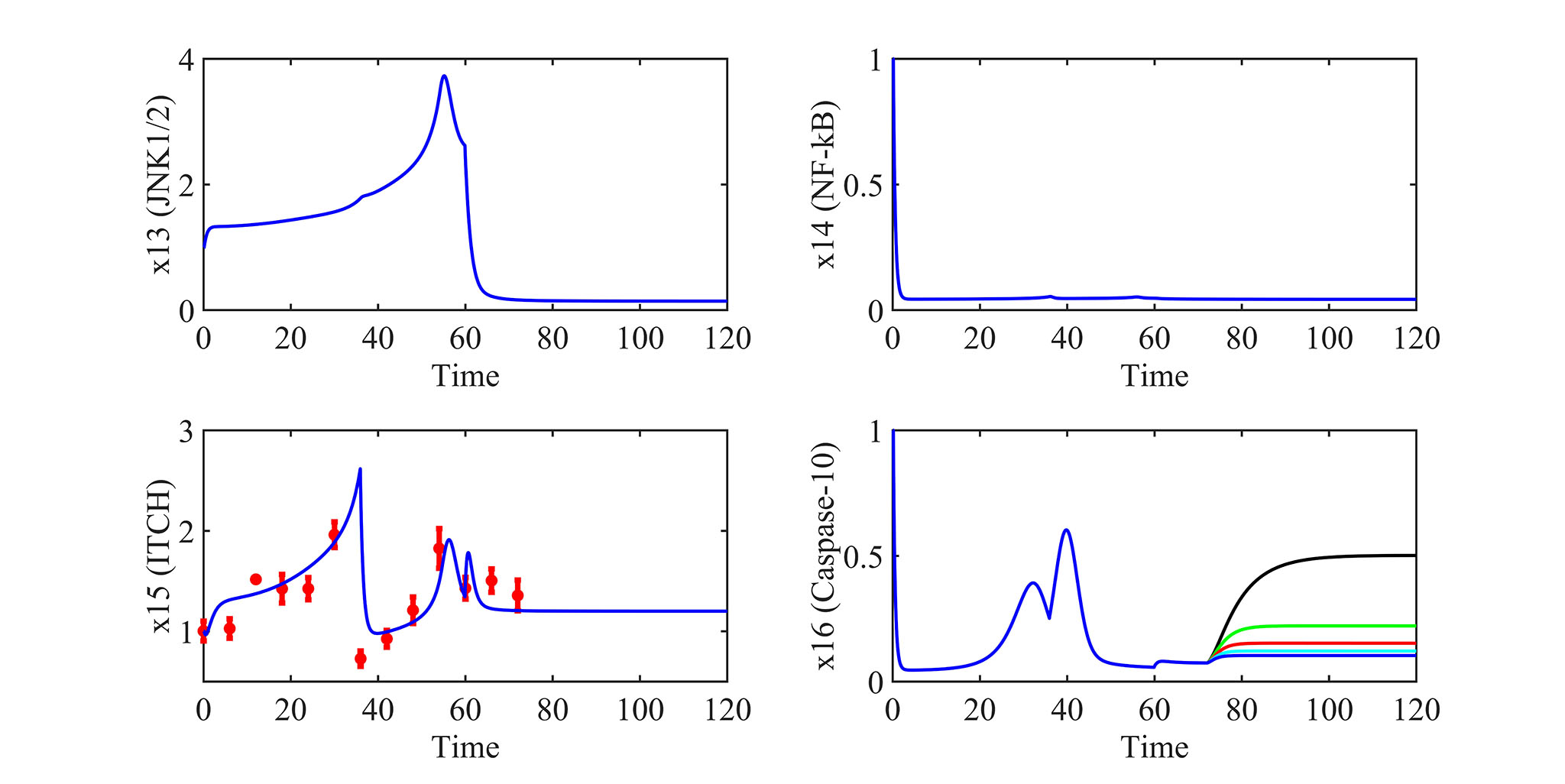

Supplement: Supplementary file 4 [file DataSheet2.zip › Supplementary material_image2/Parameter_d12(大)/4.jpg]

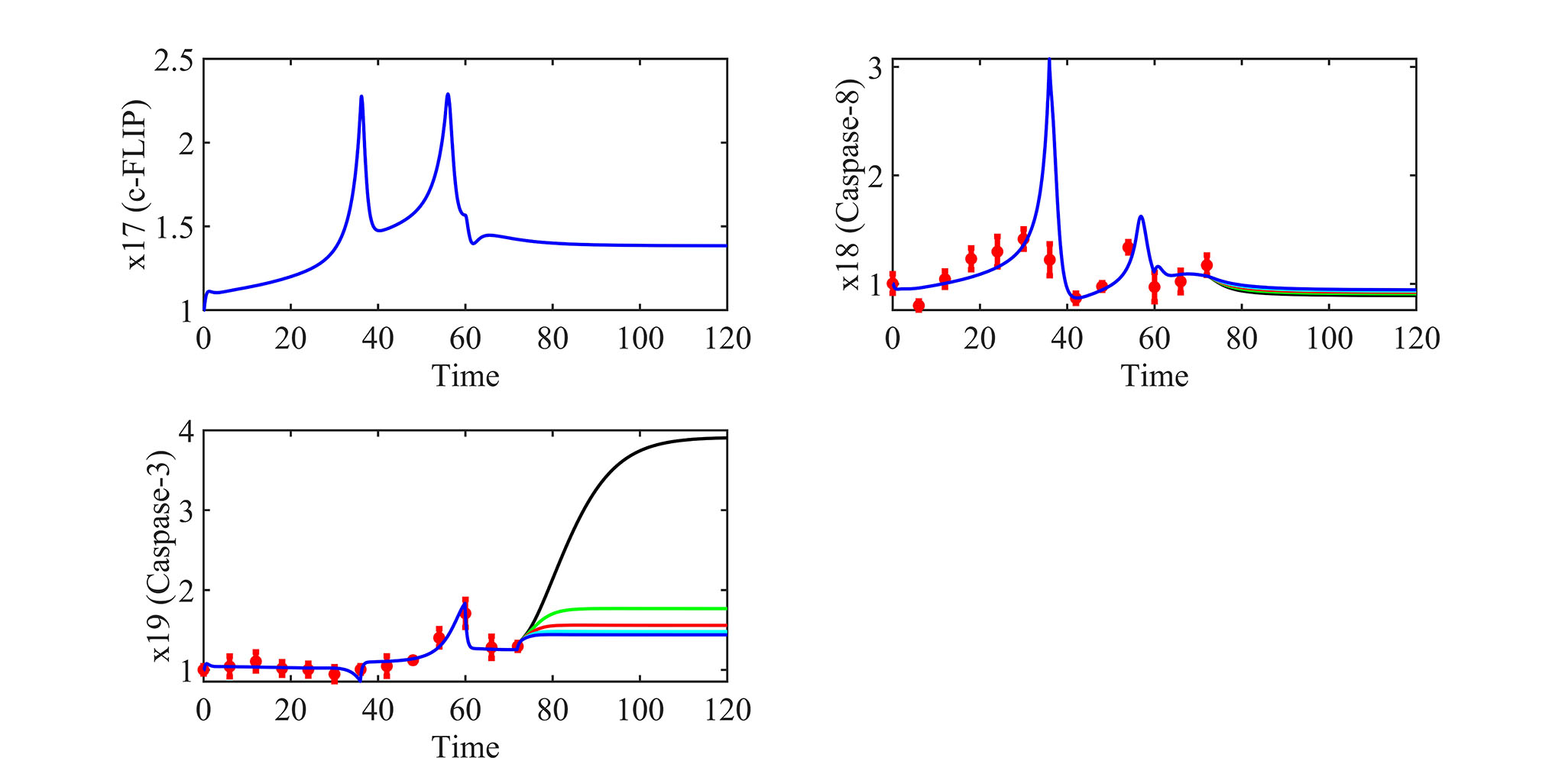

Supplement: Supplementary file 4 [file DataSheet2.zip › Supplementary material_image2/Parameter_d12(大)/5.jpg]

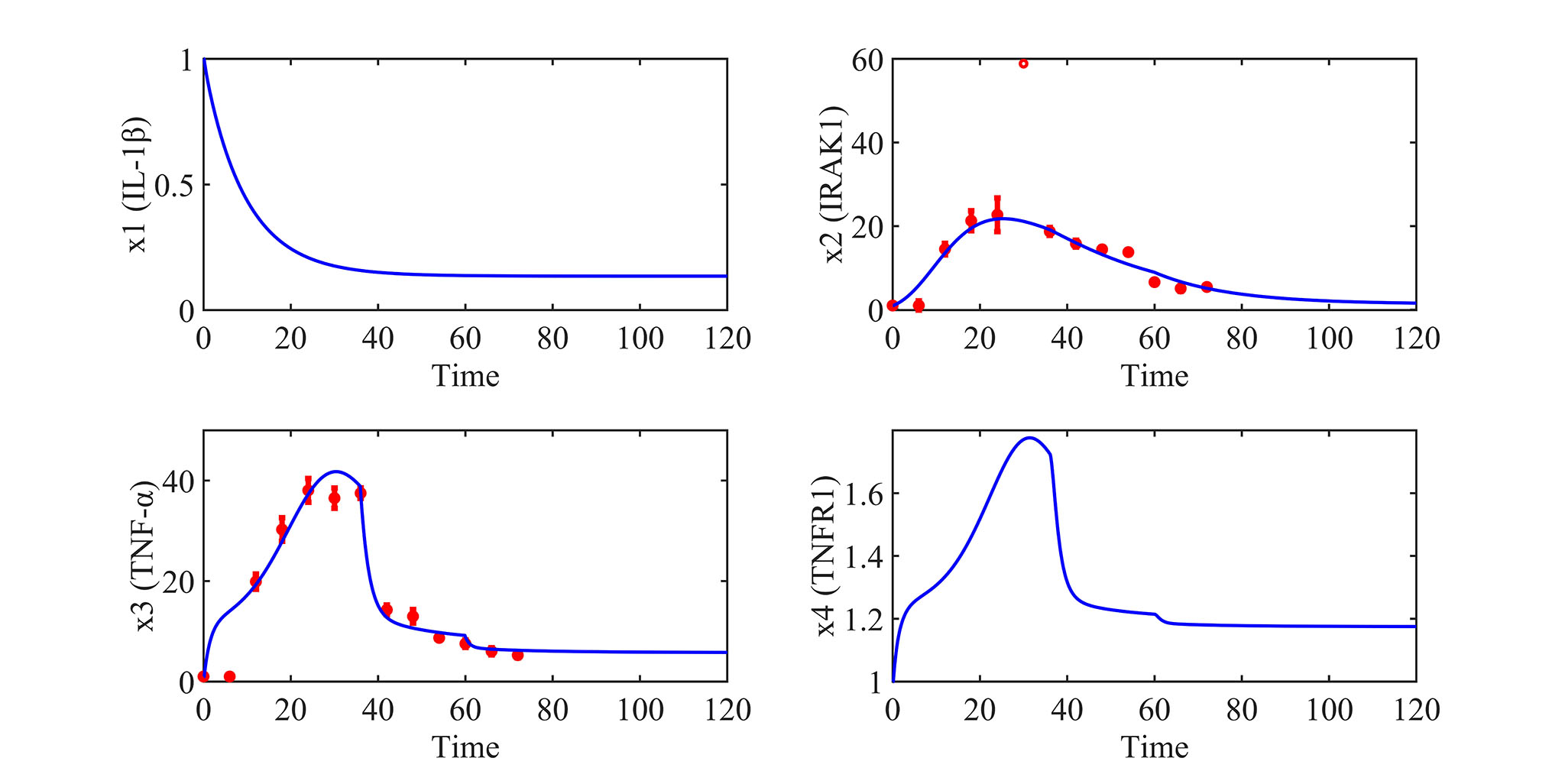

Supplement: Supplementary file 4 [file DataSheet2.zip › Supplementary material_image2/Parameter_d13(小)/1.jpg]

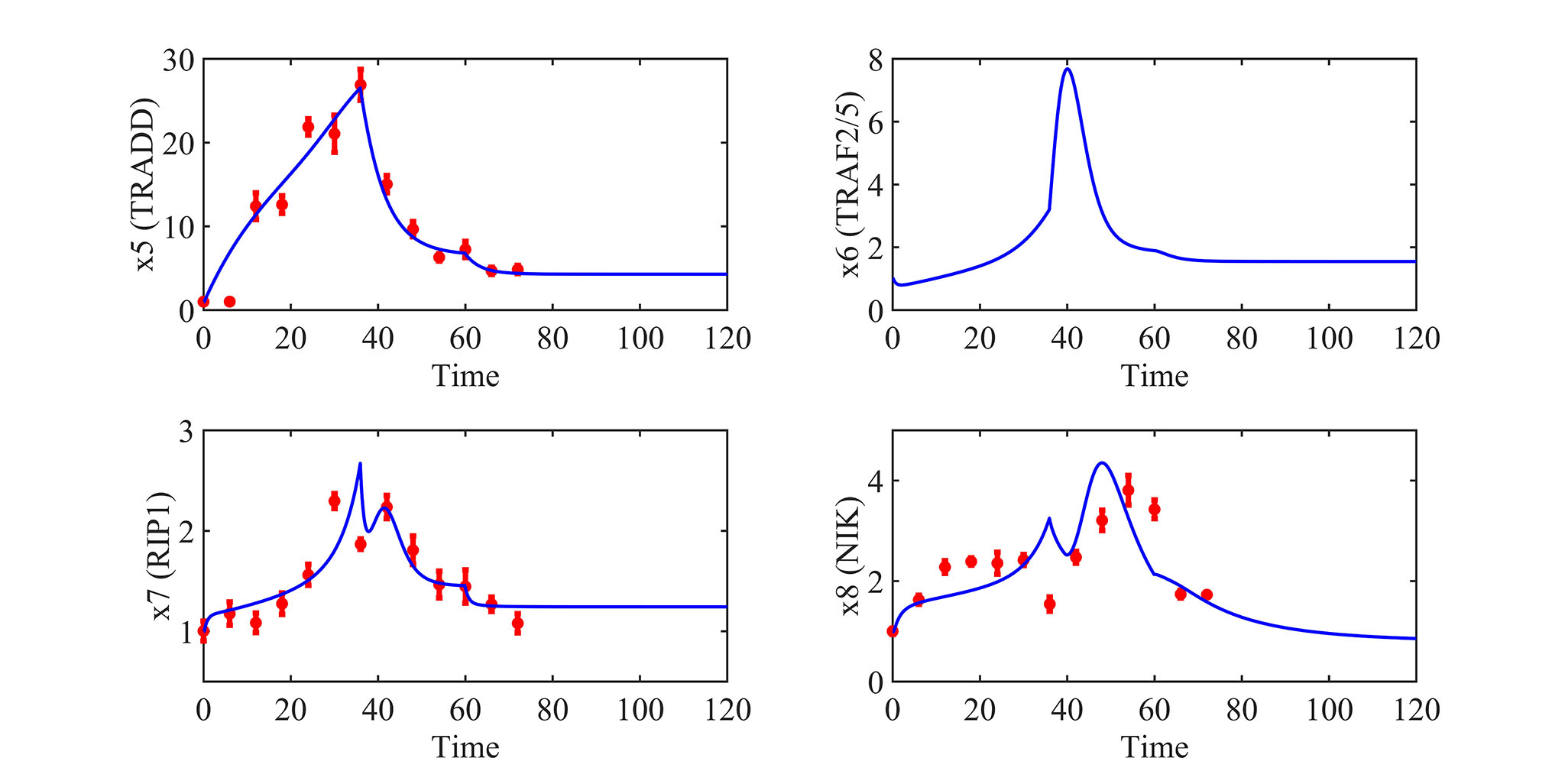

Supplement: Supplementary file 4 [file DataSheet2.zip › Supplementary material_image2/Parameter_d13(小)/2.jpg]

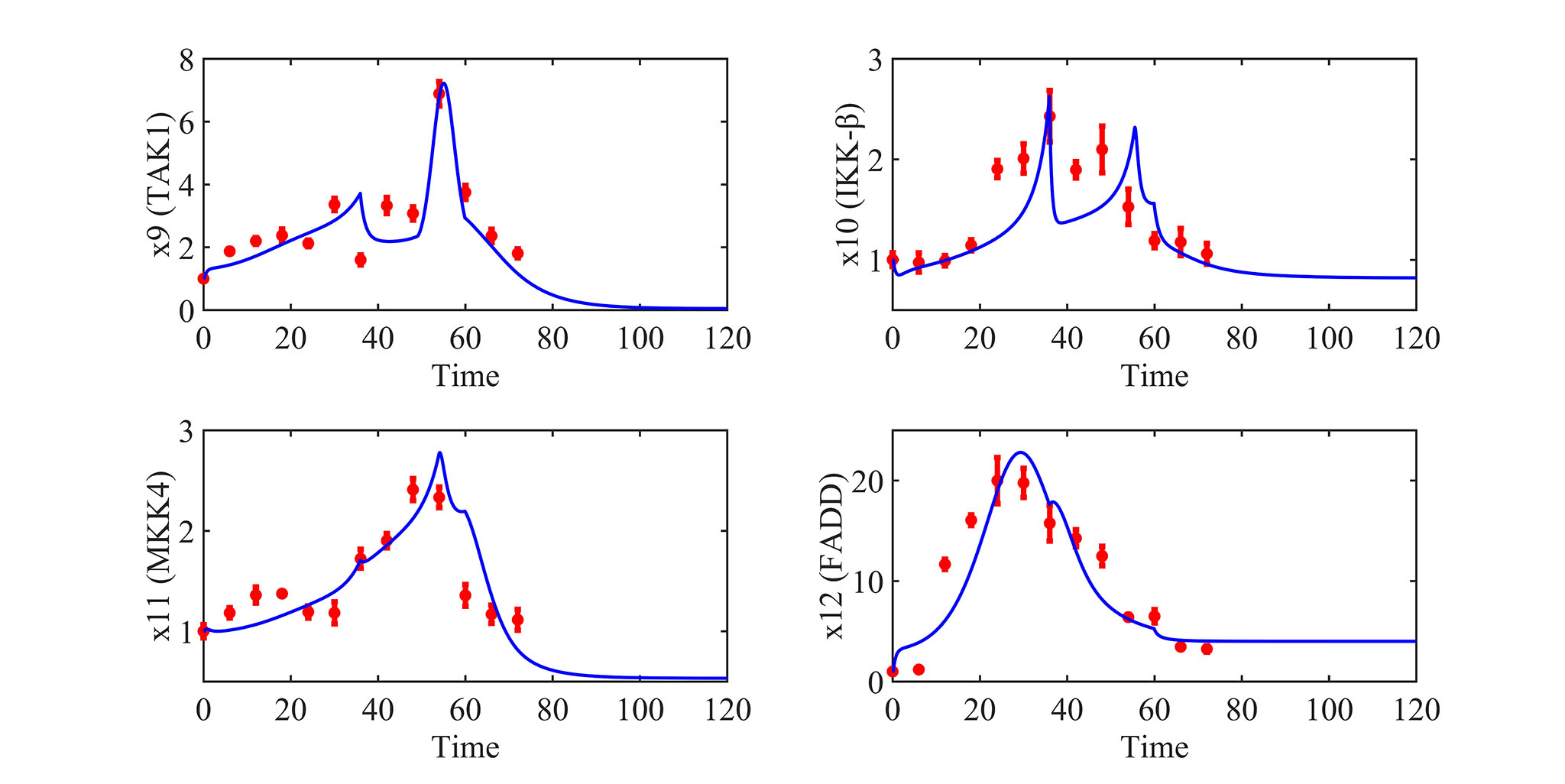

Supplement: Supplementary file 4 [file DataSheet2.zip › Supplementary material_image2/Parameter_d13(小)/3.jpg]

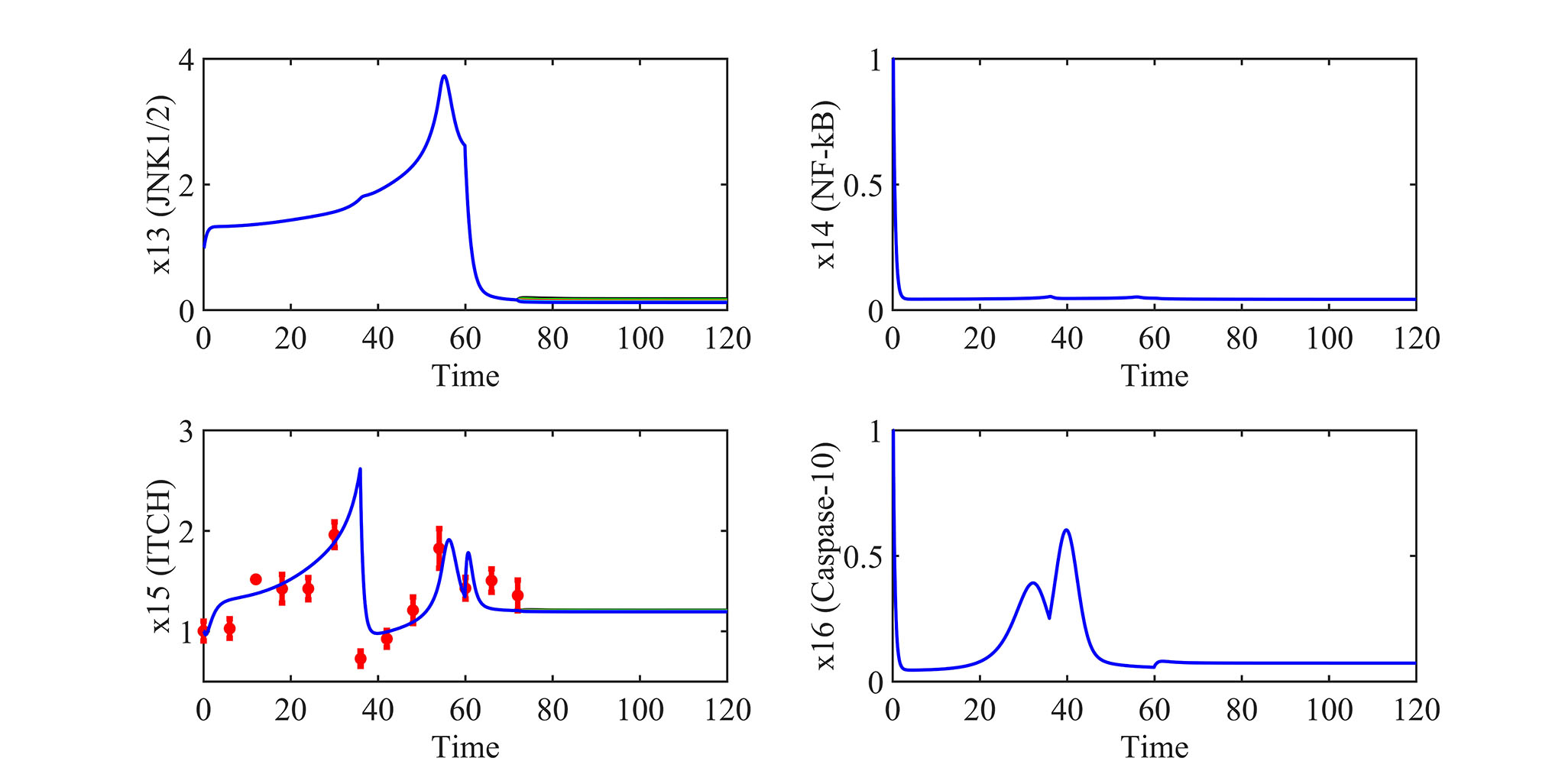

Supplement: Supplementary file 4 [file DataSheet2.zip › Supplementary material_image2/Parameter_d13(小)/4.jpg]

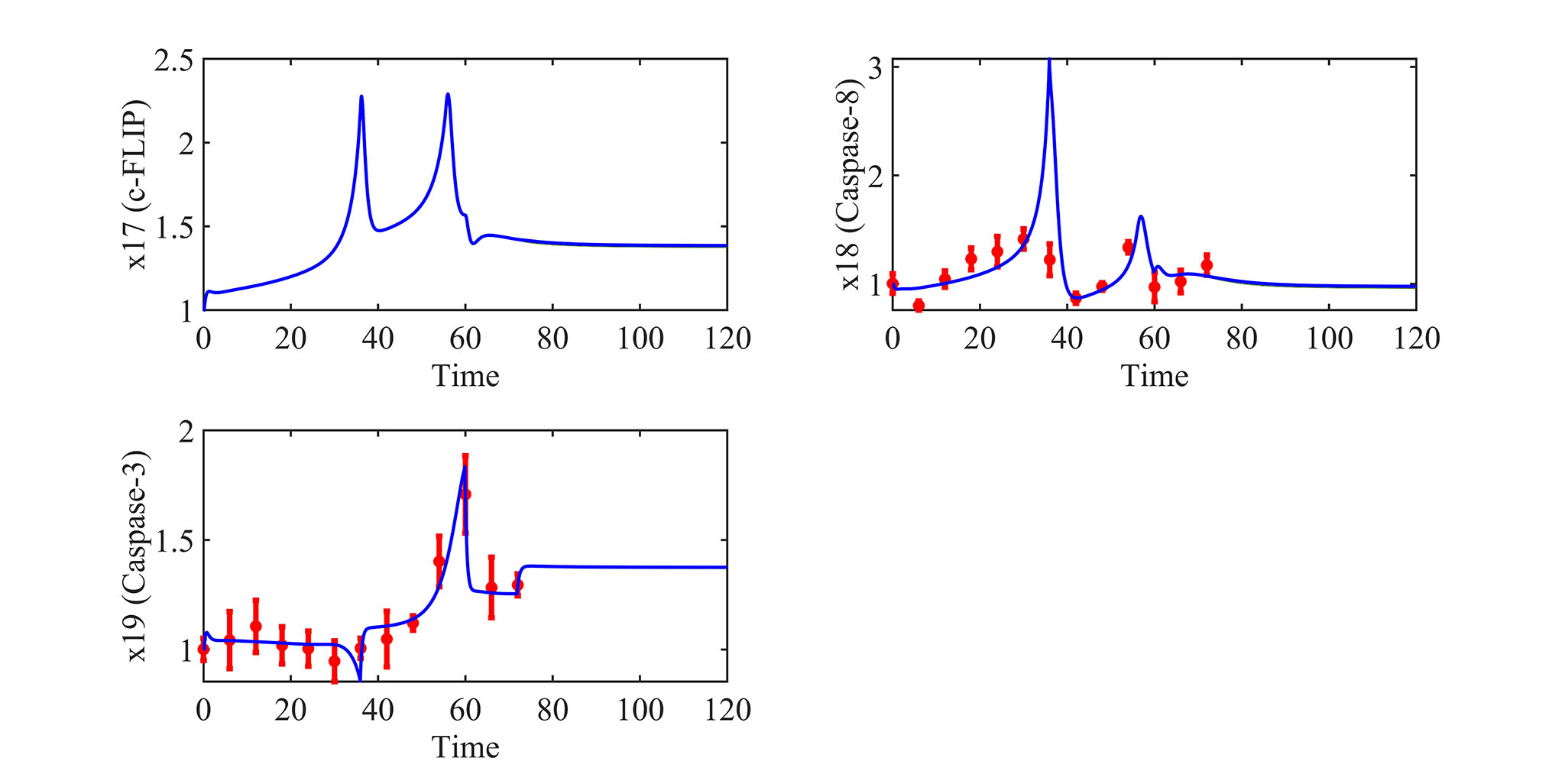

Supplement: Supplementary file 4 [file DataSheet2.zip › Supplementary material_image2/Parameter_d13(小)/5.jpg]

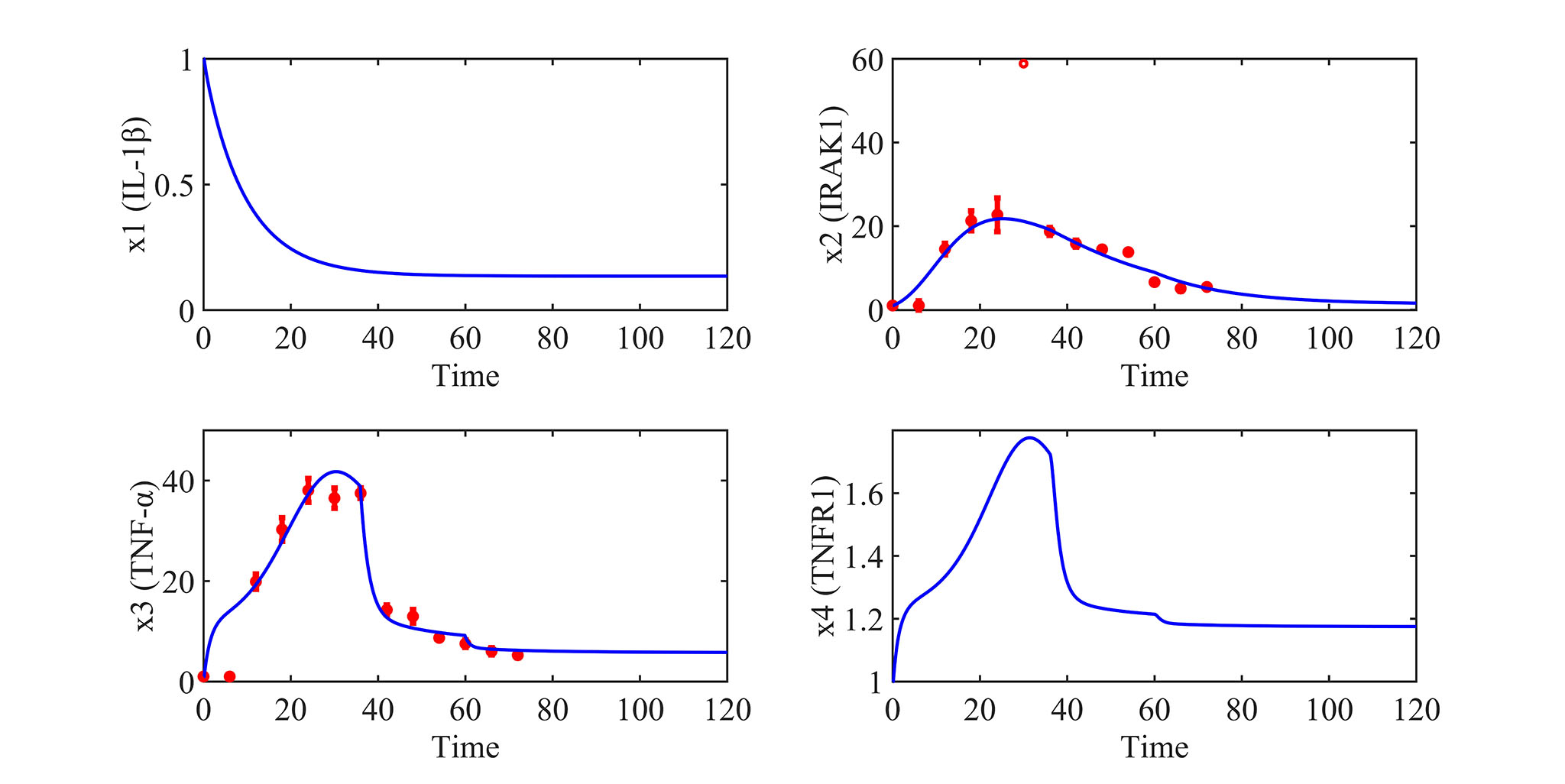

Supplement: Supplementary file 4 [file DataSheet2.zip › Supplementary material_image2/Parameter_d14(小)/1.jpg]

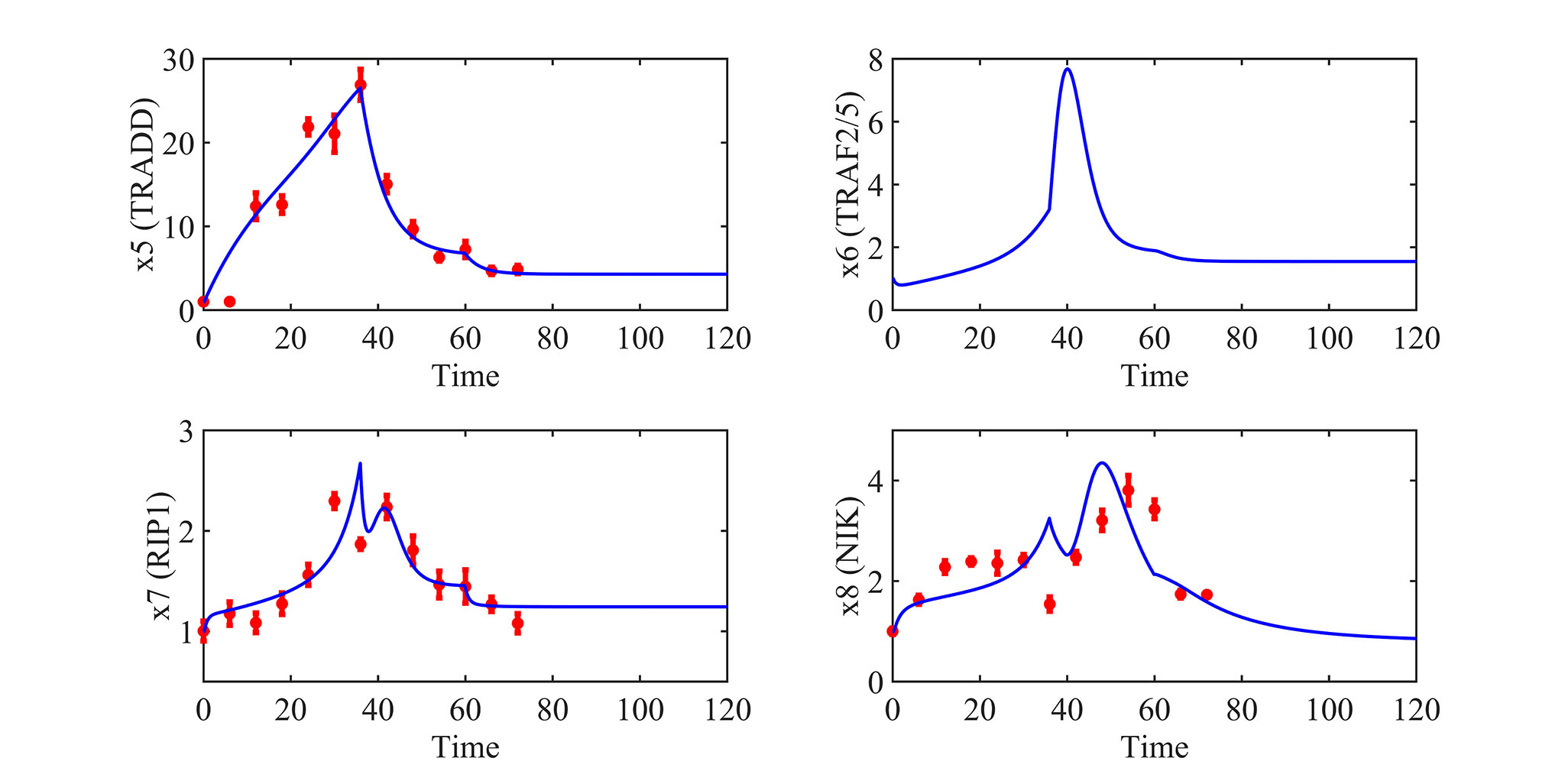

Supplement: Supplementary file 4 [file DataSheet2.zip › Supplementary material_image2/Parameter_d14(小)/2.jpg]

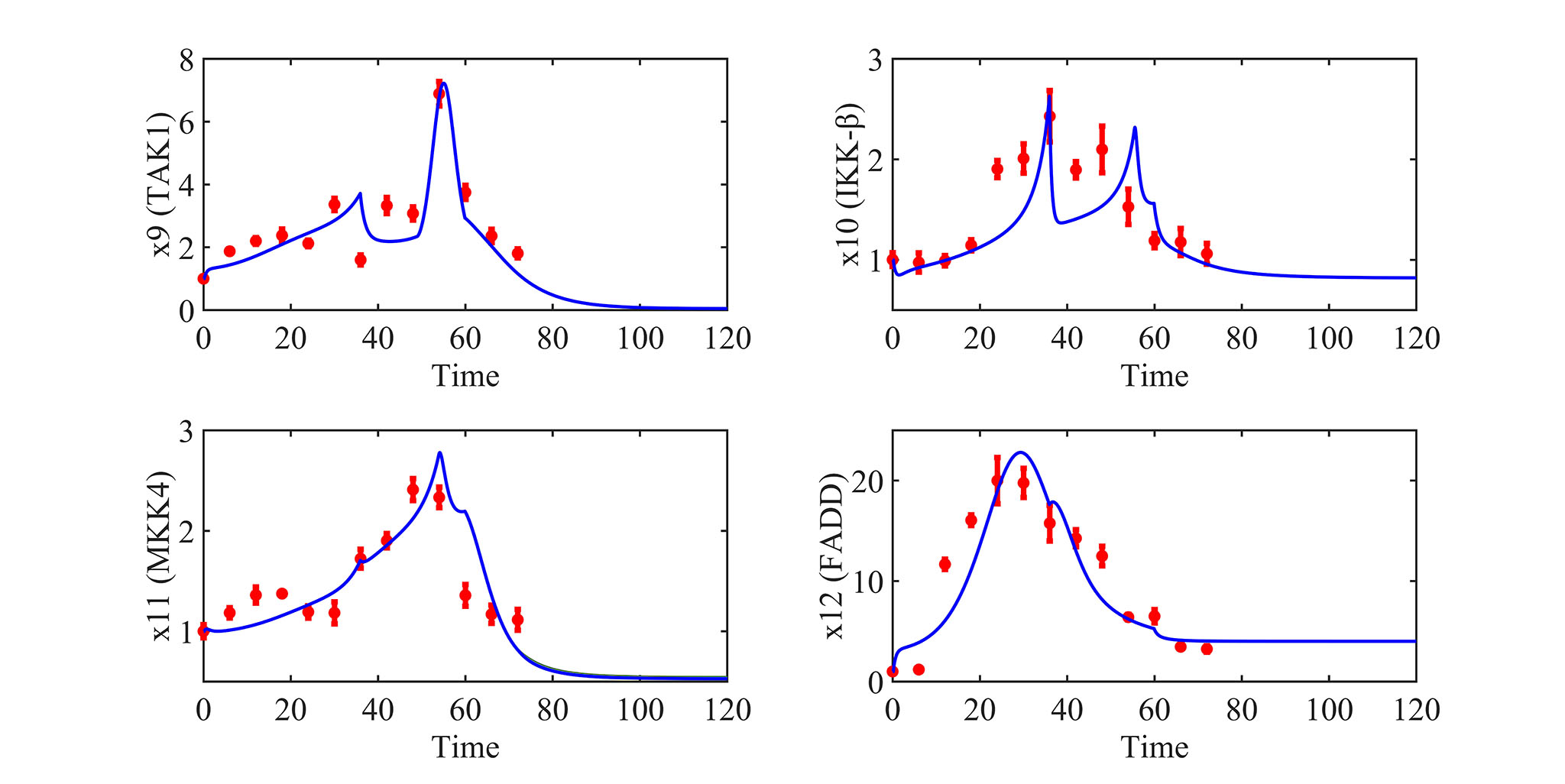

Supplement: Supplementary file 4 [file DataSheet2.zip › Supplementary material_image2/Parameter_d14(小)/3.jpg]

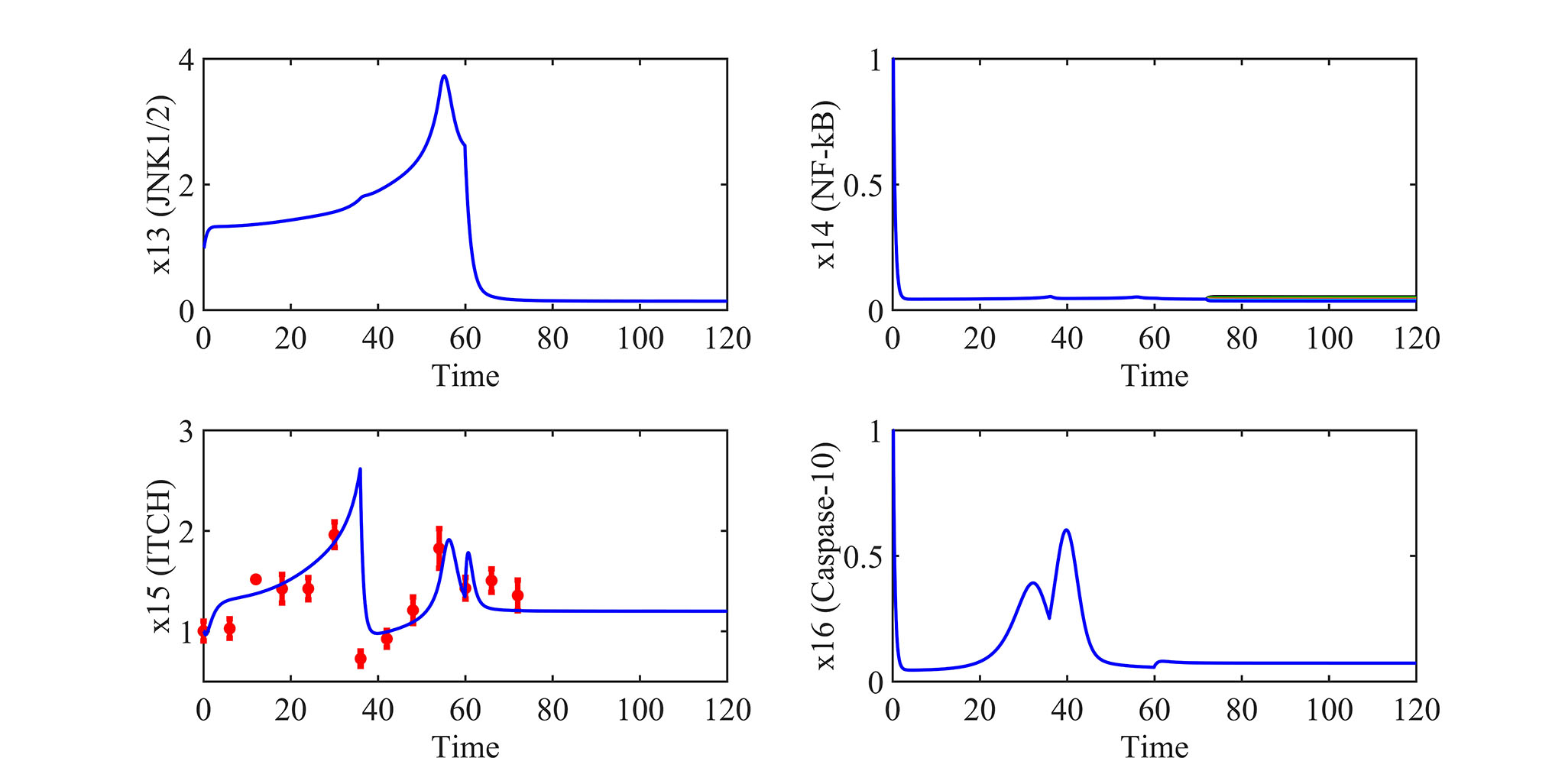

Supplement: Supplementary file 4 [file DataSheet2.zip › Supplementary material_image2/Parameter_d14(小)/4.jpg]

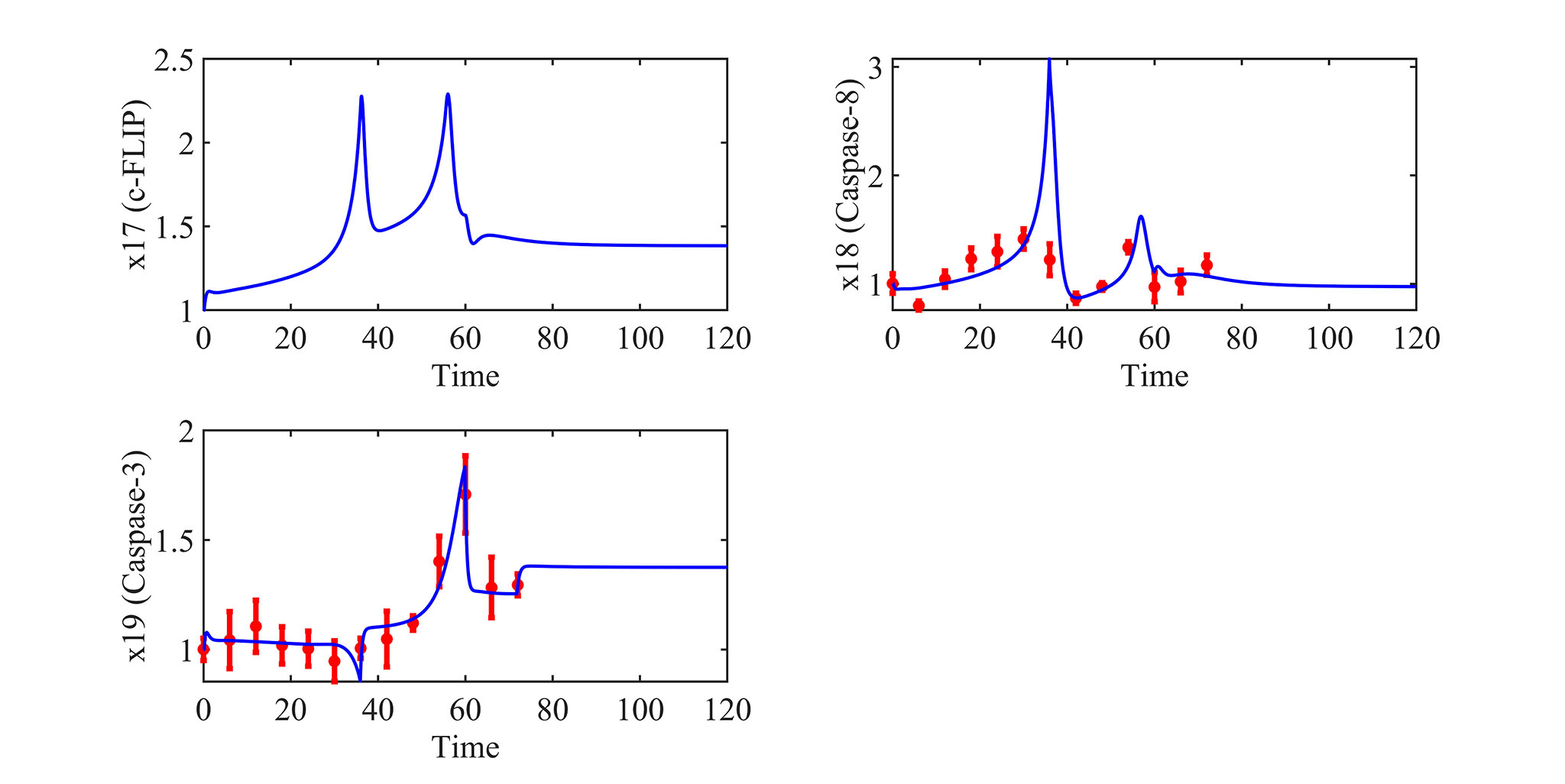

Supplement: Supplementary file 4 [file DataSheet2.zip › Supplementary material_image2/Parameter_d14(小)/5.jpg]
