# Supplementary figures and images for: Mathematical modeling and application of IL-1β/TNF signaling pathway in regulating chondrocyte apoptosis (part 1 of 4)
Source: Front Cell Dev Biol. 2023 Nov 2;11:1288431. doi: 10.3389/fcell.2023.1288431 (PMC10652750; doi:10.3389/fcell.2023.1288431)

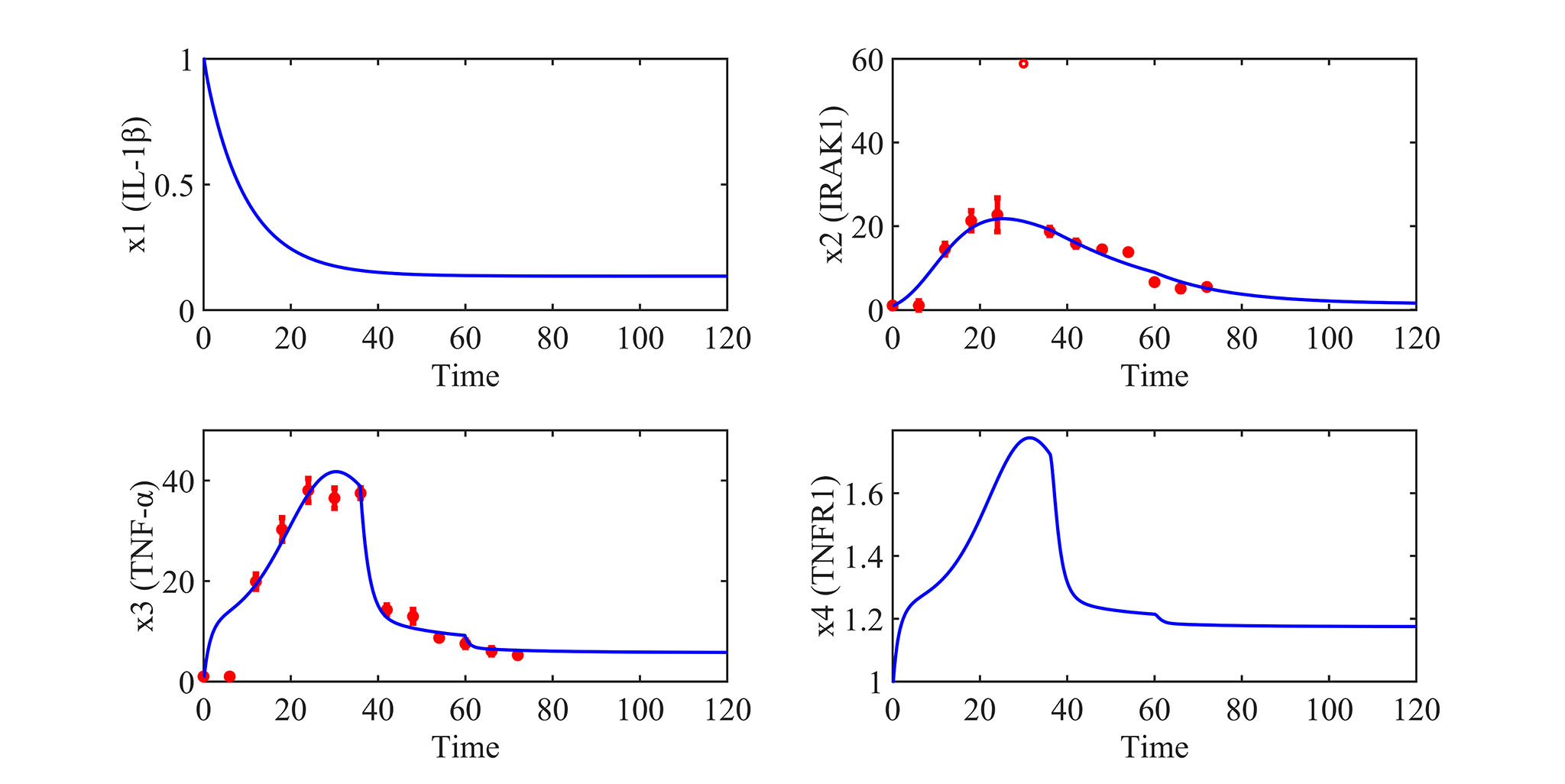

Supplement: Supplementary file 2 [file DataSheet1.zip › Supplementary material_image1/Parameter_a10(大)/1.jpg]

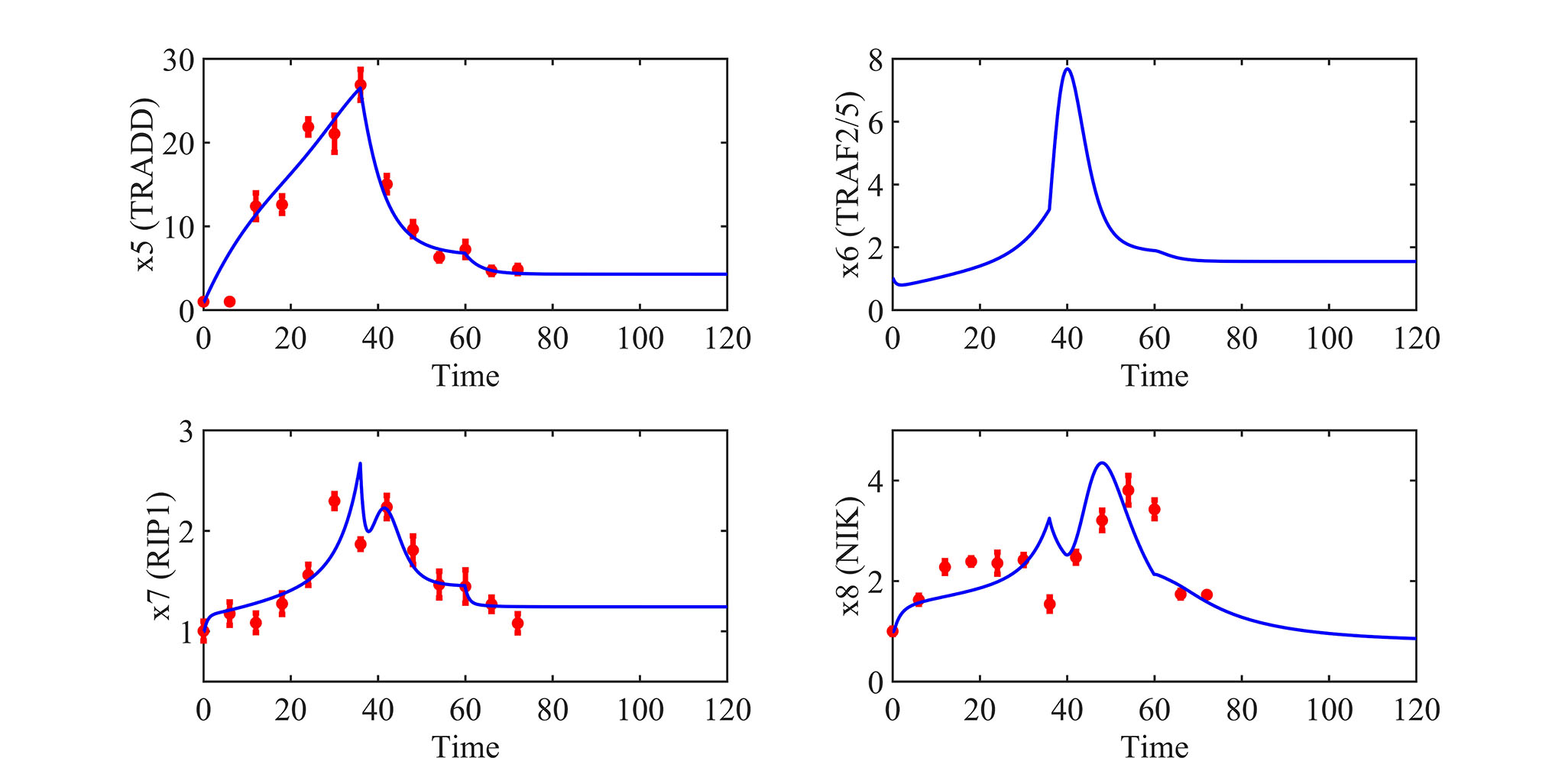

Supplement: Supplementary file 2 [file DataSheet1.zip › Supplementary material_image1/Parameter_a10(大)/2.jpg]

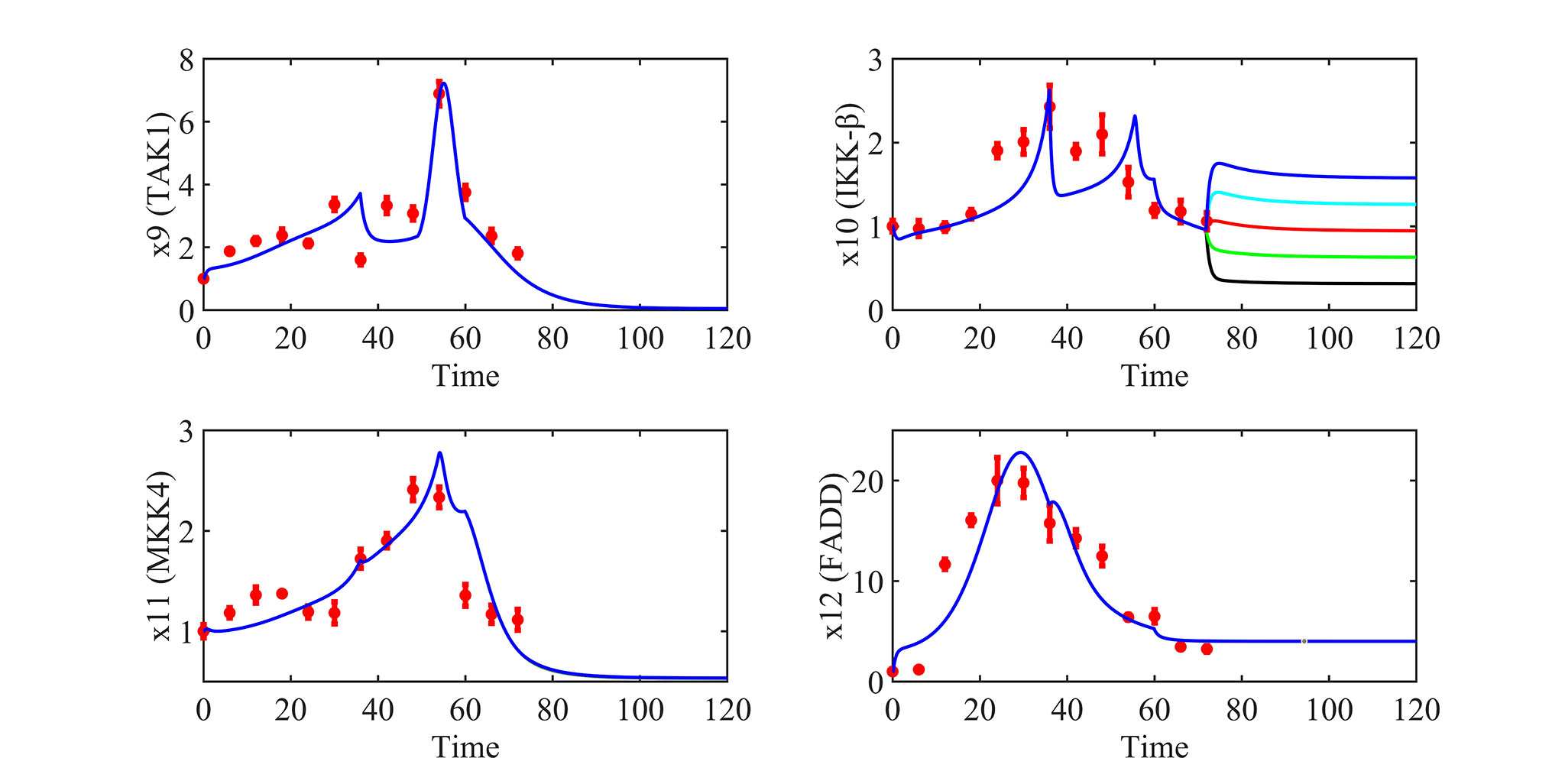

Supplement: Supplementary file 2 [file DataSheet1.zip › Supplementary material_image1/Parameter_a10(大)/3.jpg]

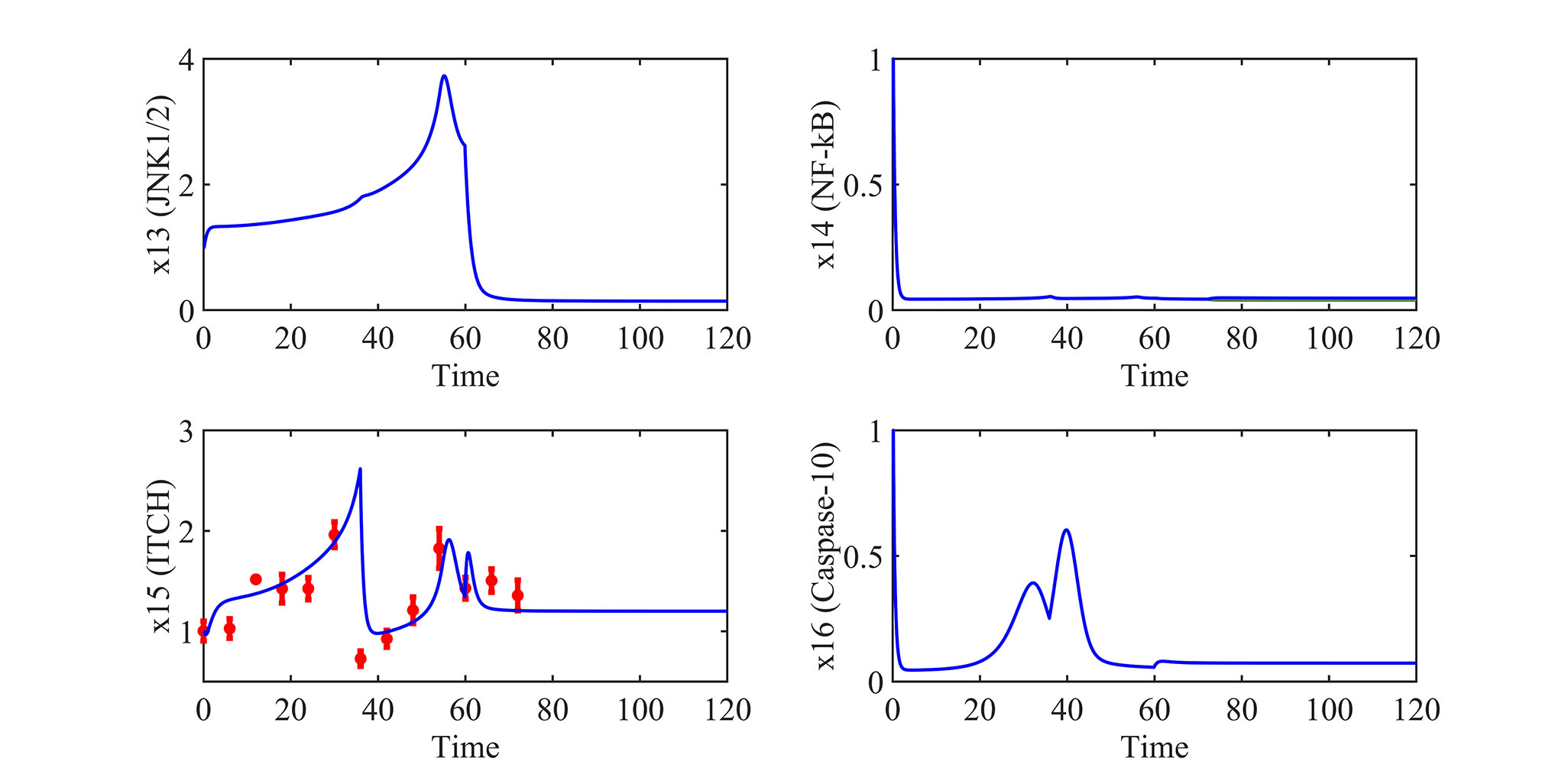

Supplement: Supplementary file 2 [file DataSheet1.zip › Supplementary material_image1/Parameter_a10(大)/4.jpg]

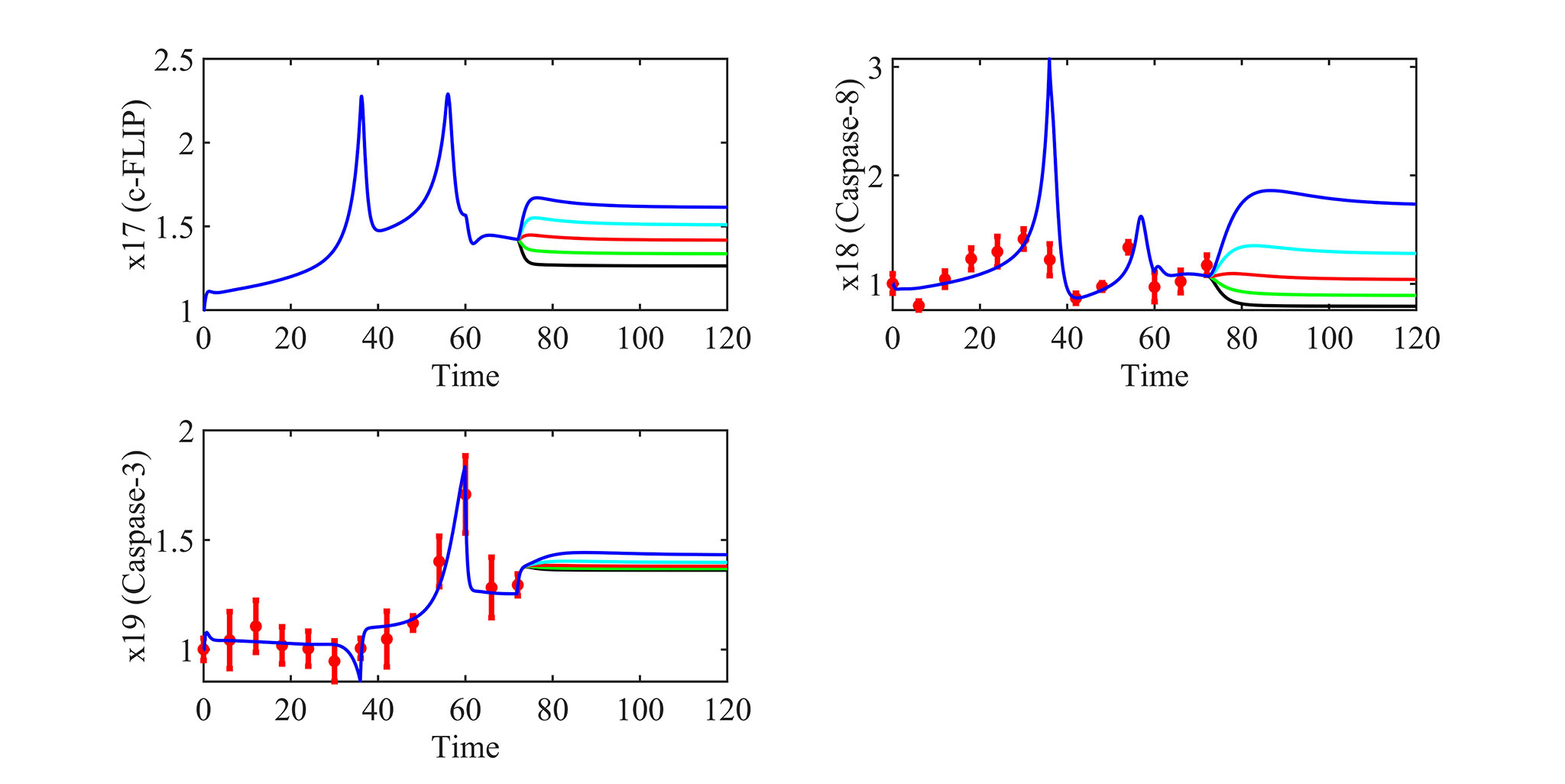

Supplement: Supplementary file 2 [file DataSheet1.zip › Supplementary material_image1/Parameter_a10(大)/5.jpg]

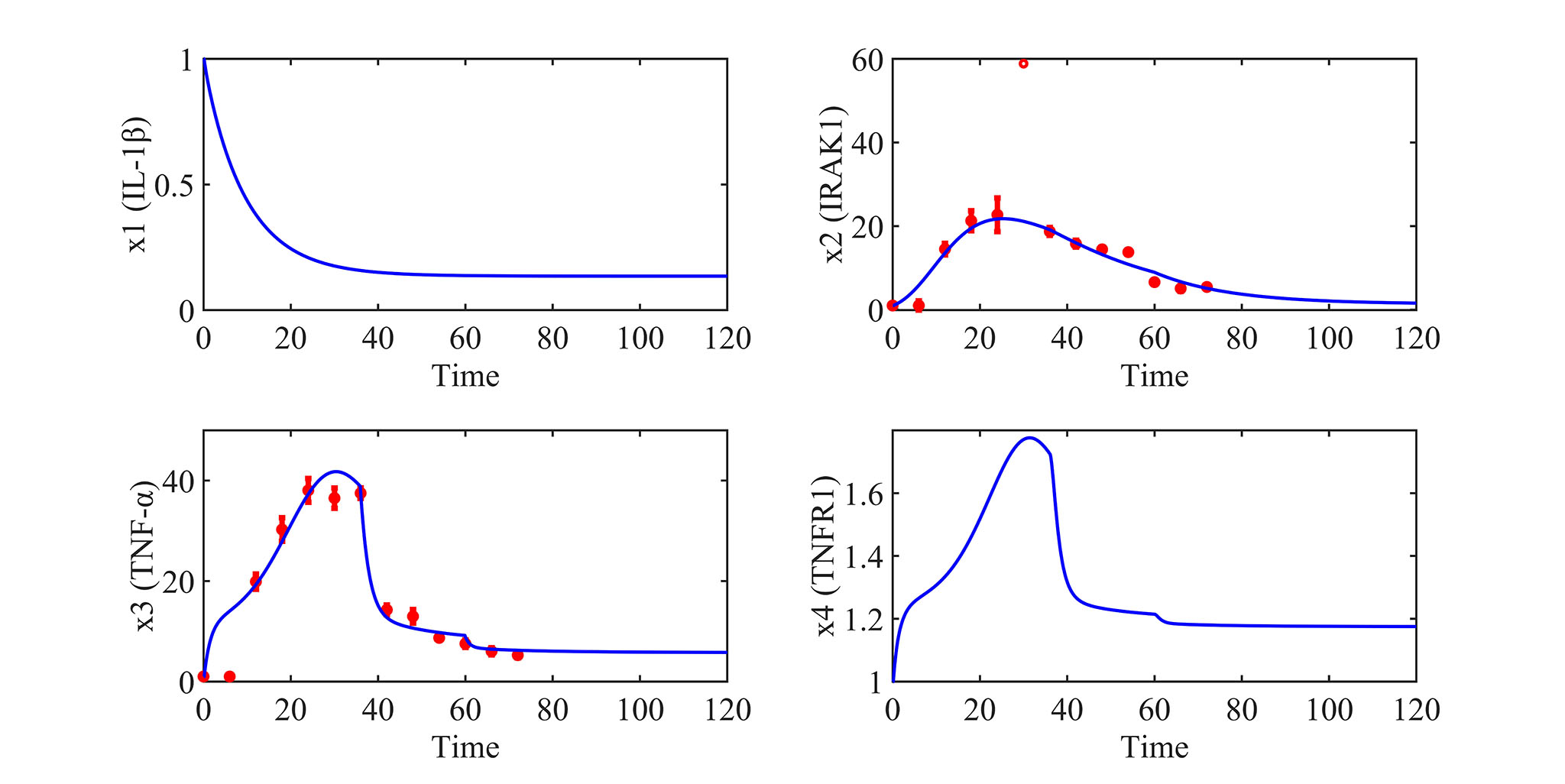

Supplement: Supplementary file 2 [file DataSheet1.zip › Supplementary material_image1/Parameter_a11(小)/1.jpg]

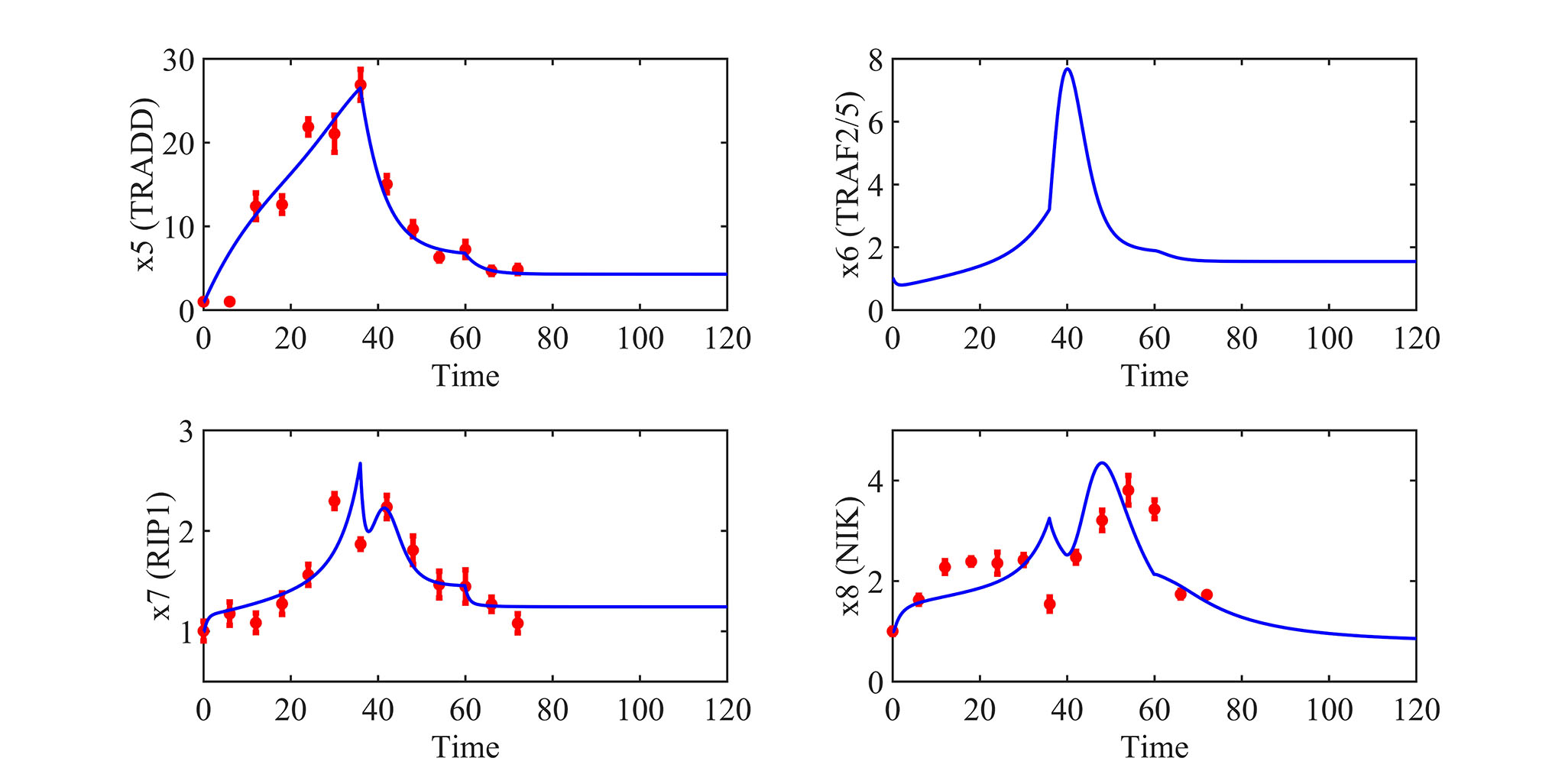

Supplement: Supplementary file 2 [file DataSheet1.zip › Supplementary material_image1/Parameter_a11(小)/2.jpg]

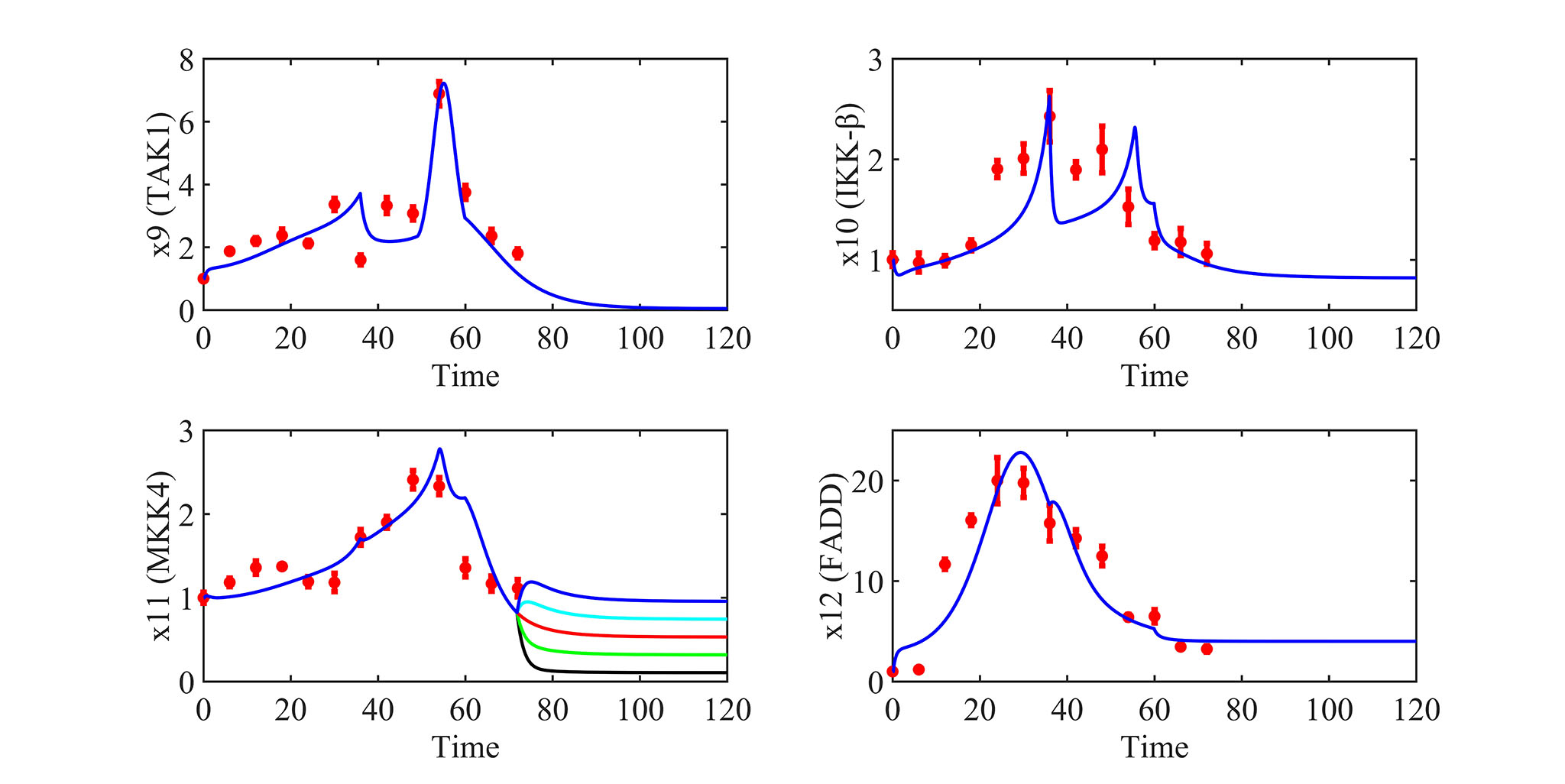

Supplement: Supplementary file 2 [file DataSheet1.zip › Supplementary material_image1/Parameter_a11(小)/3.jpg]

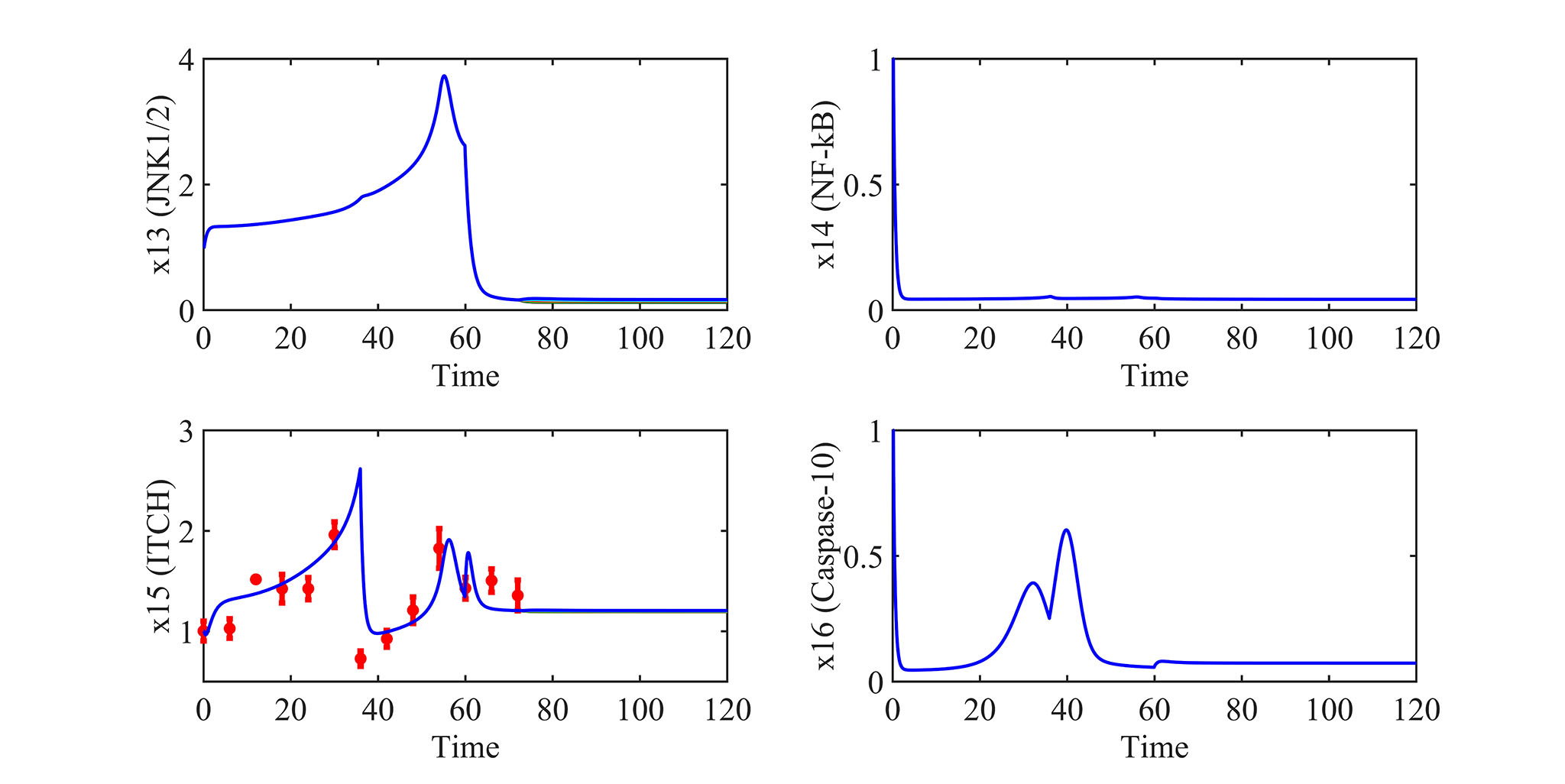

Supplement: Supplementary file 2 [file DataSheet1.zip › Supplementary material_image1/Parameter_a11(小)/4.jpg]

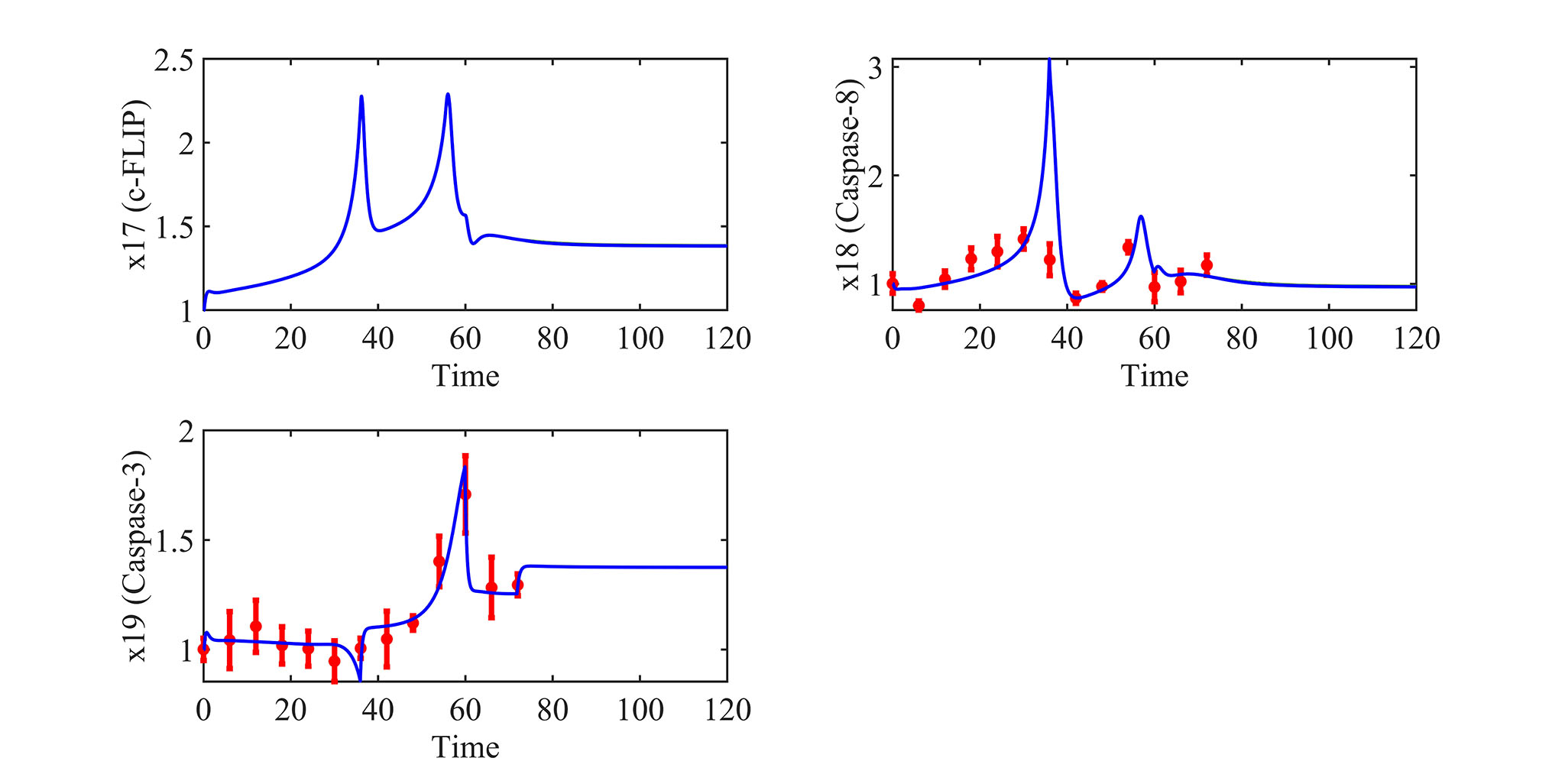

Supplement: Supplementary file 2 [file DataSheet1.zip › Supplementary material_image1/Parameter_a11(小)/5.jpg]

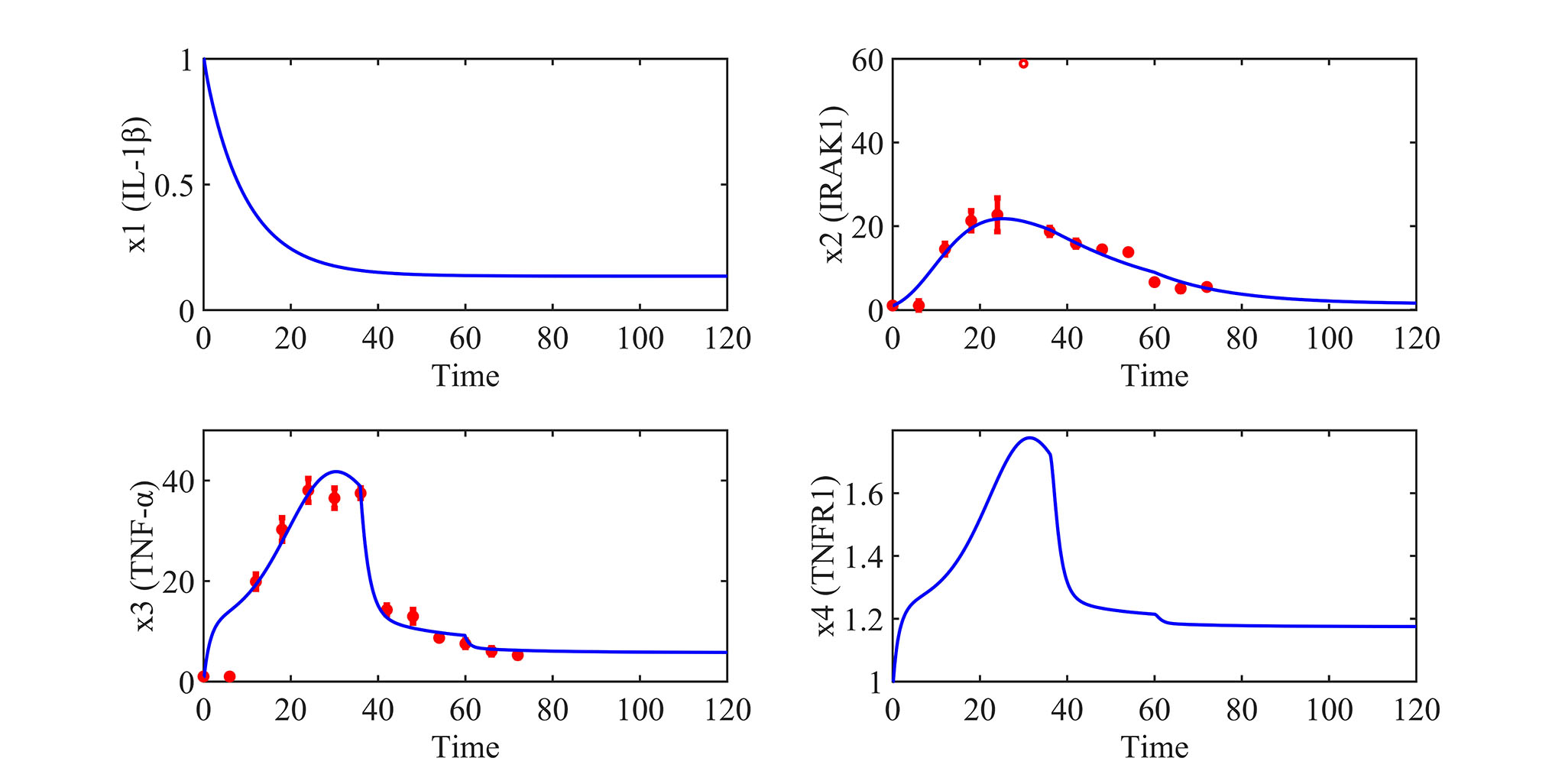

Supplement: Supplementary file 2 [file DataSheet1.zip › Supplementary material_image1/Parameter_a12(中)/1.jpg]

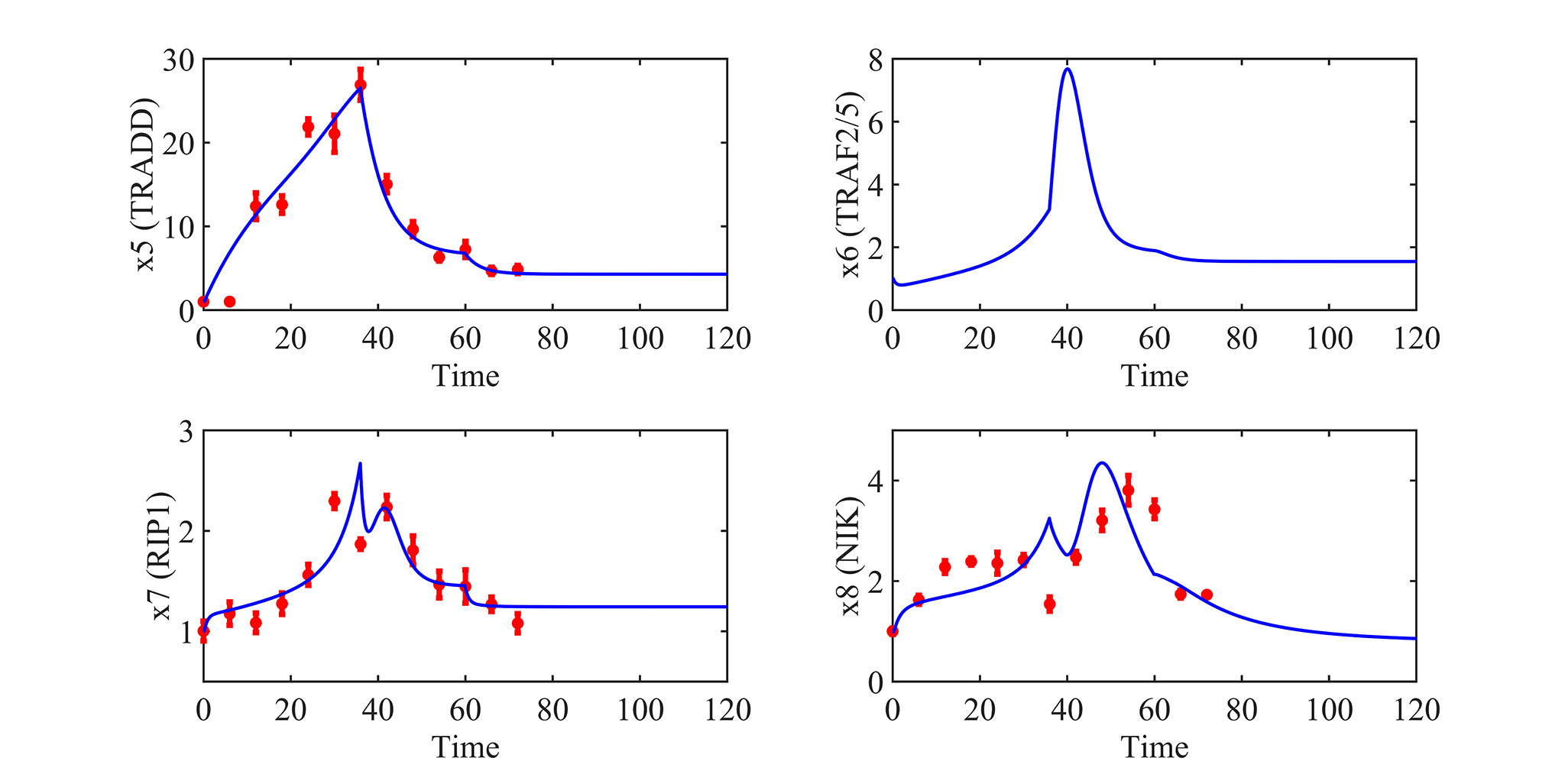

Supplement: Supplementary file 2 [file DataSheet1.zip › Supplementary material_image1/Parameter_a12(中)/2.jpg]

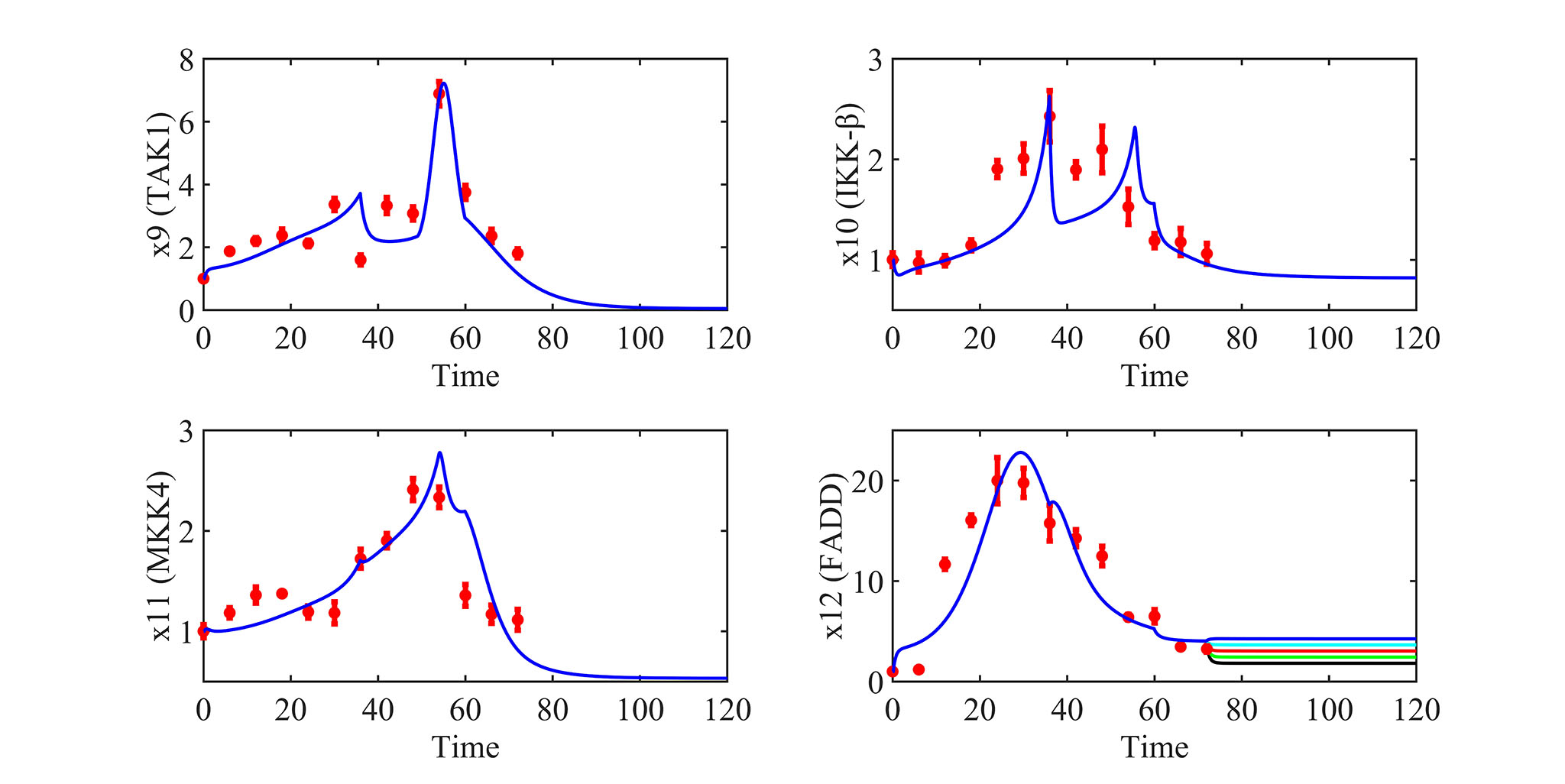

Supplement: Supplementary file 2 [file DataSheet1.zip › Supplementary material_image1/Parameter_a12(中)/3.jpg]

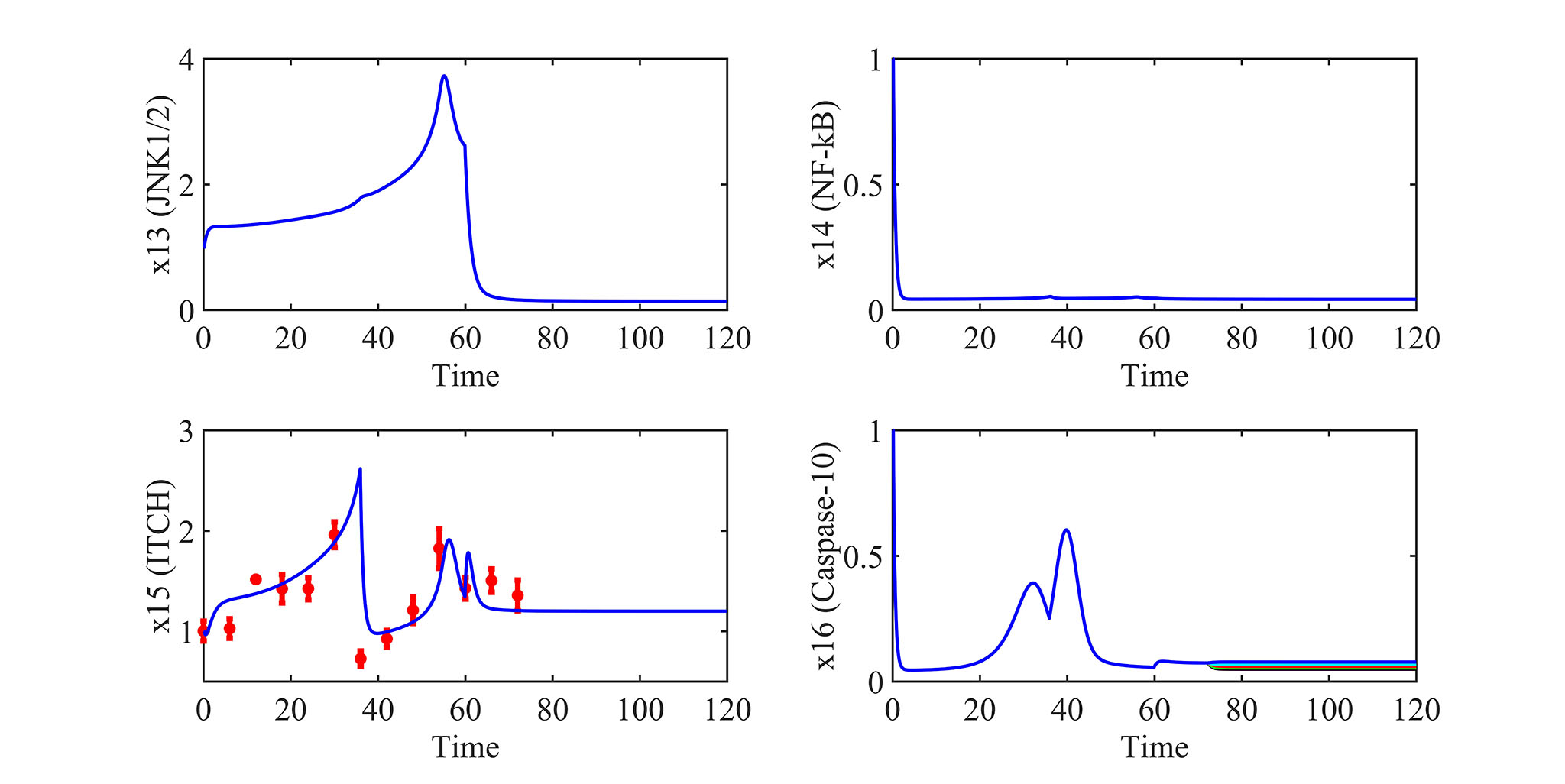

Supplement: Supplementary file 2 [file DataSheet1.zip › Supplementary material_image1/Parameter_a12(中)/4.jpg]

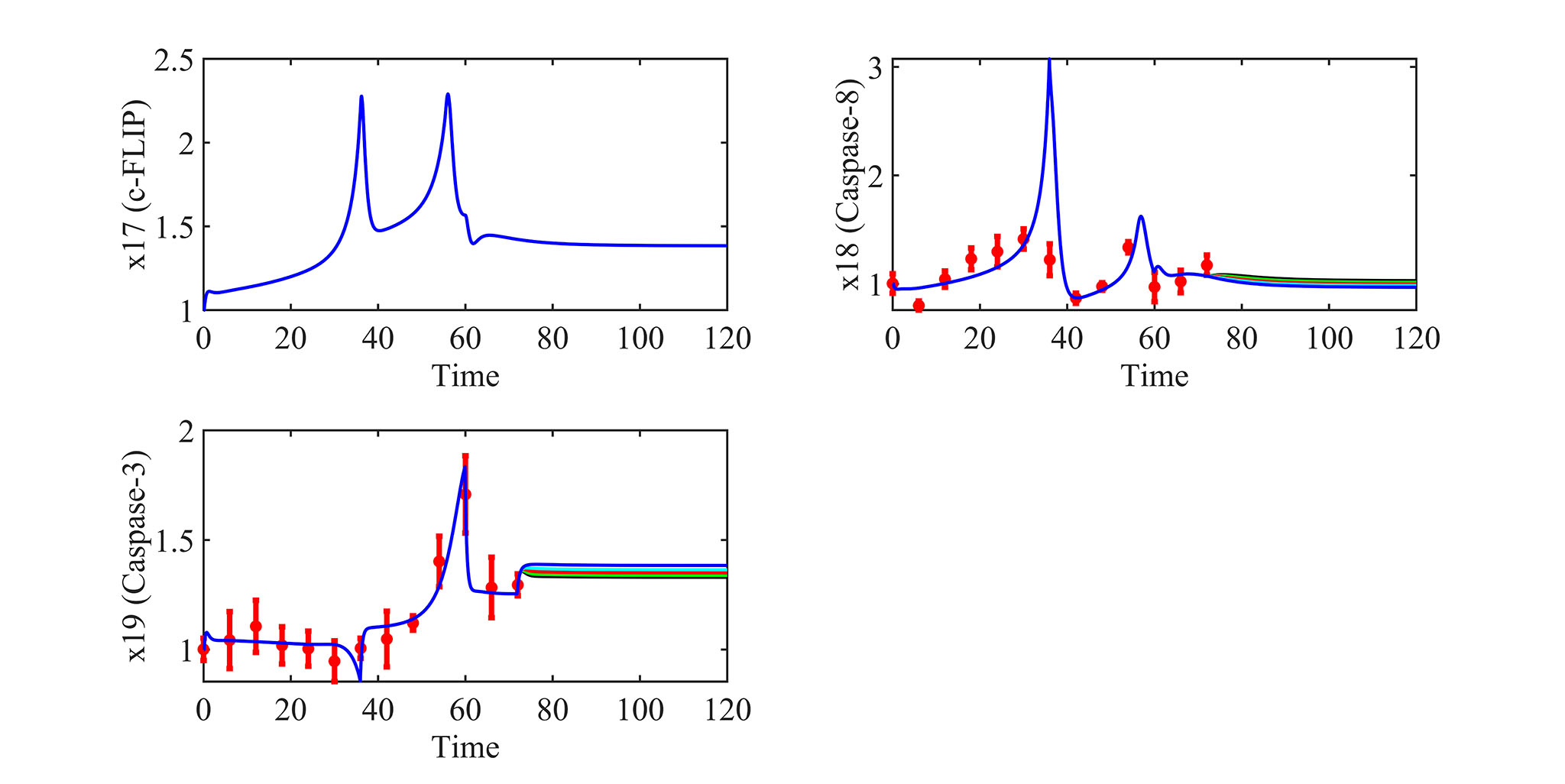

Supplement: Supplementary file 2 [file DataSheet1.zip › Supplementary material_image1/Parameter_a12(中)/5.jpg]

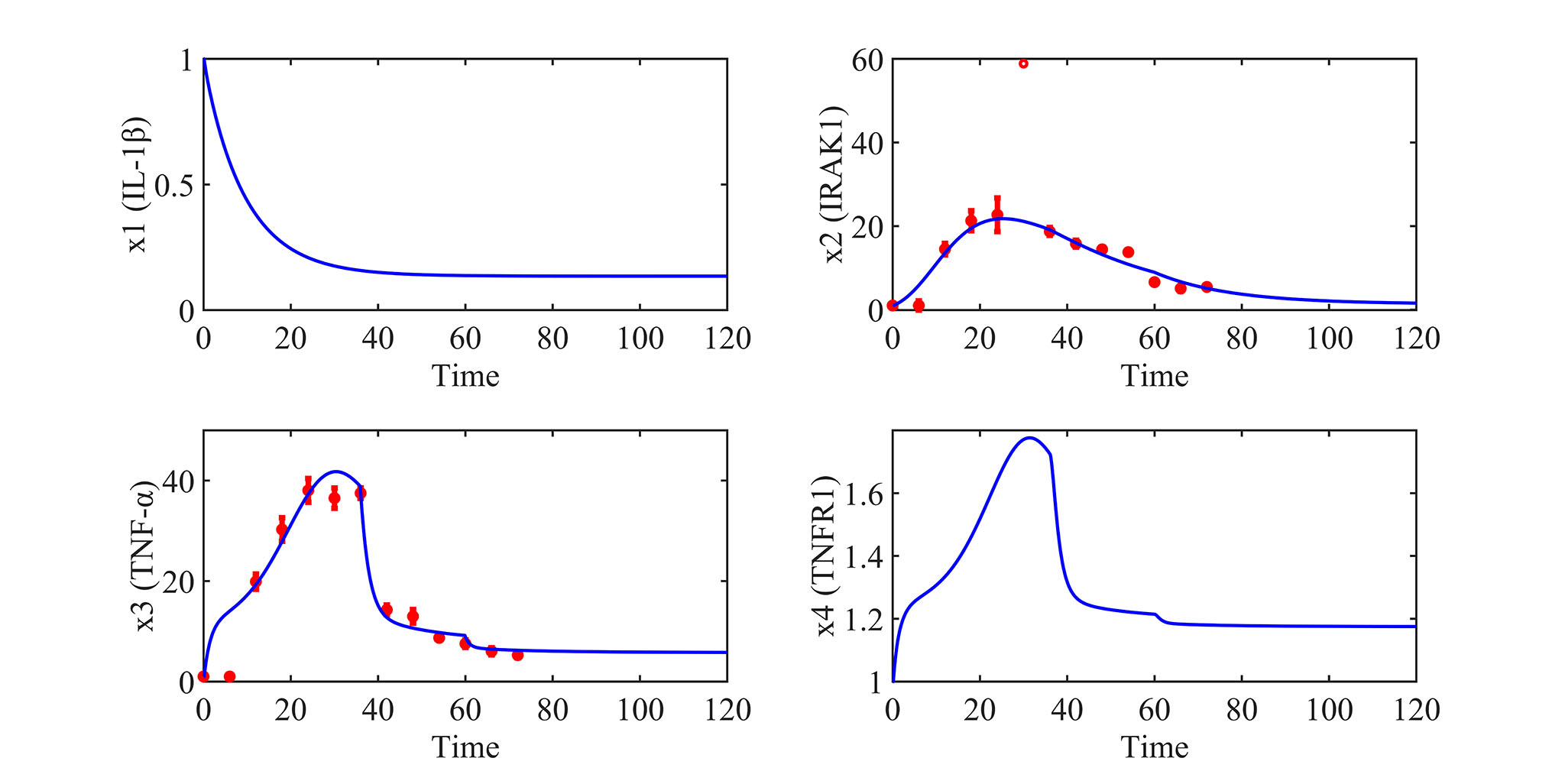

Supplement: Supplementary file 2 [file DataSheet1.zip › Supplementary material_image1/Parameter_a13(小)/1.jpg]

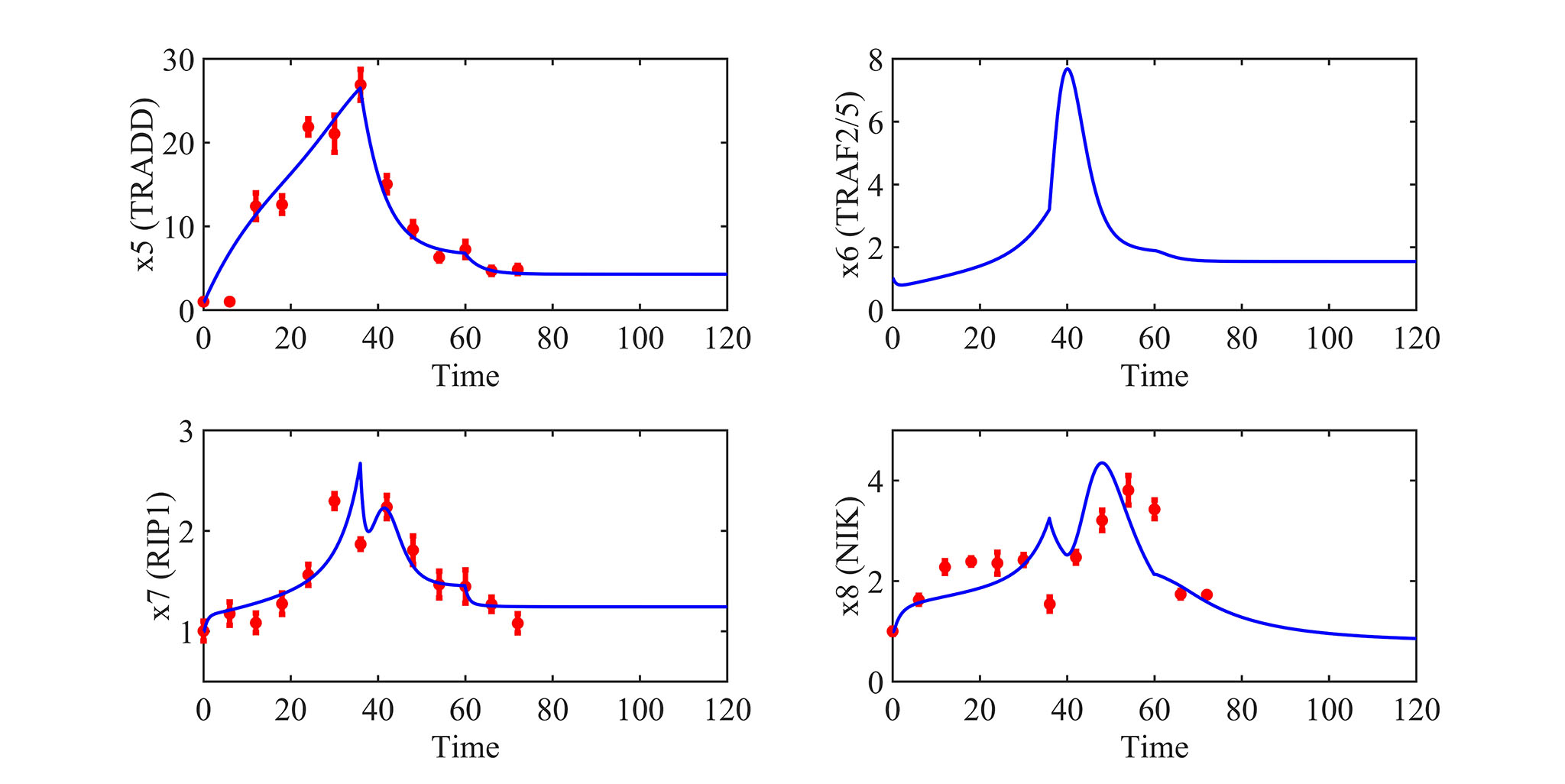

Supplement: Supplementary file 2 [file DataSheet1.zip › Supplementary material_image1/Parameter_a13(小)/2.jpg]

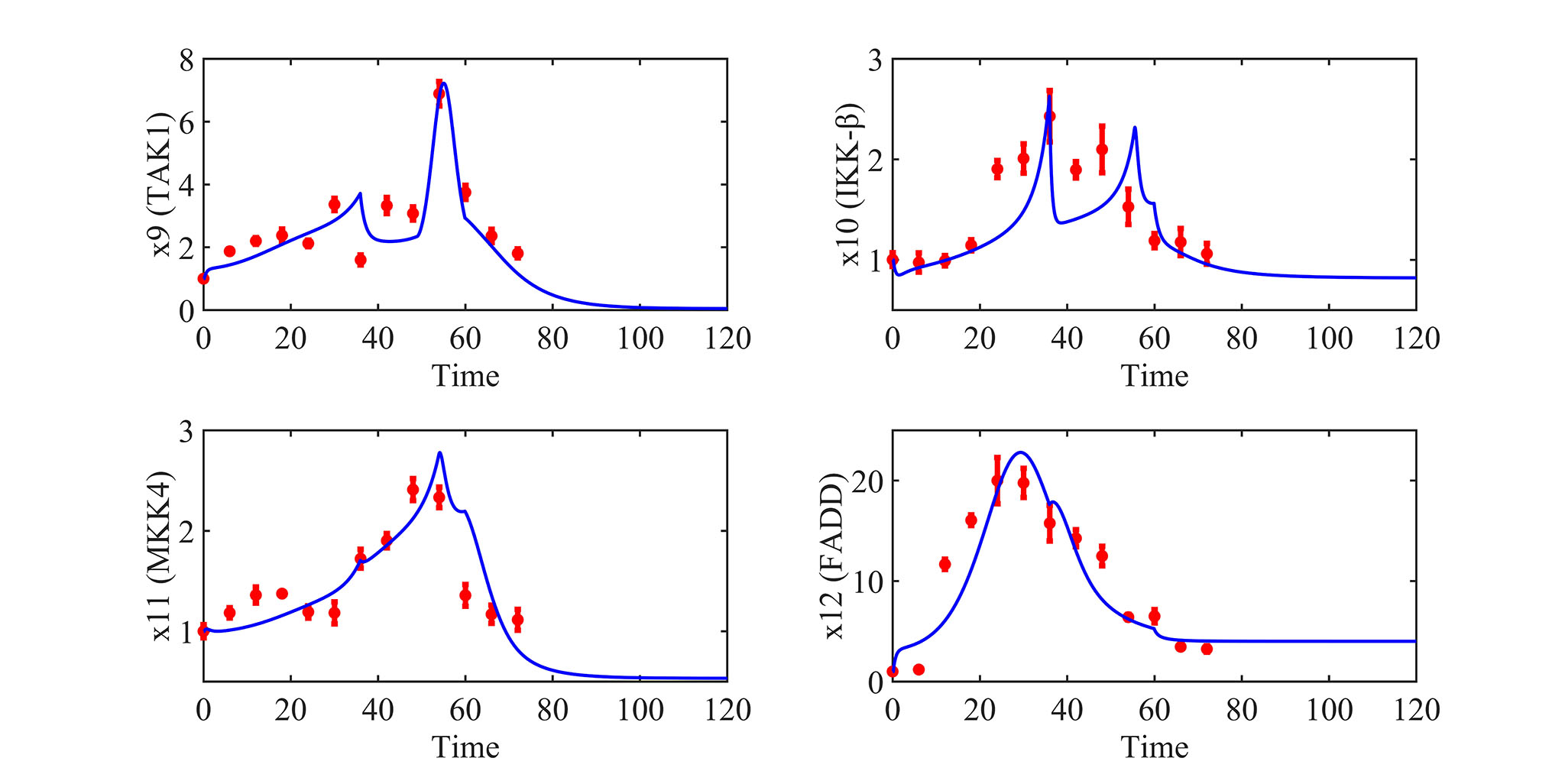

Supplement: Supplementary file 2 [file DataSheet1.zip › Supplementary material_image1/Parameter_a13(小)/3.jpg]

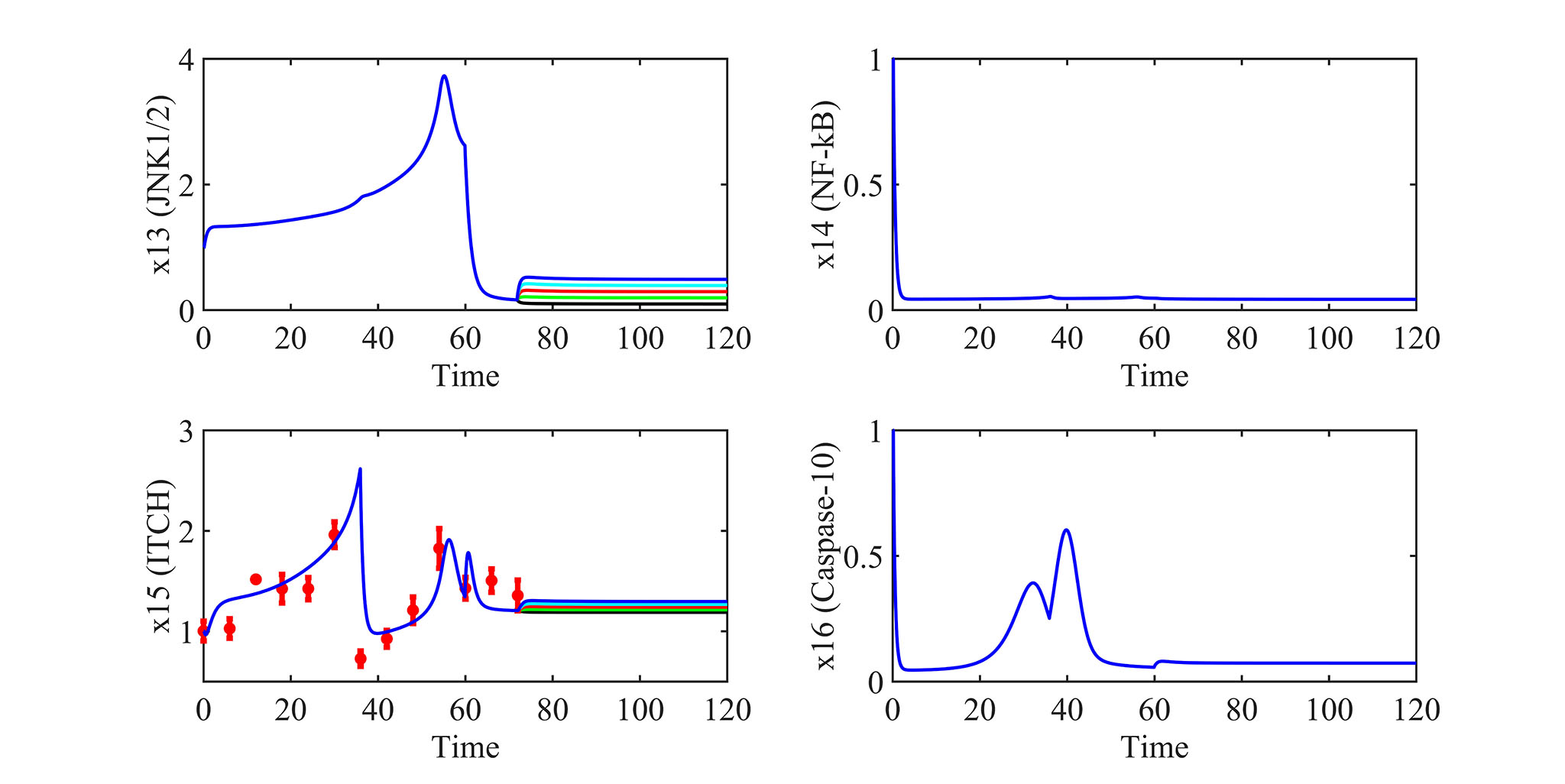

Supplement: Supplementary file 2 [file DataSheet1.zip › Supplementary material_image1/Parameter_a13(小)/4.jpg]

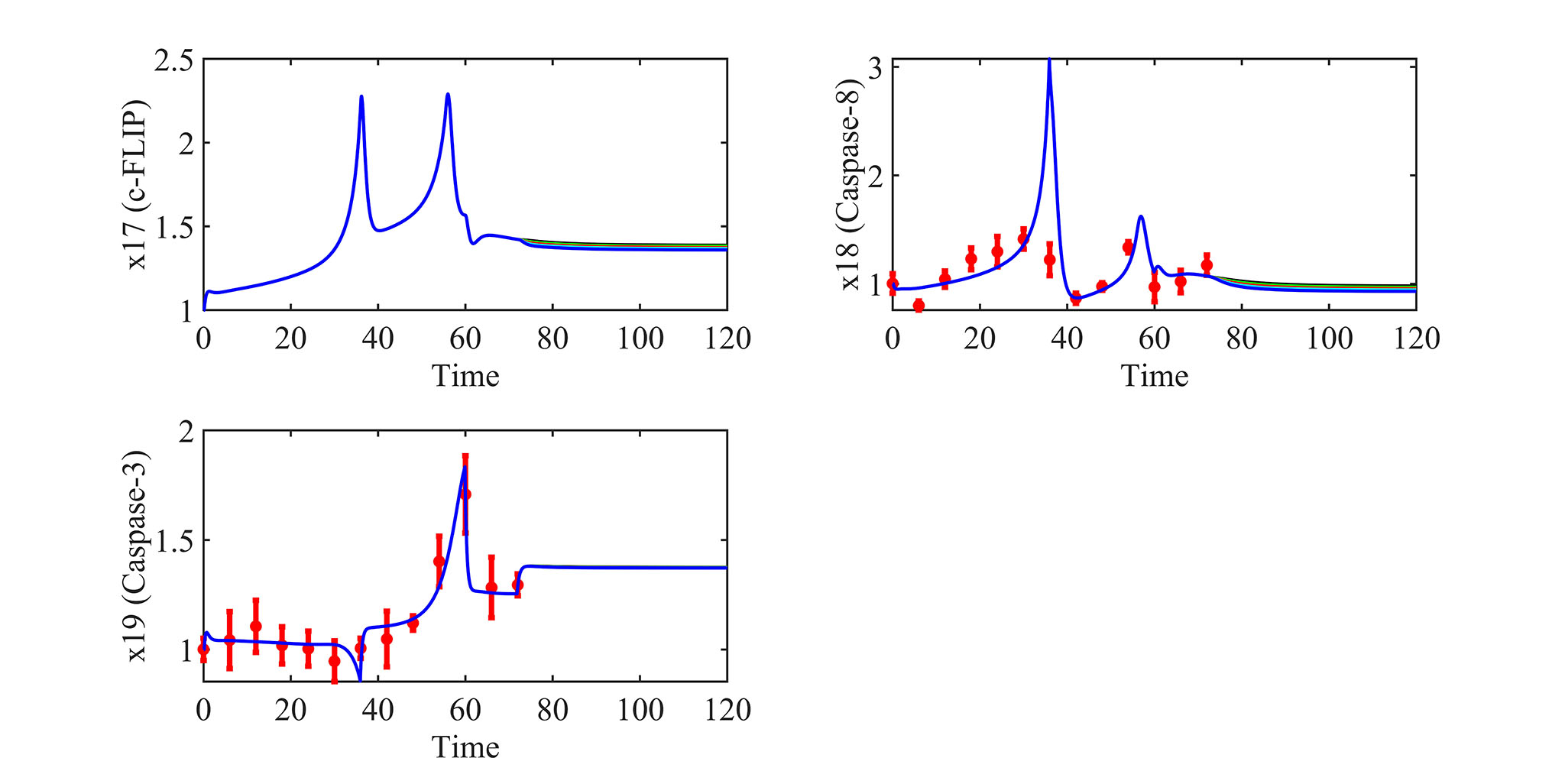

Supplement: Supplementary file 2 [file DataSheet1.zip › Supplementary material_image1/Parameter_a13(小)/5.jpg]

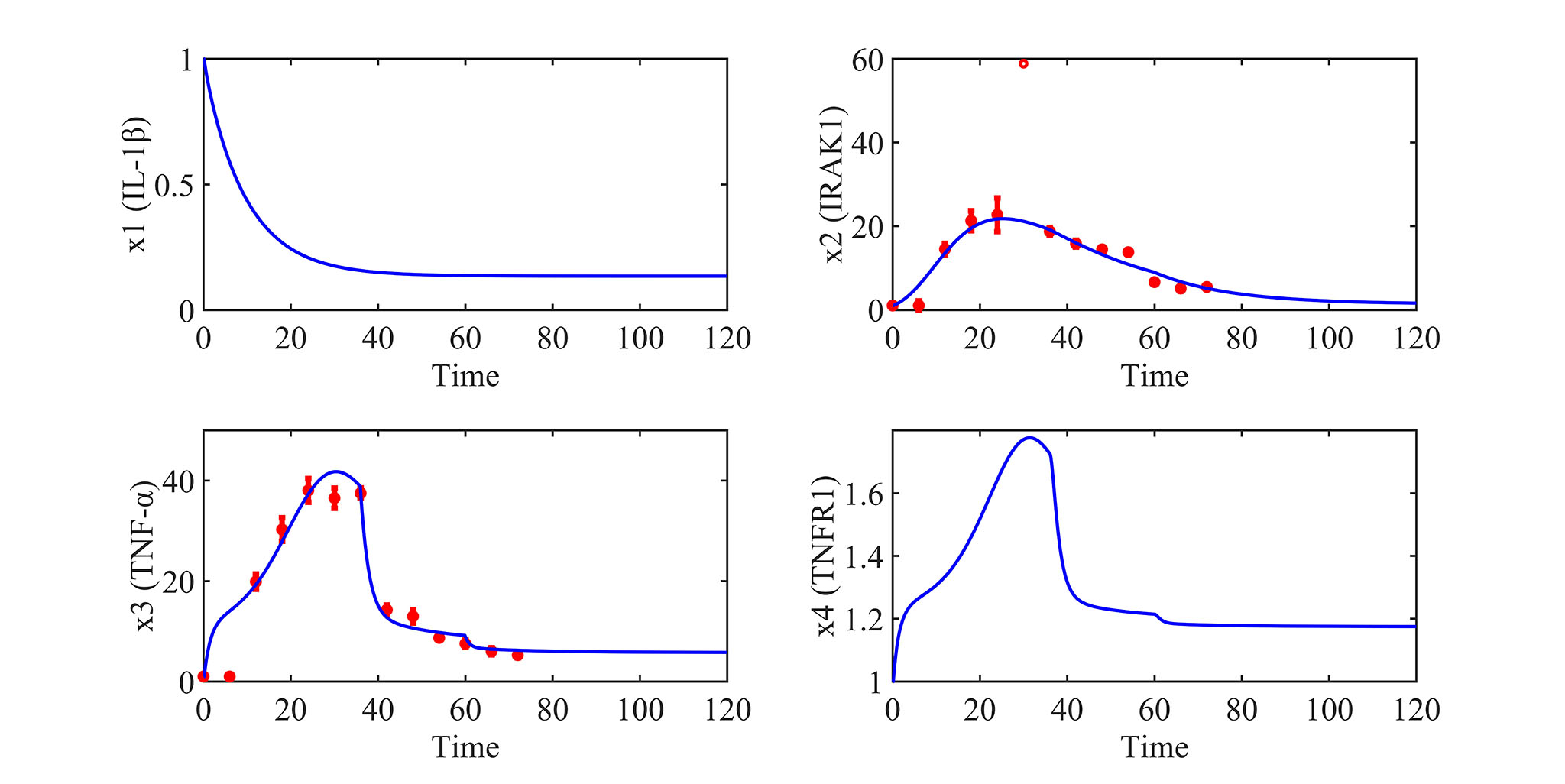

Supplement: Supplementary file 2 [file DataSheet1.zip › Supplementary material_image1/Parameter_a14(小)/1.jpg]

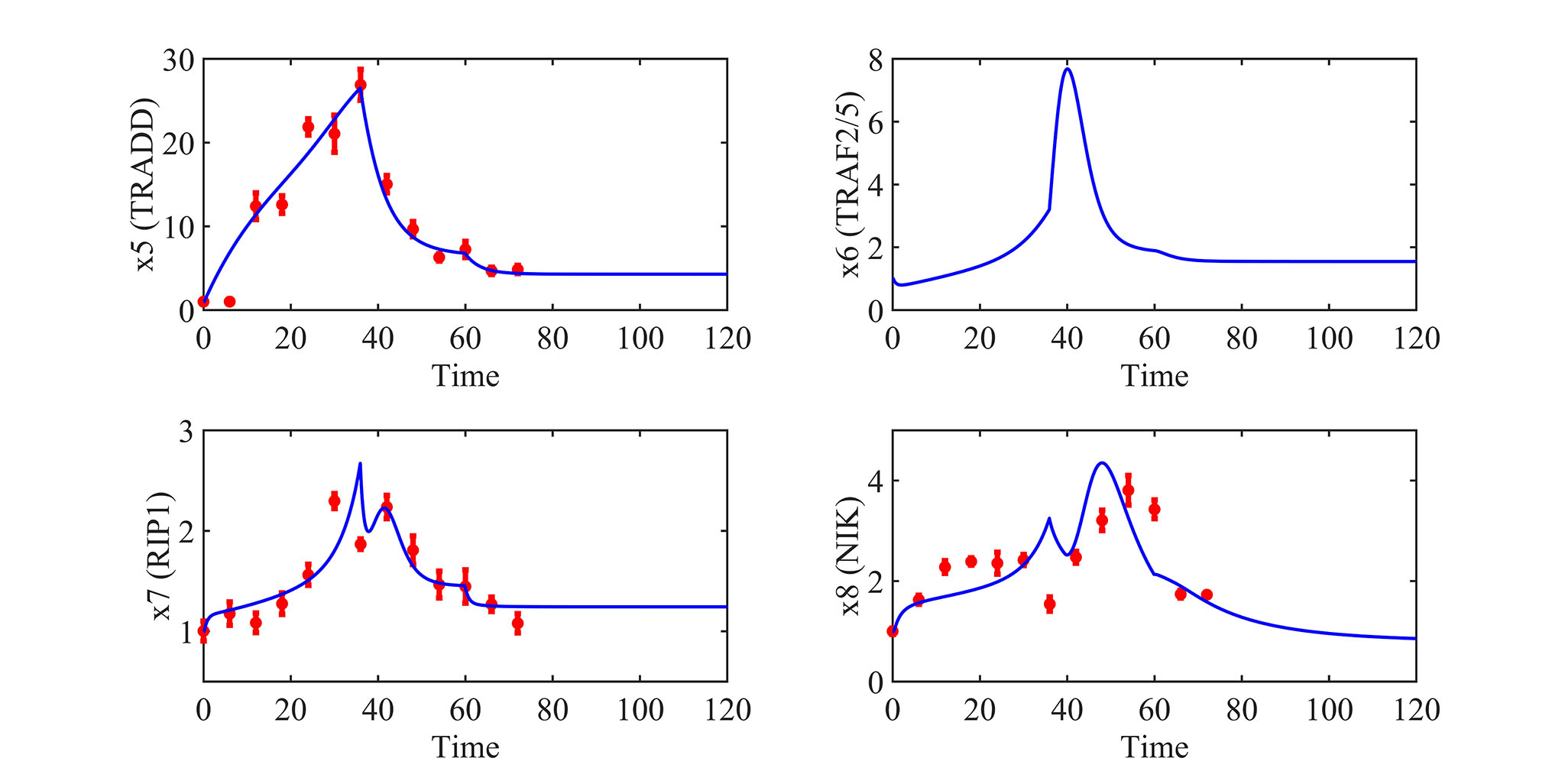

Supplement: Supplementary file 2 [file DataSheet1.zip › Supplementary material_image1/Parameter_a14(小)/2.jpg]

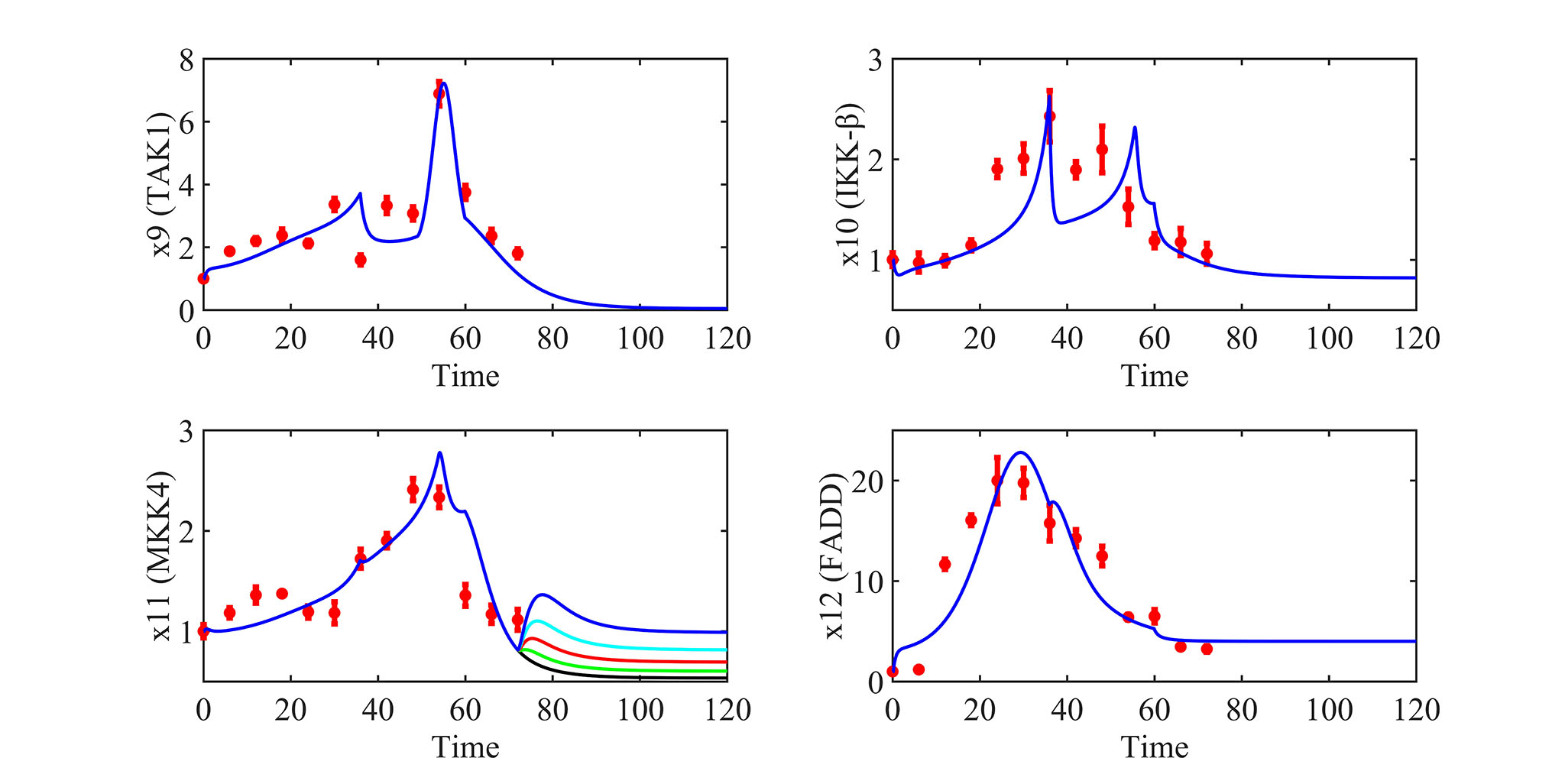

Supplement: Supplementary file 2 [file DataSheet1.zip › Supplementary material_image1/Parameter_a14(小)/3.jpg]

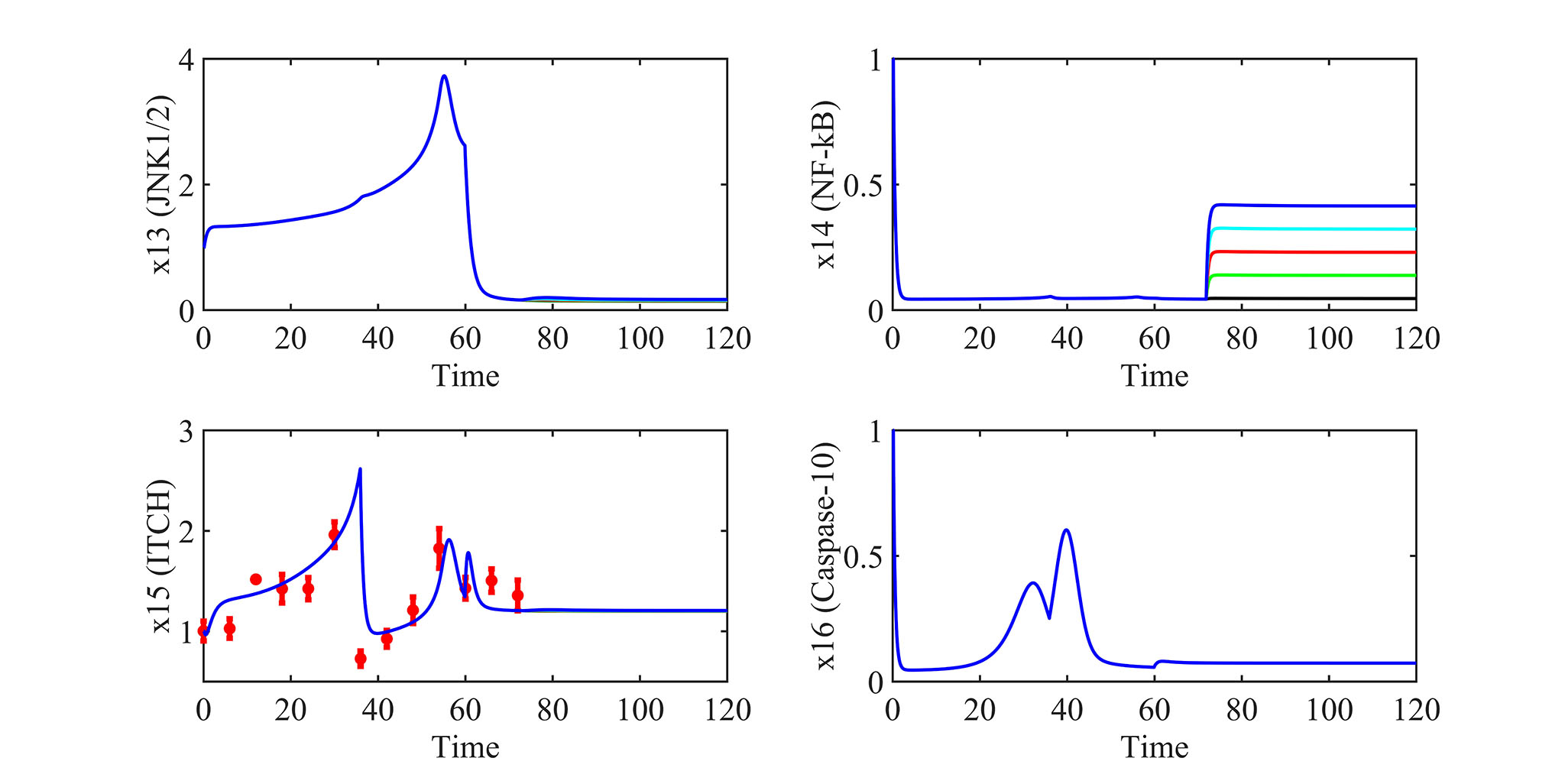

Supplement: Supplementary file 2 [file DataSheet1.zip › Supplementary material_image1/Parameter_a14(小)/4.jpg]

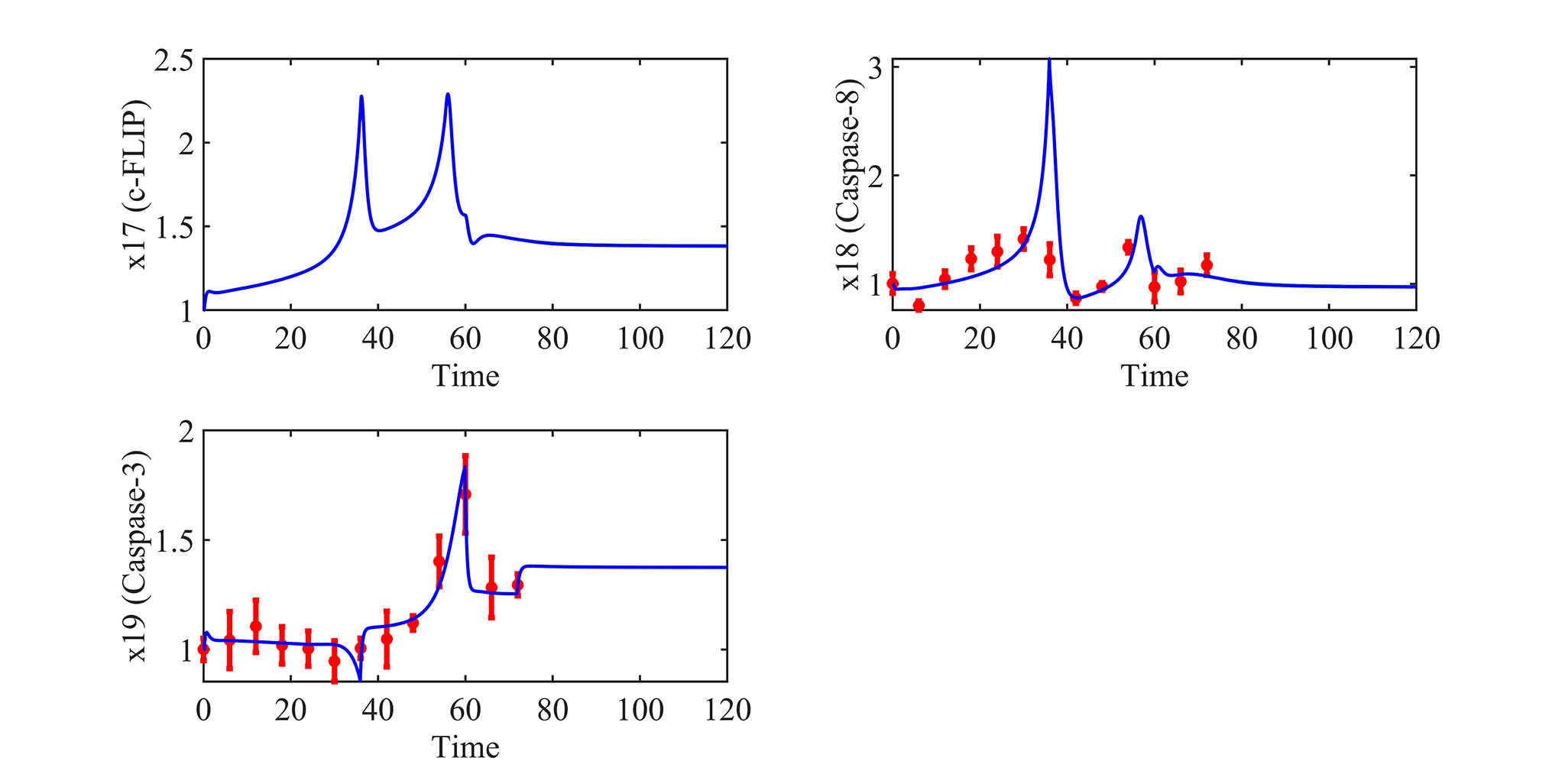

Supplement: Supplementary file 2 [file DataSheet1.zip › Supplementary material_image1/Parameter_a14(小)/5.jpg]

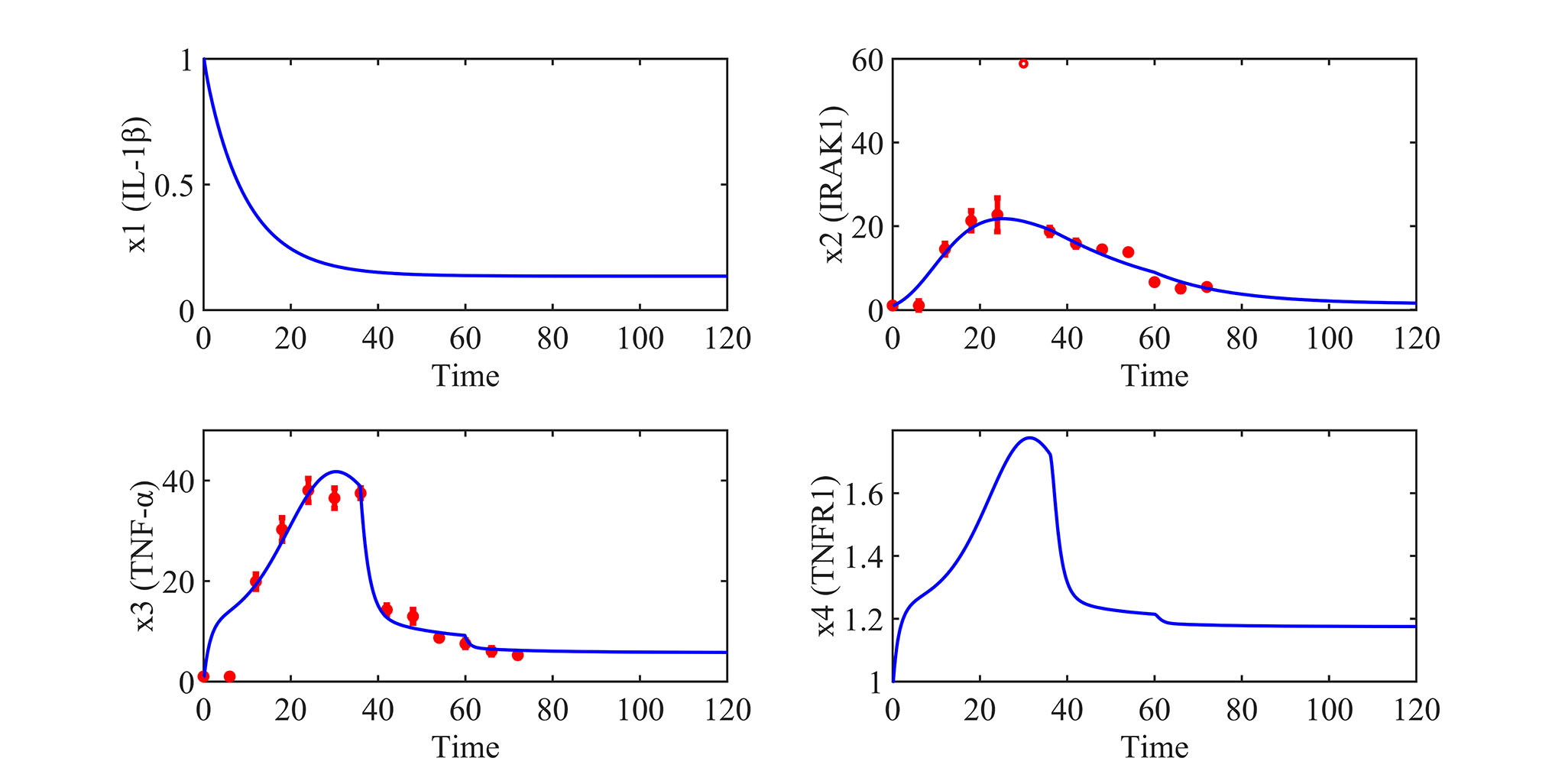

Supplement: Supplementary file 2 [file DataSheet1.zip › Supplementary material_image1/Parameter_a15(小)/1.jpg]

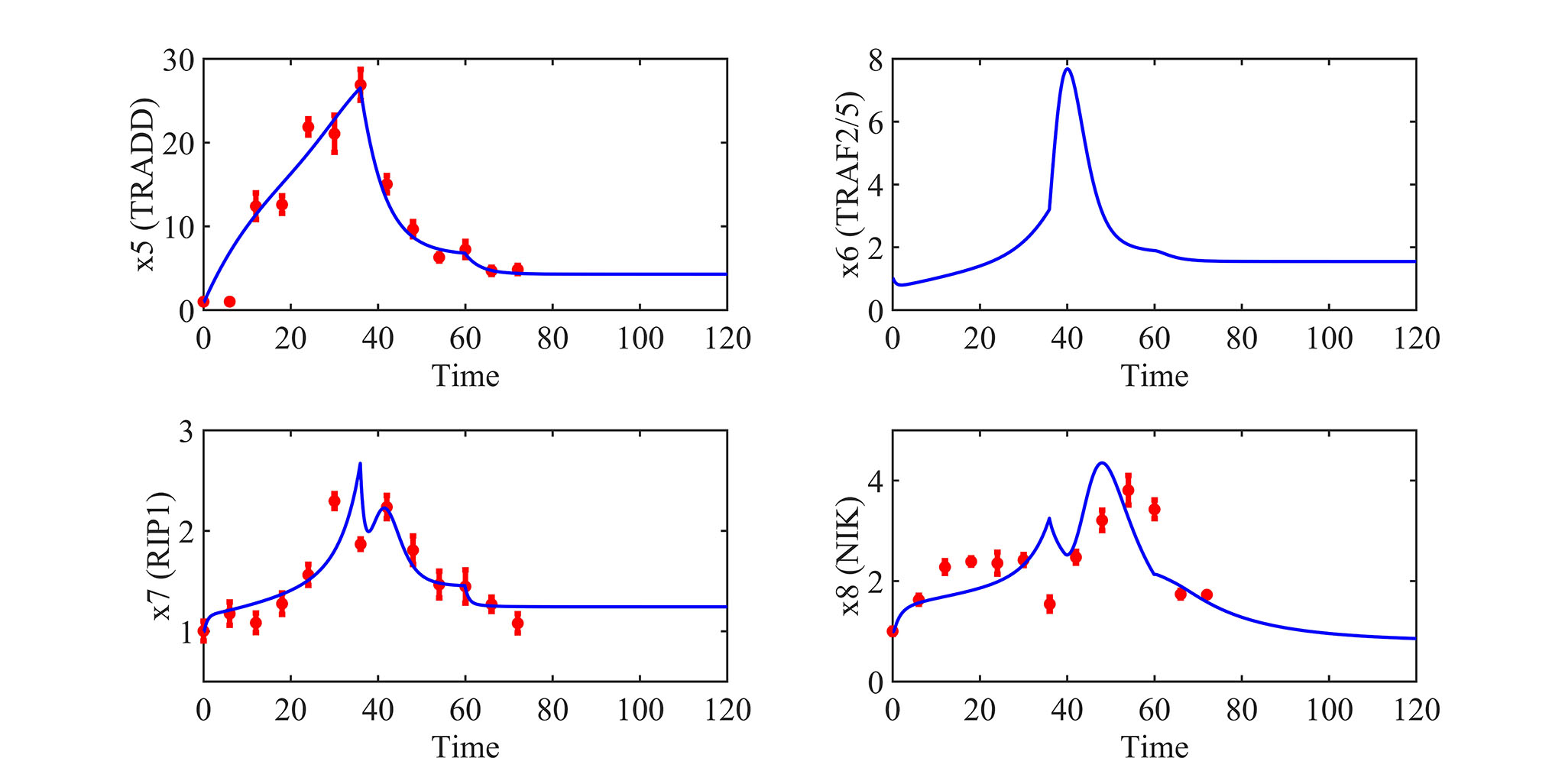

Supplement: Supplementary file 2 [file DataSheet1.zip › Supplementary material_image1/Parameter_a15(小)/2.jpg]

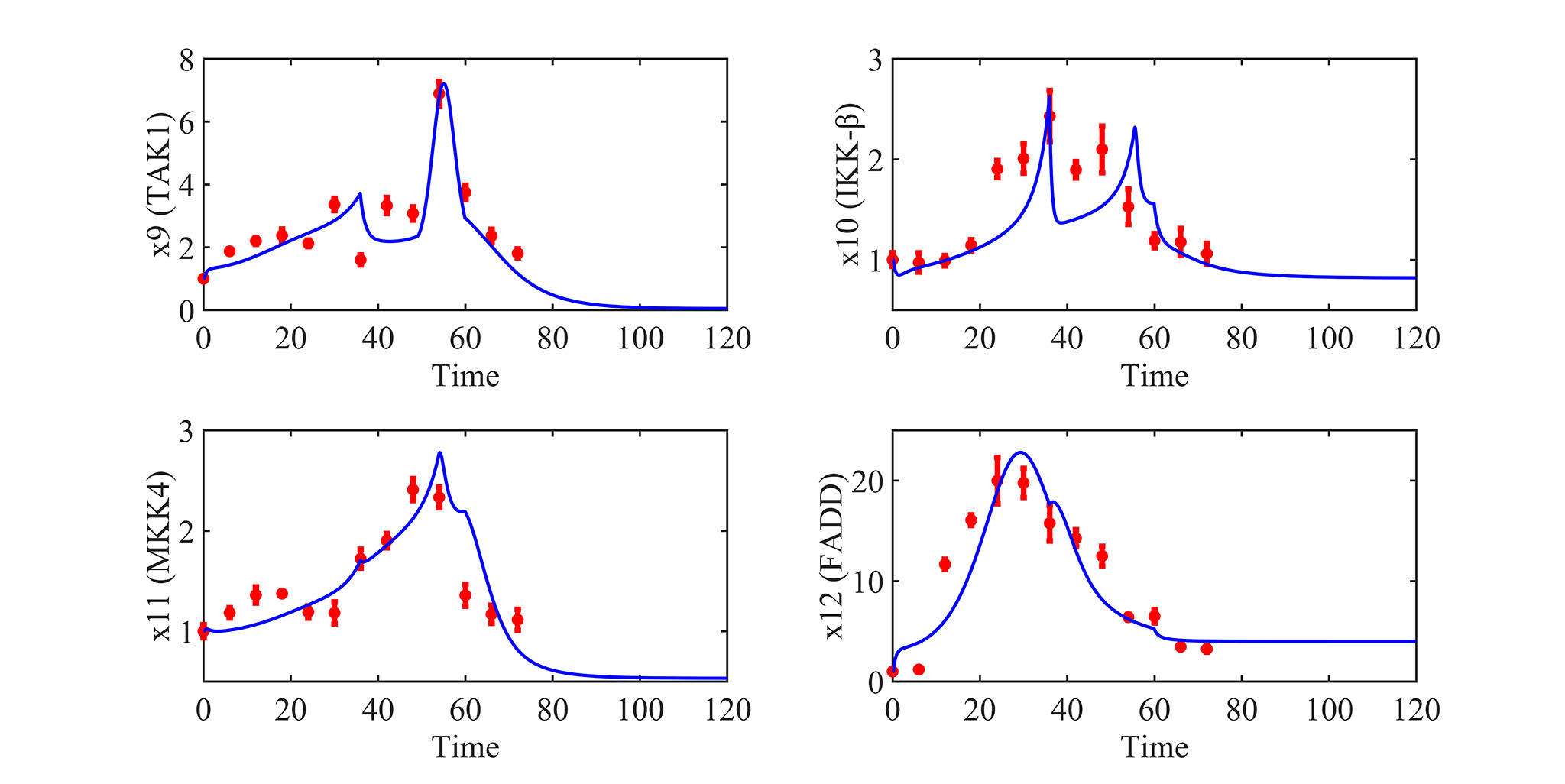

Supplement: Supplementary file 2 [file DataSheet1.zip › Supplementary material_image1/Parameter_a15(小)/3.jpg]

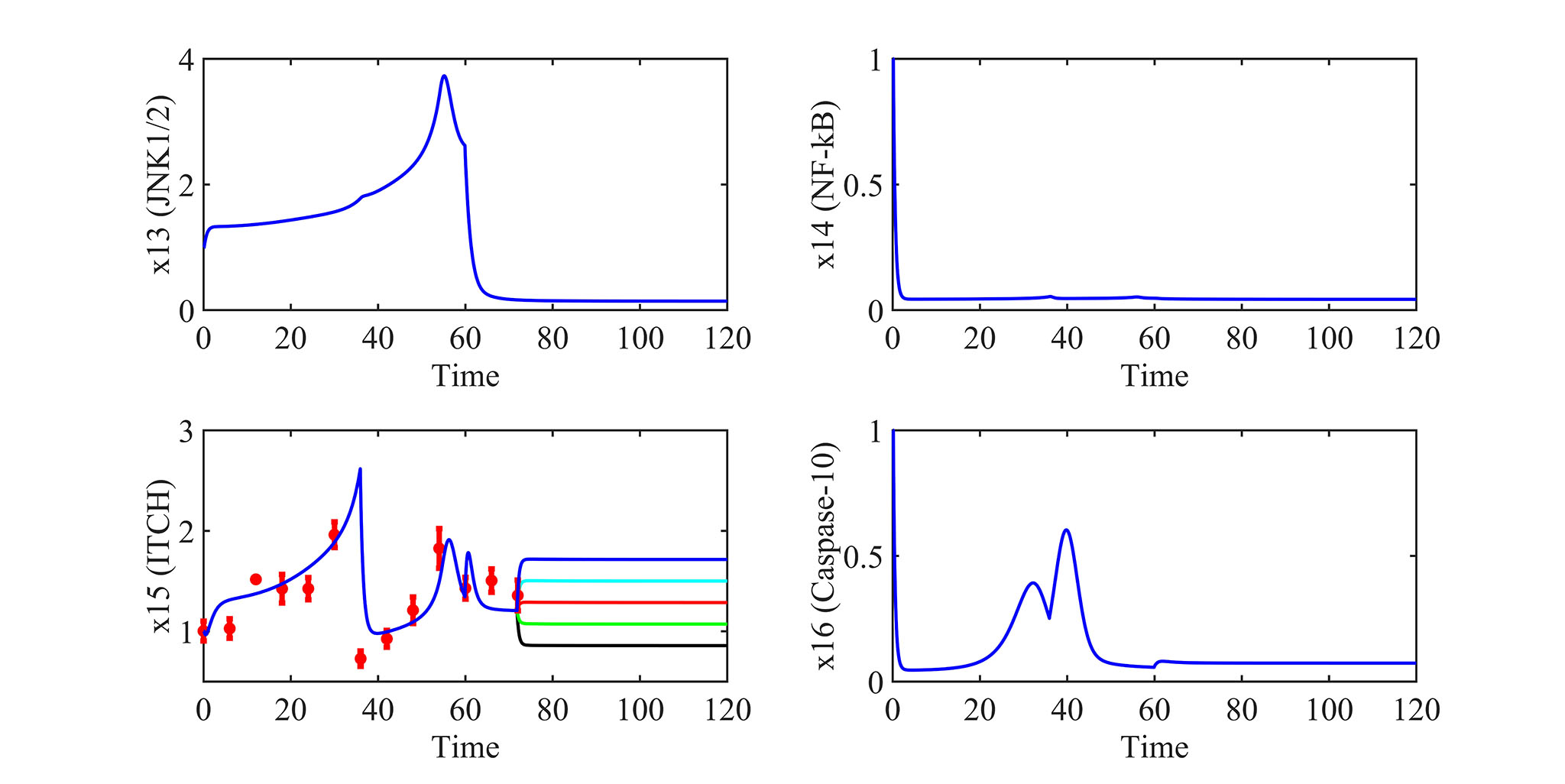

Supplement: Supplementary file 2 [file DataSheet1.zip › Supplementary material_image1/Parameter_a15(小)/4.jpg]

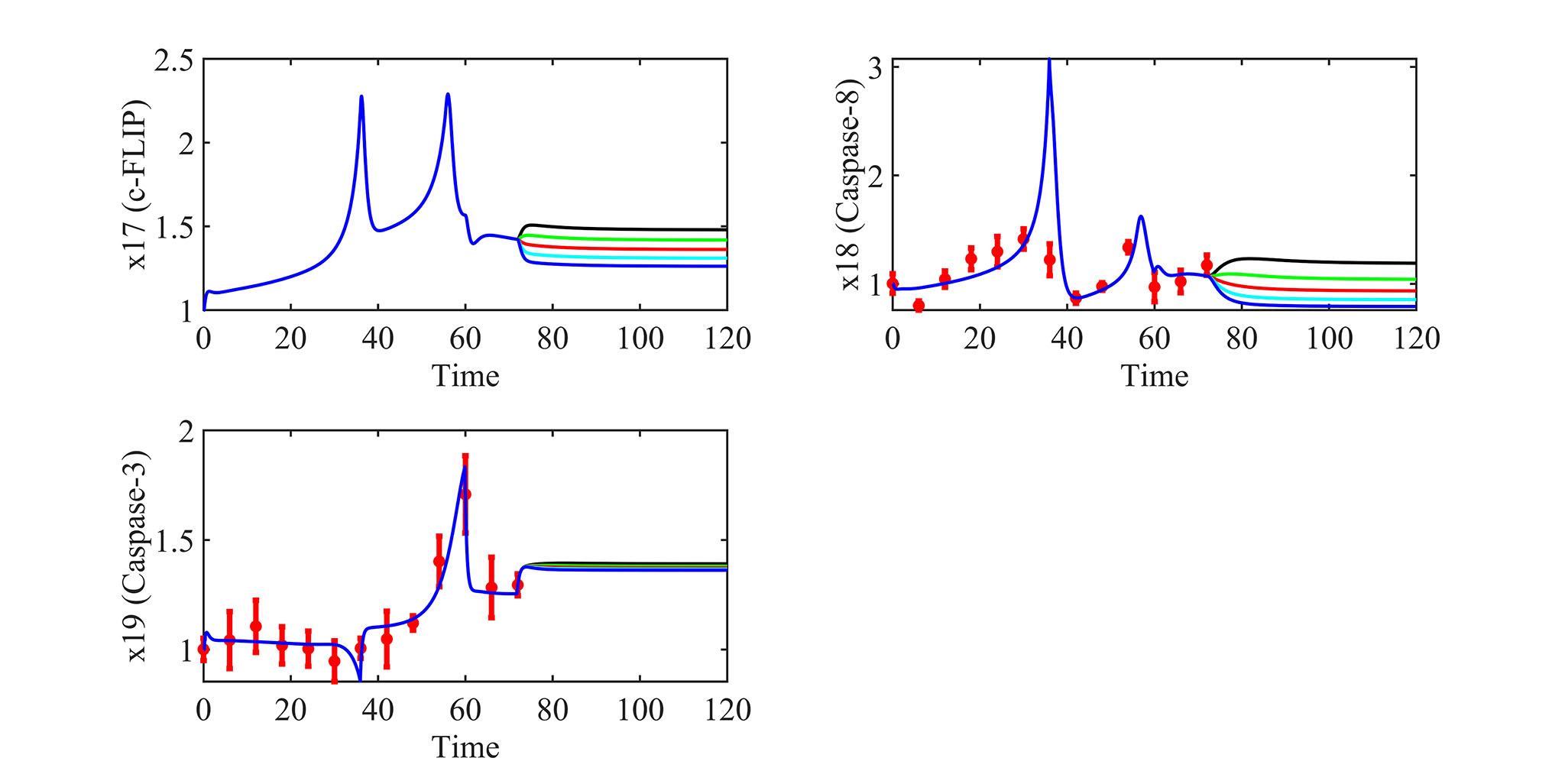

Supplement: Supplementary file 2 [file DataSheet1.zip › Supplementary material_image1/Parameter_a15(小)/5.jpg]

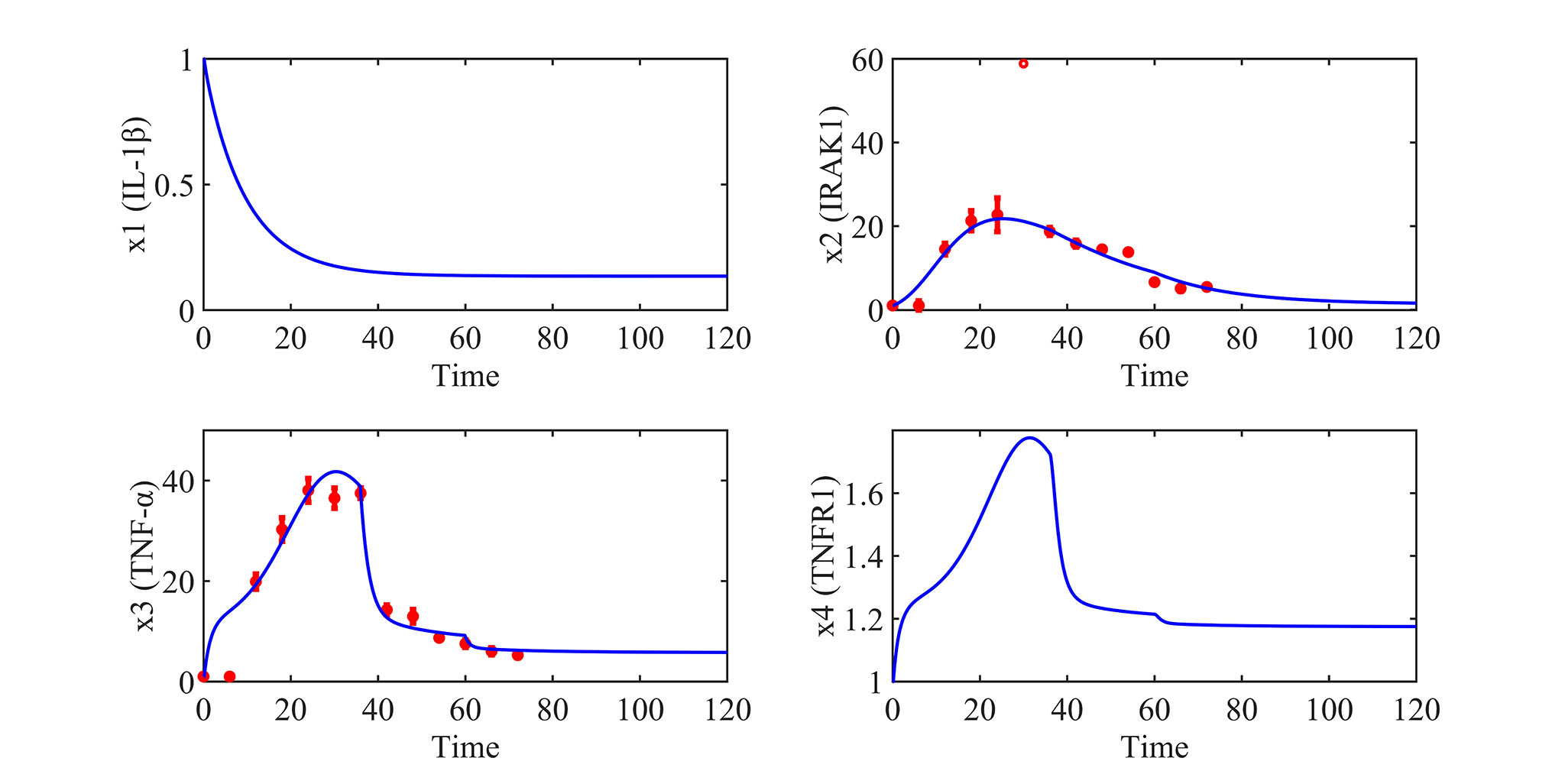

Supplement: Supplementary file 2 [file DataSheet1.zip › Supplementary material_image1/Parameter_a16(大)/1.jpg]

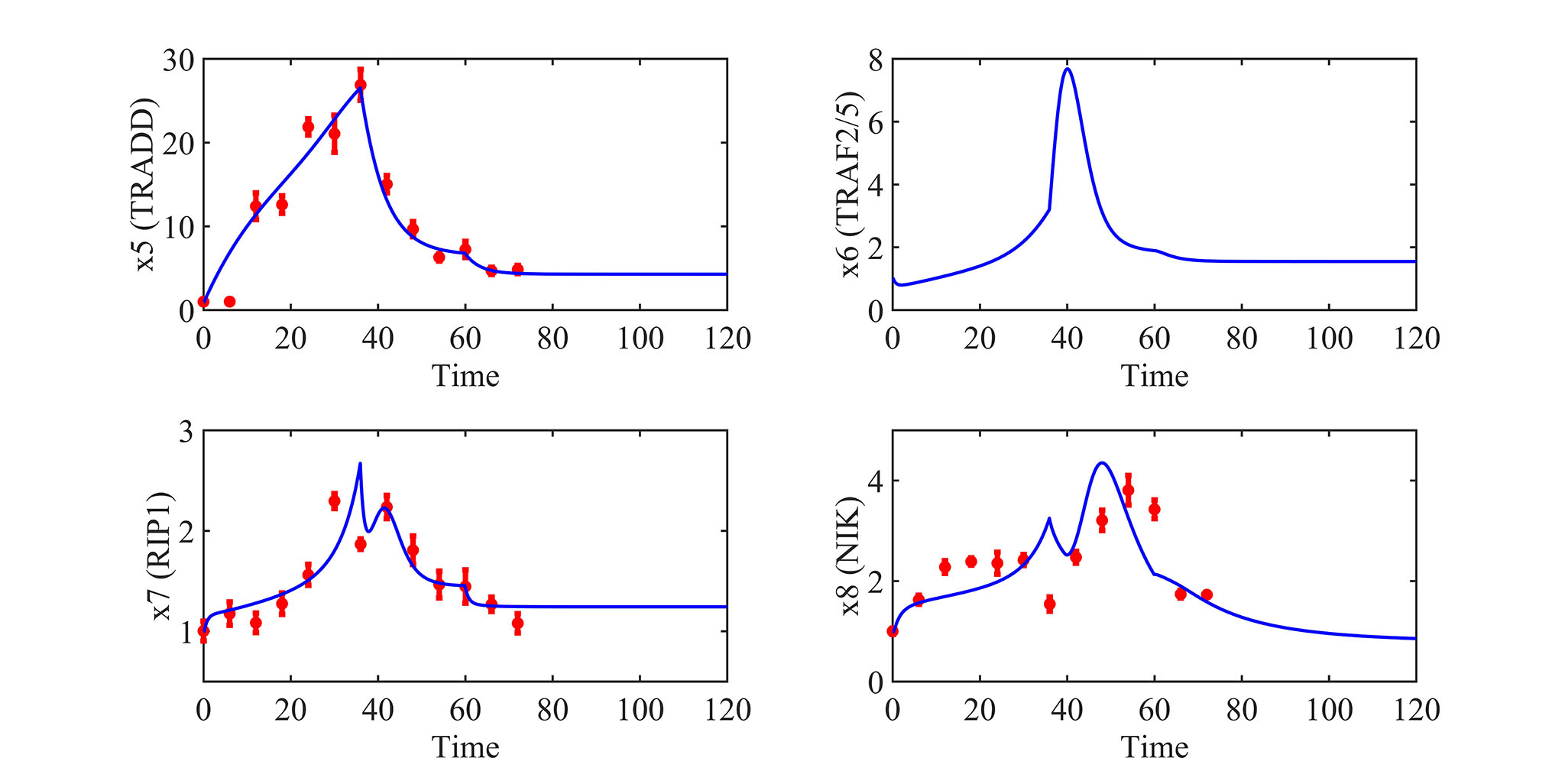

Supplement: Supplementary file 2 [file DataSheet1.zip › Supplementary material_image1/Parameter_a16(大)/2.jpg]

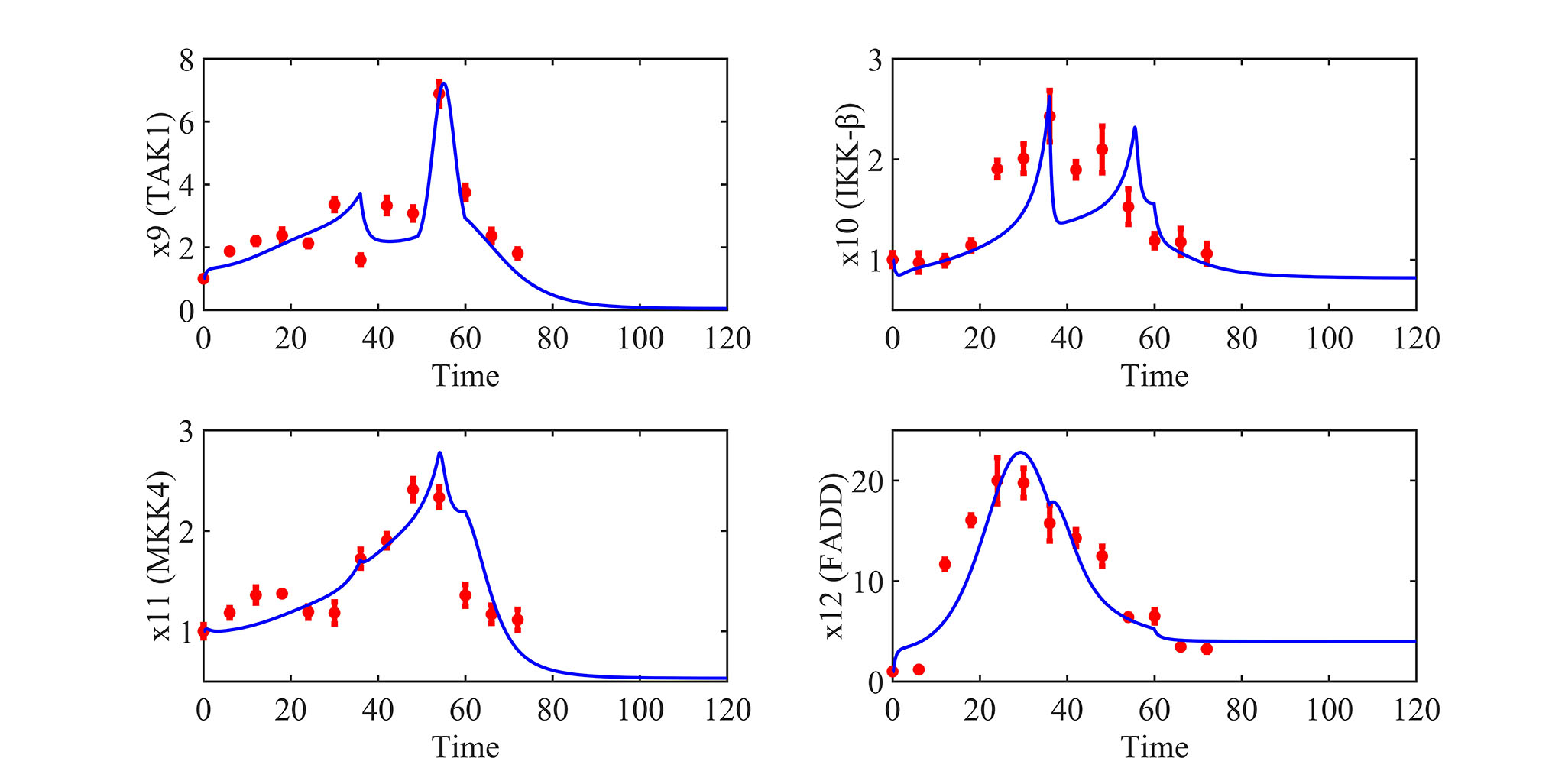

Supplement: Supplementary file 2 [file DataSheet1.zip › Supplementary material_image1/Parameter_a16(大)/3.jpg]

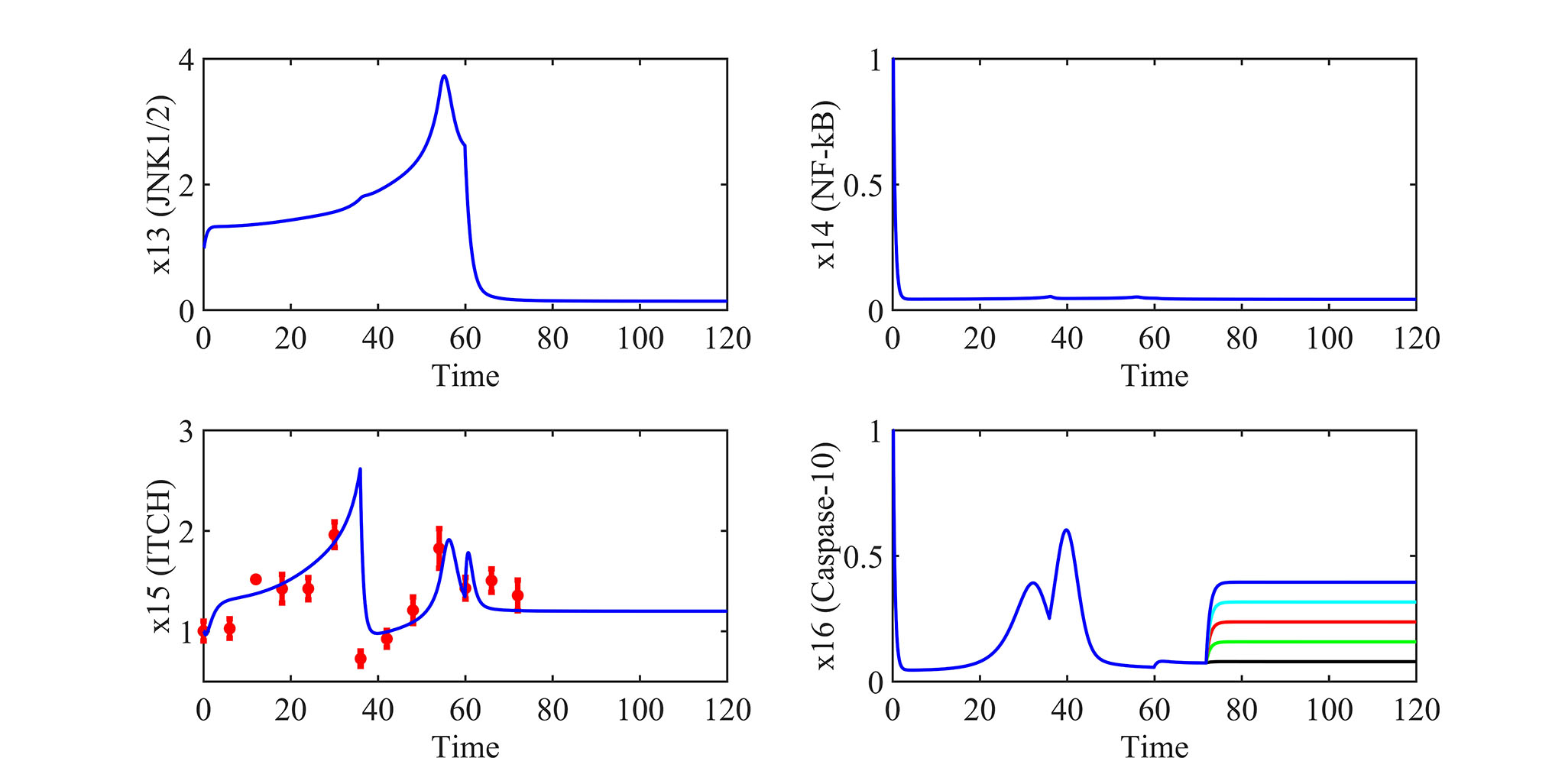

Supplement: Supplementary file 2 [file DataSheet1.zip › Supplementary material_image1/Parameter_a16(大)/4.jpg]

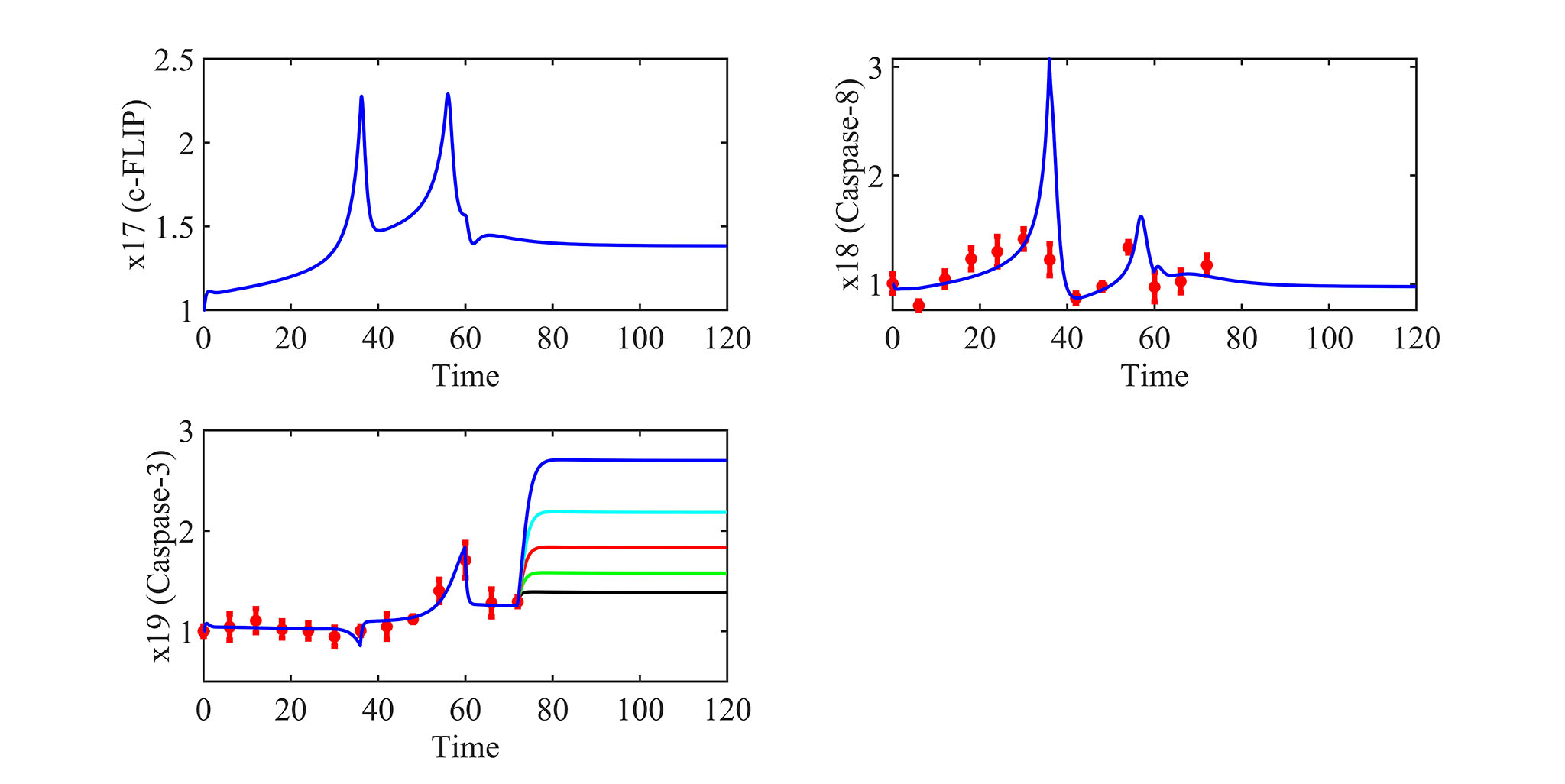

Supplement: Supplementary file 2 [file DataSheet1.zip › Supplementary material_image1/Parameter_a16(大)/5.jpg]

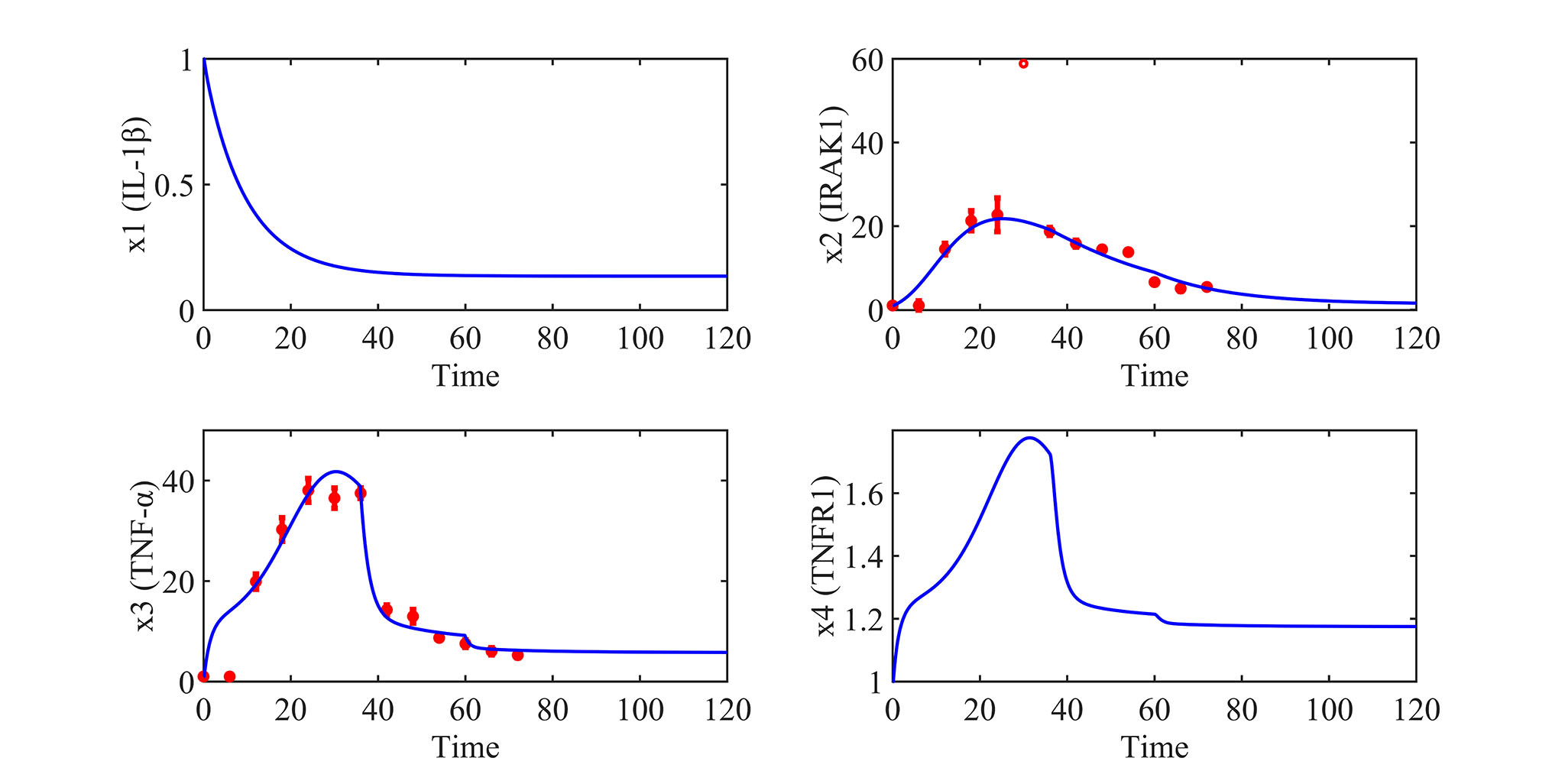

Supplement: Supplementary file 2 [file DataSheet1.zip › Supplementary material_image1/Parameter_a17(大)/1.jpg]

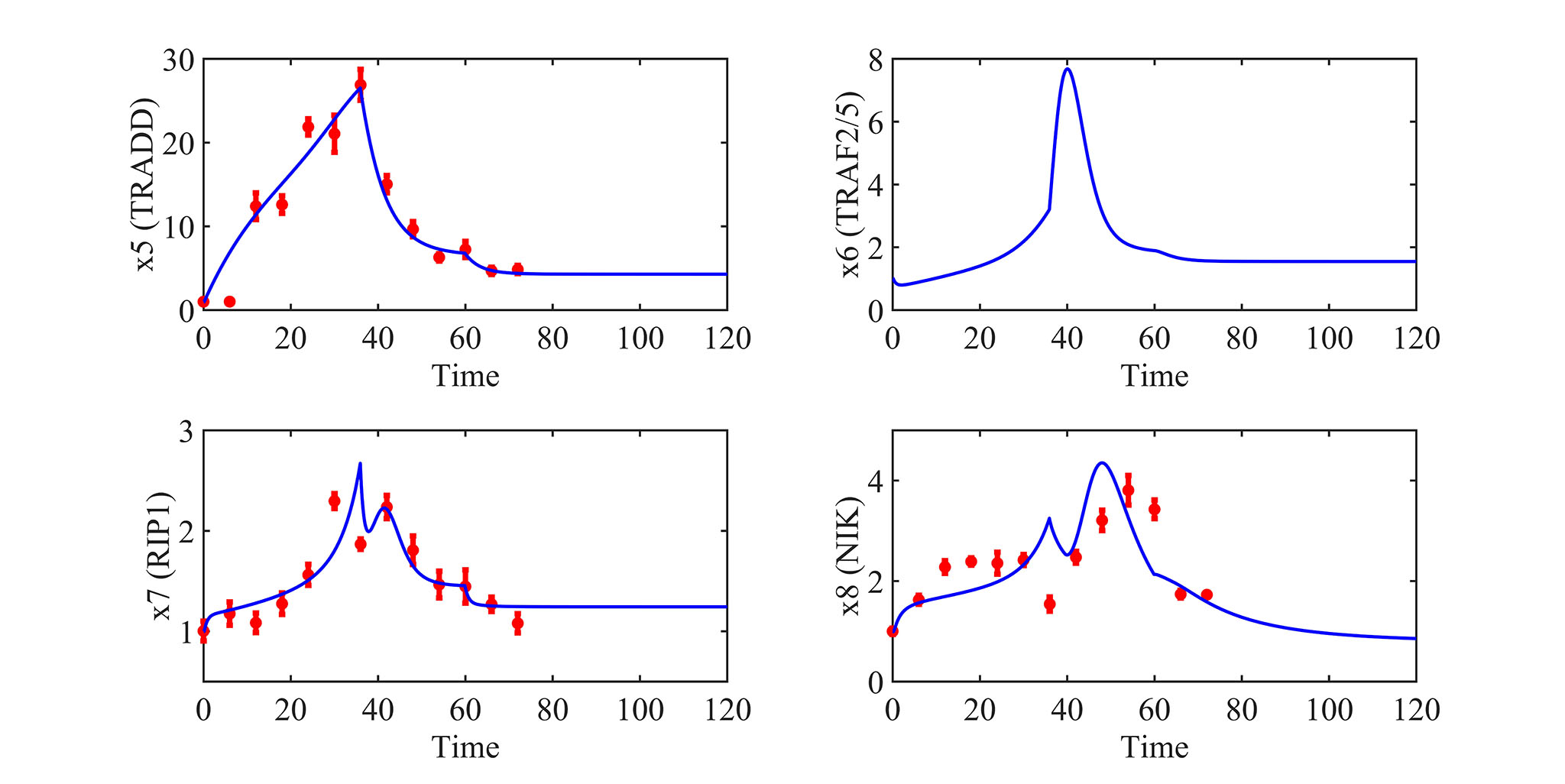

Supplement: Supplementary file 2 [file DataSheet1.zip › Supplementary material_image1/Parameter_a17(大)/2.jpg]

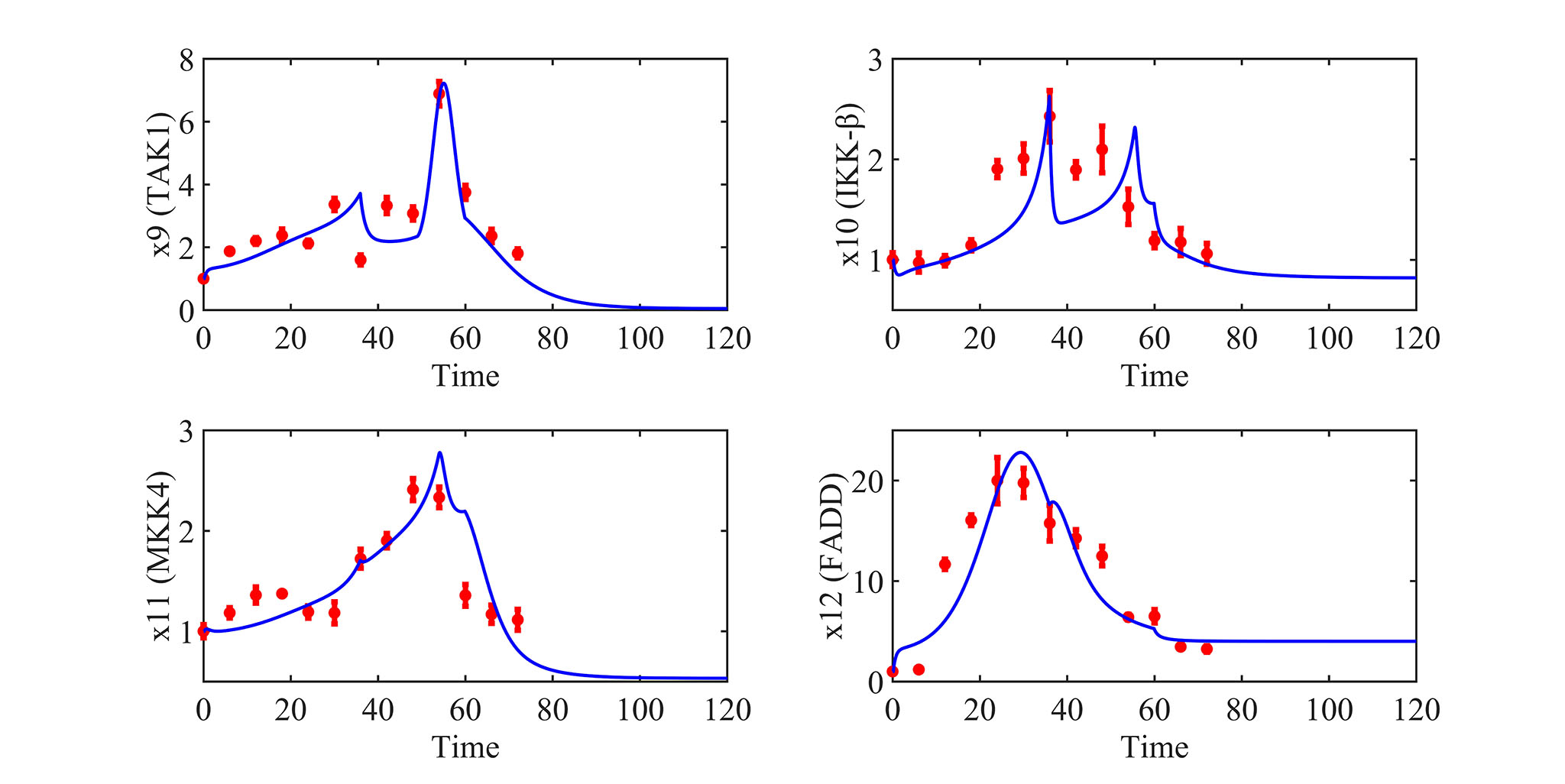

Supplement: Supplementary file 2 [file DataSheet1.zip › Supplementary material_image1/Parameter_a17(大)/3.jpg]

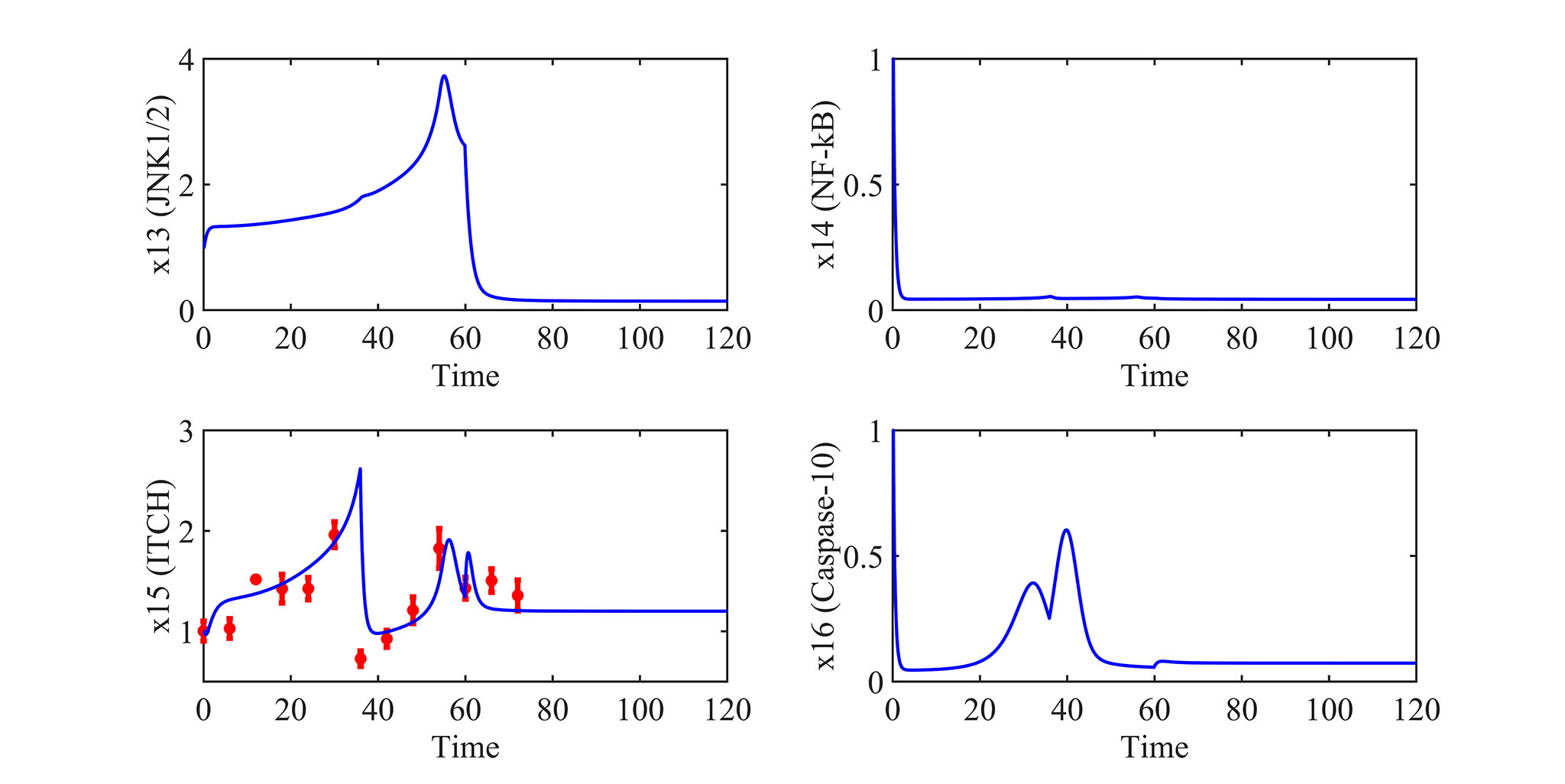

Supplement: Supplementary file 2 [file DataSheet1.zip › Supplementary material_image1/Parameter_a17(大)/4.jpg]

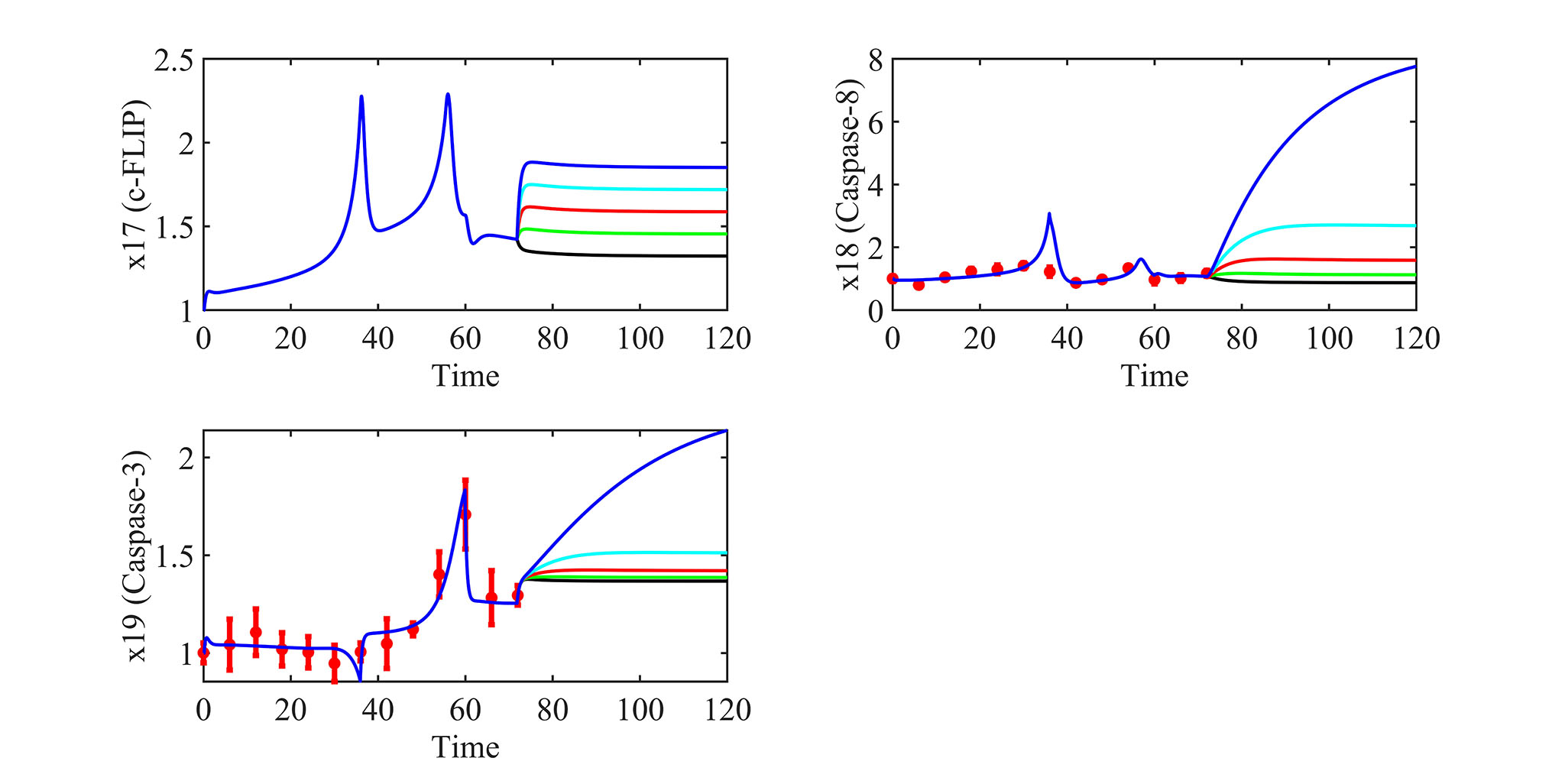

Supplement: Supplementary file 2 [file DataSheet1.zip › Supplementary material_image1/Parameter_a17(大)/5.jpg]

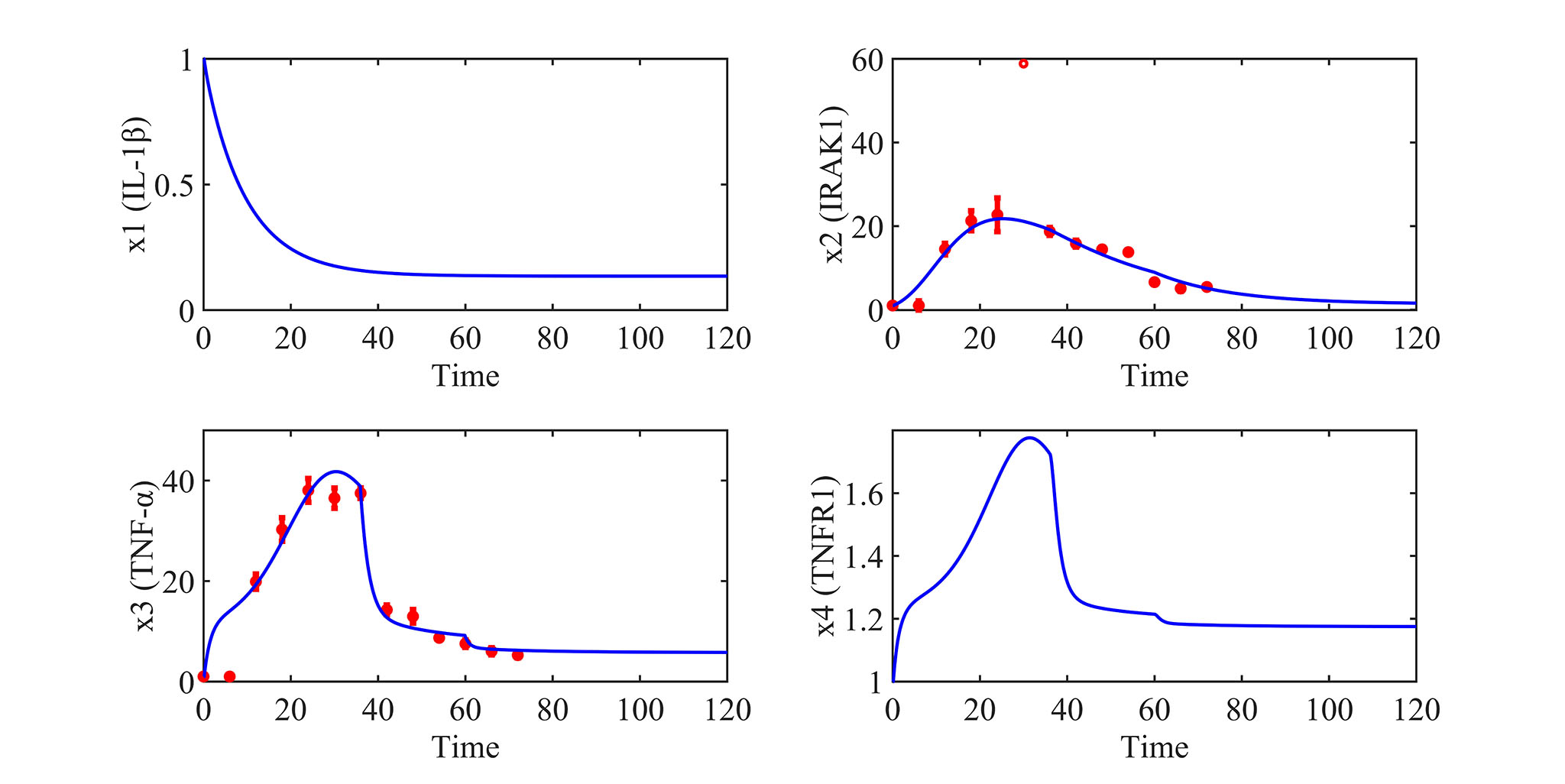

Supplement: Supplementary file 2 [file DataSheet1.zip › Supplementary material_image1/Parameter_a18(大)/1.jpg]

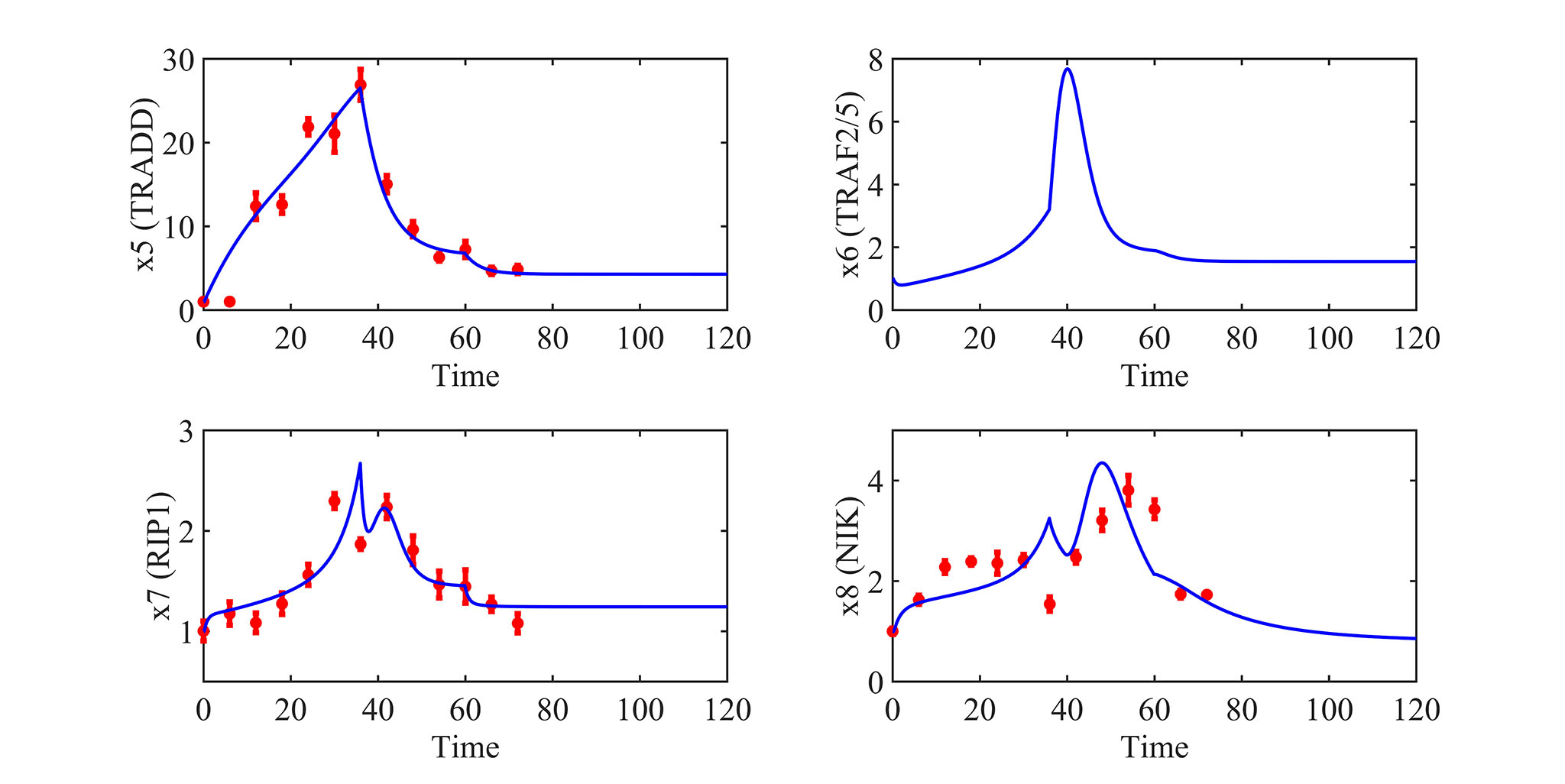

Supplement: Supplementary file 2 [file DataSheet1.zip › Supplementary material_image1/Parameter_a18(大)/2.jpg]

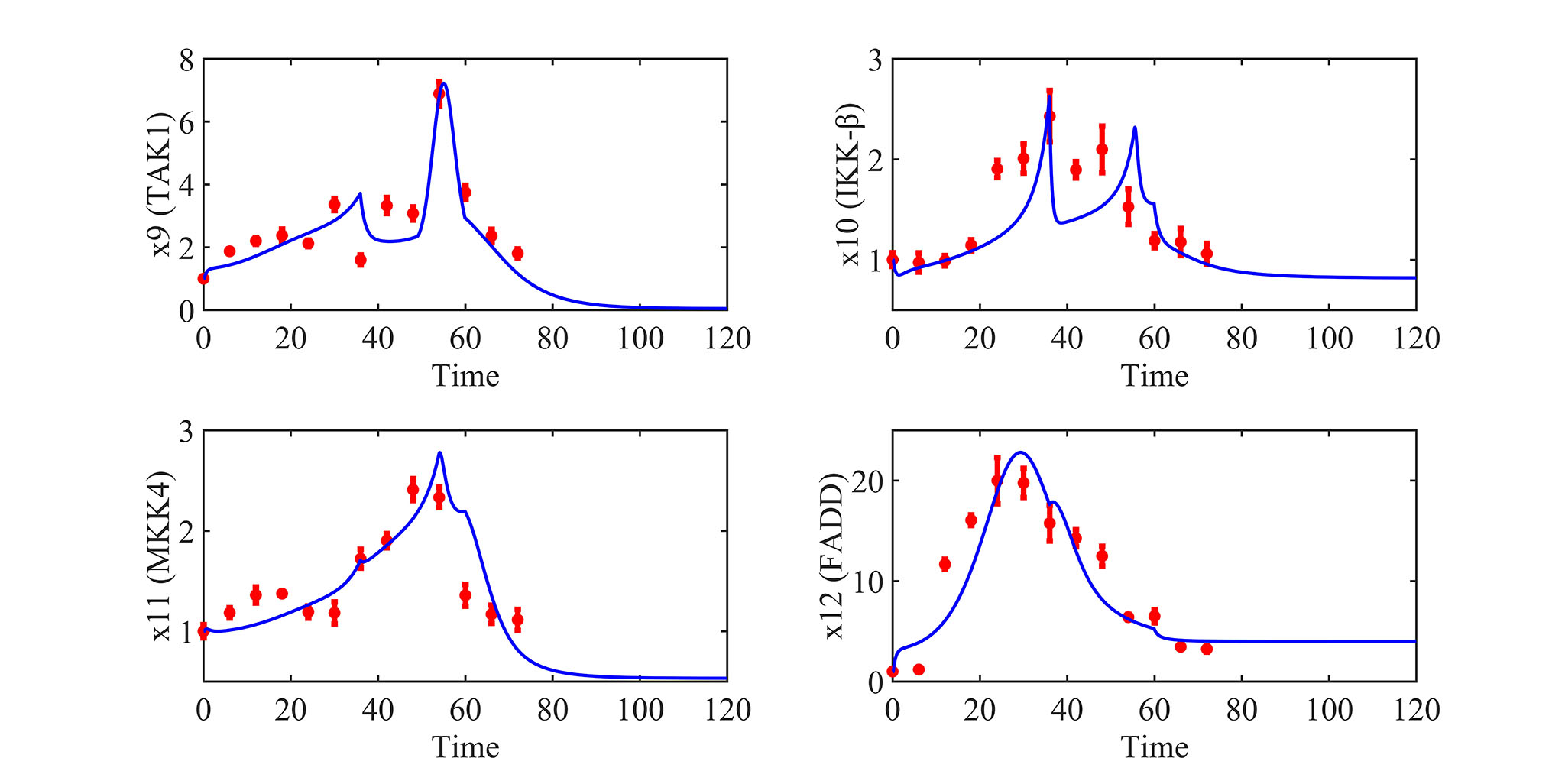

Supplement: Supplementary file 2 [file DataSheet1.zip › Supplementary material_image1/Parameter_a18(大)/3.jpg]

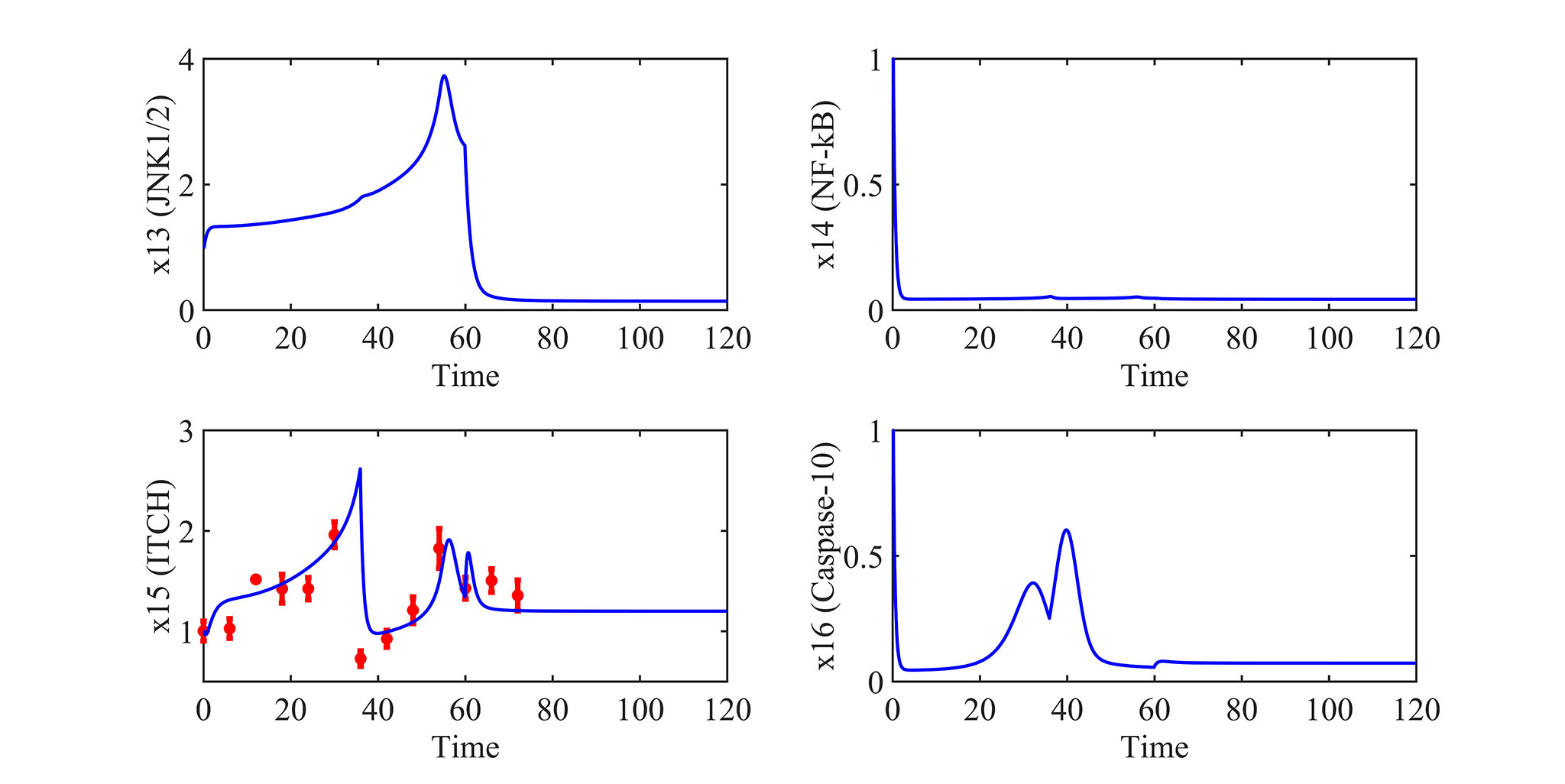

Supplement: Supplementary file 2 [file DataSheet1.zip › Supplementary material_image1/Parameter_a18(大)/4.jpg]

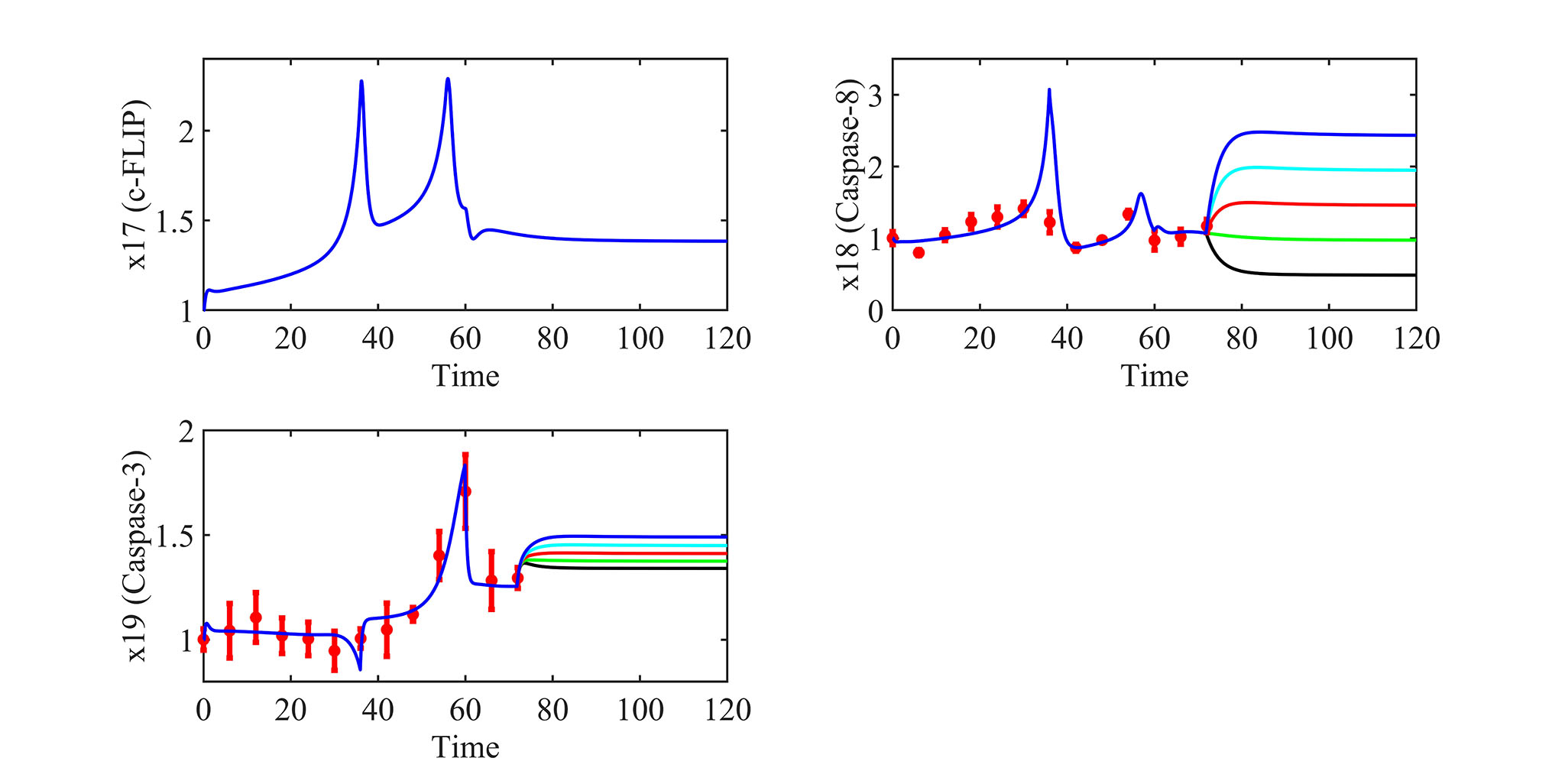

Supplement: Supplementary file 2 [file DataSheet1.zip › Supplementary material_image1/Parameter_a18(大)/5.jpg]

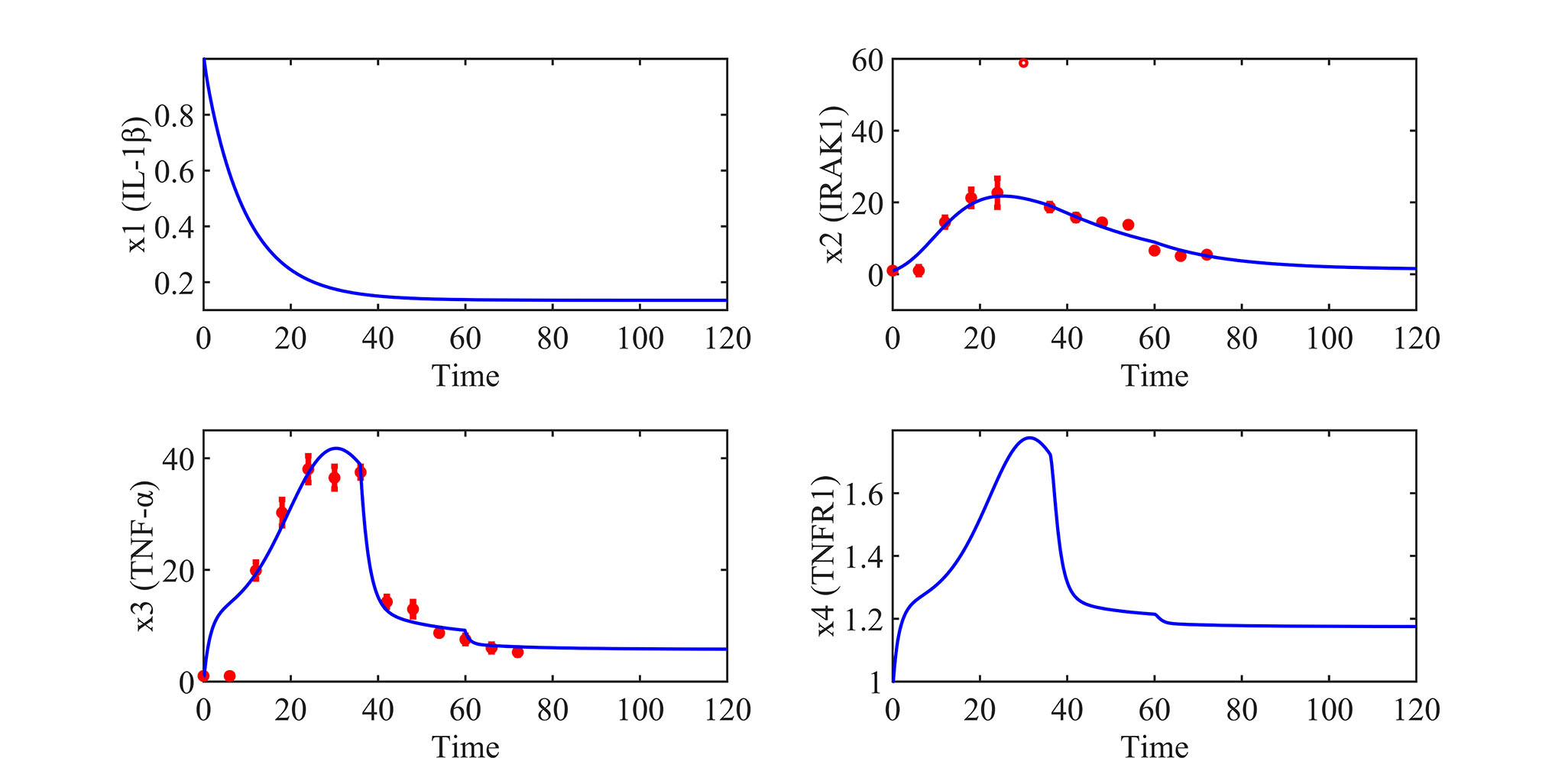

Supplement: Supplementary file 2 [file DataSheet1.zip › Supplementary material_image1/Parameter_a19(大)/1.jpg]

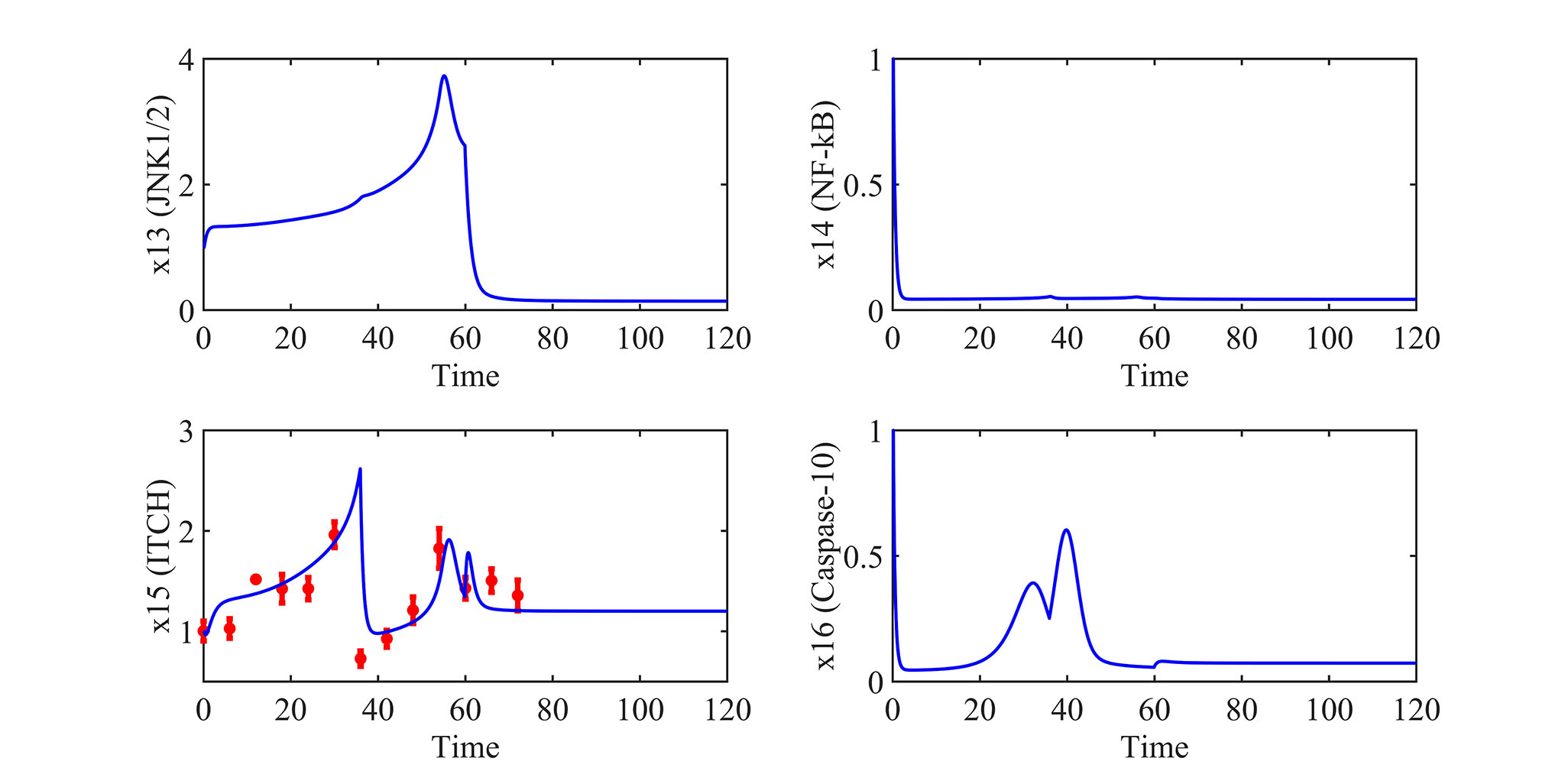

Supplement: Supplementary file 2 [file DataSheet1.zip › Supplementary material_image1/Parameter_a19(大)/2.jpg]

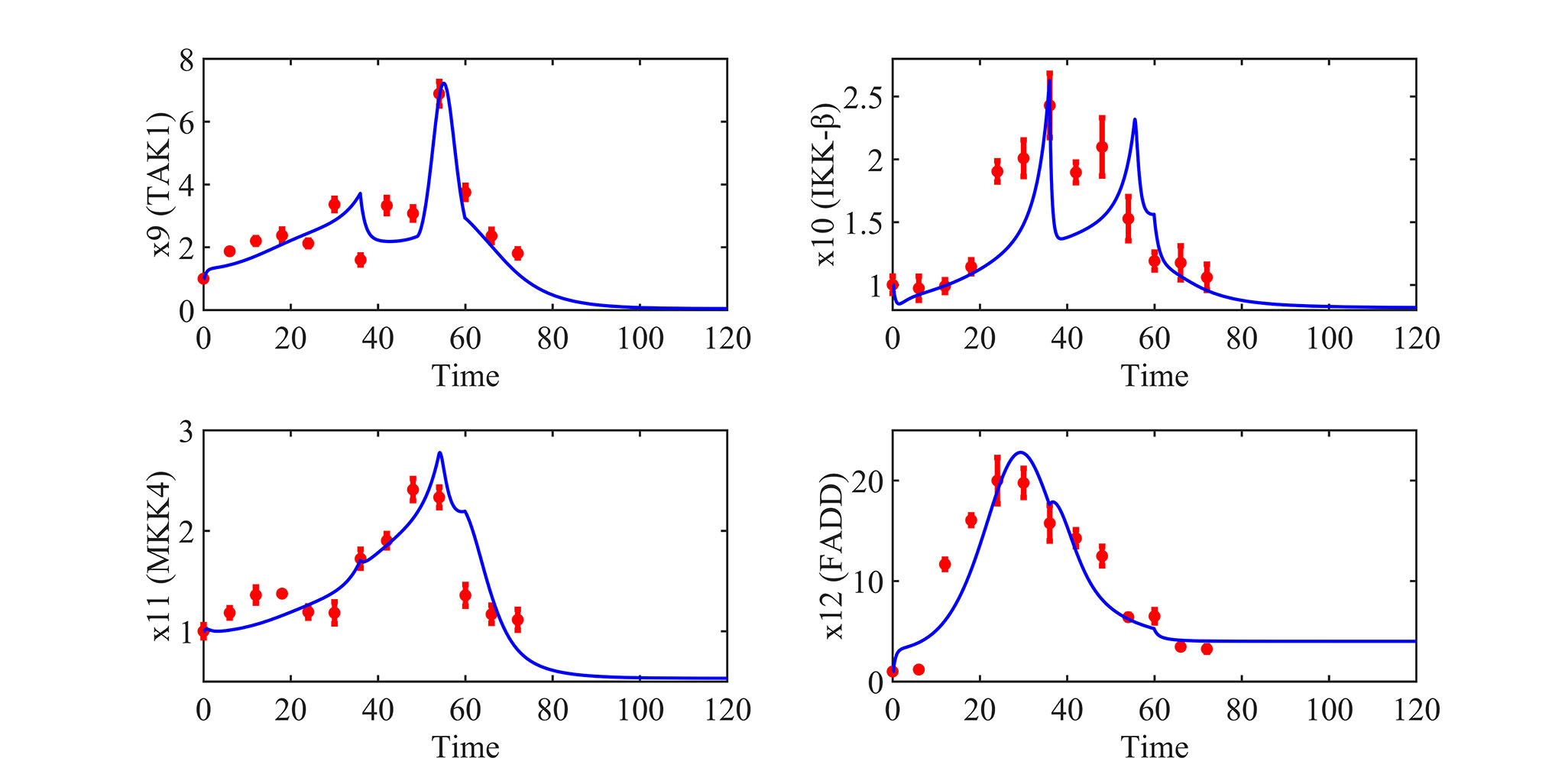

Supplement: Supplementary file 2 [file DataSheet1.zip › Supplementary material_image1/Parameter_a19(大)/3.jpg]

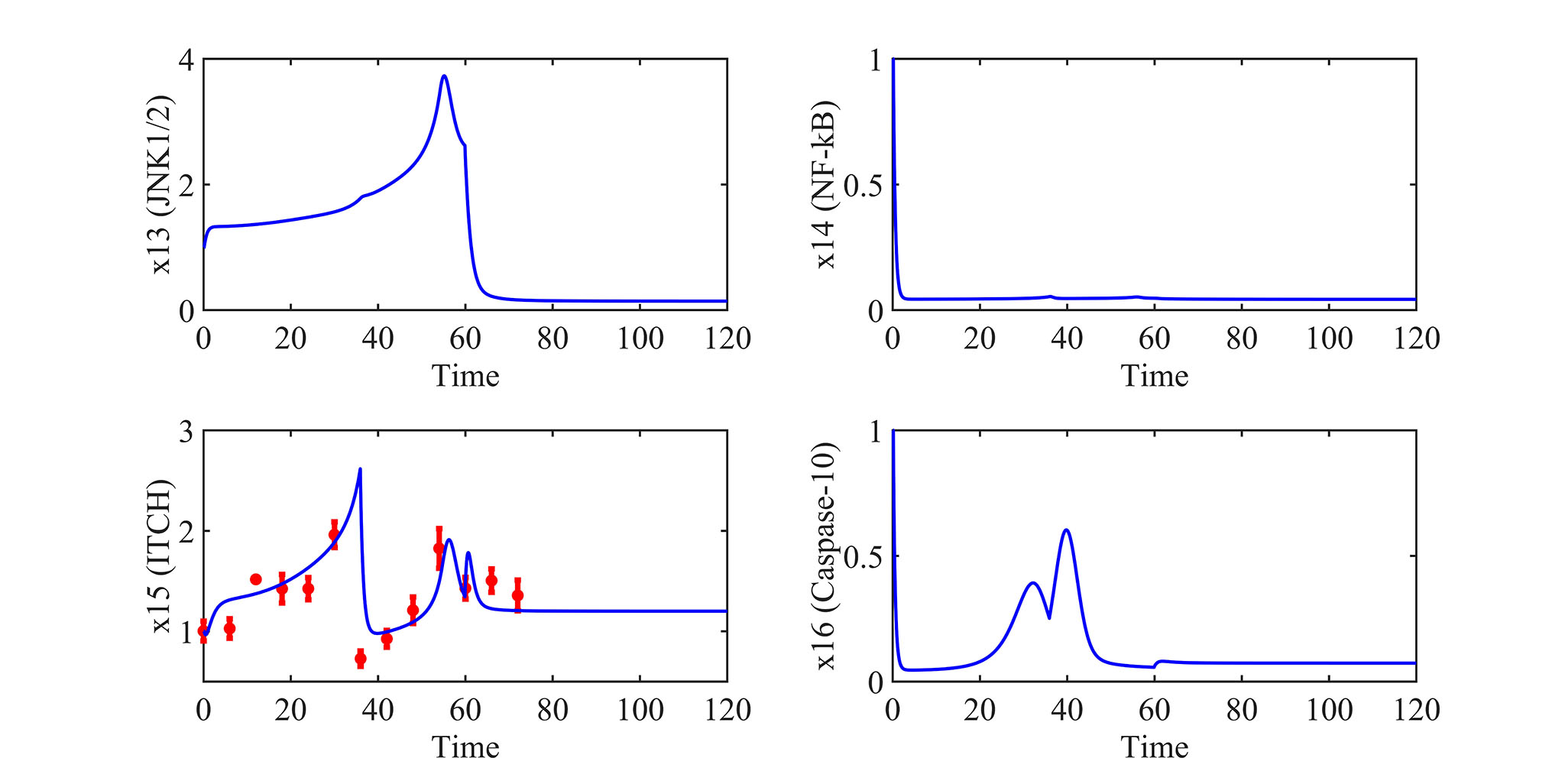

Supplement: Supplementary file 2 [file DataSheet1.zip › Supplementary material_image1/Parameter_a19(大)/4.jpg]

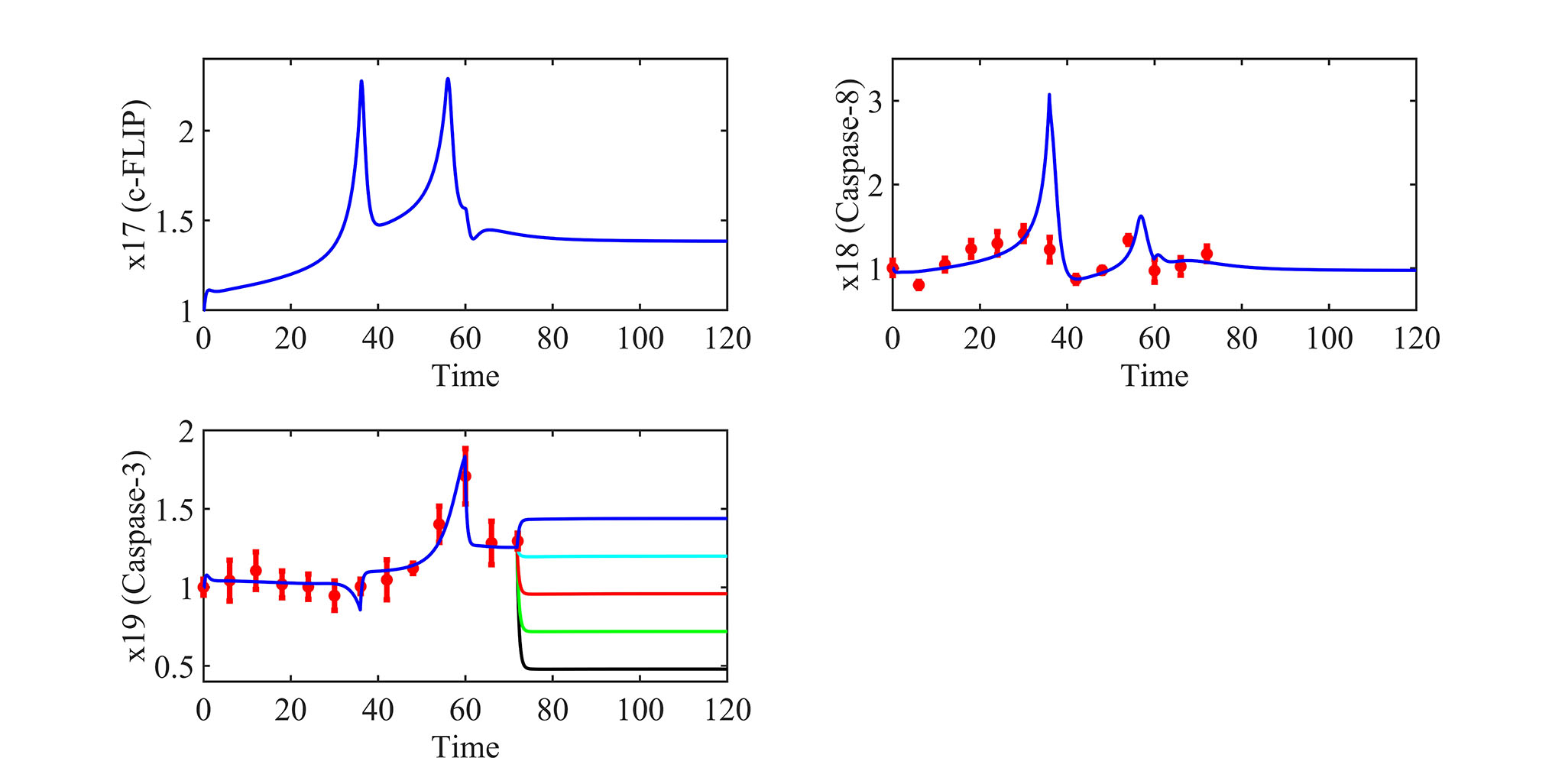

Supplement: Supplementary file 2 [file DataSheet1.zip › Supplementary material_image1/Parameter_a19(大)/5.jpg]

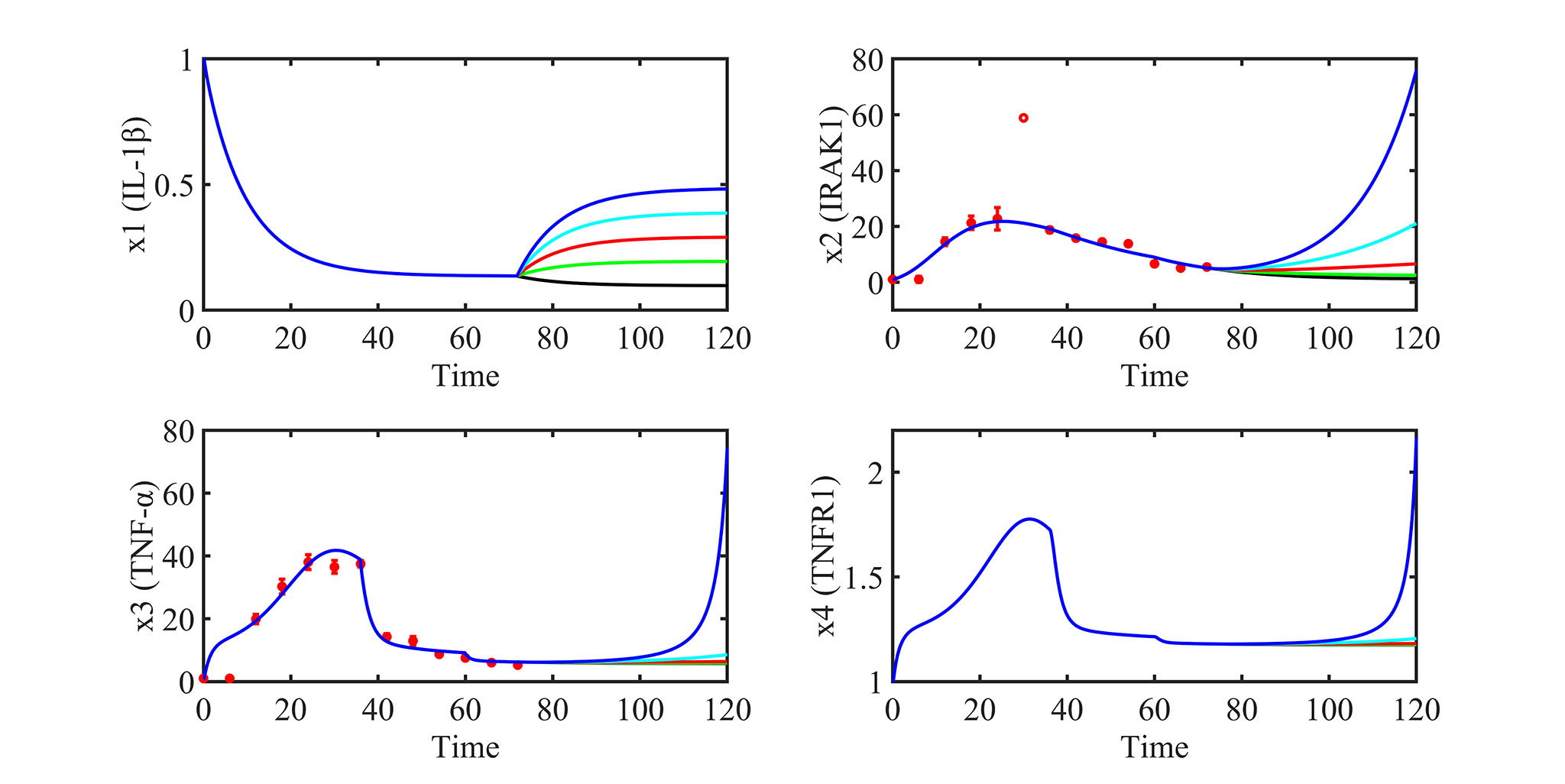

Supplement: Supplementary file 2 [file DataSheet1.zip › Supplementary material_image1/Parameter_a1(小)/1.jpg]

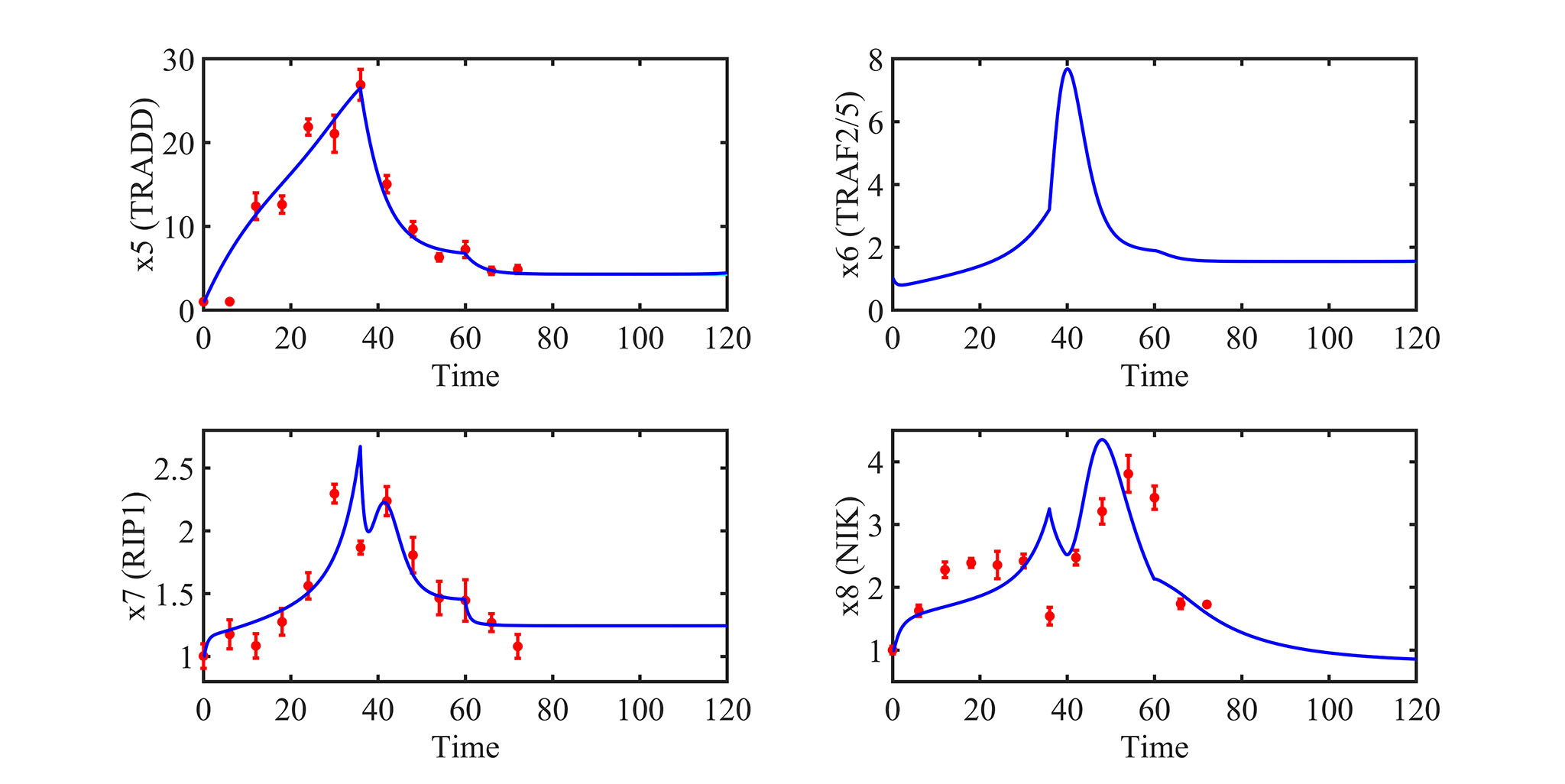

Supplement: Supplementary file 2 [file DataSheet1.zip › Supplementary material_image1/Parameter_a1(小)/2.jpg]

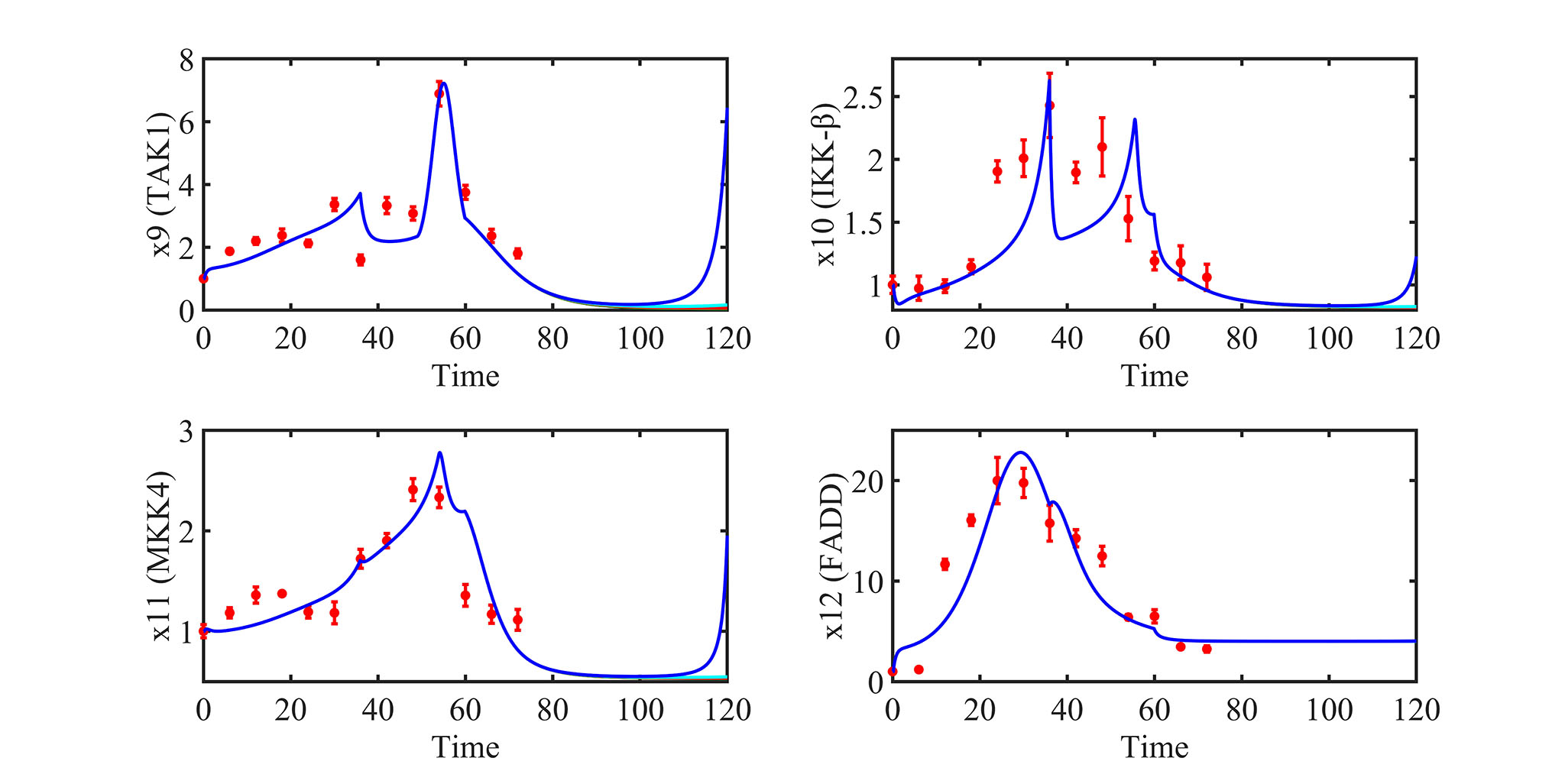

Supplement: Supplementary file 2 [file DataSheet1.zip › Supplementary material_image1/Parameter_a1(小)/3.jpg]

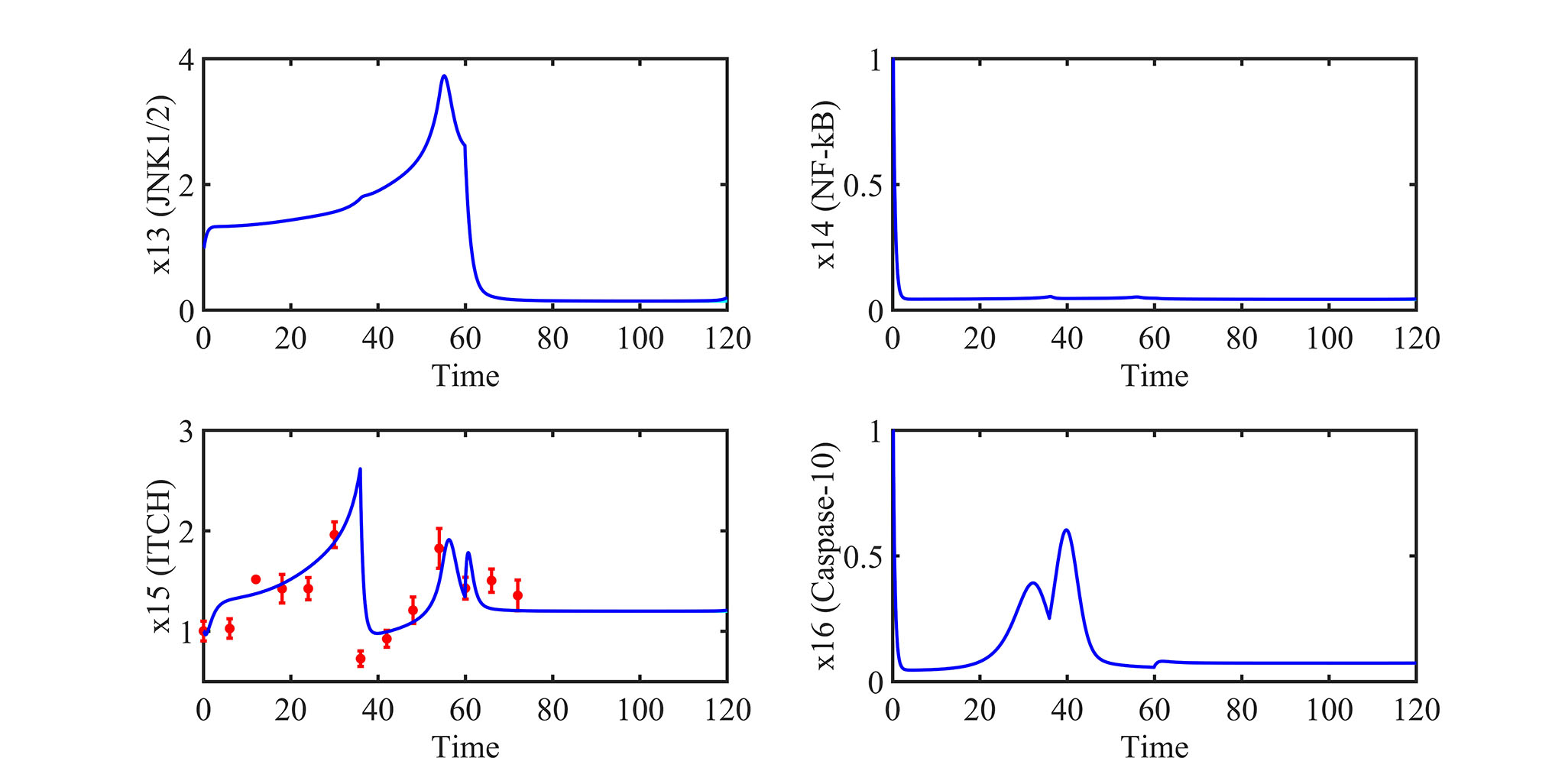

Supplement: Supplementary file 2 [file DataSheet1.zip › Supplementary material_image1/Parameter_a1(小)/4.jpg]

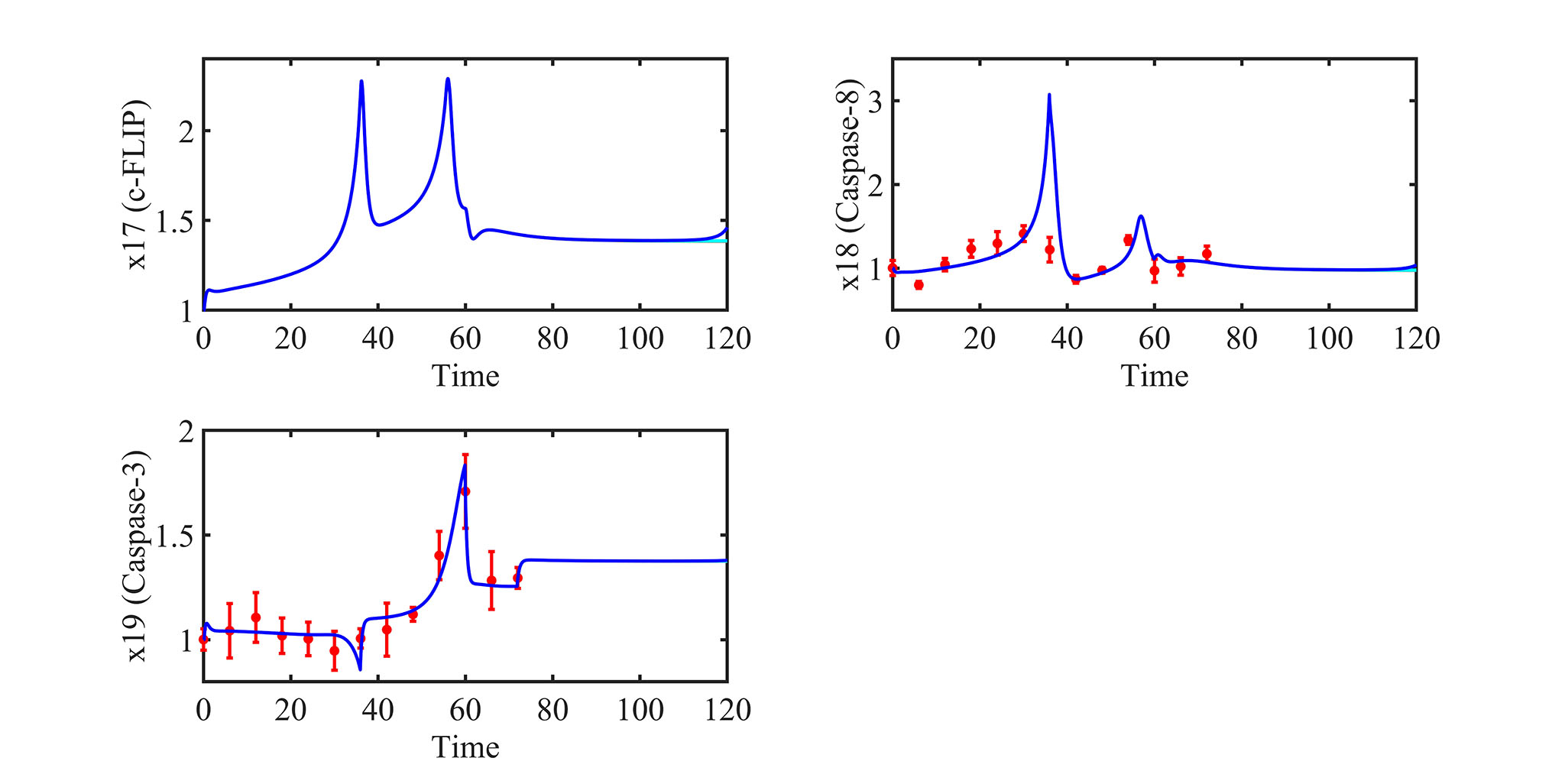

Supplement: Supplementary file 2 [file DataSheet1.zip › Supplementary material_image1/Parameter_a1(小)/5.jpg]

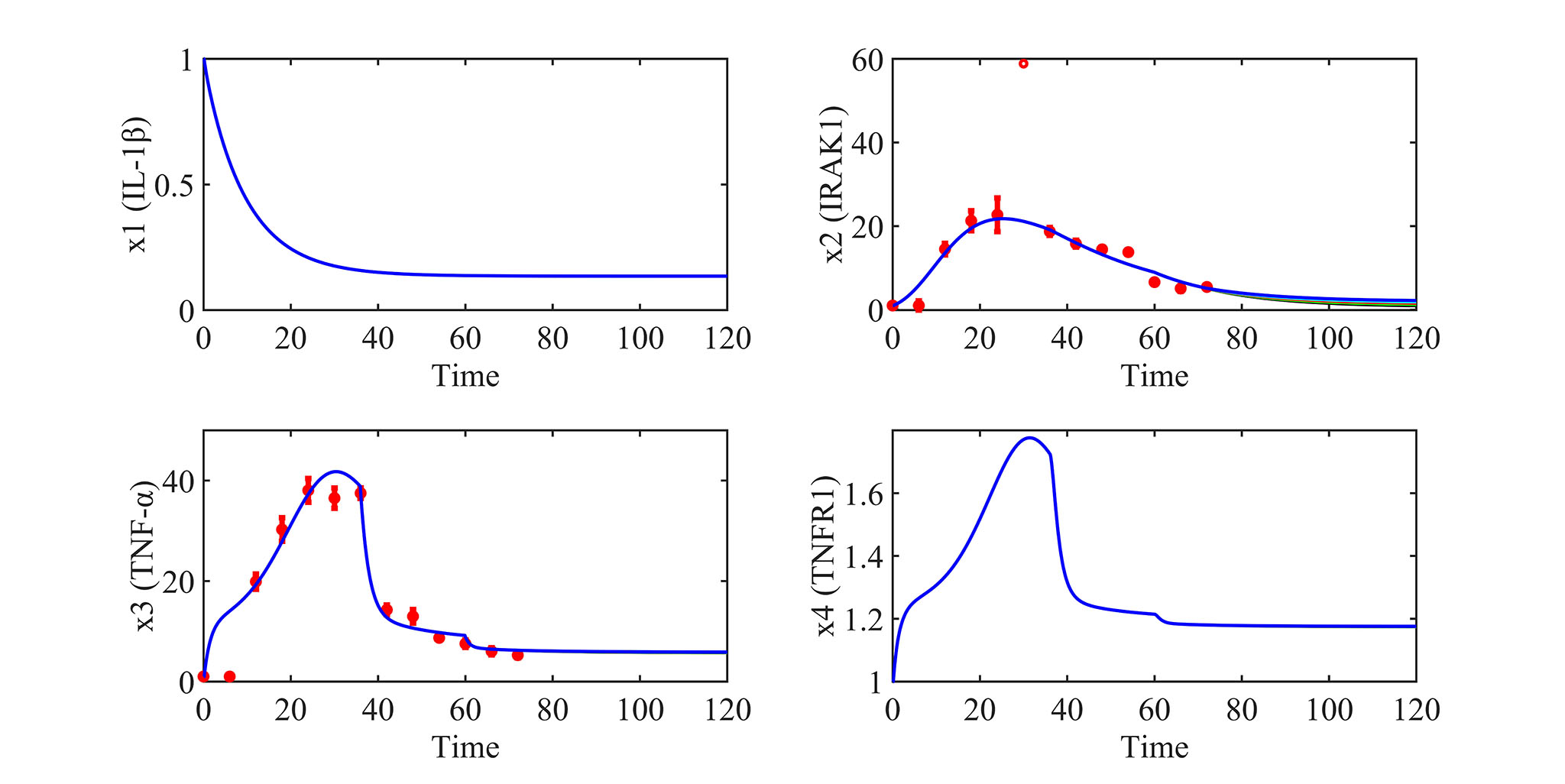

Supplement: Supplementary file 2 [file DataSheet1.zip › Supplementary material_image1/Parameter_a2(小)/1.jpg]

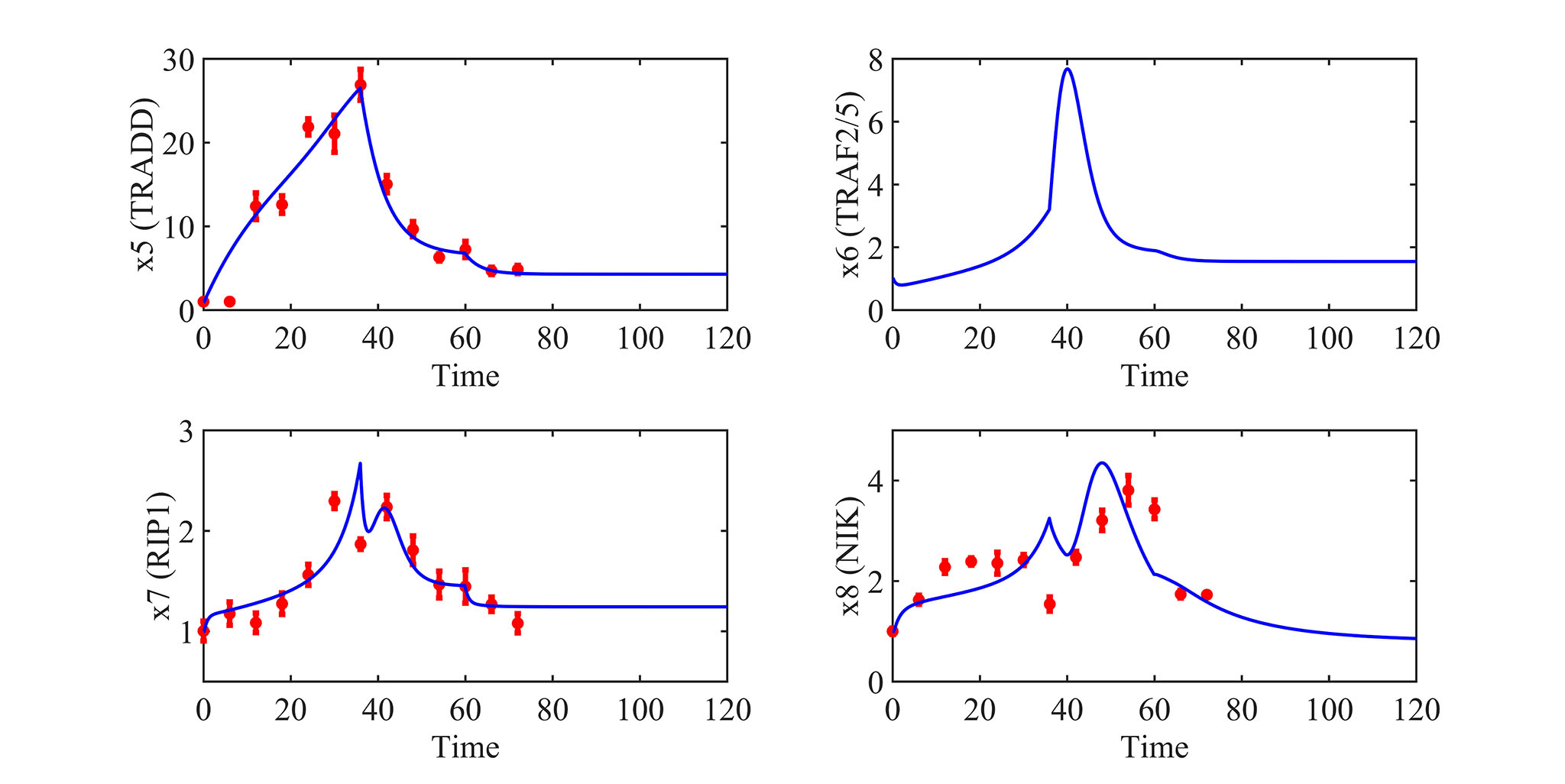

Supplement: Supplementary file 2 [file DataSheet1.zip › Supplementary material_image1/Parameter_a2(小)/2.jpg]

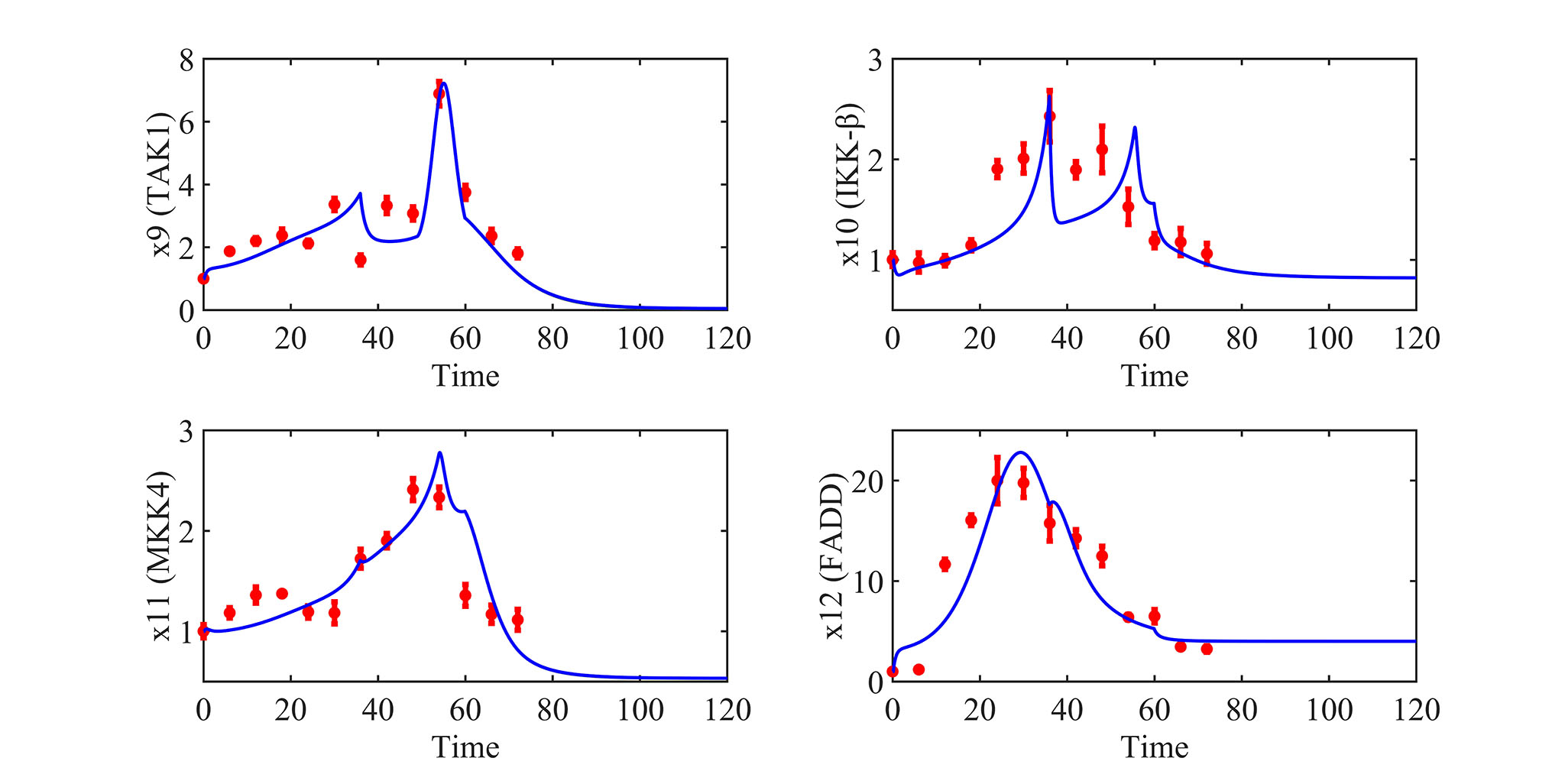

Supplement: Supplementary file 2 [file DataSheet1.zip › Supplementary material_image1/Parameter_a2(小)/3.jpg]

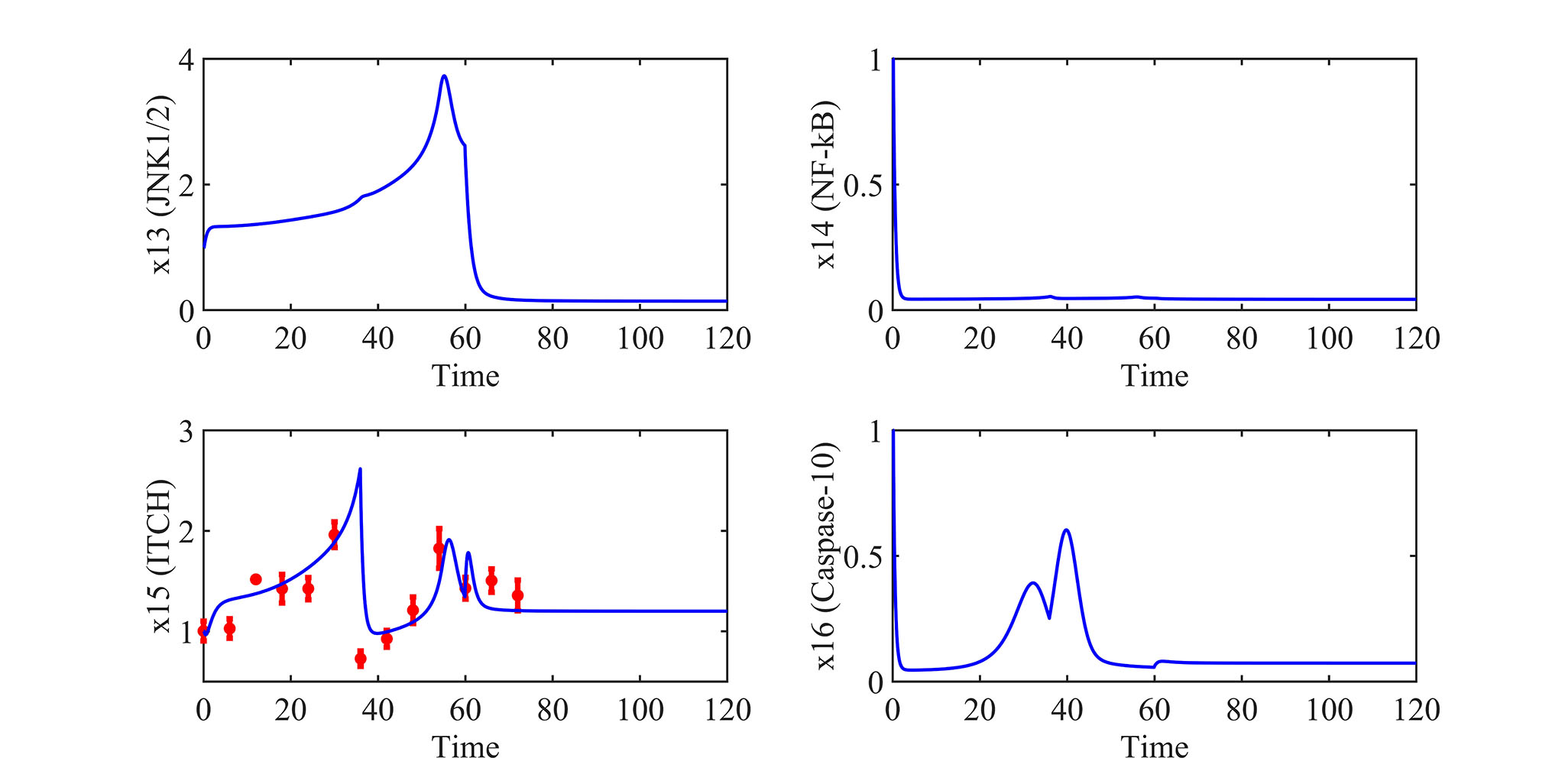

Supplement: Supplementary file 2 [file DataSheet1.zip › Supplementary material_image1/Parameter_a2(小)/4.jpg]

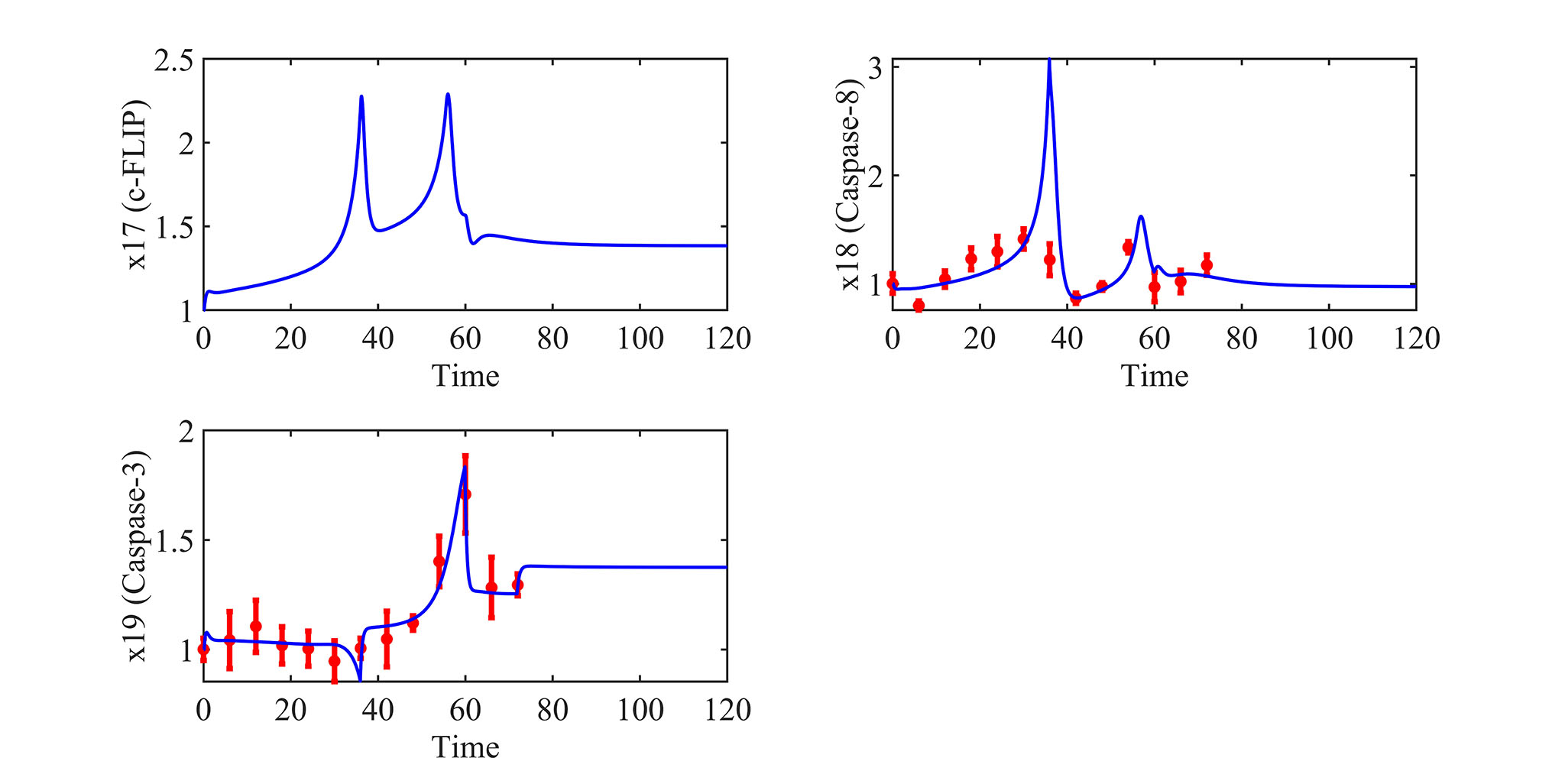

Supplement: Supplementary file 2 [file DataSheet1.zip › Supplementary material_image1/Parameter_a2(小)/5.jpg]

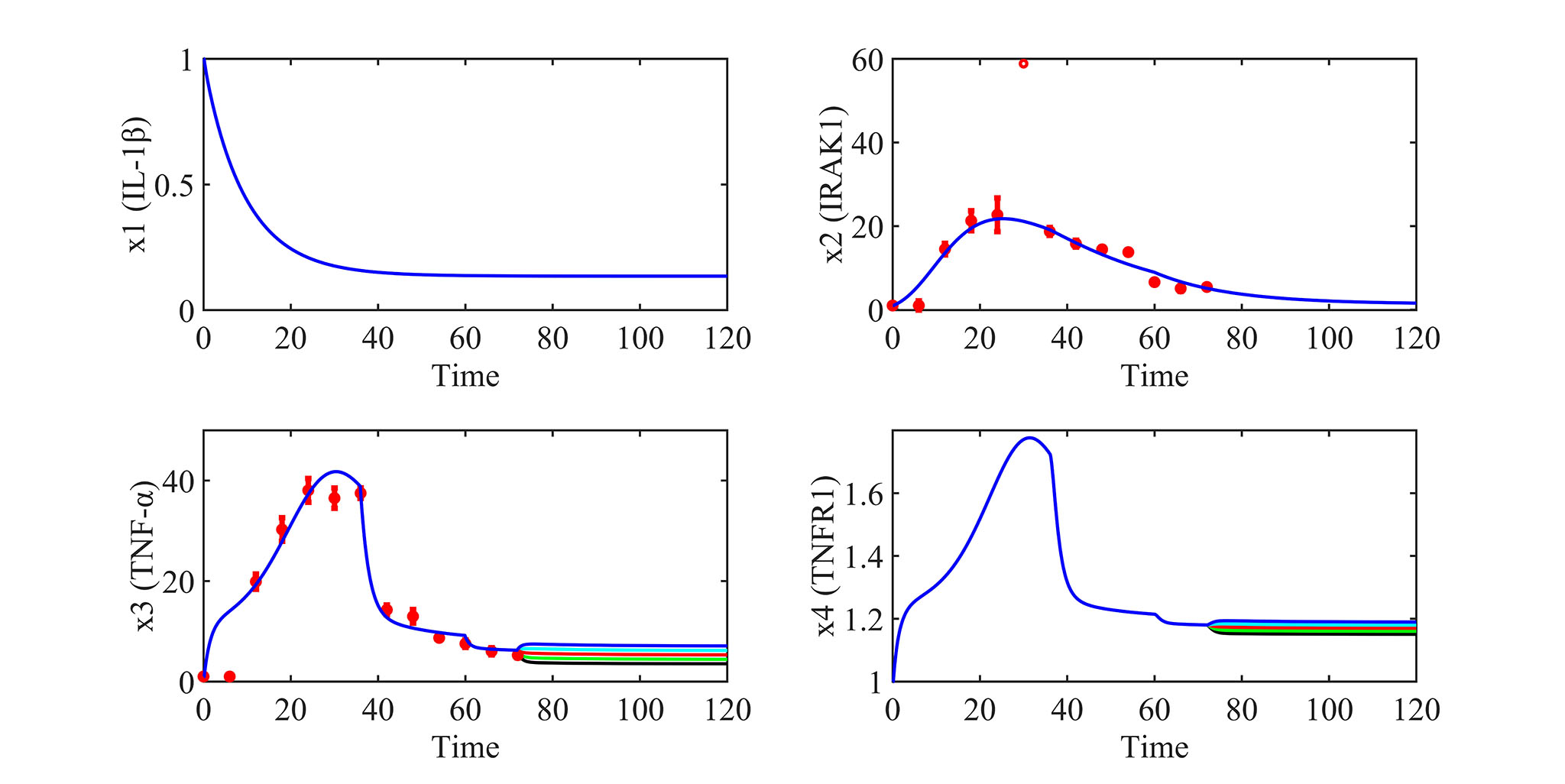

Supplement: Supplementary file 2 [file DataSheet1.zip › Supplementary material_image1/Parameter_a3(小)/1.jpg]

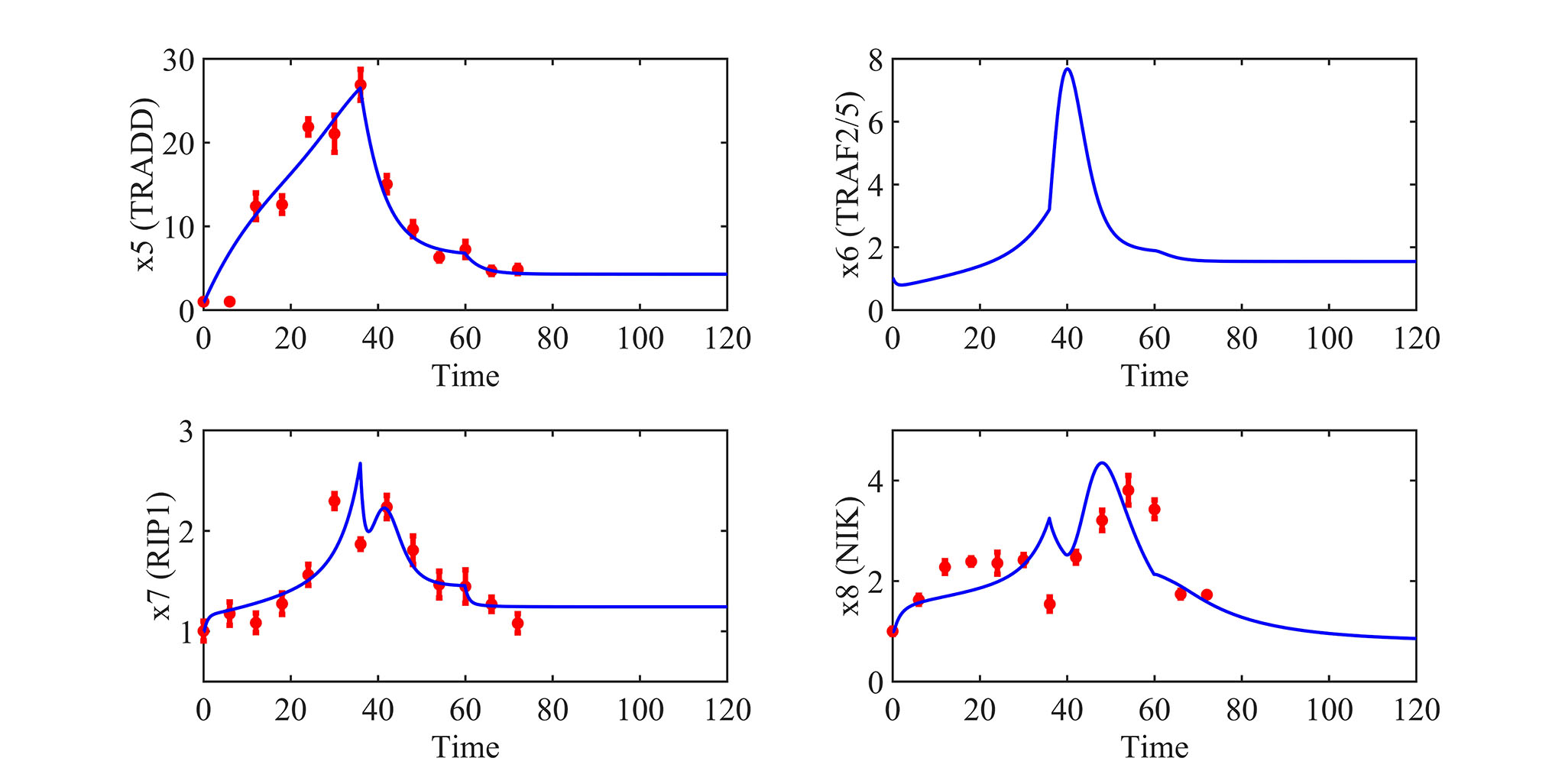

Supplement: Supplementary file 2 [file DataSheet1.zip › Supplementary material_image1/Parameter_a3(小)/2.jpg]

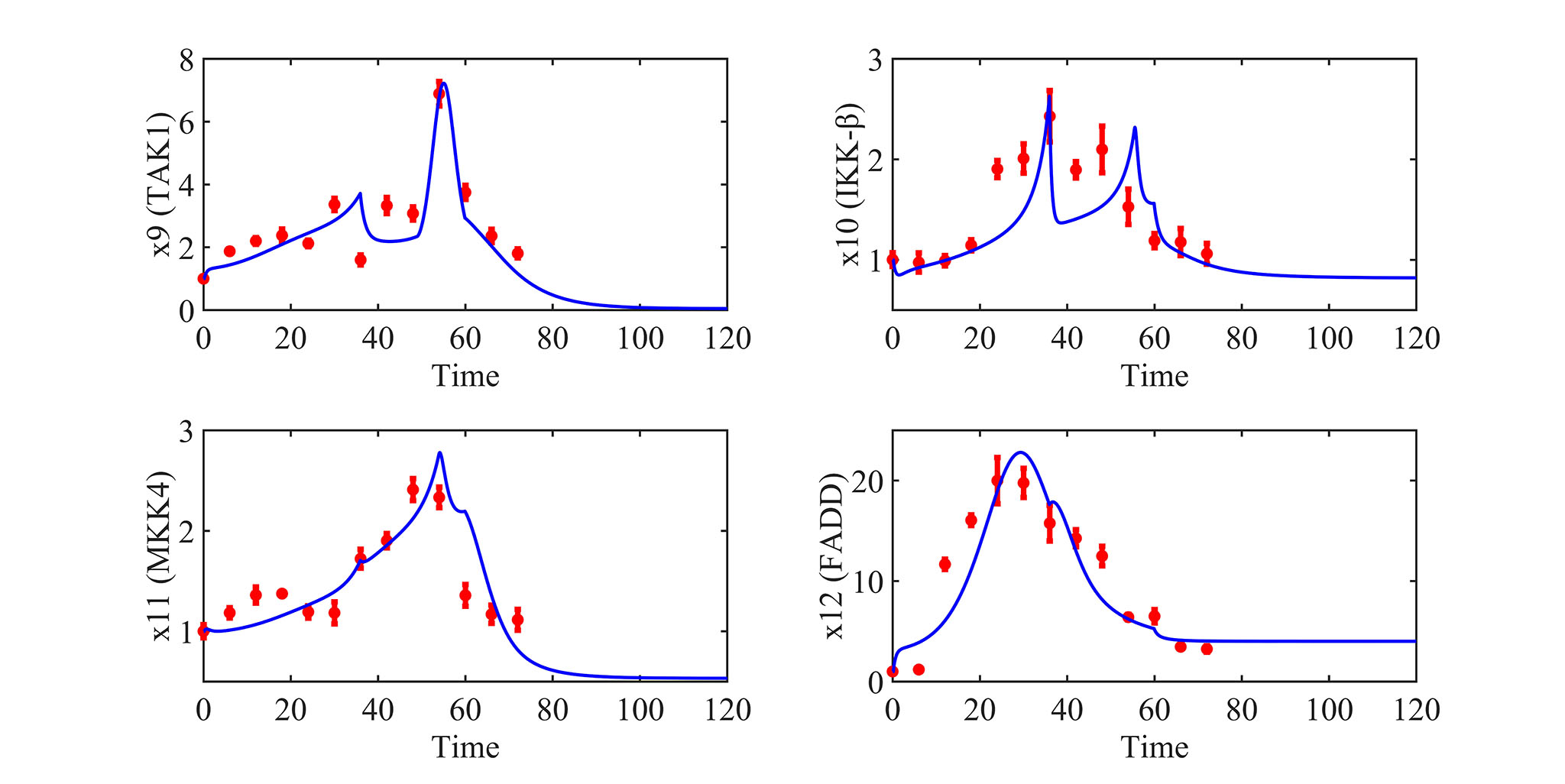

Supplement: Supplementary file 2 [file DataSheet1.zip › Supplementary material_image1/Parameter_a3(小)/3.jpg]

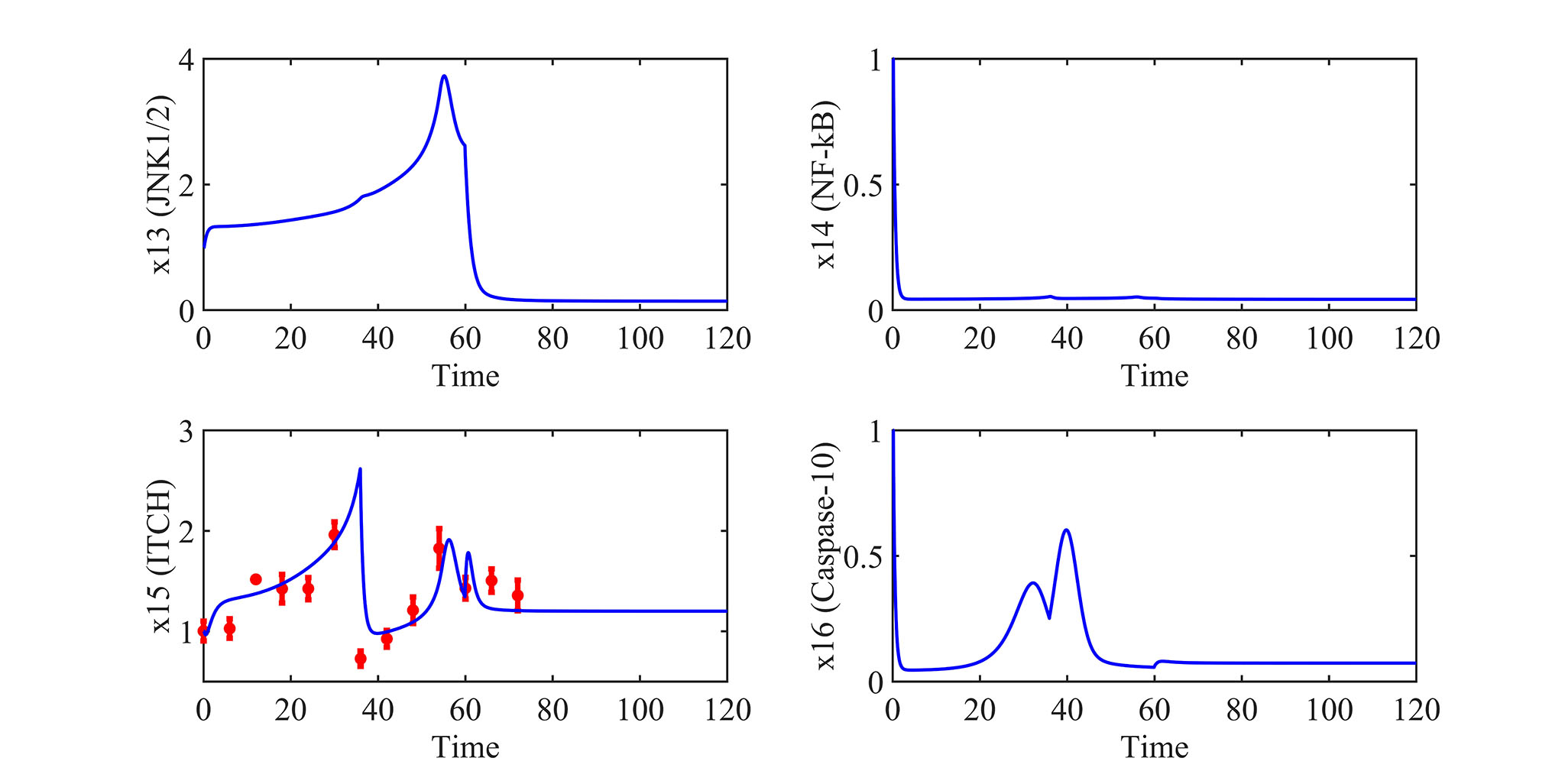

Supplement: Supplementary file 2 [file DataSheet1.zip › Supplementary material_image1/Parameter_a3(小)/4.jpg]

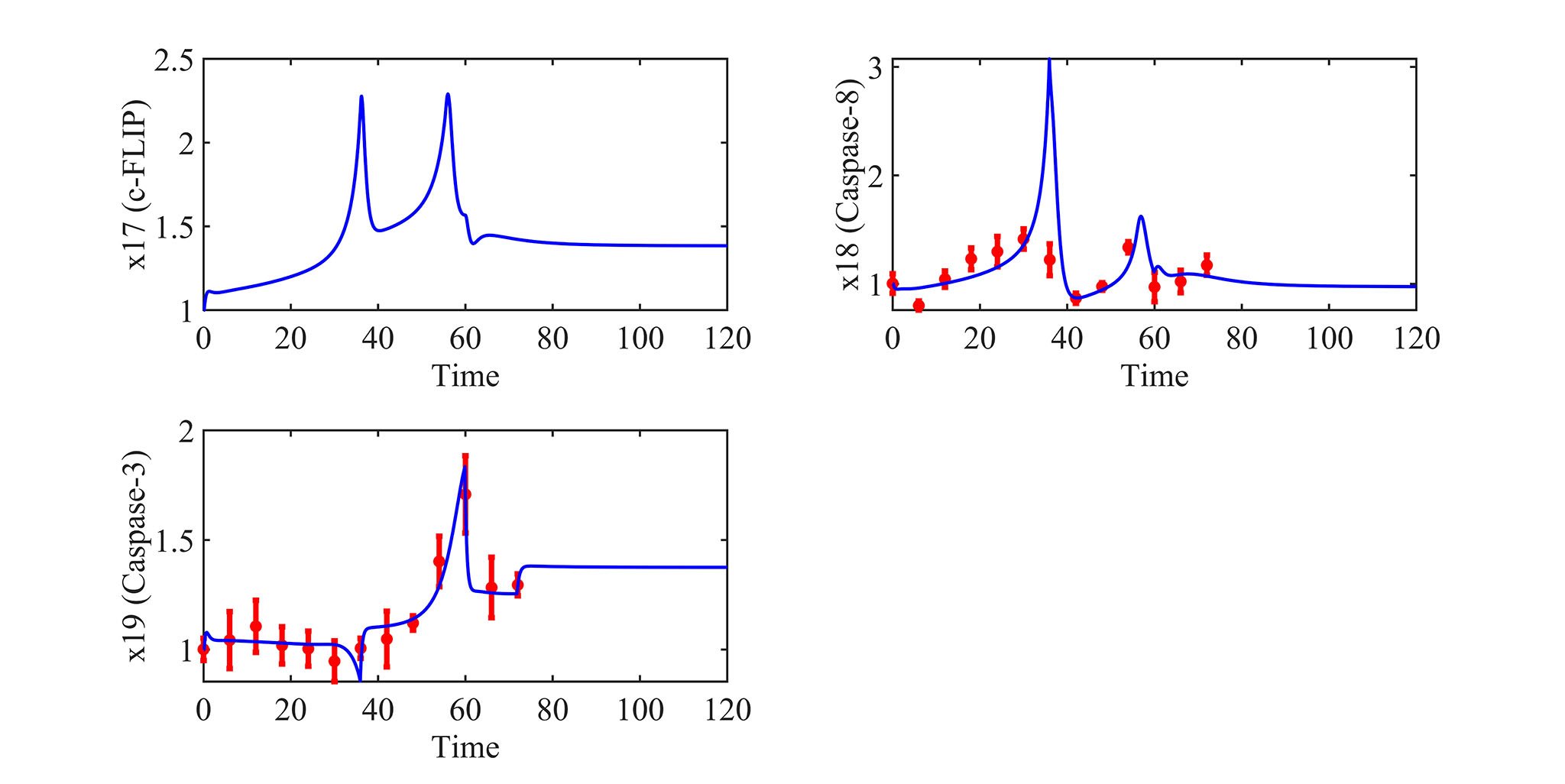

Supplement: Supplementary file 2 [file DataSheet1.zip › Supplementary material_image1/Parameter_a3(小)/5.jpg]

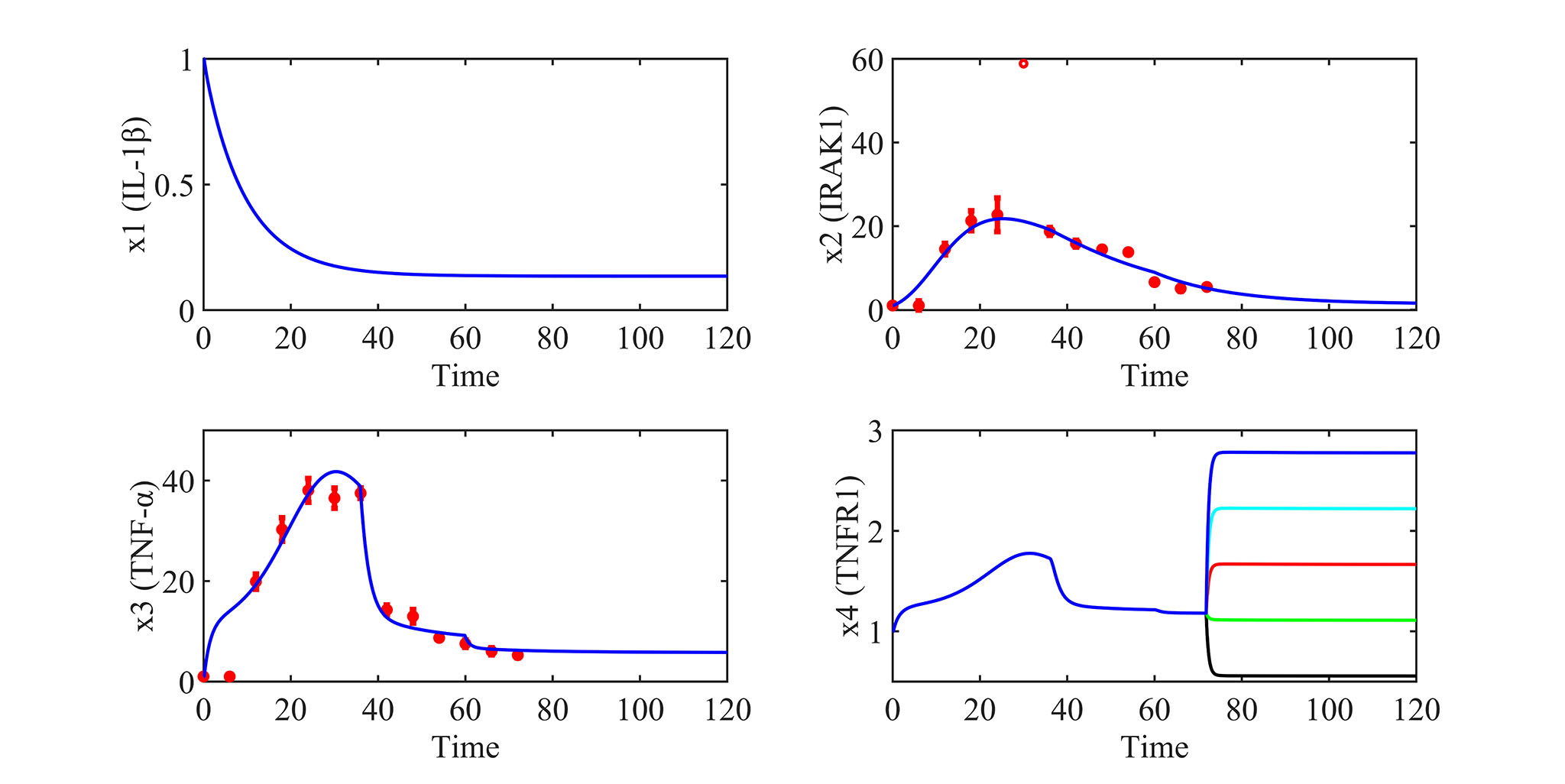

Supplement: Supplementary file 2 [file DataSheet1.zip › Supplementary material_image1/Parameter_a4(小)/1.jpg]

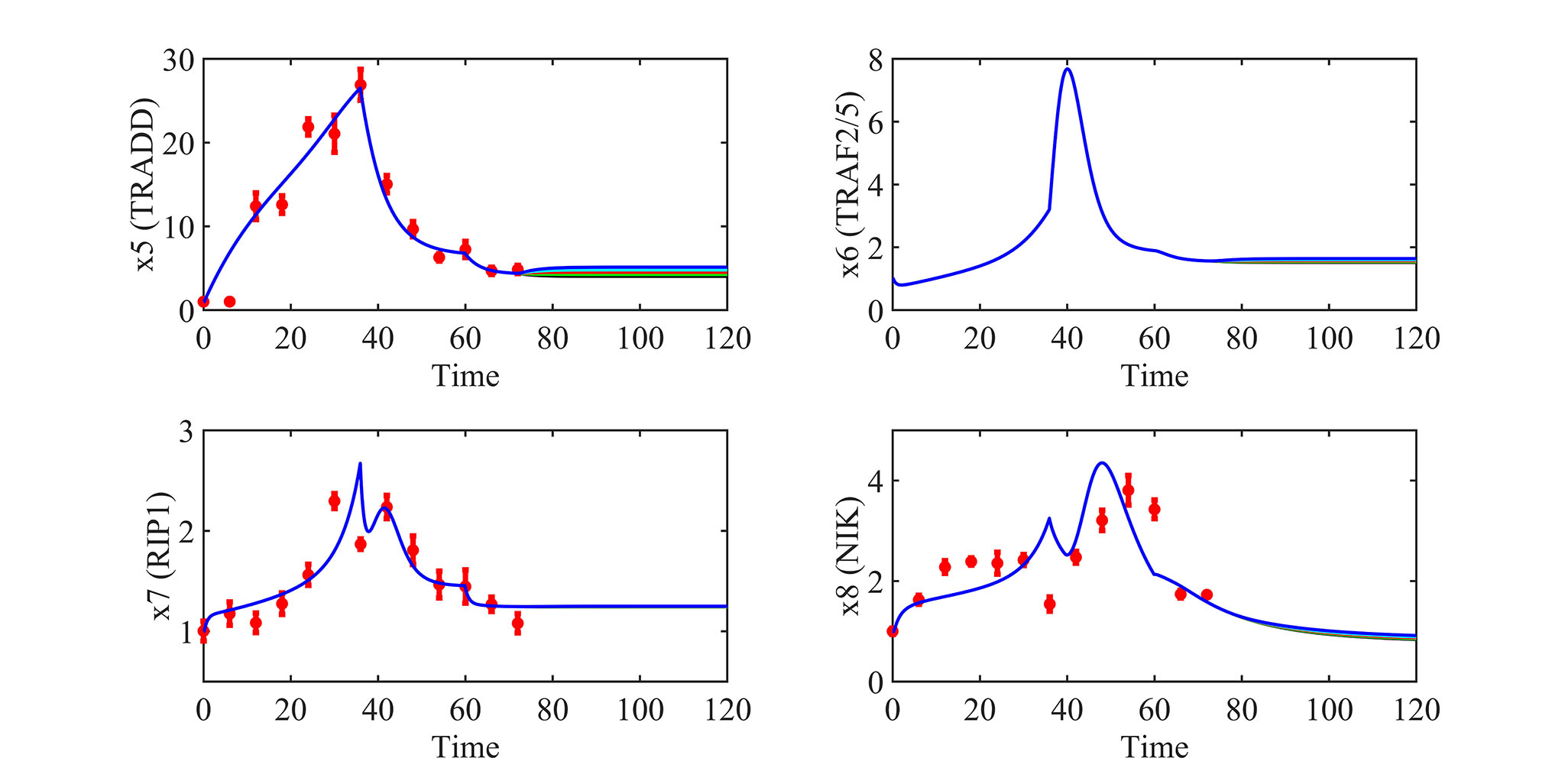

Supplement: Supplementary file 2 [file DataSheet1.zip › Supplementary material_image1/Parameter_a4(小)/2.jpg]

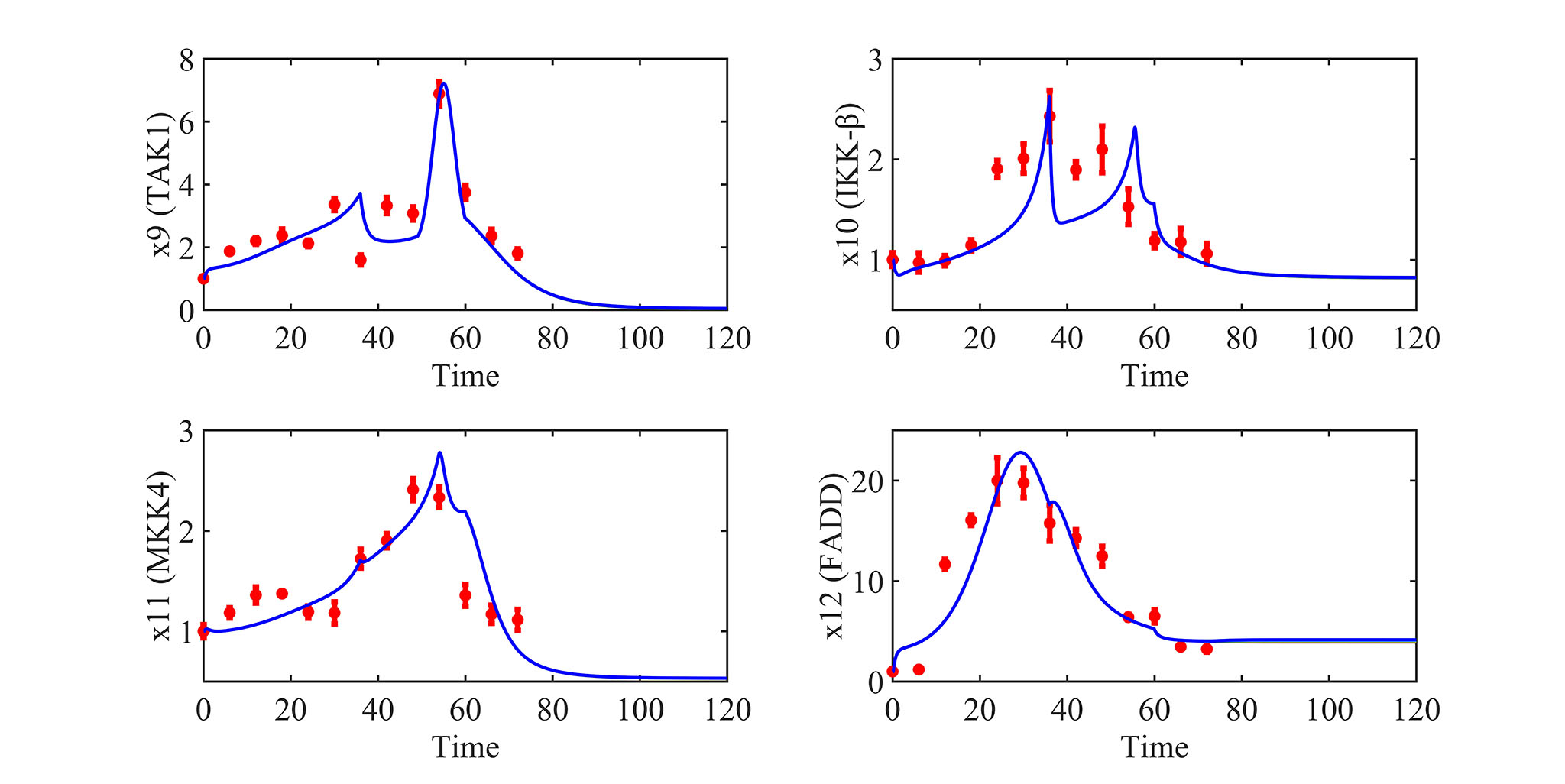

Supplement: Supplementary file 2 [file DataSheet1.zip › Supplementary material_image1/Parameter_a4(小)/3.jpg]

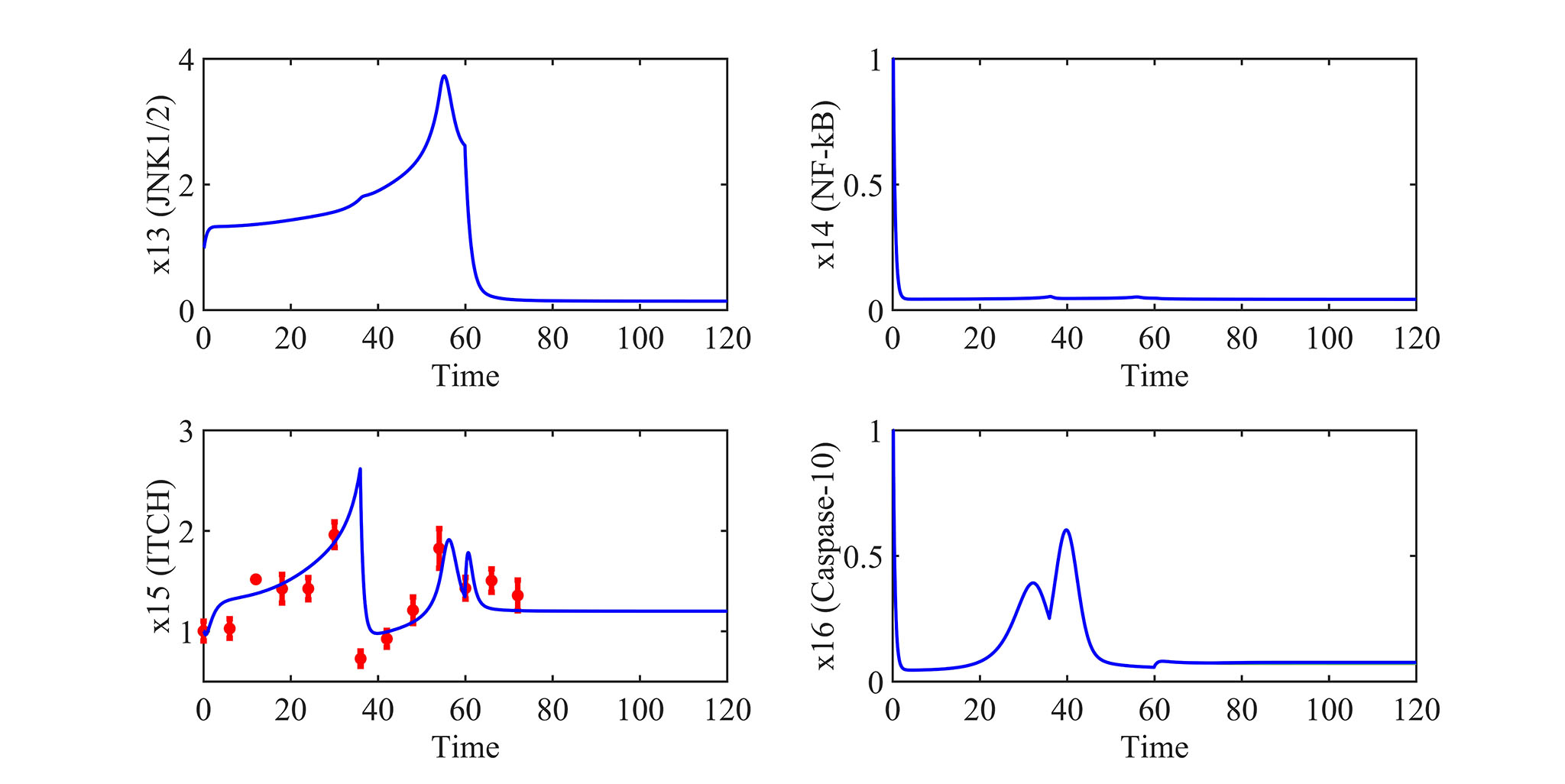

Supplement: Supplementary file 2 [file DataSheet1.zip › Supplementary material_image1/Parameter_a4(小)/4.jpg]

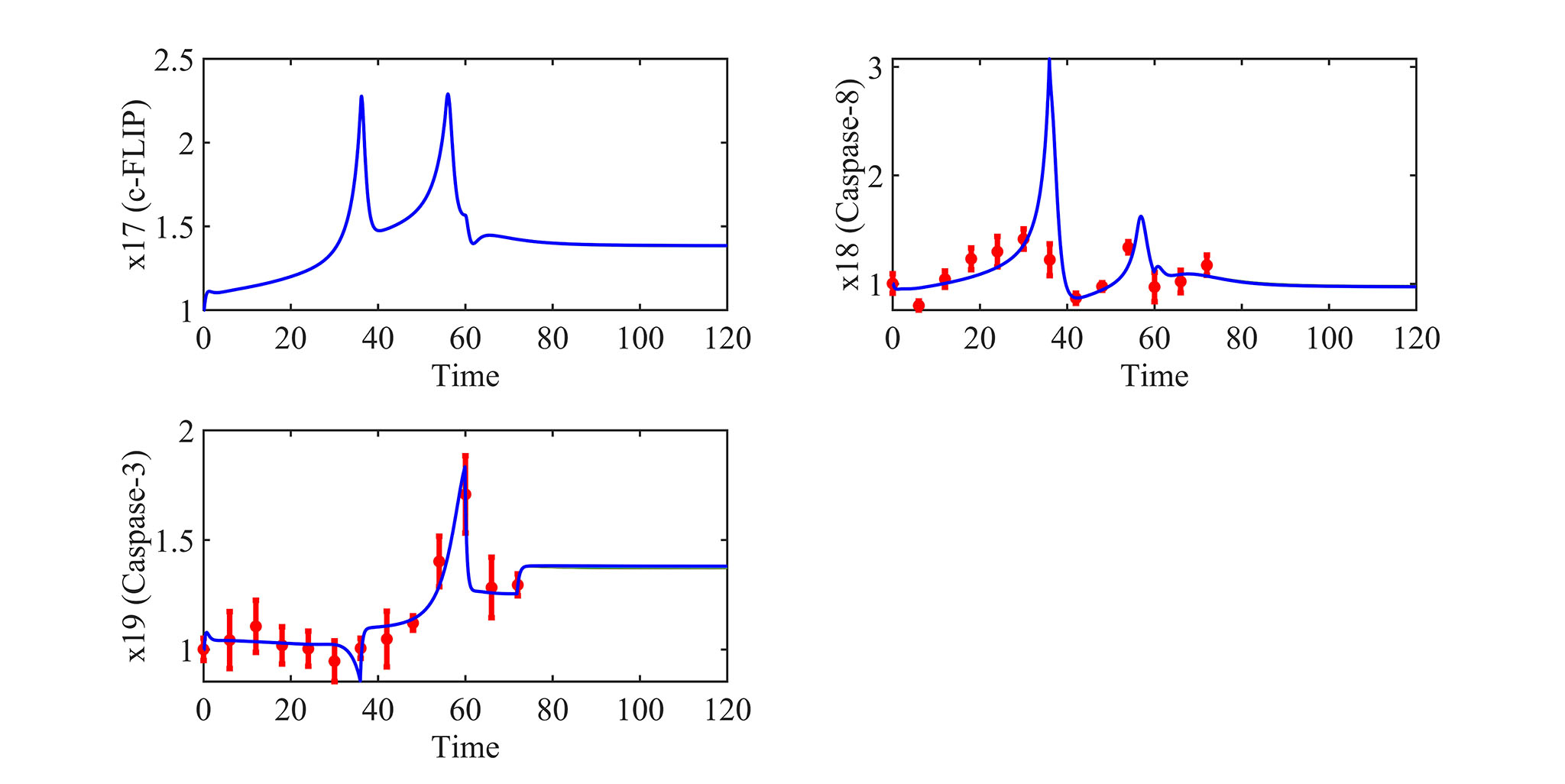

Supplement: Supplementary file 2 [file DataSheet1.zip › Supplementary material_image1/Parameter_a4(小)/5.jpg]

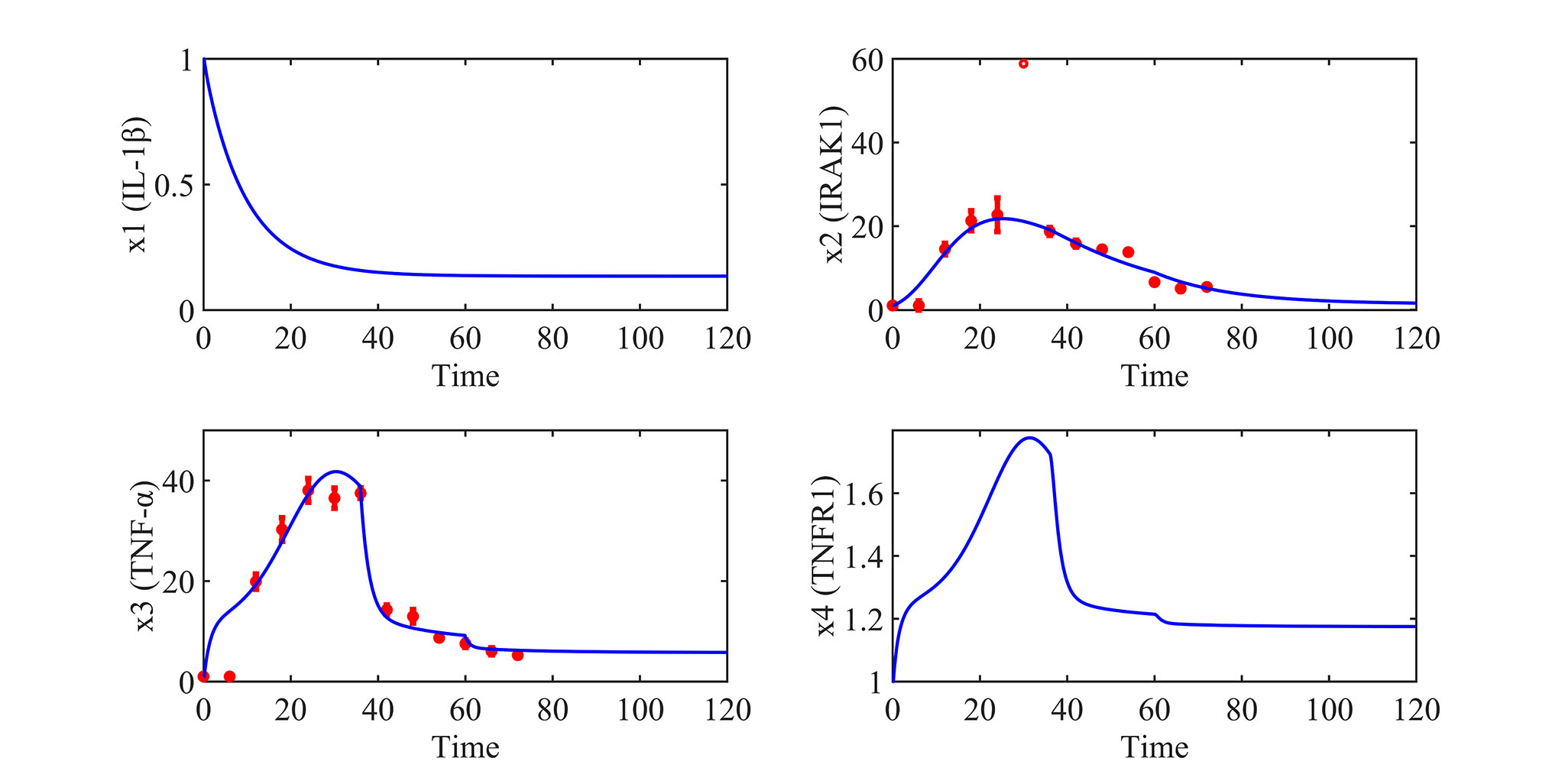

Supplement: Supplementary file 2 [file DataSheet1.zip › Supplementary material_image1/Parameter_a5(大)/1.jpg]

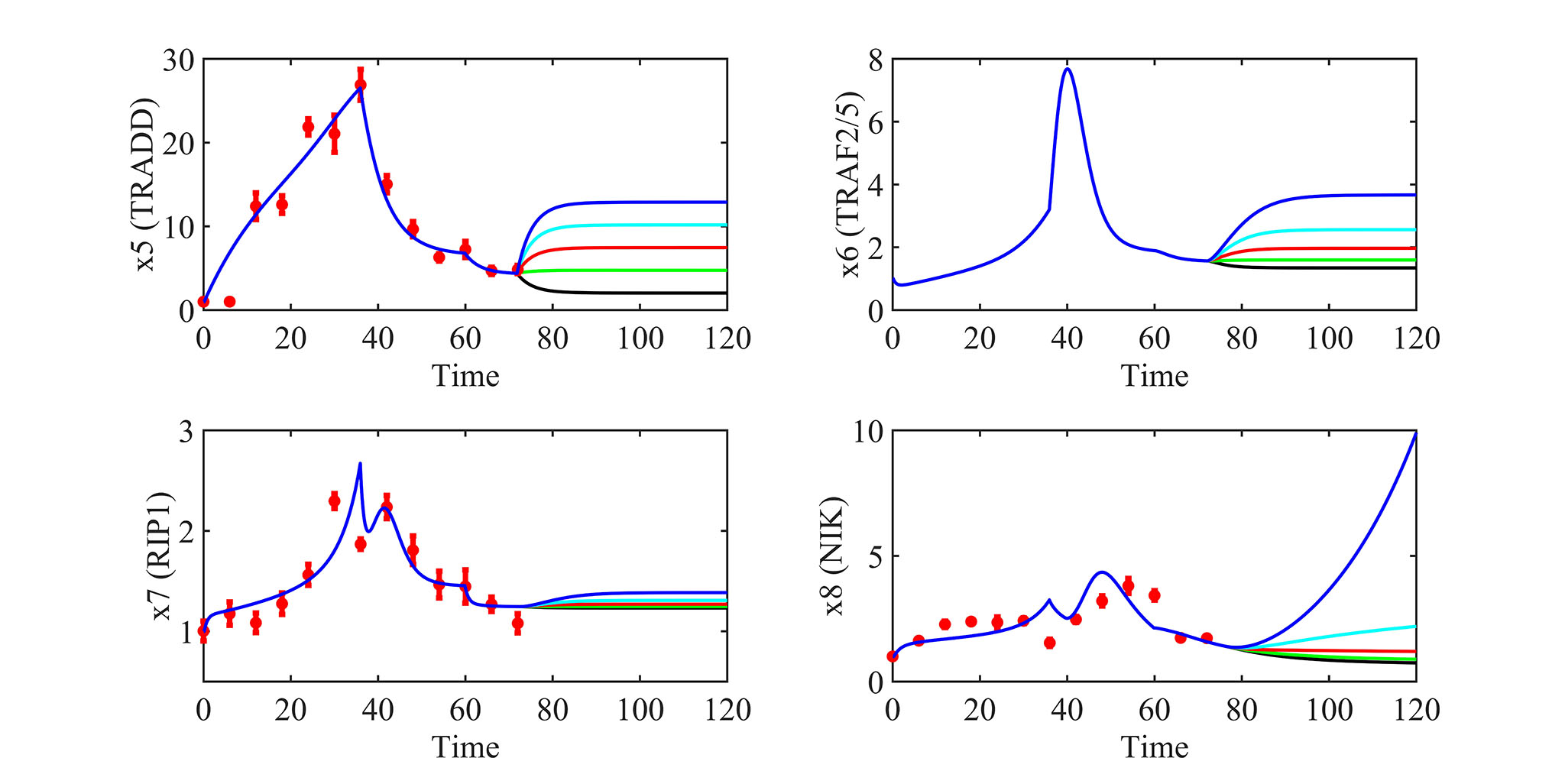

Supplement: Supplementary file 2 [file DataSheet1.zip › Supplementary material_image1/Parameter_a5(大)/2.jpg]

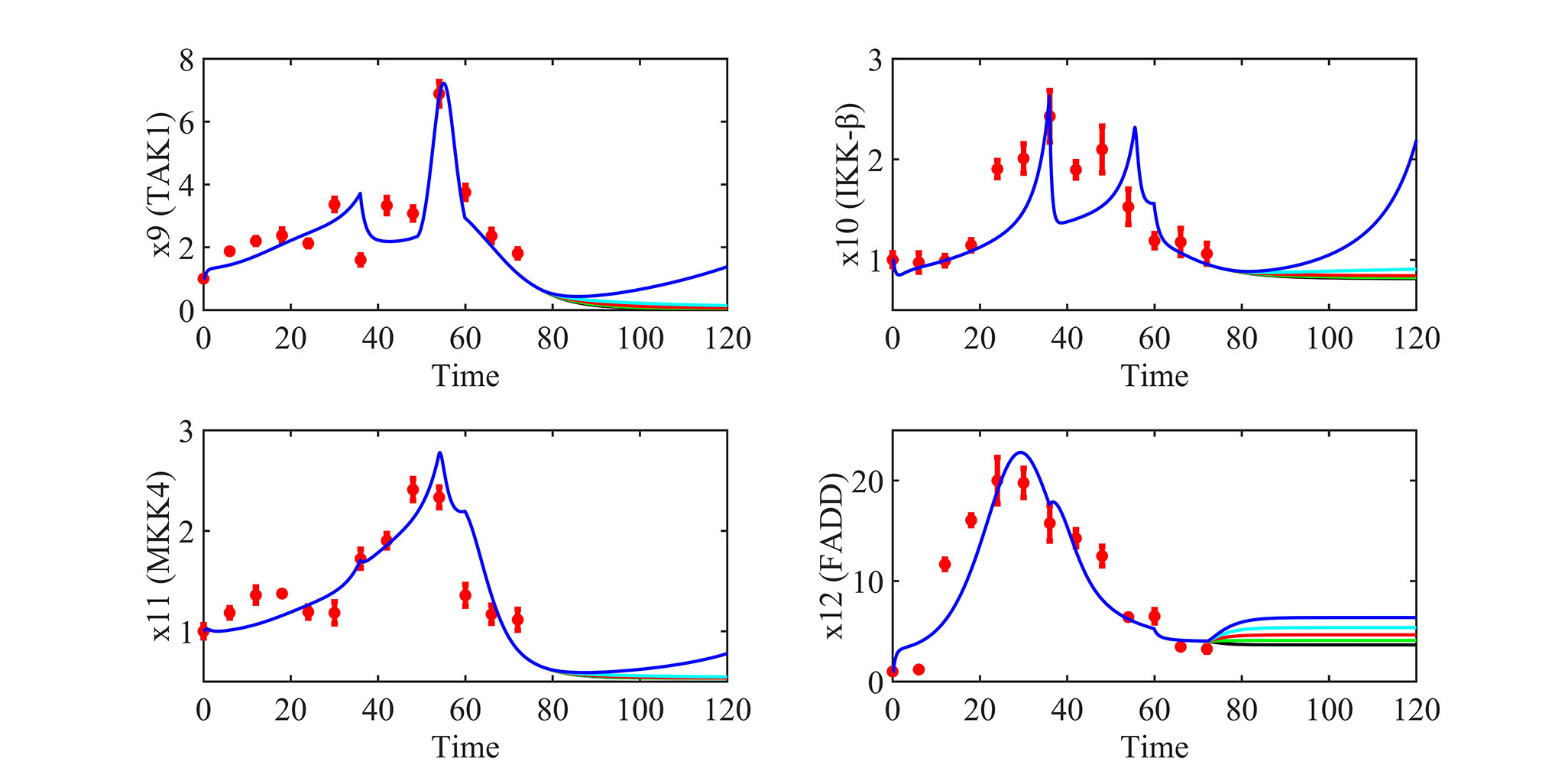

Supplement: Supplementary file 2 [file DataSheet1.zip › Supplementary material_image1/Parameter_a5(大)/3.jpg]

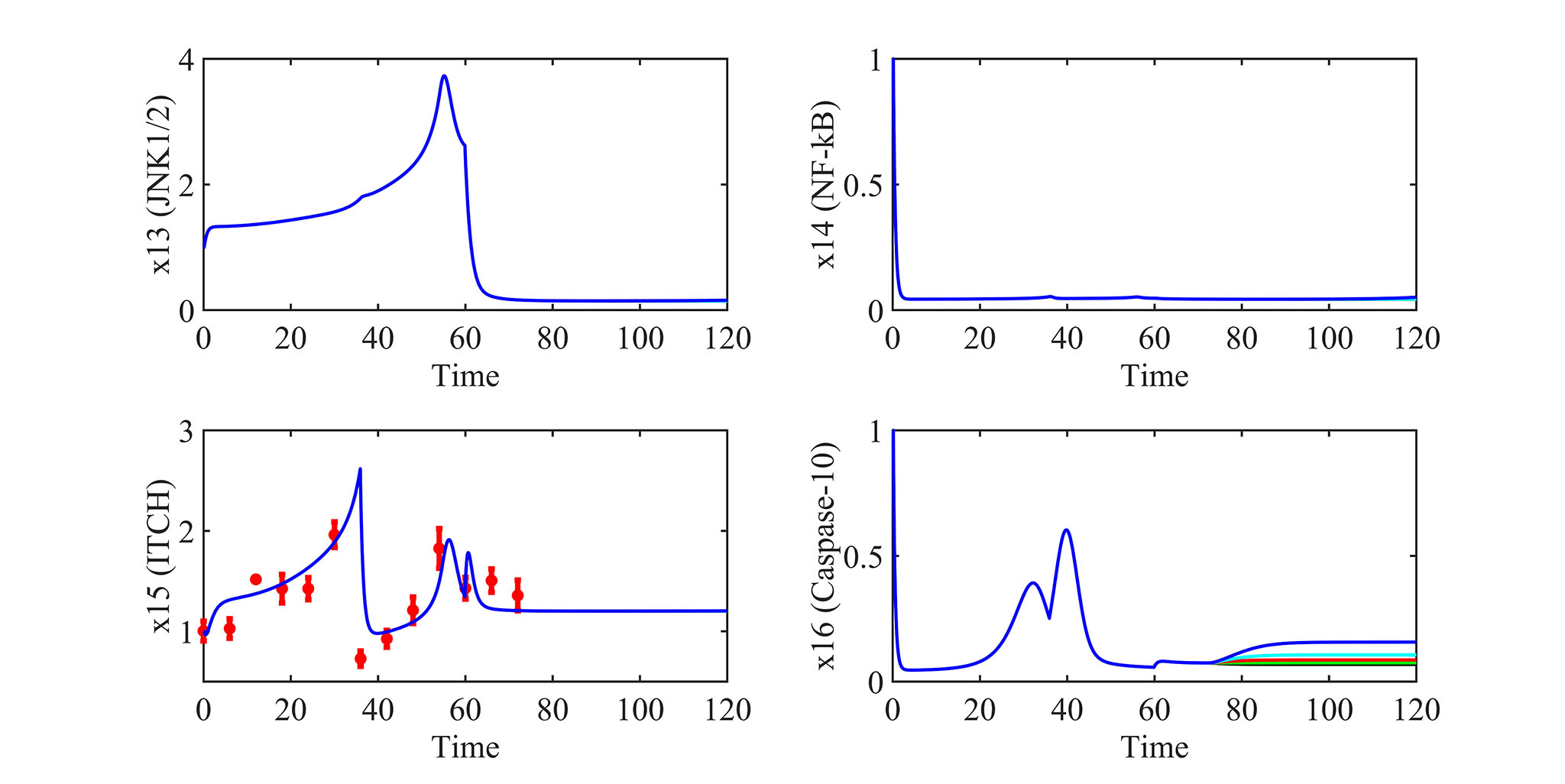

Supplement: Supplementary file 2 [file DataSheet1.zip › Supplementary material_image1/Parameter_a5(大)/4.jpg]

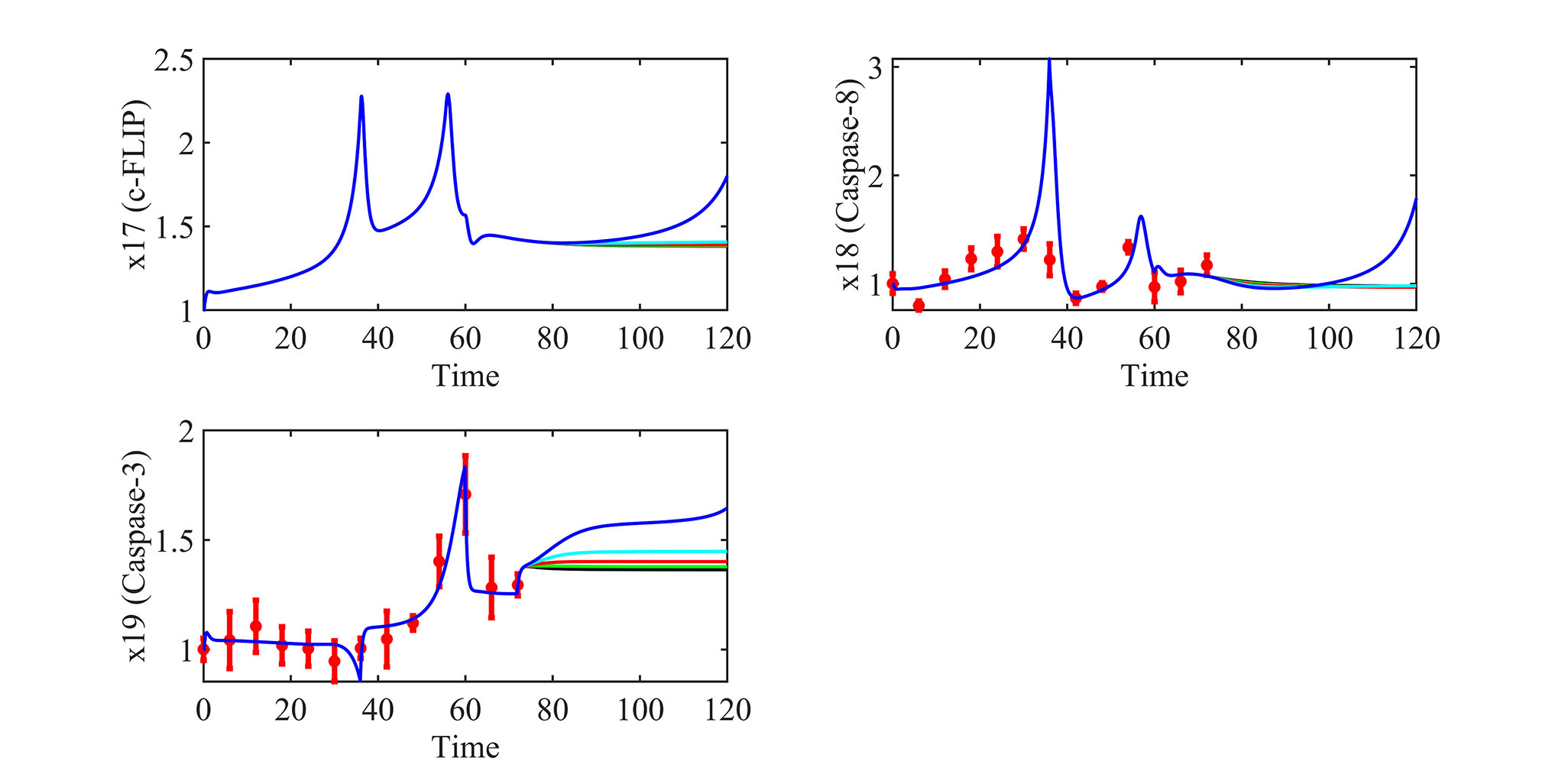

Supplement: Supplementary file 2 [file DataSheet1.zip › Supplementary material_image1/Parameter_a5(大)/5.jpg]

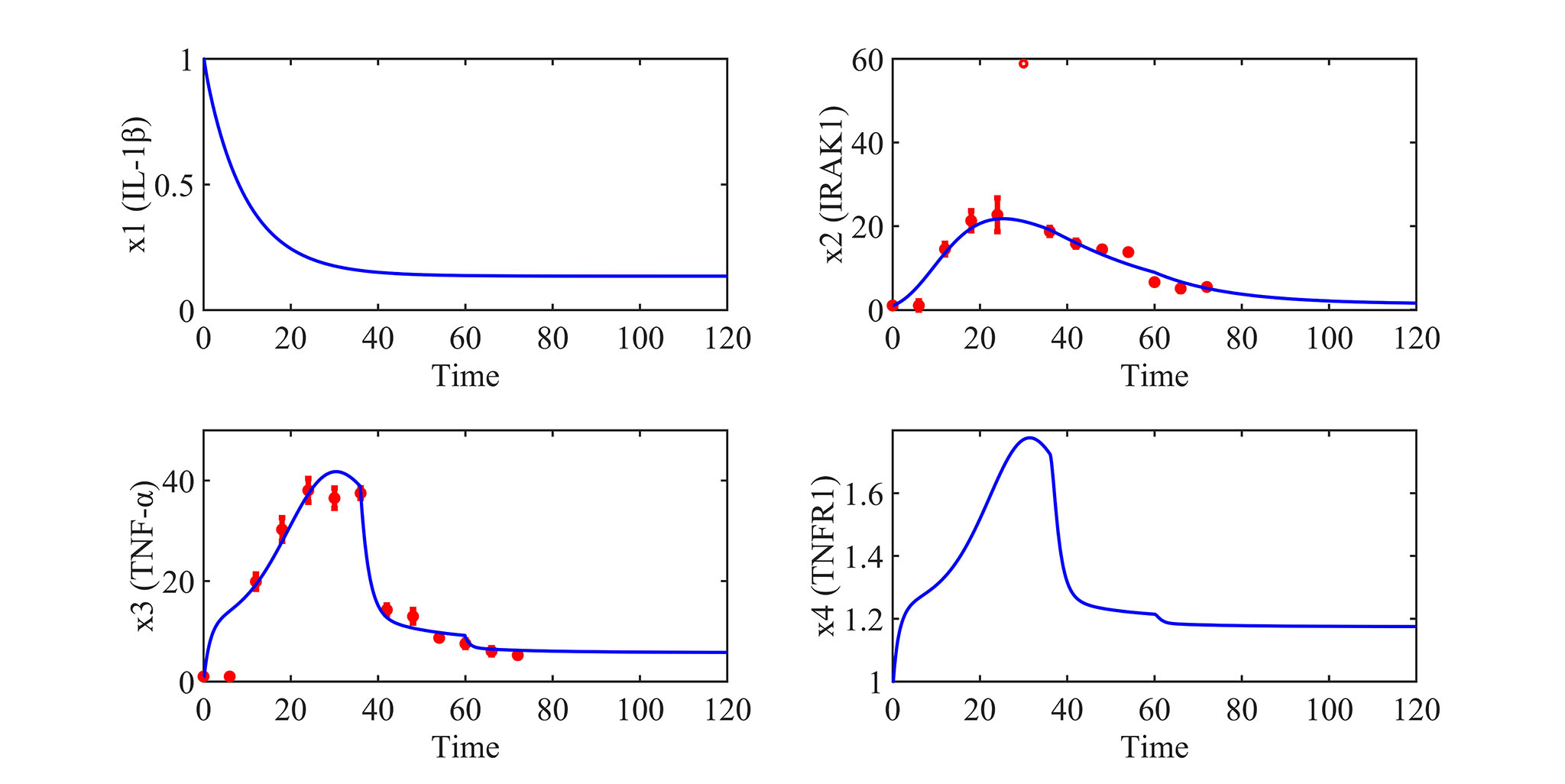

Supplement: Supplementary file 2 [file DataSheet1.zip › Supplementary material_image1/Parameter_a6(大)/1.jpg]

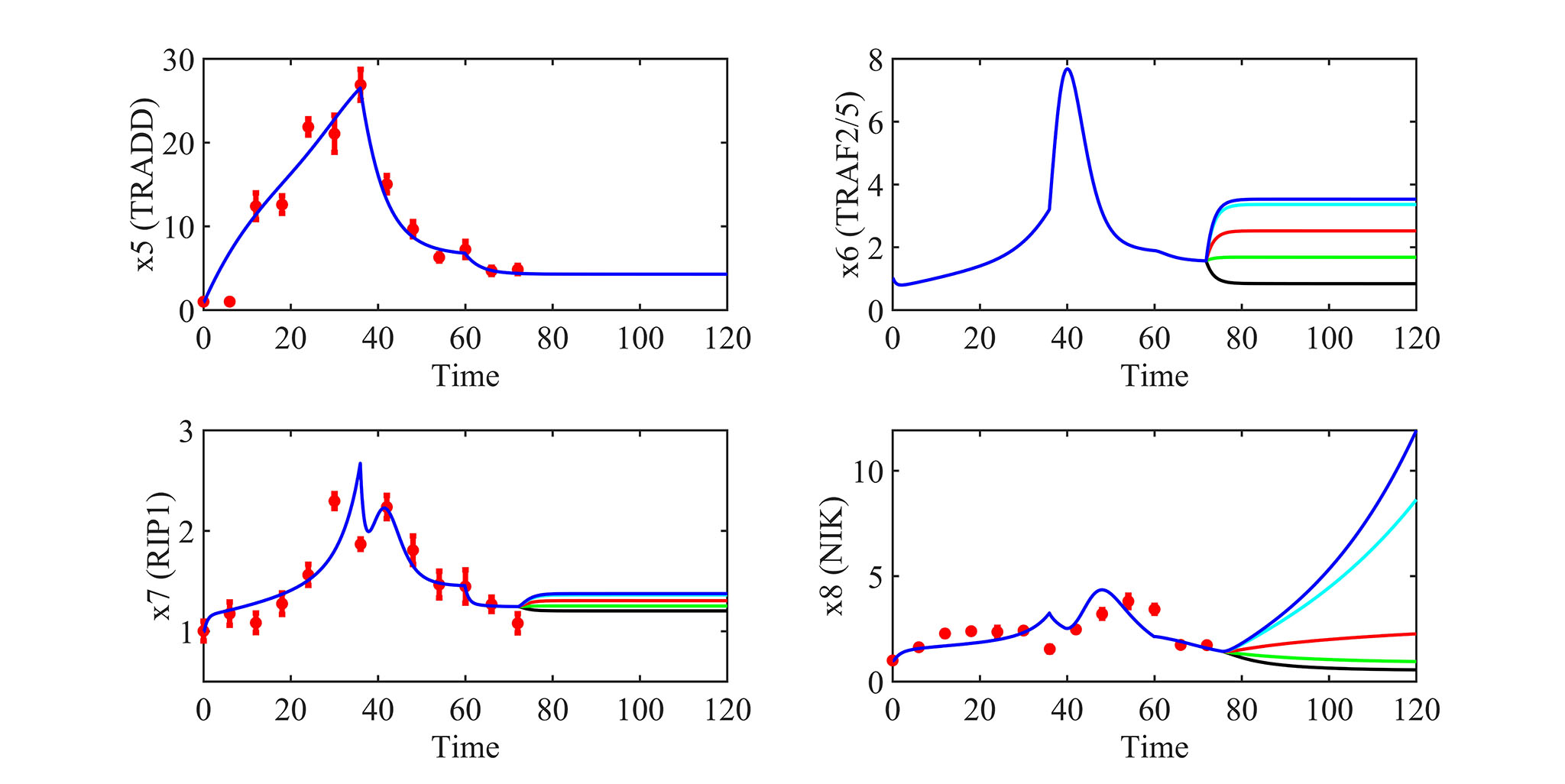

Supplement: Supplementary file 2 [file DataSheet1.zip › Supplementary material_image1/Parameter_a6(大)/2.jpg]

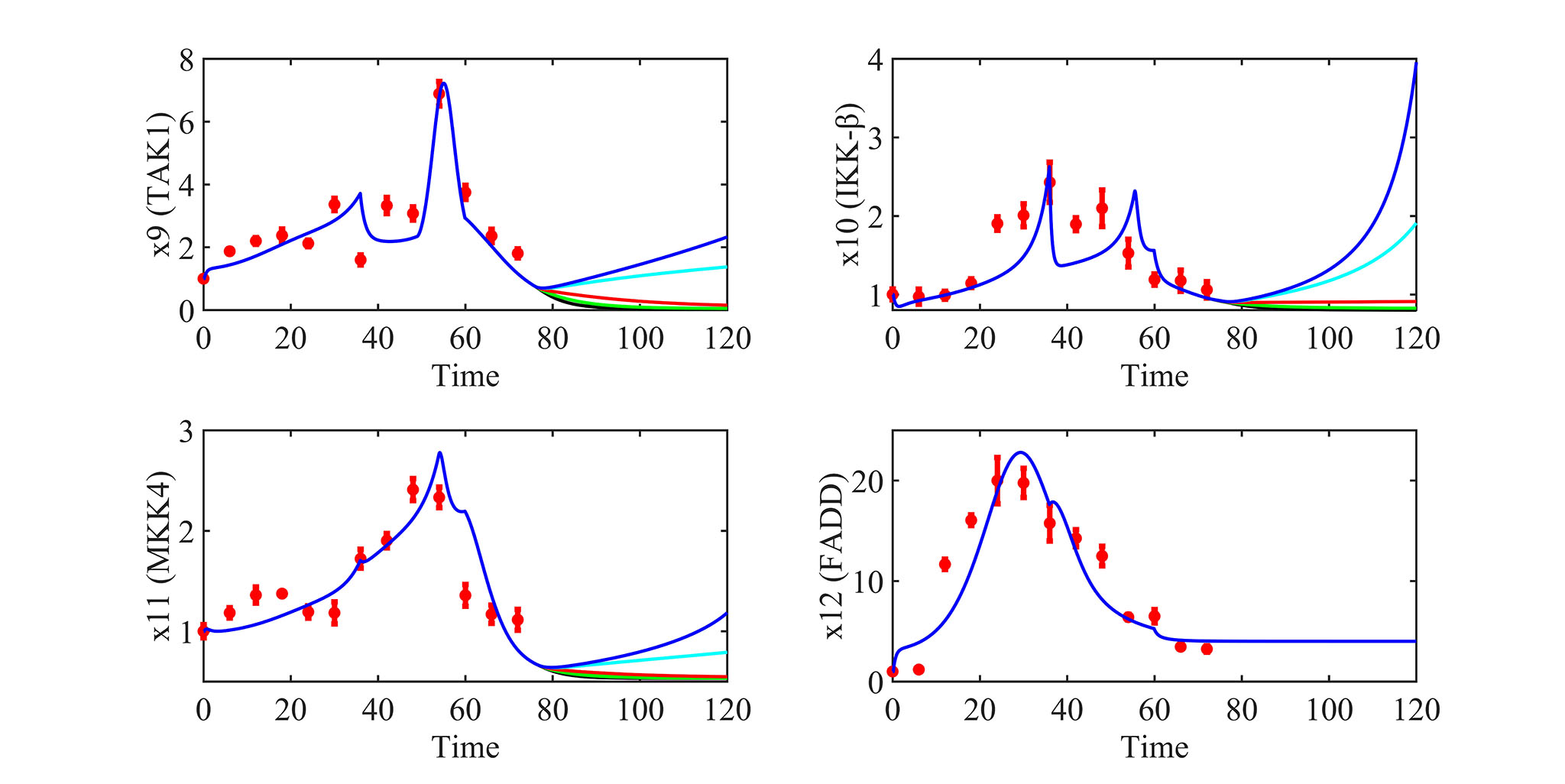

Supplement: Supplementary file 2 [file DataSheet1.zip › Supplementary material_image1/Parameter_a6(大)/3.jpg]

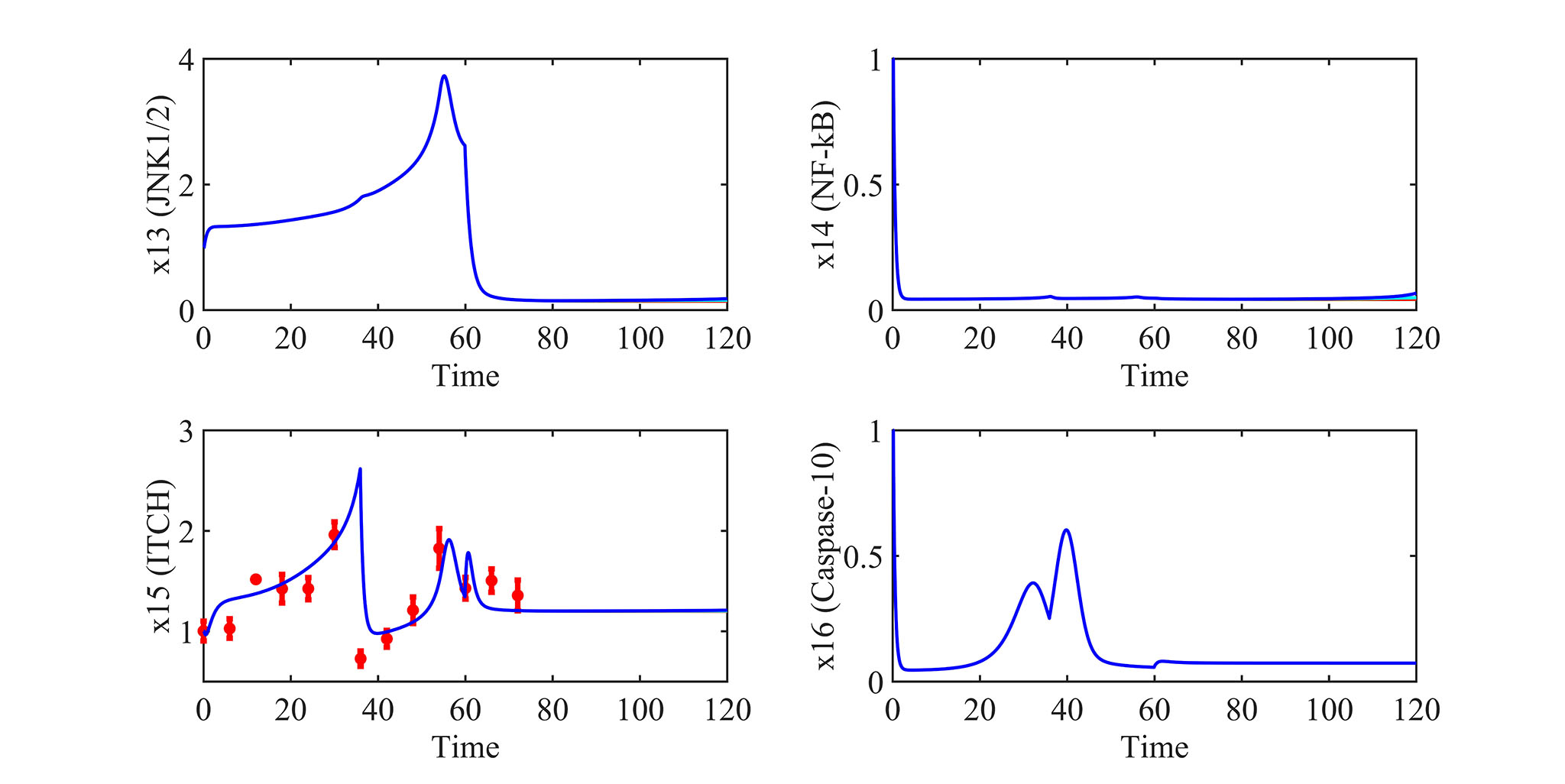

Supplement: Supplementary file 2 [file DataSheet1.zip › Supplementary material_image1/Parameter_a6(大)/4.jpg]

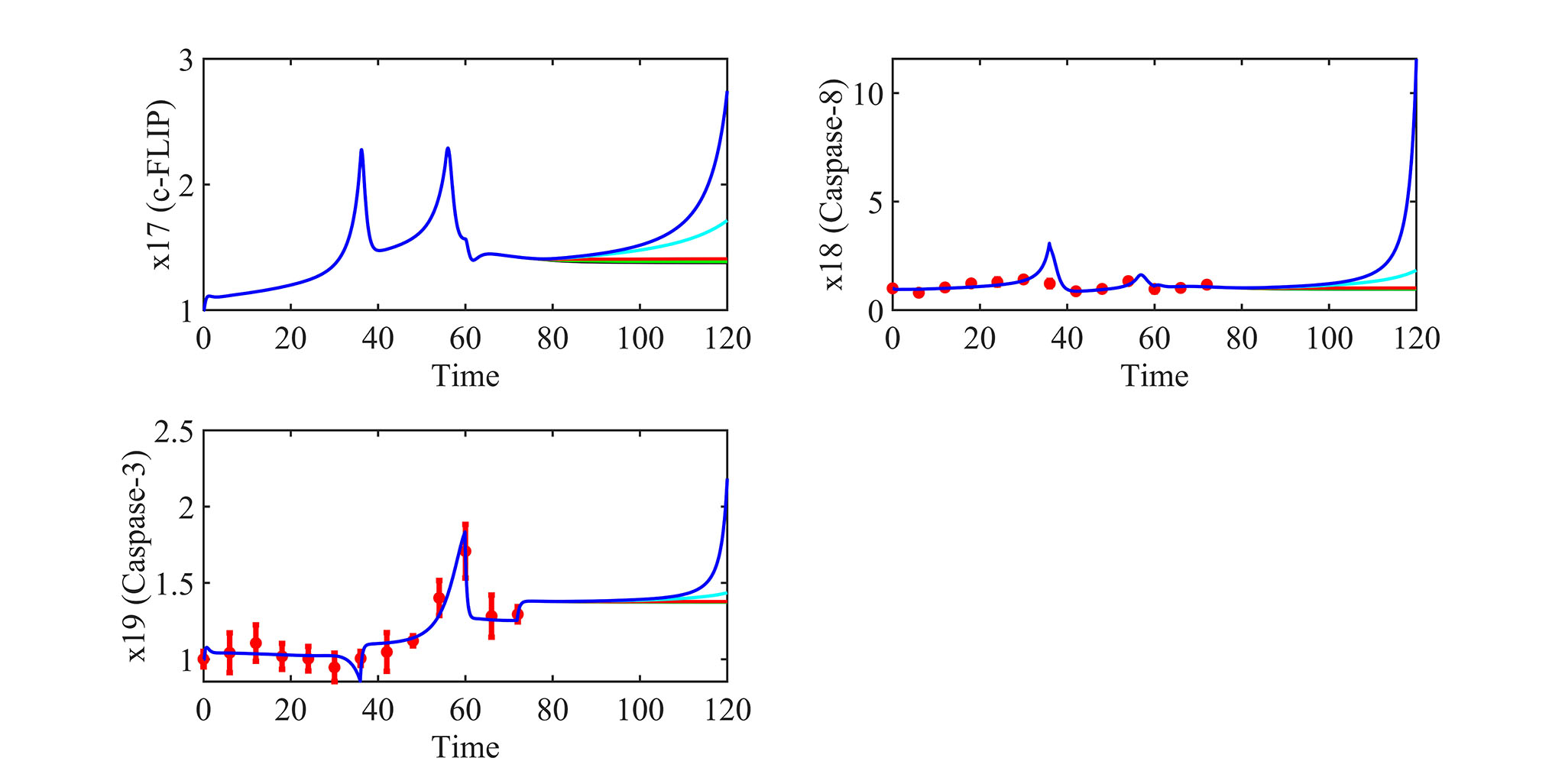

Supplement: Supplementary file 2 [file DataSheet1.zip › Supplementary material_image1/Parameter_a6(大)/5.jpg]

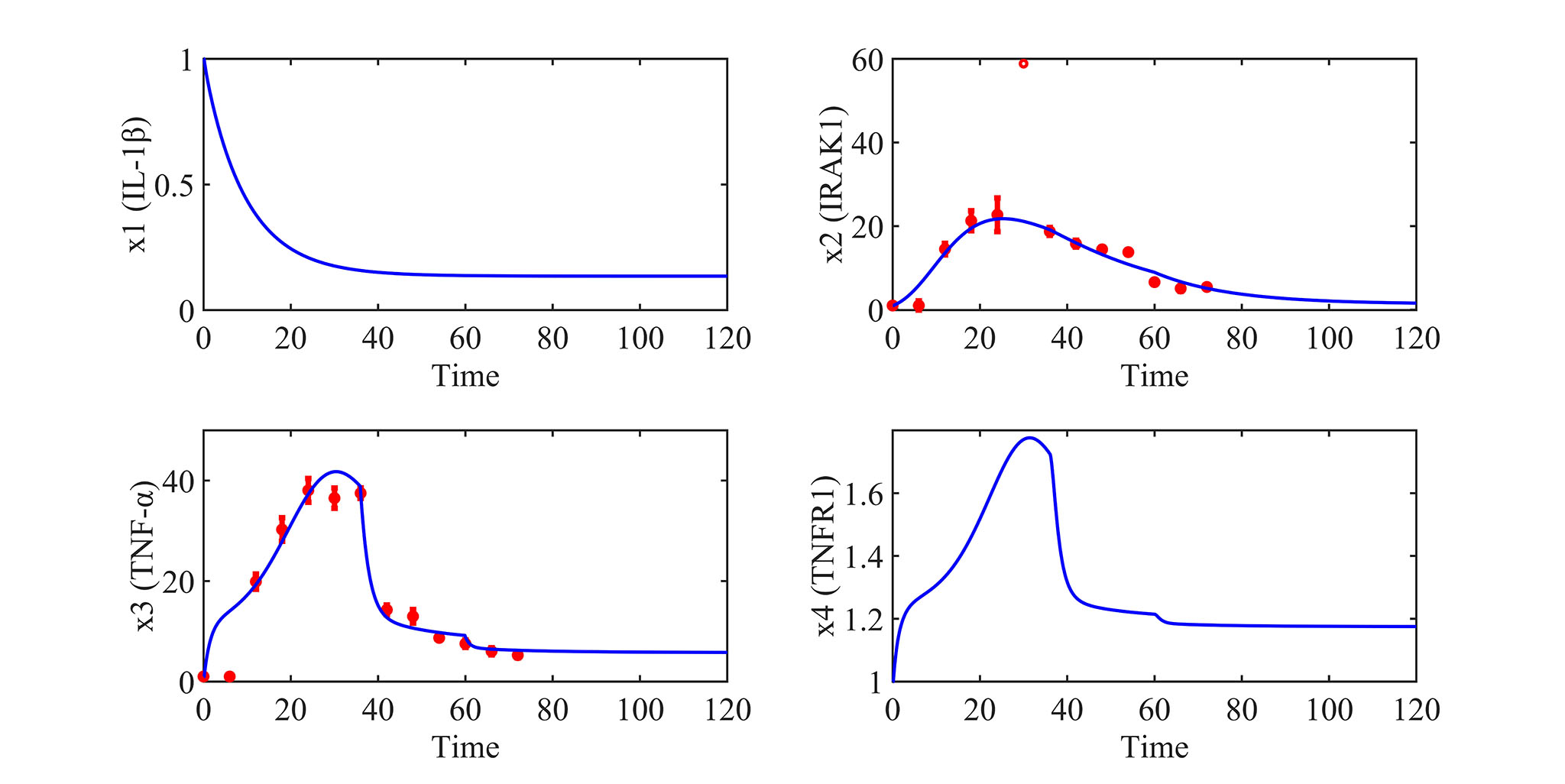

Supplement: Supplementary file 2 [file DataSheet1.zip › Supplementary material_image1/Parameter_a7(小)/1.jpg]

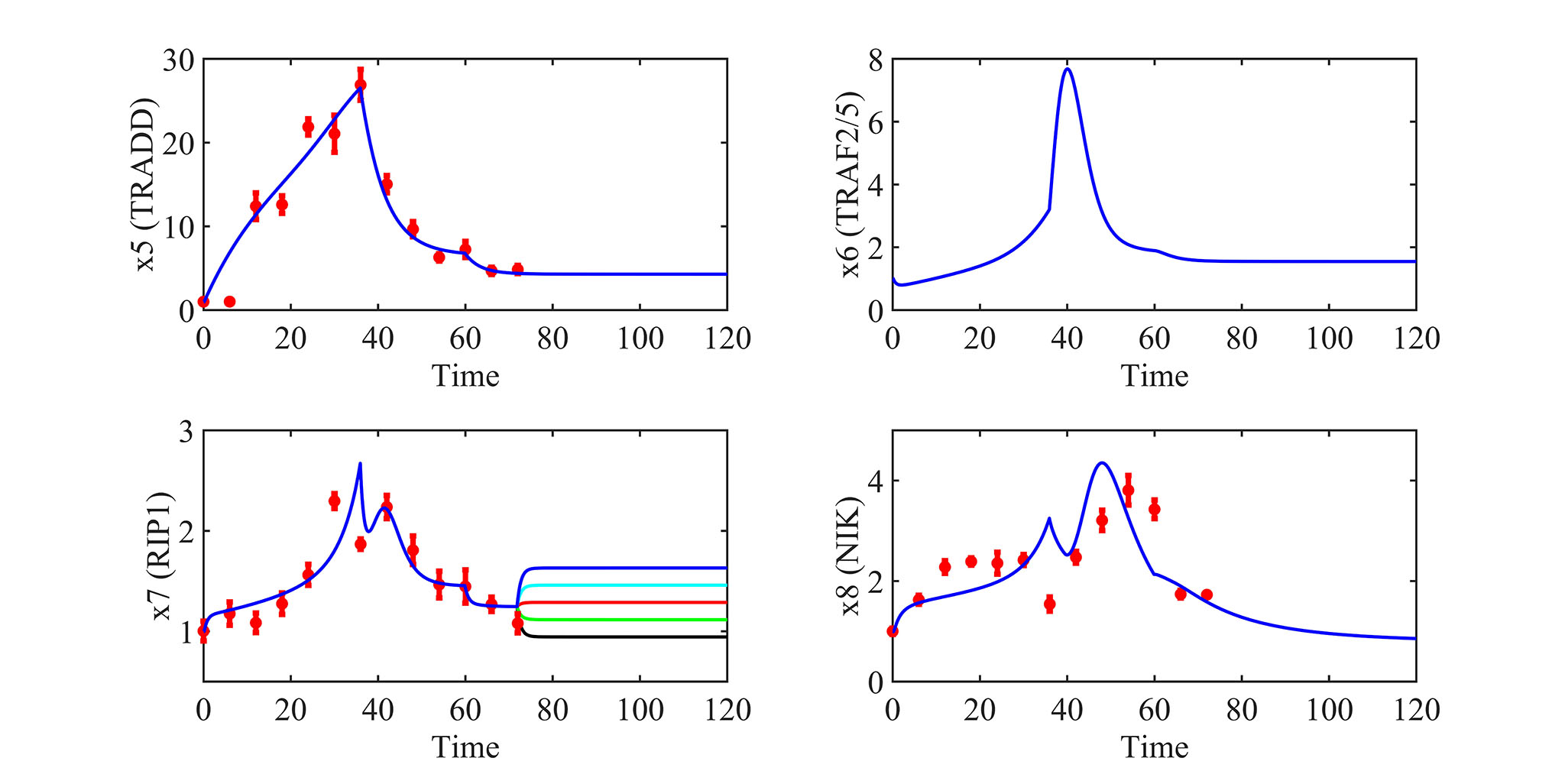

Supplement: Supplementary file 2 [file DataSheet1.zip › Supplementary material_image1/Parameter_a7(小)/2.jpg]

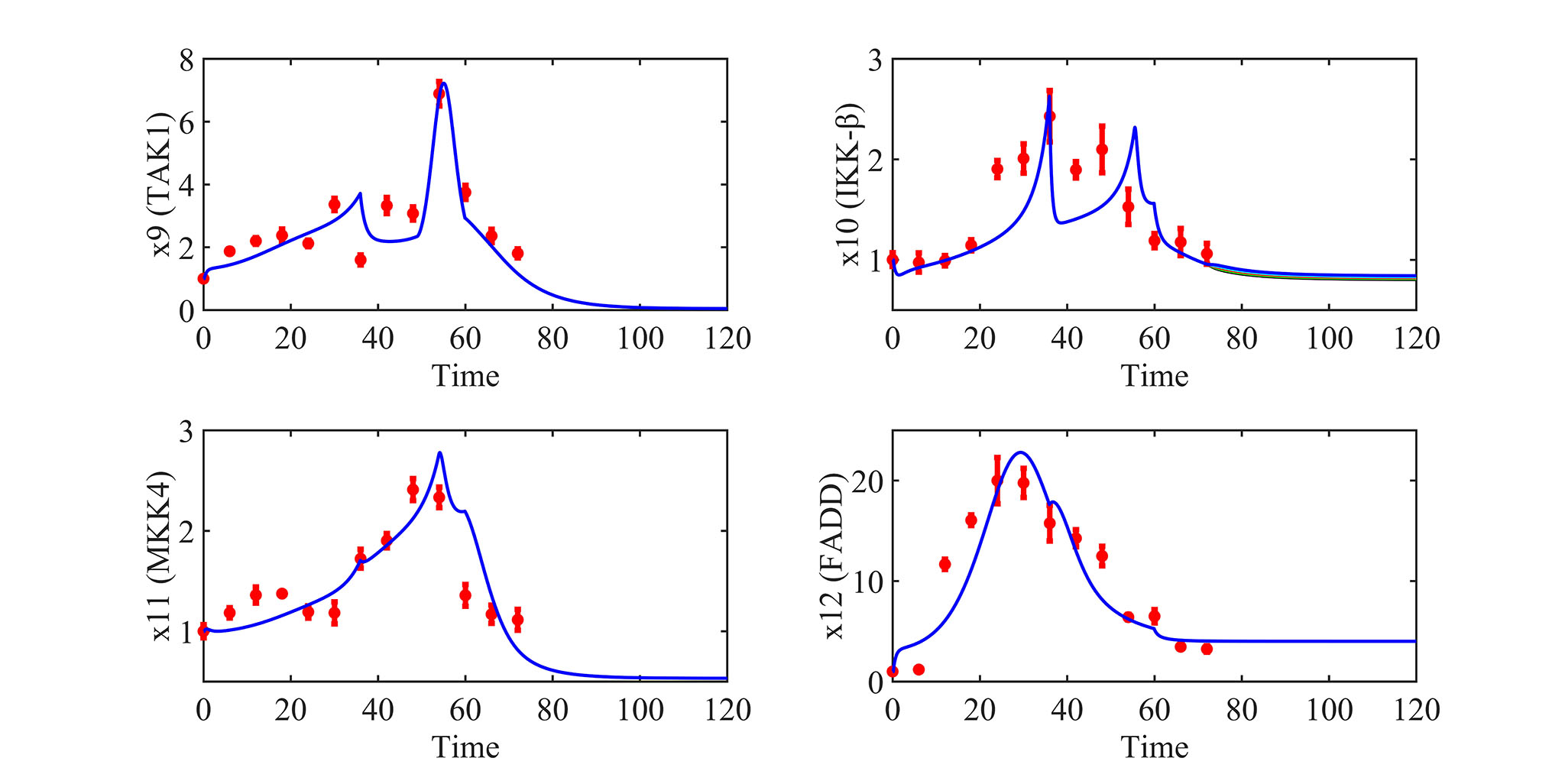

Supplement: Supplementary file 2 [file DataSheet1.zip › Supplementary material_image1/Parameter_a7(小)/3.jpg]

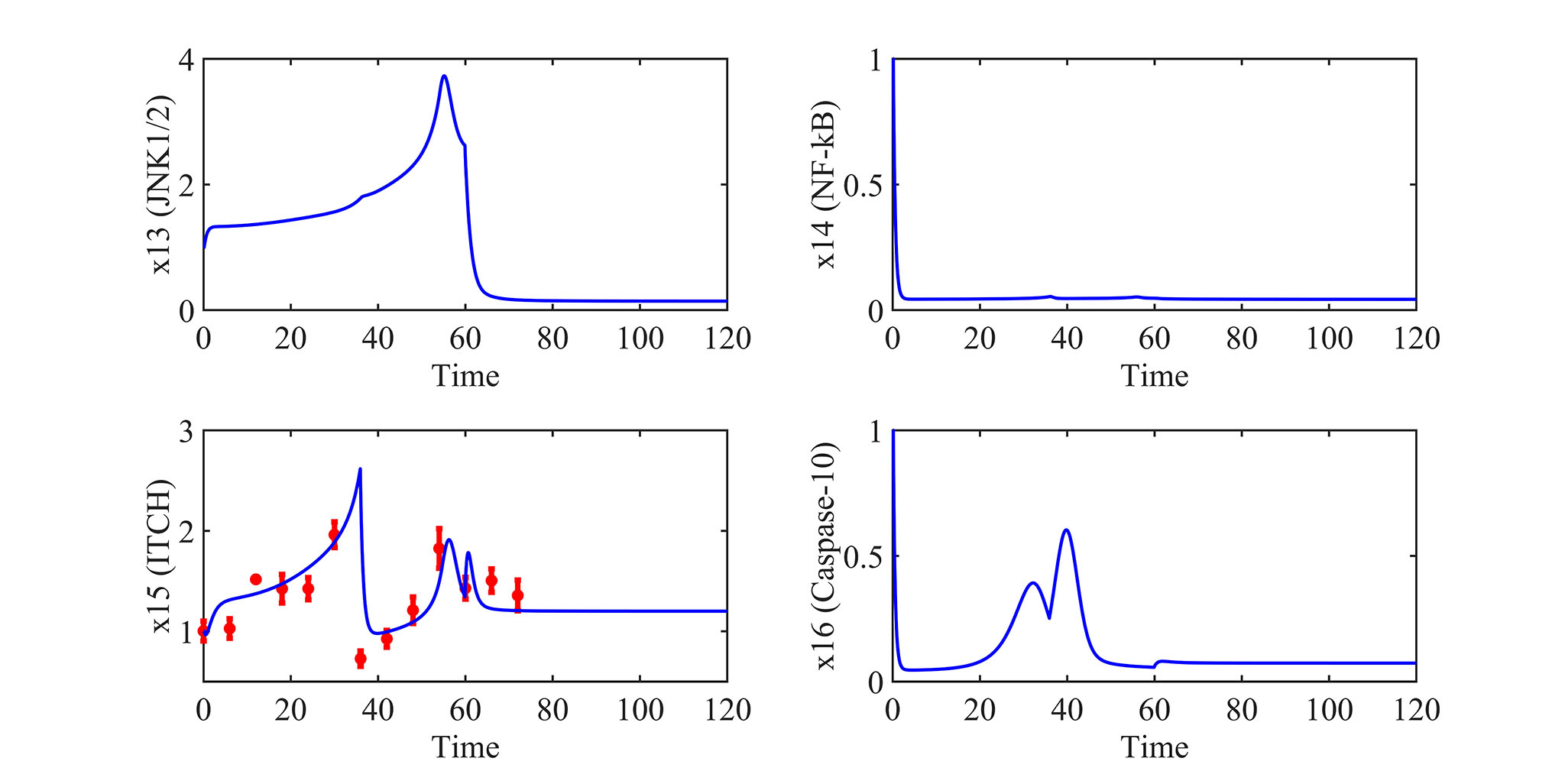

Supplement: Supplementary file 2 [file DataSheet1.zip › Supplementary material_image1/Parameter_a7(小)/4.jpg]

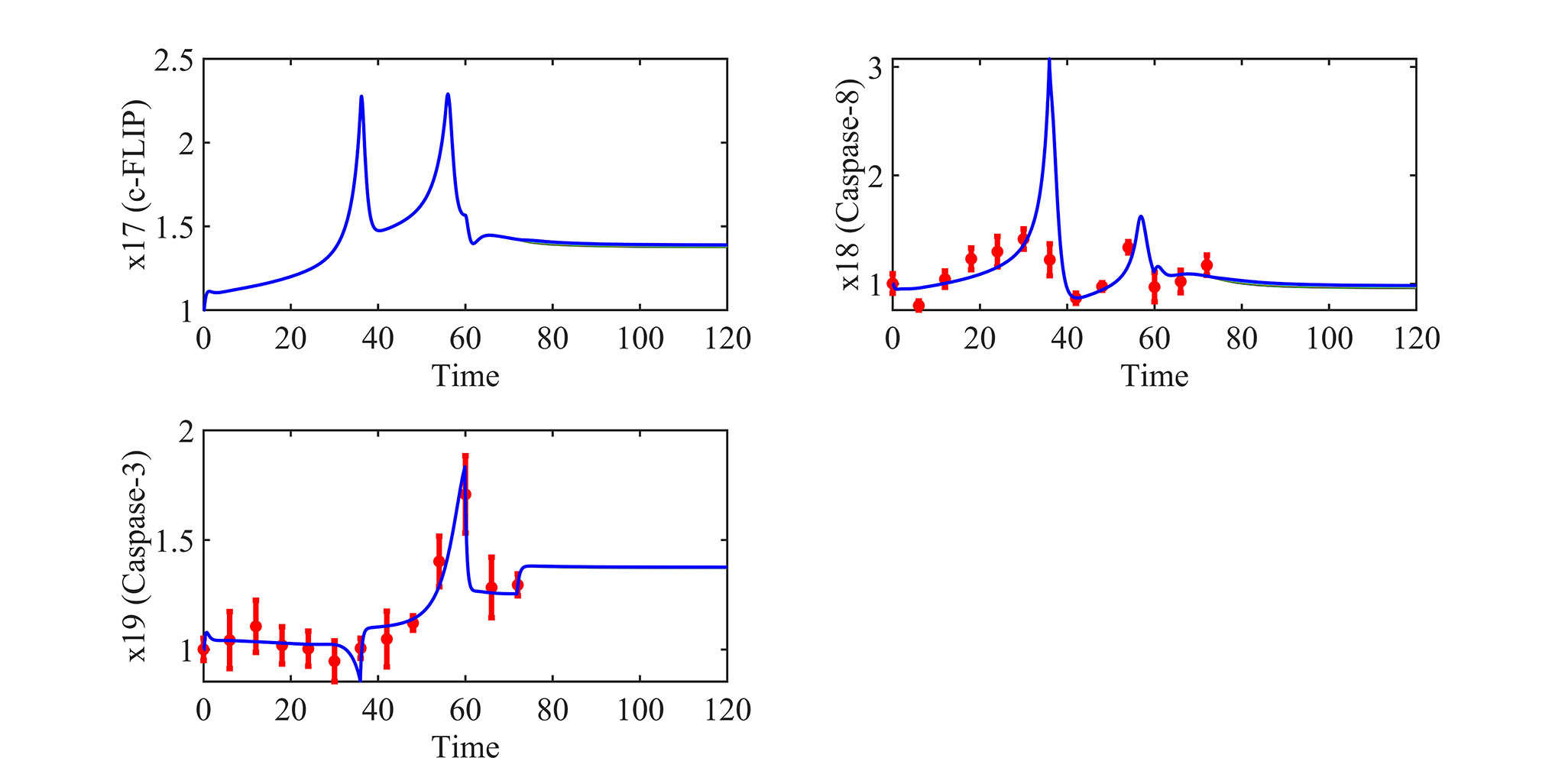

Supplement: Supplementary file 2 [file DataSheet1.zip › Supplementary material_image1/Parameter_a7(小)/5.jpg]

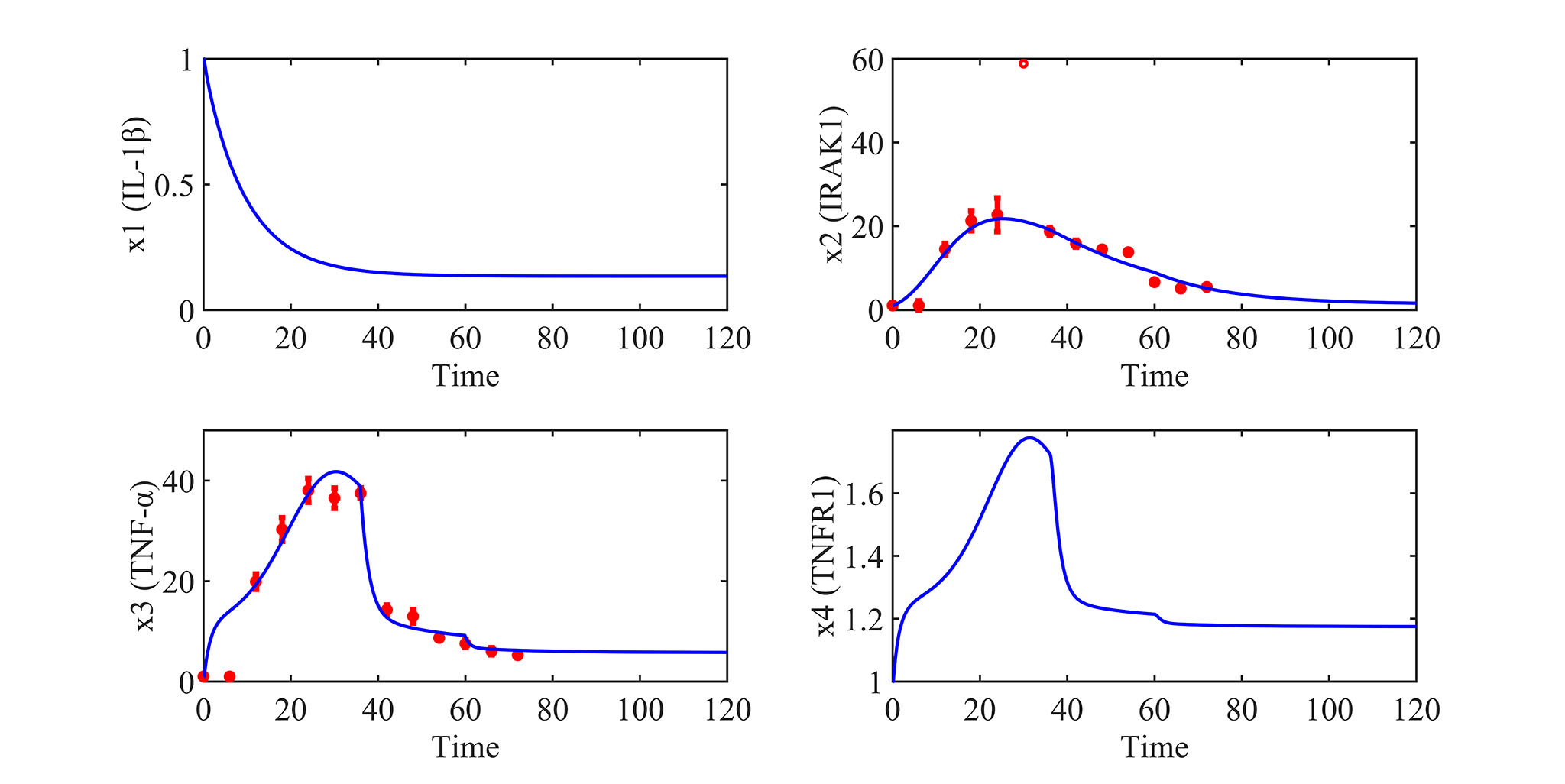

Supplement: Supplementary file 2 [file DataSheet1.zip › Supplementary material_image1/Parameter_a8(小)/1.jpg]

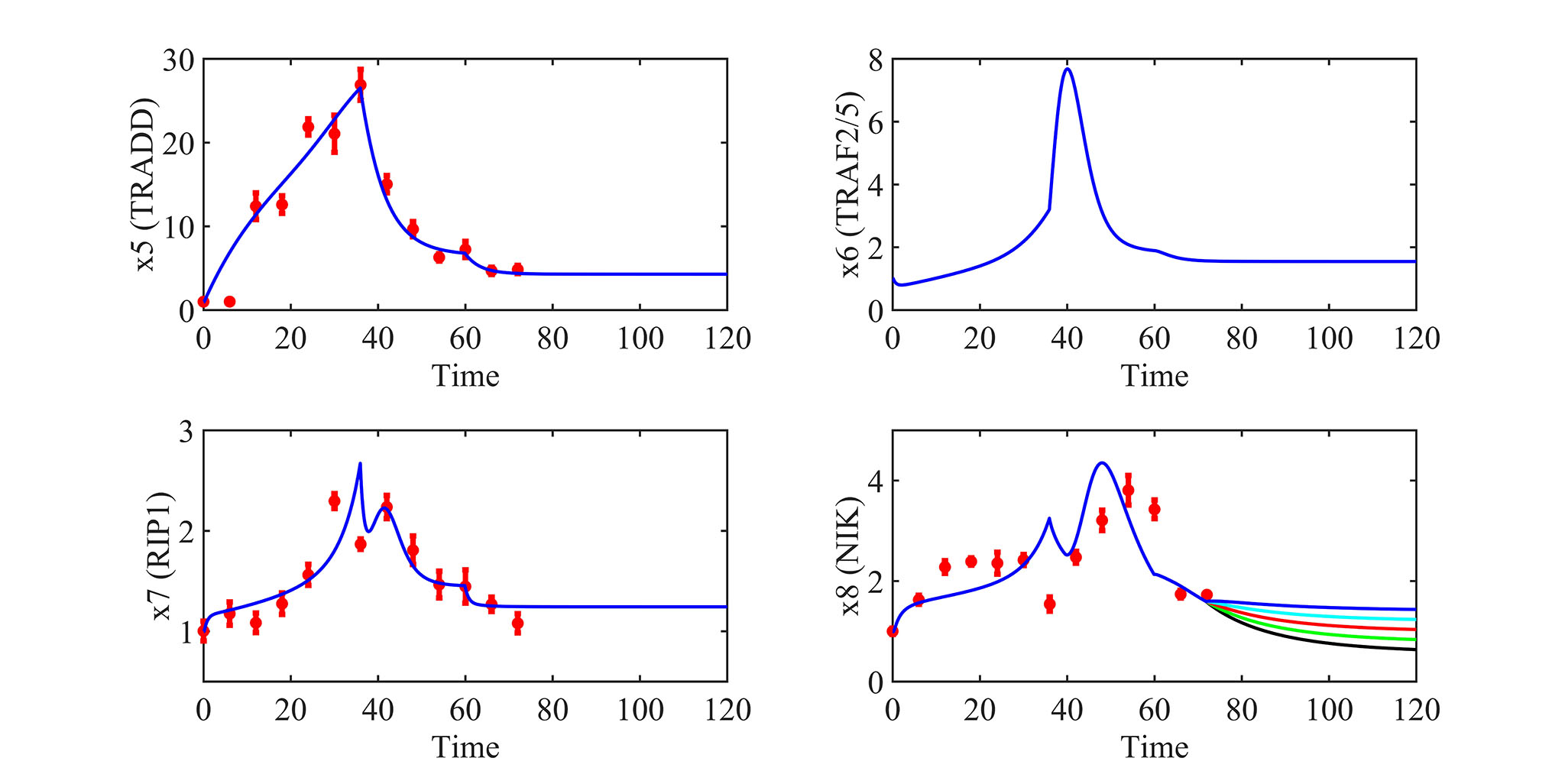

Supplement: Supplementary file 2 [file DataSheet1.zip › Supplementary material_image1/Parameter_a8(小)/2.jpg]

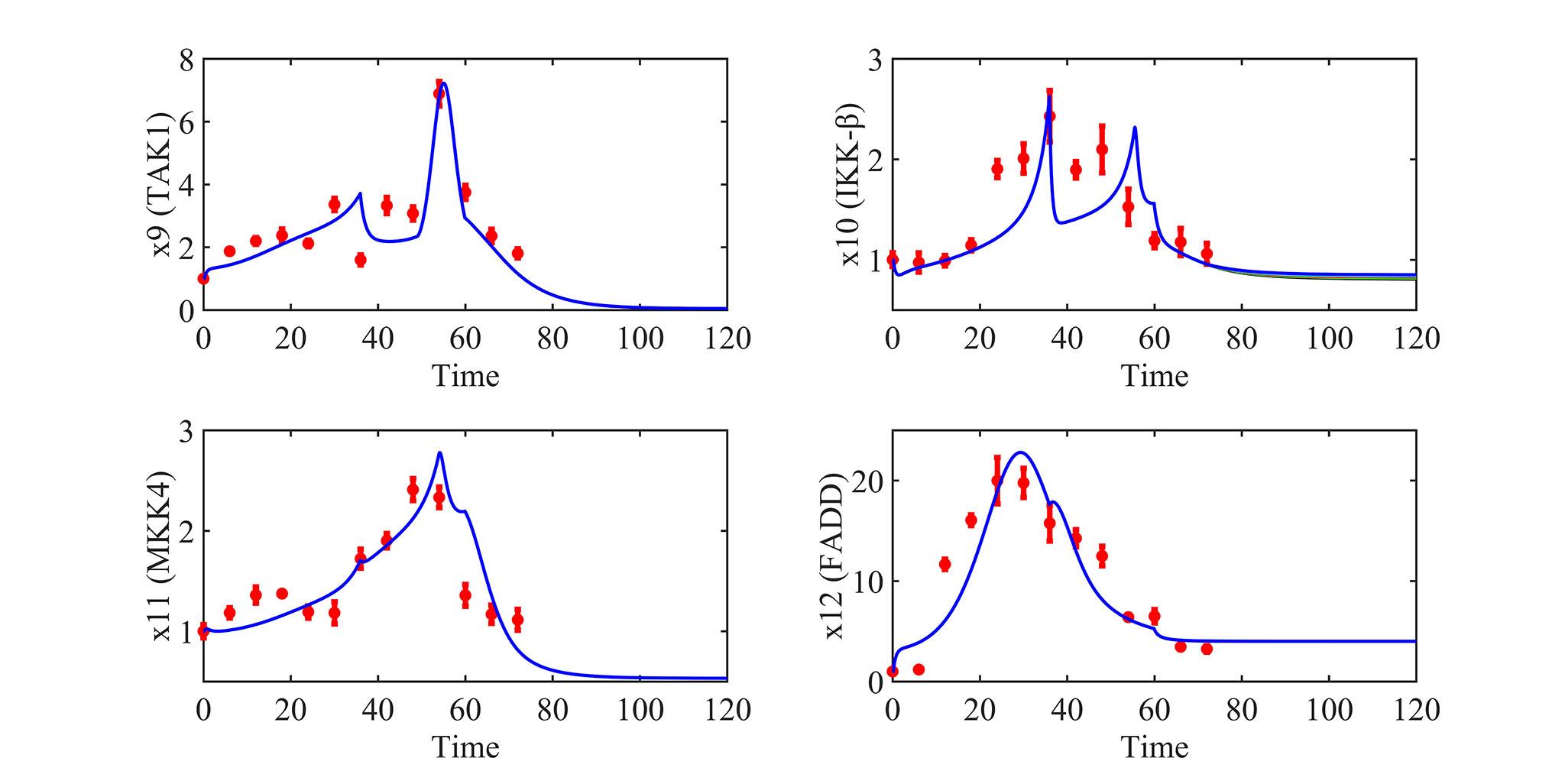

Supplement: Supplementary file 2 [file DataSheet1.zip › Supplementary material_image1/Parameter_a8(小)/3.jpg]

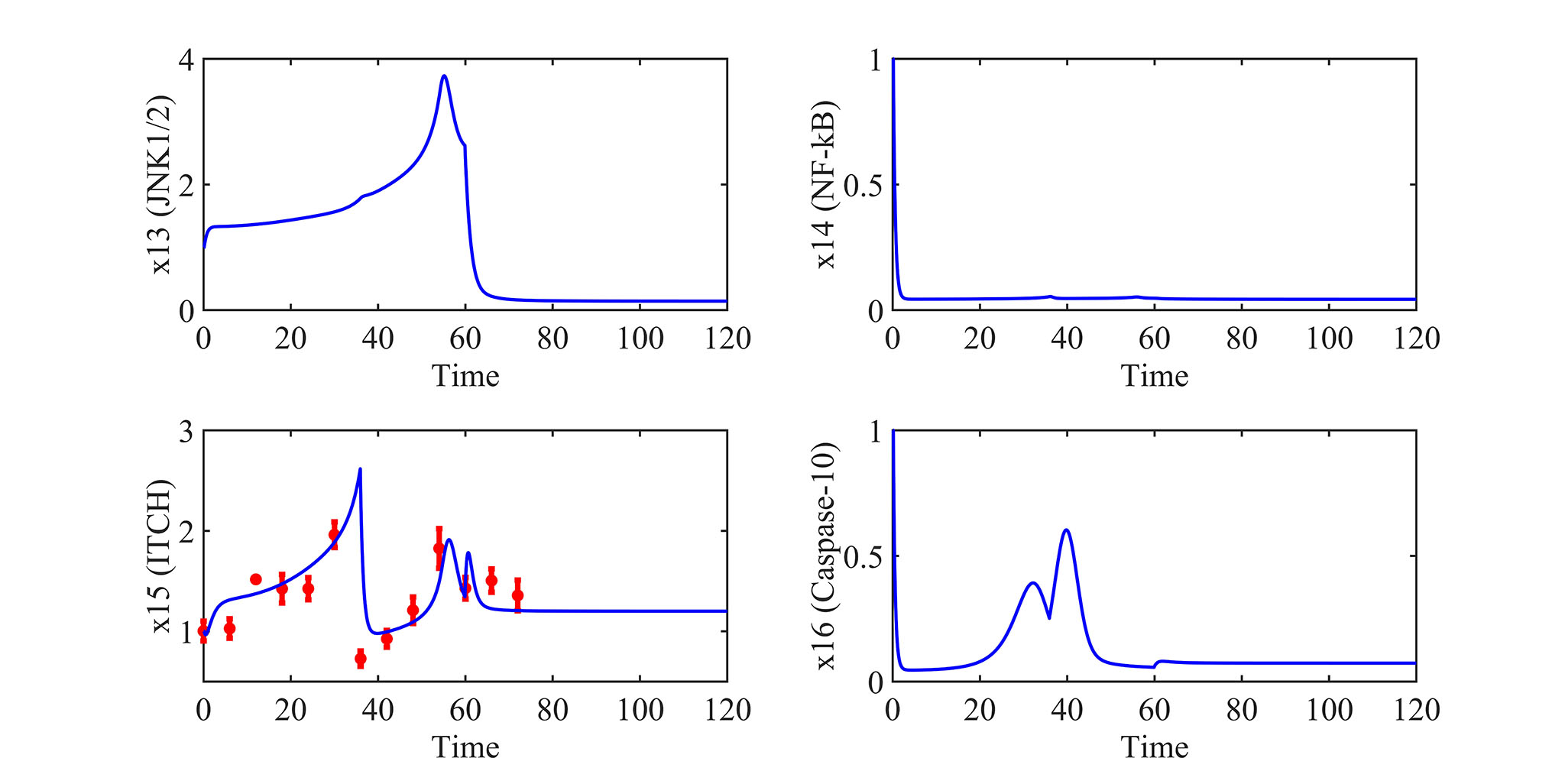

Supplement: Supplementary file 2 [file DataSheet1.zip › Supplementary material_image1/Parameter_a8(小)/4.jpg]

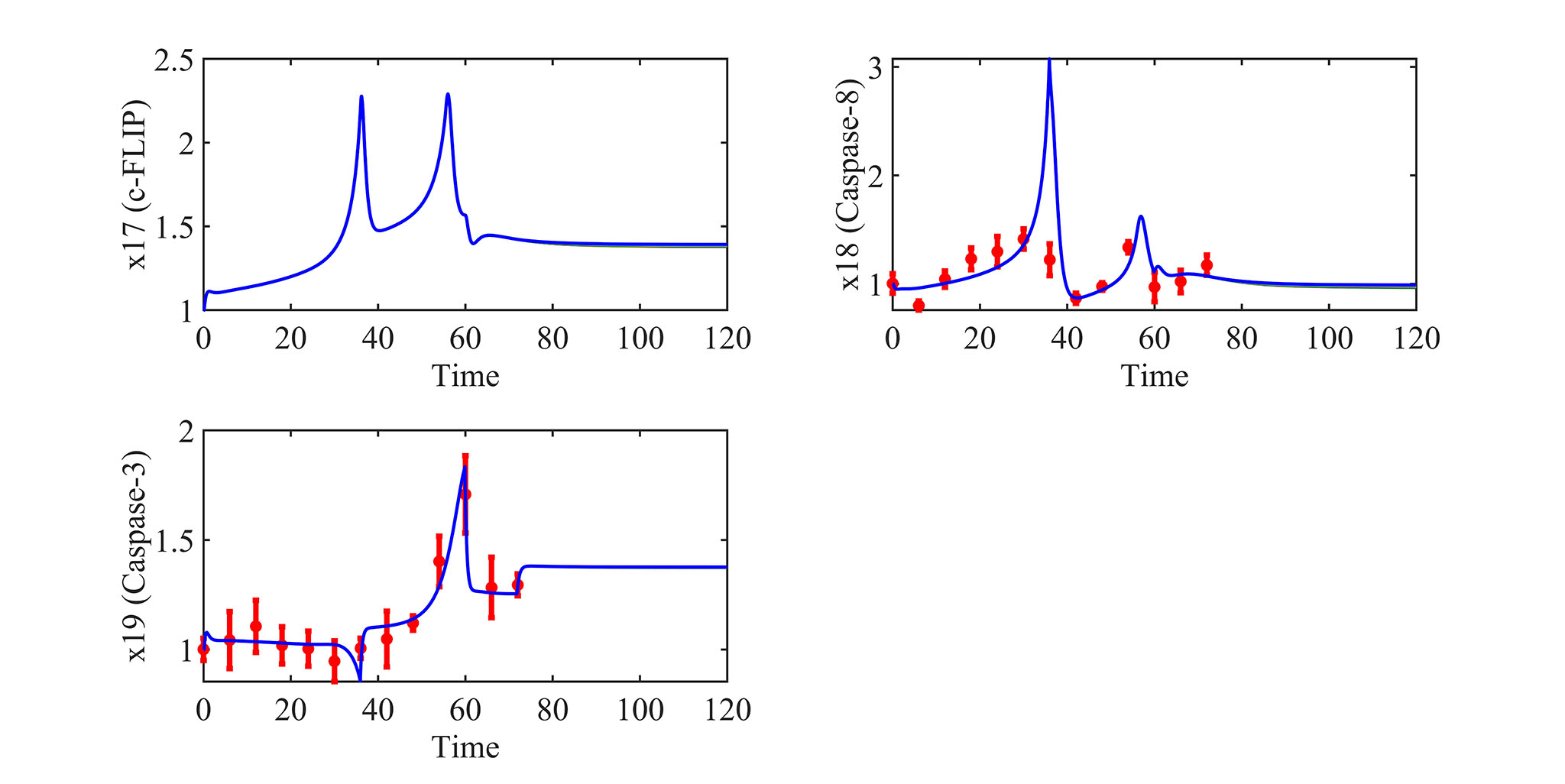

Supplement: Supplementary file 2 [file DataSheet1.zip › Supplementary material_image1/Parameter_a8(小)/5.jpg]

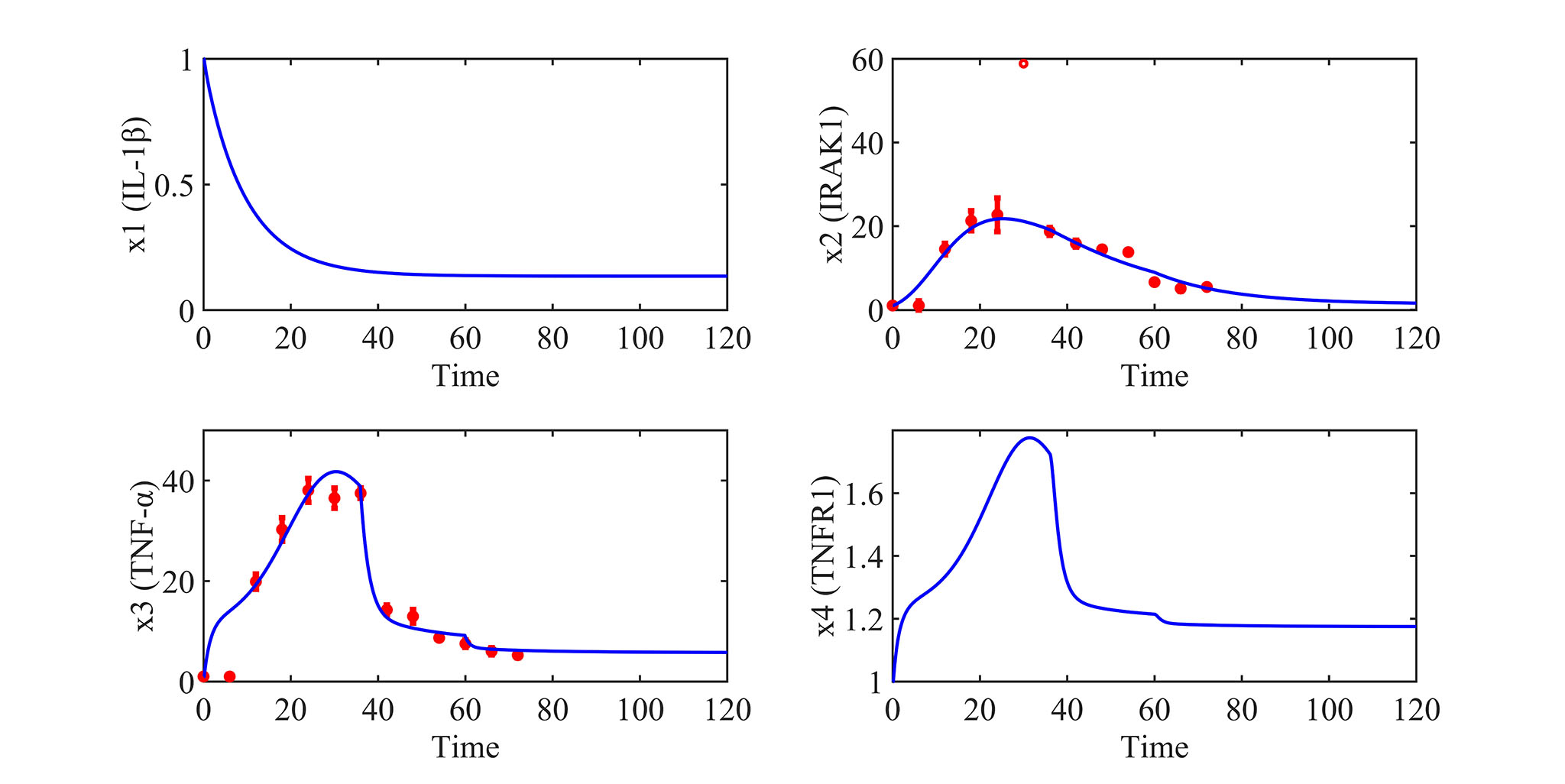

Supplement: Supplementary file 2 [file DataSheet1.zip › Supplementary material_image1/Parameter_a9(小)/1.jpg]

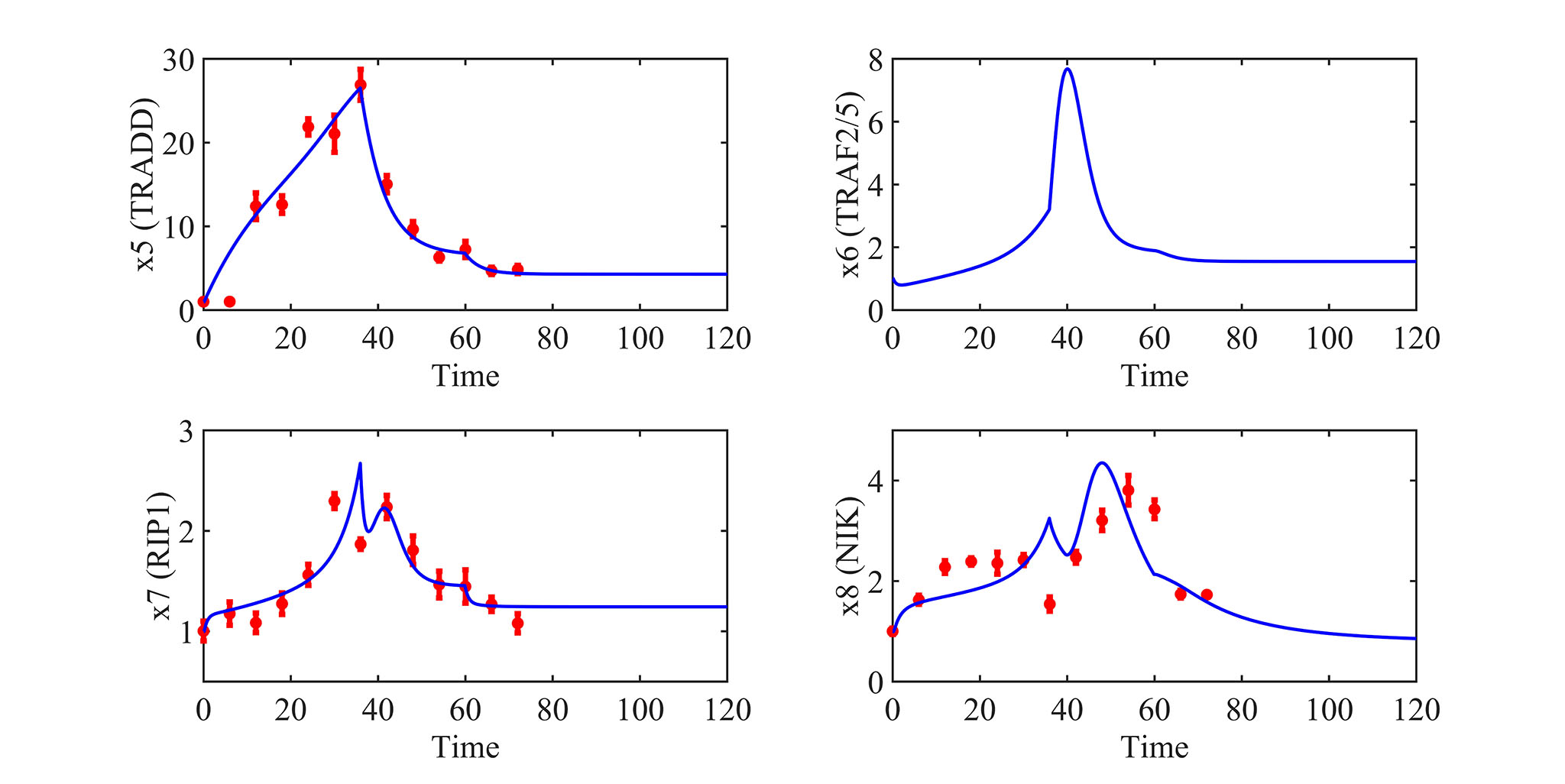

Supplement: Supplementary file 2 [file DataSheet1.zip › Supplementary material_image1/Parameter_a9(小)/2.jpg]

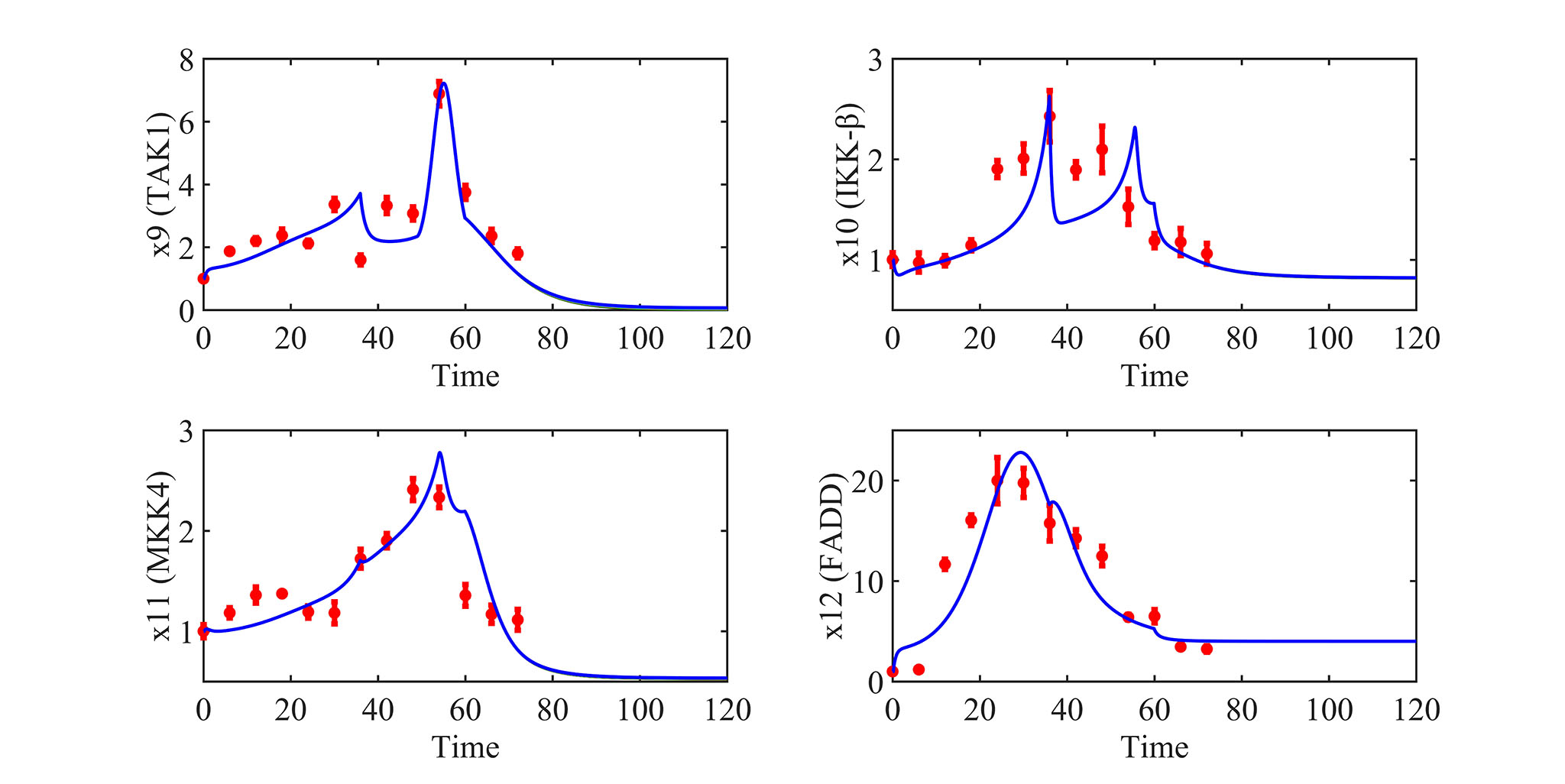

Supplement: Supplementary file 2 [file DataSheet1.zip › Supplementary material_image1/Parameter_a9(小)/3.jpg]

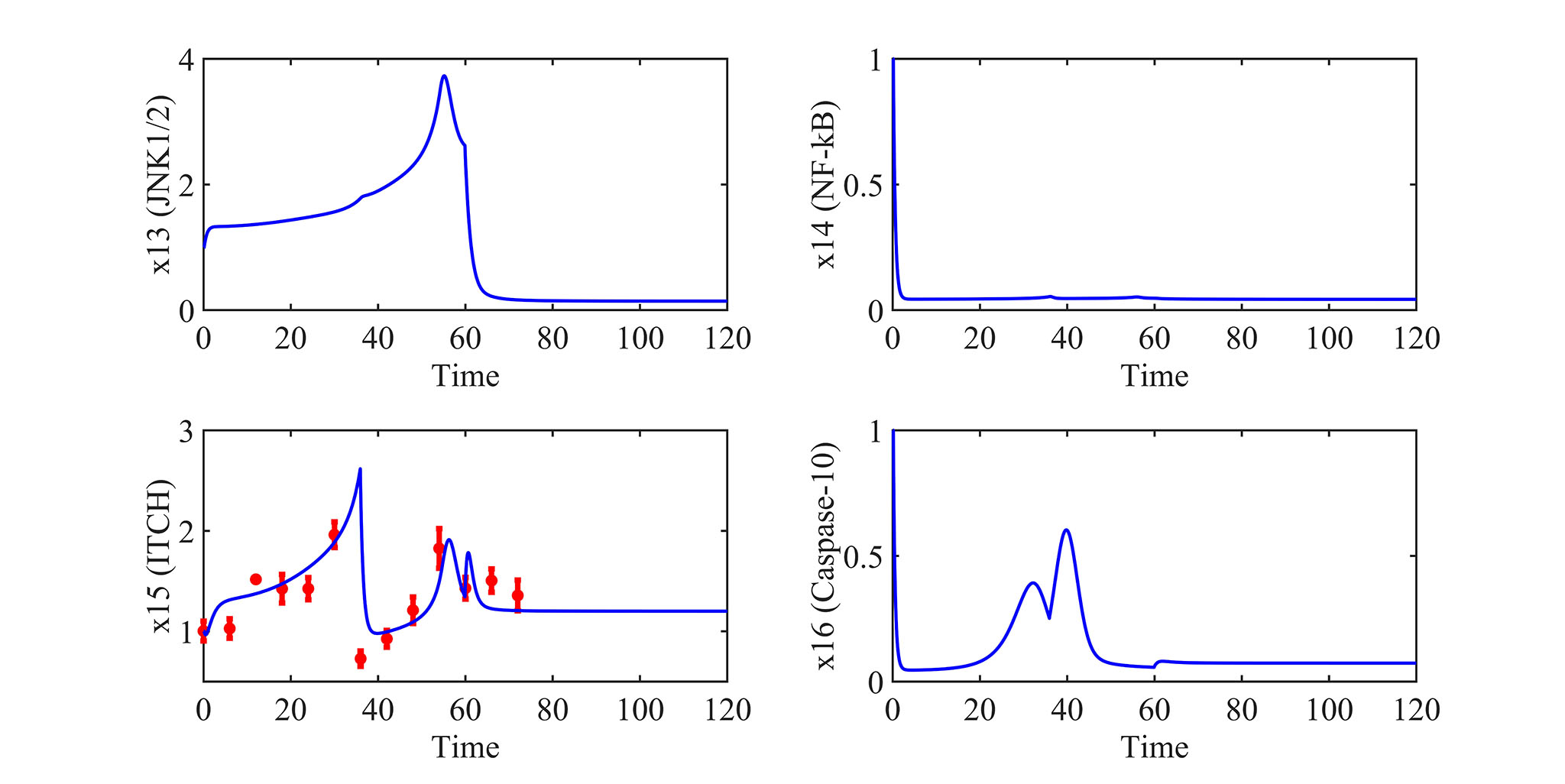

Supplement: Supplementary file 2 [file DataSheet1.zip › Supplementary material_image1/Parameter_a9(小)/4.jpg]

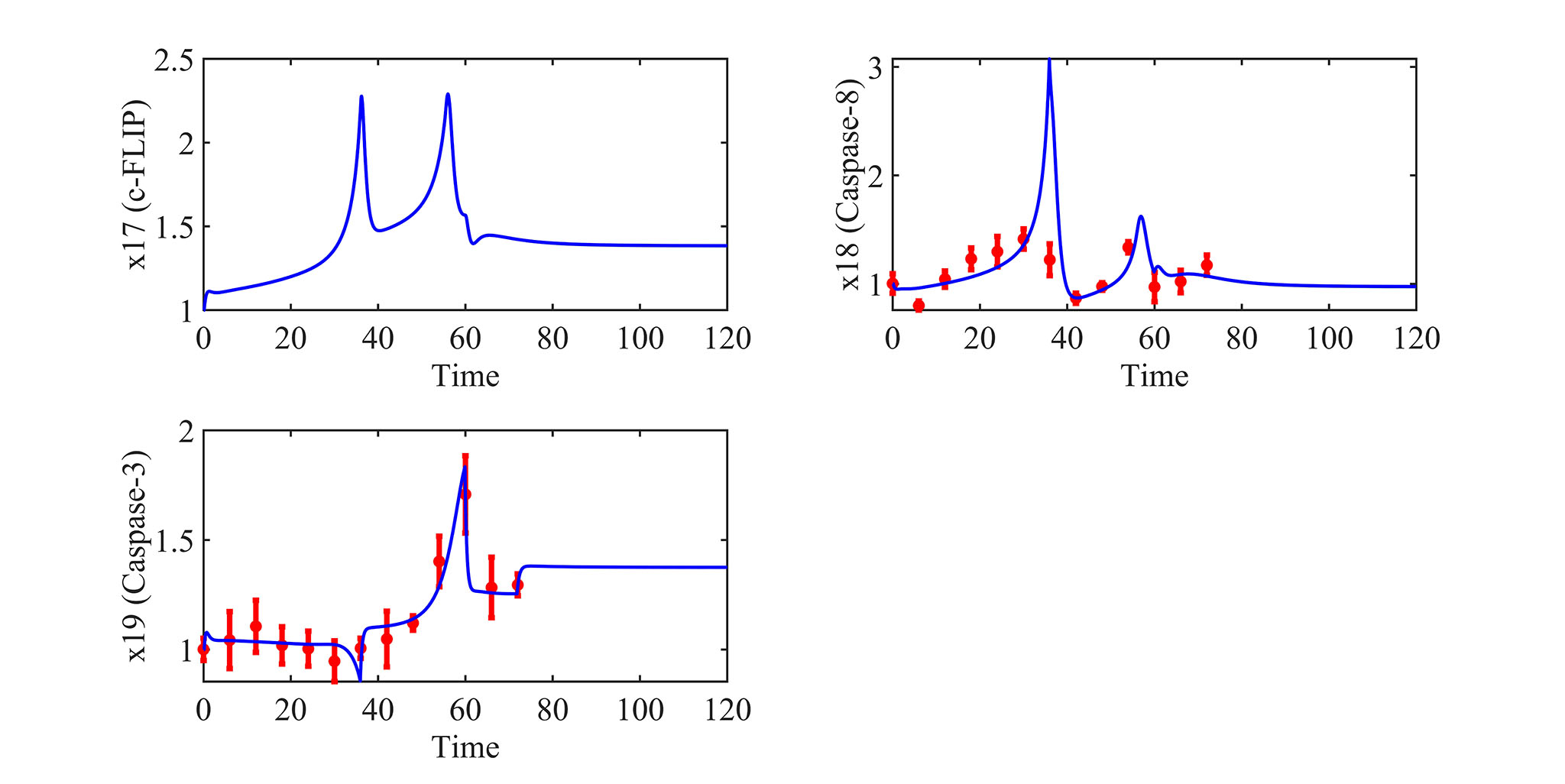

Supplement: Supplementary file 2 [file DataSheet1.zip › Supplementary material_image1/Parameter_a9(小)/5.jpg]

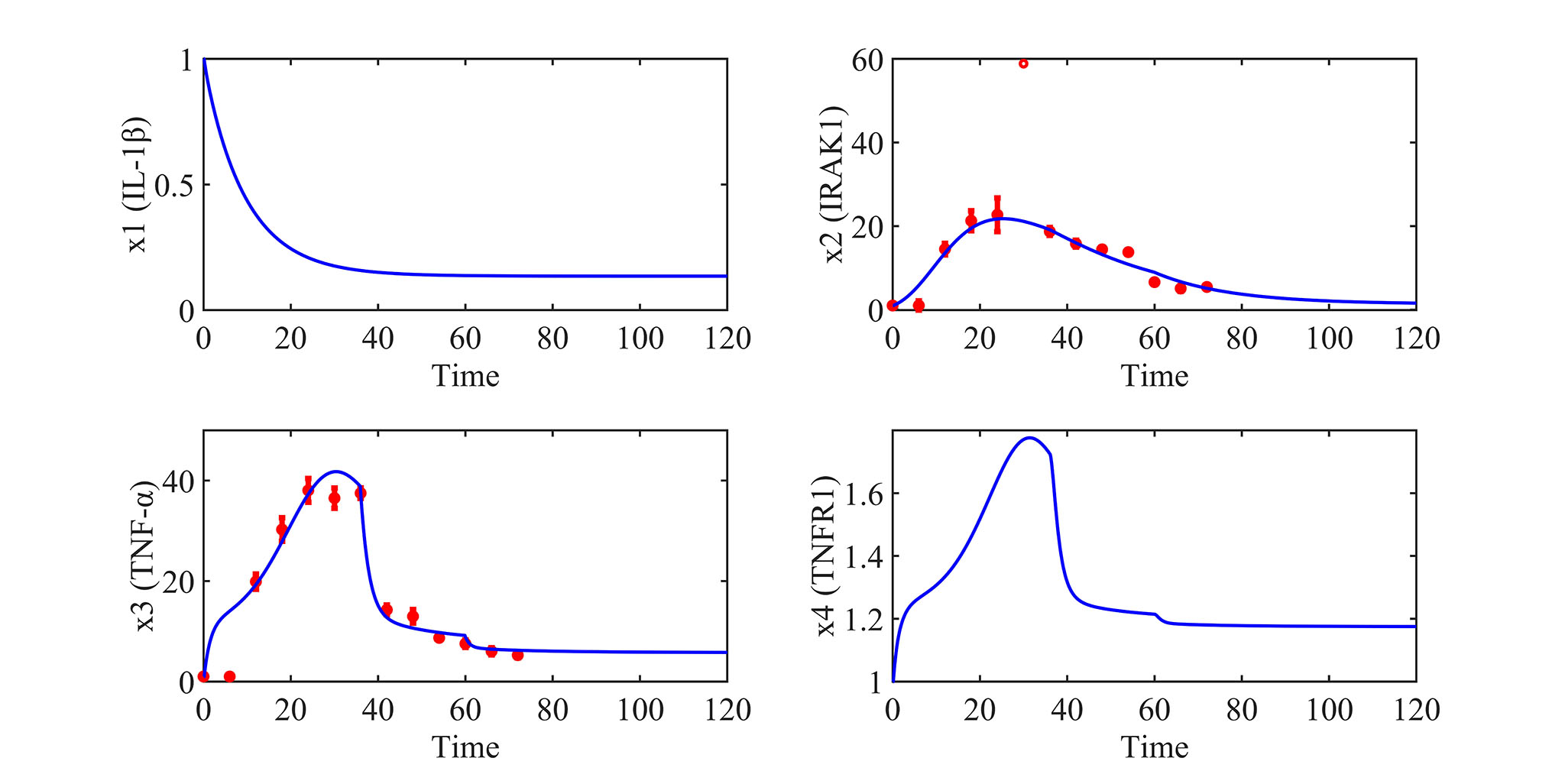

Supplement: Supplementary file 2 [file DataSheet1.zip › Supplementary material_image1/Parameter_b10(大)/1.jpg]

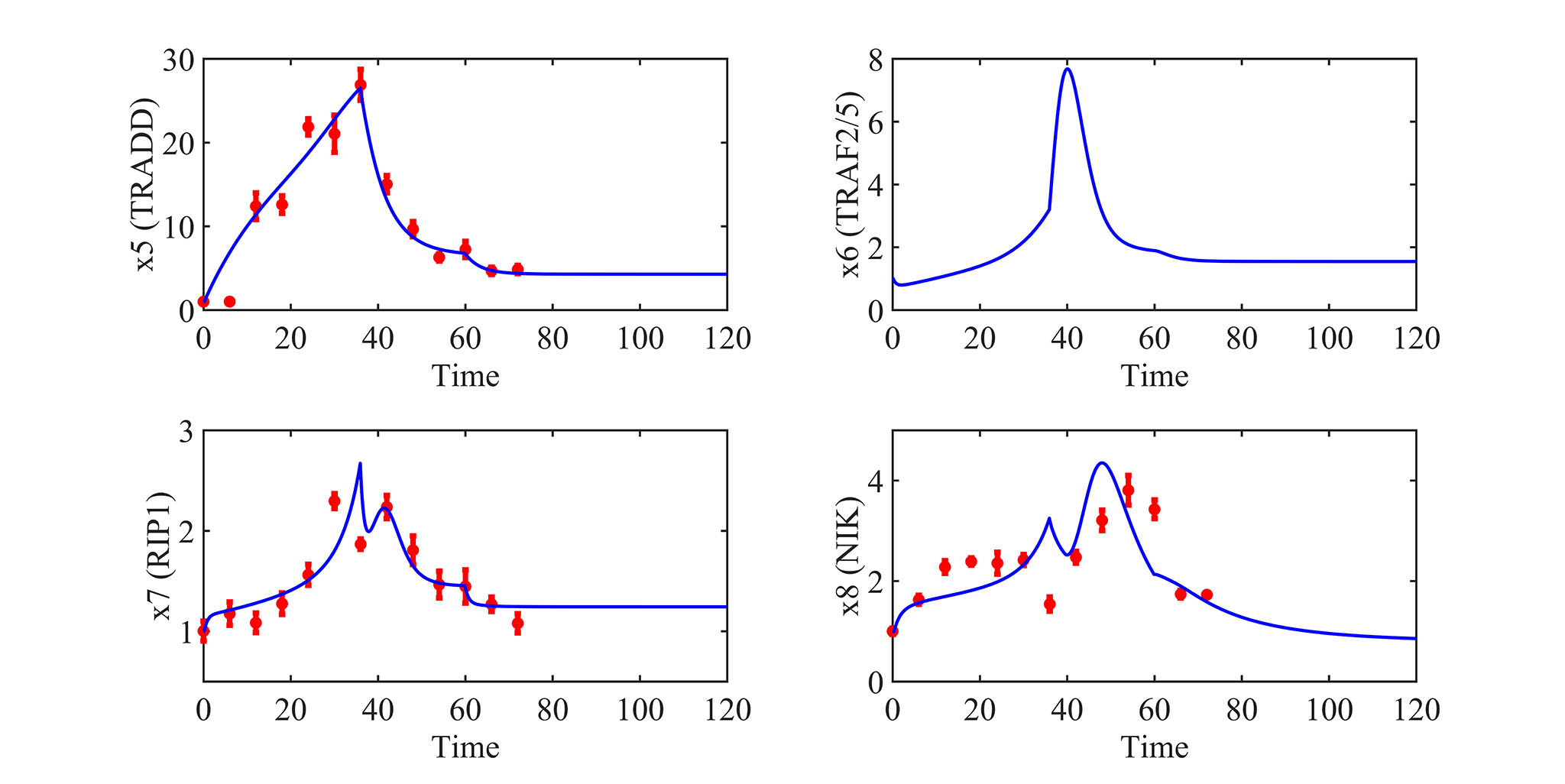

Supplement: Supplementary file 2 [file DataSheet1.zip › Supplementary material_image1/Parameter_b10(大)/2.jpg]

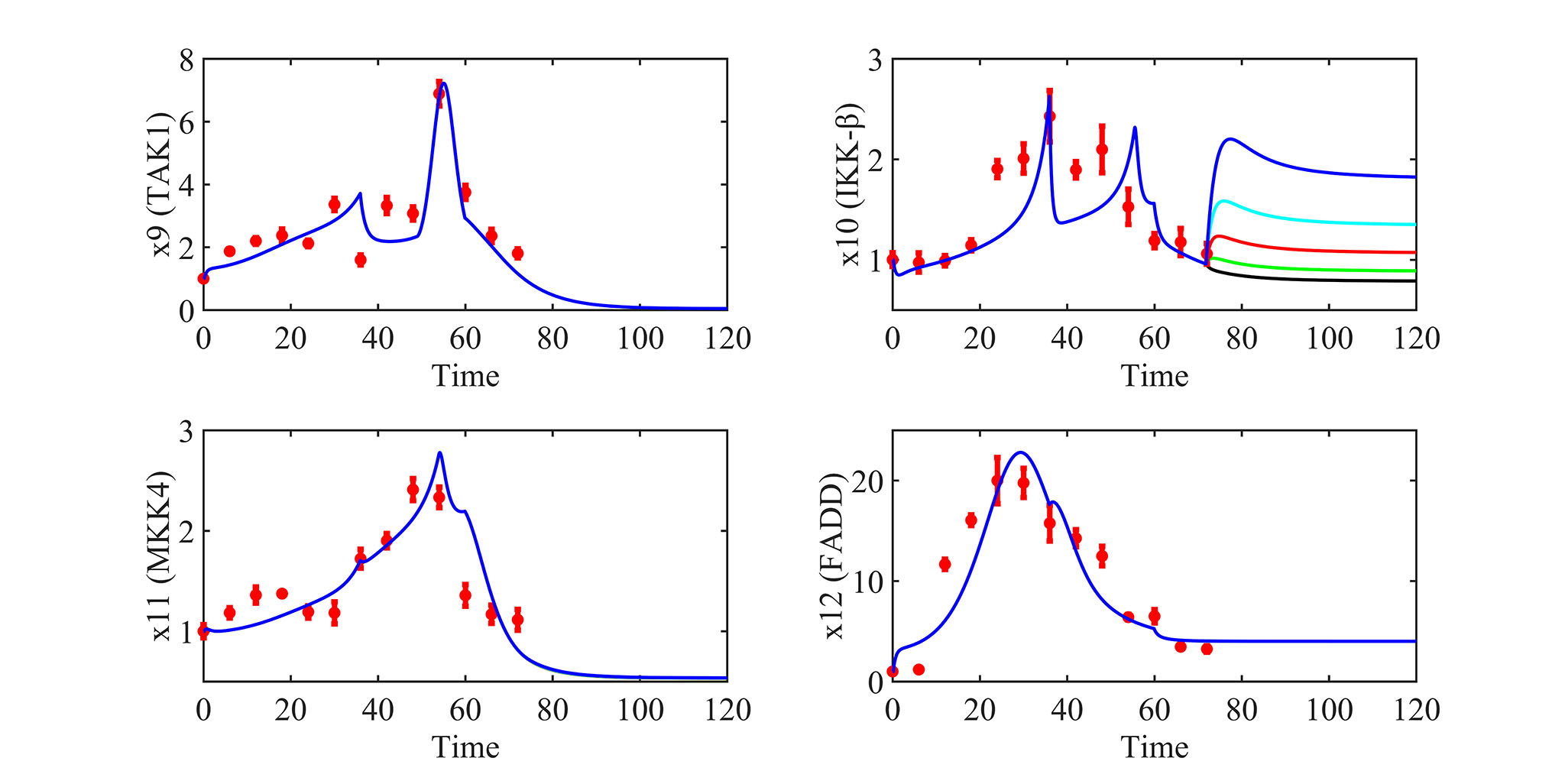

Supplement: Supplementary file 2 [file DataSheet1.zip › Supplementary material_image1/Parameter_b10(大)/3.jpg]

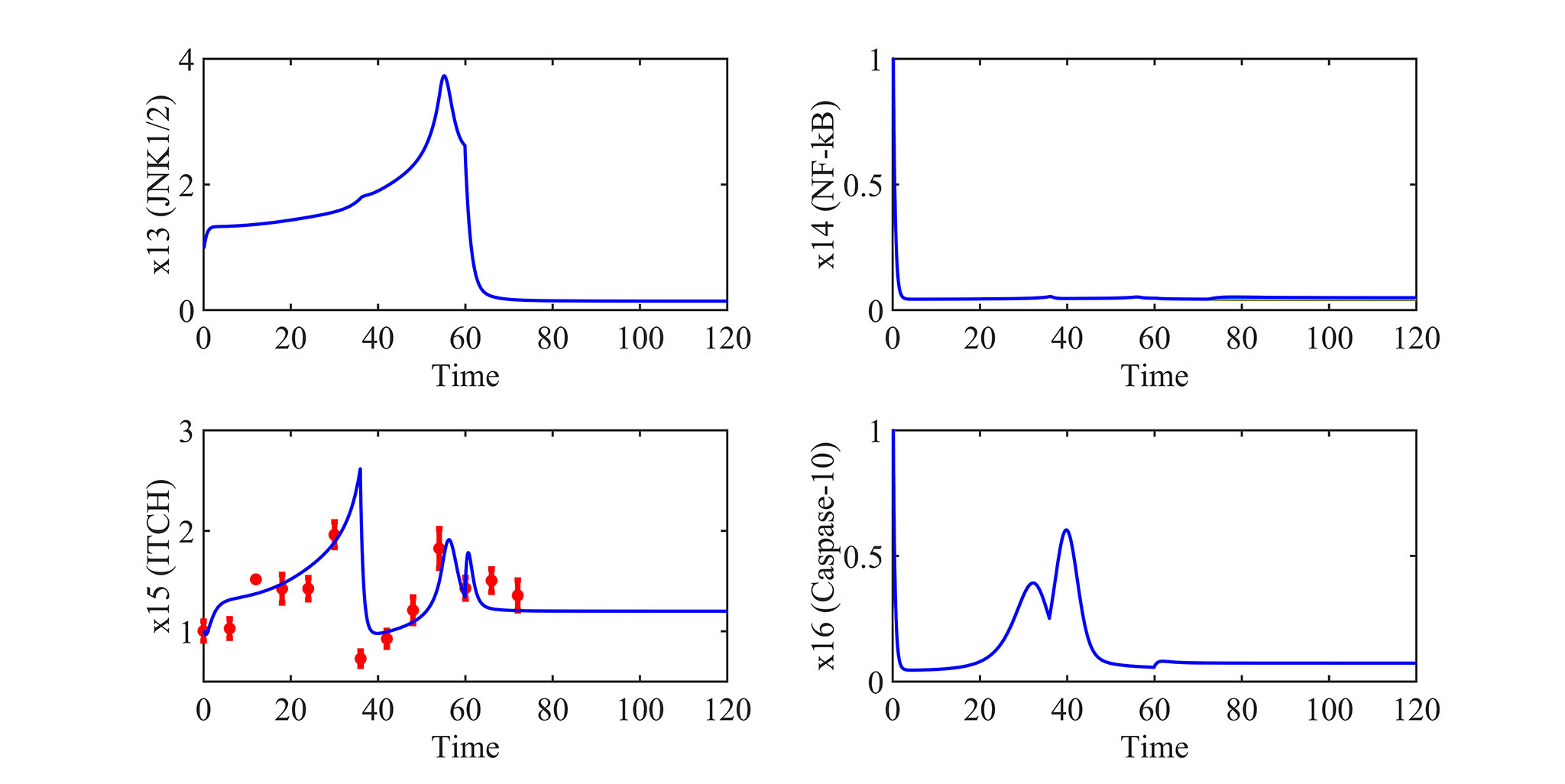

Supplement: Supplementary file 2 [file DataSheet1.zip › Supplementary material_image1/Parameter_b10(大)/4.jpg]

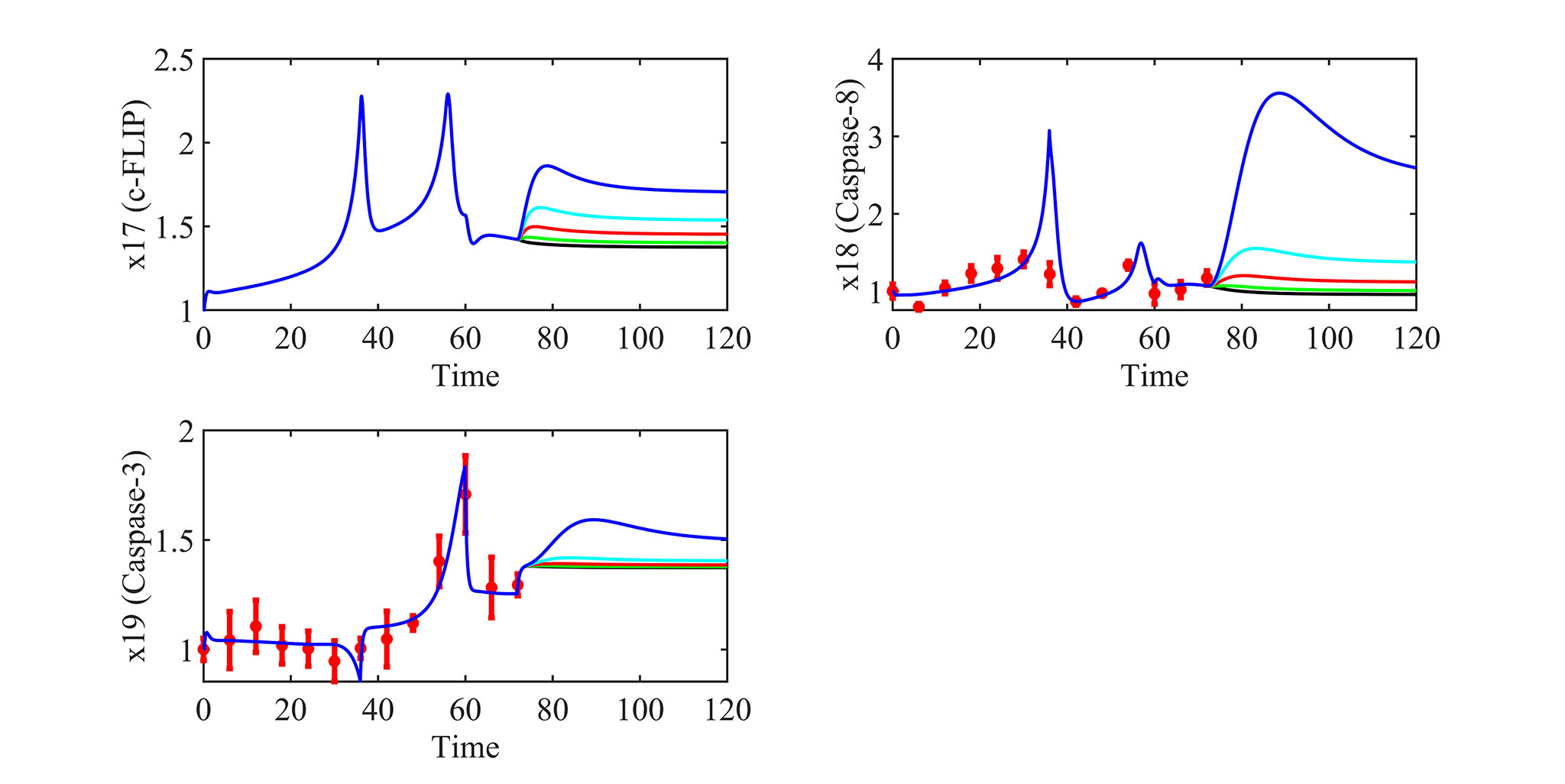

Supplement: Supplementary file 2 [file DataSheet1.zip › Supplementary material_image1/Parameter_b10(大)/5.jpg]
