# Supplementary material for: Mathematical modeling and application of IL-1β/TNF signaling pathway in regulating chondrocyte apoptosis
Source: Front Cell Dev Biol. 2023 Nov 2;11:1288431. doi: 10.3389/fcell.2023.1288431 (PMC10652750; doi:10.3389/fcell.2023.1288431)
Supplement: Supplementary file 3 [file DataSheet1.pdf]

Table S1 Genomic DNN removal reaction system

Reagent	Volume (μl)
RNA template	1μg
gDNA Remover	1
10× gDNA Remover Buffer	1
RNAase-free Water	Up to 10

1) Conventional retrotranscriptional reaction components

Table S2 The conventional retrotranscriptional response system

Reagent	Volume (μl)
Last step reaction solution	10
5×RT Reaction Mix	4
SynScript™ III RT Enzyme Mix	1
RNAase-free Water	Up to 20

2) miRNA retrotranscriptional reaction components

The primer's sequences are as follows:

Table S3 The primer's sequence

**Name**	**Sequence (5'-3')**	**Size**
Homo GAPDH	TCAAGAAGGTGGTGAAGCAGG	115bp
	TCAAAGGTGGAGGAGTGGGT	
Homo TNF-α	CTCGAACCCCGAGTGACAAG	159bp
	TGAGGTACAGGCCCTCTGAT	
TRADD	GGATGAAGAACTGGCTGAGC	246bp
	CATTTGAGACCCACAGAGCG	
IRAK1	GTGGAGAGTGACGAGAGC	236bp
	GGGTTGATGATAATCTGCGGTG	
TAK1	GTCATCCAGTCCCAGTGTCA	188bp
	ATCCTGGTCCAGTTCTGCAA	
NIK	ACCGAGAAGAAGTCCACTGG	185bp
	AGGGACAATTCTGGGTGAGG	
RIP1	CCGAGATGAGTACTCCGCTT	244bp
	CCATTCTTCTTAGCGGTGCC	
FADD	CTGGGGAAGAAGACCTGTGT	194bp
	TGCGTTCTCCTTCTCTGTGT	
IKK-β	AAAAGTGCGGCAGAAGAGTG	219bp
	ATGTCATCCAGGGCCTTGAA	
Mkk4	GACTGAGAACACACAGCATT	246bp

	CACTACTCCGCATTACTACA	
ITCH	GCACGGGCGAGTTTACTATG	225bp
	TGCTGCATTGCTCCTTGAAG	
Caspase8	GGAGGAGTTGTGTGGGGTAA	207bp
	CCTGCATCCAAGTGTGTTCC	
Caspase3	ACTGGACTGTGGCATTGAGA	162bp
	GCACAAAGCGACTGGATGAA	
BAX	CCCGAGAGGTCTTTTTCCGAG	155bp
	CCCGAGAGGTCTTTTTCCGAG	
BCL-2	GGTGGGGTCATGTGTGTGG	89bp
	GGTGGGGTCATGTGTGTGG	

Table S4 The miRNA retrotranscriptional response system

Reagent	Volume（μl)
Last step reaction solution	10
dNTPs(10 μM)	2
miRNA Loop Primer(10μM)	2
SynScript™ III RT Enzyme Mix	1
RNAase-free Water	Up to 20

Table S5 Real-time PCR reaction program

**Period**	**Temperature**	**Time**	**Cycle number**
pre-deformation	95°C	1min	1 cycle
circular reaction	95°C	10s	40 cycle Fluorescence signal was collected at 72°C
	60°C	10s	
	72°C	10-15s	
Solution curve analysis and collection			
