# Supplementary figures and images for: Mathematical modeling and application of IL-1β/TNF signaling pathway in regulating chondrocyte apoptosis (part 4 of 4)
Source: Front Cell Dev Biol. 2023 Nov 2;11:1288431. doi: 10.3389/fcell.2023.1288431 (PMC10652750; doi:10.3389/fcell.2023.1288431)

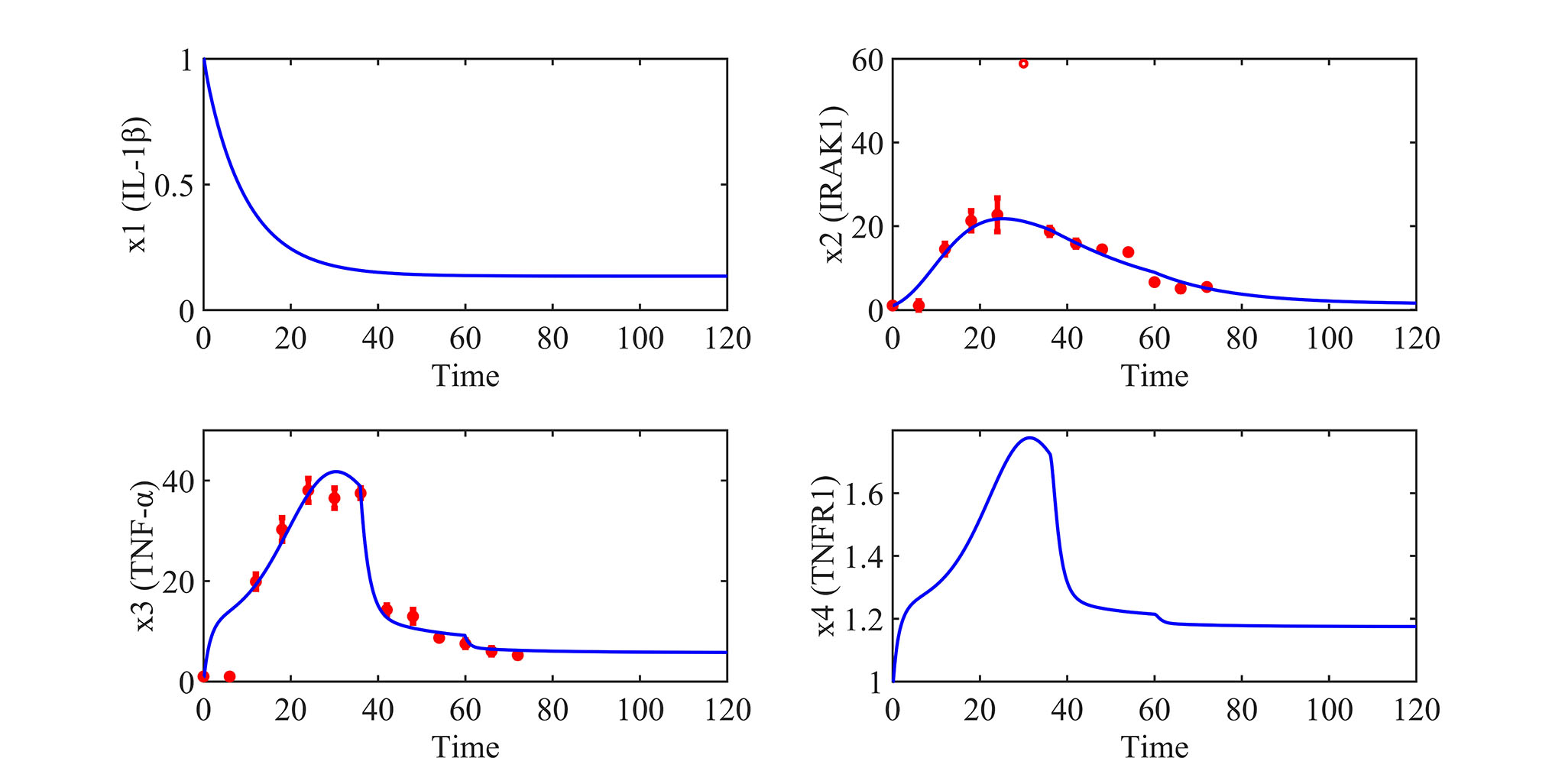

Supplement: Supplementary file 4 [file DataSheet2.zip › Supplementary material_image2/Parameter_g9(小)/1.jpg]

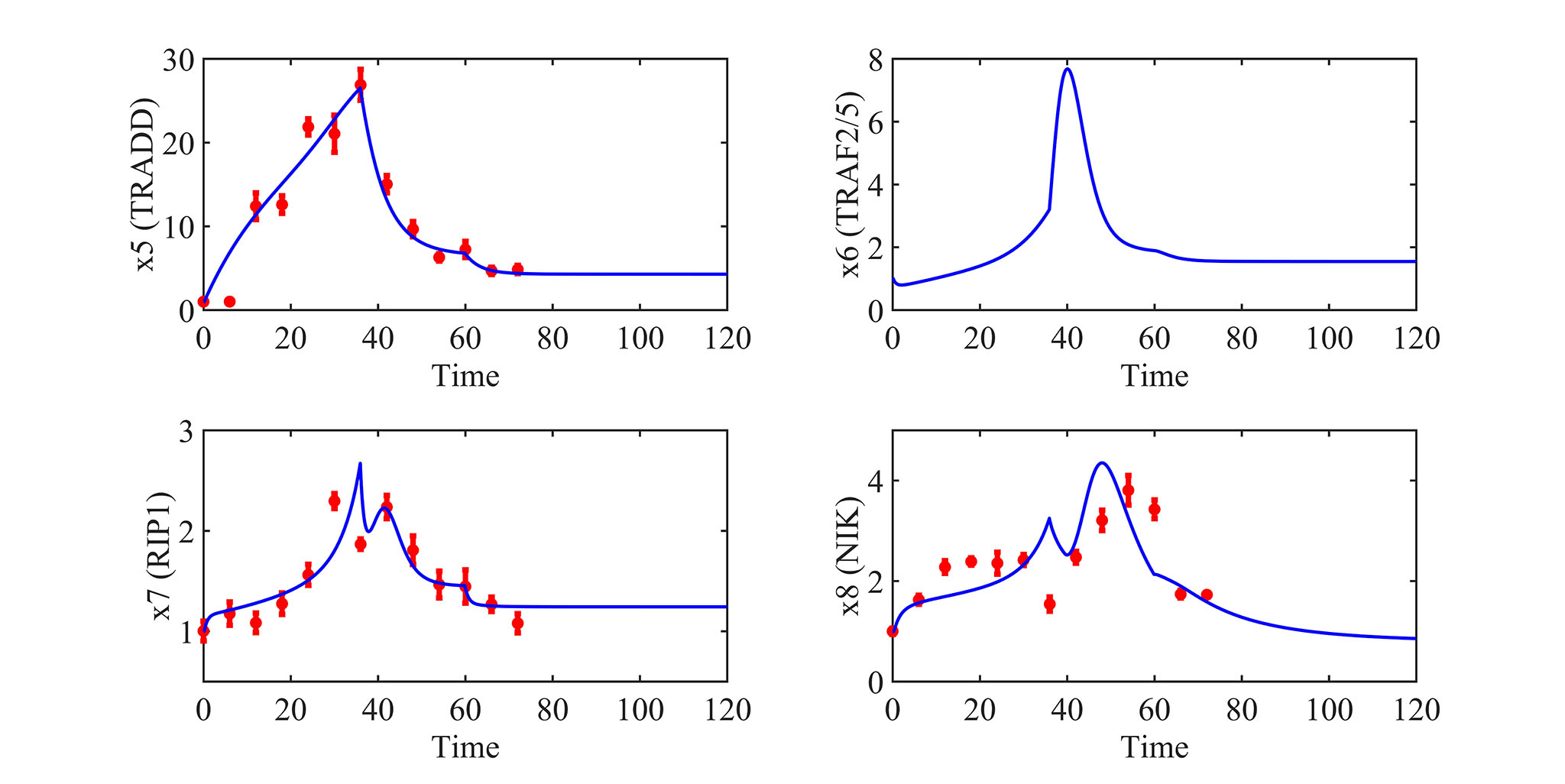

Supplement: Supplementary file 4 [file DataSheet2.zip › Supplementary material_image2/Parameter_g9(小)/2.jpg]

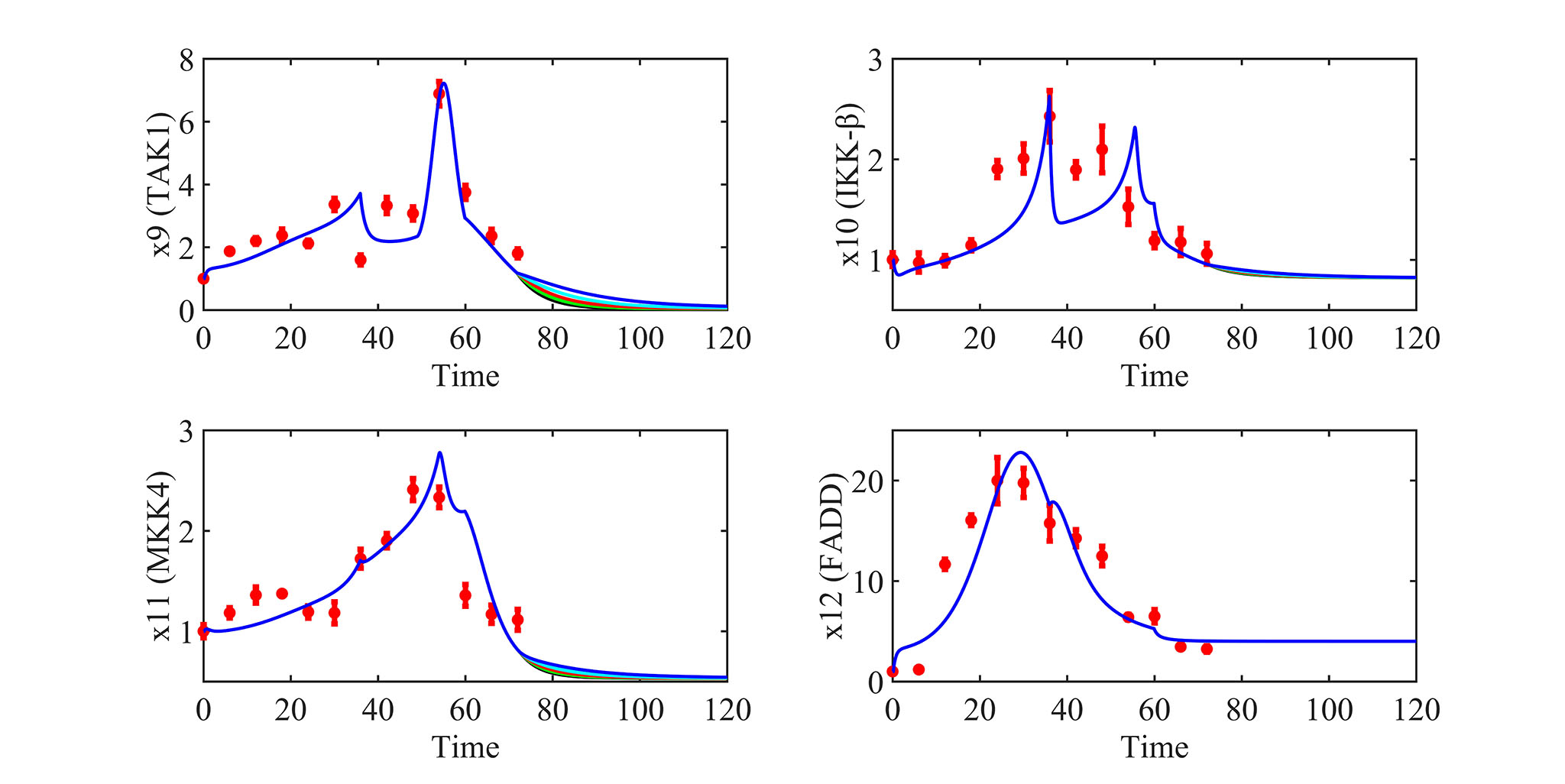

Supplement: Supplementary file 4 [file DataSheet2.zip › Supplementary material_image2/Parameter_g9(小)/3.jpg]

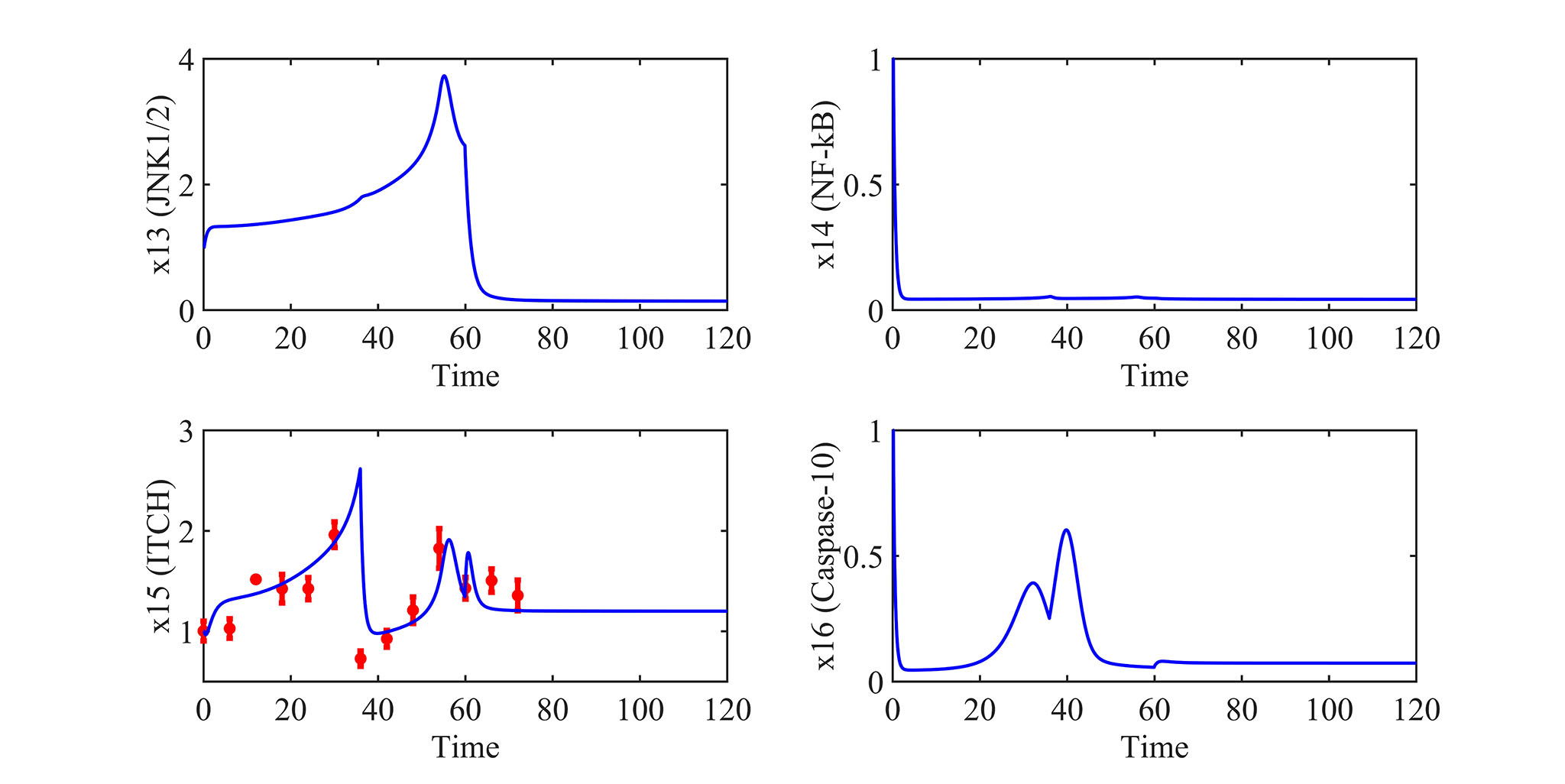

Supplement: Supplementary file 4 [file DataSheet2.zip › Supplementary material_image2/Parameter_g9(小)/4.jpg]

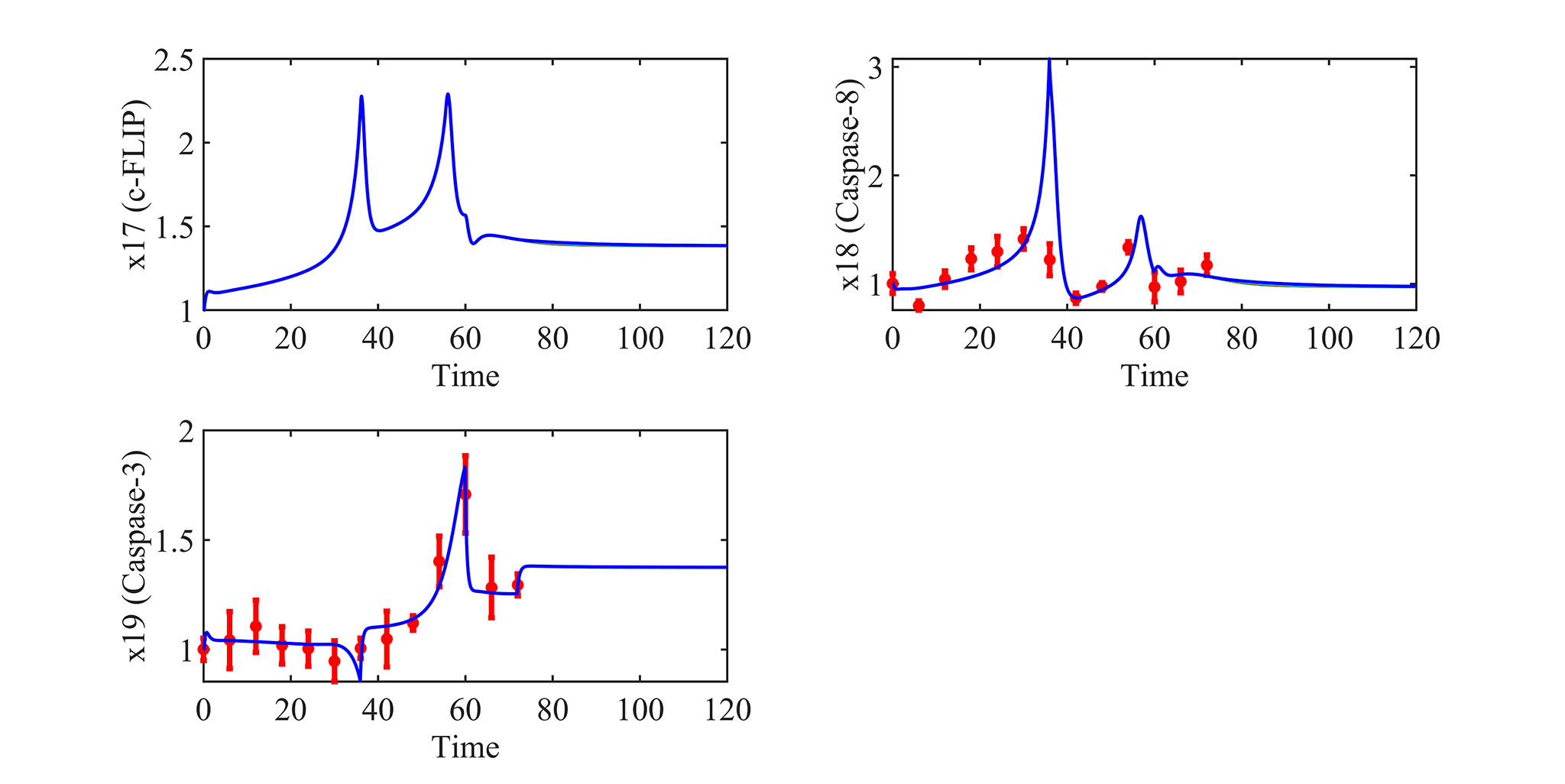

Supplement: Supplementary file 4 [file DataSheet2.zip › Supplementary material_image2/Parameter_g9(小)/5.jpg]
