# Supplementary figures and images for: Mathematical modeling and application of IL-1β/TNF signaling pathway in regulating chondrocyte apoptosis (part 3 of 4)
Source: Front Cell Dev Biol. 2023 Nov 2;11:1288431. doi: 10.3389/fcell.2023.1288431 (PMC10652750; doi:10.3389/fcell.2023.1288431)

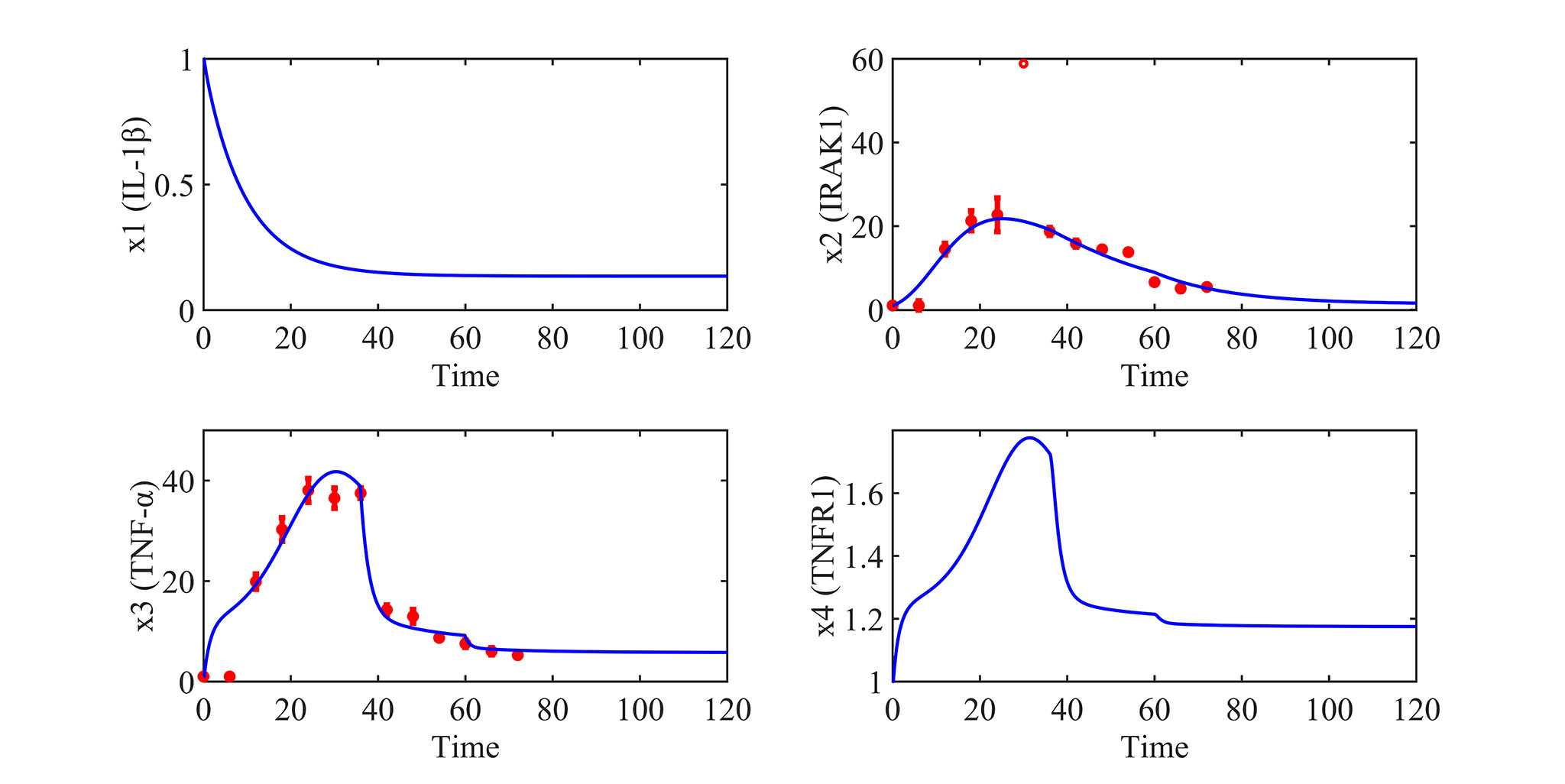

Supplement: Supplementary file 4 [file DataSheet2.zip › Supplementary material_image2/Parameter_d15(小)/1.jpg]

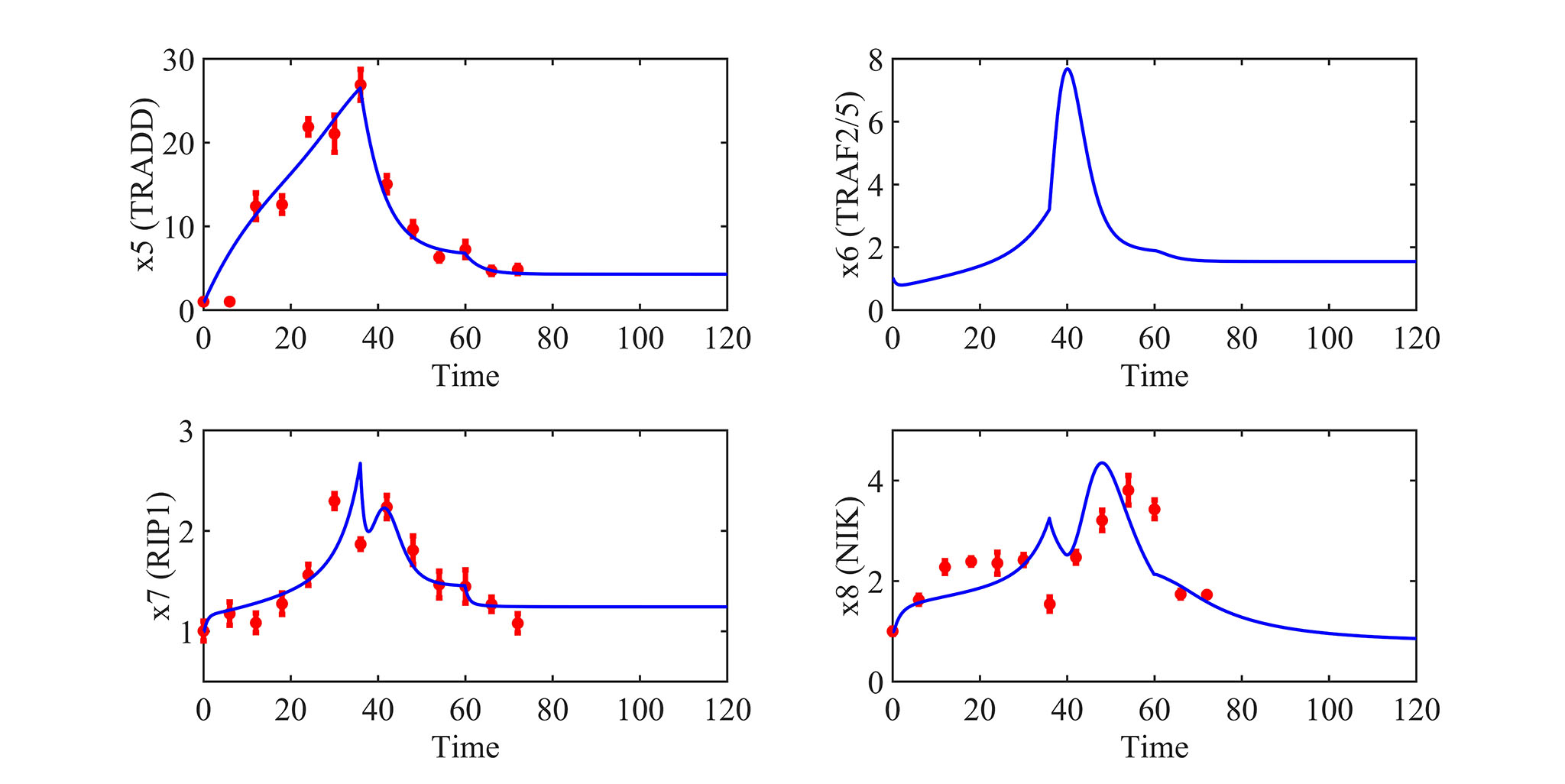

Supplement: Supplementary file 4 [file DataSheet2.zip › Supplementary material_image2/Parameter_d15(小)/2.jpg]

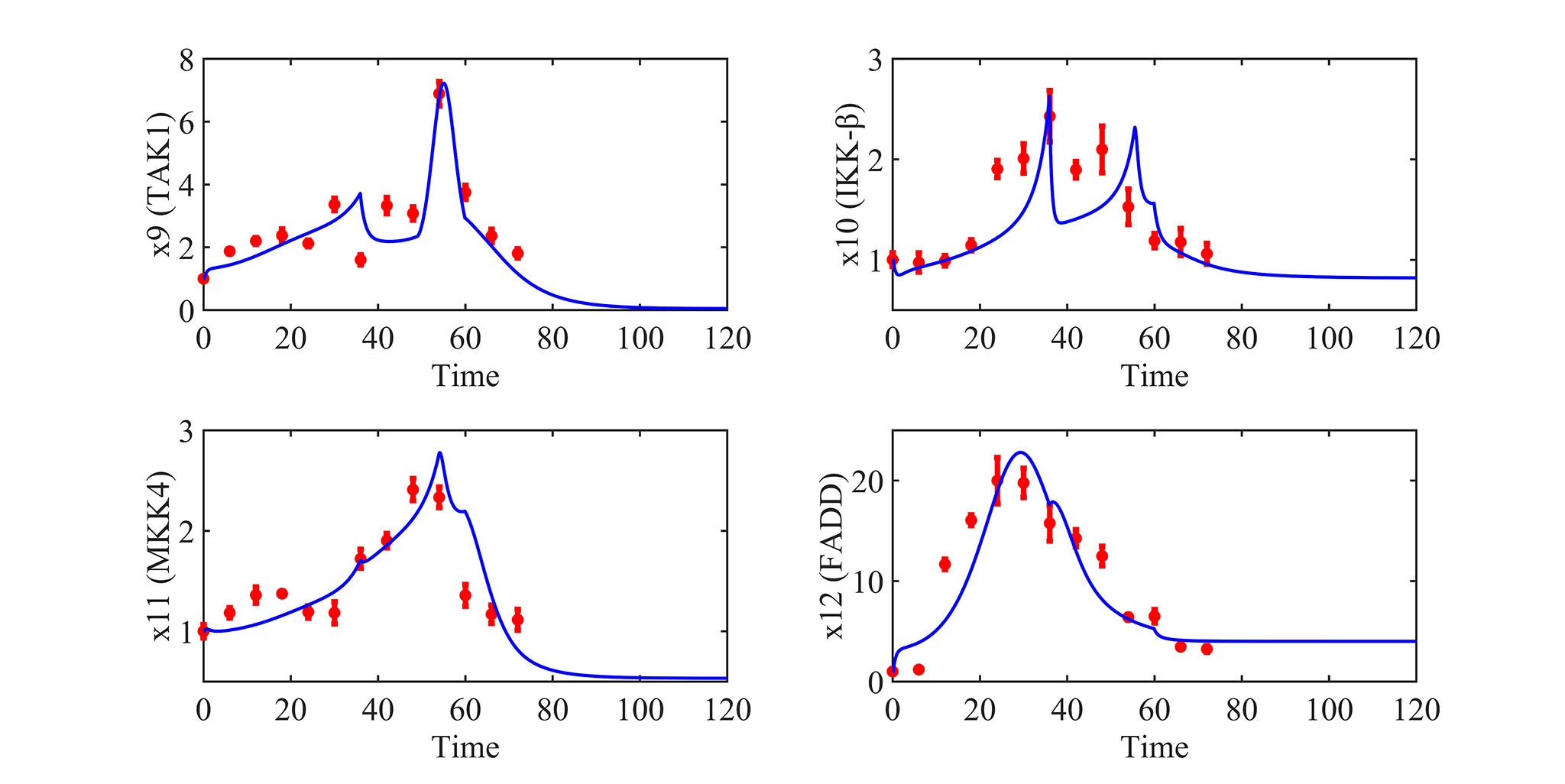

Supplement: Supplementary file 4 [file DataSheet2.zip › Supplementary material_image2/Parameter_d15(小)/3.jpg]

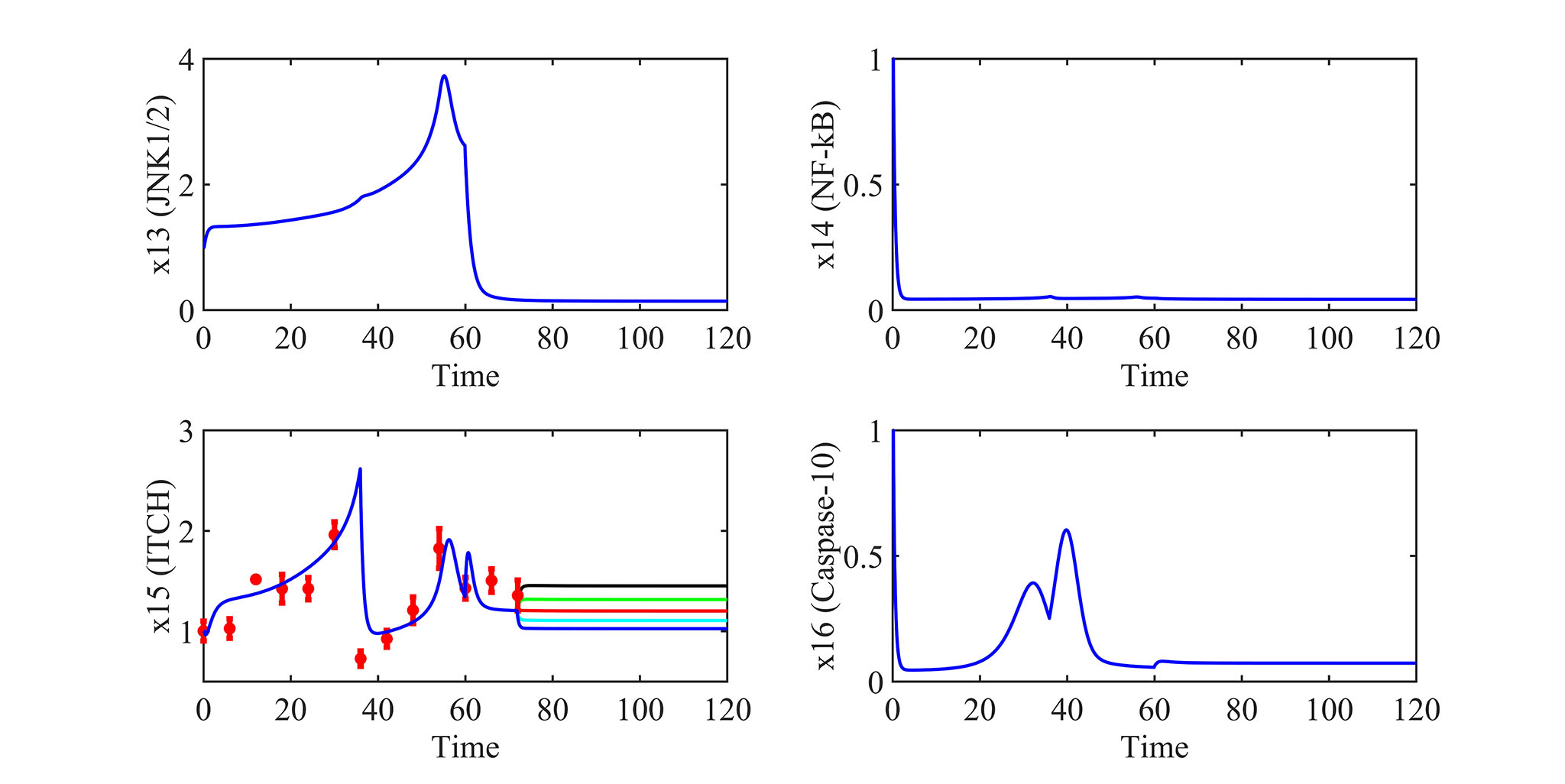

Supplement: Supplementary file 4 [file DataSheet2.zip › Supplementary material_image2/Parameter_d15(小)/4.jpg]

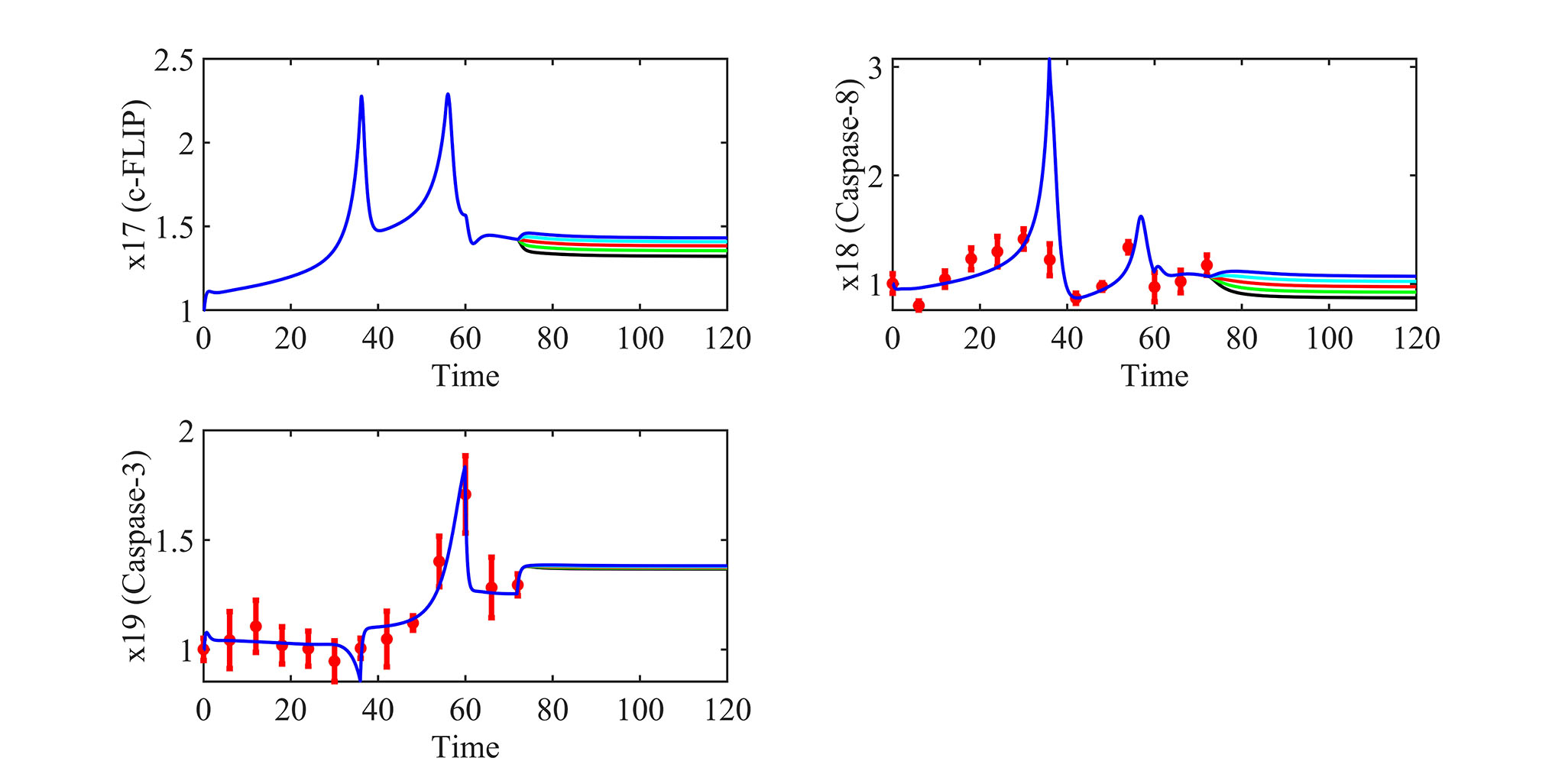

Supplement: Supplementary file 4 [file DataSheet2.zip › Supplementary material_image2/Parameter_d15(小)/5.jpg]

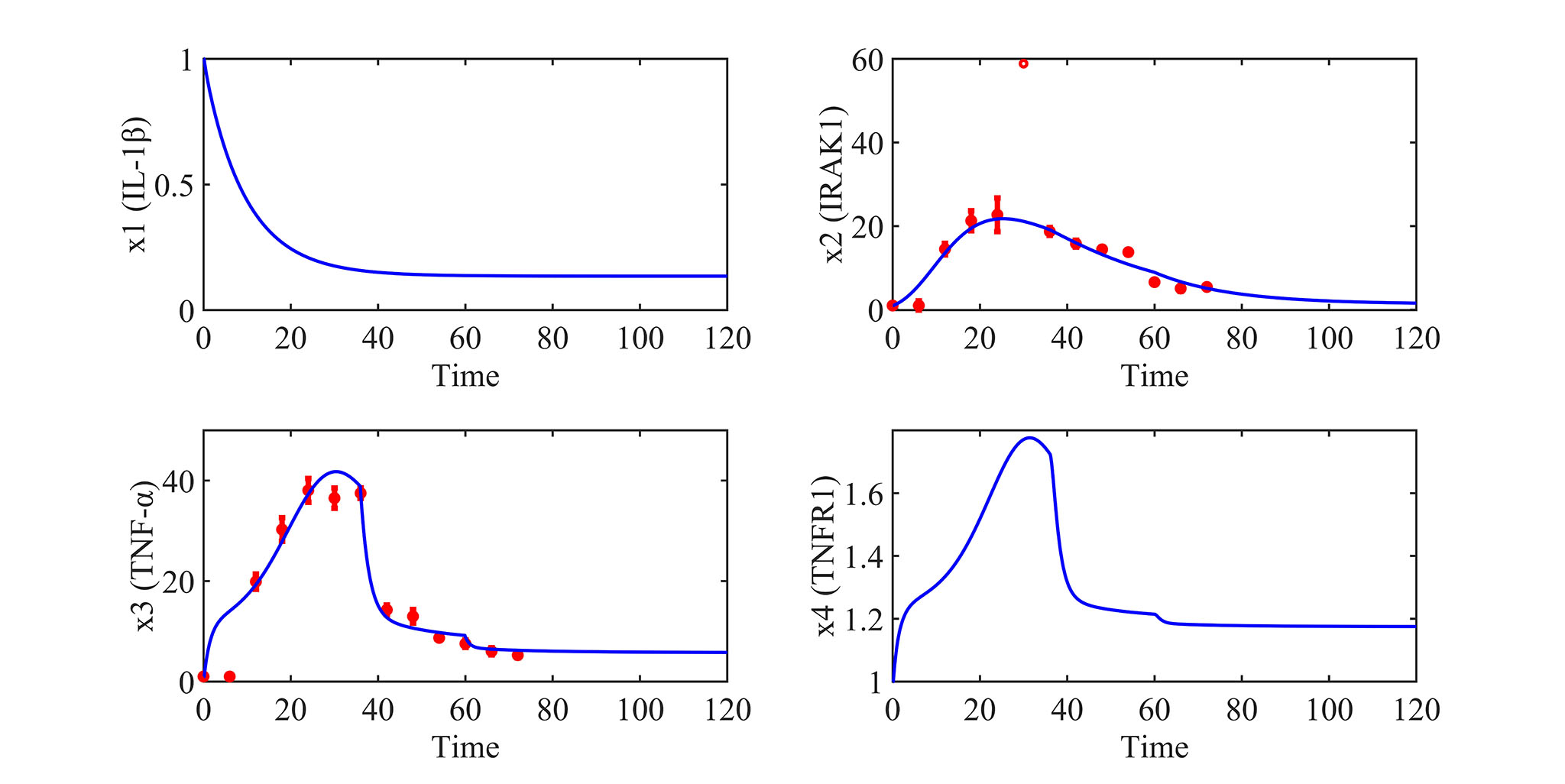

Supplement: Supplementary file 4 [file DataSheet2.zip › Supplementary material_image2/Parameter_d16(大)/1.jpg]

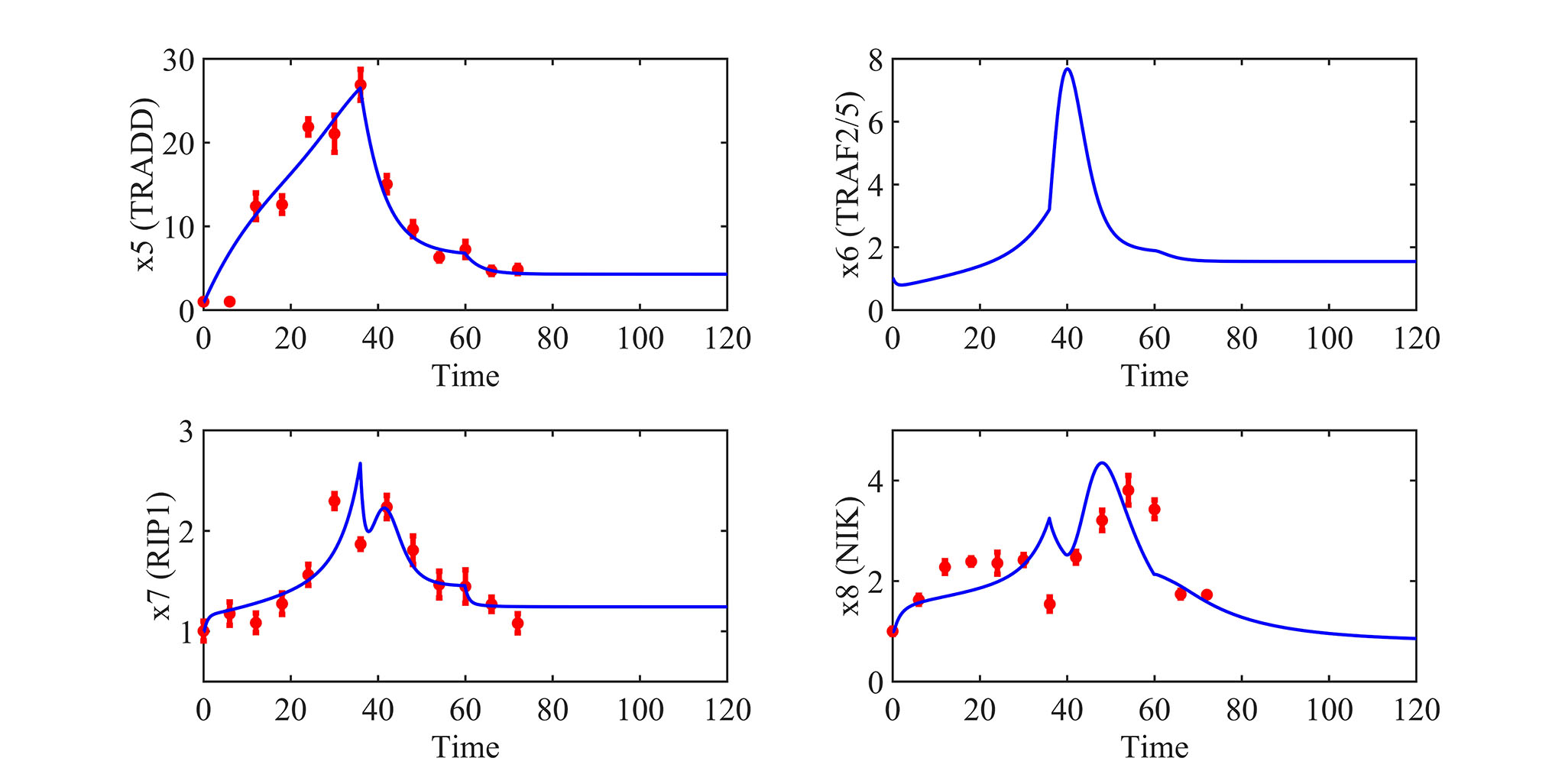

Supplement: Supplementary file 4 [file DataSheet2.zip › Supplementary material_image2/Parameter_d16(大)/2.jpg]

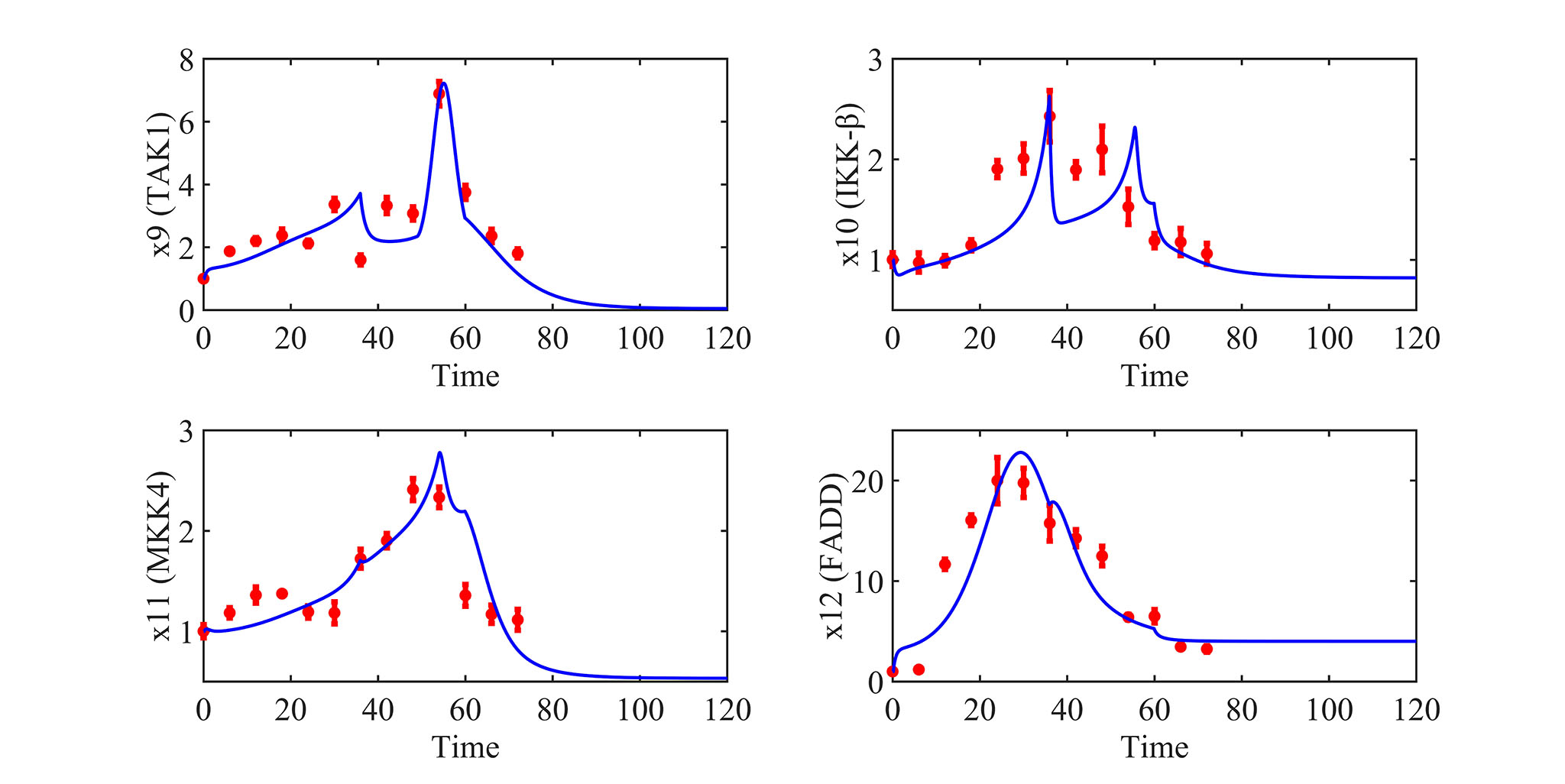

Supplement: Supplementary file 4 [file DataSheet2.zip › Supplementary material_image2/Parameter_d16(大)/3.jpg]

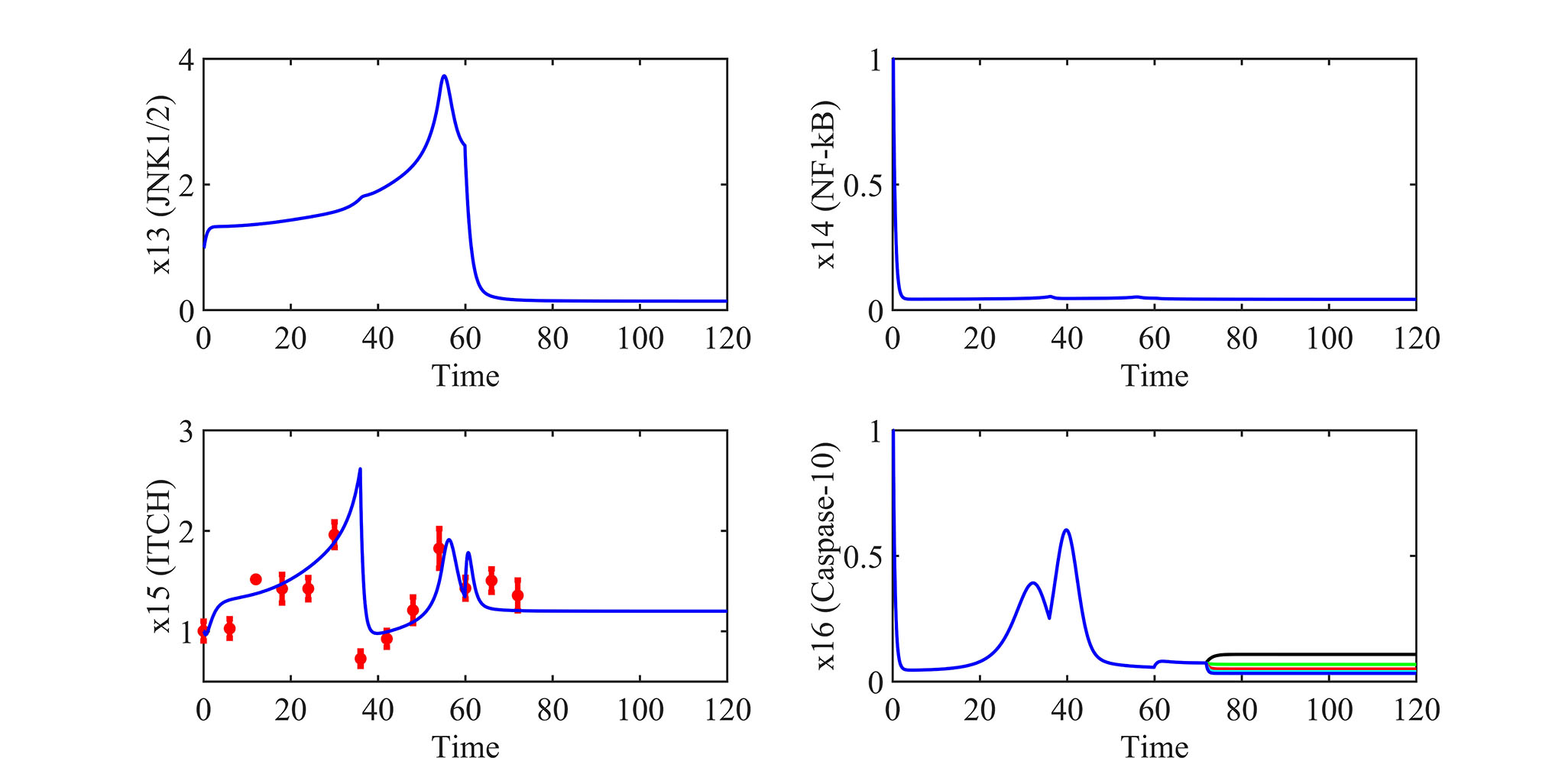

Supplement: Supplementary file 4 [file DataSheet2.zip › Supplementary material_image2/Parameter_d16(大)/4.jpg]

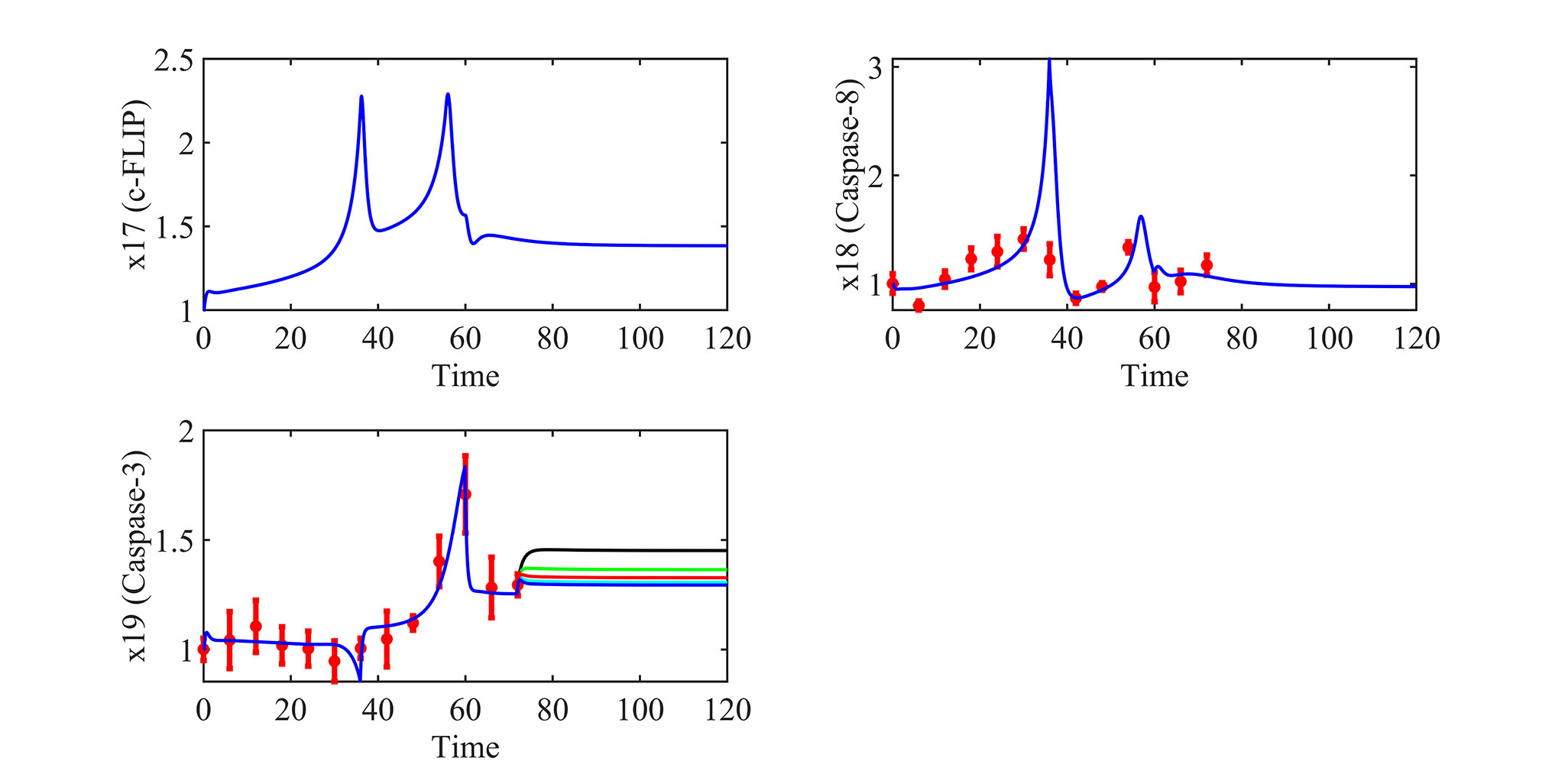

Supplement: Supplementary file 4 [file DataSheet2.zip › Supplementary material_image2/Parameter_d16(大)/5.jpg]

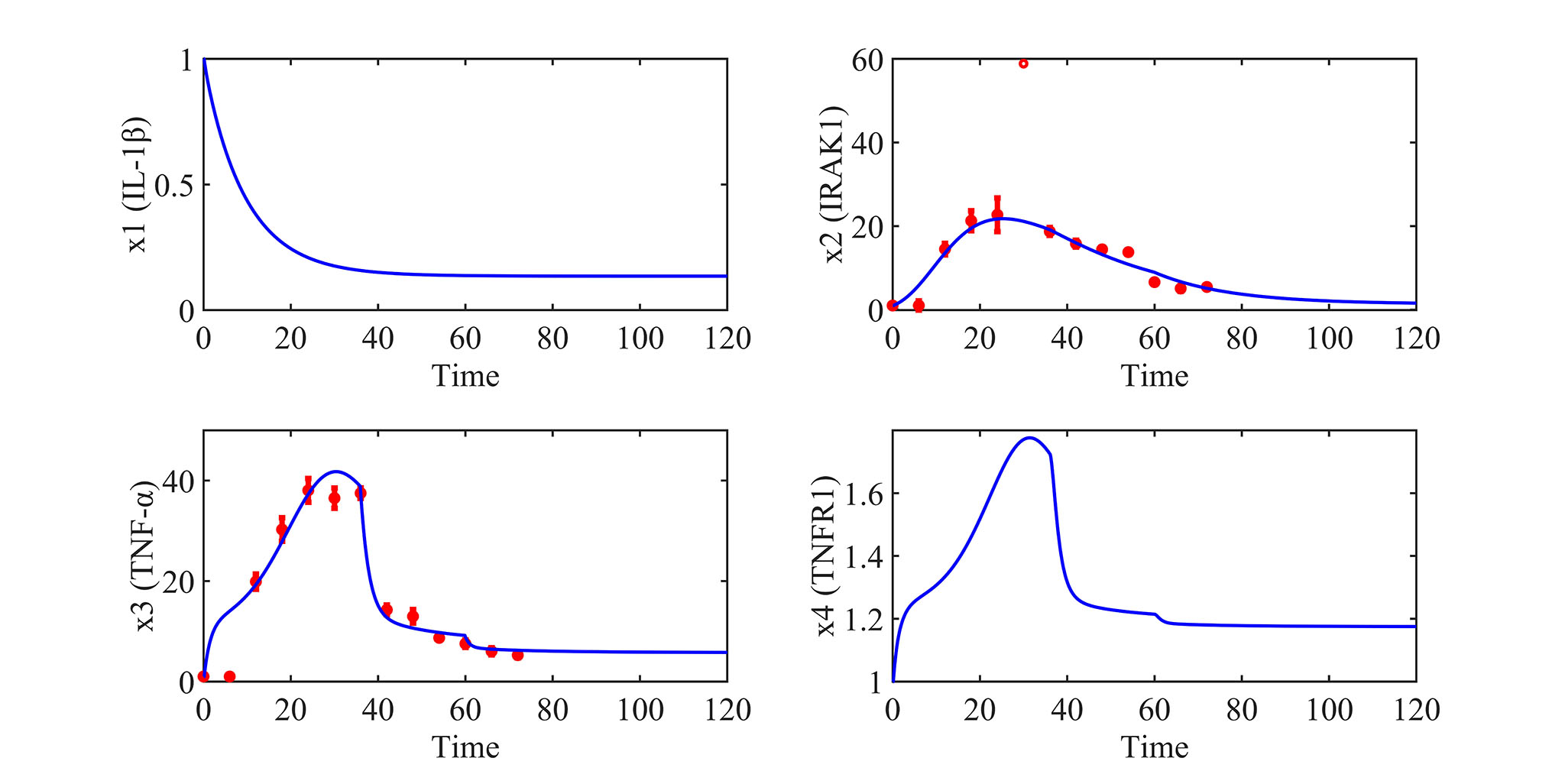

Supplement: Supplementary file 4 [file DataSheet2.zip › Supplementary material_image2/Parameter_d17(中)/1.jpg]

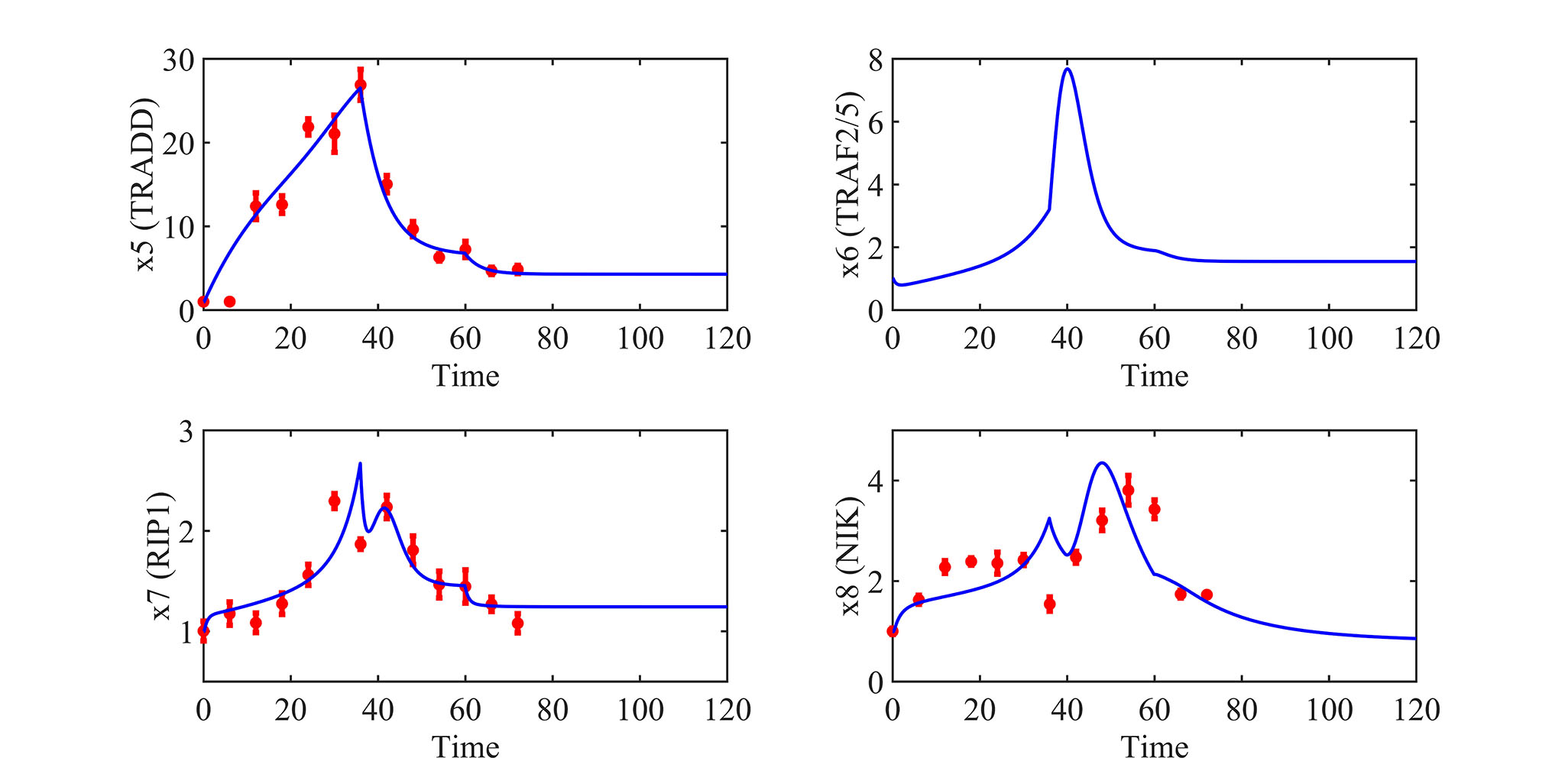

Supplement: Supplementary file 4 [file DataSheet2.zip › Supplementary material_image2/Parameter_d17(中)/2.jpg]

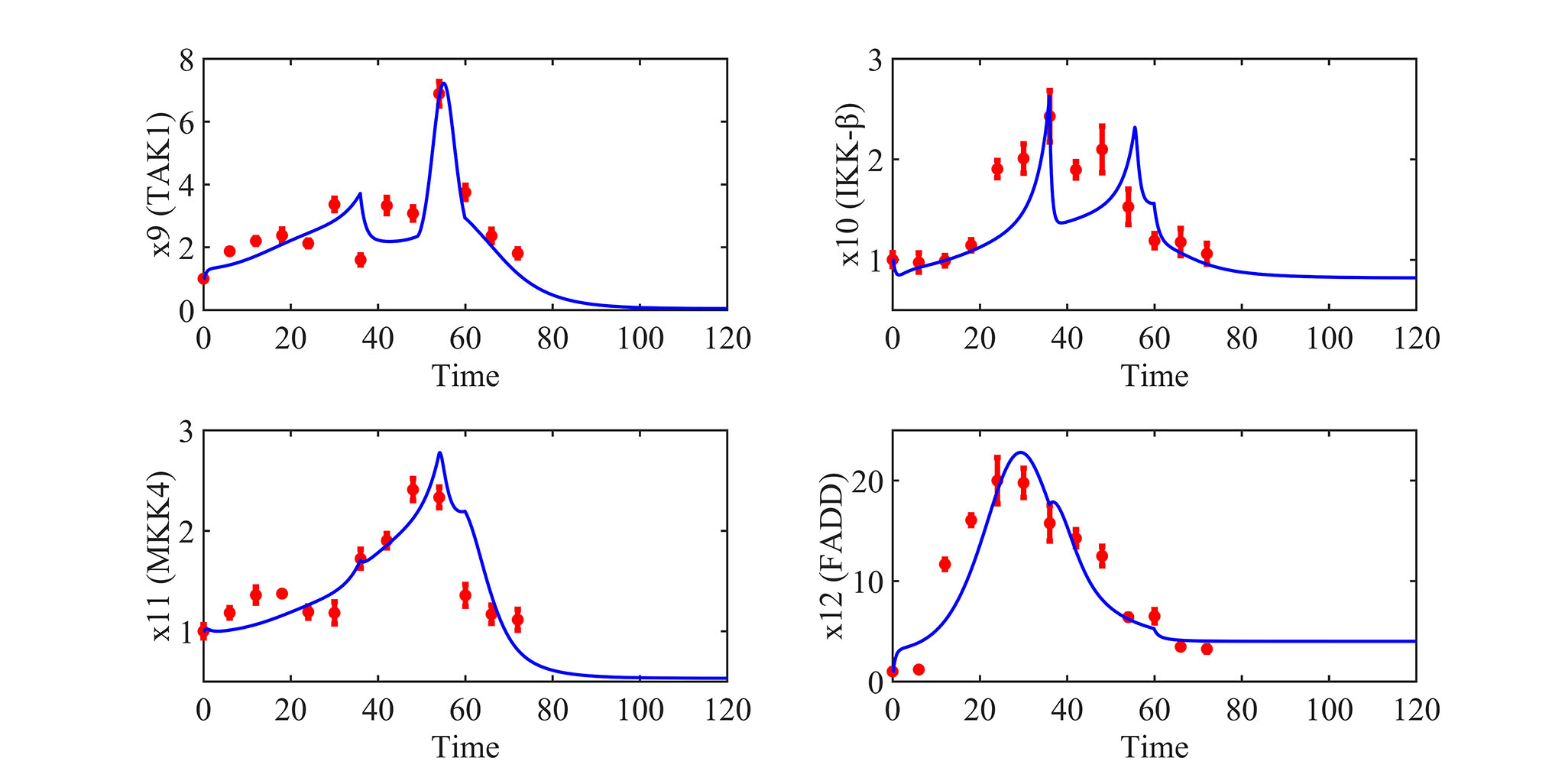

Supplement: Supplementary file 4 [file DataSheet2.zip › Supplementary material_image2/Parameter_d17(中)/3.jpg]

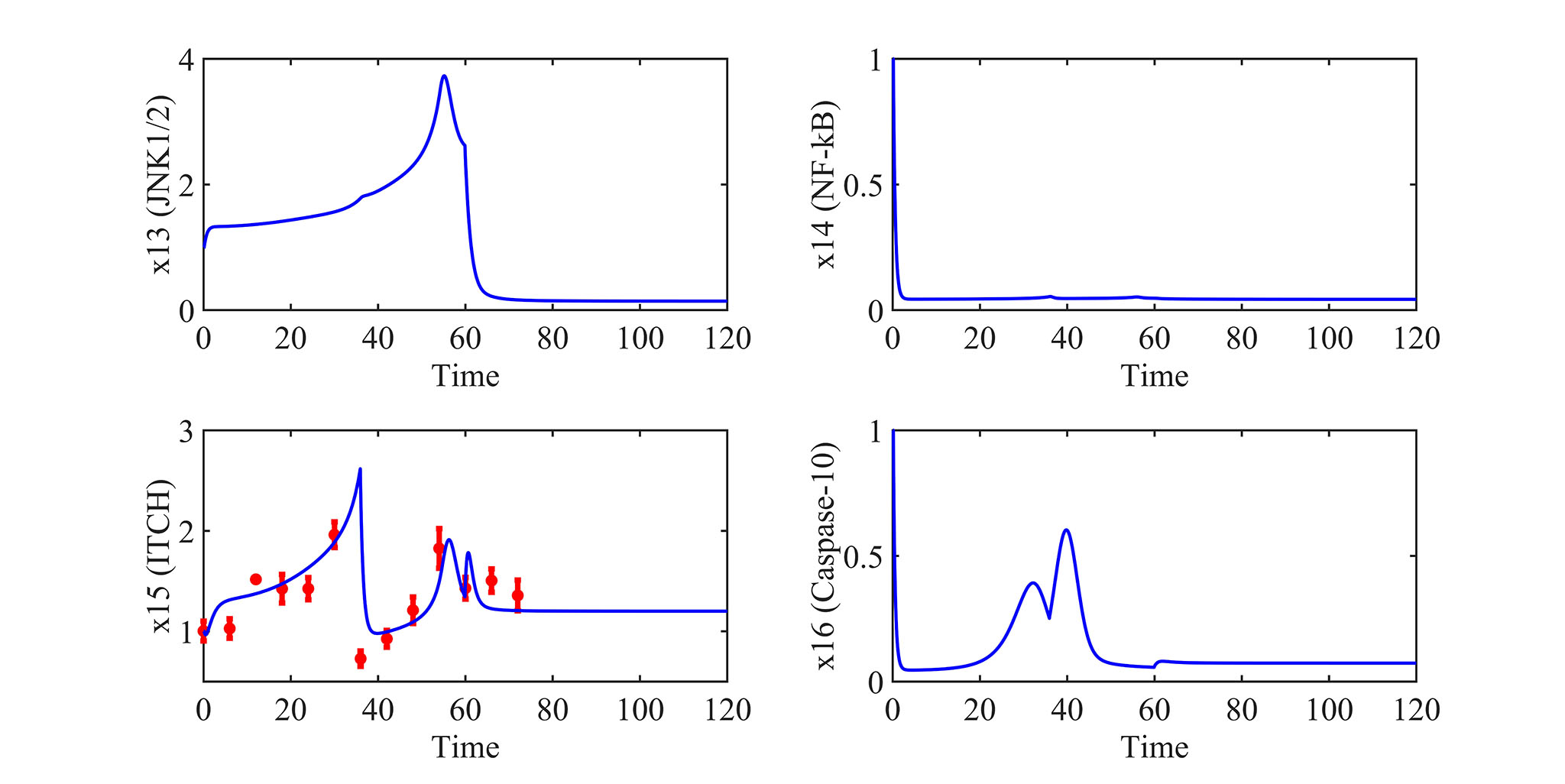

Supplement: Supplementary file 4 [file DataSheet2.zip › Supplementary material_image2/Parameter_d17(中)/4.jpg]

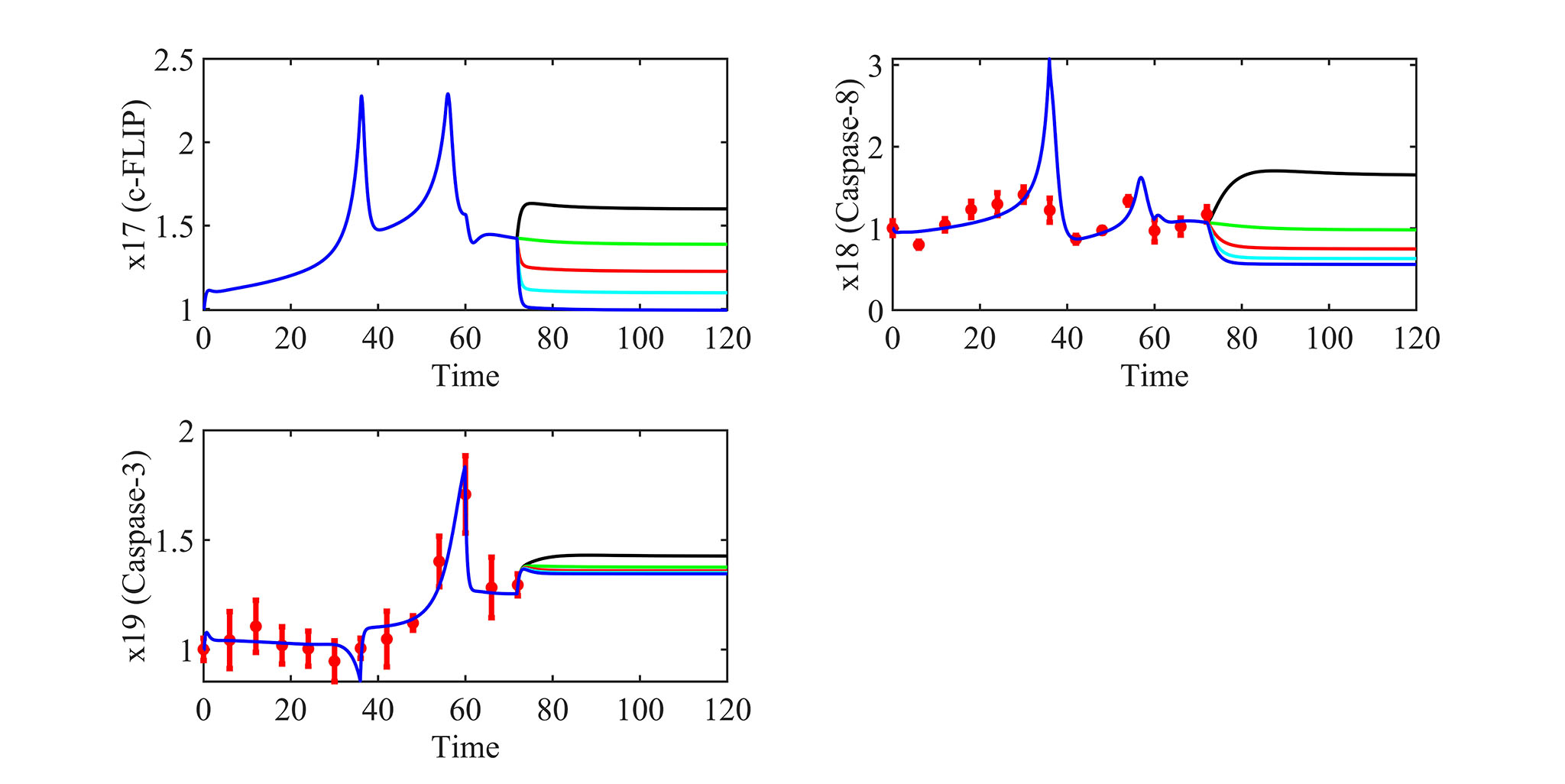

Supplement: Supplementary file 4 [file DataSheet2.zip › Supplementary material_image2/Parameter_d17(中)/5.jpg]

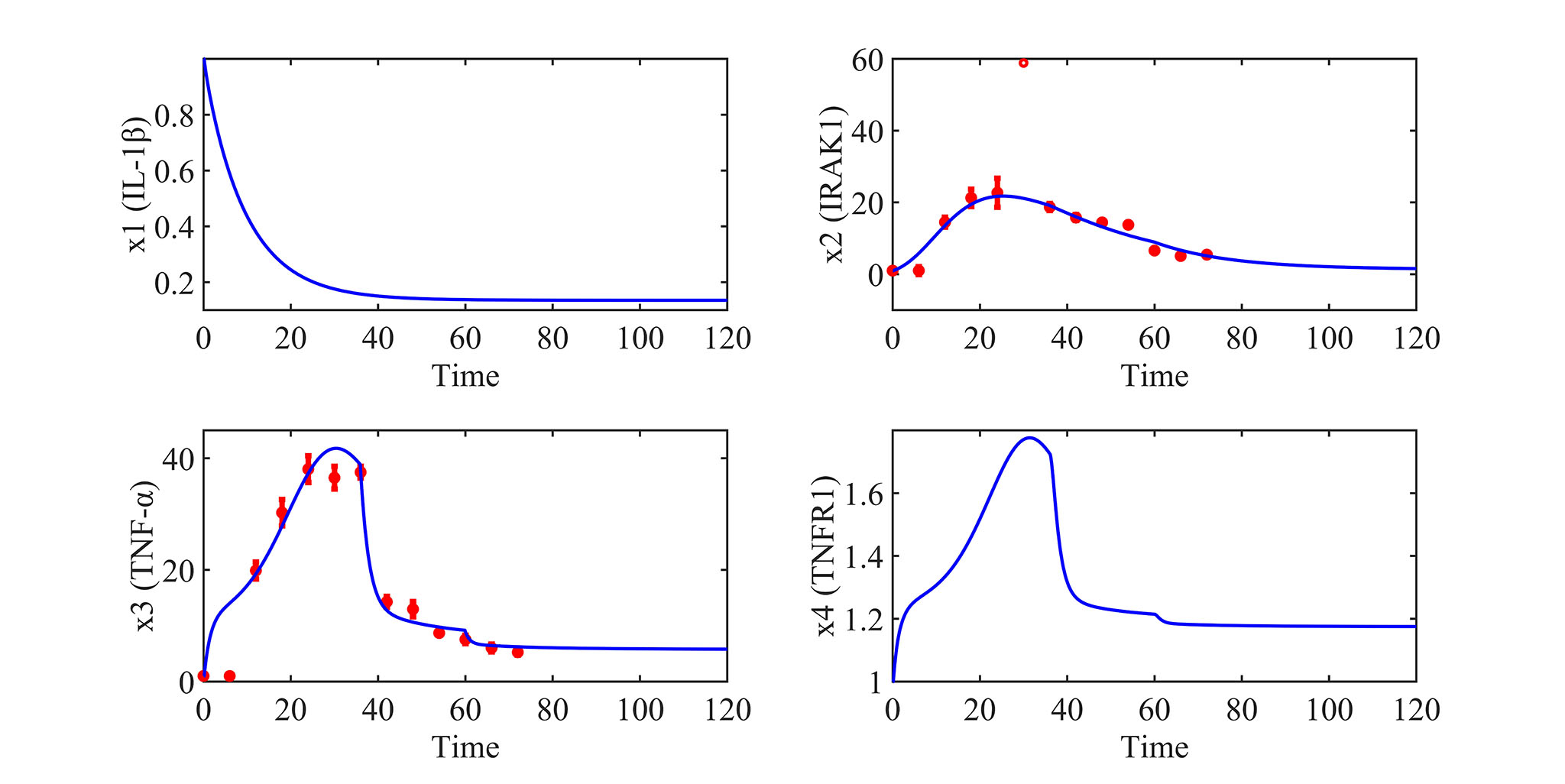

Supplement: Supplementary file 4 [file DataSheet2.zip › Supplementary material_image2/Parameter_d18(小)/1.jpg]

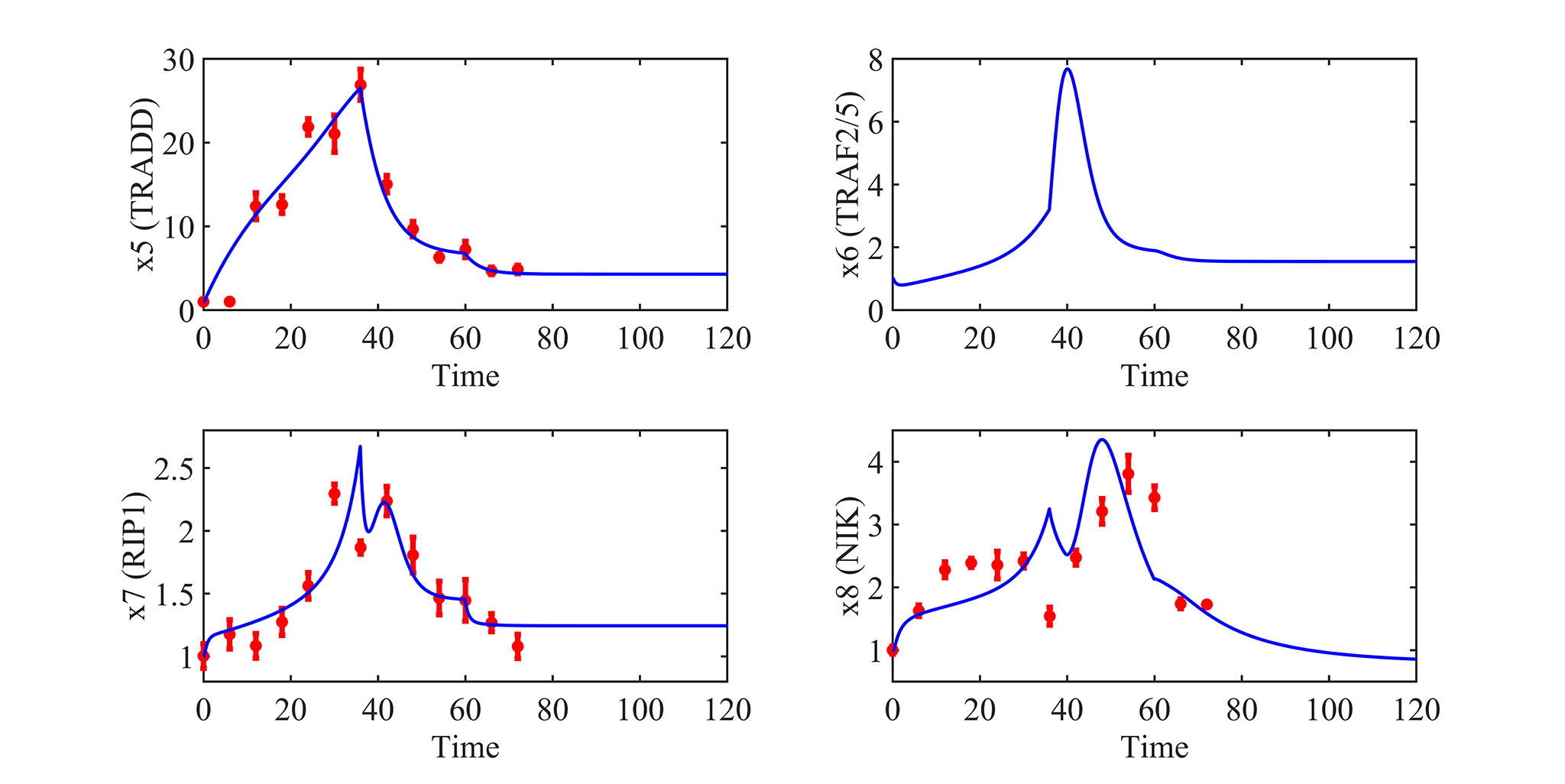

Supplement: Supplementary file 4 [file DataSheet2.zip › Supplementary material_image2/Parameter_d18(小)/2.jpg]

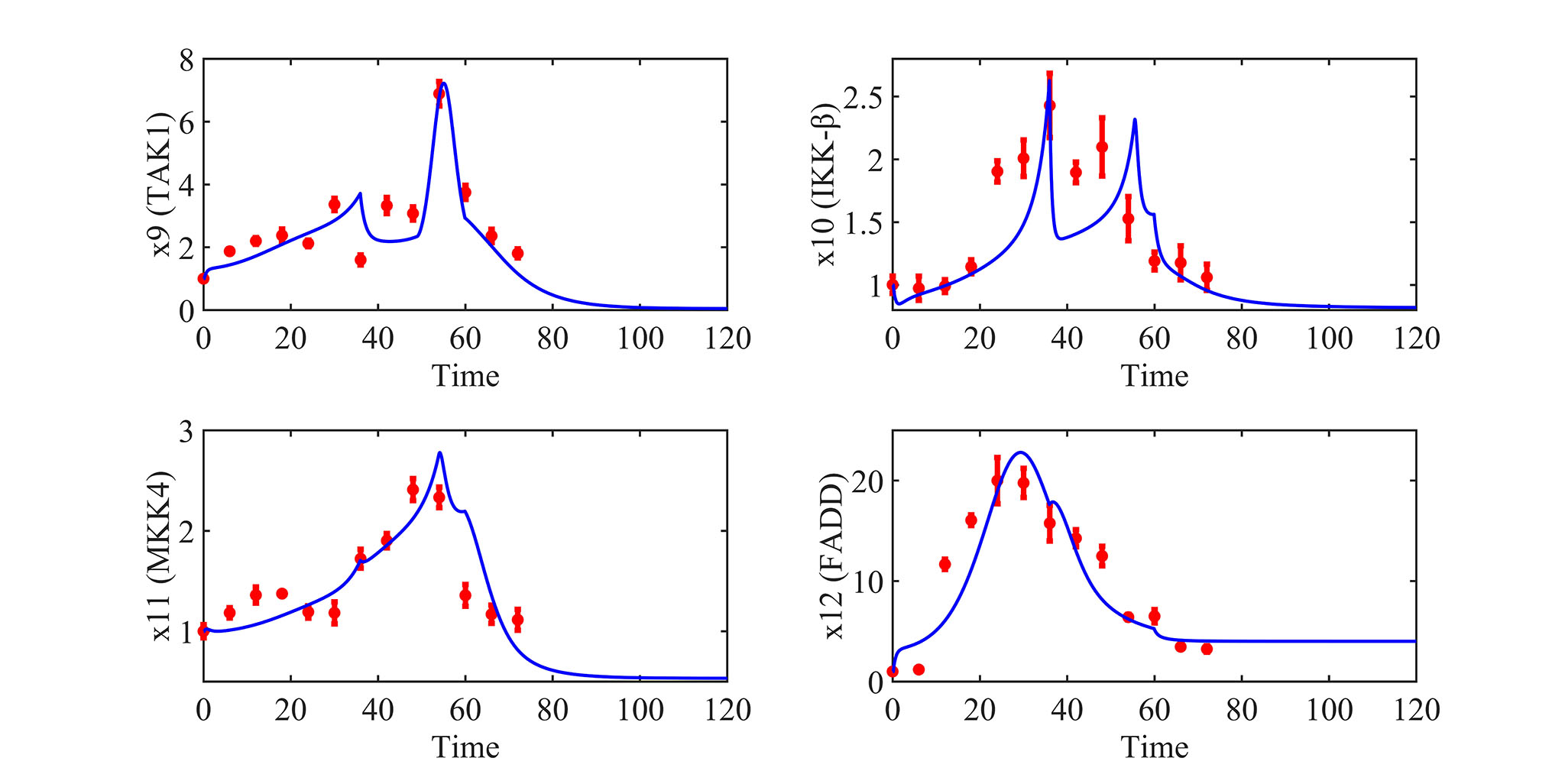

Supplement: Supplementary file 4 [file DataSheet2.zip › Supplementary material_image2/Parameter_d18(小)/3.jpg]

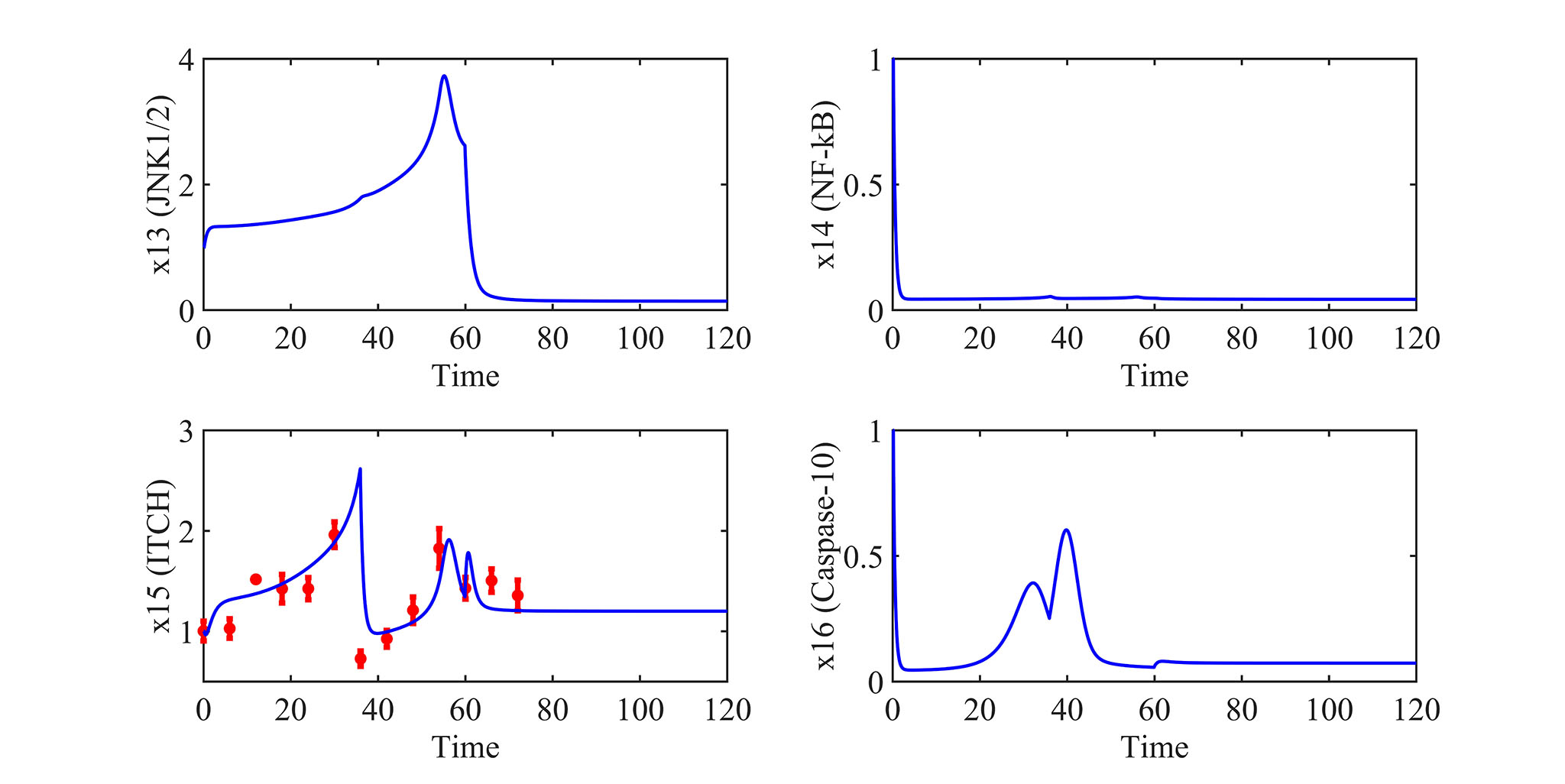

Supplement: Supplementary file 4 [file DataSheet2.zip › Supplementary material_image2/Parameter_d18(小)/4.jpg]

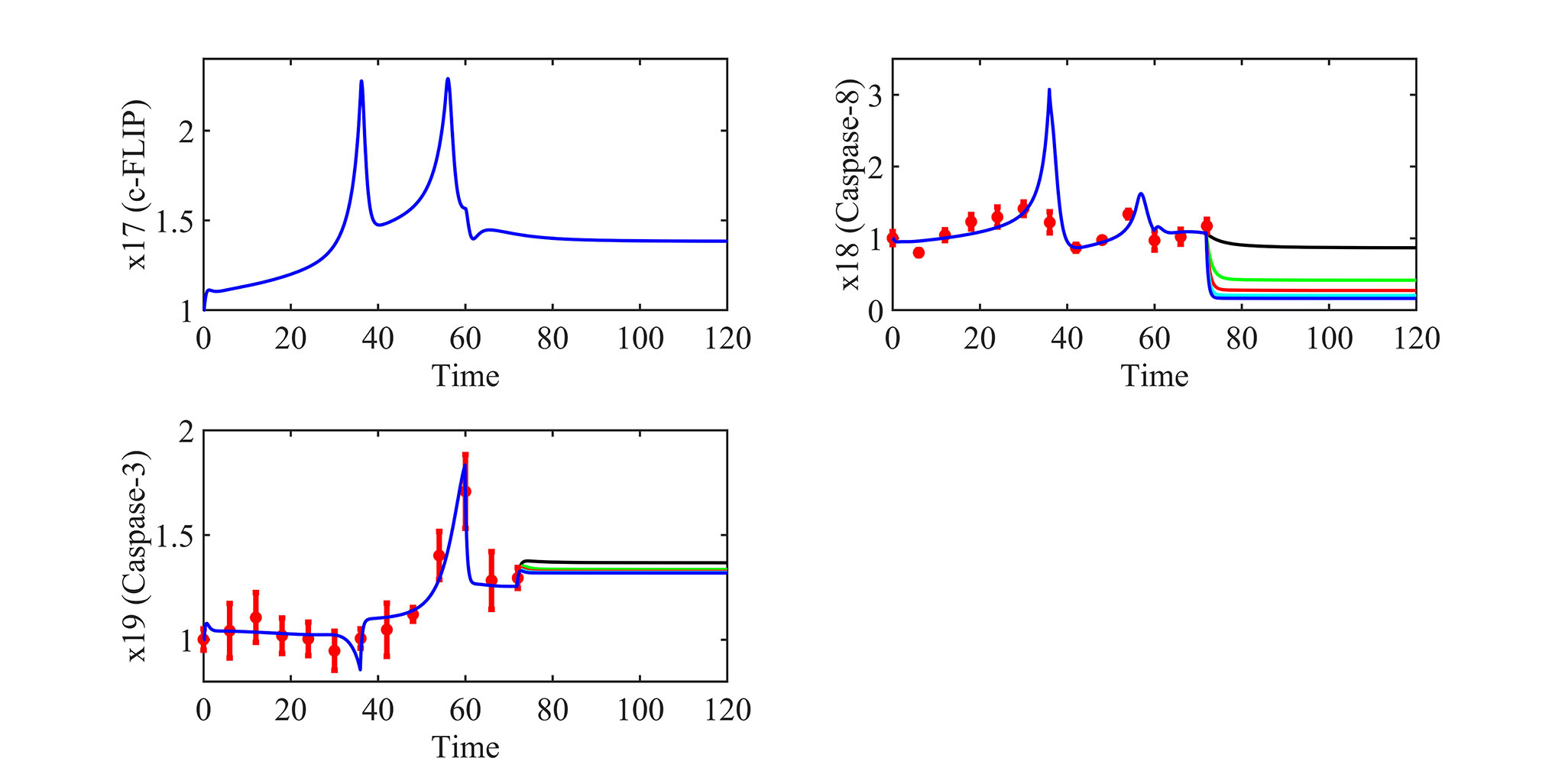

Supplement: Supplementary file 4 [file DataSheet2.zip › Supplementary material_image2/Parameter_d18(小)/5.jpg]

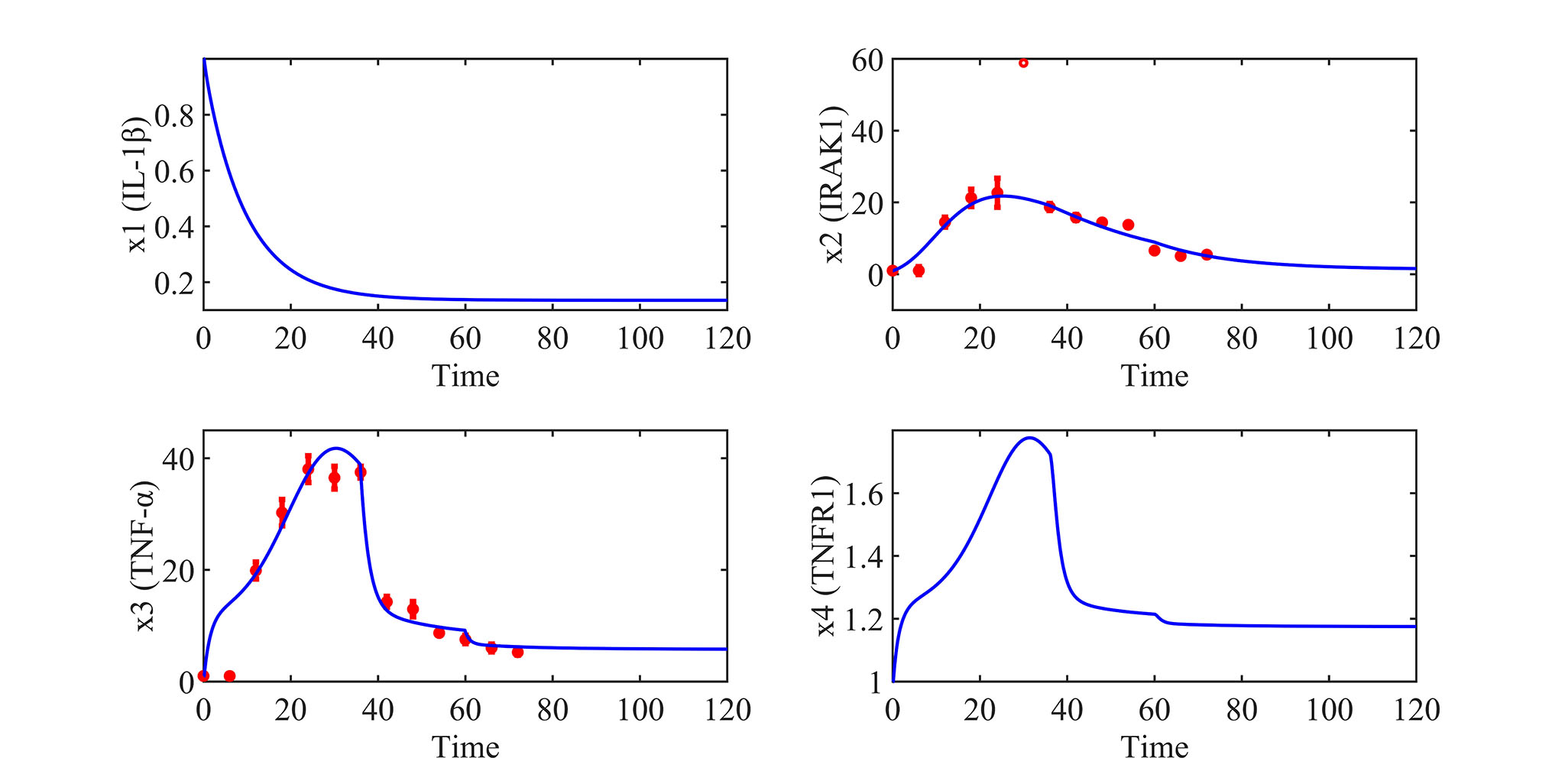

Supplement: Supplementary file 4 [file DataSheet2.zip › Supplementary material_image2/Parameter_d19(大)/1.jpg]

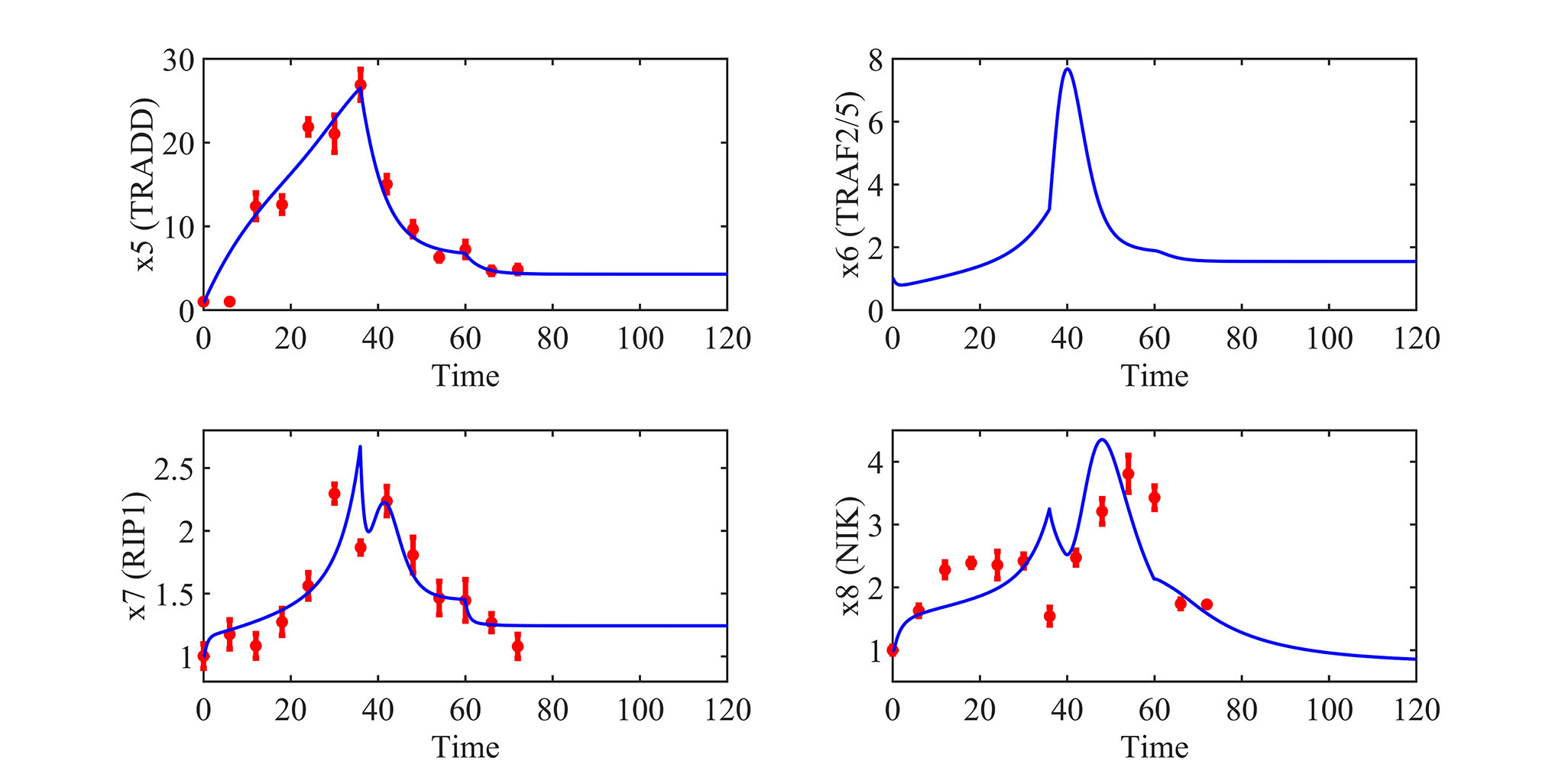

Supplement: Supplementary file 4 [file DataSheet2.zip › Supplementary material_image2/Parameter_d19(大)/2.jpg]

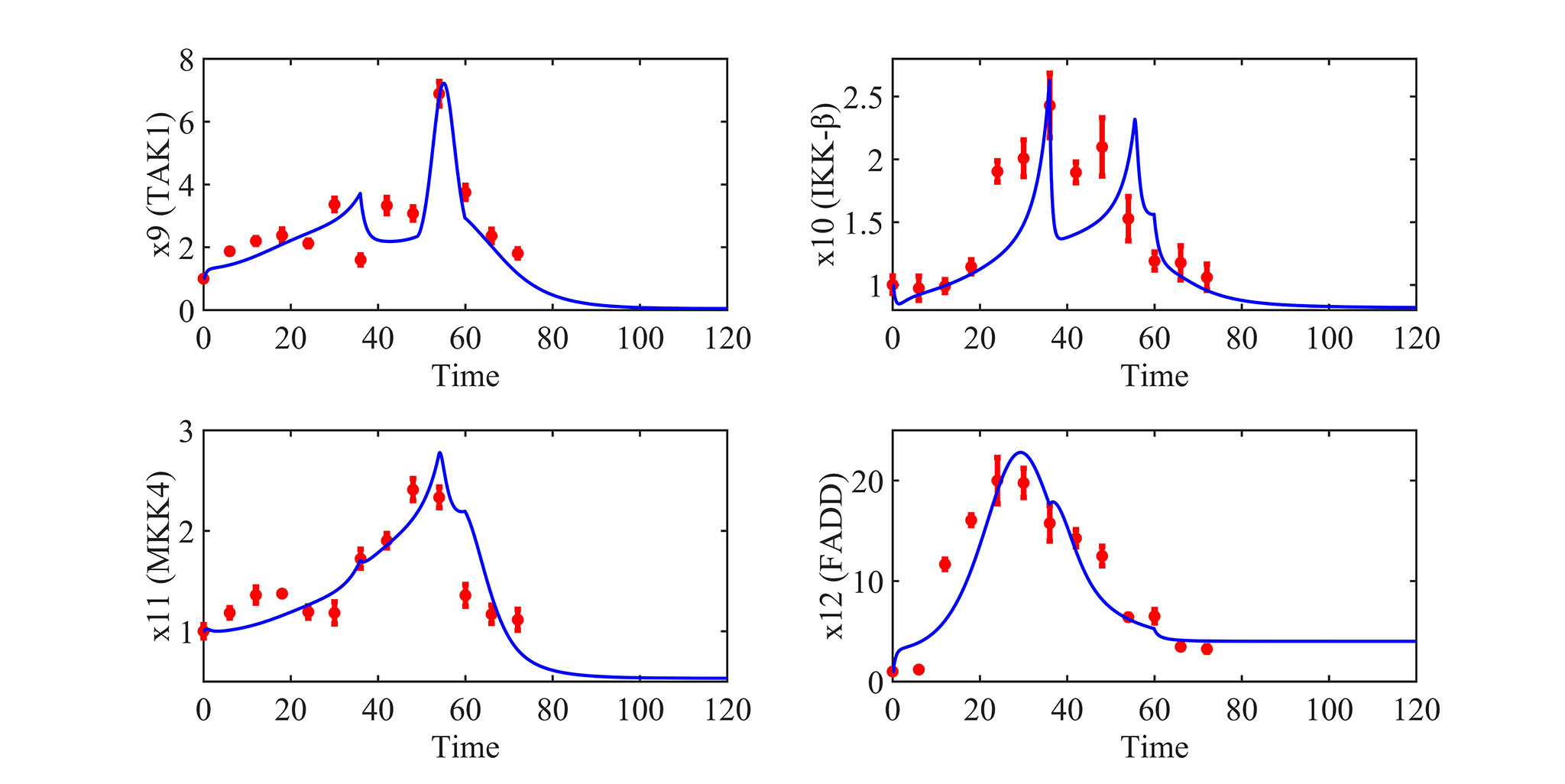

Supplement: Supplementary file 4 [file DataSheet2.zip › Supplementary material_image2/Parameter_d19(大)/3.jpg]

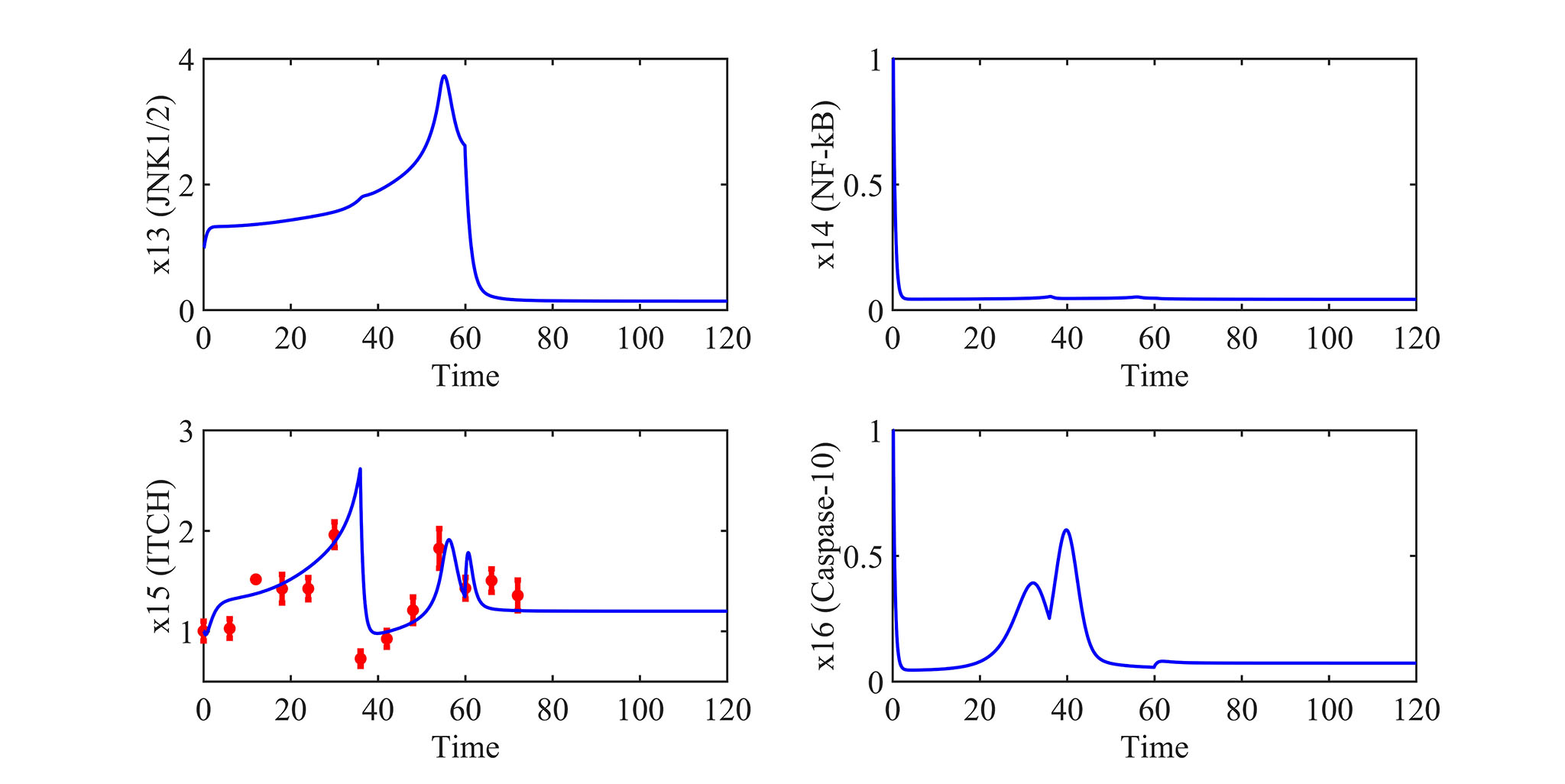

Supplement: Supplementary file 4 [file DataSheet2.zip › Supplementary material_image2/Parameter_d19(大)/4.jpg]

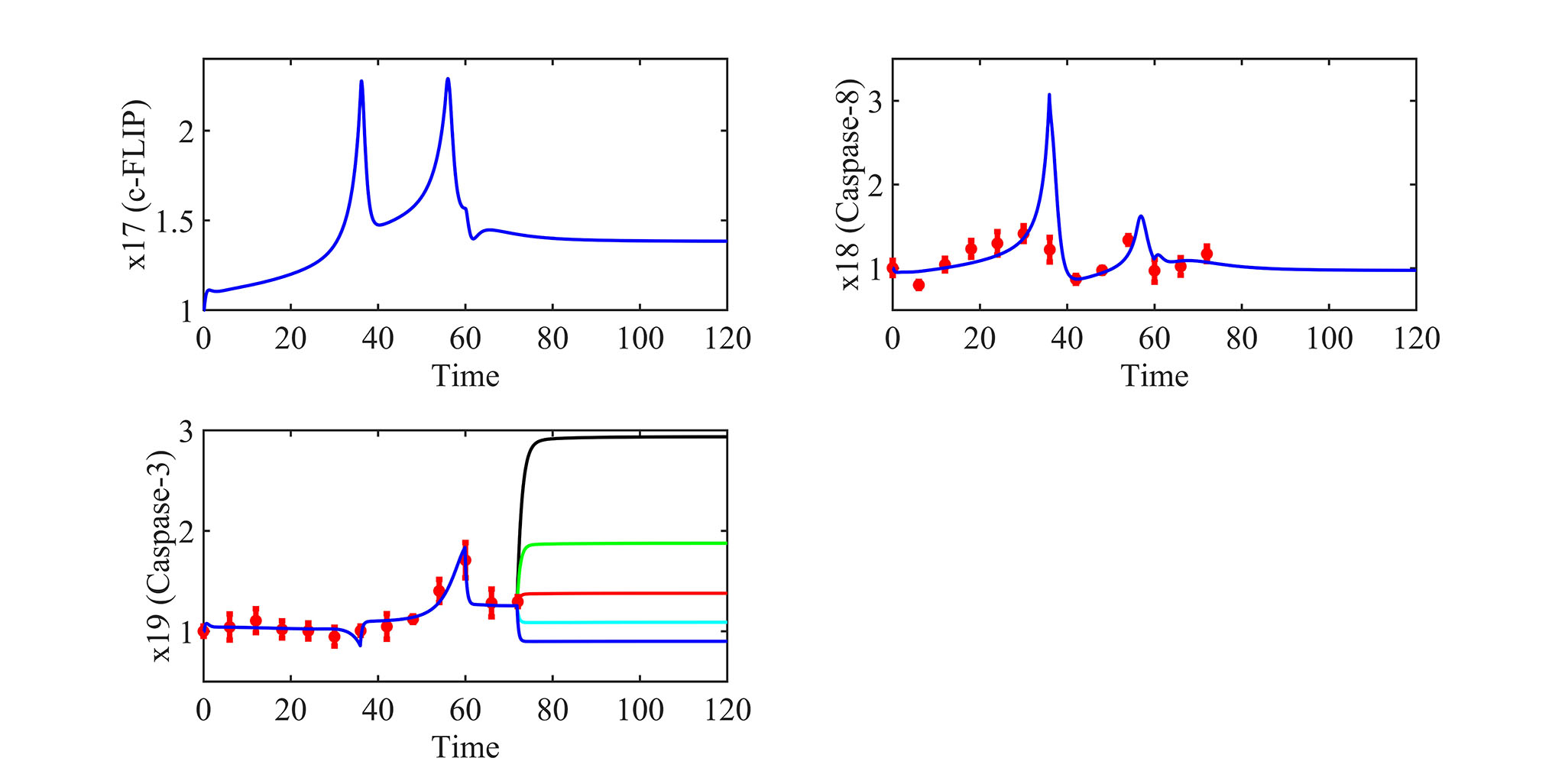

Supplement: Supplementary file 4 [file DataSheet2.zip › Supplementary material_image2/Parameter_d19(大)/5.jpg]

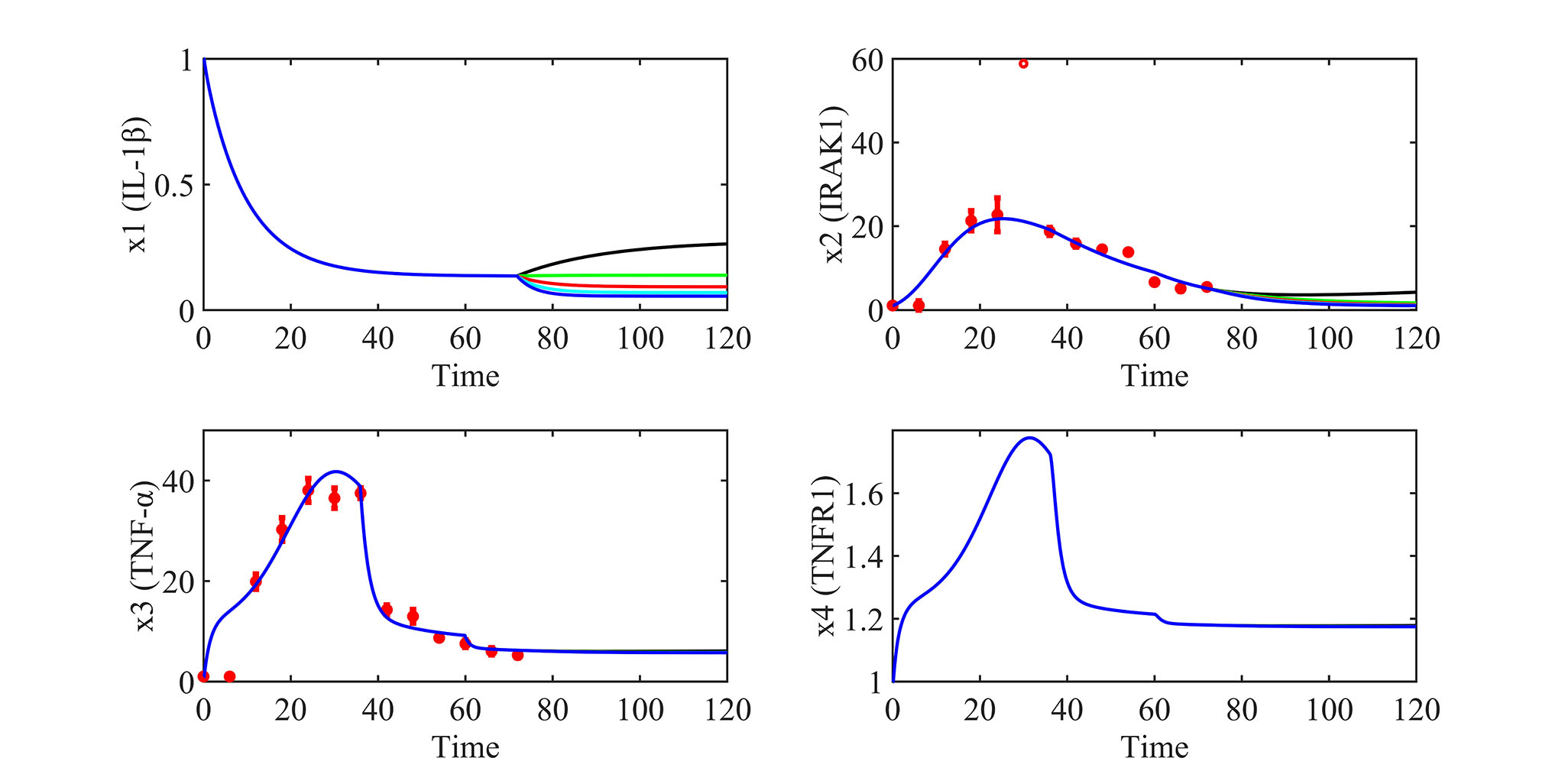

Supplement: Supplementary file 4 [file DataSheet2.zip › Supplementary material_image2/Parameter_d1(小)/1.jpg]

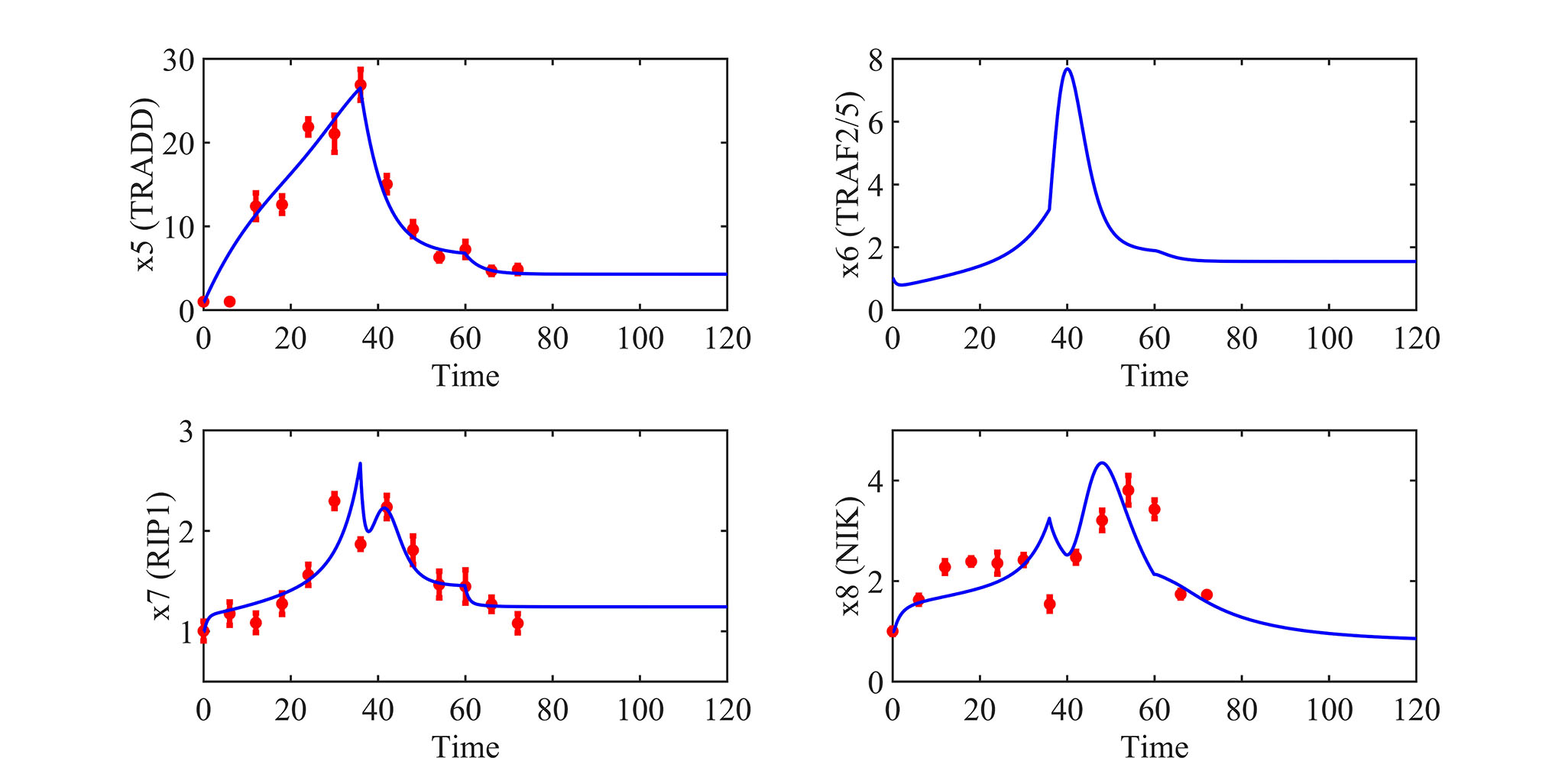

Supplement: Supplementary file 4 [file DataSheet2.zip › Supplementary material_image2/Parameter_d1(小)/2.jpg]

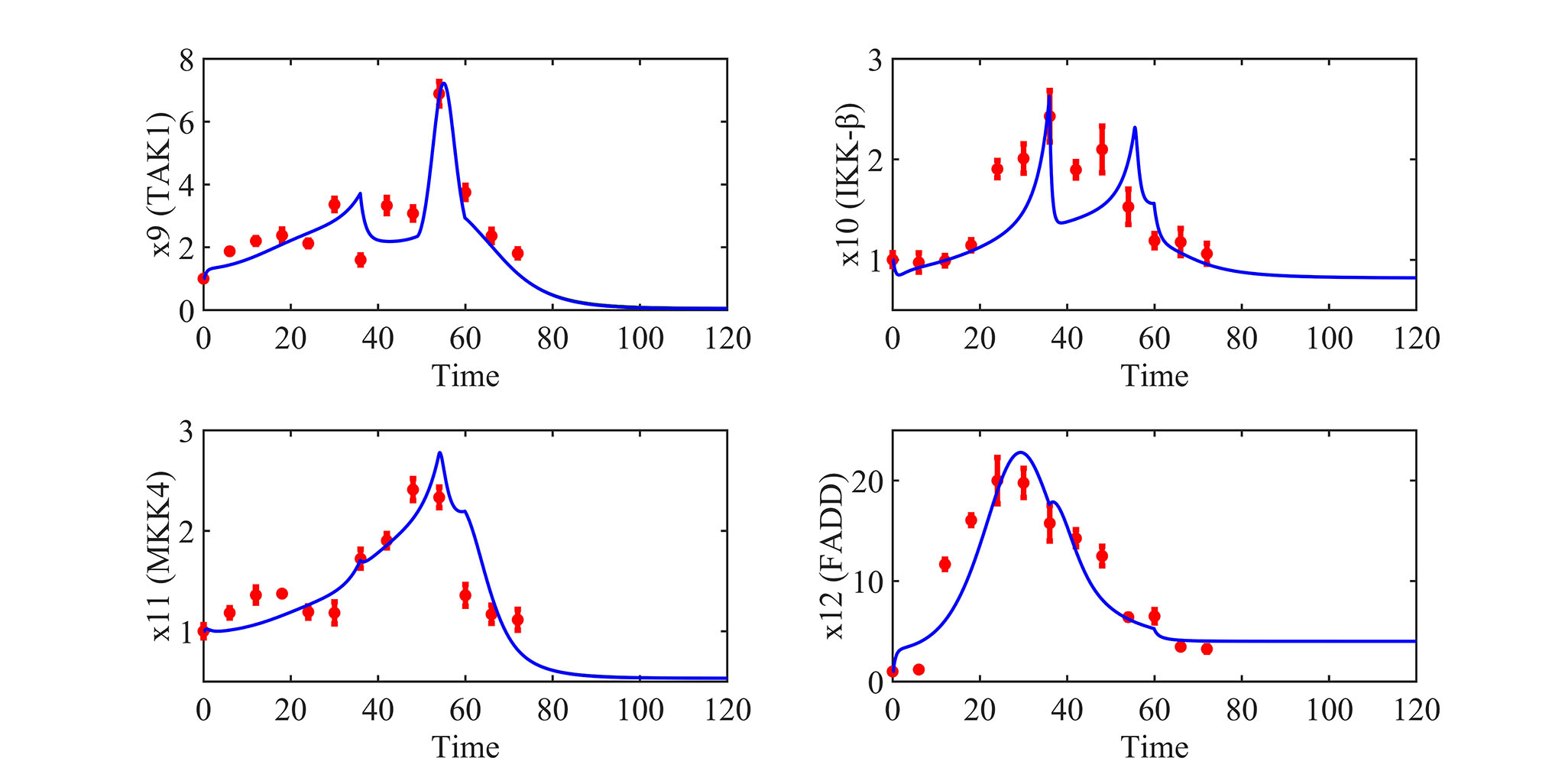

Supplement: Supplementary file 4 [file DataSheet2.zip › Supplementary material_image2/Parameter_d1(小)/3.jpg]

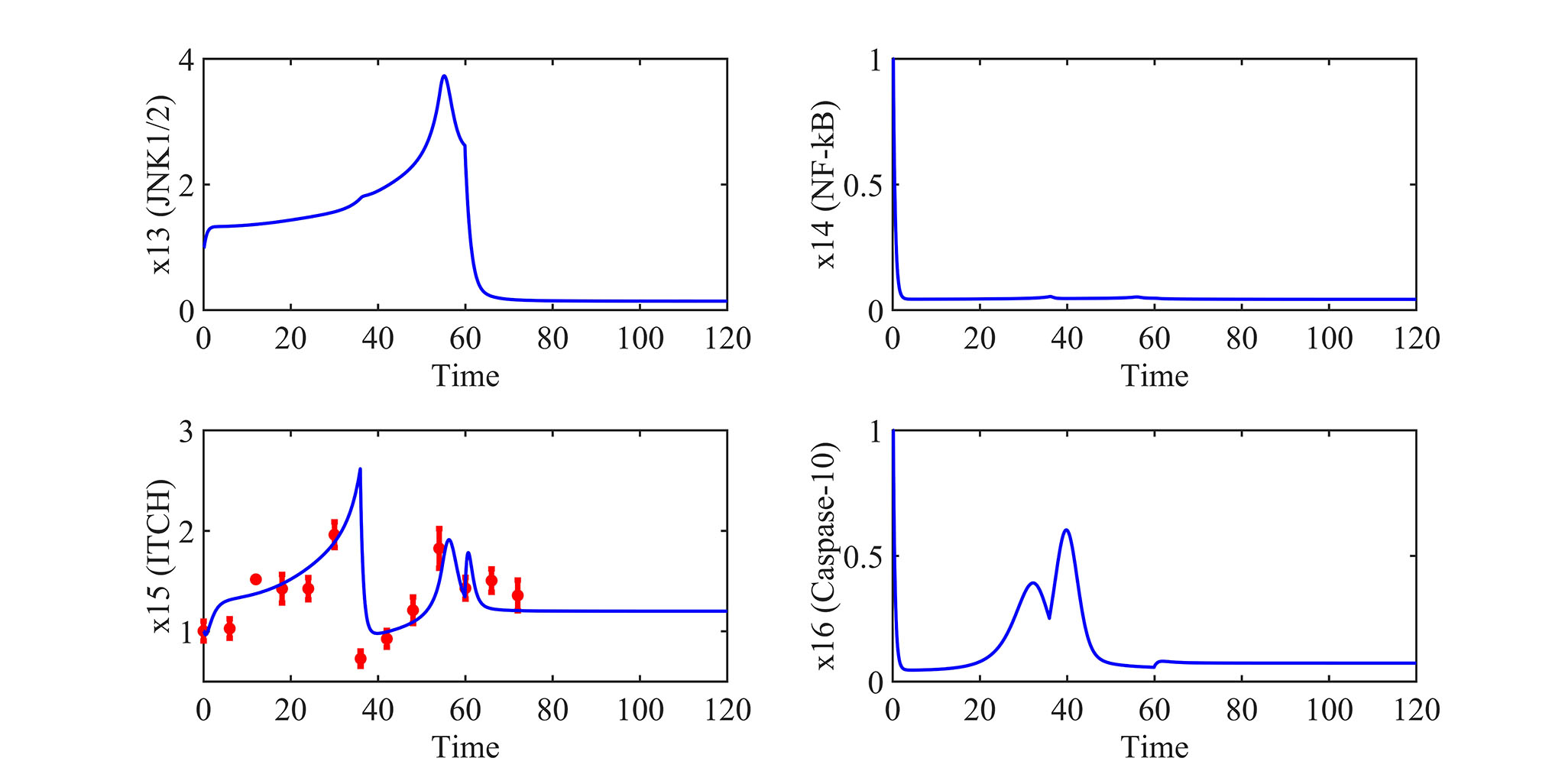

Supplement: Supplementary file 4 [file DataSheet2.zip › Supplementary material_image2/Parameter_d1(小)/4.jpg]

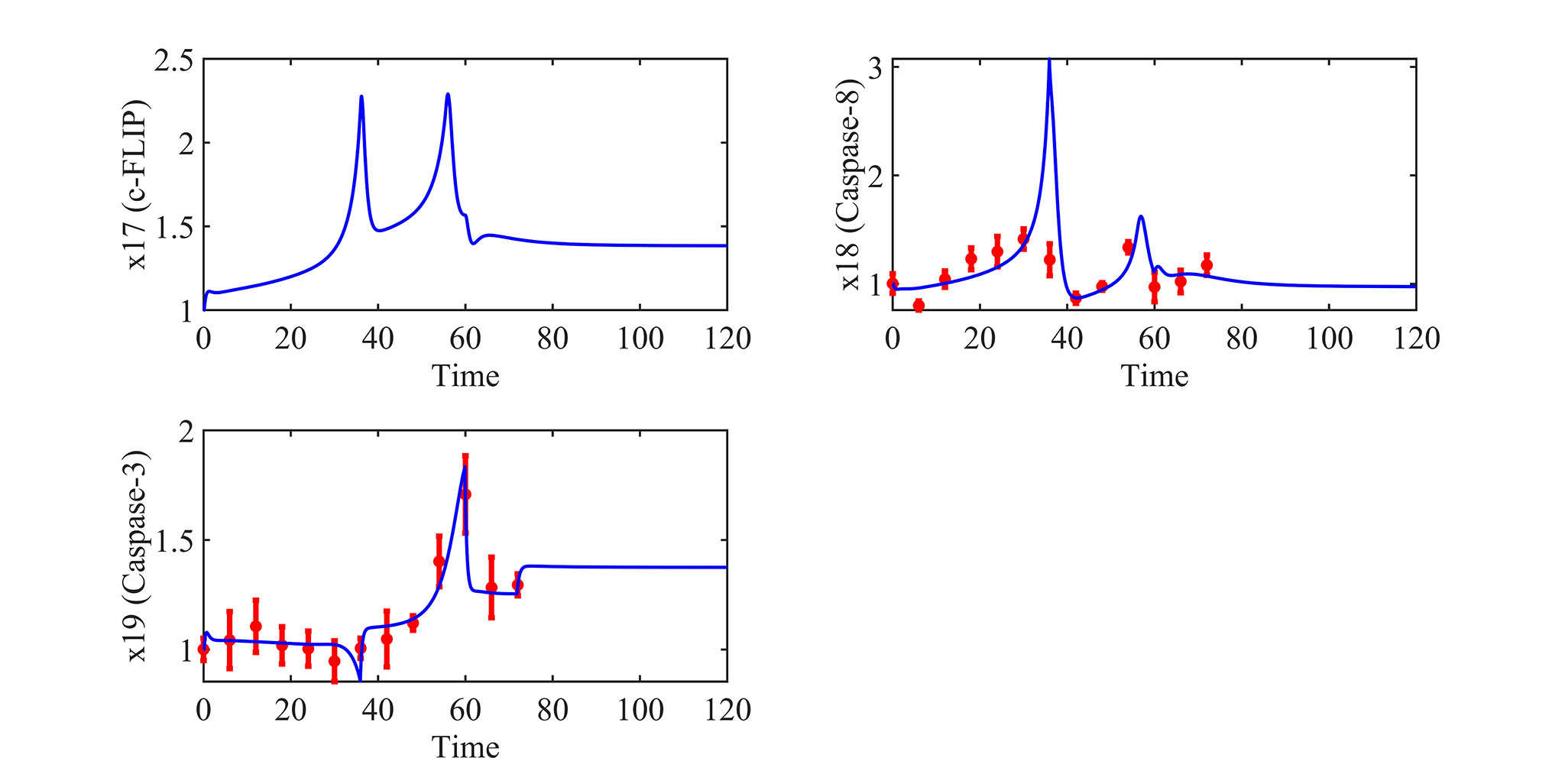

Supplement: Supplementary file 4 [file DataSheet2.zip › Supplementary material_image2/Parameter_d1(小)/5.jpg]

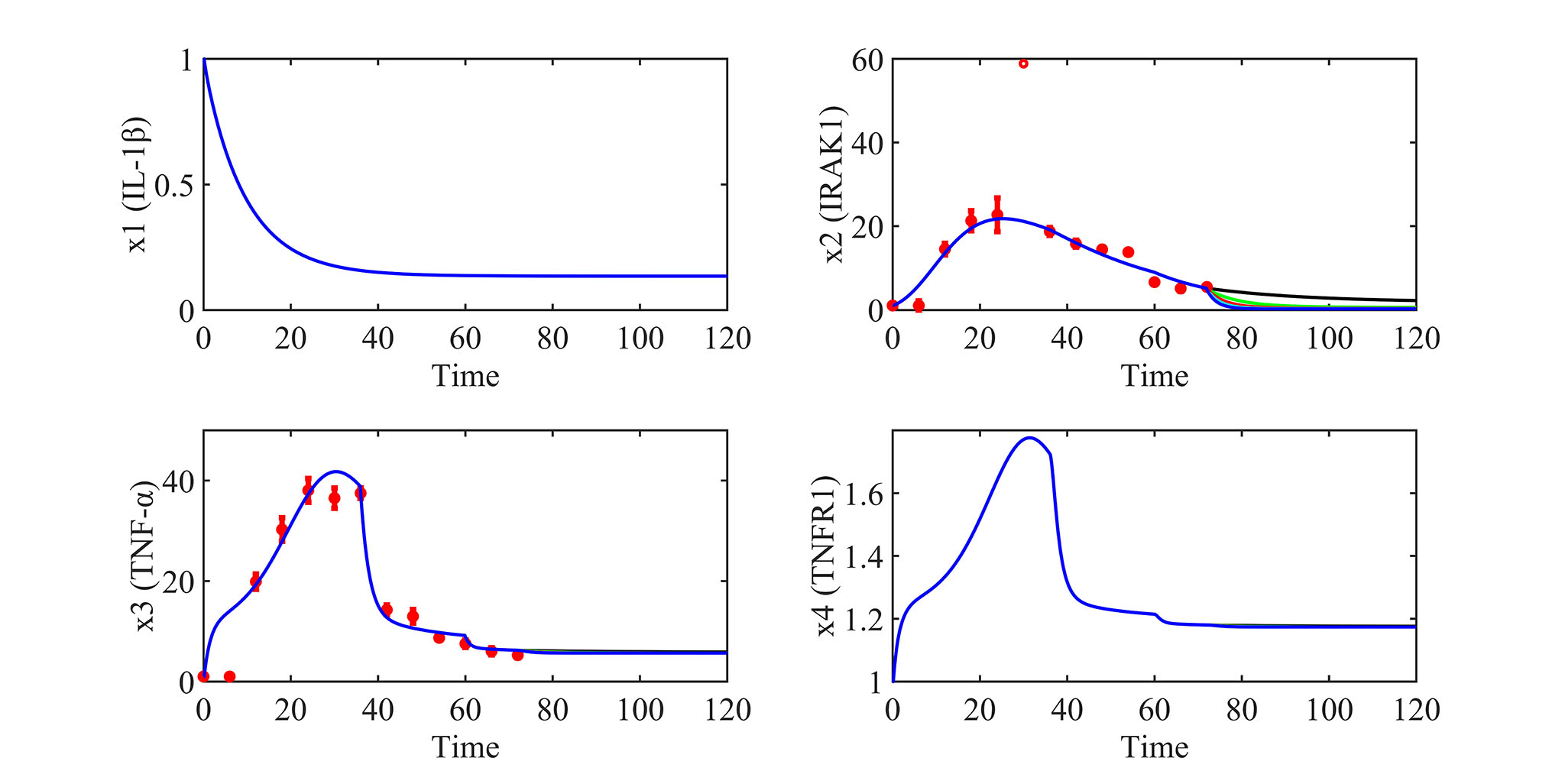

Supplement: Supplementary file 4 [file DataSheet2.zip › Supplementary material_image2/Parameter_d2(小)/1.jpg]

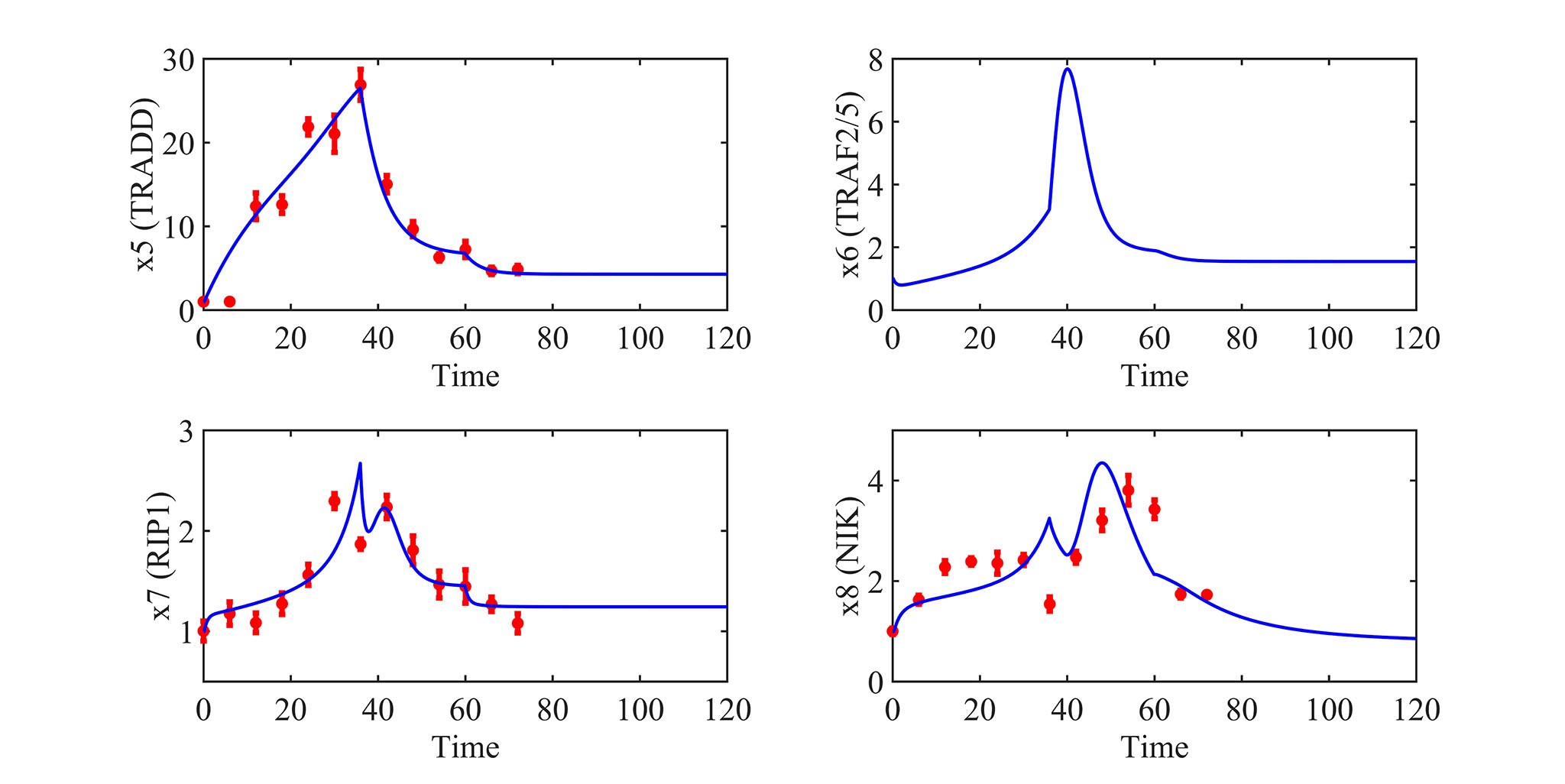

Supplement: Supplementary file 4 [file DataSheet2.zip › Supplementary material_image2/Parameter_d2(小)/2.jpg]

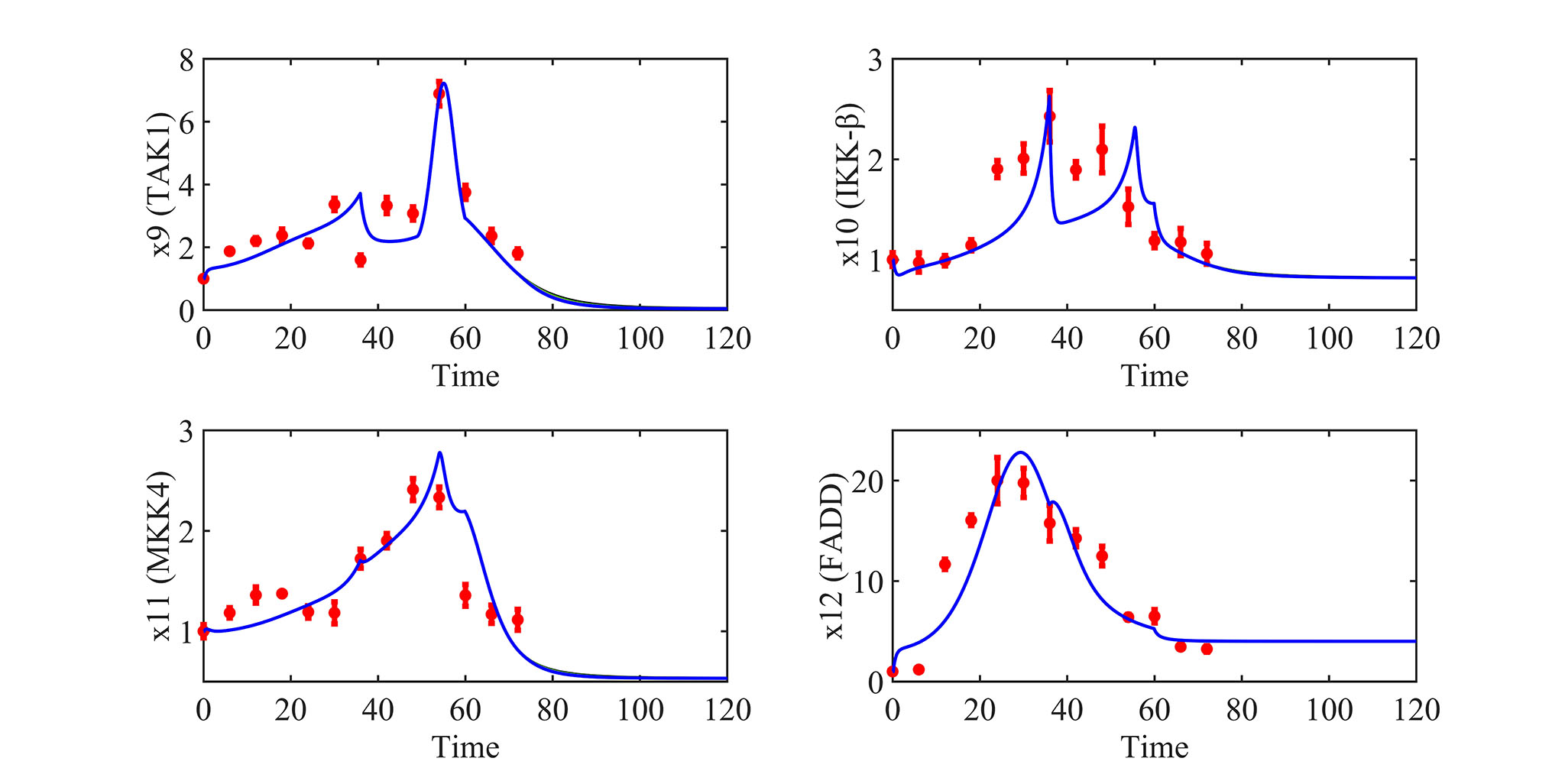

Supplement: Supplementary file 4 [file DataSheet2.zip › Supplementary material_image2/Parameter_d2(小)/3.jpg]

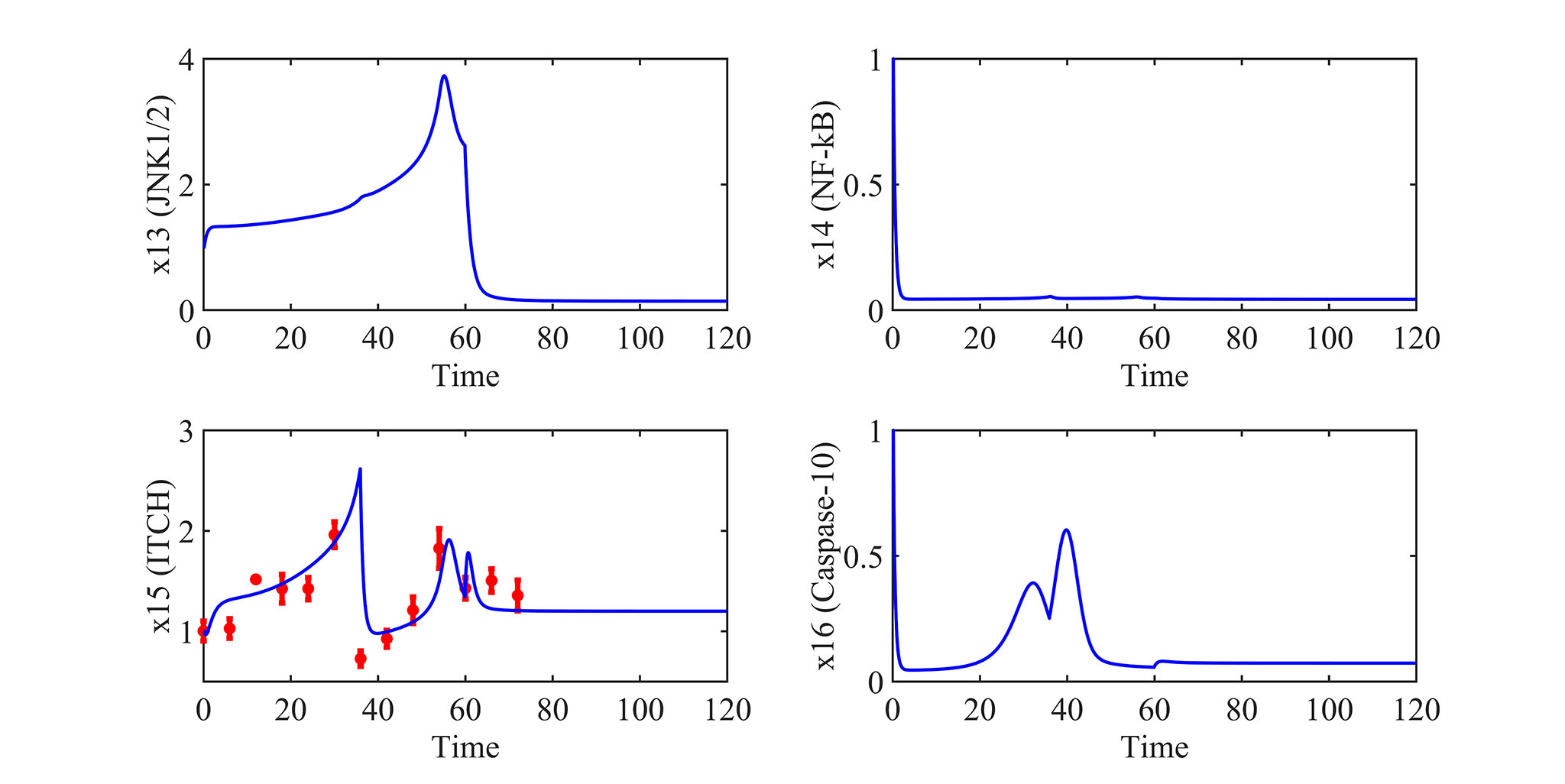

Supplement: Supplementary file 4 [file DataSheet2.zip › Supplementary material_image2/Parameter_d2(小)/4.jpg]

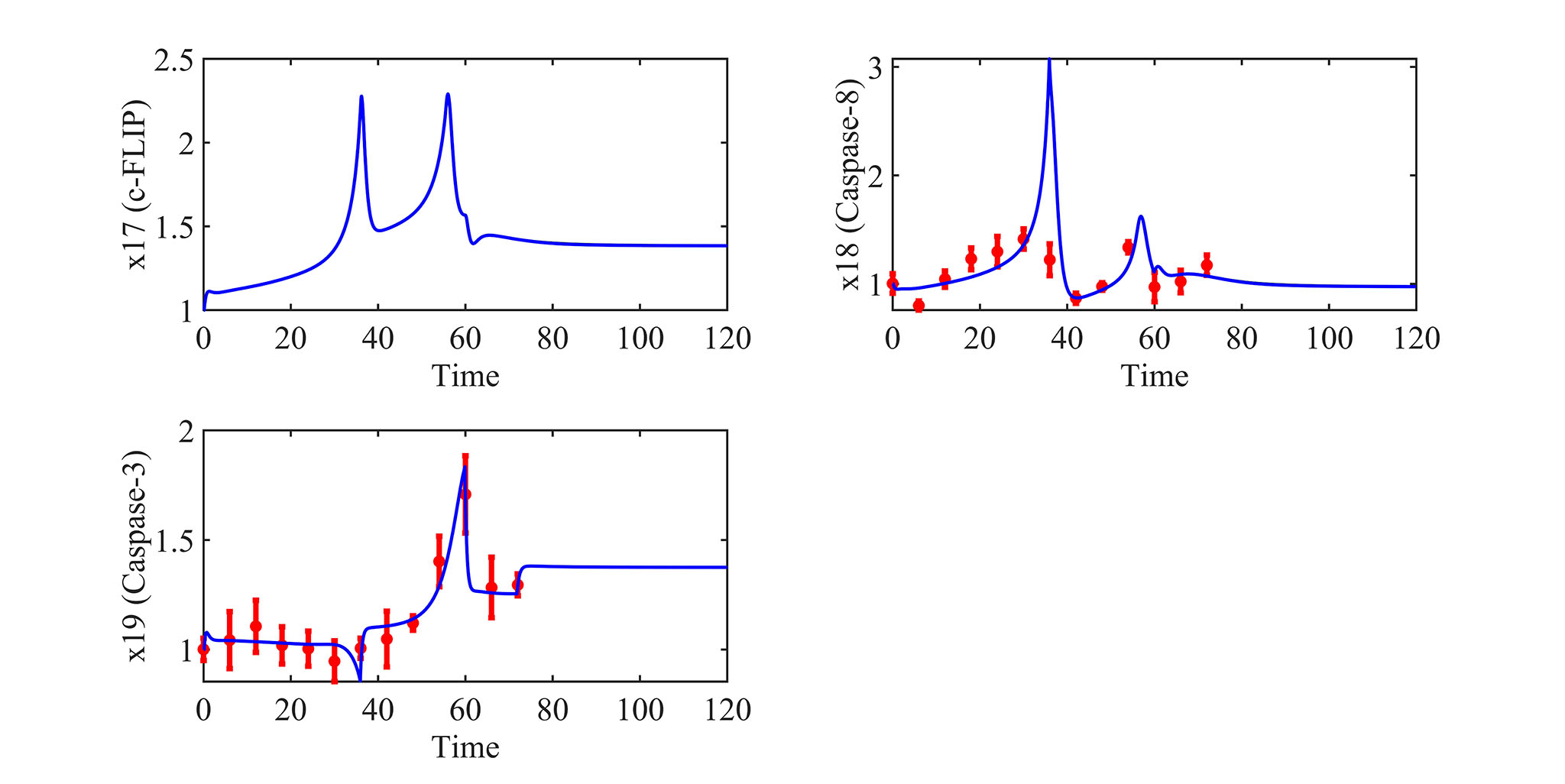

Supplement: Supplementary file 4 [file DataSheet2.zip › Supplementary material_image2/Parameter_d2(小)/5.jpg]

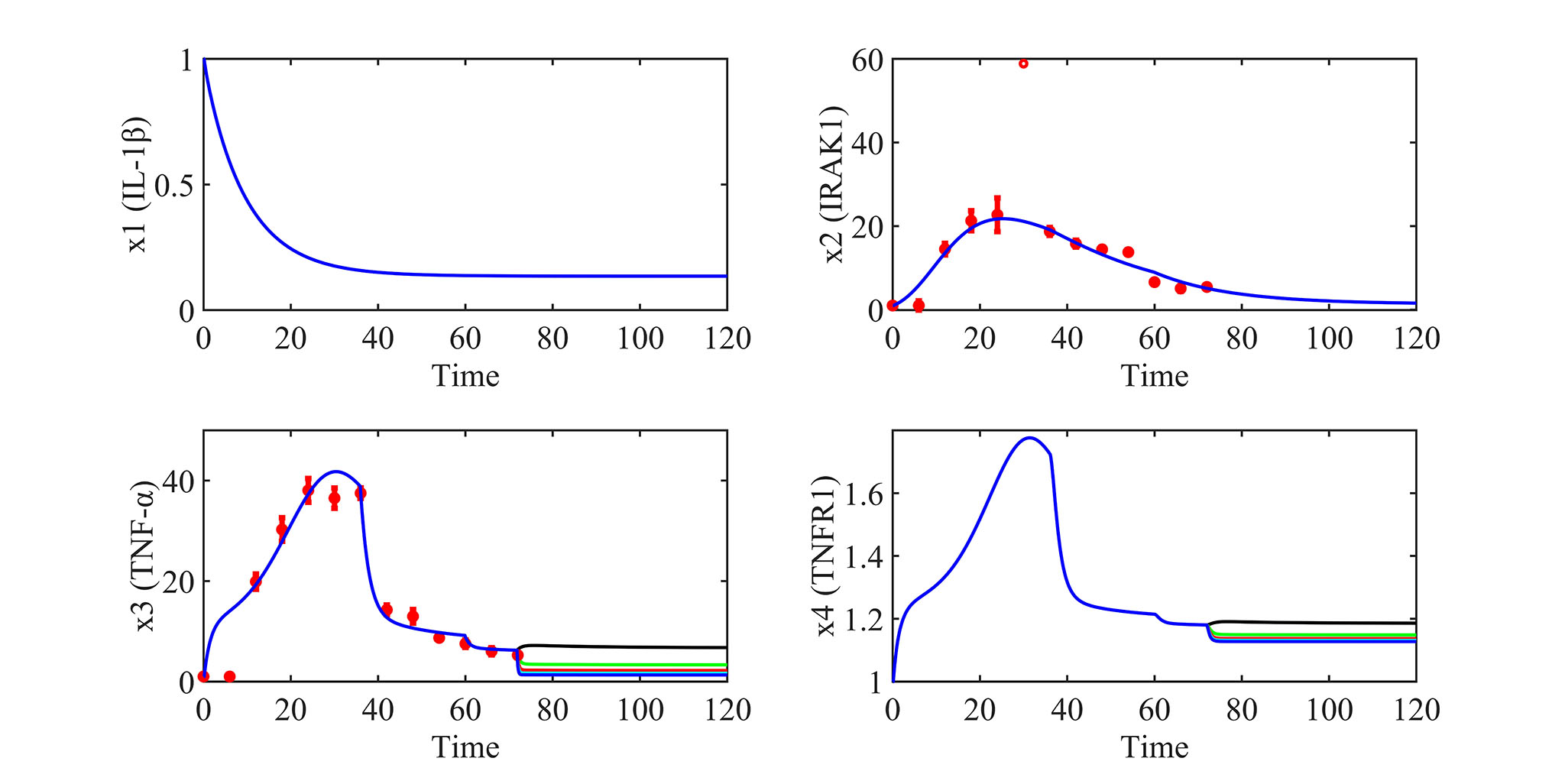

Supplement: Supplementary file 4 [file DataSheet2.zip › Supplementary material_image2/Parameter_d3(小)/1.jpg]

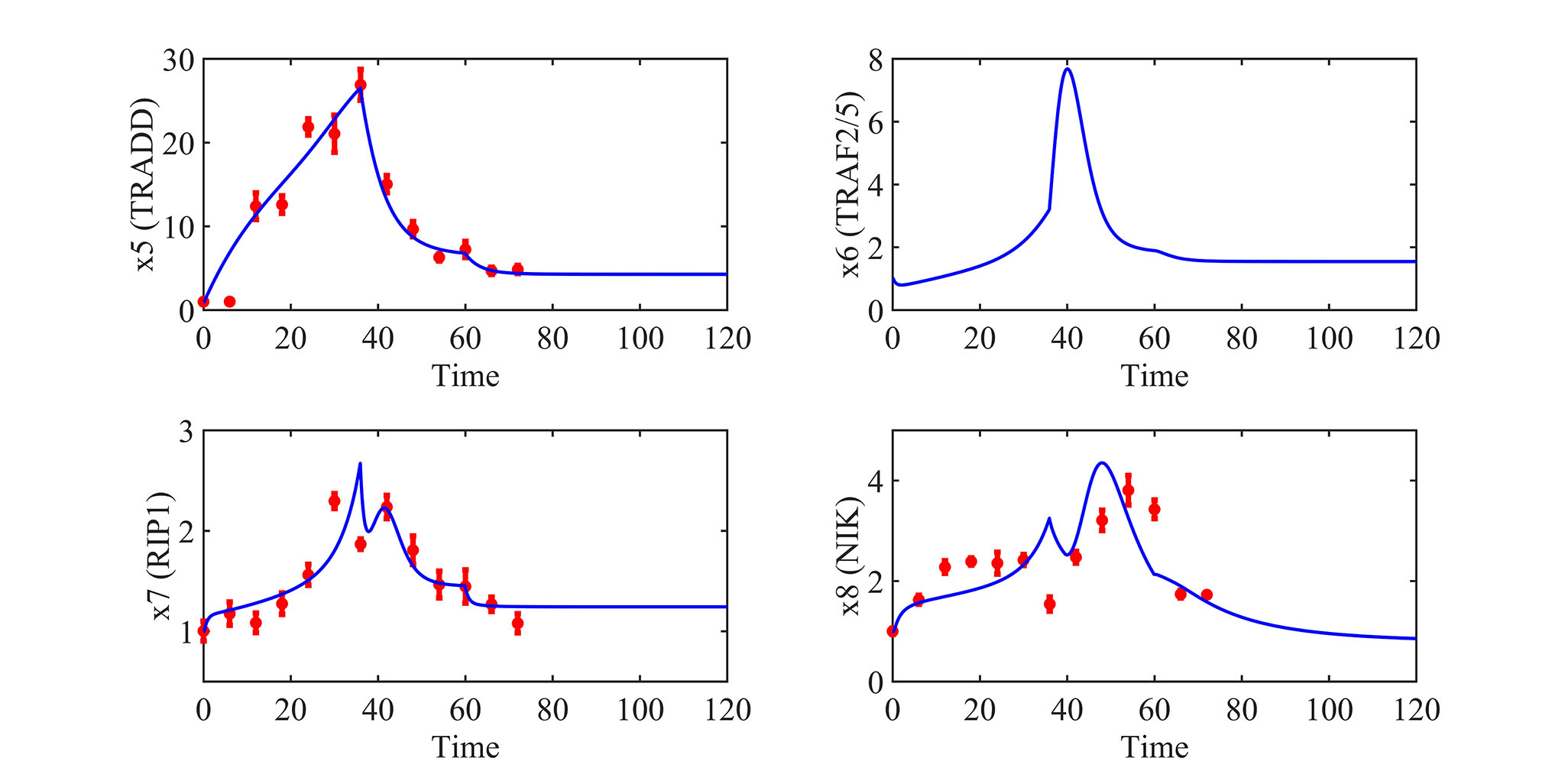

Supplement: Supplementary file 4 [file DataSheet2.zip › Supplementary material_image2/Parameter_d3(小)/2.jpg]

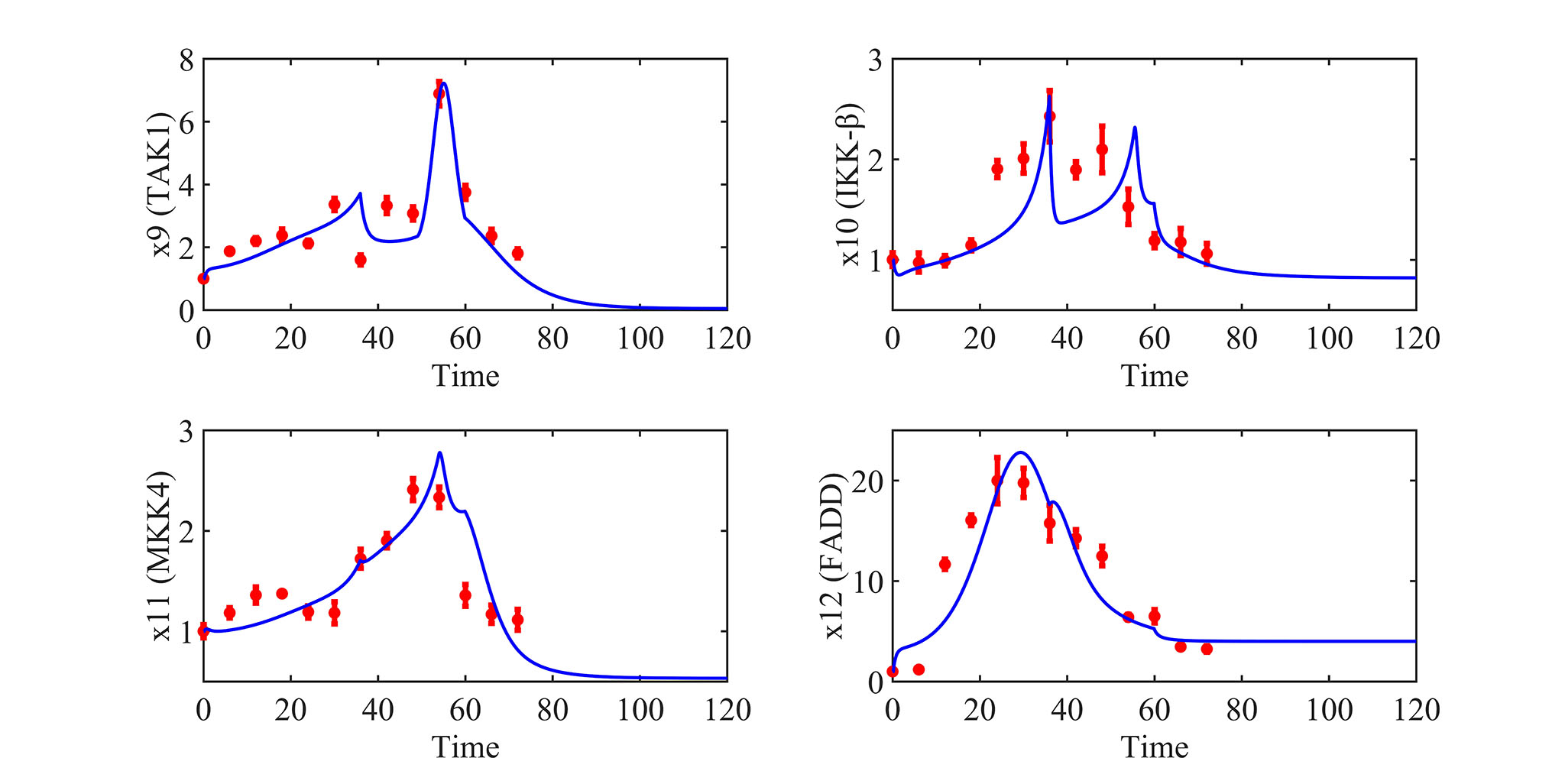

Supplement: Supplementary file 4 [file DataSheet2.zip › Supplementary material_image2/Parameter_d3(小)/3.jpg]

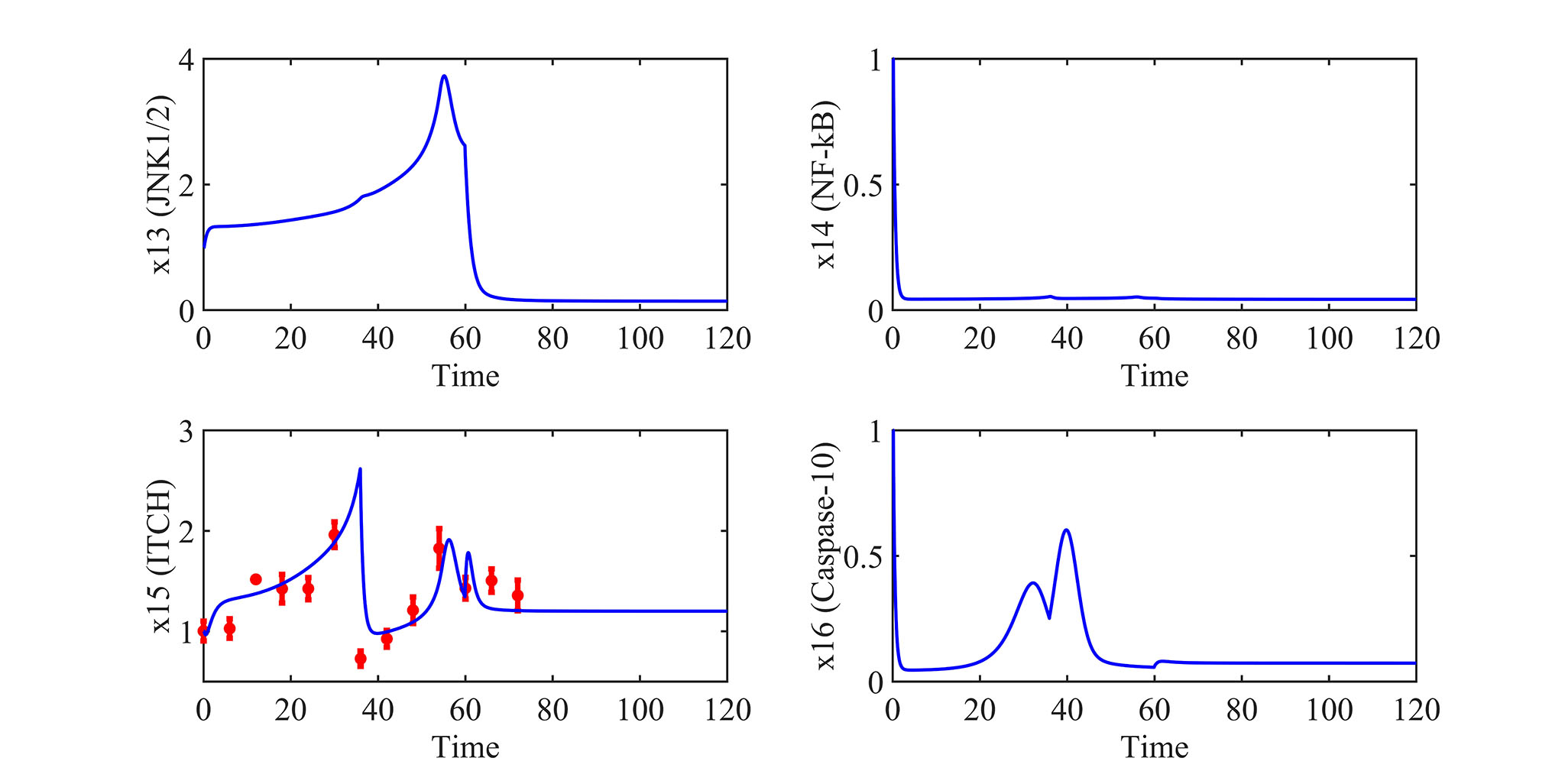

Supplement: Supplementary file 4 [file DataSheet2.zip › Supplementary material_image2/Parameter_d3(小)/4.jpg]

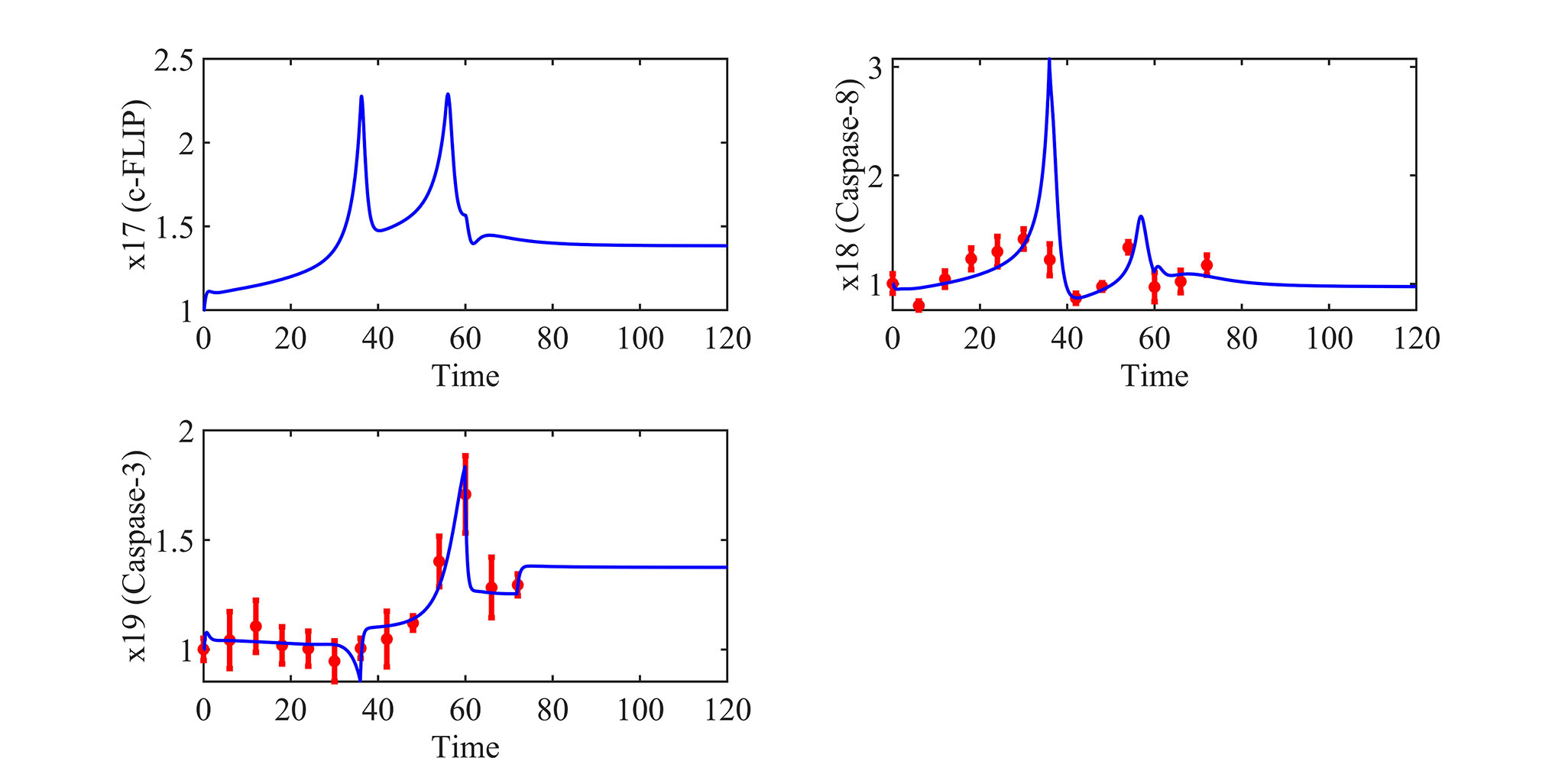

Supplement: Supplementary file 4 [file DataSheet2.zip › Supplementary material_image2/Parameter_d3(小)/5.jpg]

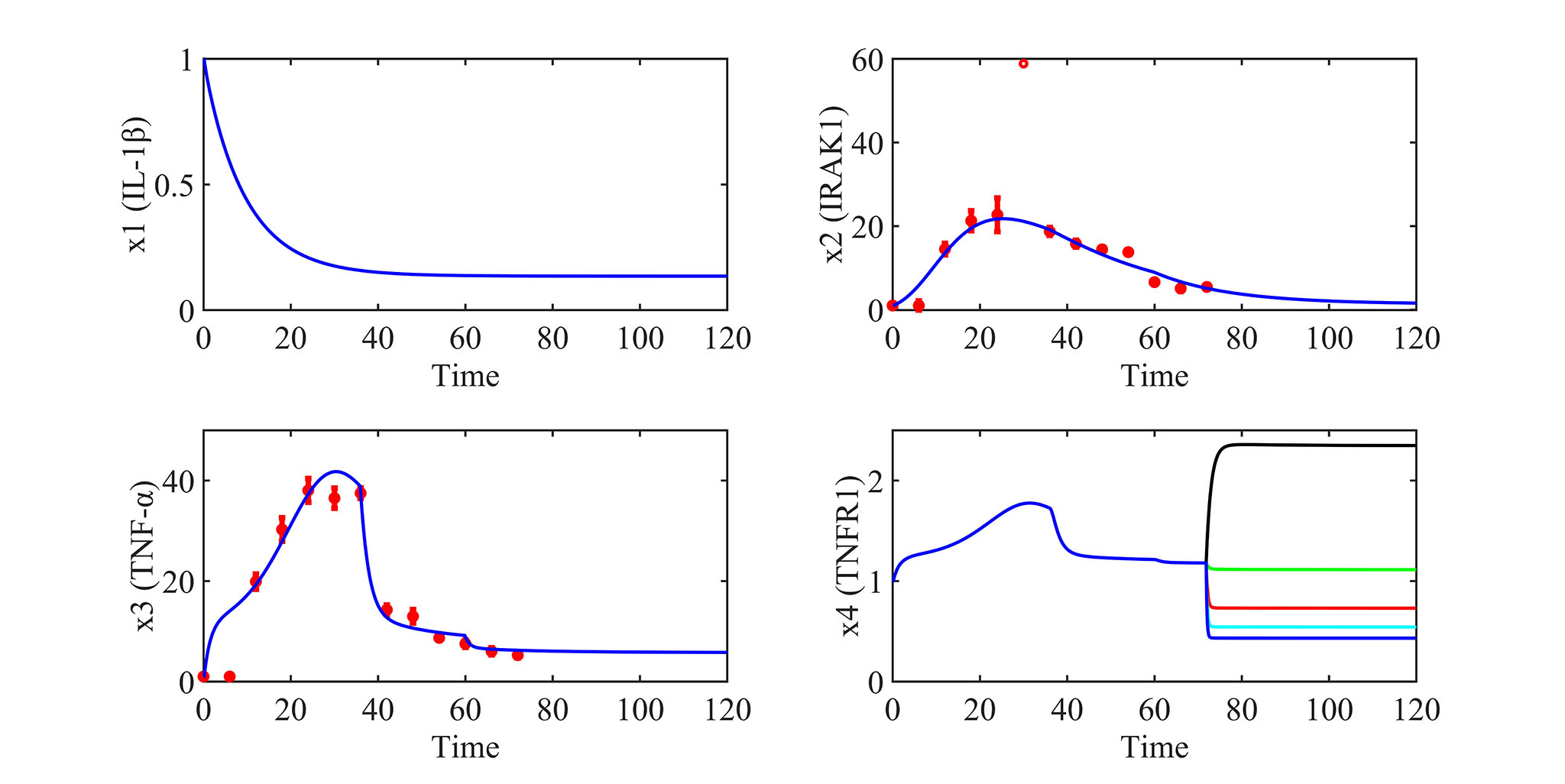

Supplement: Supplementary file 4 [file DataSheet2.zip › Supplementary material_image2/Parameter_d4(小)/1.jpg]

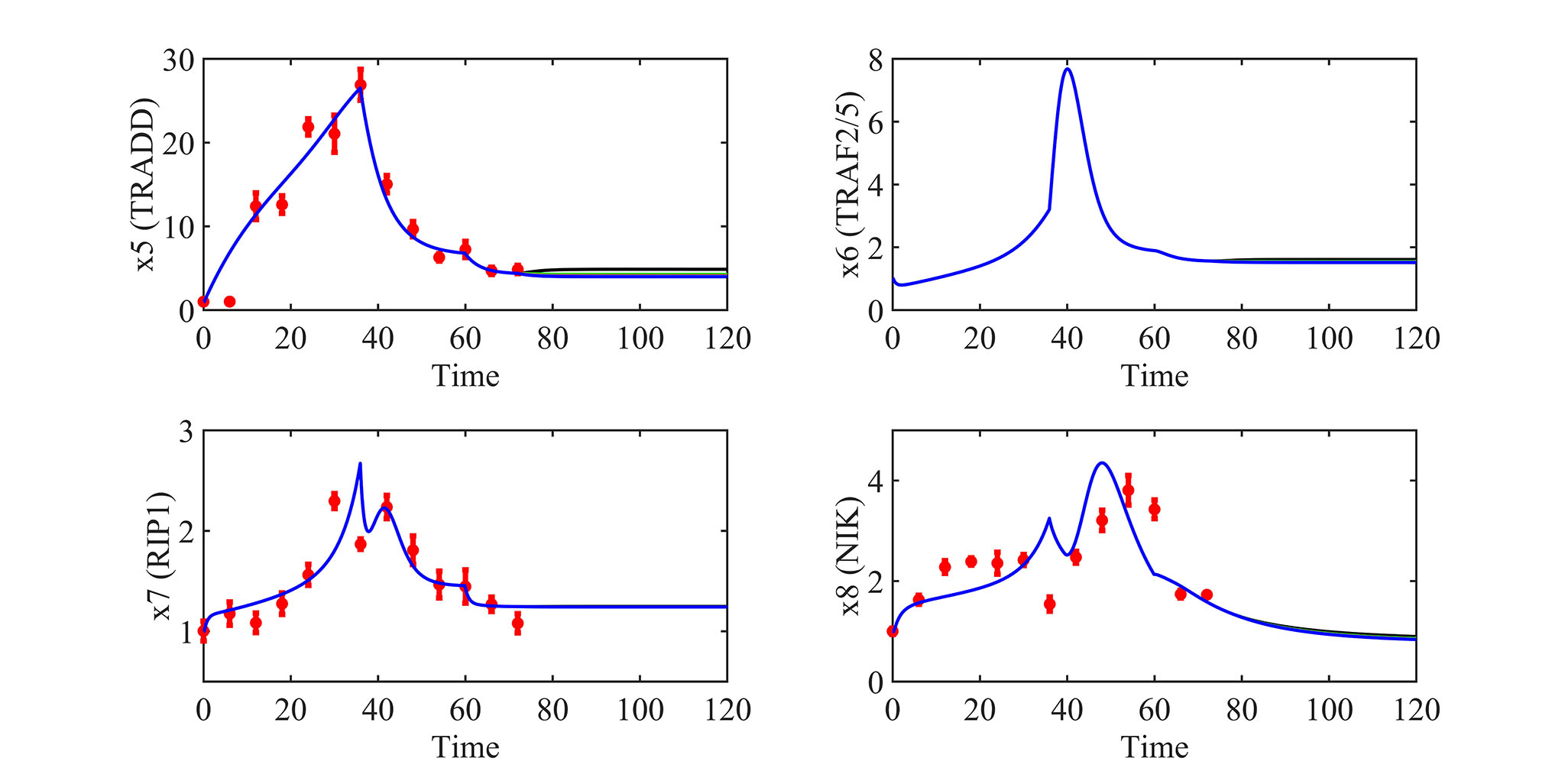

Supplement: Supplementary file 4 [file DataSheet2.zip › Supplementary material_image2/Parameter_d4(小)/2.jpg]

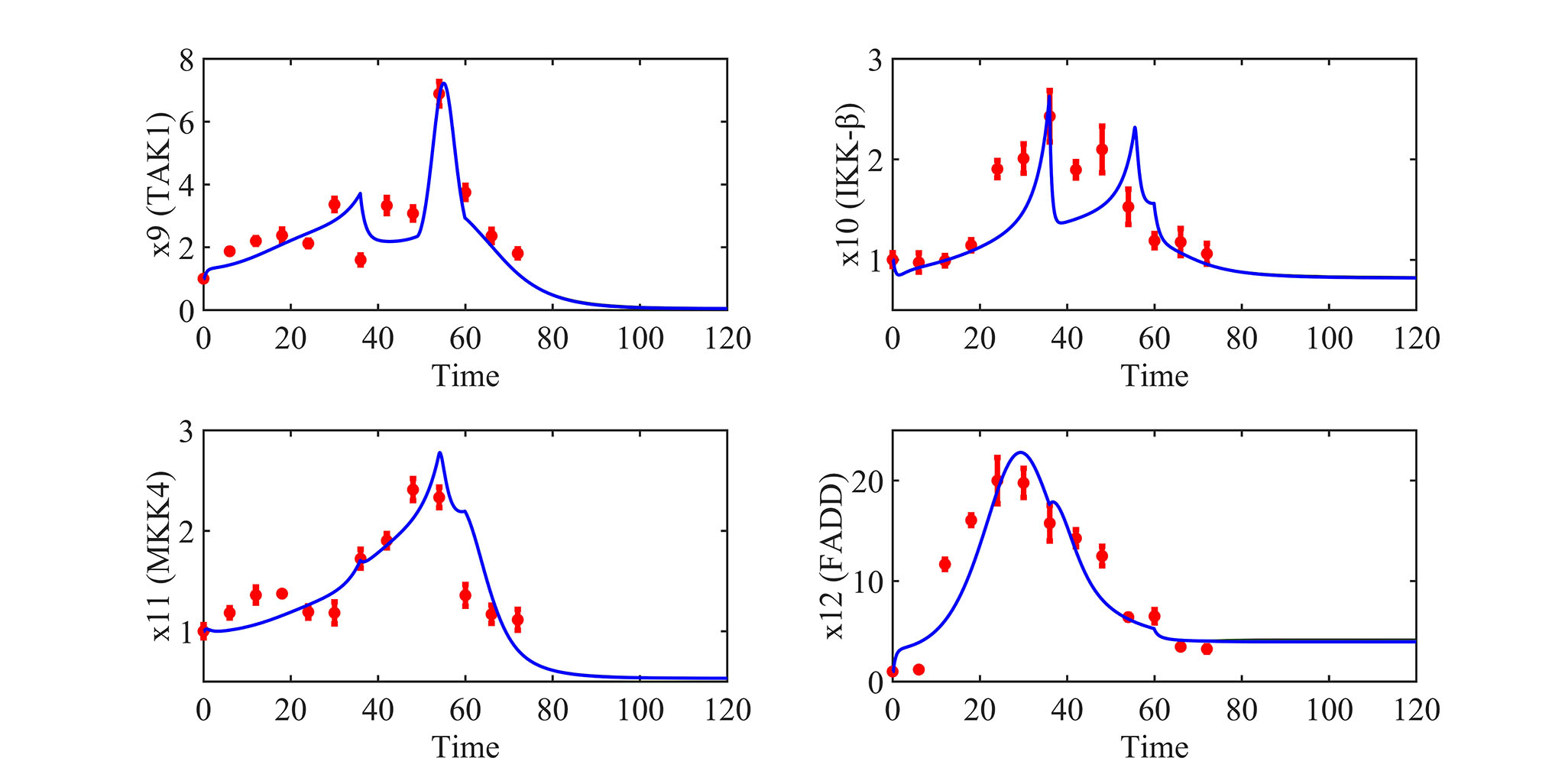

Supplement: Supplementary file 4 [file DataSheet2.zip › Supplementary material_image2/Parameter_d4(小)/3.jpg]

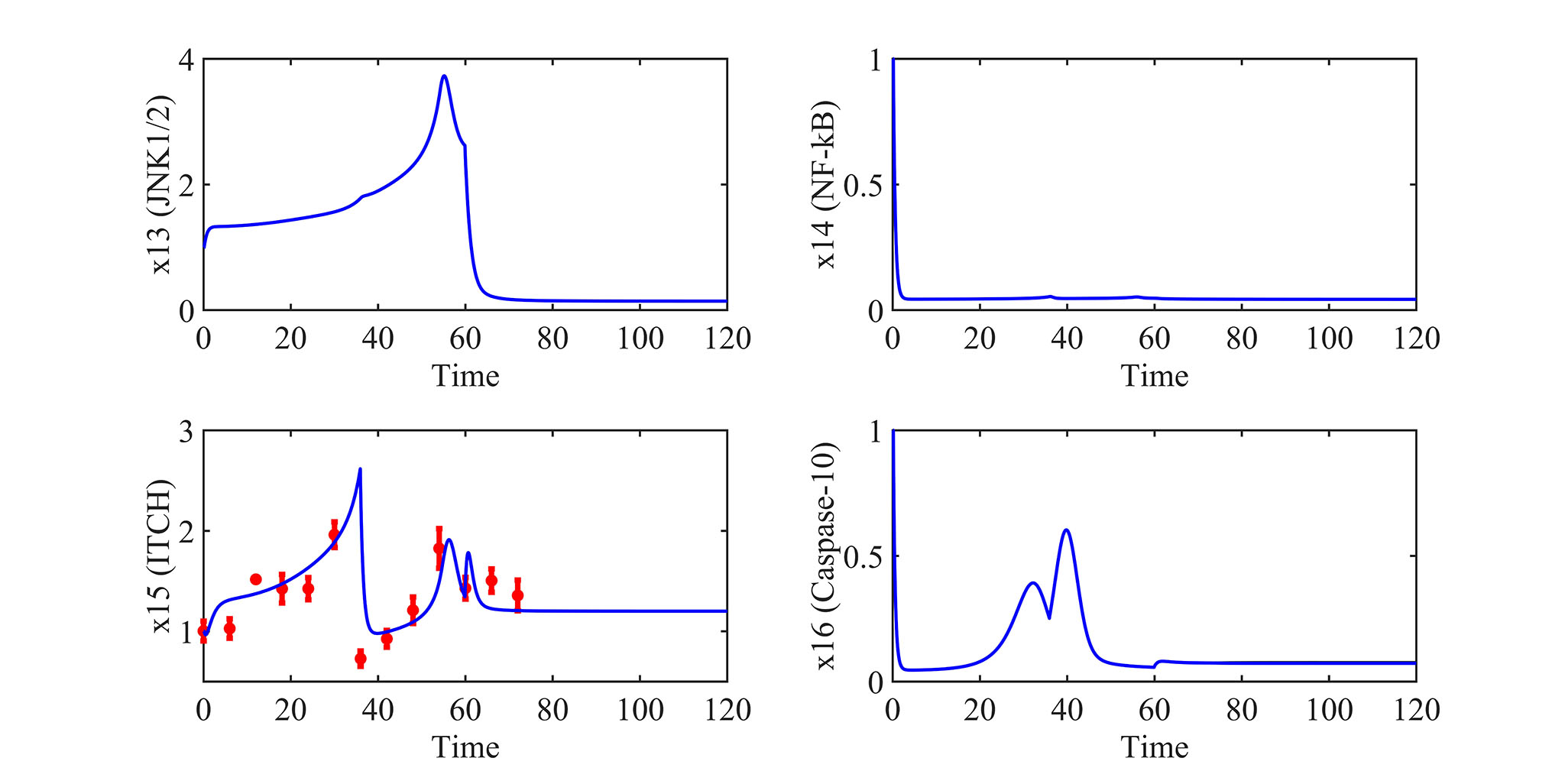

Supplement: Supplementary file 4 [file DataSheet2.zip › Supplementary material_image2/Parameter_d4(小)/4.jpg]

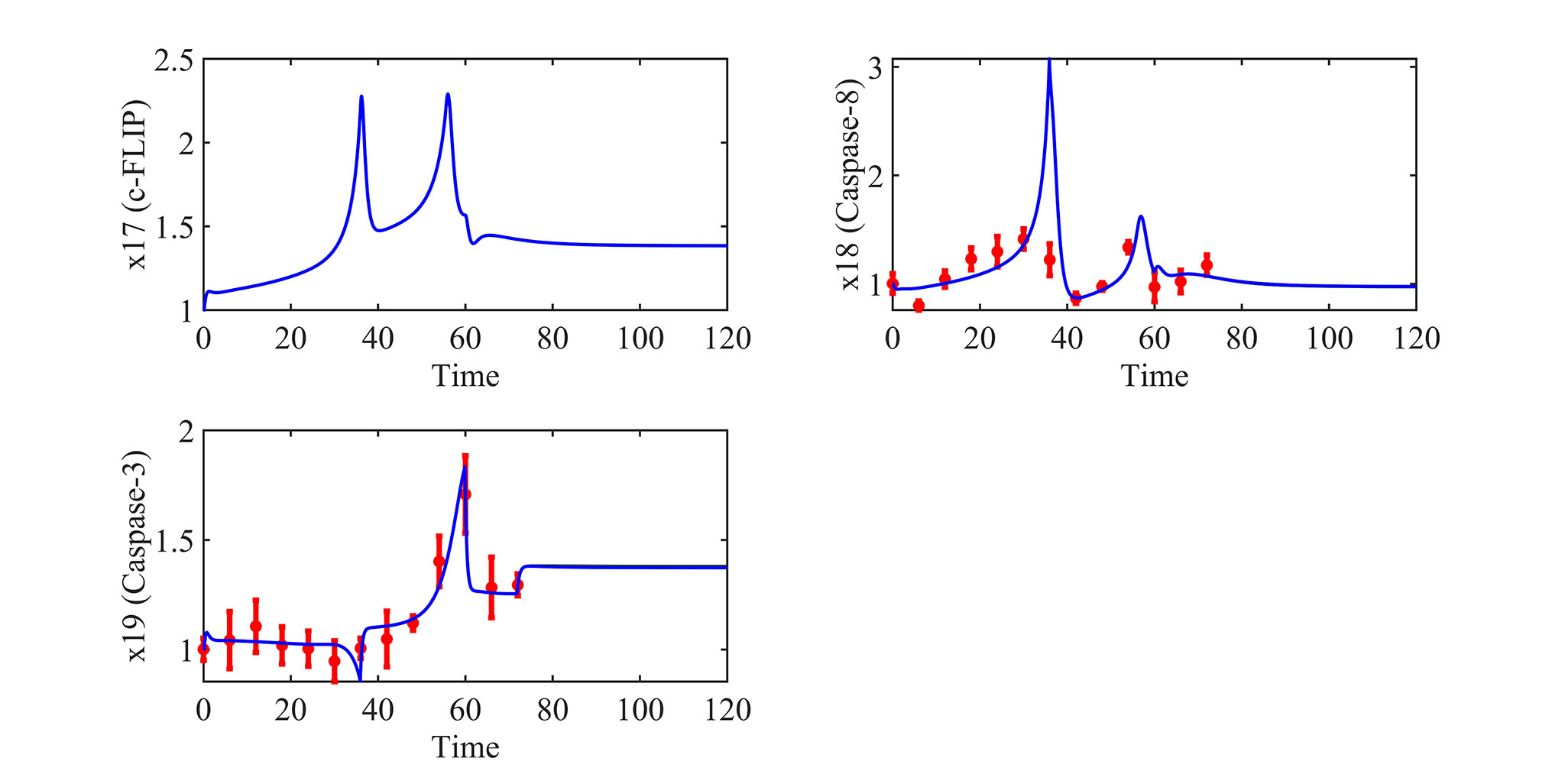

Supplement: Supplementary file 4 [file DataSheet2.zip › Supplementary material_image2/Parameter_d4(小)/5.jpg]

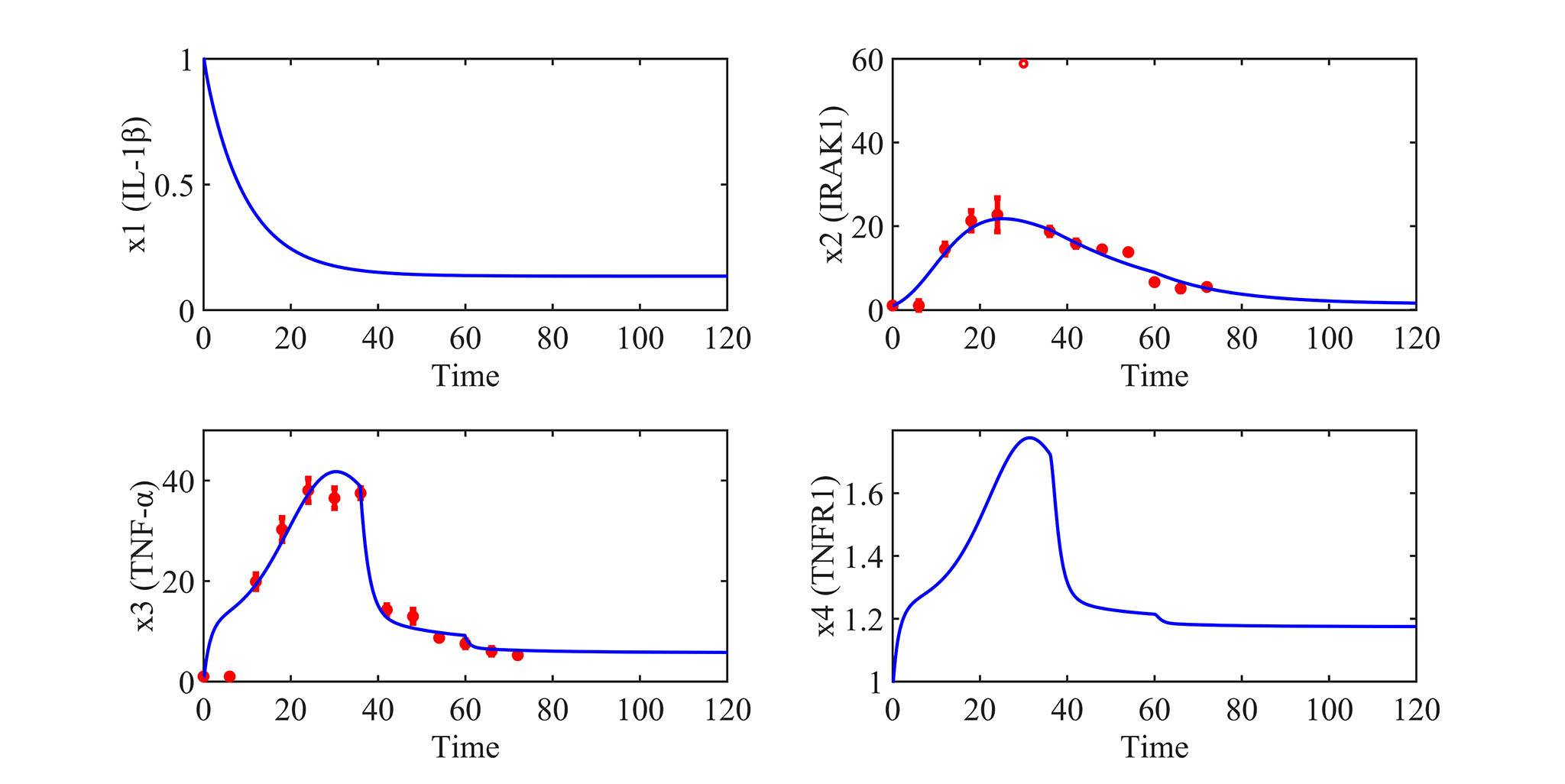

Supplement: Supplementary file 4 [file DataSheet2.zip › Supplementary material_image2/Parameter_d5(大)/1.jpg]

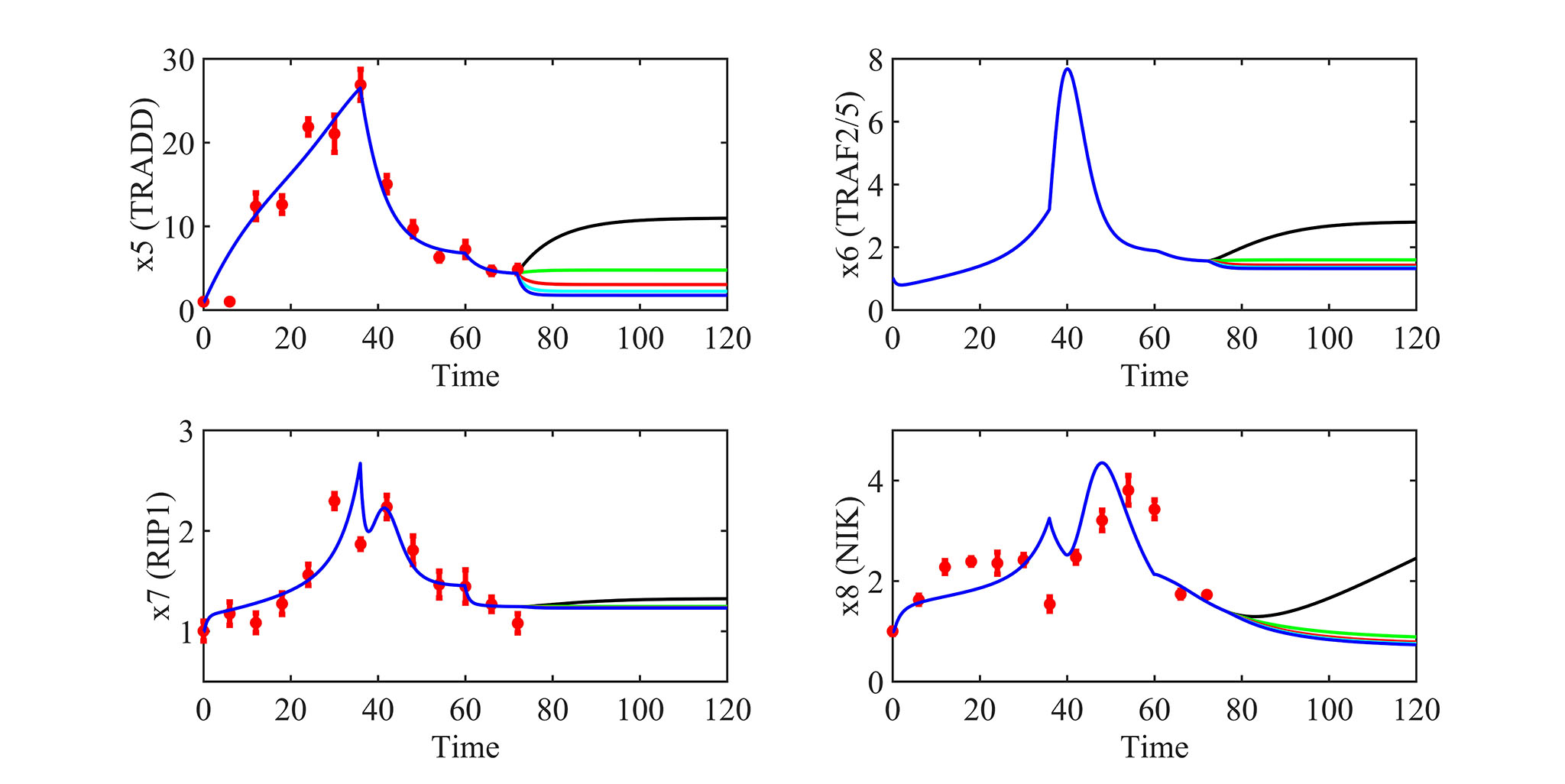

Supplement: Supplementary file 4 [file DataSheet2.zip › Supplementary material_image2/Parameter_d5(大)/2.jpg]

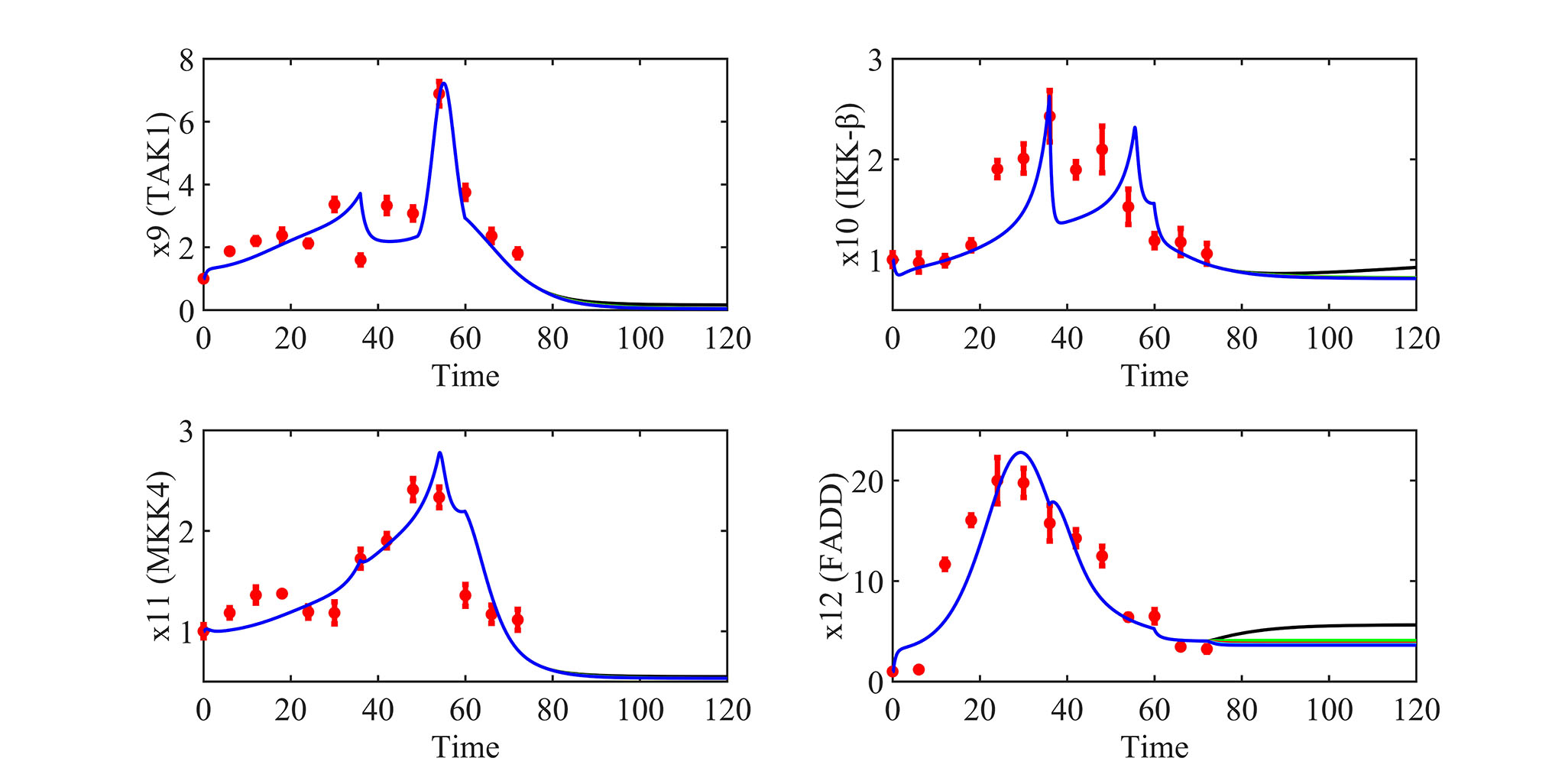

Supplement: Supplementary file 4 [file DataSheet2.zip › Supplementary material_image2/Parameter_d5(大)/3.jpg]

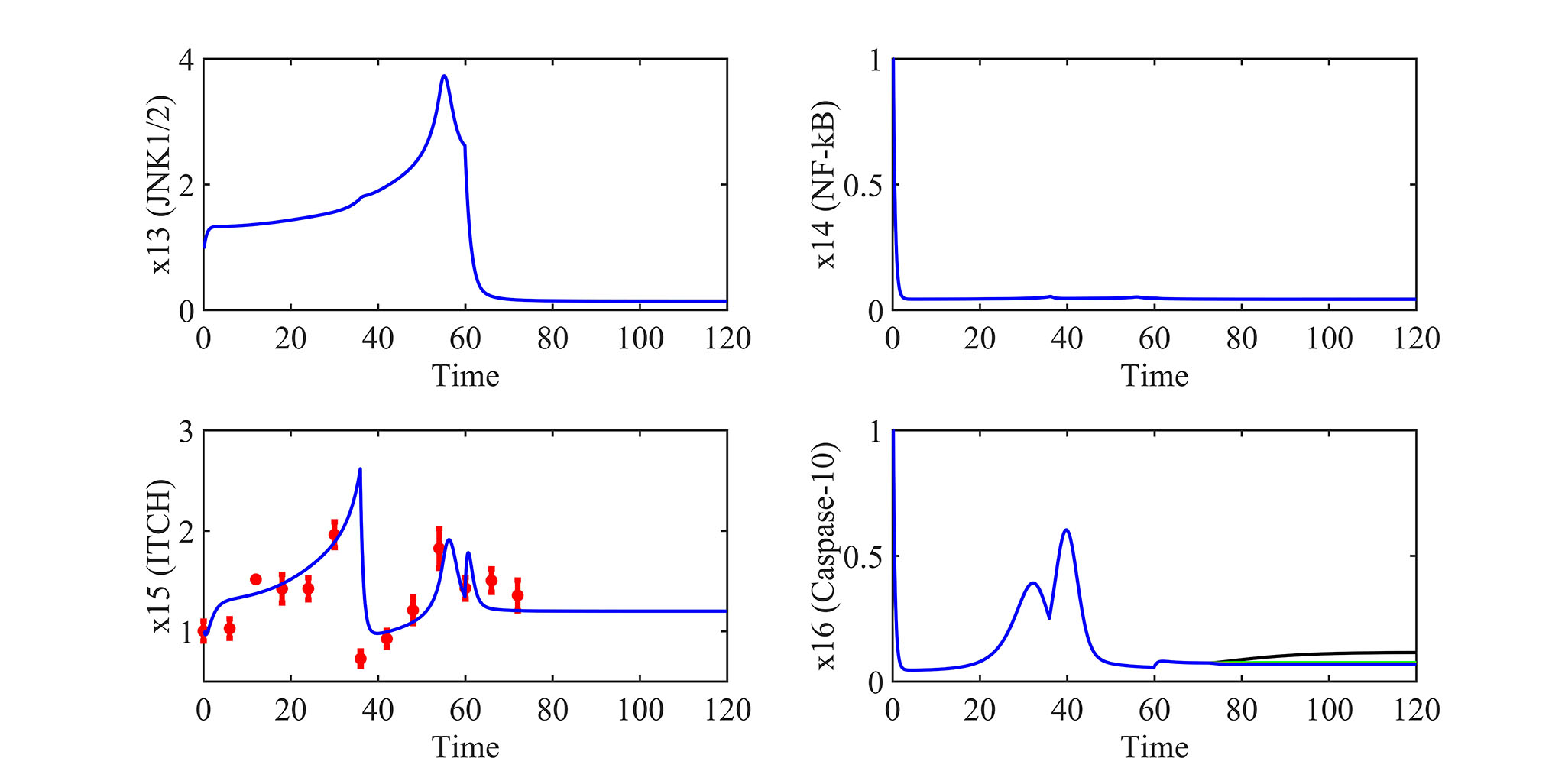

Supplement: Supplementary file 4 [file DataSheet2.zip › Supplementary material_image2/Parameter_d5(大)/4.jpg]

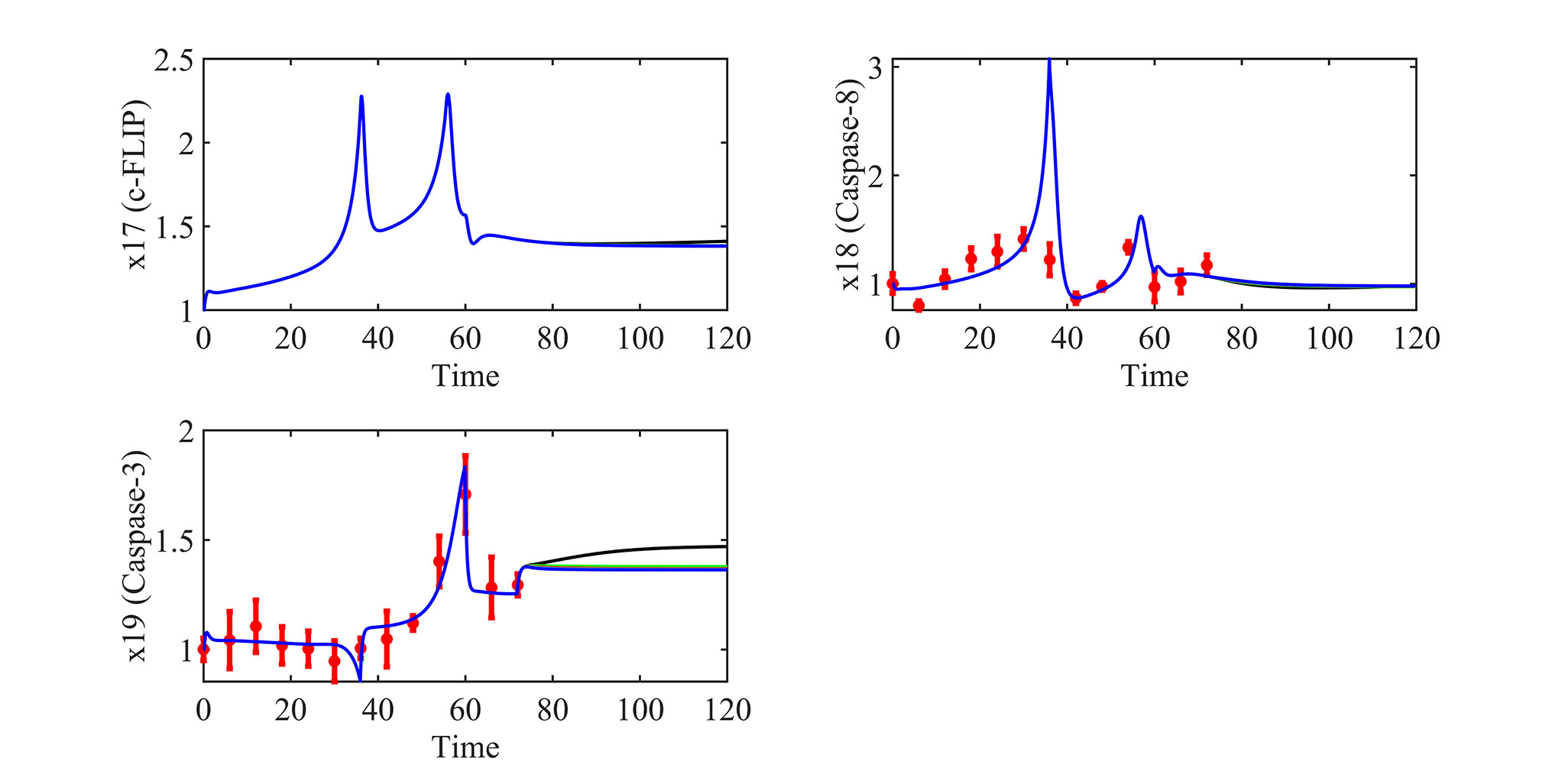

Supplement: Supplementary file 4 [file DataSheet2.zip › Supplementary material_image2/Parameter_d5(大)/5.jpg]

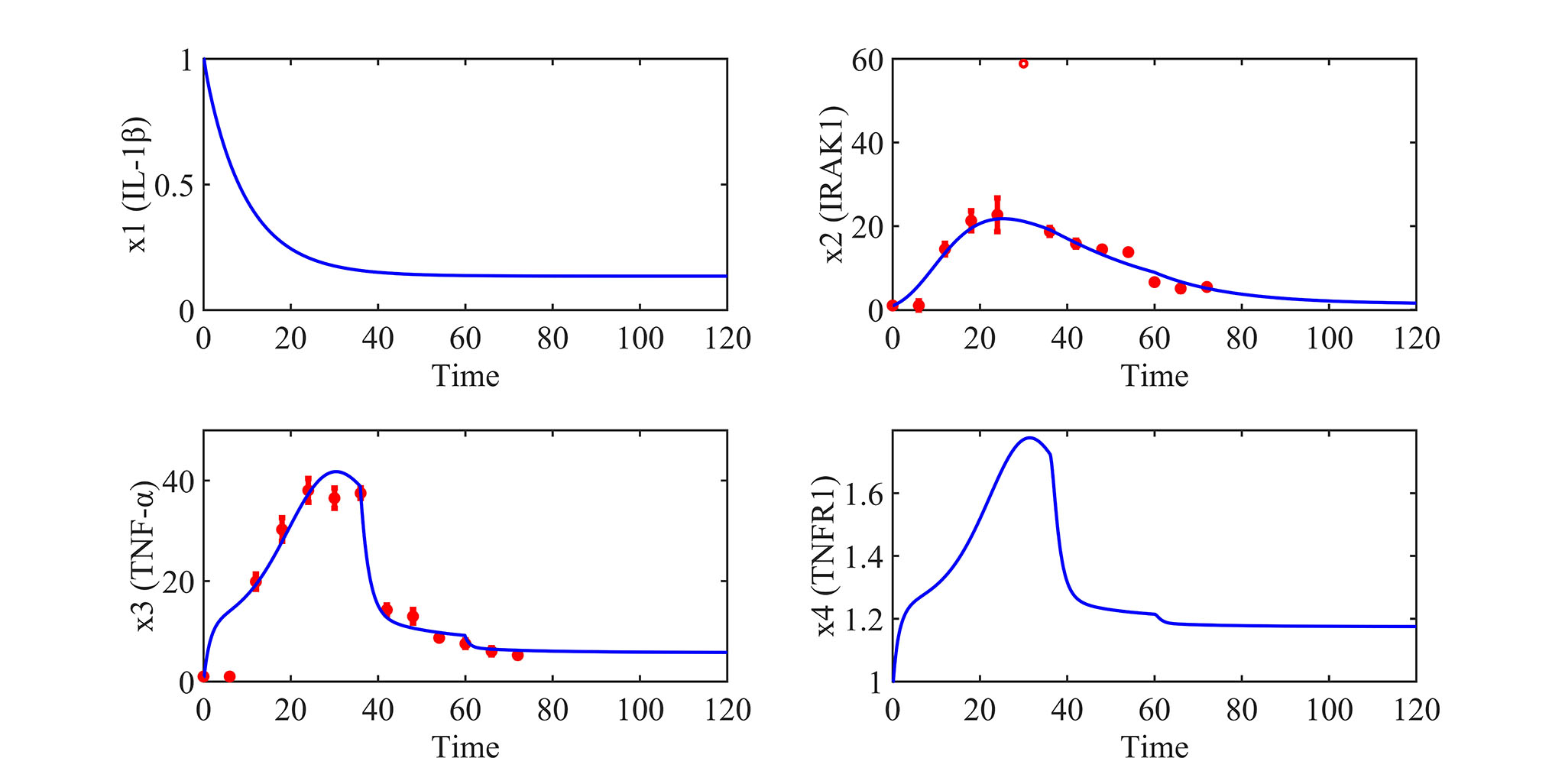

Supplement: Supplementary file 4 [file DataSheet2.zip › Supplementary material_image2/Parameter_d6(小)/1.jpg]

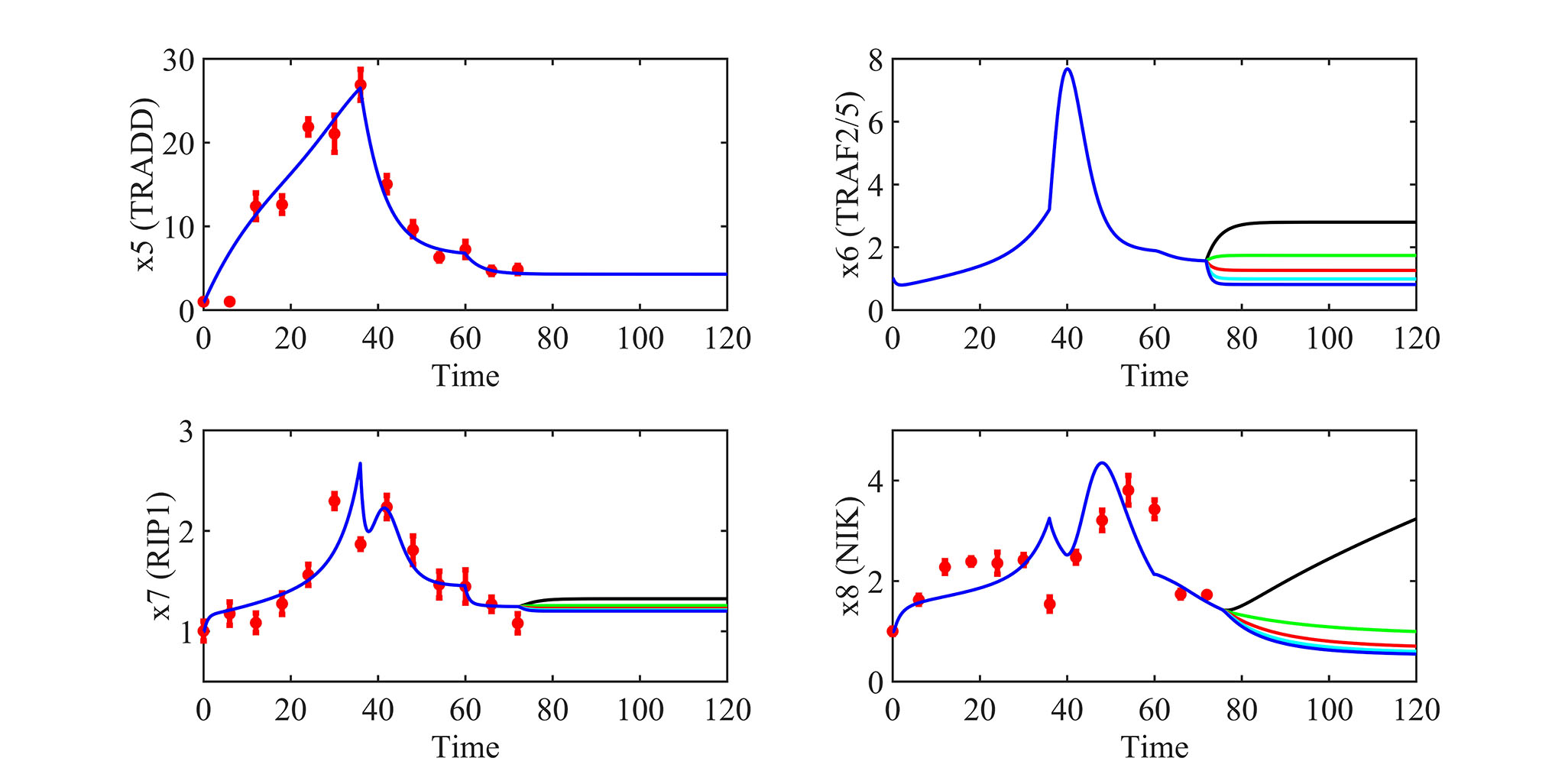

Supplement: Supplementary file 4 [file DataSheet2.zip › Supplementary material_image2/Parameter_d6(小)/2.jpg]

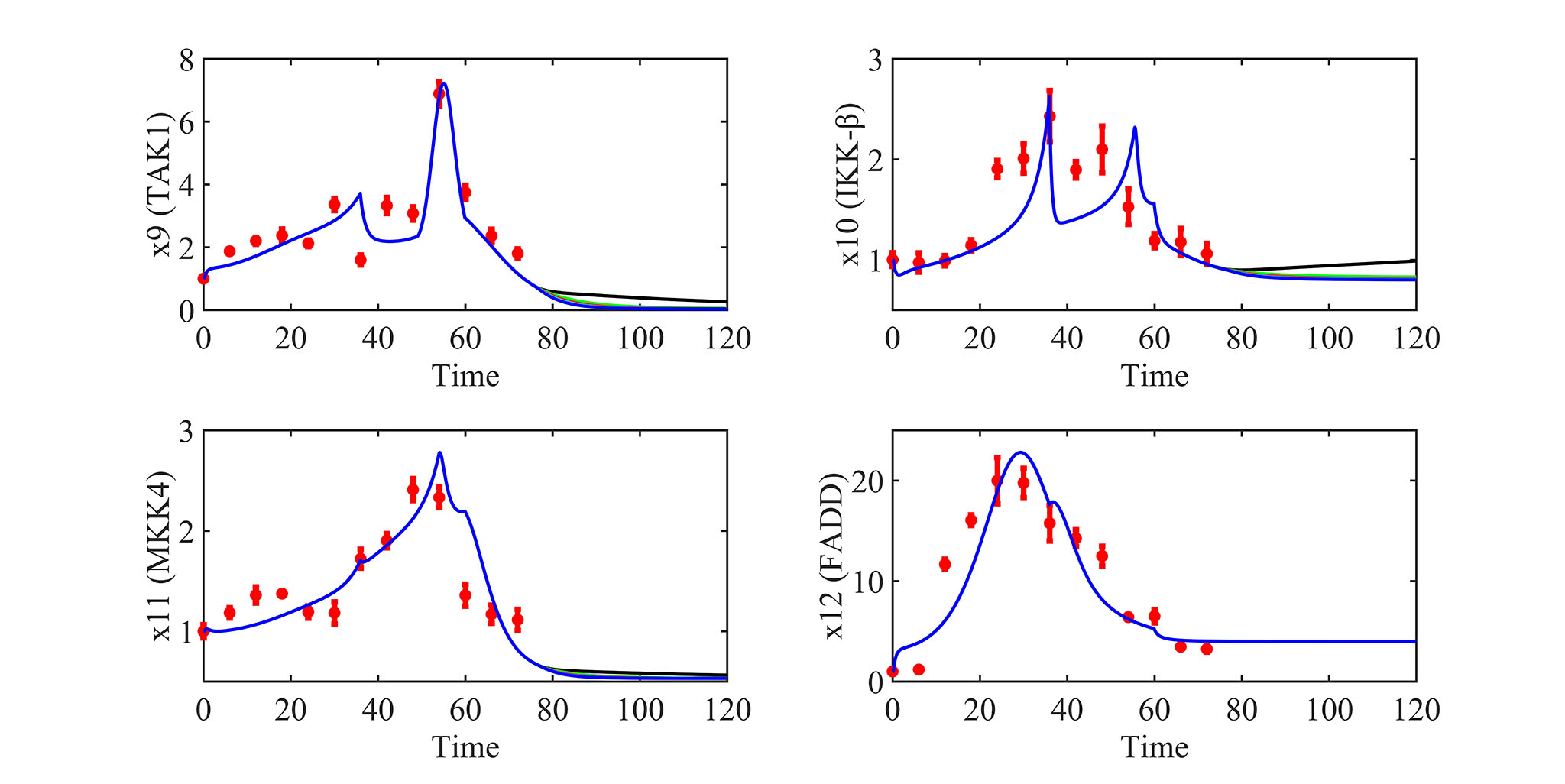

Supplement: Supplementary file 4 [file DataSheet2.zip › Supplementary material_image2/Parameter_d6(小)/3.jpg]

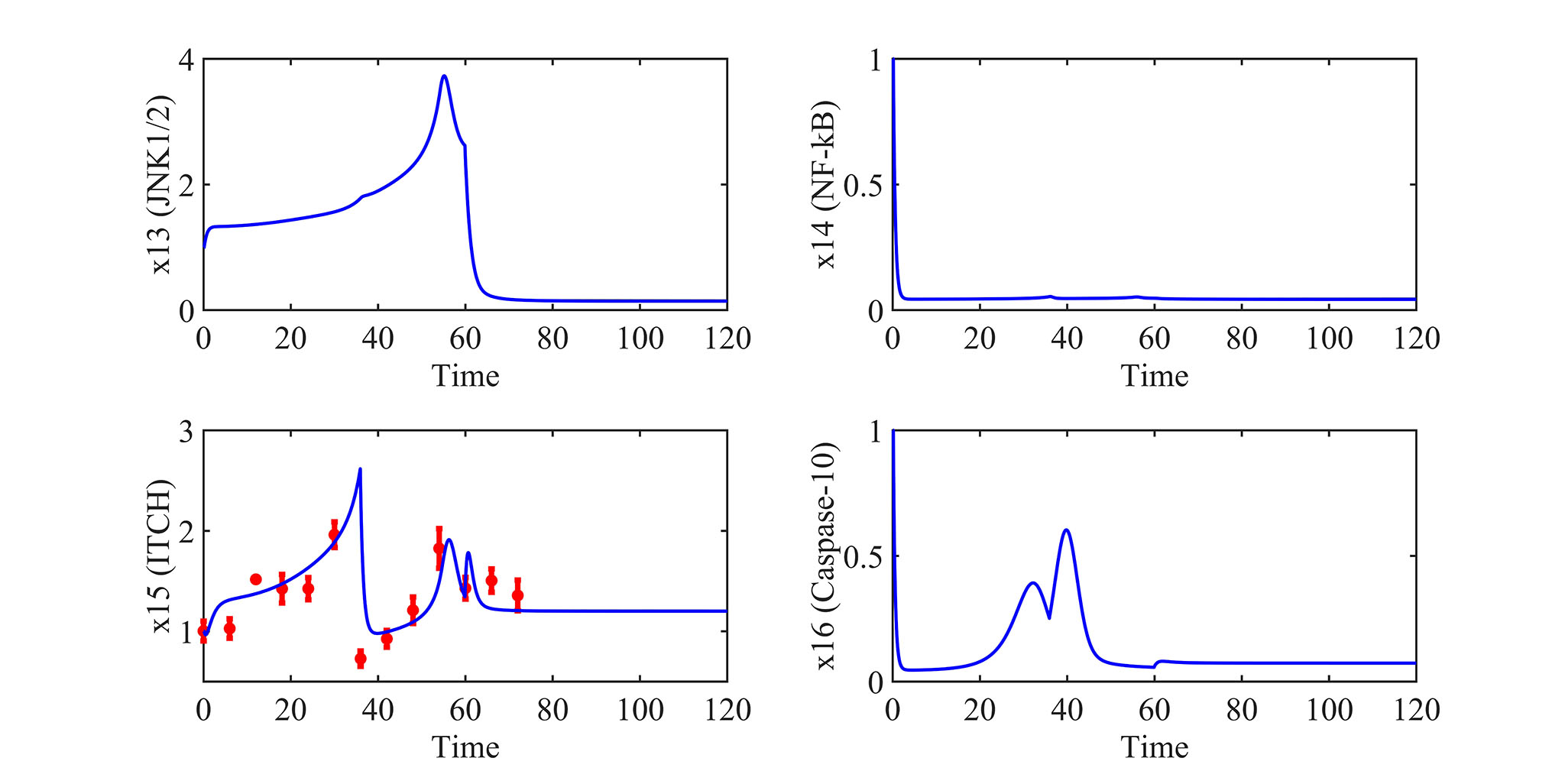

Supplement: Supplementary file 4 [file DataSheet2.zip › Supplementary material_image2/Parameter_d6(小)/4.jpg]

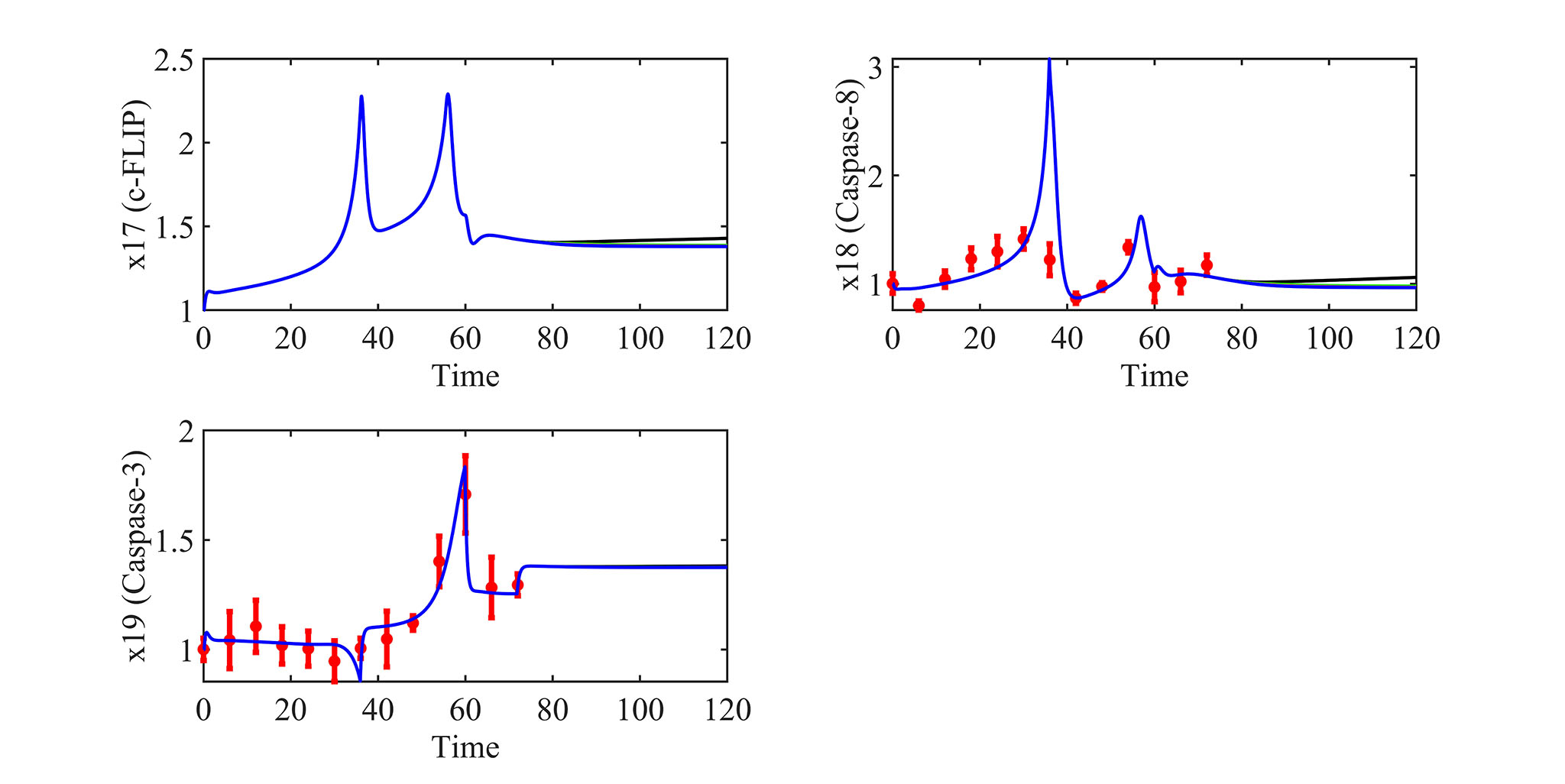

Supplement: Supplementary file 4 [file DataSheet2.zip › Supplementary material_image2/Parameter_d6(小)/5.jpg]

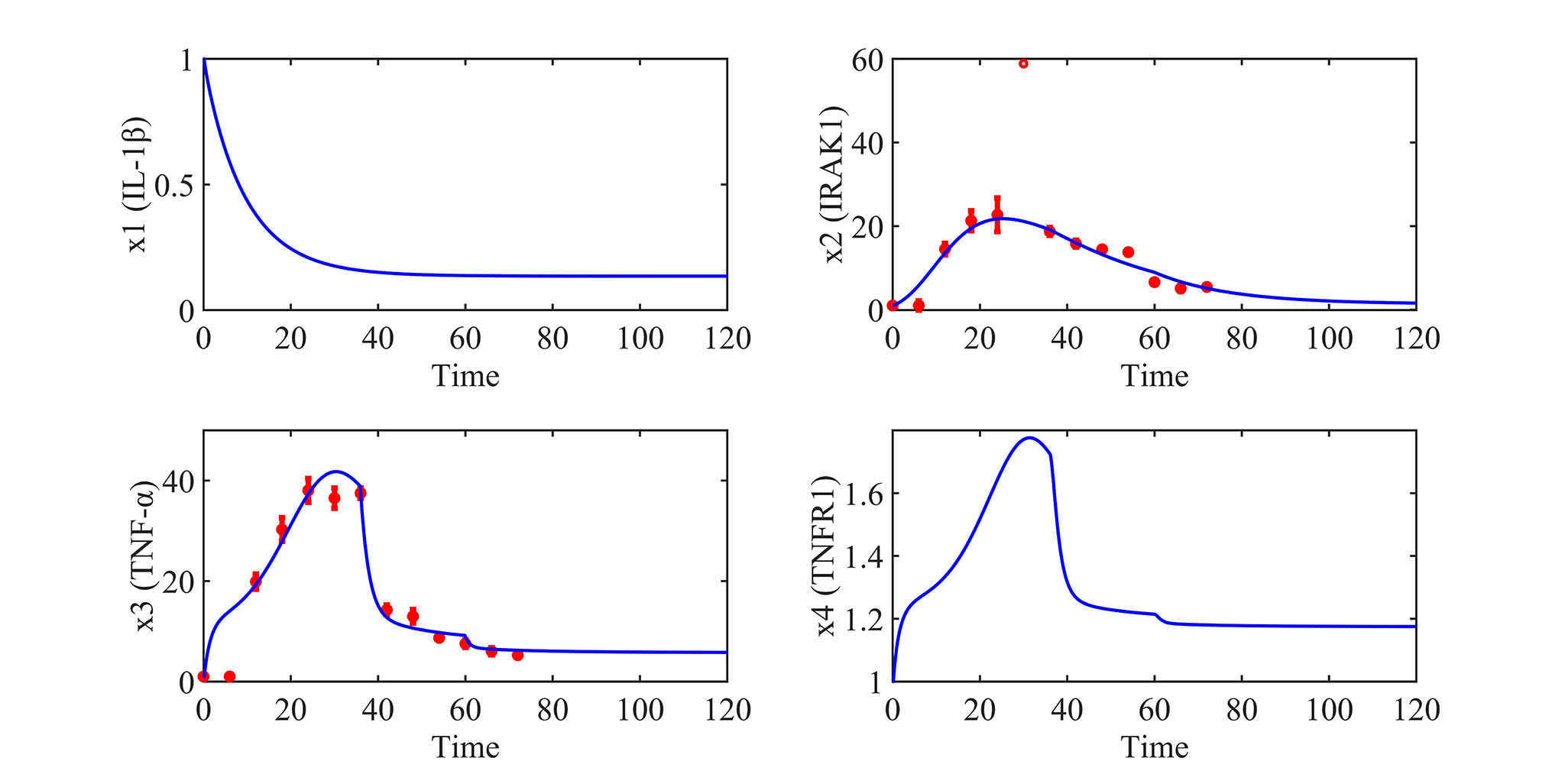

Supplement: Supplementary file 4 [file DataSheet2.zip › Supplementary material_image2/Parameter_d7(小)/1.jpg]

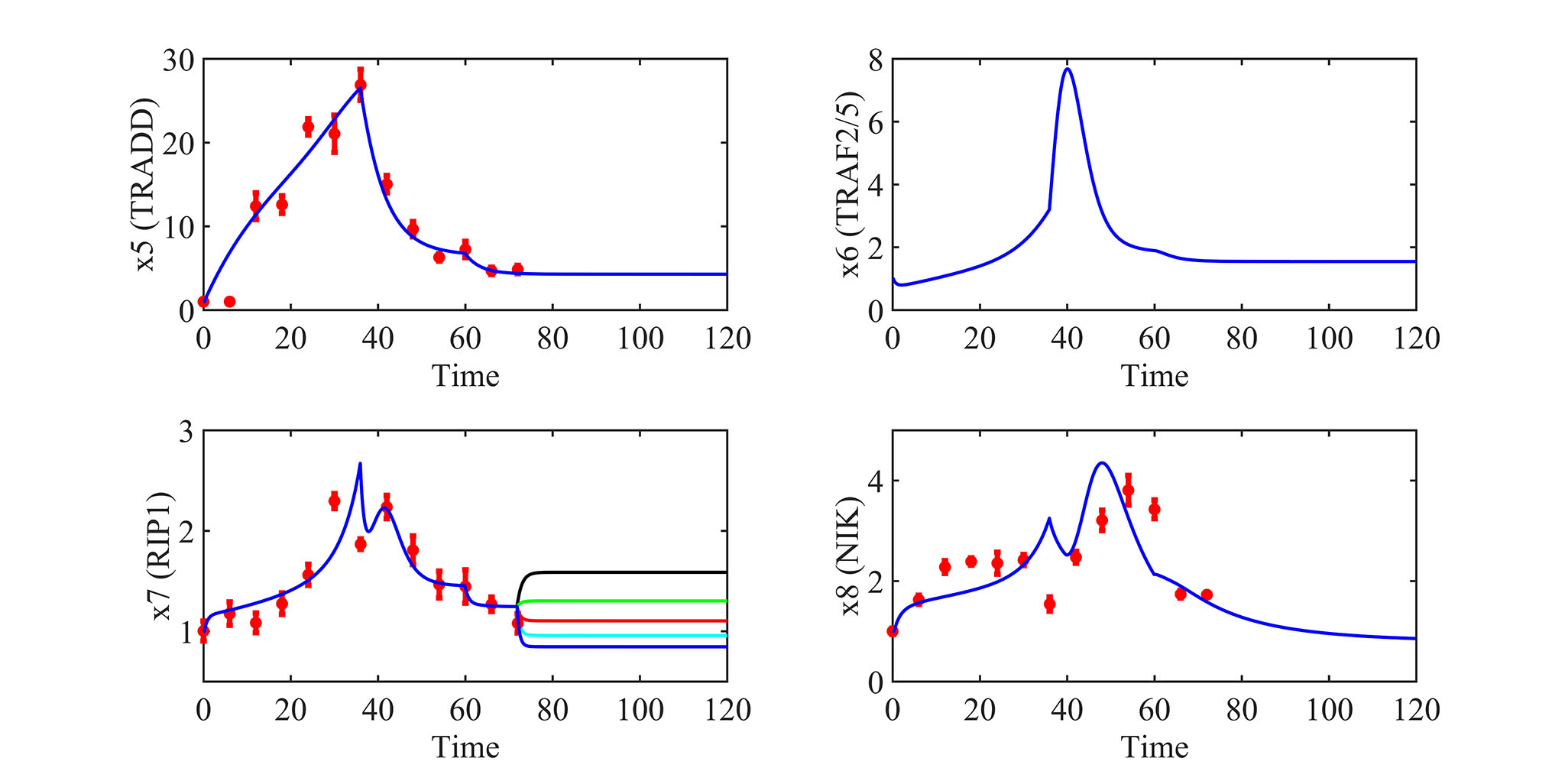

Supplement: Supplementary file 4 [file DataSheet2.zip › Supplementary material_image2/Parameter_d7(小)/2.jpg]

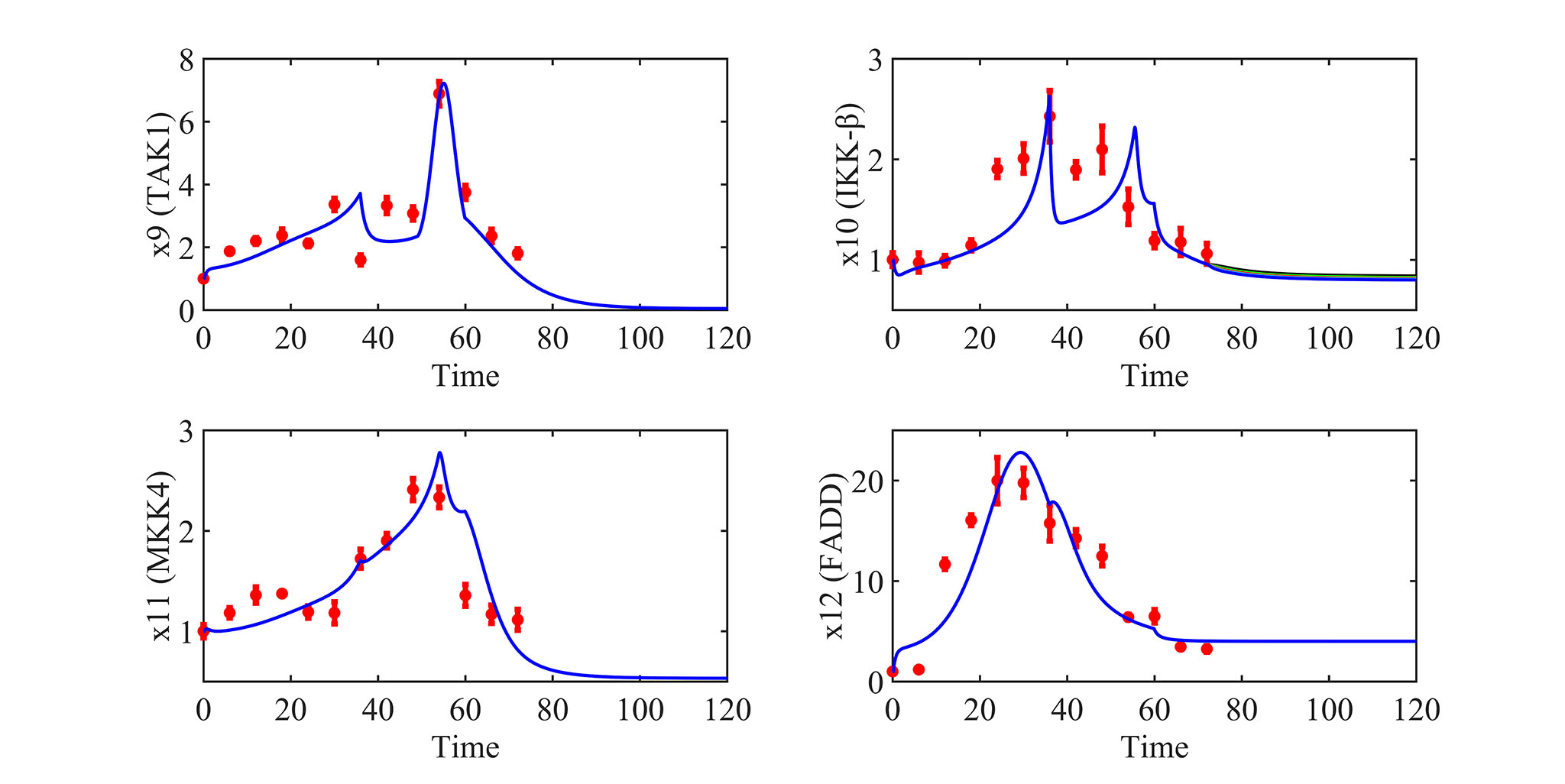

Supplement: Supplementary file 4 [file DataSheet2.zip › Supplementary material_image2/Parameter_d7(小)/3.jpg]

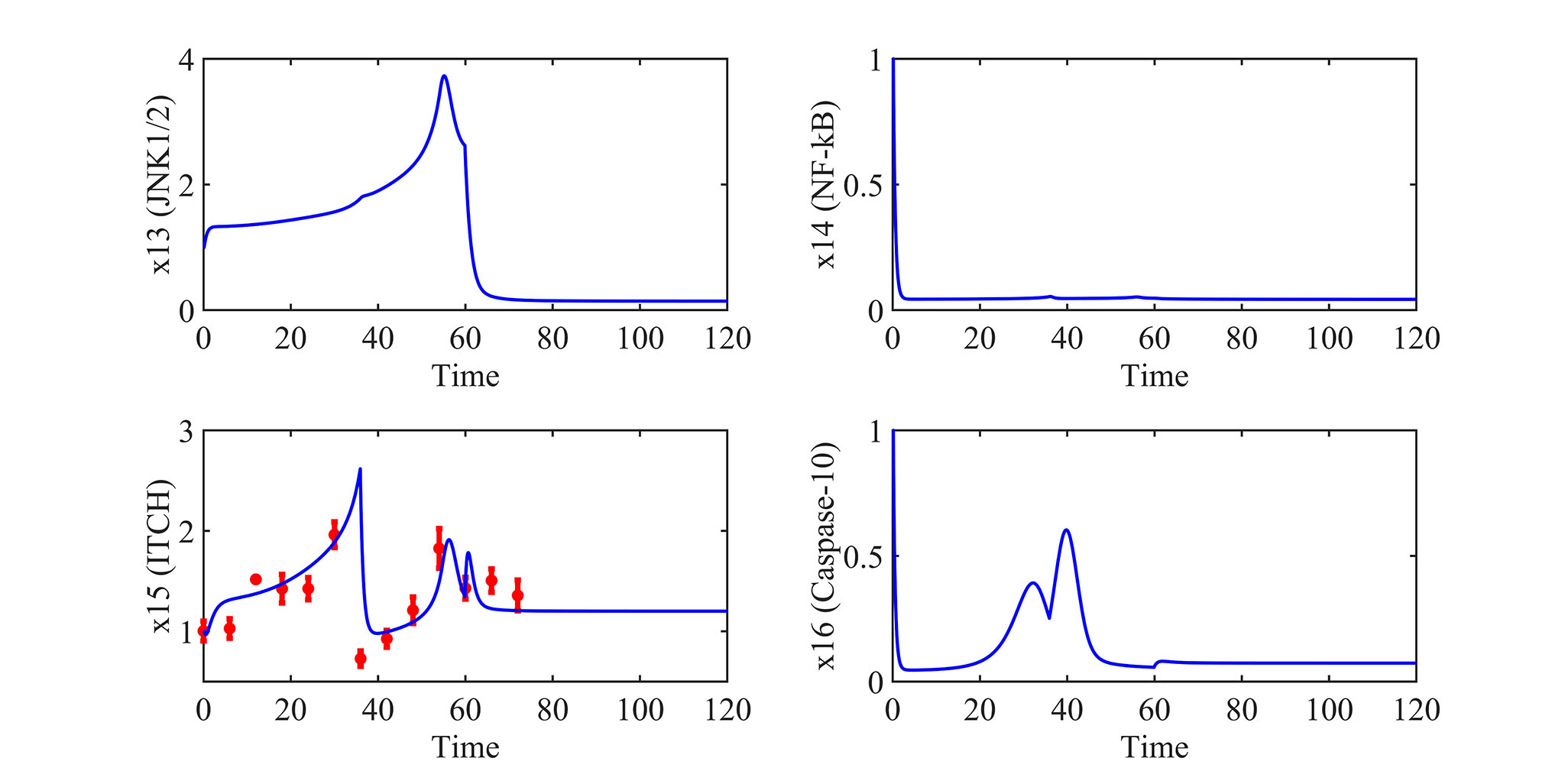

Supplement: Supplementary file 4 [file DataSheet2.zip › Supplementary material_image2/Parameter_d7(小)/4.jpg]

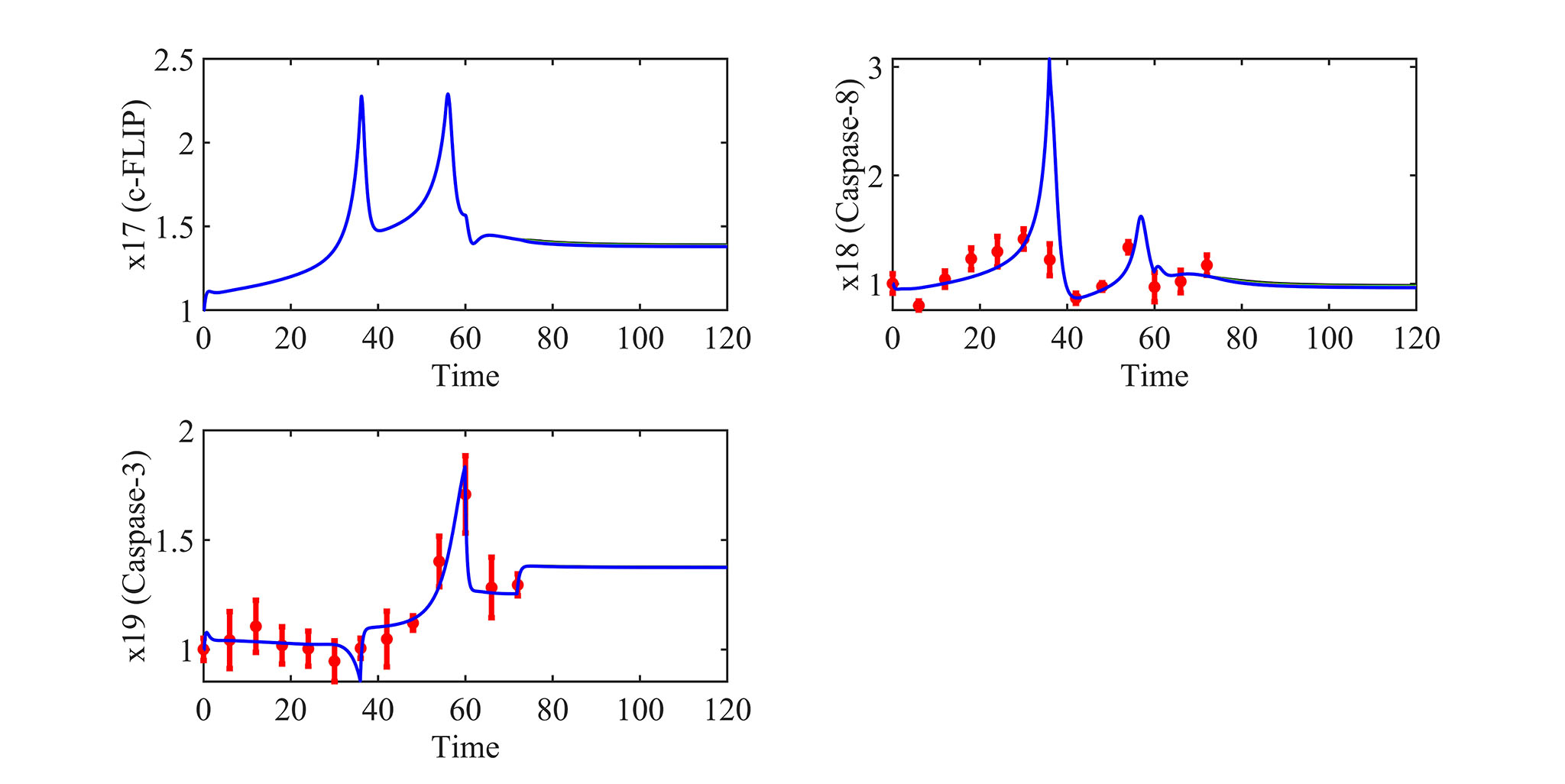

Supplement: Supplementary file 4 [file DataSheet2.zip › Supplementary material_image2/Parameter_d7(小)/5.jpg]

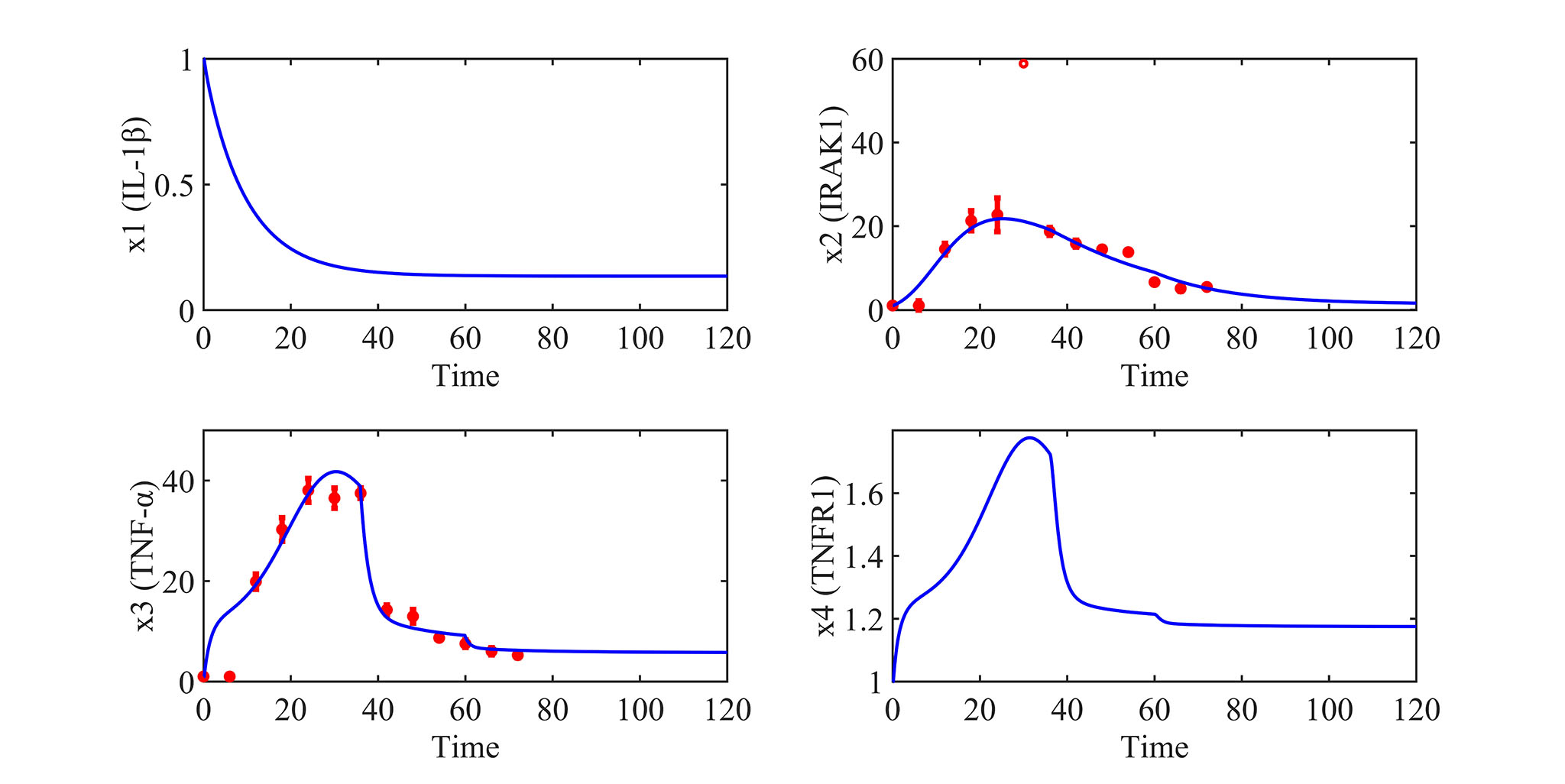

Supplement: Supplementary file 4 [file DataSheet2.zip › Supplementary material_image2/Parameter_d8(小)/1.jpg]

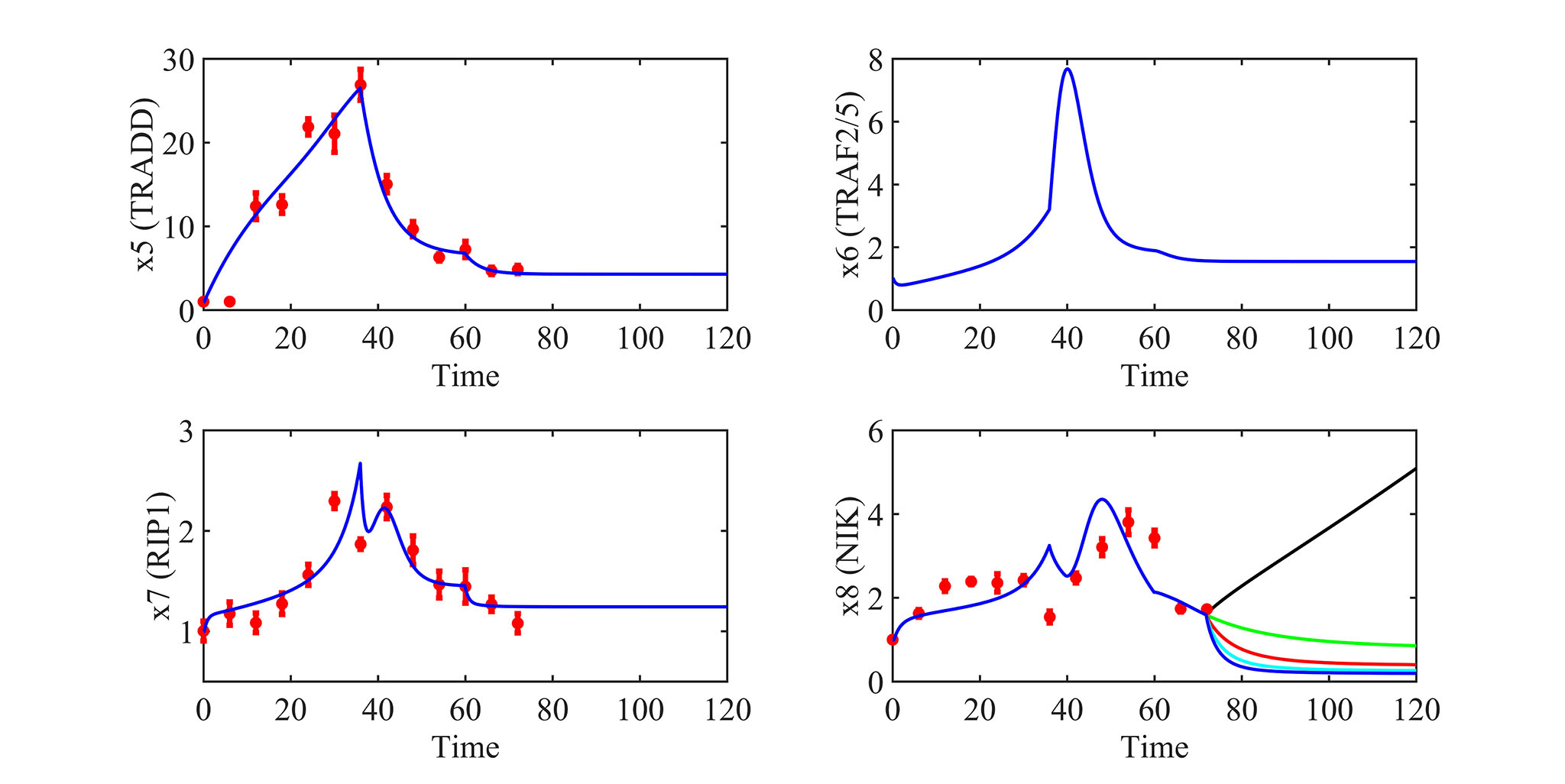

Supplement: Supplementary file 4 [file DataSheet2.zip › Supplementary material_image2/Parameter_d8(小)/2.jpg]

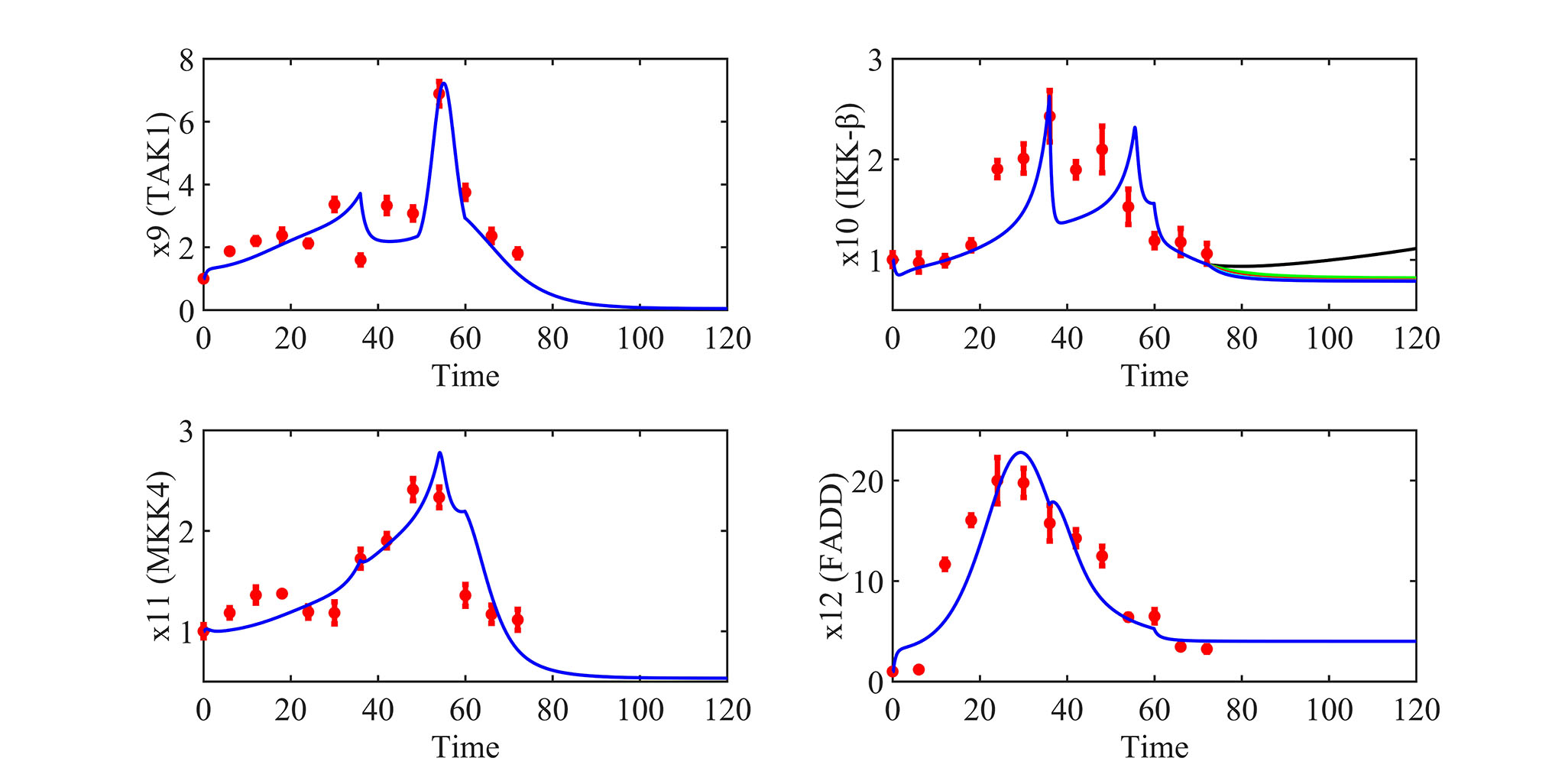

Supplement: Supplementary file 4 [file DataSheet2.zip › Supplementary material_image2/Parameter_d8(小)/3.jpg]

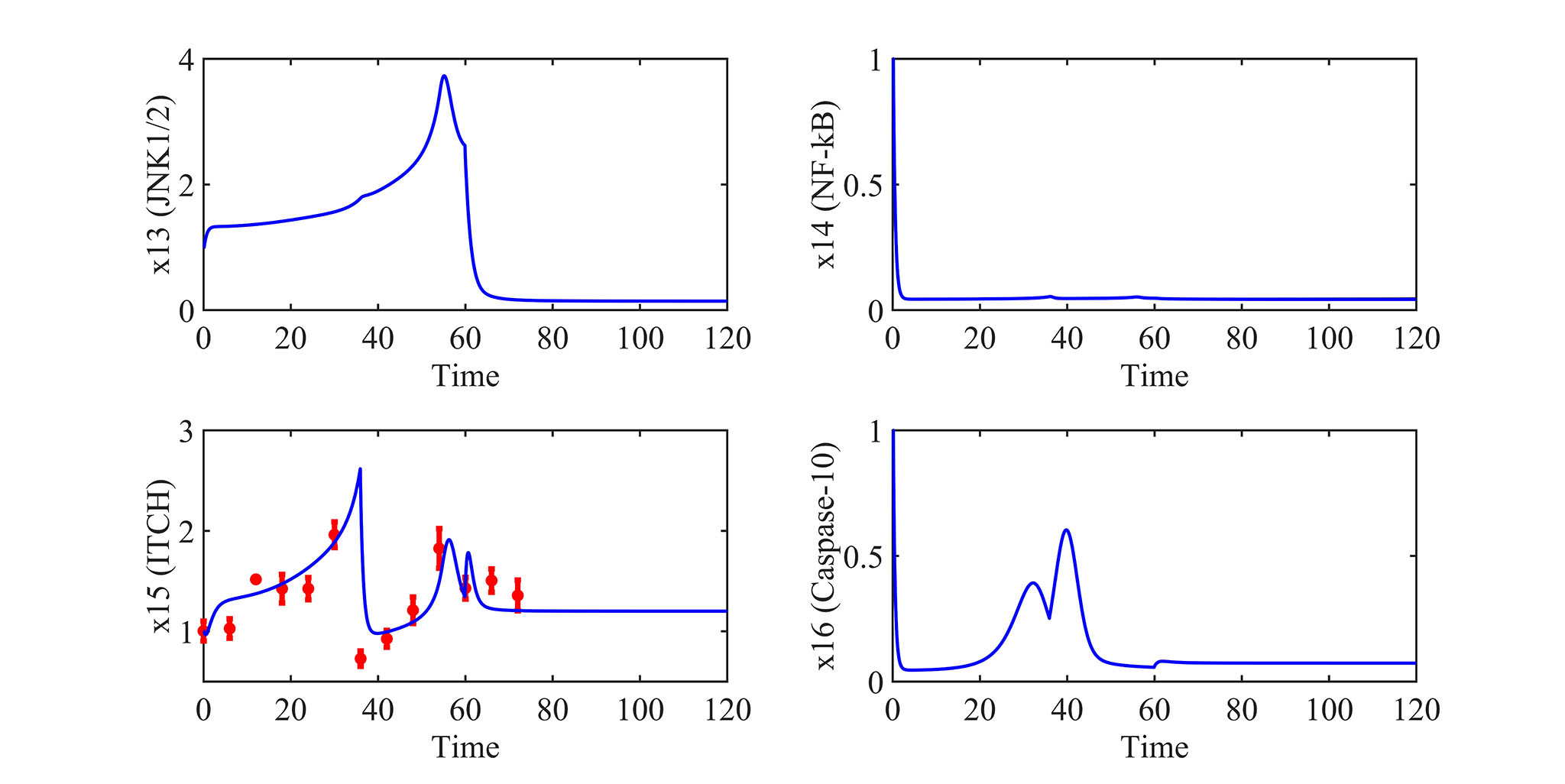

Supplement: Supplementary file 4 [file DataSheet2.zip › Supplementary material_image2/Parameter_d8(小)/4.jpg]

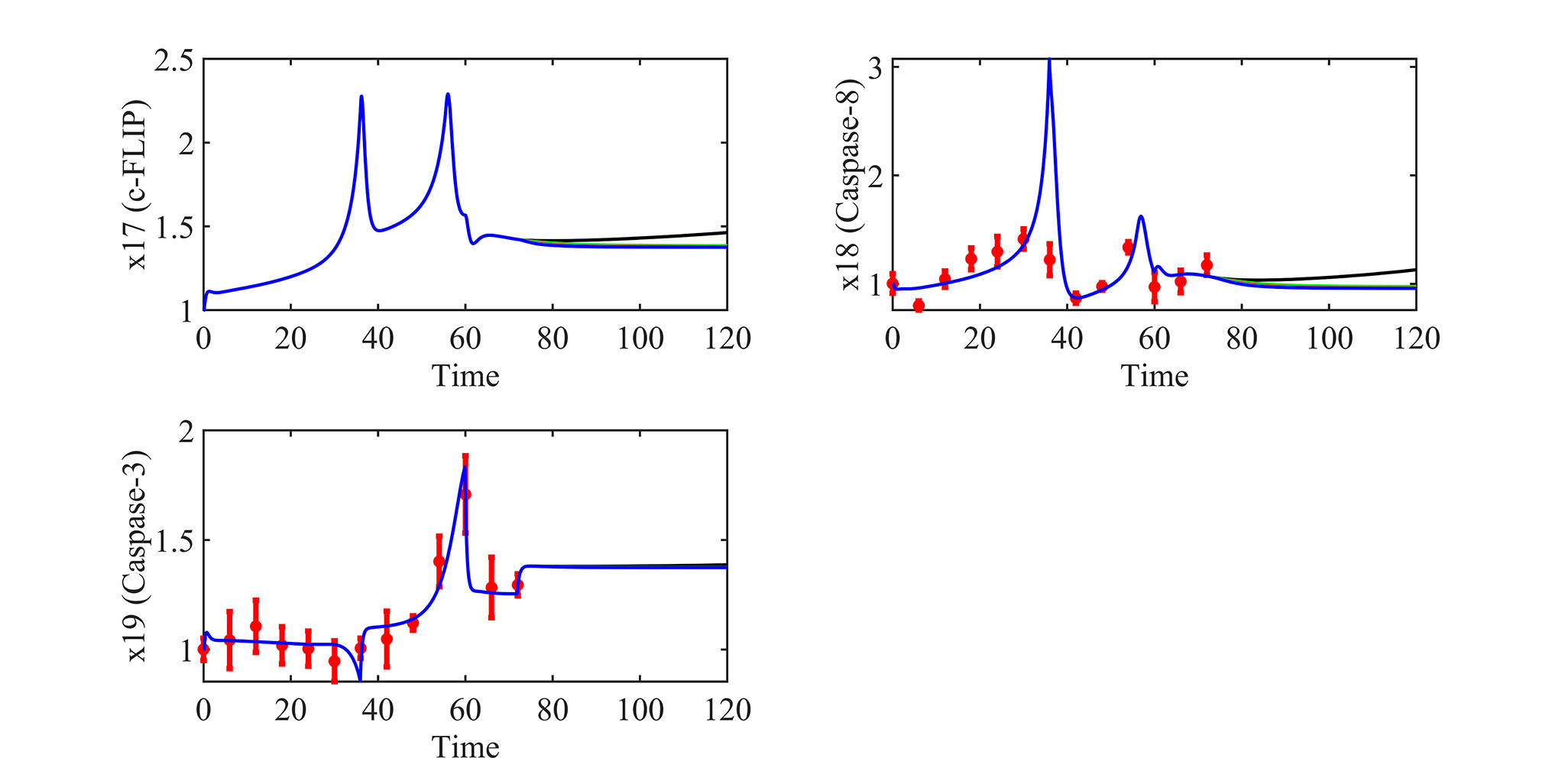

Supplement: Supplementary file 4 [file DataSheet2.zip › Supplementary material_image2/Parameter_d8(小)/5.jpg]

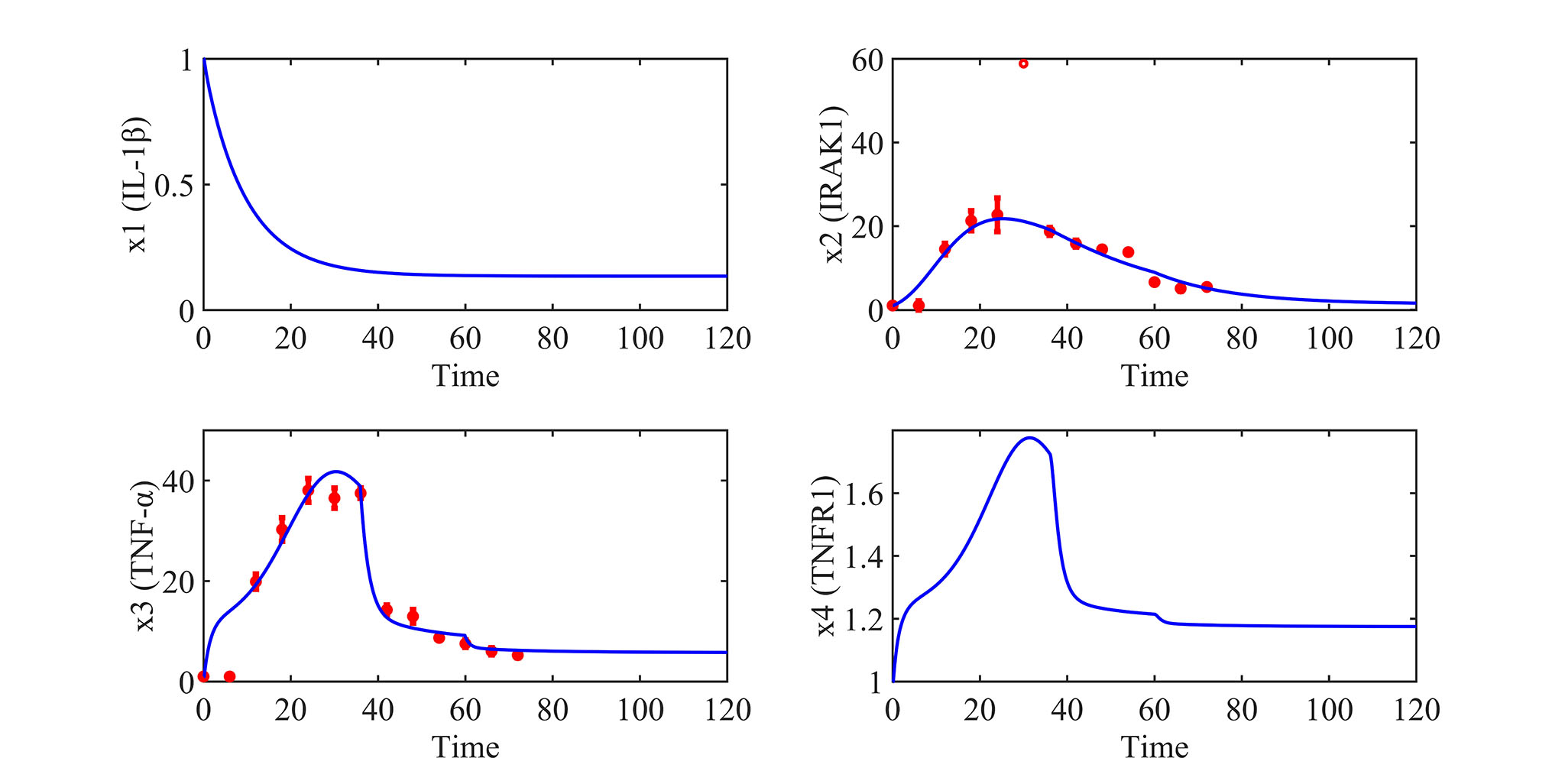

Supplement: Supplementary file 4 [file DataSheet2.zip › Supplementary material_image2/Parameter_d9(小)/1.jpg]

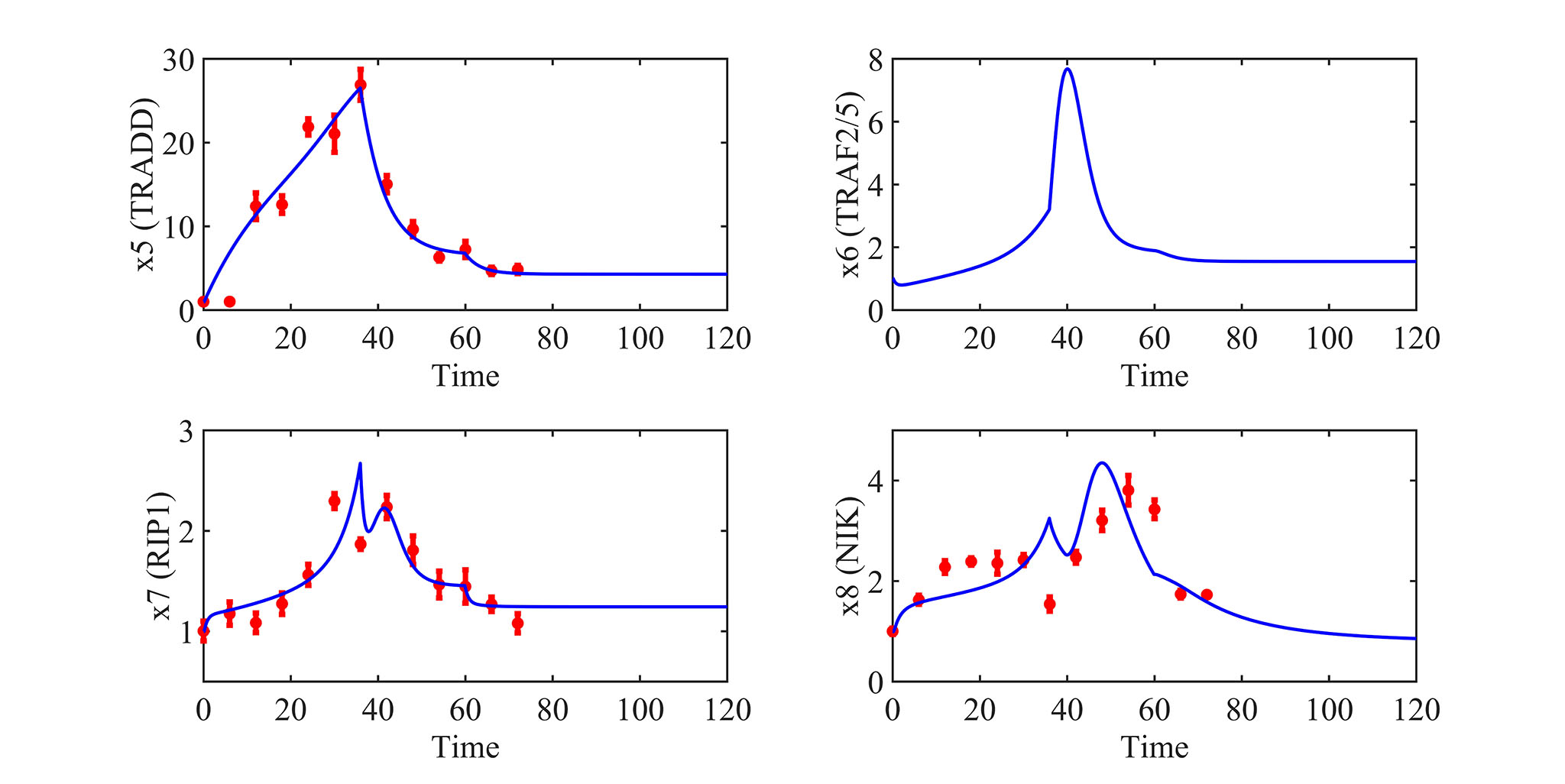

Supplement: Supplementary file 4 [file DataSheet2.zip › Supplementary material_image2/Parameter_d9(小)/2.jpg]

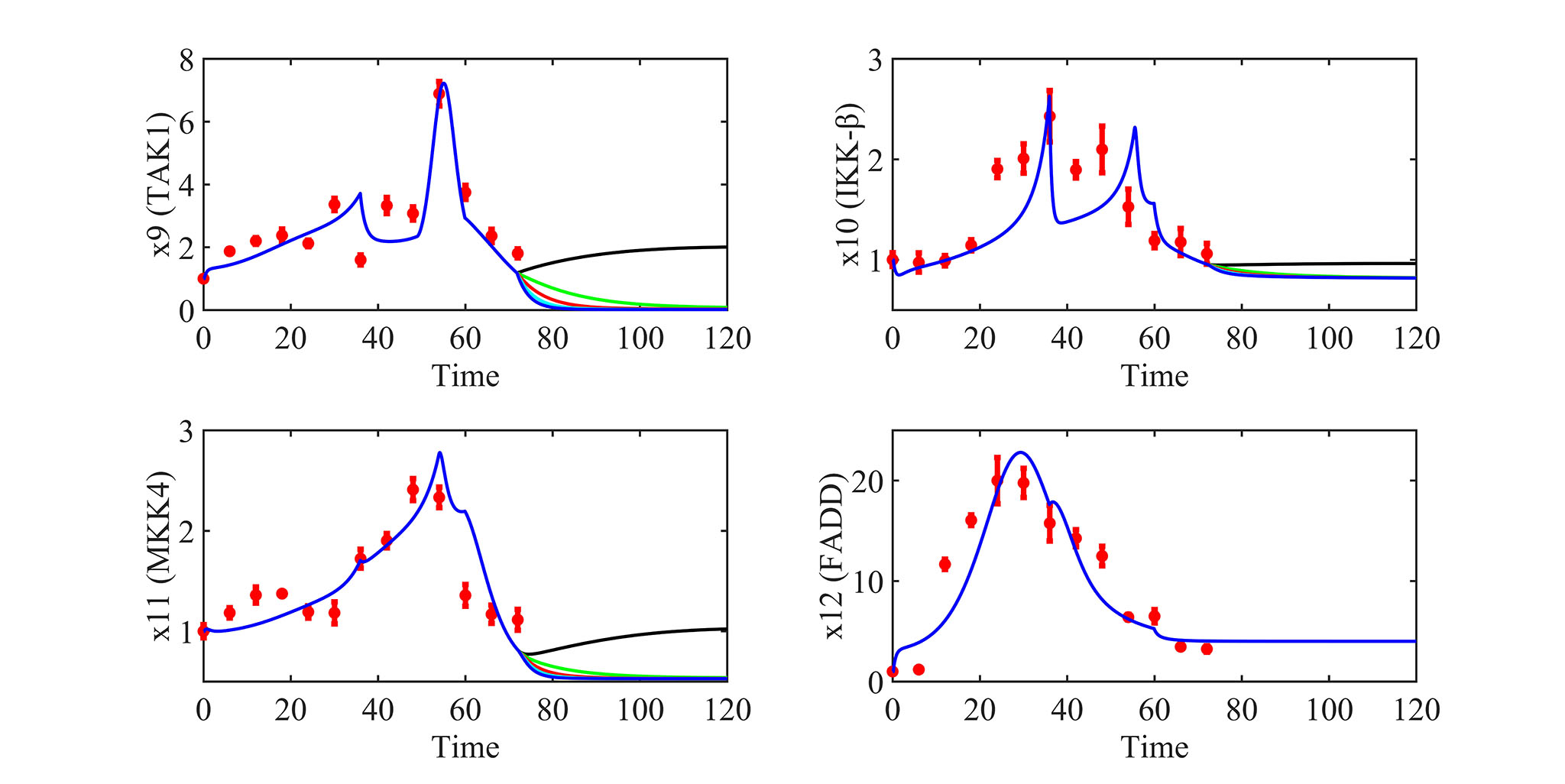

Supplement: Supplementary file 4 [file DataSheet2.zip › Supplementary material_image2/Parameter_d9(小)/3.jpg]

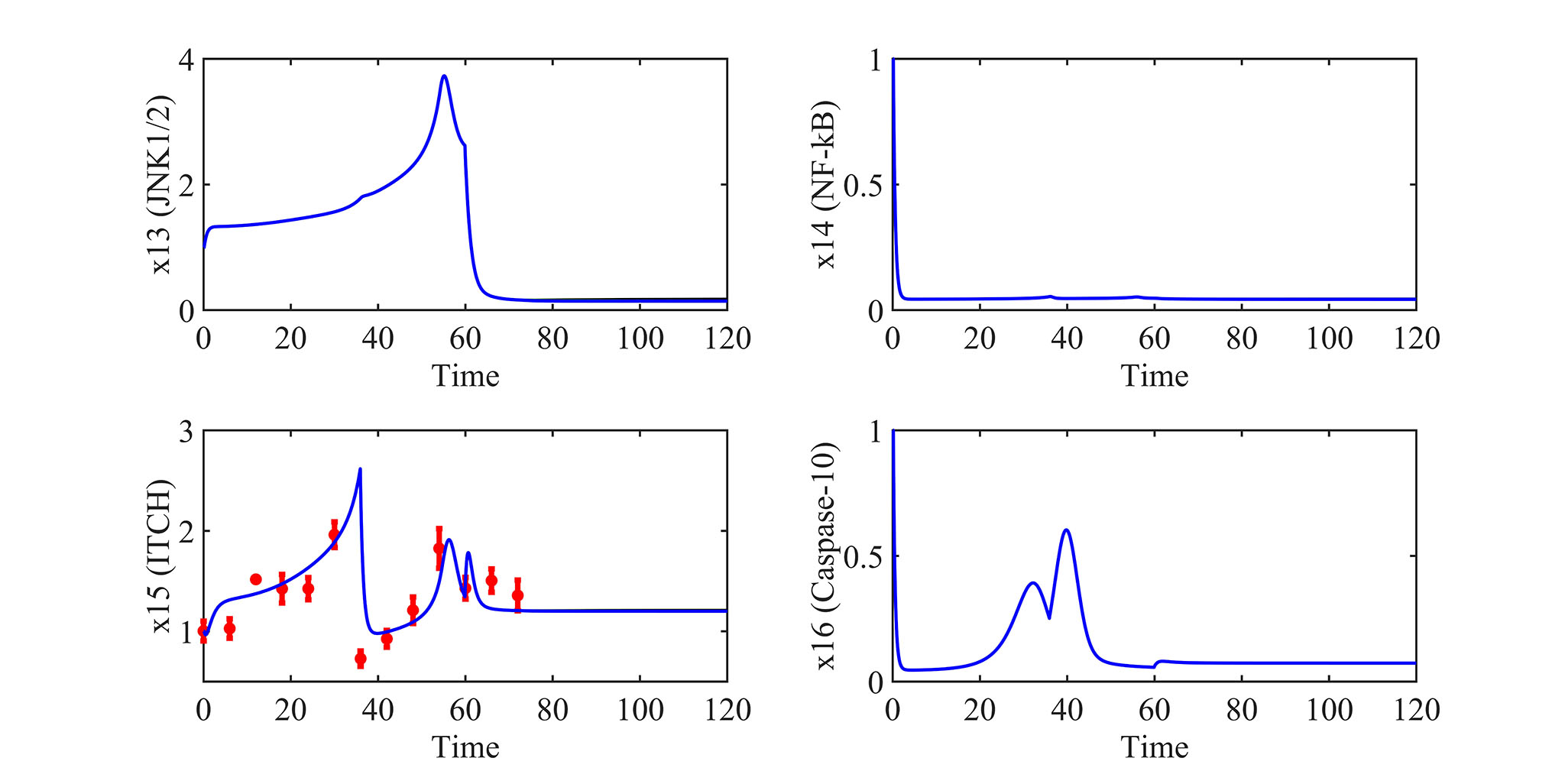

Supplement: Supplementary file 4 [file DataSheet2.zip › Supplementary material_image2/Parameter_d9(小)/4.jpg]

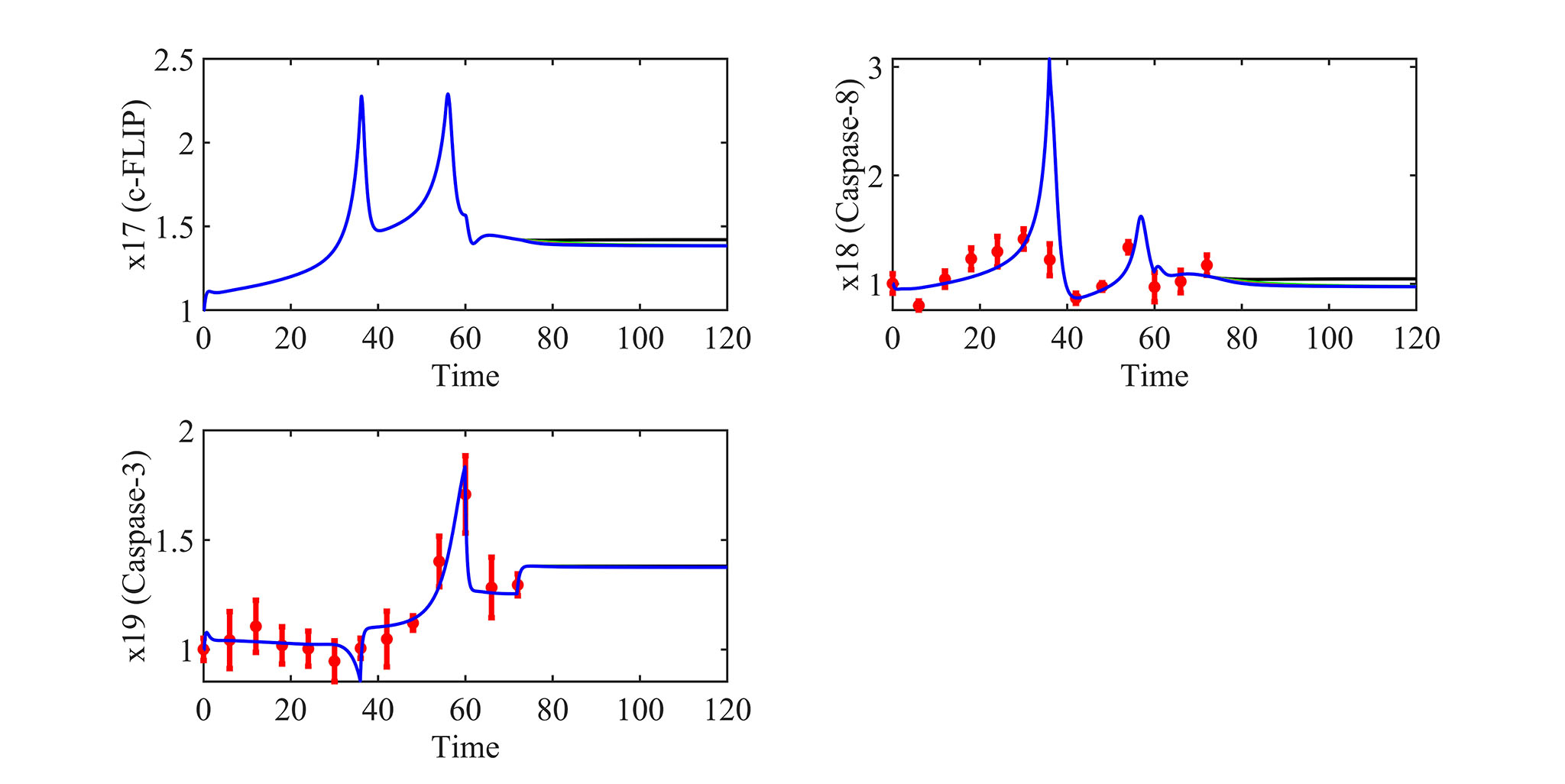

Supplement: Supplementary file 4 [file DataSheet2.zip › Supplementary material_image2/Parameter_d9(小)/5.jpg]

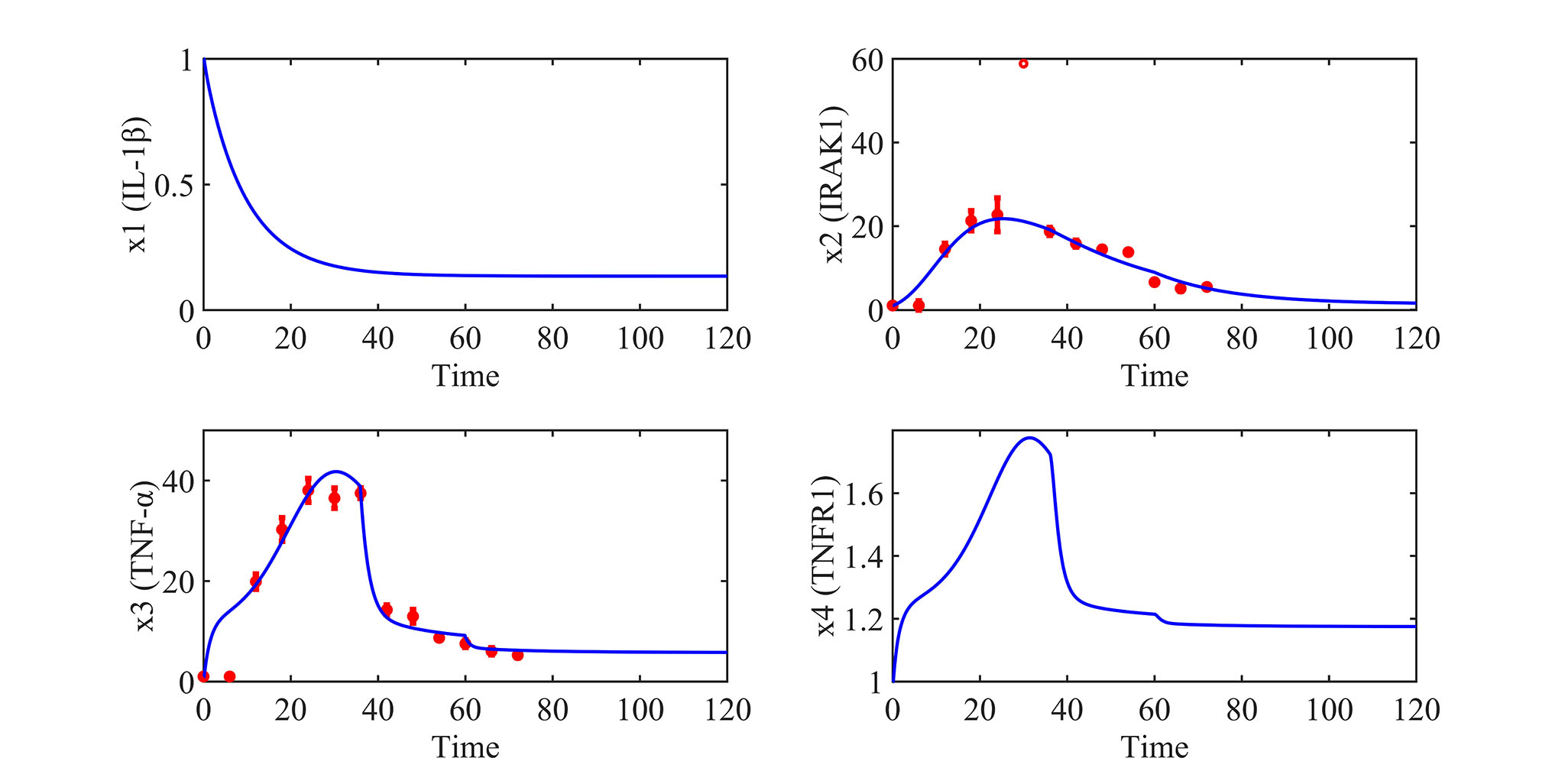

Supplement: Supplementary file 4 [file DataSheet2.zip › Supplementary material_image2/Parameter_f10(大)/1.jpg]

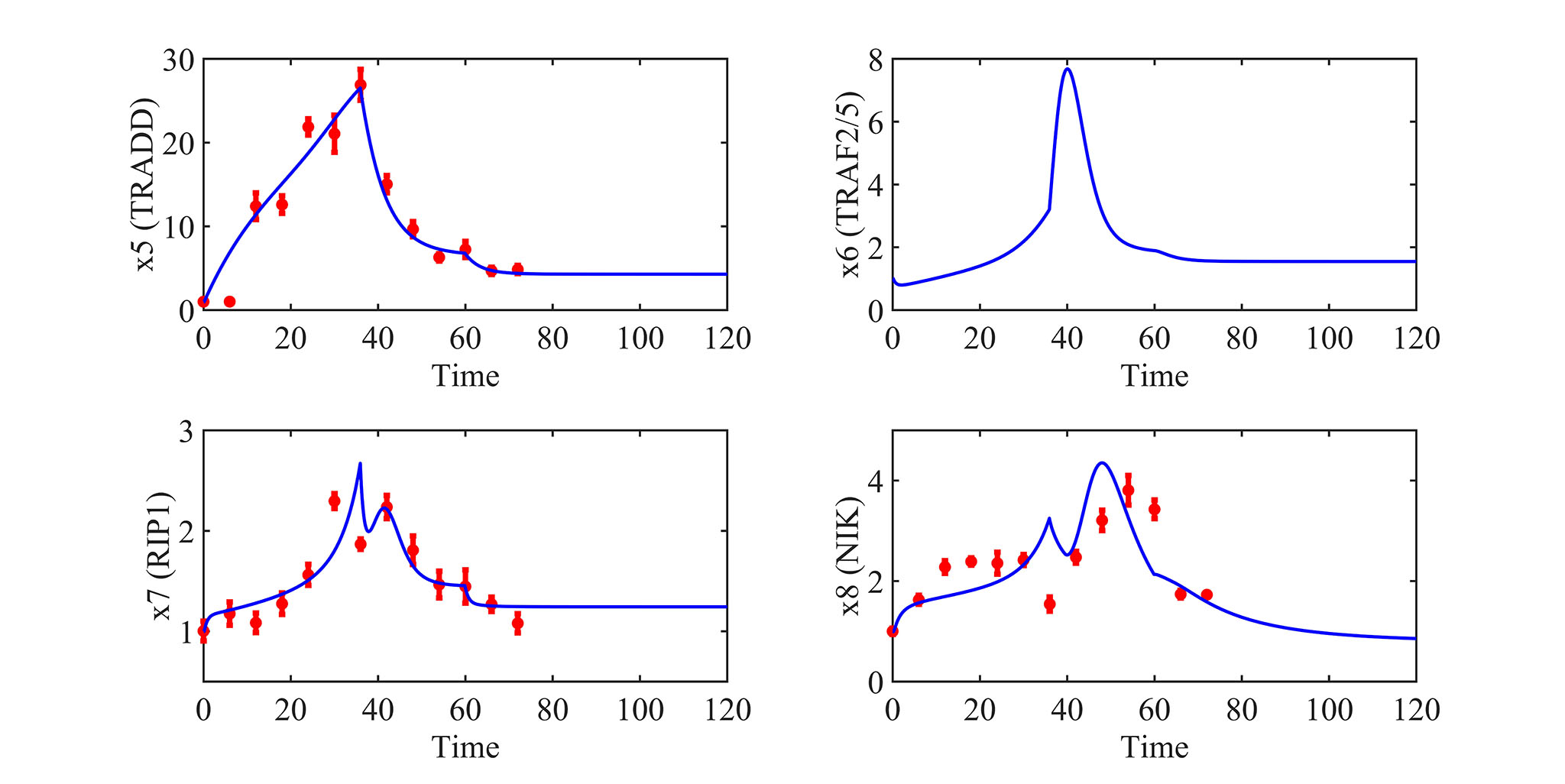

Supplement: Supplementary file 4 [file DataSheet2.zip › Supplementary material_image2/Parameter_f10(大)/2.jpg]

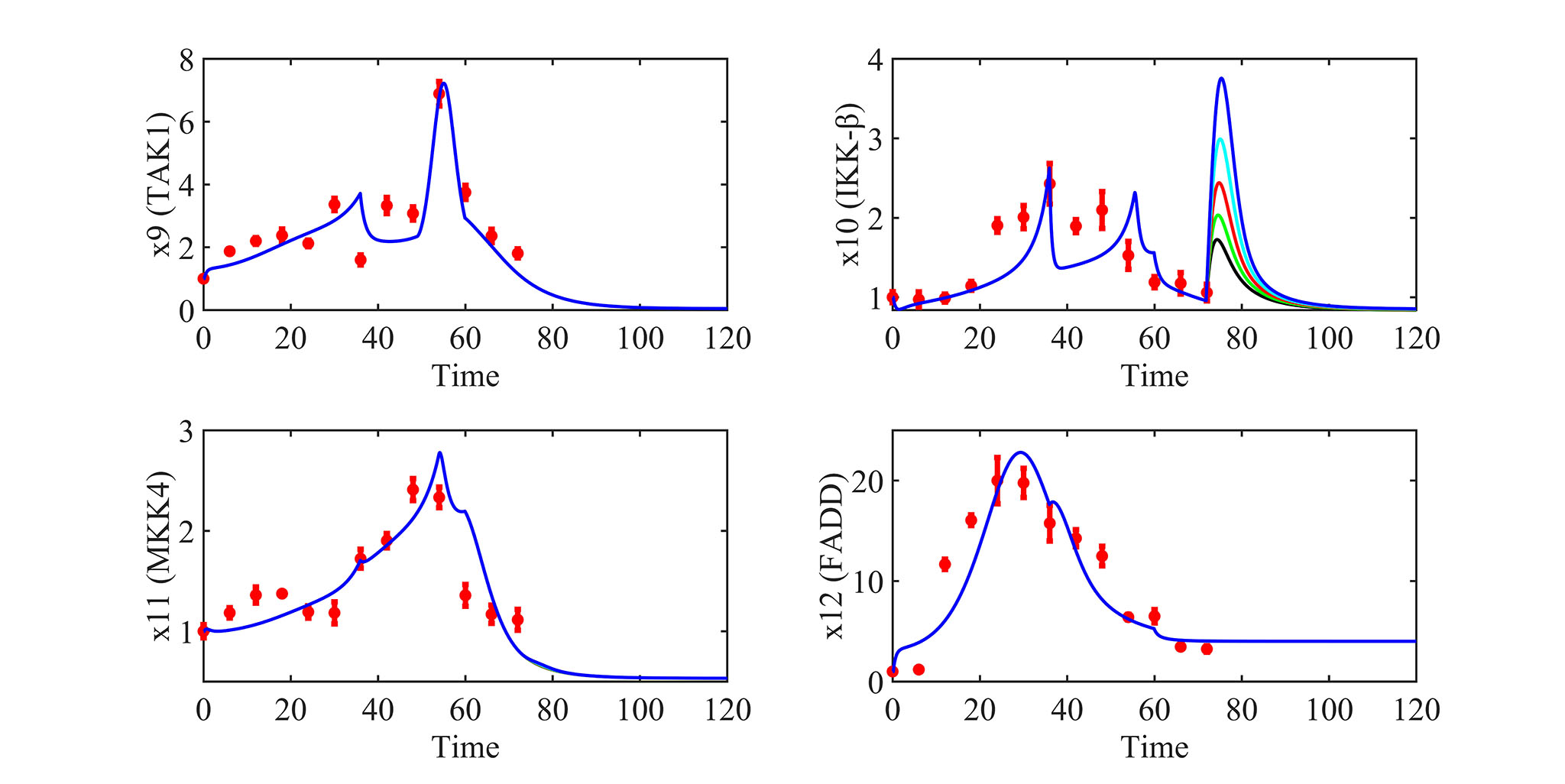

Supplement: Supplementary file 4 [file DataSheet2.zip › Supplementary material_image2/Parameter_f10(大)/3.jpg]

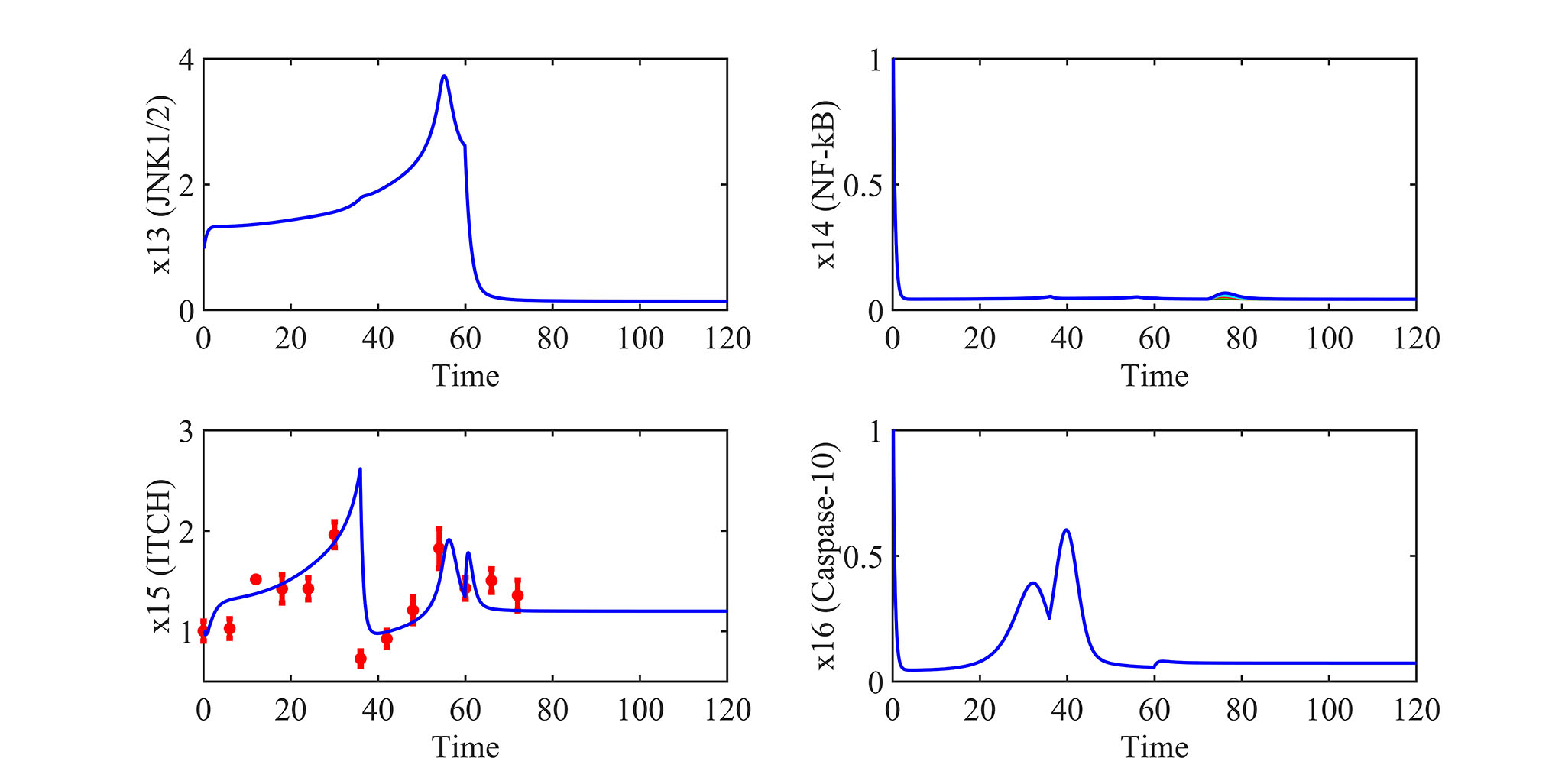

Supplement: Supplementary file 4 [file DataSheet2.zip › Supplementary material_image2/Parameter_f10(大)/4.jpg]

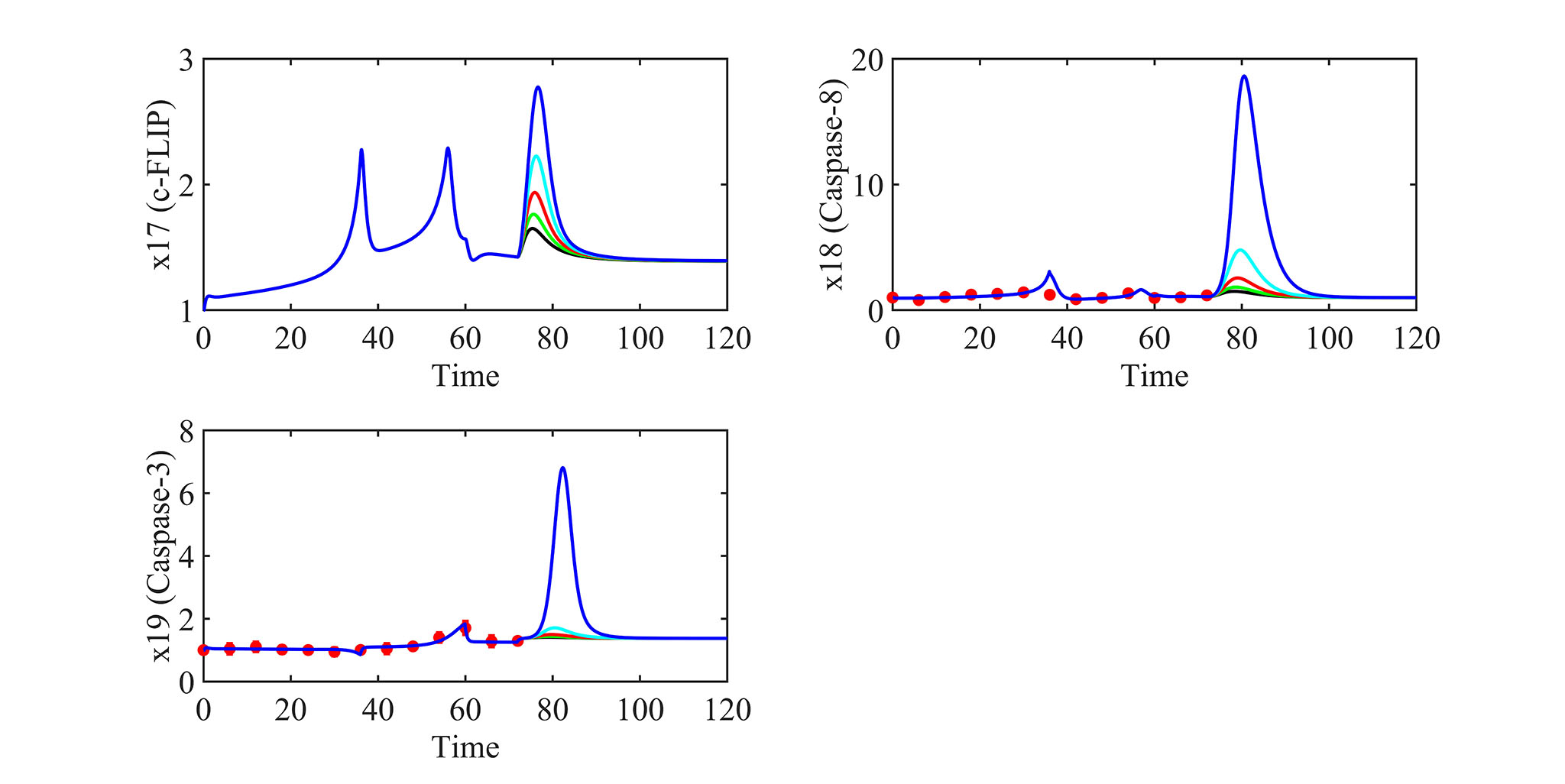

Supplement: Supplementary file 4 [file DataSheet2.zip › Supplementary material_image2/Parameter_f10(大)/5.jpg]

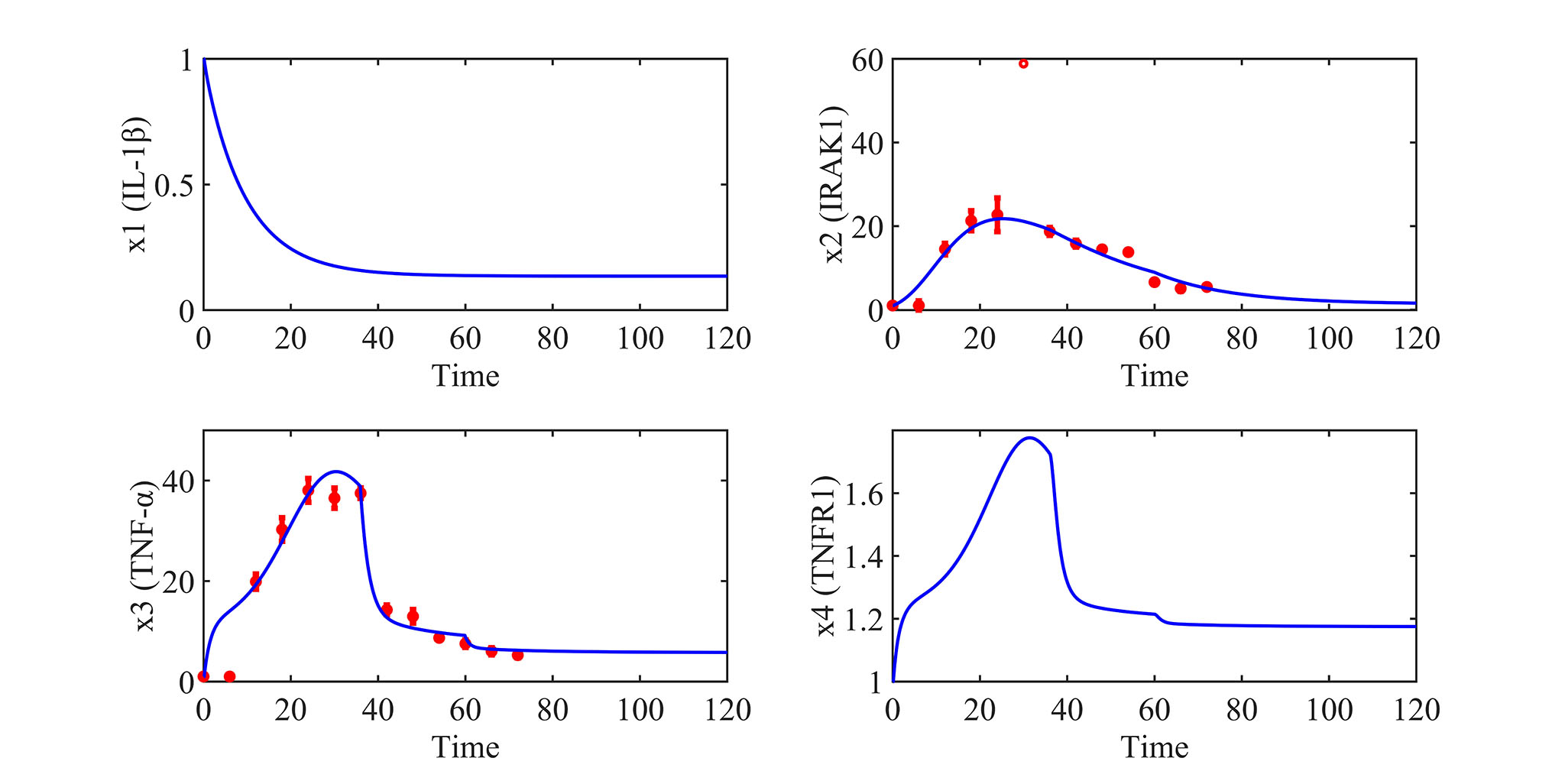

Supplement: Supplementary file 4 [file DataSheet2.zip › Supplementary material_image2/Parameter_g10(大)/1.jpg]

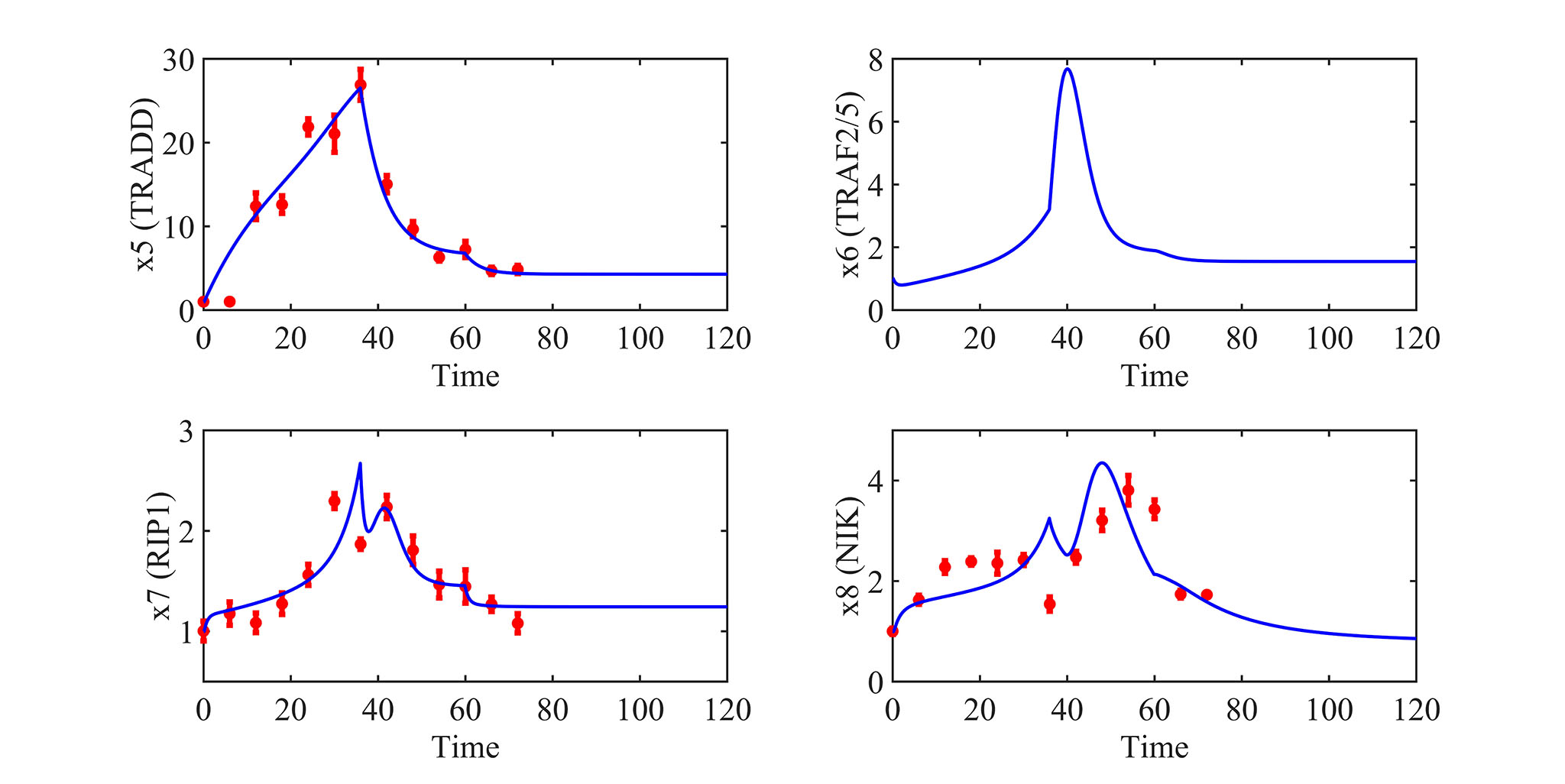

Supplement: Supplementary file 4 [file DataSheet2.zip › Supplementary material_image2/Parameter_g10(大)/2.jpg]

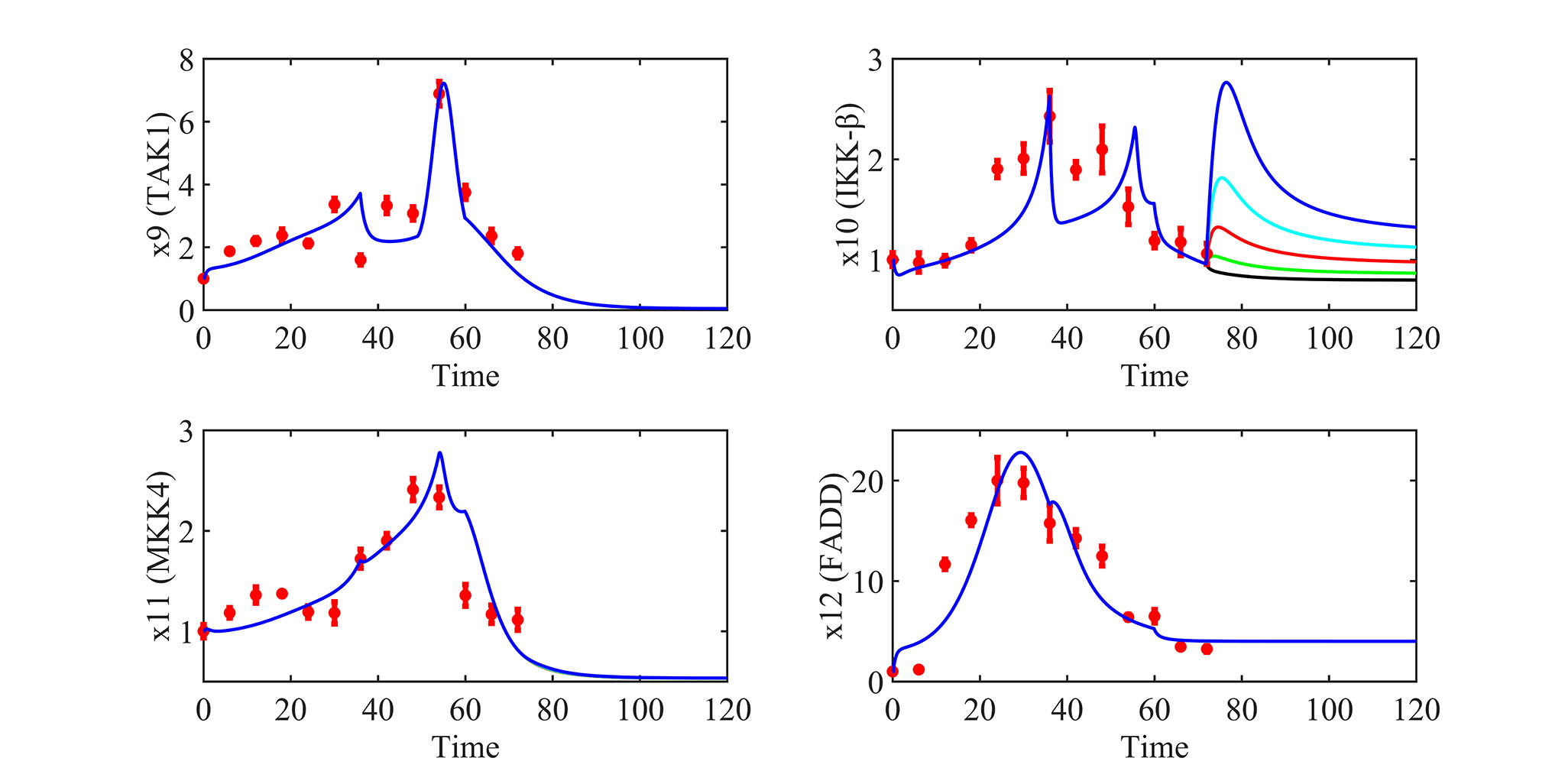

Supplement: Supplementary file 4 [file DataSheet2.zip › Supplementary material_image2/Parameter_g10(大)/3.jpg]

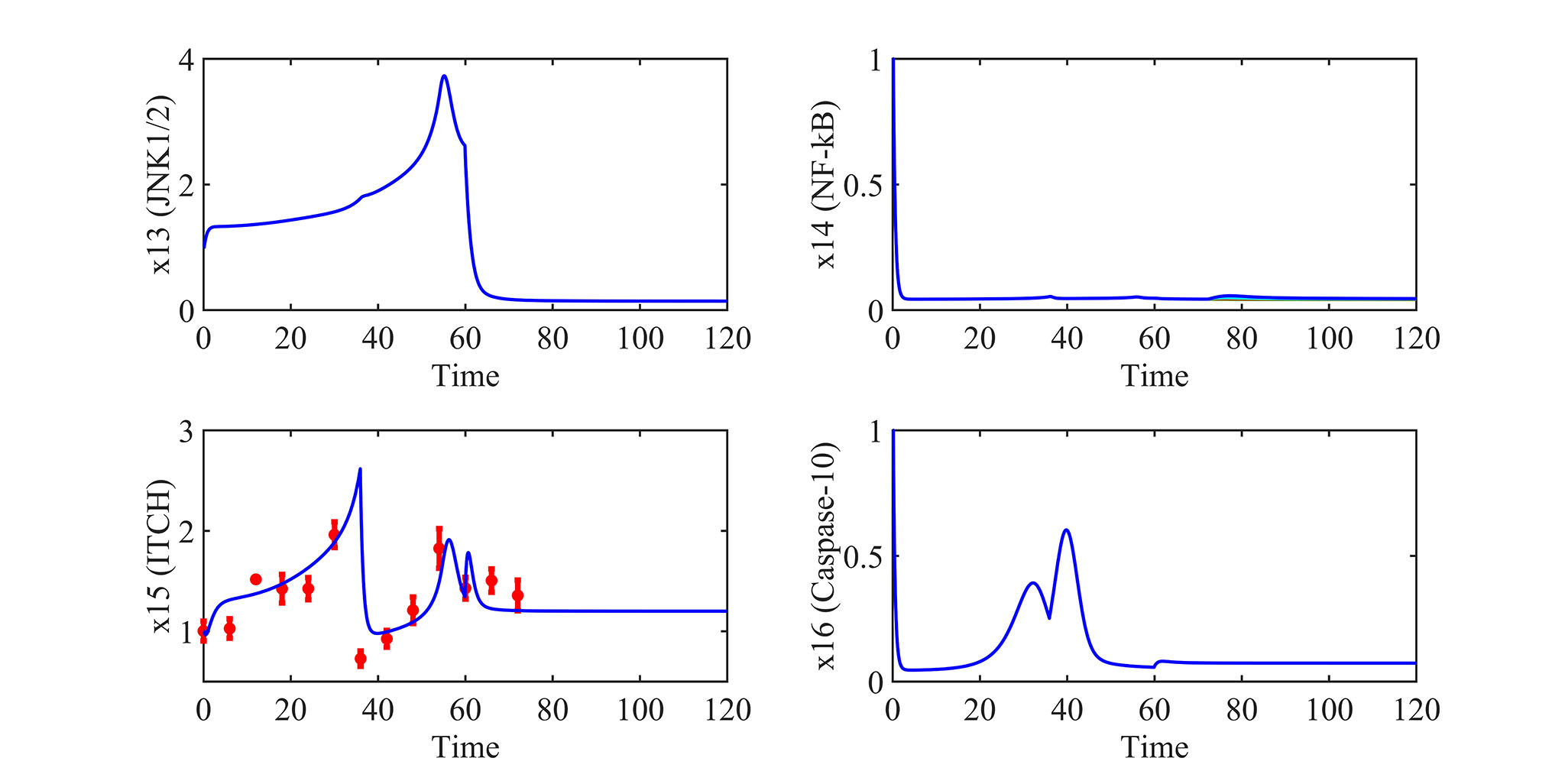

Supplement: Supplementary file 4 [file DataSheet2.zip › Supplementary material_image2/Parameter_g10(大)/4.jpg]

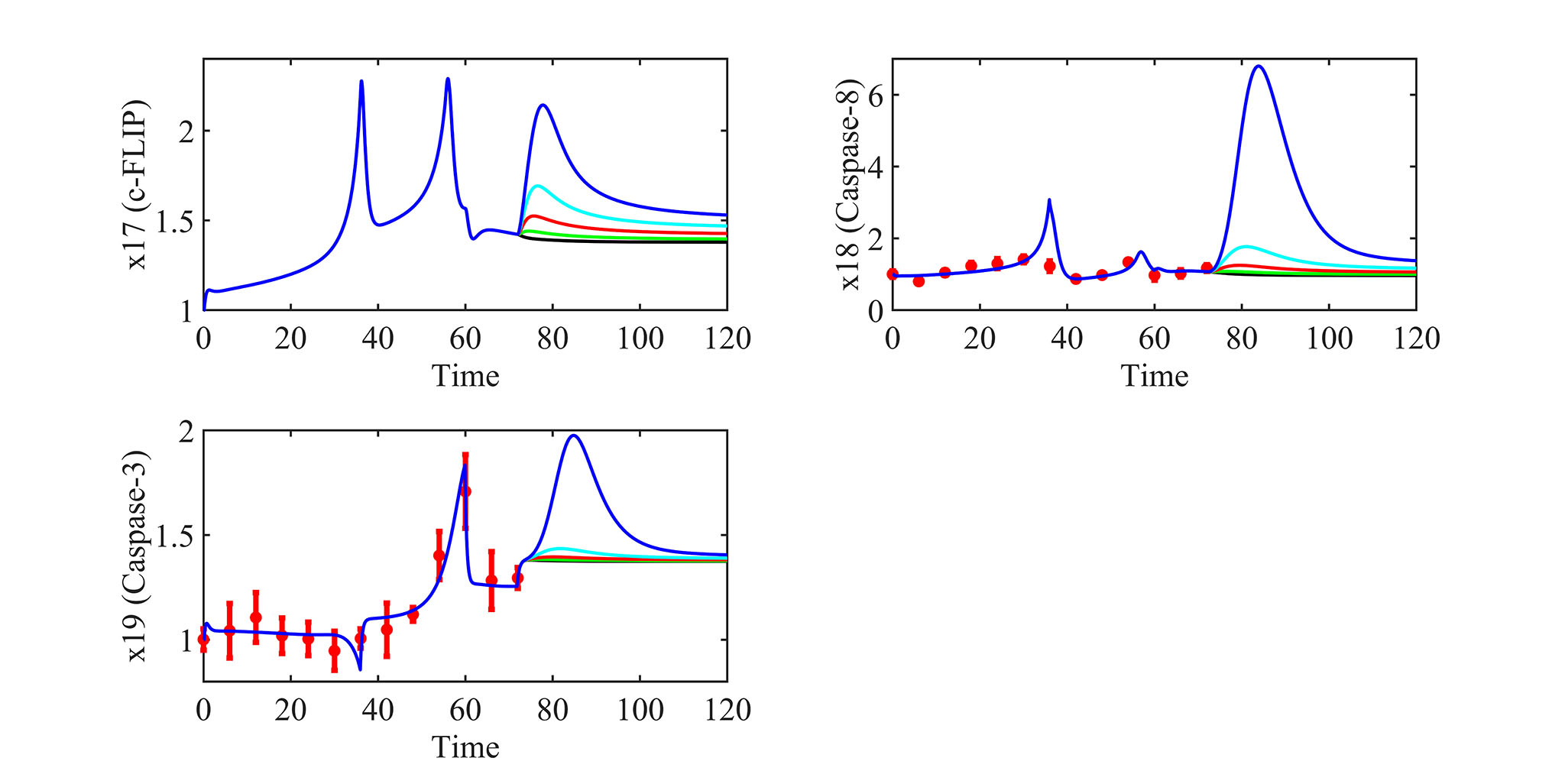

Supplement: Supplementary file 4 [file DataSheet2.zip › Supplementary material_image2/Parameter_g10(大)/5.jpg]

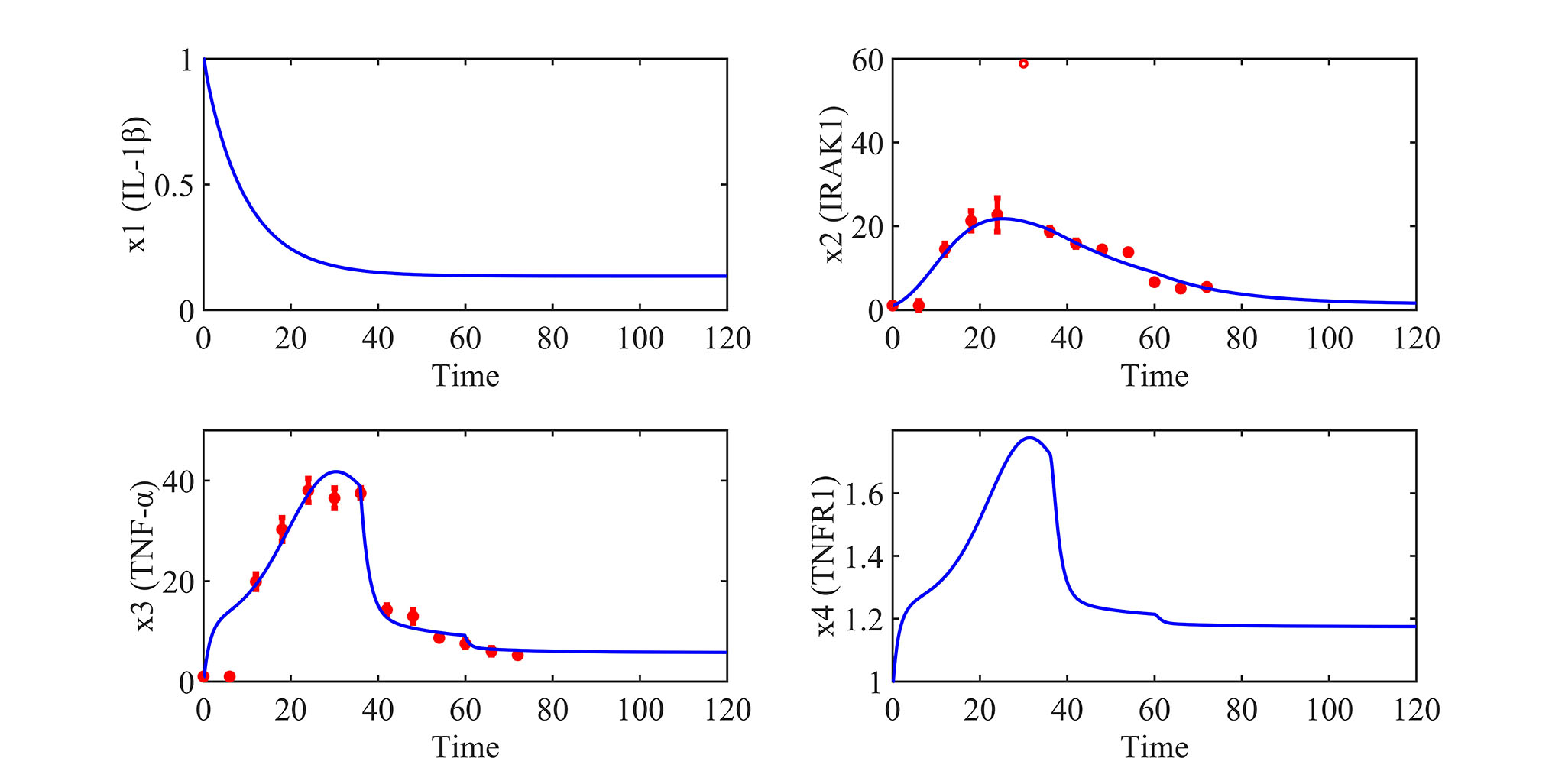

Supplement: Supplementary file 4 [file DataSheet2.zip › Supplementary material_image2/Parameter_g11(小)/1.jpg]

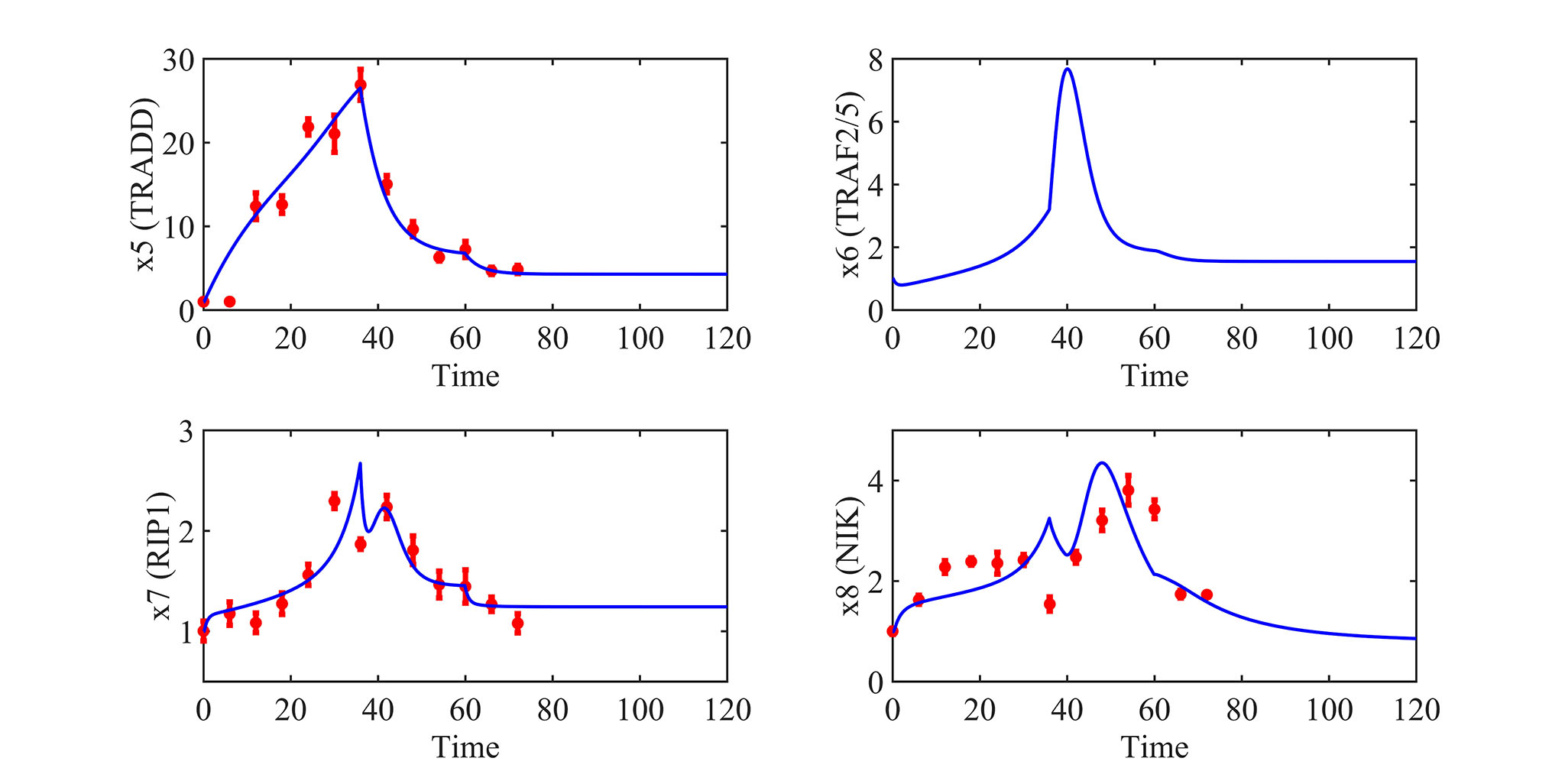

Supplement: Supplementary file 4 [file DataSheet2.zip › Supplementary material_image2/Parameter_g11(小)/2.jpg]

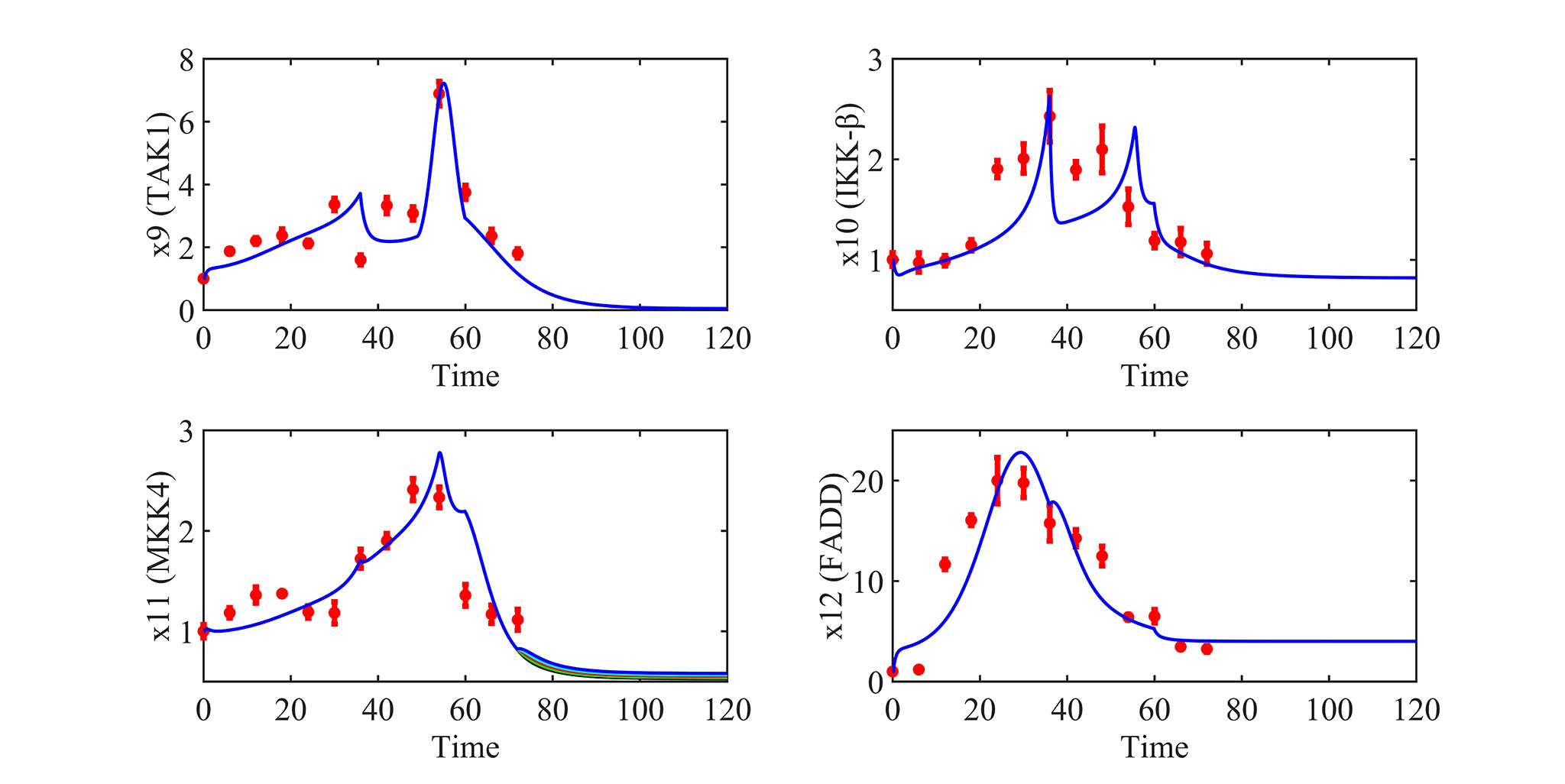

Supplement: Supplementary file 4 [file DataSheet2.zip › Supplementary material_image2/Parameter_g11(小)/3.jpg]

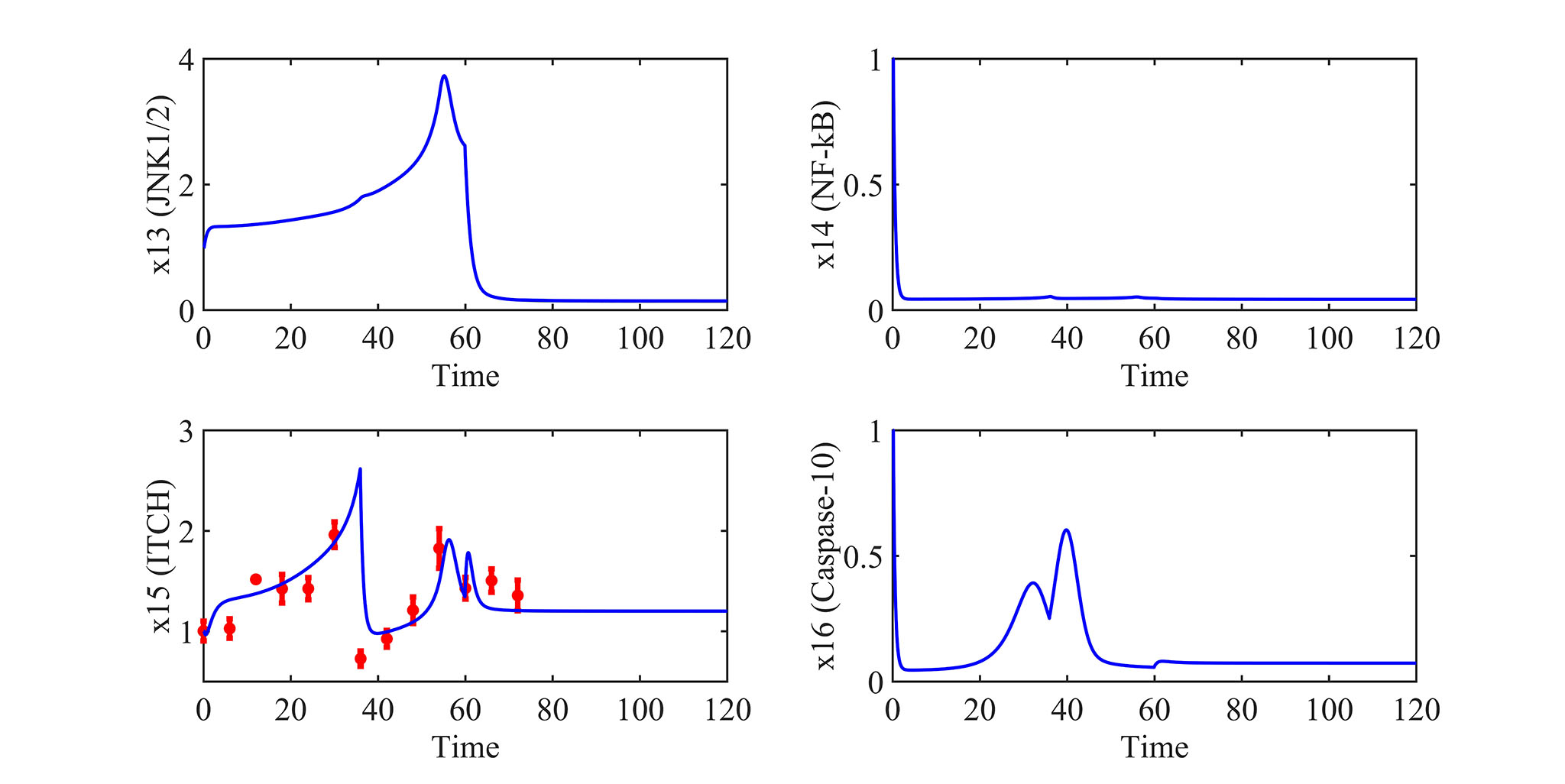

Supplement: Supplementary file 4 [file DataSheet2.zip › Supplementary material_image2/Parameter_g11(小)/4.jpg]

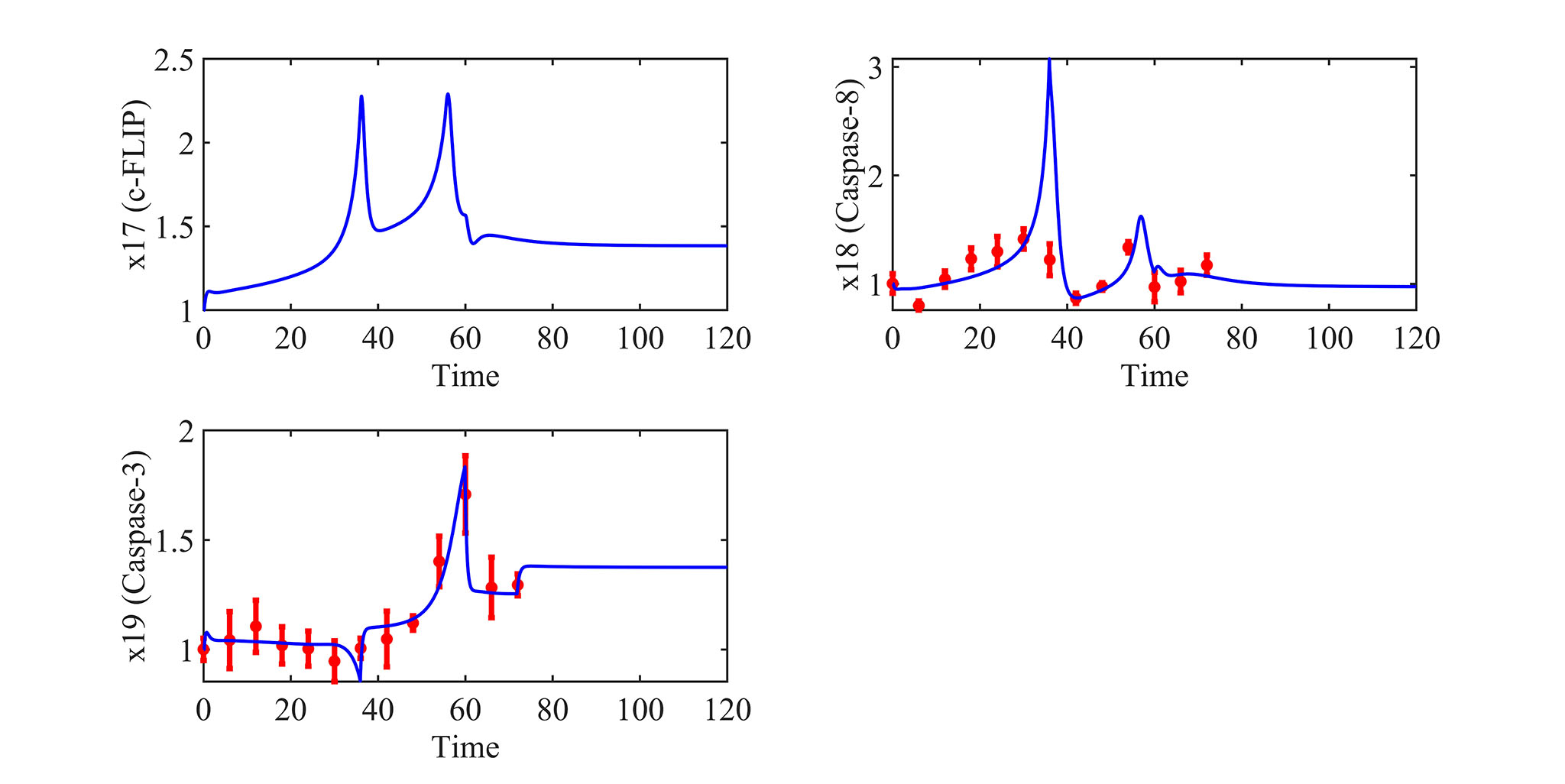

Supplement: Supplementary file 4 [file DataSheet2.zip › Supplementary material_image2/Parameter_g11(小)/5.jpg]

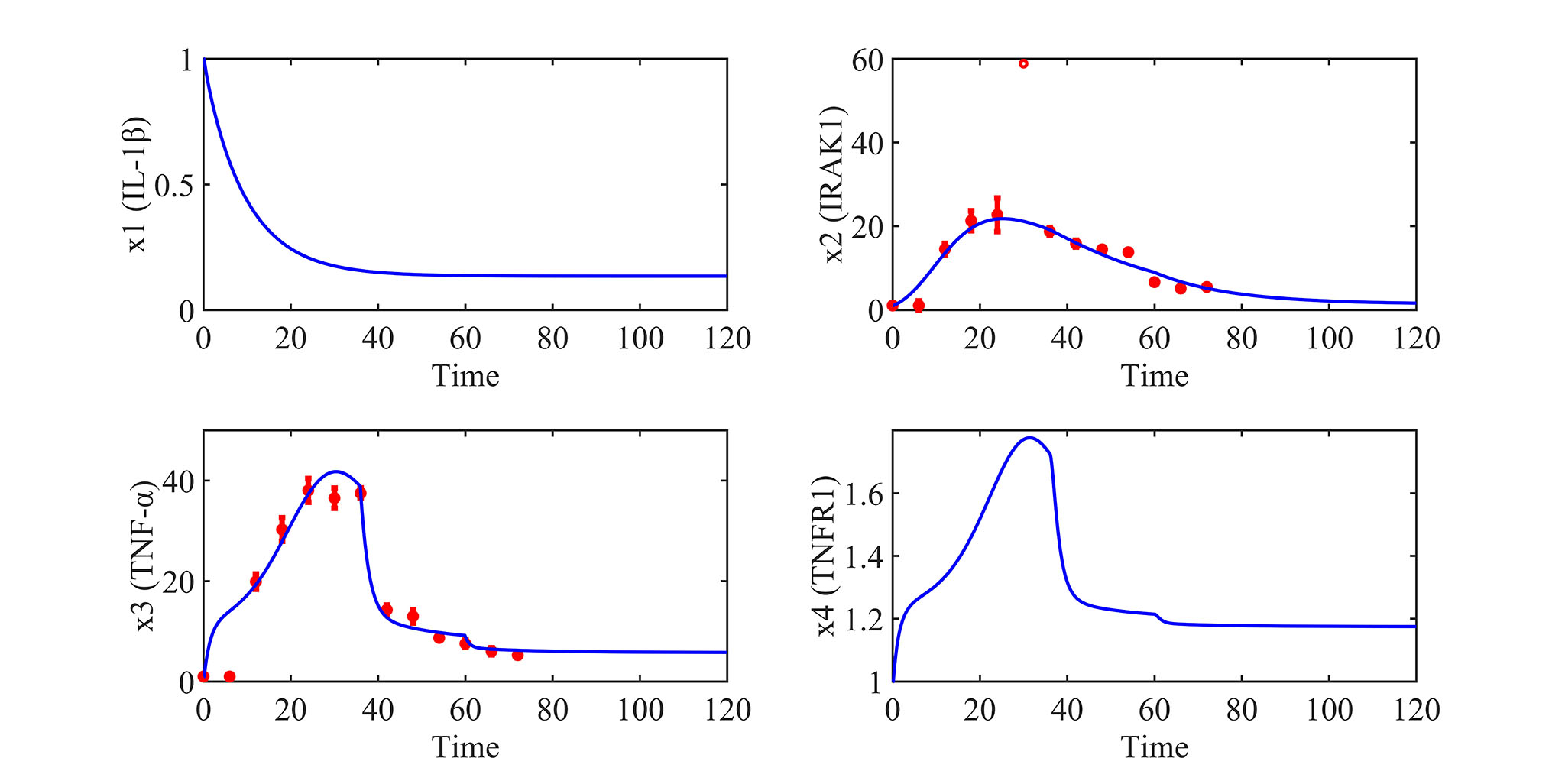

Supplement: Supplementary file 4 [file DataSheet2.zip › Supplementary material_image2/Parameter_g17(中)/1.jpg]

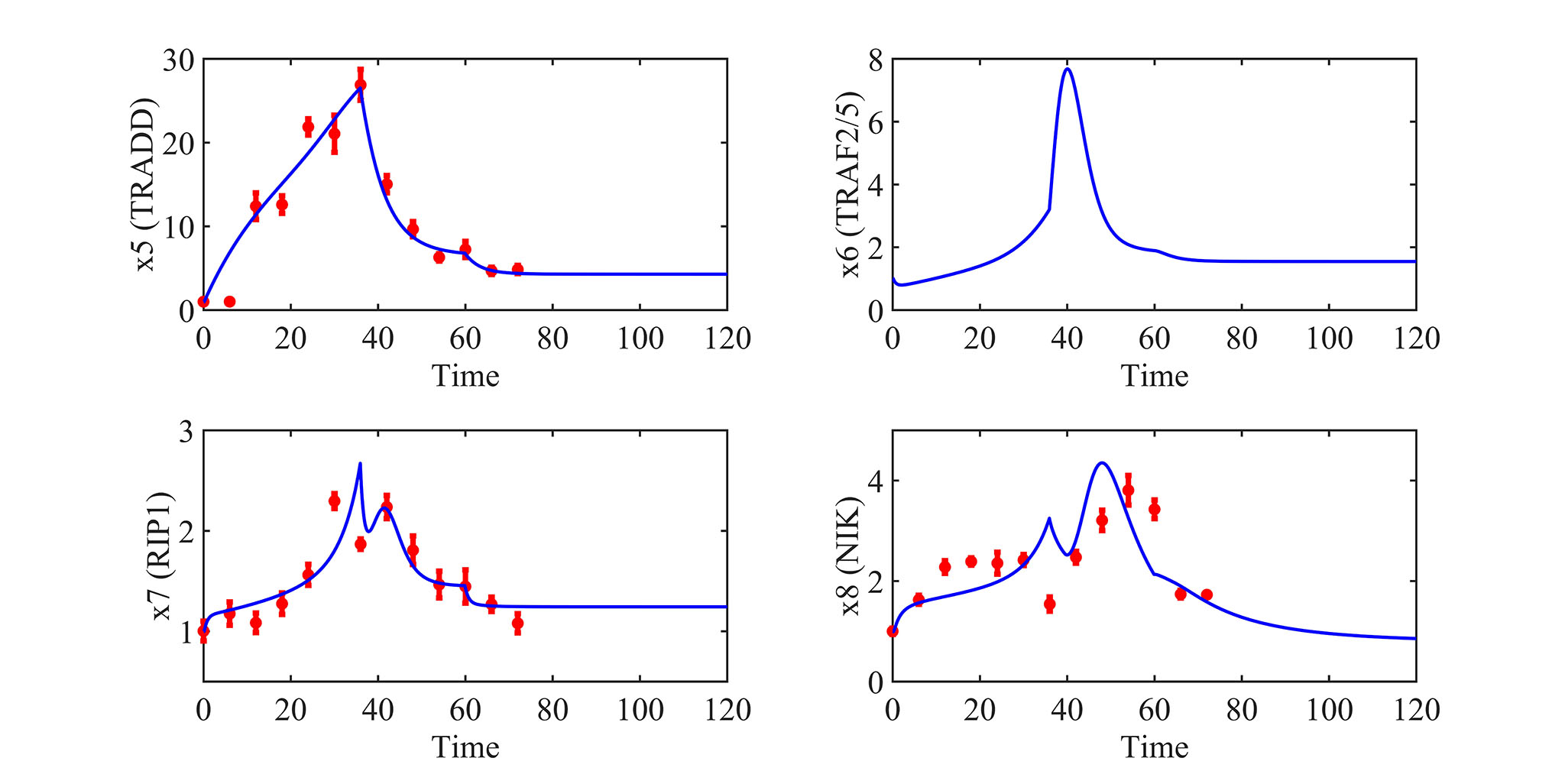

Supplement: Supplementary file 4 [file DataSheet2.zip › Supplementary material_image2/Parameter_g17(中)/2.jpg]

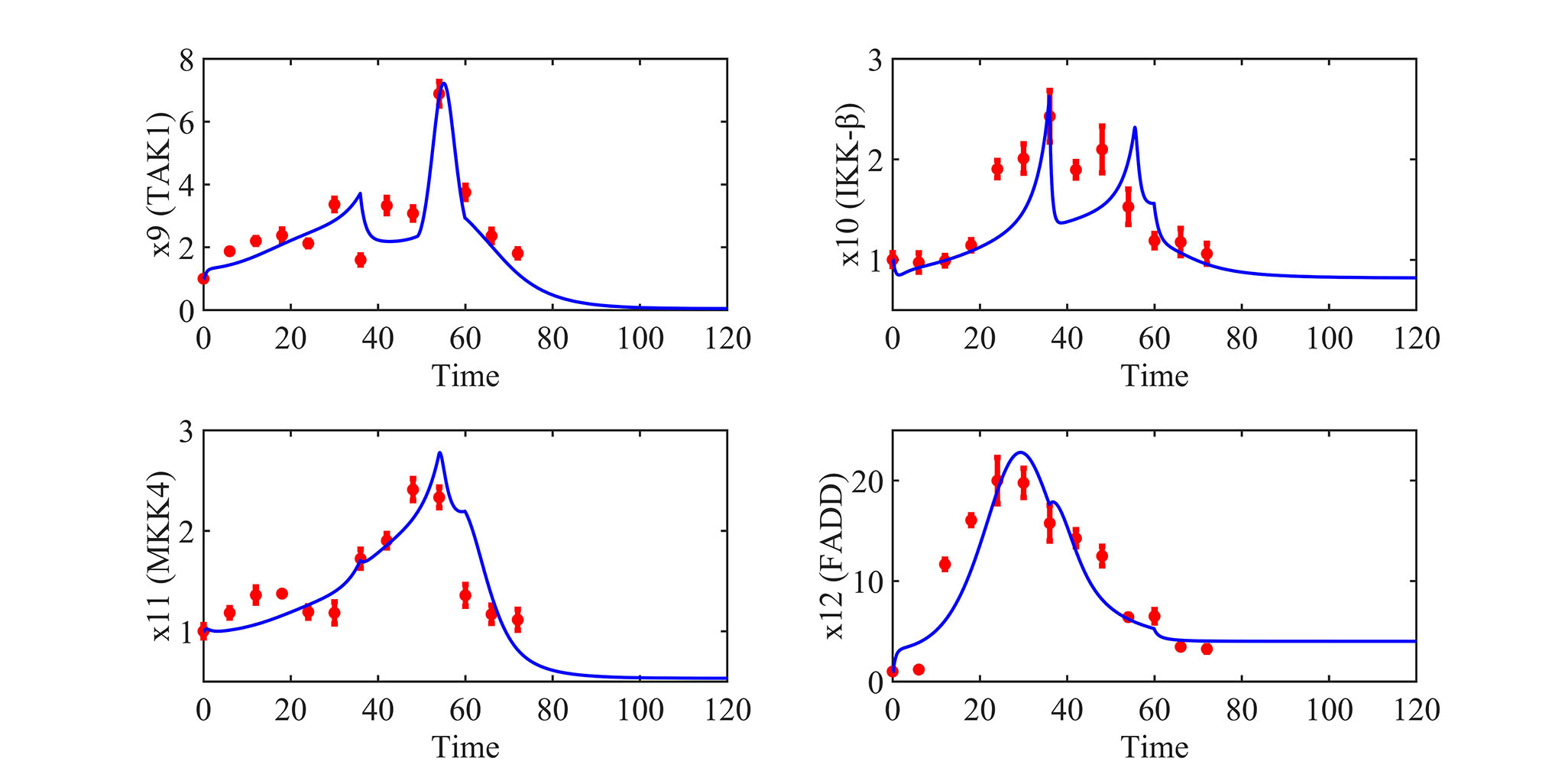

Supplement: Supplementary file 4 [file DataSheet2.zip › Supplementary material_image2/Parameter_g17(中)/3.jpg]

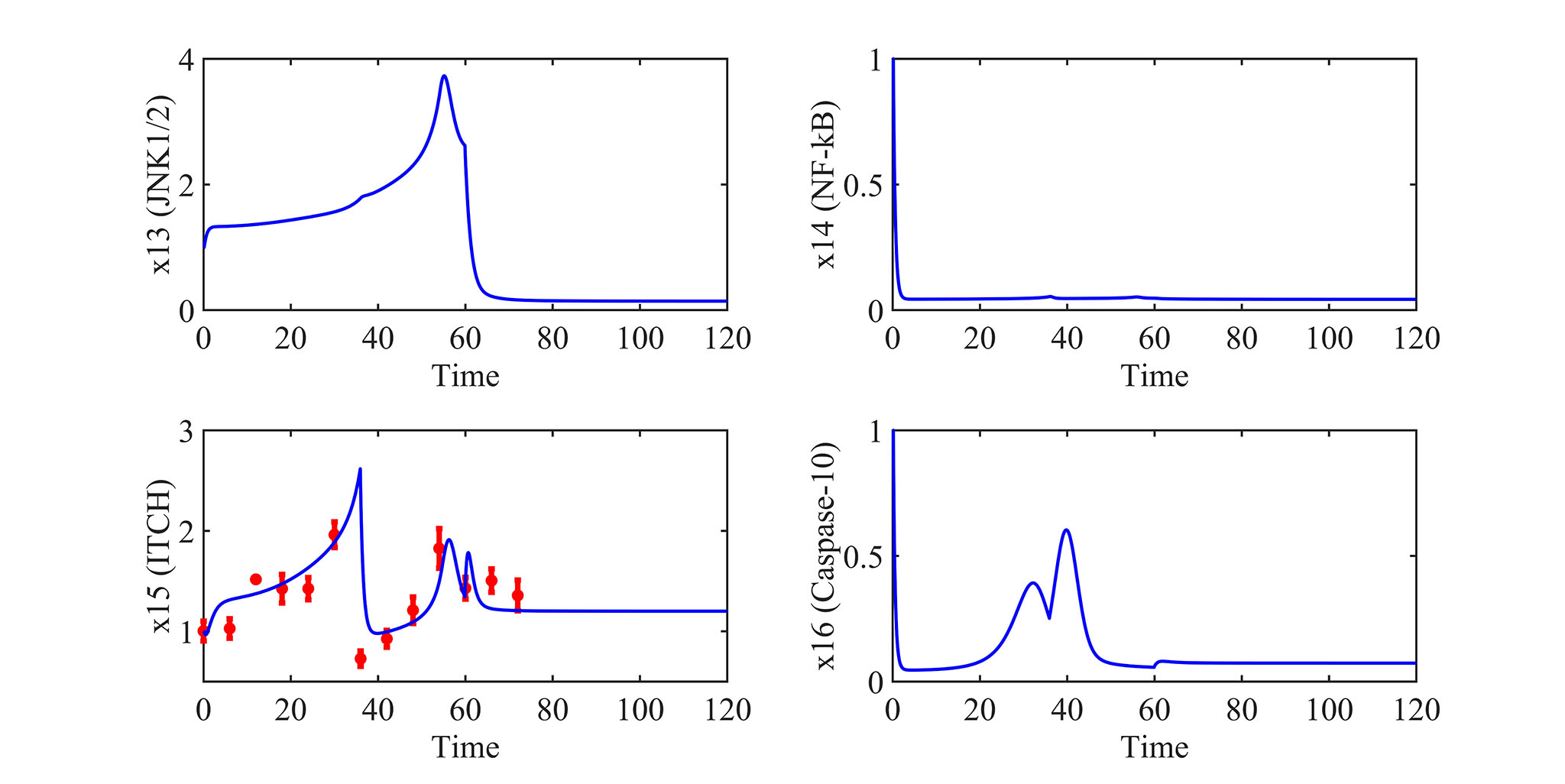

Supplement: Supplementary file 4 [file DataSheet2.zip › Supplementary material_image2/Parameter_g17(中)/4.jpg]

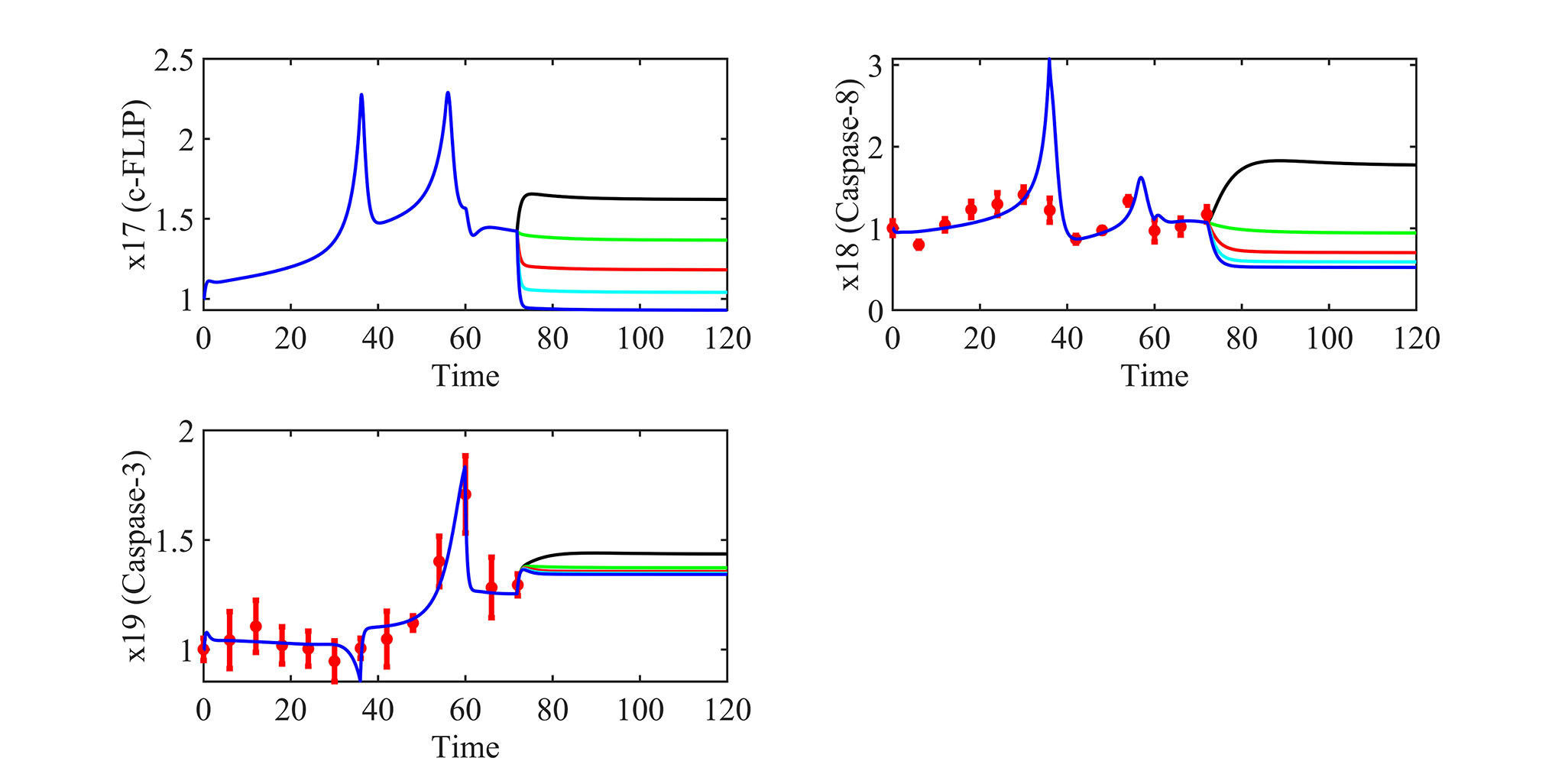

Supplement: Supplementary file 4 [file DataSheet2.zip › Supplementary material_image2/Parameter_g17(中)/5.jpg]

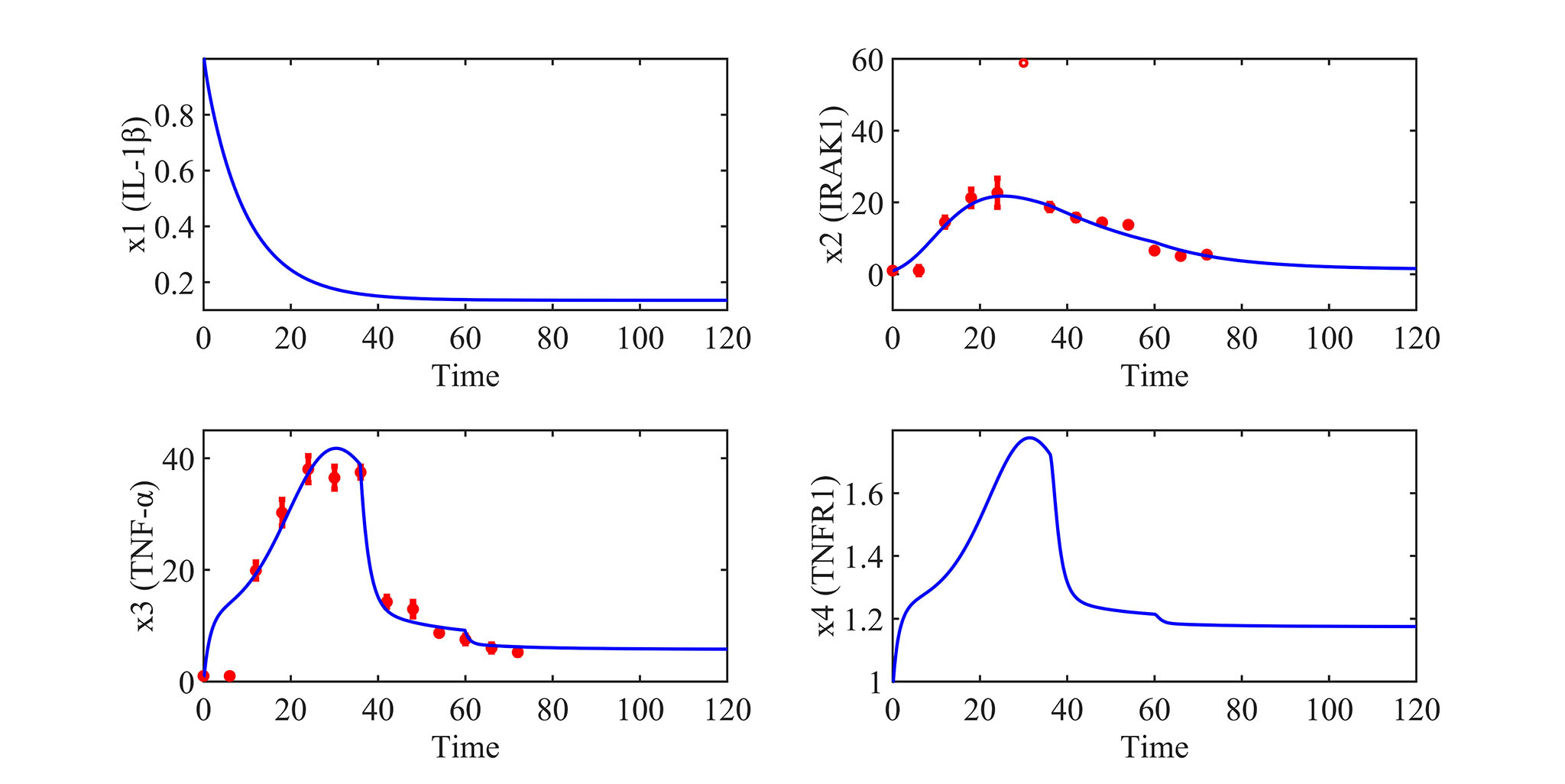

Supplement: Supplementary file 4 [file DataSheet2.zip › Supplementary material_image2/Parameter_g18(小)/1.jpg]

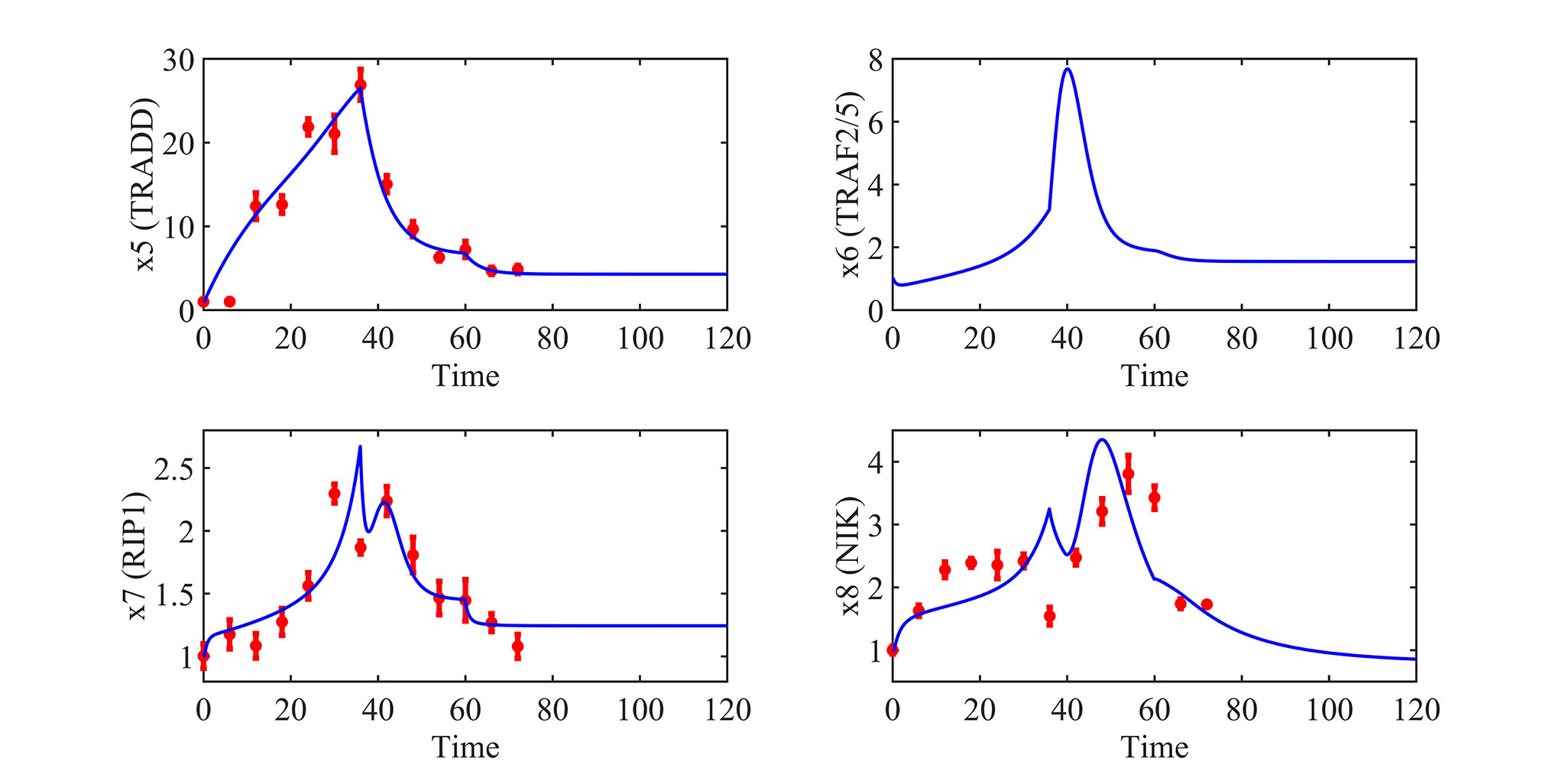

Supplement: Supplementary file 4 [file DataSheet2.zip › Supplementary material_image2/Parameter_g18(小)/2.jpg]

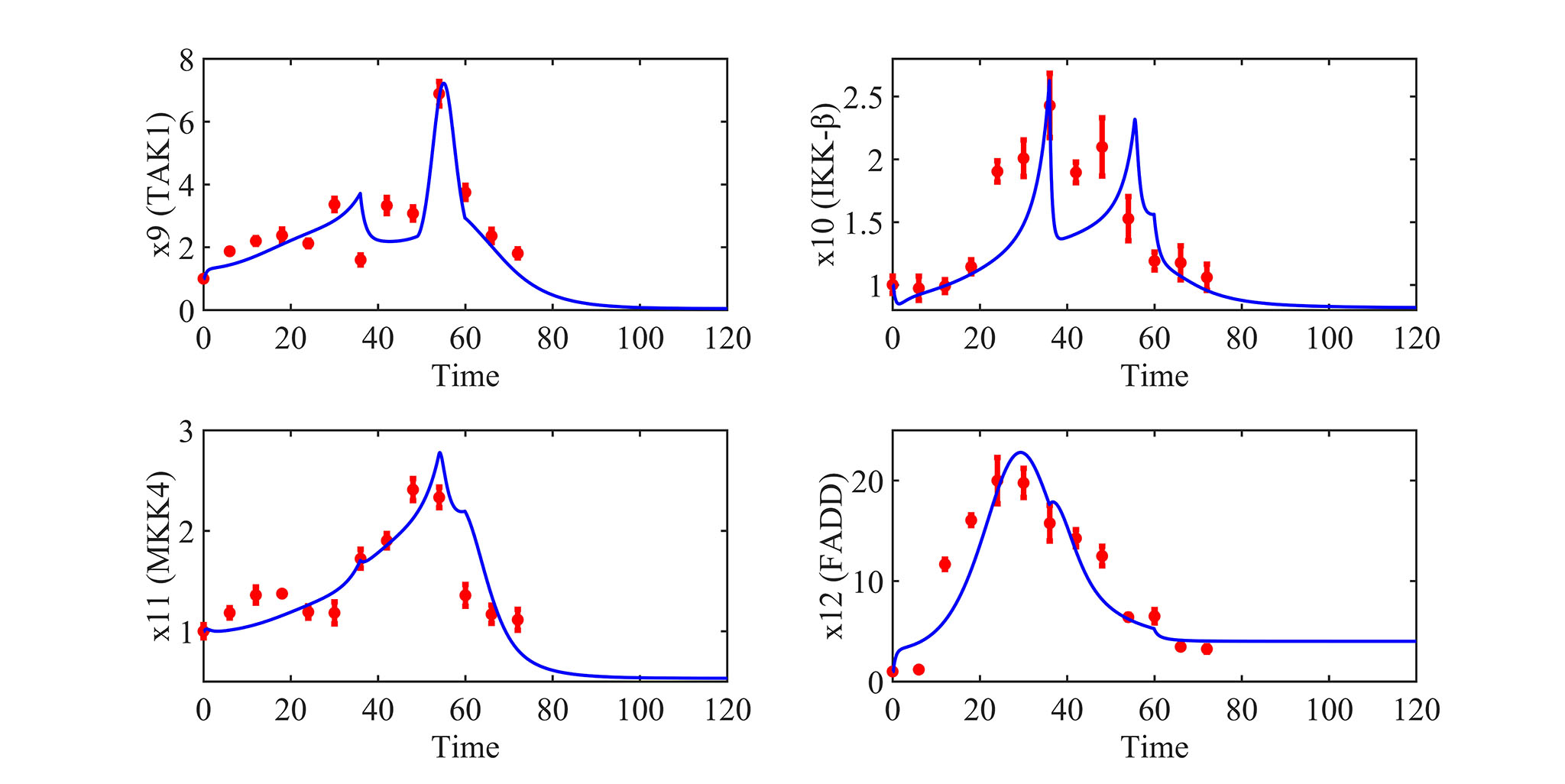

Supplement: Supplementary file 4 [file DataSheet2.zip › Supplementary material_image2/Parameter_g18(小)/3.jpg]

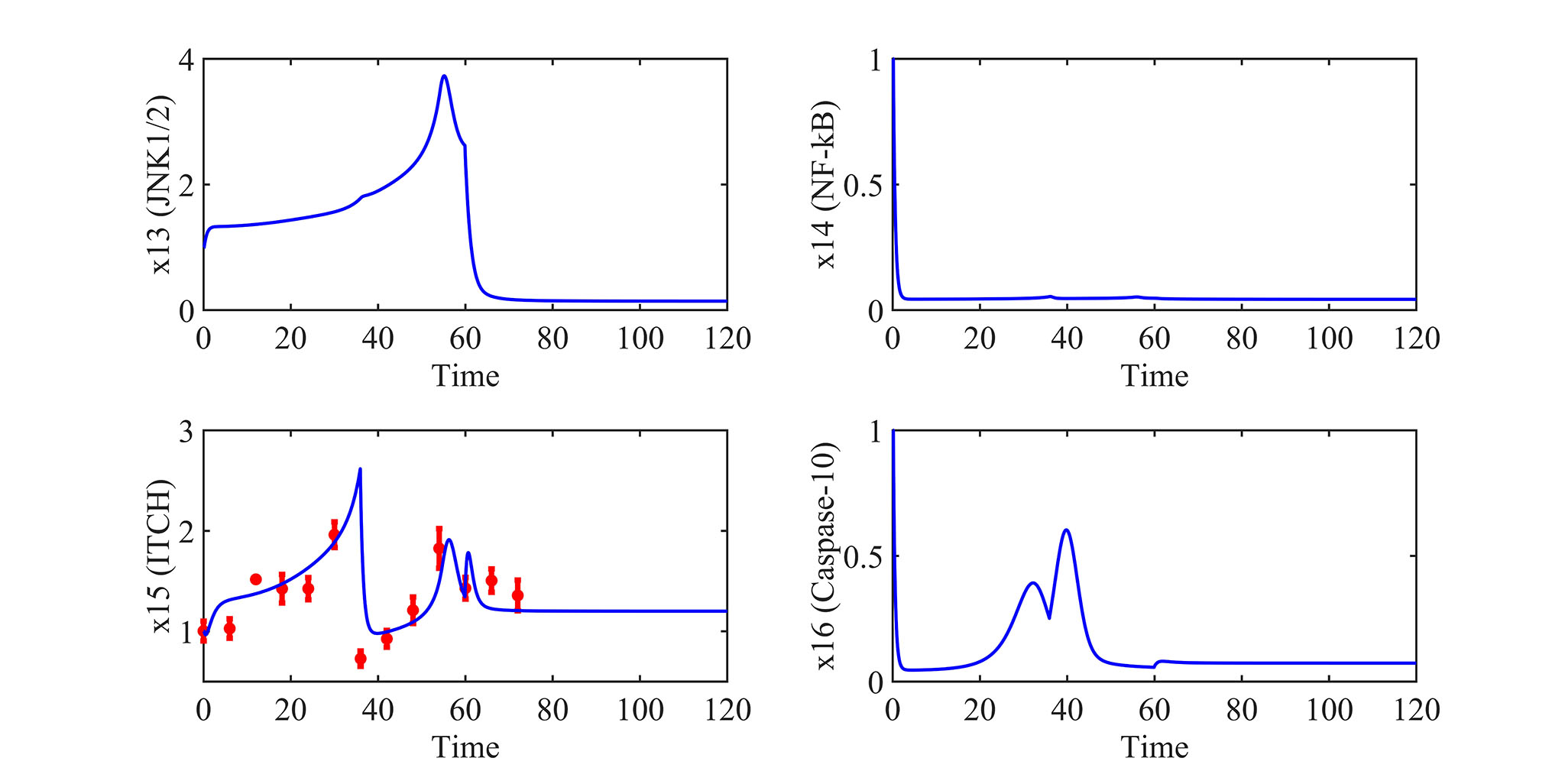

Supplement: Supplementary file 4 [file DataSheet2.zip › Supplementary material_image2/Parameter_g18(小)/4.jpg]

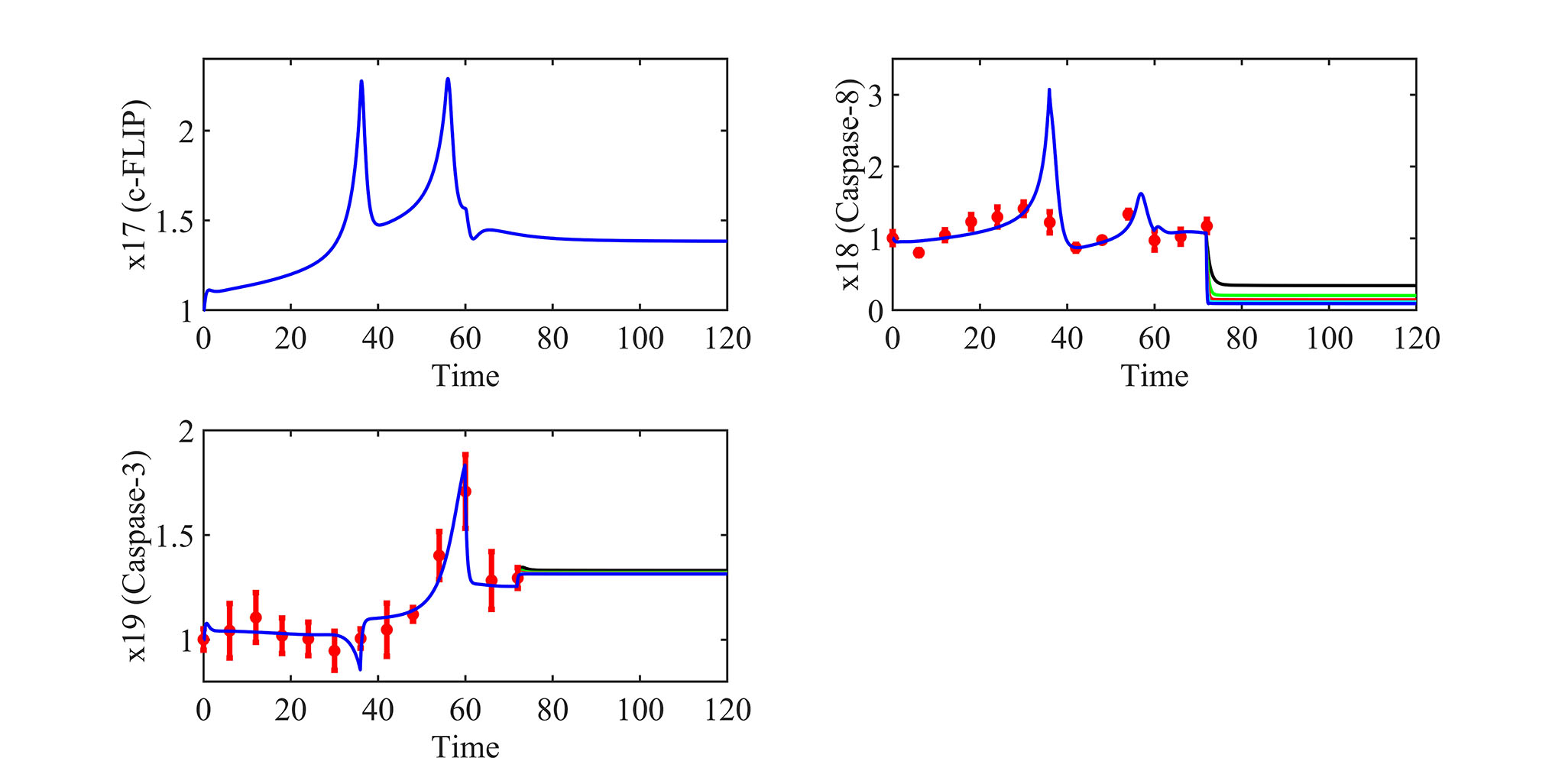

Supplement: Supplementary file 4 [file DataSheet2.zip › Supplementary material_image2/Parameter_g18(小)/5.jpg]

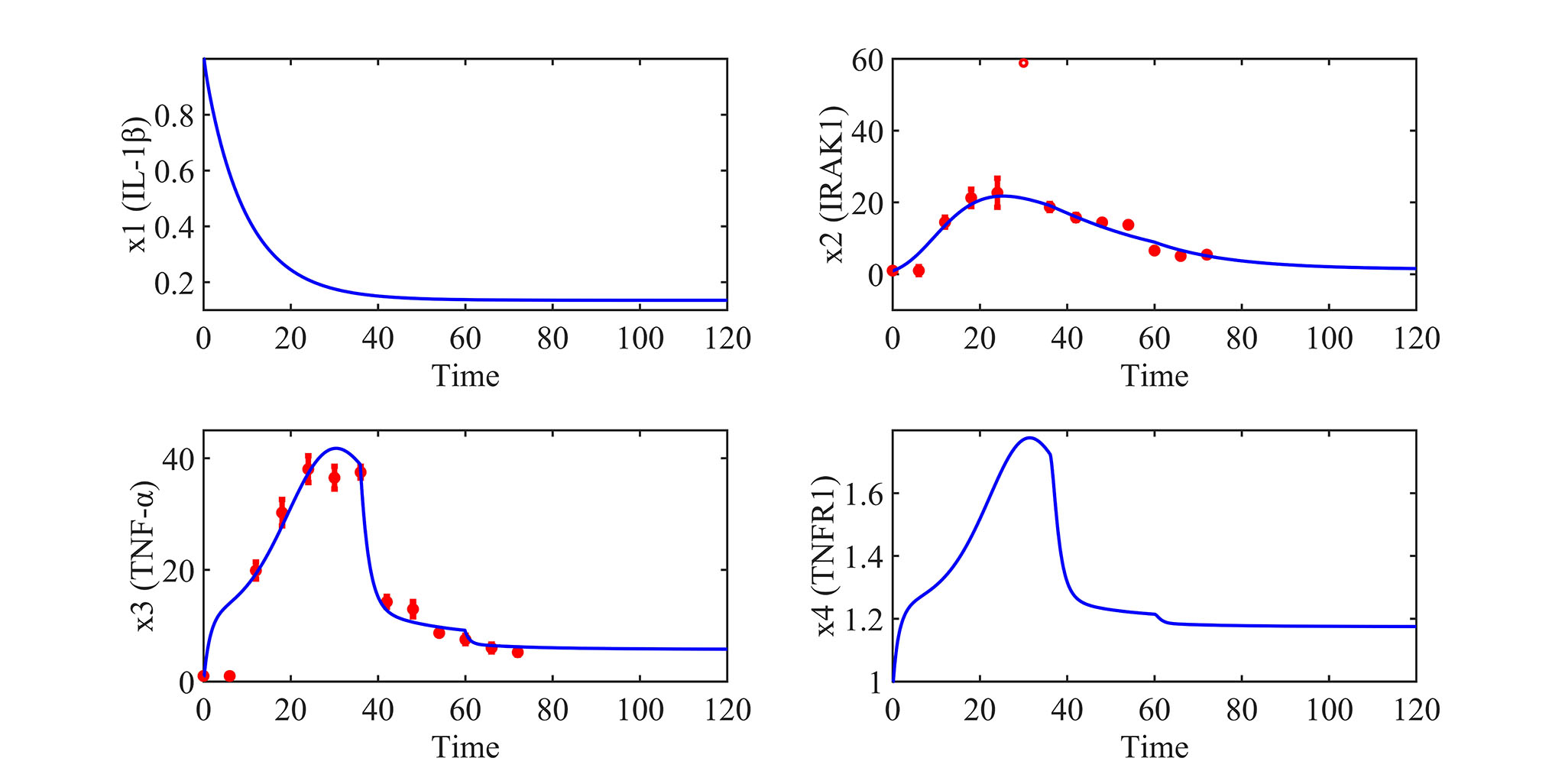

Supplement: Supplementary file 4 [file DataSheet2.zip › Supplementary material_image2/Parameter_g19(大)/1.jpg]

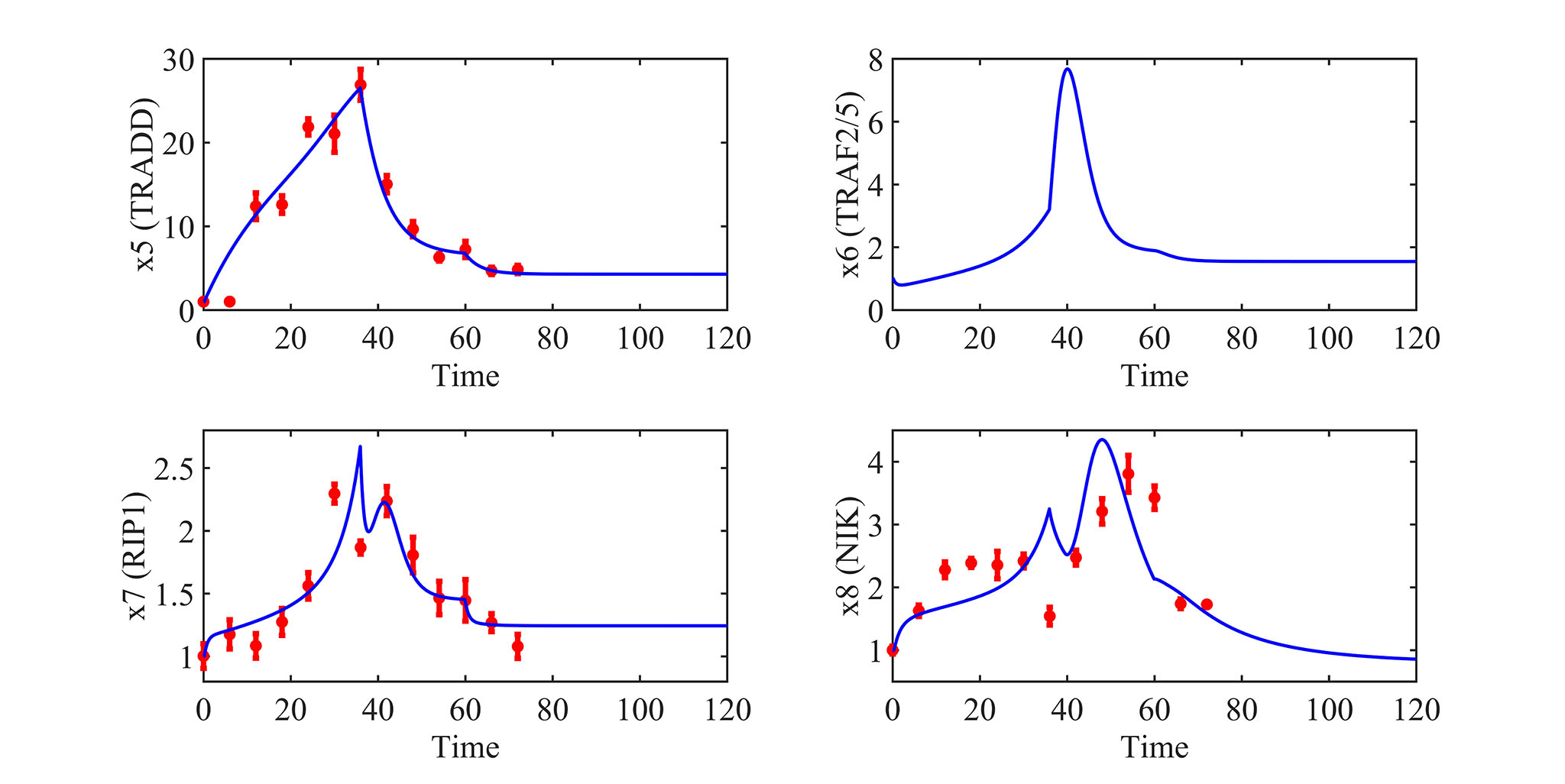

Supplement: Supplementary file 4 [file DataSheet2.zip › Supplementary material_image2/Parameter_g19(大)/2.jpg]

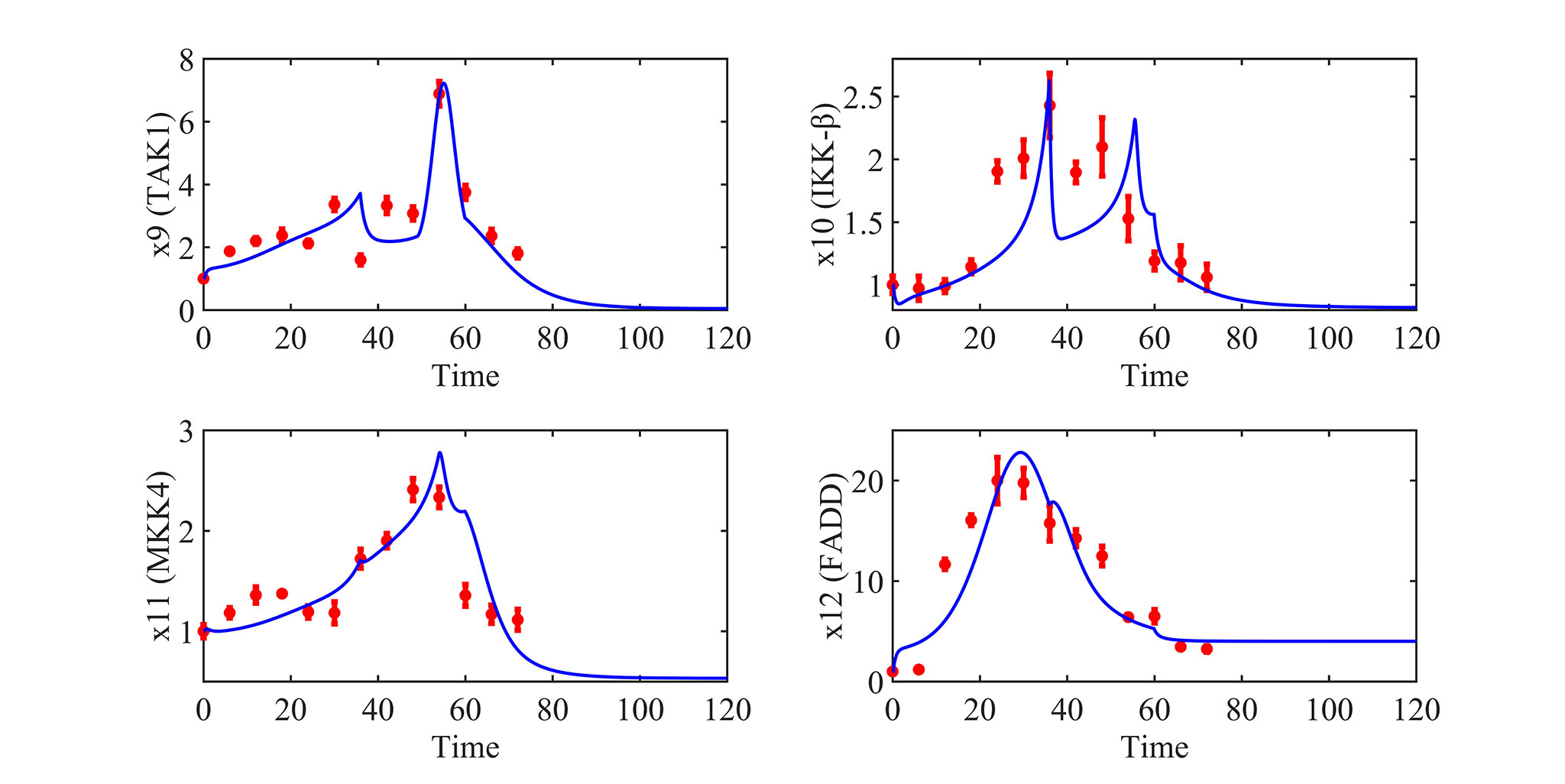

Supplement: Supplementary file 4 [file DataSheet2.zip › Supplementary material_image2/Parameter_g19(大)/3.jpg]

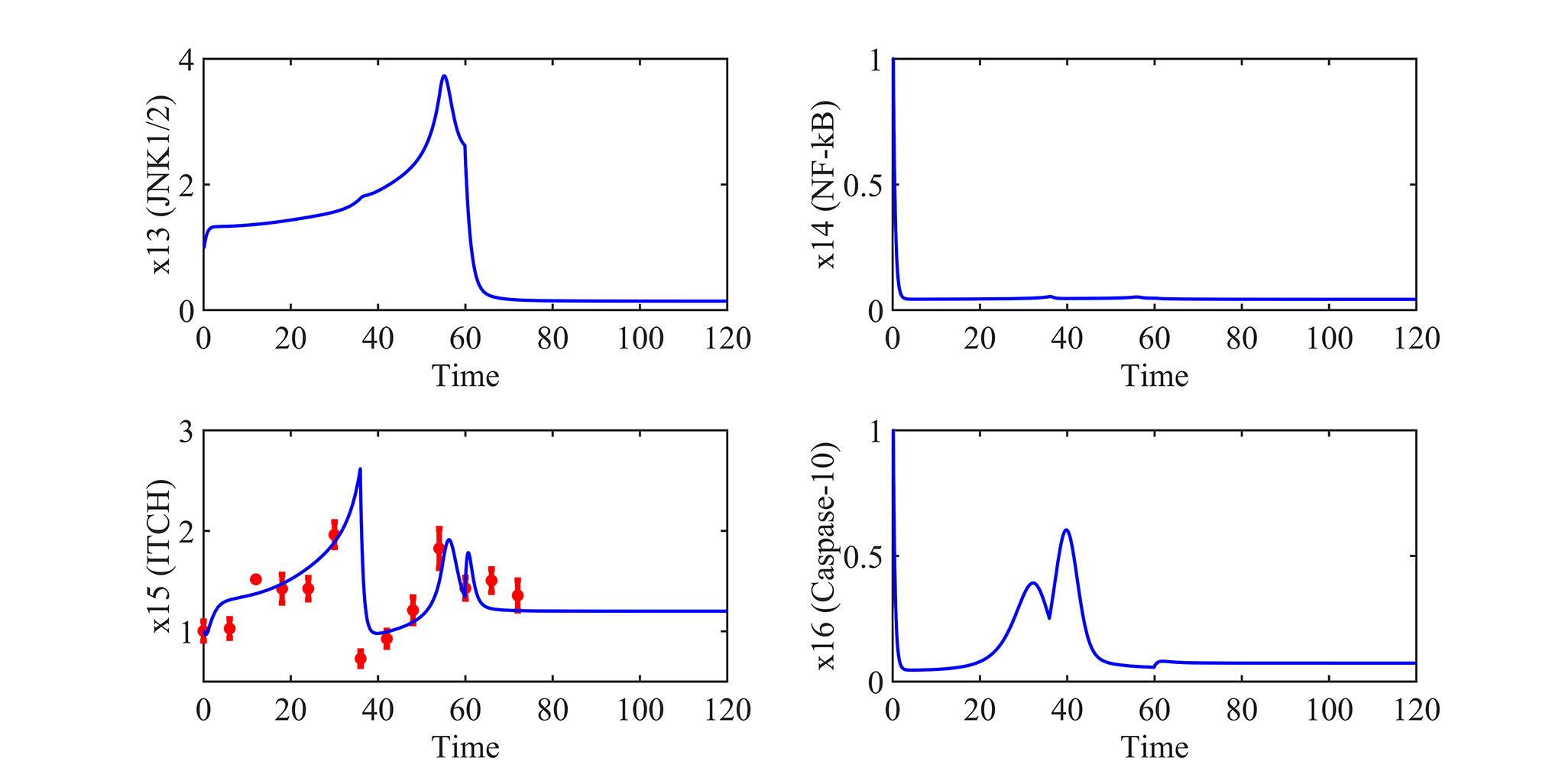

Supplement: Supplementary file 4 [file DataSheet2.zip › Supplementary material_image2/Parameter_g19(大)/4.jpg]

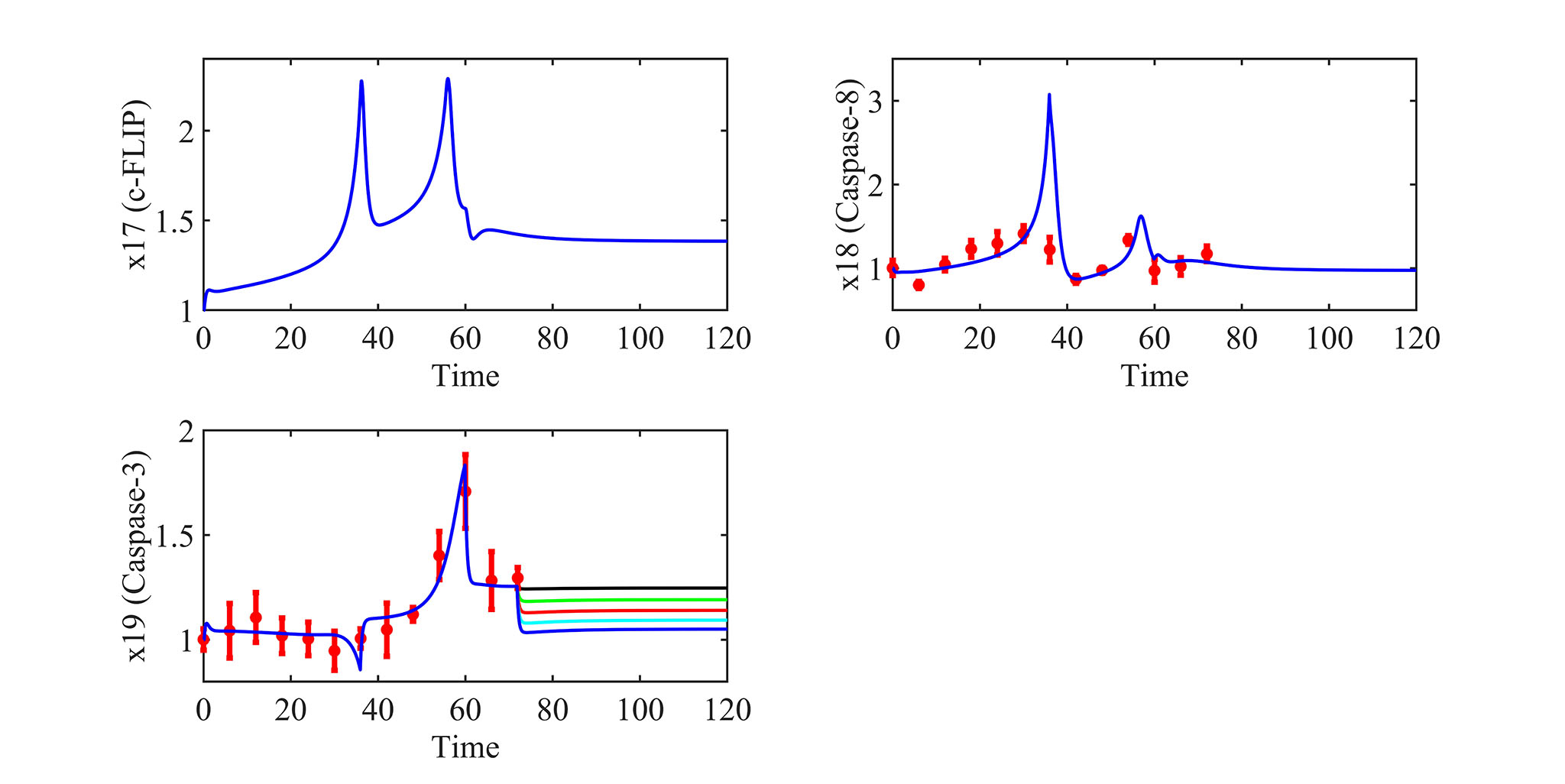

Supplement: Supplementary file 4 [file DataSheet2.zip › Supplementary material_image2/Parameter_g19(大)/5.jpg]
